# Supplementary material for: Stakeholder analysis with regard to a recent European restriction proposal on microplastics
Source: PLoS One. 2020 Jun 22;15(6):e0235062. doi: 10.1371/journal.pone.0235062 (PMC7307934; doi:10.1371/journal.pone.0235062)
Supplement: S2 Table — (DOCX) [file pone.0235062.s003.docx]

S2 Table: Companies categorisation table

| ***Companies*** | | | | | | |
| --- | --- | --- | --- | --- | --- | --- |
| **No**. | **Stakeholder** | **Criteria**  (for statement e.g. economy, innovation, environment etc.) | **Principles**  (separate from criteria e.g. PP, values etc.p) | **Scientific argumentation**  Case Reports & Case Series (observational)  Case-control (observational)  Cohort (observational)  Randomized-controlled trials (experimental)  Systematic review | **Research needs**  (identified needs in statement) | **Other** |
| 1 | ADLER-Werk Lackfabrik Johann Berghofer GmbH & Co KG (Austria) |  |  | Comments from the ADLER-Werk Lackfabrik Johann Berghofer GmbH & Co KG on the scope of the Annex XV restriction report.  Our Company deals successfully for over 80 years particularly in the area of liquid paintings, coatings and wood preservatives. The requirements on these products are continually on the rise, so the restrictions are. On the one hand, the products have to be long lasting, stable and easy to use and on the other hand, they have to have zero emissions (volatile organic compounds) and nearly zero biocide content. To fulfill these criteria polymers are needed, especially for film forming (binders).  The function of binders is to serve film formation by binding the components of paints and coatings with each other and with the substrate. Only binders enable film formation in coatings through polymerisation, polycondensation or polyaddition. Film formation, e.g. drying and hardening, brings about a hard and mechanically resistant layer that adheres to the substrate. Through the physico-chemical process of film formation, binders lose the particle property of microplastics according to the definition under 2 and, consequently, fall under the mentioned rule 5.b. Furthermore, these are firmly incorporated in a polymer structure (binder matrix) by curing, so that they are subject to rule 5.c of the restriction proposal. The share of binders in our compositions varies from about 20 % (paints) up to 80 % (UV-coatings). Small quantities of polymer-based additives (e.g. waxes or spheres) are added to coating materials in order to improve or modify their properties. Additives for paints and coatings are bound in a polymer structure in curing and fall under rule 5.c of the proposal. Additives are used in our paints and coatings in quantities from 2 % to 10%.  With the existing definition of microplastics (REACH Annex XV), the labelling and reporting requirements of the proposed regulation would impact the majority of our 900 different product groups. Based on the current proposal, we estimate at least 50% of one full-time position for a suitably qualified person to fulfill the reporting requirements. This would mean additional costs of at least 100.000 Euro per annum.  The ECHA proposal ignores the fact that we do not obtain details – e.g. on the identity of the polymers used in pre-products (for example, binders and additives) – from our upstream suppliers. The reason is that otherwise confidential business information would need to be disclosed.  Nearly all industrial sites, such as we, have wastewater treatment plants which limit the release of solids. Industrial waste is disposed as hazardous waste, so a direct release into the sewage systems or waters is invariably banned.  Furthermore retailers and consumers are exempted from the reporting requirement (no “downstream users”). Craftspeople are exempted too (no “party placing on the market”). However, especially building paints – which ECHA resorts to for justifying a regulation – are mainly distributed by retailers and used by professional craftspeople (painters, varnishers, plasterers) and consumers. Already for this reason, tracking (“tracking of the identity and quantities of the microplastics used and released to the environment”) – as pursued with the reporting requirement – is factually impossible in the building paint sector.  According to rule no. 7 (labelling requirements), the manufacturers, importers and downstream users responsible for the placing on the market of paints and coatings containing microplastics must ensure that every label and/or safety data sheet includes “instructions for use” to avoid releases of microplastics to the environment.  To conclude, the reporting requirement of proposal no. 8 should not apply to uses at industrial sites (better “industrial installations”, see annex XVII REACH), because (1.) there are already sufficient regulations in place at national and regional level to prevent the release of microplastics, (2.) the reporting requirements as proposed would not be sufficient to achieve the monitoring target. Therefore, the reference in no. 8 to no. 4a should be deleted. Furthermore, the labelling requirements are already implemented in our safety data sheets and therefore we do not need any prescribed phrases on the labels of consumer products. Yet another adaptation of labels hardly offers any advantages so that it is unnecessary. Any change in labelling takes much time and involves disproportionate spending. Existing labels that are no longer fit for use need to be disposed with costs. |  |  |
| **2** | Borregaad AS (Norway) |  |  | Lignosulfonates, or sulfonated lignins, are water-soluble anionic polyelectrolytes, manufactured from the sulfite pulping of wood. In the manufacturing process, the native lignin polymer is hydrolyzed, followed by sulfonation, rendering the lignin water soluble to allow the non-soluble cellulose and the soluble lignosulfonate to be separated by filtration. Lignosulfonates can hence be defined as chemically modified natural polymers.  In the dossier summary of the Annex XV restriction report (ref 1) it is clearly stated that the “intent of the proposed restriction is not to regulate the use of polymers generally, but only where they meet the specific conditions that identify them as being microplastics and where their use will result in releases of microplastics to the environment.” This is further specified in section 1.1.1, where it is stated that for the purposes of the assessment, the submitter of the dossier proposes that “any synthetic polymer (with or without additives) that has the potential to exists as a small (typically microscopic) solid particle in the environment, and which is resistant to (bio)degradation should be considered to be consistent with the concerns associated with the term ‘microplastic’”.  Lignosulfonates are water soluble polymers, which are used in their completely dissolved state when performing their function as dispersants, binders, complexing agents etc. in the formulations and/or applications they are used. Furthermore, due to their complete water solubility, they can never exist as “solid particles” in the environment.  Lignosulfonates will never exist in their particulate form in consumer and professional products at the point of use/disposal, nor can they ever exist as microparticles (as defined in paragraph 2(a)) in the environment. In our view this should exclude them from the scope of the proposed restriction. We find evidence for this in the regulatory definition of a microplastic where it is stated that “polymers that lose their particulate form in solution (e.g. at the point of use/disposal) do not fulfill the definition of microplastic” (ref 2). Moreover, in the ECHA online information session held on April 3 2019, it was clearly stated that “if a polymer is in solution, and not in a solid state, it would not fall within the scope of the restriction” and “soluble polymers which are not in the form of particles do not fulfill the definition of a microplastic” (ref 3).  In the current restriction proposal, solubility of polymers is not taken into consideration, which may leave room for interpretation, e.g. it could be understood that dissolved polymer molecules, which are not considered to be particles in the common sense, will also fall within the scope of the restriction. We therefore call for a clarification in the microplastic definition in the final restriction, where it will be stated specifically that water soluble polymers that are not in the form of solid particles, like lignosulfonates, are excluded from the scope of the restriction.  References  1 Annex XV Restriction Report, intentionally added microplastics (Ver 1.1, 11 March 2019)  2 Annex to the Annex XV Restriction Report (Ver 1.1, 11 March 2019), Section B.1.1.9  3 https://echa.europa.eu/-/information-session-public-consultation-on-microplastics-restriction |  |  |
| **3** | Celanese (Germany) |  |  | Scope or restriction option analysis;  Other socio economic analysis (SEA) issues;  Request for exemption |  |  |
| 4 | Clariant (Germany) |  |  | **Content:**  Hazard or exposure;  Environmental emissions;  Baseline;  Description of analytical methods;  Request for exemption |  |  |
| 5 | Covestro Deutschland AG (Germany) | Covestro is a producer of polymeric raw materials for diverse industries. Following the definitions of the proposed restriction on intentionally added microplastics, polymer pellets and at least some polymer dispersions could fall into the definition. Covestro produces polymer pellets from polycarbonates (PC) and thermoplastic polyurethanes (TPU) which are meant to be converted into articles in extrusion or injection molding processes, and waterborne dispersions containing polyurethanes and/or acrylics, all being film forming in their intended uses.  Polymer pellets are a typical intermediate to transfer polymers between industrial sites which produce polymers (Covestro) and industrial sites which process polymers (DU), e.g. to produce master batches or to transform pellets by melting and subsequent extrusion or injection molding into articles (e.g. headlamp lenses, sheets, ski boots, etc.) Covestro produces PC and TPU. Both polymers are typically not used in single use packaging applications due to properties and price. Instead of that our products are used in applications which aim for lightweight and for longevity of the resulting articles.  Polymer dispersions are successfully used since decades in coatings, printing inks and adhesives and helped to significantly reduce VOC emissions and improve workplace hygiene due to reduction of solvents in coatings and adhesives. Polymer dispersions are produced in industrial sites and they are transferred to other industrial sites (DU) which produce e.g. paints or adhesives using such dispersions among other ingredients. | We believe the proportionality principle is violated and therefore, we call for the exemption of polymer pellets and film forming dispersions and their processing and use within industrial sites. | From the point of view of Covestro all materials which could fall under the criteria for Microplastics according to ECHAs proposal are produced in and transferred between industrial sites which have to comply with tight legal obligations according to wastewater treatment and solid waste being treated mainly as hazardous waste. During regular processing our plants have no emission of intentionally produced “Microplastics” into the environment. Accidental losses would have to be reported to local and regional authorities already according to existing regulations. Covestro actively supports Operation Clean Sweep which is an international program to help prevent plastic pellet losses entering the terrestrial and aquatic environment. The Report 2018 has just been published in March. Due to suitable technical measures and continuous training and awareness raising of our coworkers our plants do not have emissions of Microplastics to the environment.  We call for exemptions from the drafted restriction for pellets and film forming dispersions used within industrial sites, because any waste release from industrial sites is already subject to extensive legal provisions on water protection on national and regional level. There is no environmental hazard which justifies additional reporting requirements beyond the obligations that are already in place.  The definition of “solid” according to CLP is not directly applicable to (polymer) dispersions. Polymers and polymer dispersions do not exhibit a melting point. Instead of that they exhibit glass transition temperatures. Dispersions which exhibit film formation at room temperature or lower must be considered to be liquid because solid particles wouldn’t be able to do so. Therefore, an additional criterion like “particles that cannot alter their shape at 20°C are considered to be solid” could help to sharpen the definition when applied to dispersions.  According to REACH, a polymer is a molecule that contains a sequence of at least 3 monomer units, which are covalently bound to at least one other monomer unit or other reactant. With a C-C single bond length being generally 1.54 angstroms = 0.154 nm, only 7 covalently bound carbon atoms in molecule’s backbone (7 x 0.154 = 1.078 nm) would qualify such small molecules as Microplastics in case they contain 3 monomer units among the 7 carbons. It is analytically impossible to distinguish a naturally occurring C8 to C12 hydrocarbon from any molecular residue resulting from i.e. polyethylene. Even a naturally occurring stearic acid would already be bigger than 1 nm.  Analytically, the lower limit proposed by ECHA is not enforceable. Enforceability needs limits that could be proven in mixtures (i.e. paints, adhesives) with available analytics. Just as an example, the identification of Microplastics with µ-FTIR in the environment already reaches limits at 5 – 10 µm due to scattering at surfaces of particles. With µ-RAMAN spectroscopy 1-2 µm could be achieved. (Analyst, 2009, 134, 1586; Baseman, Microplastics Analyses in European Waters 2019, p. 18) Both methods are not standard in analytical laboratories. On the Microplastics conference 2018, only a few studies were published where authors claimed to be able to identify the chemical basis for particles in the range of 500 nm. So, 1 nm is far away from any kind of standard analytics. At 1 nm it is impossible to distinguish between a medium sized organic molecule of any kind and residues of polymers; let alone determining the chemical identity of a potentially present polymer.  So for both reasons, size of molecules and analytics, we call for a realistic lower size limit which fits at least to polymers and is not just arbitrary. Since we are talking about “micro”plastics, we would call for 1 µm as lower limit.  Proportionality  It is well known, that the main “entry path” of Microplastics to the environment are secondary microplastics generated from decomposing plastic waste, tire abrasion and laundering of synthetic clothing. The planned restriction will have no effect on such emissions into the environment.  ECHA estimates 400 kt emission reduction of microplastics in 20 years (equals 20 kt/a) through the planned restriction at estimated costs of €9.4 bn. This corresponds to only ca. 0.2 wt-% of total amount of improperly handled plastic waste in the EU.  As described above for industrial sites, emissions are already regulated and controlled by regional and local authorities under existing regulations. On the other hand, retail and professional users will not be obliged to report even though this stands for almost half of the dispersions paint market and a potential source for possible losses into the drain.  So, a lot of effort and cost is burdened to industrial sites which exhibit the lowest contribution to the described problem. Therefore, most of this measure is not targeted to the issue and monitoring of the efficacy of the restriction based on this approach is not possible.  Furthermore, a transition time of EiF + 12 month for reporting means in practice no transition time at all, because 12 month after EiF everybody has to report on the past 12 month. So, depending on the detail-level required for reporting, any kind of possibly necessary system adaptation to purpose is not scheduled.  Covestro acknowledges the call for (bio-)degradable polymers in cases where microplastics are inevitably released into the environment.  We consider the OECD 301 method as being appropriate to assess cosmetic ingredients biodegradability for several reasons. First the method is a screening method for the polymer in a cosmetic product in water (ii) the method is known and already used by cosmetic industry, so data are already available (iii) we have had reliable results even with poorly soluble substances, as opposed to the 302 method that gave un-logical results .  • BUT: The 28 days time-frame is too short for polymers to have a reasonable chance to reach the 60% threshold.  • This single threshold does not allow differentiating between polymers with different biodegradation behavior. It is only a “yes” or “no” classification. Because there are so few readily biodegradable alternatives on the market, the result could well be that most of the polymers will fall into the ‘not biodegradable’ category. At the same time, there are current alternatives in the market that are well biodegradable; hence a big improvement on the persistent polymers used today, but just not reaching the 60% threshold.  • Therefore, we would suggest introducing a new threshold. Based on our results, we would suggest a threshold of 20 to 30% which would already be a significant improvement over benchmark (mostly all persistent) while being achievable therefore also promoting innovation and switch to better biodegradable materials.  Considering dispersions, Covestro is not aware of any application in coatings or adhesives where such a low amount of an insoluble powder exhibits intended technical effects. No meaningful effect is ever seen below 0.1 %. (e.g. for matting)  Applying unreasonable low limits creates problems with existing analytics in mixtures and compromises enforceability of the regulation. |  |  |
| 6 | DAW SE (Germany) | Wir unterstützen vollinhaltlich die Position des Verbandes der Deutschen Lack- und Druckfarbenindustrie e.V. (VdL), die wir als Anlage hinzugefügt haben. Als größter privater Europäischer Farbenhersteller mit einem Jahresumsatz von ca. 1,3 Mrd. € verleihen wir unserer tiefen Besorgnis über die geplante, unverhältnismäßige Beschränkung Ausdruck. |  |  |  |  |
| 7 | Evonik Industries AG (Germany) | Evonik Industries AG would like to express some general comments and concerns relating to the restriction proposal on intentionally added microplastics. We agree that the REACH regulation is an appropriate regulatory tool to address risk related to chemicals EU-wide. However, we believe that the current Annex XV proposal for a restriction on intentionally added microplastics does not meet the REACH regulation requirements, and as a result would not meet the intended objective.  Our comments on the Annex XV restriction report focus on issues with relevance for the Company Group and its diverse business. In addition subsidiaries of the Group will provide detailed comments and information focusing on products and applications specific to their business as this is the best way to provide ECHA with the information needed. Comments will be provided successively during the commenting phase.  Comments and concerns  1. The public consultation on this proposed restriction officially started on 20/03/2019 and ends on 20/09/2019. It is unclear to us that there is a deadline for verification of the scope and conclusion on hazard in the June meeting of RAC corresponding to a deadline for sending comments on these topics by latest 20th May 2019. All comments that are sent until 20/09/2019 should be taken into consideration by RAC and SEAC.  2. Polymers in general are addressed as a generic group of substances in the draft restriction dossier. According to our understanding of the REACH regulation substances to be restricted have to be identified individually in order to prepare a sound risk assessment. “An Annex XV dossier has to provide sufficient information to support the restriction of all substances covered by the proposal.” (Guidance for the preparation of an Annex XV dossier for restrictions). We acknowledge that there are cases for group-based approaches, e.g. for mercury, chromium and lead compounds and PFOA, its salts and related substances. In these cases a limited amount of substances is grouped and the individual substances have some principal criteria and properties in common. Therefore these examples do not justify the very huge scope of the present restriction proposal on intentionally added microplastics.  4. The scientific evidence that is compiled in the Annex XV dossier is contradictory and inconsistent and thus does not justify the application of the precautionary principle.  5. The costs of the restriction are indicated to be approximately €9.4 billion. These costs seem to be completely disproportionate to a reduction of approximately 0.2% of the total plastic waste that is disposed without proper control (0.2% in the form of intentionally added microplastics that would fall under the scope of the restriction dossier).  6. It is not acceptable that a detailed labelling and an extensive annual reporting requirement are to be introduced for all polymer-containing products even if they are exempted from the restriction. Such obligations have to be fulfilled along the supply chain leading to extensive workload. Moreover there is no sufficient legal basis for this.  According to the above mentioned comments Evonik Industries AG would like to suggest that the restriction dossier as presented should be reviewed and revised.  The following adjustments should be implemented:  - increase of the lower limit up to 1µm  - increase of the concentration limit for polymer containing particles from 1% w/w to a concentration where the polymer content is relevant for the properties of the whole particle  - increase of the concentration limit from 0,01% w/w to 1% w/w  - the requirements of the restriction dossier should be enforceable especially with regards to suitable analytical methods for analysis  - water-soluble polymers should be excluded  In general Evonik Industries AG suggests to include clear indicative uses into the restriction dossier comparable to the concept as it has been presented in the “Note on substance identification and potential scope of a restriction on uses of microplastics” (Version 1 11/07/2018).  Furthermore the restriction dossier should reflect the opinion of the Scientific Advisory Mechanism (SAPEA). |  | 7. It is stated that polymers shall not be placed on the market as a substance on its own or in a mixture as a microplastic in a concentration equal to or greater than 0.01% w/w. This concentration limit is even lower than 0.1% w/w for PBT and vPvB substances. The justification for lowering the concentration limit to 0.01% w/w seems to be arbitrary.  8. It is stated that microplastics are considered to be similar to PBT/vPvB substances and that they should be treated as a non-threshold substances for the purpose of risk assessment. The approaches for the risk assessment as described in the draft restriction dossier are solely based on the “extreme” persistence of microplastics. The REACH regulation does not foresee a restriction that is solely based on persistence. The property of extreme persistence does also not justify the use of the “case-by-case”-risk assessment approach since persistence is already covered by the legal text.  9. Within the restriction dossier almost all considerations regarding substance identity and risk evaluation are based on “microplastics.” Microplastics cannot be considered as a substance according to the REACH regulation Art. 3(1). Furthermore the term “microplastics” is not in compliance with the requirements of the Guidance for the preparation of an Annex XV dossier for restrictions, especially Annex I and II, as here detailed information on the identity of a substance are requested. Finally the title of the draft restriction report is mentioning intentionally added microplastics as a substance but in fact the restriction is focusing on polymers as defined under the REACH regulation. But not every polymer is a plastic and not every plastic falls under the definition for polymers under the REACH regulation. Thus the definition and the scope of what is restricted is not clear.  10. The lower limit for polymer-containing particles of 1nm doesn’t seem to be reasonable. This is already the size range of single molecules. According to the current state of technology there are no analytical methods and techniques available that would allow the qualitative and quantitative analysis of particles in this size range. Therefore, it is questionable how the restriction requirements can be enforced.  11. Water-soluble polymers fall under the current definition for microplastics as well as non-water-soluble polymers. Currently there do not exist appropriate analytical methods to determine water-soluble polymers. It is therefore unclear how water-soluble polymers should be monitored regarding the restriction. Furthermore the original request from the European Commission excluded water-soluble polymers explicitly. | 3. Hazards and risks are not clearly identified on a scientific basis. In several parts of the restriction dossier there are comments on the lack of a clear hazard and/or risk, e.g. “incomplete information on the risks arising from exposure to these materials is currently available…”, “…but only limited evidence that risks are likely to be occurring in the environment;”, “..there is currently insufficient evidence to fully assess these risks.” |  |
| 8 | GE Healthcare Life Sciences (Sweden) | Our Company would like to provide comments on socio-economic impacts of the restriction proposal as well as comments/suggestions regarding Paragraphs 4, 5, 7 and 8 of the restriction proposal.  See attached non-confidential attachement. |  |  |  |  |
| 9 | Geberit International AG (Switzerland) | The globally operating Geberit Group is a European leader in the field of sanitary products. Geberit operates with a strong local presence in most European countries, providing unique added value when it comes to sanitary technology and bathroom ceramics. Whilst Geberit supports the need to minimize emissions of waste, we do not support the current Annex XV proposal for a restriction of intentionally added microplastics and would like to comment specifically. |  | 1. Non-conformities to requirements of the REACH regulations  REACH is a substance-based regulation. Therefore, a precise and individual identification of substances to be restricted on their own, in a mixture or incorporated into an article is mandatory. The Annex XV restriction proposal violates this principle by general addressing of polymers or microplastics as a generic group of substances. This approach has to be regarded as a precedent that is in danger of diluting the principles that underpin REACH and goes against the provisions of the REACH regulation. The provisions of Title VIII of REACH are further disregarded by proposing a restriction in the absence of a hazard and/or a risk specific to the microplastics themselves. Quite the contrary, in several parts of the restriction dossier it is explicitly referred to the lack of a clear hazard and/or risk. With the sole reference to the persistence of microplastic particles an alleged risk is constructed that does not legitimate the proposed measures. As the above statement implies the scientific evidence provided to justify the proposed restriction does not meet the standards required on the application of the precautionary principle.  2. Technical comments:  The lower limit for polymer-containing particles of 1 nm is not reasonable. This size range will include nano-sized particles and even single molecules. According to the current state of technology there are no analytical methods and techniques available for industry that would allow the qualitative and quantitative analysis of particles in this size range.  3. Requirements that arise for producers of plastic articles:  On the formal level a detailed labelling and an extensive annual reporting requirement for polymer-based microparticles even though they are exempted from the restriction do not have a sufficient legal basis. At technical level microplastics in terms of plastic raw materials are industrially transformed into plastic items and then cannot be intentionally released into the environment anymore. Any releases would be purely accidental. In addition to the fact that the financial value of plastic raw materials will limit unintended waste inherently the reporting on pellet loss cannot bring meaningful results. Taking into account the low levels of plastic losses compared to the overall production the information gathered by following the requirements of the restriction will not lead to a crucial gain of information compared to what is known today. In view of the questionable usefulness reporting requirements for plastic resin producers and processors will create a disproportionate administrative burden throughout the supply chain. Moreover, the request for polymer identity information will have Confidential Business Information implications. In the light of the above arguments, plastic raw materials should not be regulated under the proposed REACH restriction for intentionally added microplastics. Instead of focusing on recording very low and difficult to evaluate unintended releases the focus should be put on preventing releases. With this awareness major plastic resin producers and processors have already committed to reduce pellet losses occurring during the transportation and handling of resin pellets under Operation Clean Sweep® which involves voluntary labelling, and this is also recognized in the “European Strategy for Plastics in a circular economy”. |  |  |
| 10 | GlaxoSmithKline plc (United Kingdom) | GSK is a science-led global healthcare Company. We discover, develop and manufacture innovative pharmaceutical medicines, vaccines and consumer healthcare products . Every day, millions of patients and consumers across the world use our products. In 2018, we delivered around 2.3 billion packs of medicine, 770 million vaccine doses and 3.8 billion consumer healthcare products.  In GSK we continuously strive to reduce the environmental impact of our products and we welcome European efforts to reduce marine plastic litter, as we share public concern about the long-term environmental impact from microplastics.  As stated by the European Commission in the recently published communication on Pharmaceuticals in the Environment , it should be an overriding principle that medicines, medical devices and healthcare products should remain available to patients who need them.  Actions to address the problem from microplastics in the environment must not jeopardise access to safe and effective pharmaceutical treatments, medical devices and healthcare products across Europe. We therefore encourage that ECHA takes into consideration the health benefits of potentially impacted products and assess if the recommended actions may in any way compromise access to healthcare products. We welcome the proposed derogation for medicines and would encourage ECHA to consider a similar derogation for medical devices, due to the role medical devices play in disease prevention and in the treatment of medical conditions.  Medicinal products for human use  We welcome the derogation of medicinal products for human use from the restriction of intentionally added microplastics.  According to ECHA’s restriction proposal the ‘package leaflet’ of medicines shall contain relevant instruction for use to avoid releases of ‘microplastics’ to the environment, including at waste lifecycle stage. In line with European Pharmaceutical law, medicinal products already have disposal instructions written in the package leaflet. Therefore, we support the position of EFPIA and AESGP that no revised package leaflet or any other additional labelling of medicinal products is needed. We support the ambition of ECHA to raise awareness on how to dispose unused and expired medicines appropriately in Europe, however we do not believe that an additional label requirement may be the most efficient way to achieve this. It is estimated that 5% or more of pharmaceuticals and healthcare products are not being disposed of properly. Industry has therefore engaged in a campaign called #medsdisposal encouraging proper disposal.  We would like to stress that the mandatory reporting proposed by ECHA would introduce an extensive set of reporting requirements, creating significant additional administrative burdens. This would be resource intensive and not negligible as considered by ECHA.  Medical devices  We welcome the derogation of medicinal products for human use from the restriction of intentionally added microplastics, and we encourage that a derogation for medical devices is also considered. Both medicines and medical devices contribute to improved healthcare across Europe. We therefore support the request by MedTech Europe and AESGP to ask for an exemption for medical devices, as this would not significantly impact on ECHA’s overall aim of reducing the release of microplastics to the environment.  Medical devices play an important role in providing healthcare for citizens across Europe. Medical devices cover a very broad field of products, from pacemakers and hospital beds, inhalers for respiratory diseases, autoinjectors, safety syringes and substance-based medical devices. From the impact assessments in both the Annex to the Annex XV Restriction Report and in the Annex XV Restriction Report it does not seem as ECHA has considered the wide range of medical devices, including substance based medical devices. Table 27 in the Annex XV Restriction Report on the impact assessment only refers to industrial and professional application of medical devices such as in hospitals, whereas many other types of medical devices are used daily by patients and consumers in their own homes across Europe to prevent or treat medical conditions.  Polymers in substance based medical devices are used for various reasons, such as therapeutic reasons and in some cases, polymers play a critical role in the product formulation. When using polymers in healthcare products, they have been assessed by various regulatory bodies as being safe for human use and have in many cases been used in humans for decades, why we would have a good understanding of their safety profiles. For some of the alternatives available, we may not have the same extensive knowledge about their safety profiles when used in healthcare products.  Substance based medical devices are required to meet high regulatory requirements. Finding alternatives with similar functionalities and safety profiles will be challenging. Furthermore, changing the formulation of medical devices requires efficacy testing and the Medical Device dossier needs to be audited for certification by a Notified Body and potentially a new registration is needed in other parts of the world. This takes time and could potentially lead to supply shortages.  Cosmetic products  In GSK we do not have microbeads of plastic in our products. The European cosmetics industry has voluntarily phased out as much as 97.6% of plastic microbeads used for exfoliating and cleansing purposes in wash-off cosmetic and personal care products.  Whilst we understand that every action is beneficial to reduce the amount of microplastics entering the environment, studies have estimated potential contribution of the European cosmetics and personal care sector to be between 0.1 % and 2 % . We support the opinion of Cosmetics Europe that leave-on cosmetics, are disproportionately impacted given the high socio-economic impact.  Importantly, many cosmetic products also provide critical public health benefits; for example, daily oral hygiene products to protect against caries and gum disease, sunscreens to prevent skin cancer and anti-dandruff shampoo for those people suffering from dandruff and seborrheic dermatitis. We encourage that ECHA in their impact assessments take into consideration that some cosmetic products are delivering significant public health benefits. |  | Proportionality  According to the European Commission it is estimated that between 75 000 and 300 000 tonnes of microplastics are released into the environment each year in the EU. The largest contributors to microplastic emissions are from the breakdown of bigger plastics, for example car tyres, road marking, plastic pellets and fibres from clothes . In comparison, according to ECHA, it is estimated that around 270 kg of microplastics is released annually from medical devices.  ECHA’s definition of microplastics  ECHA’s proposed definition of microplastics is very broad. Plastic is not defined in the report by ECHA, but ECHA uses the definition of polymers (REACH Art 3.5). ECHA’s proposed definition of microplastic comprises all solid polymers at ambient conditions with a particle size smaller than 5 mm in all dimensions. Not subject to restrictions are polymers which are naturally occurring and not chemically modified, and (bio)degradable polymers according to interim criteria set out in the Annex XV dossier.  The definition proposed by ECHA means that the use of synthetic polymers in medicinal products for human use is derogated from ban, however labelling and reporting is required.  Furthermore, the approach by ECHA with an assessment of a group of substances in a generic manner i.e. polymers with very different properties and behaviours makes the restriction difficult to understand, difficult to interpret, and ultimately it will make it difficult to enforce in a harmonised way across Europe.  The 5B derogation  This derogation refers to microplastic where the physical properties of the microplastic are permanently modified. We believe this derogation would profit from clearer wording and examples to clarify the interpretation of this derogation, i.e. to derogate polymers that no longer fulfil the meaning of a microplastic given in paragraph 2(a) when entering the aquatic environment, as when in water they can no longer be considered a solid particle. | Polymers play an important role in various healthcare products and in many cases alternatives to these polymers may not be available or may not be able to fulfil the safety criteria’s, or the same functions in the product.  As the EU is the first regulatory body in the world to address all microplastics intentionally added in products, GSK believes it is of critical importance that ECHA’s definition and scope of microplastics is appropriate, that there are alternatives available, and that health benefits and the contribution of these products to public health is taken into consideration. The potential for unintended consequences and adverse impacts on the healthcare of the population across Europe is significant if such an approach is not taken.  Eunomia , an independent research organisation focusing on the sources and impacts of waste in the marine environment, has recommended that regulatory actions should address marine litter in the areas where it will be most effective:  “As policy makers, campaigners and the public increase their awareness of this topic, it is important that action to address marine litter is focused in the areas where it will be most effective” .  We would therefore like to stress that health benefits and the contribution of these products to public health must be taken into consideration when regulatory actions to reduce microplastics are being recommended, to avoid unintended consequences and adverse impacts on the healthcare of the population across Europe.  Conclusion  GSK welcomes the ambition of the European Union and ECHA to reduce marine plastic litter and microplastics in the environment. As a healthcare Company, we believe it is critical that any action taken is proportionate and appropriate, recognising the important role that medicines, medical devices and other healthcare products play in preventing and treating medical conditions. We welcome the proposed derogation for medicines and would encourage ECHA to consider a similar derogation for medical devices, due to the role medical devices play in disease prevention and in the treatment of medical conditions.  We are available to provide any further information and answer any questions you may have. |  |
| 11 | Henkel AG & Co KGaA (Germany) | The current definition of microplastics and its application in the restriction proposal appears not to be focused. By lacking the property of water solubility polymeric substances could lead to reporting and labelling requirements, although not contributing to the concern of solid microplastic in the environment.  The water solubility/extractability of polymers is a crucial parameter to distinguish solid microplastic from dissolved polymeric matter and, thus, provides the key parameter for the ECHA restriction proposal. Water solubility/extractability of polymers can effectively be determined via OECD TG 120 and Commission Regulation (EC) No. 440/2008 A.20. Thereby, complex polymers can be assessed using adequate analytical strategies to quantify the water-soluble part, i.e. the dissolved part on the molecular level, in the extracted fraction.  “Solubility” should therefore be included as an element in the regulatory definition. |  |  |  |  |
| 12 | I.R.C.A. SERVICE Company (Italy) | The I.R.C.A. SERVICE Company works in the biocide and crop protection field, using several production technologies to make all kind of liquid formulations. We know both the pros and cons of each, so we can propose the best solutions to our customers. We perform several kind of microencapsulated products, from biocide to Crop Protection Products, even fragrances and perfumes.  We believe that with the restriction of micro plastics one of this kind of formulation , Microencapsulation, will be ruled out, causing a lost in the rate cost /benefit and we try to explain it considering environmental pros and cons. In the meantime we are also explaining why this technology will clearly disappear and why the transitional period is too short and doesn’t consider some critical aspects such as stability and authorization time.  MICROENCAPSULATION WILL GET LOST  Of course this are sensational words, but not far from the reality in our opinion, if referred to biocide or crop protection products at least.  According to the proposed draft, the biodegradation of a polymer has to be tested measuring the CO2 produced by a biological effect. It makes no sense to use a biodegradable polymer when the Active ingredient won’t permit a biodegradation. Let us explain it by an example:  For the biodegradability in soil 100g of polymer are required  • You could require to test the polymer in REAL conditions , then -together with 100g of microcapsules- there would be 1 kg of pesticide. Result: None Biodegradation would be possible, none polymer will pass the test, none Company will start any kind of research.  • You could require to test the polymer in IDEAL conditions (ignoring that for these two categories it encapsulates a pesticide), Result: Someone could spend money for the research of biodegradable film/capsules and for biodegradation test. The results wouldn’t represent the real condition and it could be a way to keep up appearances.  But Is the biodegradability the real goal? Isn’t it the accumulation in the environment?  PROS  The graphic shows the number of rainy days in Lombardy ITALY ATT1  The graphic shows the concentration of PM10 in the air ATT2  Th3 graphic shows the number of days when the limit of PM10 is exceeded ATT3 |  | Microencapsulated products (in biocide and CPP) are made with interfacial polymerisation, active ingredients inside in an a-polar means and water outside.  The active ingredients are mainly solid or viscous liquids , thus they need to be dissolved in solvents. The capsules must not be dissolved neither by heavy solvent (for ex Solvesso 200) nor by water (external part), so it is hard to find a general solution allowing a fast degradation. Furthermore, we need to consider that, according to the proposed restrictions, the degradation required is to CO2 and water (complete degradation). For sure, we can suppose that for some active ingredients, “it could be possible to find a Biodegradable polymer but we need to remember that “biodegradable “ polymer has contained pesticide, fungicide, insecticide or herbicide. It will really perform the same degradation of the study that one Company will produce encapsulating soybean oil? “. Take into consideration that his kind of products need to be registered following international standards and thus the quality and the stability of the product but even of the formulation are very strict. That is why a wild range microencapsulation technology wouldn’t be possible. It will take out the confidence with this technology and step by step could disappear.  Take for instance the what could happen with a biocide product: a biocide product dossier is 150 000-200 000€. In case of a biodegradable microencapsulation technology, the cost of biodegradability test for the capsule is over 40000 € in soil and about 20000€ in water . So we are pushed for other kind of formulations.  WHAT KIND OF PROS WILL BE LOST  In the last few years it has become more and more important to reduce the quantity of active ingredients dispersed in the environment. Both for biocide products and Crop Protection Products the environmental risk assessment is then the most critical point of the registration.  Microencapsulation allows to release the active ingredients over time and thus the reduction of the dosage.  Even more important, in the last few months we have been contacted by several companies that tried to find eco-friendly solution with the replacement of the Traditional Active ingredients with more “natural“ ones leading to a much lower environmental impact. The problem of these actives (natural pirethrum, geraniol …) is the stability over time. The natural pirethrum has a problem of activity, It has an efficacy of hours, microencapsules surely improve the efficacy over time. Geraniol is a repellent also included in the biocide list , but it is affected by hydrolysis. Microencapsulation will give this active ingredients a reasonable shelf life. The restriction will allow not to disperd micro plastic (mainly inert and “potentially” dangerous) in the environment but we will disperd more pesticide (not inert and surely heavily dangerous). We need to consider that on the microencapsulation the rate of micro plastic vs active ingredient is 1:10 – 1:20.  WHY THE RESTRICTION OF MICROPLASTICS FROM MICROENCAPSULATION IN BIOCIDAL AND CROP PROTECTION PRODUCTS WILL WORSEN THE ENVIROMENTAL CONDITION  Both fields (biocide products and CPP product) are strongly regulated and both kind of products need an environmental risk assessment that defines the dosage.  On these defined dosage it is possible to make an analysis and mostly none insecticide registered as BIOCIDE is allowed for external use.  The formulation of microencapsulation in these fields is more expensive than other kind of productions and its use can be justified if having concrete pros. The pros required is long term release. This means a longer activity of the product and then the reduction of the dosage.  From the definition of micro plastics, PM10 should be partially considered micro plastics themselves, and the parts that cannot be considered “micro plastic” have the same physical properties but more dangerous chemical properties ( no degradation and more dangerous classification).  If we consider 100m of column of air for each square meter of soil; 35 μg/m^3 as mean and we consider hust 60 days of rains, this means : 35 μg/m^3 x 100m x 60 day/year= 0,21g/m^2/year  A typical dosage of a pesticide in field is 1 l/ha, the typical concentration is 100g/l of active, the typical rate Polimer: Active ingredient is 1:10- 1:20 =10g/l of polimer  It means: 10g (of polimer) / 10 000m^2/y =0,001 g/m^2/year  The microparticles coming from direct rain on EACH square meter (of ground) is 200-400 times higher than the contribution of the single square meter TREATED by a pesticide.  The reduction of micro plastics / micro particles in the environment is not substantial.  ACCUMULATION IN THE ENVIRONMENT  Almost all the microencapsulated products for CPP and Biocides are made of polyurethane walls. The degradation of the polymer is given by oxidation and photo-degradation; only when the polymer is small, it can be degraded by bacteria or fungi. The microbial degradation will take time in particular if the wall is “protected” by the action of the active it has contained. But as recognised in literature,if the oxidation and photo degradation –being superficial phenomenos - are not significant in a “macro” scale they are relevant in the “micro” scale.  In microencapsulated products, the degradation of the wall is required: the active has to be released in 0-6 weeks. The wall in this period needs to crack otherwise the product doesn’t come out.  If we suppose a crack (not a biodegradation) every 2 months and we suppose it is linear ( while it’s likely to be exponential) the dimension of the wall could change as follows:  TAB 1  During this time no biodegradation should happen, but just a degradation.  Furthermore if we should consider that a total biodegradation could happen in 10 years,  the accumulation in the environment should be:  0,001g/m^2 /years * 10 years / 2 ( factor of linear reduction) = 0,005 g/m^2  This is the equivalent to 1 day of rain with 50 PM10 ( 50μg/m^3*100m=0,005g/m^2)  If ever we consider 20 years, the accumulation should be comparable to two rainy days.  In conclusion, even in the worst condition, the contribution of Biocidal and CPP on accumulation of micro plastics in the environment isn’t relevant  CONS  If no significant pros are coming with this restriction, the concrete con is the following: more pesticides will be used.  First we need to remember the ratio between a.i. and polymer wall : 10 AI /1 Wall  If microencapsulation allows to have a longer time release of around 10 % ,compared to not microencapsulated product, we could save the same quantity of active ingredient as the polymer used.The difference is that the Active ingredients are usually much more dangerous than the micro plastics.  The real aim of microencapsulation is to increase the efficacy time of 20%, 50% , or even 100%.  This means that for every 1g of capsule wall, we could save from 2g to 10g of pesticide.  TRANSITIONAL PERIOD TIME  The transitional period time of 5 years for the restriction can’t be considered sufficient for the following dead lines:  2 years of research and development  +  2 - 3 years of stability (especially if the polymer is biodegradable, it is not reasonable any kind of accelerated stability test)  +  2 years test of biodegradation in soil  +  2/3 years of authorization  ____________________________________________________________  8-10 years are required to complete the process of authorization  CONCLUSIONS  The pros coming from the restrictions on biocide and Crop Protection Products are not significant:  • The accumulation of micro plastics in the soil should be comparable to 1-2 rainy days, even in the worst conditions  • The improvement of microencapsulation technology would be just academic, because no test would be performed with the real concentration of the active ingredient.  • The dosage and the directions for use are indicated and even limited in the dossier of the products; a complete risk assessment is made for more dangerous substances and it could be integrated. It could be required even the evidence of the effective pros of microencapsulation.  • The transitional period wouldn’t be enough to complete the authorization process.  The environmental dispross are heavy:  • Increase of consume of active ingredient (at the same efficacy)  FOR CROP PROTECTION PRODUCT AND BIOCIDES  PROS  The graphic shows the number of rainy days in Lombardy ITALY ATT1  The graphic shows the concentration of PM10 in the air ATT2  Th3 graphic shows the number of days when the limit of PM10 is exceeded ATT3    From the definition of micro plastics, PM10 should be partially considered micro plastics themselves, and the parts that cannot be considered “micro plastic” have the same physical properties but more dangerous chemical properties ( no degradation and more dangerous classification).  If we consider 100m of column of air for each square meter of soil; 35 μg/m^3 as mean and we consider hust 60 days of rains, this means : 35 μg/m^3 x 100m x 60 day/year= 0,21g/m^2/year  A typical dosage of a pesticide in field is 1 l/ha, the typical concentration is 100g/l of active, the typical rate Polimer: Active ingredient is 1:10- 1:20 =10g/l of polimer  It means: 10g (of polimer) / 10 000m^2/y =0,001 g/m^2/year  The microparticles coming from direct rain on EACH square meter (of ground) is 200-400 times higher than the contribution of the single square meter TREATED by a pesticide.  The reduction of micro plastics / micro particles in the environment is not substantial.  ACCUMULATION IN THE ENVIRONMENT  Almost all the microencapsulated products for CPP and Biocides are made of polyurethane walls. The degradation of the polymer is given by oxidation and photo-degradation; only when the polymer is small, it can be degraded by bacteria or fungi. The microbial degradation will take time in particular if the wall is “protected” by the action of the active it has contained. But as recognised in literature,if the oxidation and photo degradation –being superficial phenomenos - are not significant in a “macro” scale they are relevant in the “micro” scale.  In microencapsulated products, the degradation of the wall is required: the active has to be released in 0-6 weeks. The wall in this period needs to crack otherwise the product doesn’t come out.  If we suppose a crack (not a biodegradation) every 2 months and we suppose it is linear ( while it’s likely to be exponential) the dimension of the wall could change as follows:  TAB 1  During this time no biodegradation should happen, but just a degradation.  Furthermore if we should consider that a total biodegradation could happen in 10 years,  the accumulation in the environment should be:  0,001g/m^2 /years * 10 years / 2 ( factor of linear reduction) = 0,005 g/m^2  This is the equivalent to 1 day of rain with 50 PM10 ( 50μg/m^3*100m=0,005g/m^2)  If ever we consider 20 years, the accumulation should be comparable to two rainy days.  In conclusion, even in the worst condition, the contribution of Biocidal and CPP on accumulation of micro plastics in the environment isn’t relevant  CONS  If no significant pros are coming with this restriction, the concrete con is the following: more pesticides will be used.  First we need to remember the ratio between a.i. and polymer wall : 10 AI /1 Wall  If microencapsulation allows to have a longer time release of around 10 % ,compared to not microencapsulated product, we could save the same quantity of active ingredient as the polymer used.The difference is that the Active ingredients are usually much more dangerous than the micro plastics.  The real aim of microencapsulation is to increase the efficacy time of 20%, 50% , or even 100%.  This means that for every 1g of capsule wall, we could save from 2g to 10g of pesticide. | We know that the restriction of micro plastics could bring generally benefits, but we are worried about Biocide and Crop Protection Products, whose cons are much higher than pros.  Basically, microencapsulation is chosen if providing the reduction of the dosage of an active ingredient. We will compare the pros of the restriction of micro plastics with the cons.  BIODEGRADABLE PLASTIC IN EVERY CONDITION?  The first point that this restriction has to consider is that Biocides and Crop Protection Products are not simple products, but pesticides. Their nature could affect (or block) the biodegradation. Will a pesticide (contained in the microcapsule) affect the biodegradation performance of the plastic? Shall the biodegradability of the plastic be tested with the active ingredient or not? |  |
| 13 | Iceland UK |  |  |  | Ian Schofield says: ”some potential alternatives, such as bio-based otoxy-biodegradable polymers, including polyethylene furanoate (PEF) and polylactic acid (PLA). However, he said, as a packaging technologist, "I’m finding it extremely difficult to introduce some of these materials, because no one can prove to me that they don’t break down into microplastics." The main barrier, in his view, is that a proven marine environment test for materials, that shows how they break down in the ocean, is not yet available. "This is something we need fast. (Stringer, 2018). |  |
| 14 | Lenzing AG | **Answer to specific info request 1:**  In general, we welcome the criterion of biodegradability in Paragraph 3(b) (2.2.1.2 Table 19, Derogations) for derogation from the scope of the Restriction, to allow innovation which can provide solutions to microplastic pollution. |  | **Comment:**  Clarification for 2 product categories manufactured by Lenzing.  1) Wood-Based (regenerated) Cellulose fibres: See our note in the attachment.  LENZING™ fibers with the generic fibre types viscose, modal, and lyocell consist of pure cellulose, a polymer that occurs in nature, that has not been chemically modified, as described in derogation paragraph 3a) in ECHA 2019, Annex XV Restriction Report – Microplastics of March 20th 2019 (2.2.1.2 Derogations, Table 19, Derogations from the scope of the proposed restriction). As such our standard fiber and powder products are biodegradable in a range of natural environments, and offer an alternative solution to plastics in many applications (e.g. wipes, cosmetics, construction materials).  2) Magnesium lignosulphonates are water-soluble polymers, originated during the magnesium bisulfite pulping of wood. These are typically marketed by Lenzing as an aqueous solution.  We would like to call for a clarification regarding the definition of “microplastics” and clearly exclude lignosulphonates from the scope of the restriction, since due to their water solubility they cannot exist as particles in the environment. A more detailed argumentation will be sent by the European forest fibre and paper industry (CEPI).” | We would like to call for a clarification that regenerated cellulose products are derogated from the scope. |  |
| 15 | LyondellBasell Industries N.V. (Netherlands) | We believe that Microplastics which are ‘fully consumed’ during their use and therefore cannot be intentionally released to the environment should be exempted from labelling and reporting requirements. Our comment mainly concerns to the Subgroup A defined as:  Subgroup A: coated raw materials, pre-production pellets and masterbatches, currently included under derogation “5(b)”.  Language in derogation 5b refers to Substances or mixtures containing microplastic where the physical properties of the microplastic are permanently modified when the substance or mixture is used such that the polymers no longer fulfil the meaning of a microplastic. As the proposed restriction is focused on the uncontrolled release of microplastic particles into the environment, we request polymers under 5b and belonging to the subgroup A to be exempted from the labelling and reporting requirements.  Labelling  For cases where the microplastics are already handled or disposed appropriately there should be no need for additional controls measures such as additional obligatory labelling.  It is well-known that substances falling under the subgroup A, will cease to be microplastics, once they are used in downstream plants and any release that could be associated with these types of microplastics would be purely accidental.  • Accidental releases of substances under subgroup A are well controlled and already regulated. Emissions to air are controlled under the Ambient Air Quality Directive and the Industrial Emissions Directive, wastewater emissions are controlled under the Wastewater Treatment Directive and solid waste emissions under the EU waste legislation.  • Major plastic resin producers including our Company have already committed to reduce significant pellet losses occurring during the transportation and handling of resin pellets under Operation Clean Sweep® which involves voluntary labelling and this is also recognized in the “European Strategy for Plastics in a circular economy”.  • For the substances included under Subgroup A, instructions regarding handling and safe-disposal are clearly included in the product specific safety data sheets.  In conclusion these mitigation measures are adequate to inform the user about the risk to environment from accidental releases and any further labelling requirements under this restriction is superfluous.  Cost of Labelling  ECHA’s assumption that cost of labelling is “negligible” is incorrect since pre-production pellets are not classified today according to CLP – so this new requirement will require creation of SDSs and/or re-design the packaging to incorporate labelling. Since the incremental administrative burdens of labelling are unknown at this stage, it cannot be concluded as “negligible”. The administrative cost of labelling could be significant if this needs to be applied on individual packaging modes of all shapes/sizes and in different languages. A more fundamental question is, “why should companies placing microplastics belonging to the Subgroup A be asked to comply with burdensome regulations without providing a clear justification of the benefits behind the requirements? |  | Reporting  Compared to labelling, these requirements for importers and downstream users are more disconcerting. The proposed reporting requirements on identification of polymers, uses, quantity used and released to the environment are redundant for intent of this Restriction.  • Identification of microplastics would involve in many cases disclosure of proprietary information which needs to be safeguarded via a construction of confidentially agreements. This would create significant administrative burden throughout the supply chain without proportional added value.  • The uses of Microplastics involved in the subgroup A are already well known to Regulatory bodies as what matters most for this restriction is the use as in 5(b) - where the physical properties of the microplastic are permanently modified when the substance or mixture is used such that the polymers no longer fulfil the meaning of a microplastic. It is not clear why the current know-how is not adequate and what additional reports are required to cover the gap.  • Reporting of quantities on an annual basis would result in double and triple counting if Company M produces masterbatch, then ships to Company P which transforms it into a final product or pellets, which is then sold to Company C to be transformed into a final product. In this case, companies M, P and C would have to report. Supply chains could be longer than this, with materials being shipped in and out of the EU throughout the value chain.  • Reporting of volumes that may potentially be released into the environment, based on estimation vs. measured data, would result in data that is not harmonized. Analytical methods for detecting microplastics in products and measure spillage are yet to be agreed. A standardization effort for detecting microplastics is still under development and thus not available for industry at this stage.  • It is impossible to identify, quantify and report on nano-forms of microplastics.  • Proposed reporting of polymers volumes as such would be not in line with REACH where reporting is done based on chemical substance level under REACH.  • Disclosing the quantity of microplastics needs to fully comply with EU competition law and requires the necessary confidentiality safeguards.  Conclusion  Polymers that are transformed along the production process and no longer appear in the form of microplastics in the final product (article) intended for downstream uses, should be exempt from the reporting and labelling obligations (e.g. raw materials, including pellets, masterbatches, additives). |  |  |
| 16 | Merck KGaA (Germany) |  |  |  | Hazard or exposure;  Environmental emissions;  Other socio economic analysis (SEA) issues;  Request for exemption |  |
| 17 | Melos GmbH | **Content:**  Scope or restriction option analysis;  Baseline;  Request for exemption |  |  |  |  |
| 18 | Polytan gmbH (Germany) |  |  | Scope or restriction option analysis;  Information on costs;  Other socio economic analysis (SEA) issues;  Request for exemption |  |  |
| 19 | Proctor & Gamble (United States) | We have provided comments on the proposed Biodegradability Derogation 3 b) in the Annex XV Restriction Report for Intentionally Added Microplastics. We are in agreement with the breadth of methods proposed for evaluating the biodegradability of microplastics. The proposed criteria and corresponding level of stringency offers reassurance that any microplastic that meets the criteria are truly not part of the “microplastics issue” (i.e. accumulative and persistent in the environment).  P&G experts on fate and biodegradation support these general comments:  Derogation 3b on biodegradability is fit for purpose; however, the recommended modifications described herein are critical for a swift implementation of these criteria.  Biodegradability criteria stability - Considering the derogation’s level of stringency, it is vital to avoid in short term further changes to the criteria that result in greater stringency. This would be damaging to any progress made over the allotted transition period and would impede the ability of ECHA and EU Commission to introduce a proportionate and fit for purpose restriction.  Longer transitional periods needed - ECHA and the EU Commission should consider longer transition periods, as this will allow time to adapt to this groundbreaking restriction while curbing the associated economic disruption. P&G recommends that ECHA consider extending transition periods an additional 5 years to support innovation.  The reality is that, although the proposed derogation is flexible, it is also very stringent. It will take significant time to develop potential alternatives and provide proof that the alternatives meet the biodegradation derogation. If testing spans multiple tiers of the biodegradation derogation and the tiers are followed in sequence, it could take several years to evaluate one potential alternative. The development of a suitable alternative will require dozens of iterations. Nevertheless, P&G will work to understand the biodegradability of current microplastics and where needed, develop and assess alternatives.  In addition to the above points, P&G has summarized the following recommendations below:  1. Derogation be considered following a weight of evidence approach specified in existing ECHA guidance documents;  2. Extension of enhanced ready biodegradation test duration from 60 days to 90 days due to microplastic’s physiochemical properties;  3. Changing of the language in Tier 4 from, “and” to “or” to signify that only one method and pass result is needed;  4. Allow modifications in simulation tests as radiolabeling and cold analytical techniques to track parent compound and metabolites are limited for microplastics;  5. Flexibility on the form of the material to be tested as the chemistry limits the testing of the actual commercial form in many cases (e.g. case study on encapsulation); and  6. Change laboratory accreditation to, “ISO 17025 and/or GLP” to increase lab options as few labs offer all proposed methods. |  |  |  |  |
| 20 | RENECAL (Spain) | RENECAL IS IN TOTALLY AGREEMENT WITH ETRMA INPUT DONE IN THIS CONSULTATION |  |  |  |  |
| 21 | Schmitz Foam Products (Netherlands) | We think that there are enough alternatives for the use of microplastics as an infill for synthetic turf fields.  We are a mayor player in the building of sport fields with synthetic turf. We provide shock and drain pads made of recycled PEX foam for about 500 fields per year. We think that there are enough alternatives for the use of SBR or TPE (microplastics) as infill for synthetic turf sport fields. There are a lot of systems (fields) with natural infills, non infills or sand only infills. We see the industry is clearly moving to less SBR (microplastics) as an infill, and it is possible to make synthetic turf fields with natural infills. (wood chips, cork, etc.) Also it is very easy to reduce microplastics as an infill by using shock pads for sport fields. |  |  |  |  |
| 22 | SIGNUS ECOVALOR (Spain) | We support and agree with the ETRMA input done at this consultation |  |  |  |  |
| 23 | Stamicarbon (Netherlands) | we believe that due to the ban on polymer coatings on fertilizers, a major target to increase the efficiency of fertilizers and the reduction of the losses to the environment will be seriously hampered. the use of polymer coated, controlled-release fertilizers is one of the few means to substantially increase the nutrient use efficiency, and to decrease the loss of nutrients to the environment, hence to reduce the current pressure of nutrients on the environment, whether it be as water pollution or as green house gas emission. the ban will seriously hamper the attempts to reduce such environmental pressures. |  | the currently used polymer for polymer coating of fertilizers is left in the field after application in very small amounts, and will degrade in a timespan which is much faster that polyolefin materials which is the main concern by authorities. furthermore being used on the field, they remain on the field and will not pollute waterways, oceans, etc. given their slow degradation they will build up only to moderate levels. they will degrade within a timespan of approx. 10 years, though not as fast as the current proposed 4 years. furthermore they provide a solution to the current problem of increasing greenhouse gas emissions and surface water pollution due to the increasing use of conventional fertilizers, have a rather low nutrient use efficiency. the polymer coated fertilizers offer an opportunity to significantly increase the efficiency and reduce to losses to the environment. there is no biodegradable polymer alternative available and hence the environmental pressure will continue to exist, will only further increase due to the forecasted increase of fertilizer use. accumulation of the current conventional polymer system is only moderate, due to the low amounts used per year/hectare and the slow degradation of the current polymer system. |  |  |
| 24 | Troy Chemical Company BV (Netherlands) | Troy Corporation is a medium sized Company, active in Europe for many years, which develops and manufactures performance materials for the adhesives, paints and coatings, metal-working fluid and wood industries. These materials are used and available in over 100 countries to produce high performing, cost-effective, and sustainable products. Troy's objective is to provide superior products and technology for the needs of customers, drawing on knowledge and expertise gained from serving markets worldwide. Troy produces over 400 preservatives, performance additives, and metal carboxylates at modern manufacturing facilities on 3 continents. Troy supports customers with worldwide sales offices, distributors, warehouses, and logistics facilities.  Troy’s concerns in connection with the proposed restriction on intentionally added microplastics, focuses on two areas of concern. A failure to satisfy the requirements of:    • legal certainty regarding the scope of the restriction; and  • requirements of Article 68 and 69 of the REACH Regulation in connection with risk assessment in general, and for biocides, in particular.  We reserve the right to comment on other aspects of the proposal later on during the consultation window.  Scope of the Restriction  Restrictions in REACH need to be adopted in respect of a specifically identified substance or group of substances (with a rationale for inclusion of each substance in the group). It is clear that the Annex XV report takes an approach which is goes against the clear language of REACH – by characterising a group of undefined substances i.e. all polymers meeting broad criteria. We note that by defining the scope of the proposed restriction proposal in these terms, all future polymers would appear to fall under this restriction automatically. This open-ended class of substances is used as a marker to restrict substances which might be considered to be similar to each other. Accordingly, the principle of legal certainty, which requires that “EU rules enable those concerned to know precisely the extent of the obligations which are imposed on them and that those persons must be able to ascertain unequivocally what their rights and obligations are and take steps accordingly” seems to have been contravened by the proposed restriction proposal. | Risk Assessment  In the Annex XV dossier proposal, it is recognised that microplastics do not present a hazard that can be identified by reference to recognized hazard categories. In this regard the scope of the Annex XV applies to a class of substances – polymers, defined by their physical form. Physico-chemical characteristics are intrinsic properties of a substance, insofar as they are determined by their chemical identity. Otherwise, the particle form is not an intrinsic property which can trigger classification because of a hazard. Since no hazard recognized by EU legislation can be identified, there can be no assessment of exposure to microplastics, or risks posed by microplastics, according to risk assessment procedure established by EU courts. | As regards biocides specifically, we would draw attention to the fact that capsule suspension biocides cannot be placed on the market without a very long and rigorous risk assessment undertaken under the Biocidal products Regulation 528/2012. While the encapsulate materials are not part of active substances, the evaluation process - in particular the concerns for the environment and human health as assessed under the BPR - is undertaken with proper emphasis on the controlled release based on efficacious low level availability of free biocidal active substance(s) due to encapsulation. All ongoing or approved active substances/biocidal products employing encapsulation technology based on polymeric materials with a physical form that might be considered to meet the microplastics definition of the restriction proposal have been assessed by EU risk assessors. Therefore, the application of a general claim of unacceptable risk posed by these encapsulated biocides undermines the whole risk assessment process under the BPR. These issues are best addressed under the substance and product specific regime established by the BPR, rather than imposing a blanket ban which ignores the lex specialis regime. The overall environmental benefits of encapsulation are can also be taken into account under the BPR’s assessment.  If, contrary to our view, the encapsulation of biocides would remain within the scope of the microplastics restriction, the transitional period for biocidal applications should be significantly extended to take into consideration the approval/authorisation timelines of biocides in general, which differs significantly from a number of other approval/authorisation regimes for non-biocidal use regimes identified in the scope of the Annex XV dossier. |  |  |
| 25 | Worlée-Chemie GmbH (Germany) | Worlée is a family owned Company that manufactures chemical raw materials for construction chemicals, paints, coatings and printing inks. The Company also produces raw materials for cosmetic applications in decorative and skin care cosmetics. Our portfolio mostly contains polymer-based raw materials such as acrylate dispersions, alkyd emulsions or polyesters. The proposed labelling and the extensive reporting requirements involve a large amount of bureaucracy.  Even though film-forming polymers and hydrogels are exempted from the ban according to paragraph 5(b), labelling as microplastics according to paragraph 7 and reporting to paragraph 8 is required. Actually, it can be assumed that manufacturers of cosmetics will no longer buy our raw materials because our products will be stigmatized as microplastics.  Therefore, it would be helpful to include the exemptions for film-forming polymers and hydrogels into paragraph 3. Thus, labelling and reporting would not be necessary and our products would not be stigmatized as microplastics.  In our opinion it is not useful to prepare a list of polymers that may or may not be impacted by the proposed restriction. Neither the polymer name (as defined by industry) nor the INCI name provide information about the physical state, particle size and biodegradability or whether it is a film-forming polymer or a hydrogel. One INCI name can be used for many different polymers and some of these polymers may be microplastics and others may not. Thus, for example, the INCI name Acrylates Copolymer could stand for a particulate, not biodegradable polymer that would be a microplastic or it could describe a soluble, non-particulate and biodegradable polymer which would not be considered as a microplastic.  Therefore the preparation and subsequent publication of such a list (Annex F. Appendix D.1 Table 88) is misleading. As a consequence, manufacturers of cosmetics as well as consumers will consider all listed polymers as microplastics which is definitely not the case. |  | First of all, it is a matter of common knowledge that the suggested test methods in Section 2.2.1.6 of the Annex XV report (Table 21 1-3 – Appendix X) are not designed for polymers and in general the laboratories are unexperienced handling hardly soluble and insoluble polymers as test item. It needs to be discussed scientifically which test methods are suitable for polymers to obtain reliable results. One should consider that there are significant differences between soluble, hardly soluble and insoluble polymers.  Our Company already commissioned different studies (OECD 301 B or F) to assess the ready biodegradation and/or enhanced/modified biodegradation of some polymers. The conclusion is, that the assessment of such studies is challenging and the results are often inconclusive especially in case of insoluble polymers. It is our experience that OECD 301 F seems to be not suitable for polymers that are insoluble in water. Additionally, there are no specific limits defined to decide whether the polymer is soluble, hardly soluble or insoluble. Moreover, it is the case that these figures vary depending on the chosen test method. | In the context of manpower and costs, the proposed requirements are extremely challenging. Especially the proposed ban on microplastics in cosmetics has a great impact on our cosmetic raw material portfolio with unpredictable consequences.  The proposed microplastics definition in paragraph 2 is too general and difficult to apply concerning polymer dispersions and there are some outstanding questions:  Are polymers, synthesized by emulsion polymerization and dispersed in an aqueous solution, covered by the definition of microplastics?  Are any methods available to determine whether the dispersed polymer is solid (paragraph 2(e)) or liquid (paragraph 2(g)) without changing the physico-chemical properties of the initial dispersed polymer?  Disclosure of confidential business information:  According to paragraph 8 every downstream user subject to at least one of the exemptions outlined in paragraph 4(a), 4(b), 5(b) and 5(c) is obliged to report certain information to ECHA including the identity of the polymer(s). It is a matter of fact that most downstream users (e.g. paint formulators) do not have any detailed information about the identity of non-classified polymers. This information is only available at the manufacturer’s level. Disclosing it to industrial downstream users or to professional users would certainly have a large negative impact on CBI protection and competitiveness – especially for those enterprises manufacturing specialty chemicals.  Paragraph 8 does not provide any details which information about the chemical identity is required. Should a CAS number be provided we would like to outline:  • CAS registration numbers (RN) are not mandatory and thus not every polymer placed on the market has a CAS RN.  • A CAS RN covers various chemically similar polymers, which may differ in terms of molecular weight, monomer ratio, production parameters, biodegradability, water solubility etc.  In case a reporting obligation for microplastics should be maintained it has to be done at the polymer manufacturer’s level and it has to be clearly defined, which parameters are suitable and required to clearly identify a specific polymer. |  |

**References**

ECHA, 2019, General Comments and answers to specific information requests, Helsinki: European Chemicals Agency, Link: <https://echa.europa.eu/registry-of-restriction-intentions/-/dislist/details/0b0236e18244cd73> - accessed 28-10-2019

Stringer, L., 2018, Feature: HCF 2018 ponders plastic, ChemicalWatch, Link: https://chemicalwatch-com.proxy.findit.dtu.dk/66766/feature-hcf-2018-ponders-plastic?q=microPlastics - accessed 14-8-2019.
