# Supplementary material for: Stakeholder analysis with regard to a recent European restriction proposal on microplastics
Source: PLoS One. 2020 Jun 22;15(6):e0235062. doi: 10.1371/journal.pone.0235062 (PMC7307934; doi:10.1371/journal.pone.0235062)
Supplement: S3 Table — (DOCX) [file pone.0235062.s004.docx]

S3 Table: Industry and trade associations’ categorisation table

| ***Industry and trade associations*** | | | | | | |
| --- | --- | --- | --- | --- | --- | --- |
| **No**. | **Stakeholder** | **Criteria**  (for statement e.g. economy, innovation, environment etc.) | **Principles**  (separate from criteria e.g. PP, values etc.p) | **Scientific argumentation**  Case Reports & Case Series (observational)  Case-control (observational)  Cohort (observational)  Randomized-controlled trials (experimental)  Systematic review | **Research needs**  (identified needs in statement) | **Other** |
| 1 | A.I.S.E., International Association for Soaps, Detergents and Maintenance Products | A.I.S.E. comments on this topic are provided in the attachment ‘Preliminary Socio-Economic Analysis of the impacts of the proposed REACH Restriction on Intentionally added Microplastics on the detergents and maintenance products sector’ submitted via this form. |  |  |  |  |
| 2 | American Chamber of Commerce to the European Union | **Content:**  Scope or restriction option analysis;  Hazard or exposure;  Description of analytical methods;  Information on alternatives;  Information on costs;  Other socio economic analysis (SEA) issues |  |  |  |  |
| 3 | American Chemistry Council | We have information discussing analytical methods that could be used to detect and quantify MPs in the products above. |  |  |  |  |
| 4 | AnimalhealthEurope | AnimalhealthEurope position on an EU proposal for restricting Microplastics  1 Background  End of January 2019, ECHA published an Annex XV restriction proposal with regard to intentionally added microplastic in consumer and professional products and its impact on the environment.  Restrictions are normally applied to limit or ban the manufacture, placing on the market (including imports) or use of a substance, and can impose additional requirements such as technical measures or specific labels.  ECHA’s definition of microplastic is very broad. Plastic is not defined as such, and ECHA uses the definition of polymers (REACH Art 3.5). The use of synthetic polymers in medicinal products for human and veterinary use is derogated from ban, but intricate labelling and reporting is required.  With this paper, AnimalhealthEurope would like to outline its position on ECHA’s Annex XV proposed restriction of intentional use, reporting and labelling of microplastic and the implication it could have for the pharmaceutical industry.  2.2 Reporting  ECHA´s draft restriction proposal requests reporting of the used excipients, which are considered as microplastics under the ECHA definition. Such reporting is required to be performed on an annual basis and includes: the identity, the use and quantity of the polymer, and the estimated quantity released to the environment. In AnimalhealthEurope point of view, this raises a high bureaucratic burden for the pharmaceutical sector without any benefit to the patient or environment. Volumes of polymeric excipients are expected to remain the same over time due to their importance for e.g. oral dosage forms. Polymers have been essential to the formulation technology of medicinal products for decades. New excipients on the market for tablets are limited and any additions will most likely only have minor structural edits by cross linking of existing polymers or differently modified cellulose backbones but all fall under the broad definition used for microplastics.  2.3 Labelling  According to the EU legislation on medicinal products for Human & Veterinary use - Eudralex, the package leaflet contains a note on disposal of unused medicinal product. This information covers the labelling needs described in ECHA’s restriction proposal.  AnimalhealthEurope therefore concludes that no revised package leaflet or any other additional labelling of medicinal products is needed.  3 Conclusions  The Pharmaceutical sector uses a variety of synthetic polymers e.g. derivatized celluloses, which would fall under the current very broad definition of microplastics by ECHA. These excipients, which are proven safe and would be difficult to replace, are critical to ensuring the uninterrupted supply of high quality and efficacious medicines of importance to patients.  We consider it inaccurate to define every polymer with a size < 5 mm as a microplastic. This definition would even include oligonucleotides, polysaccharides and peptides, when being chemically treated during manufacture. Even microcrystalline cellulose, as being chemically treated during the manufacturing process and commonly used in galenics, would fall under the definition of microplastic, while chemically untreated cellulose, also used in galenics, would not fall under this definition.  The key points and requests AnimalhealthEurope would like to make to ECHA are:  - We strongly support the derogation of medicinal products from the REACH restriction.  - We believe the definition of microplastics, whereby it includes common polymeric excipients, would benefit from a refinement of the scope/definition.  - The requirements for reporting and labelling of medicinal products will entail a high bureaucratic burden for companies without a beneficial impact on society or the environment. To this point we ask that you support our proposal whereby current medicinal product labelling regulations are adequate to meet the requirements of ECHA’s restriction proposal and the medicinal product derogation should extend to reporting requirements. |  | 2 Impact on the veterinary pharmaceutical industry AnimalhealthEurope Position  ECHA’s proposed definition of microplastic comprises all solid polymers at ambient conditions with a particle size smaller than 5 mm in all dimensions. Not subject to the restriction are naturally occurring, not chemically modified polymers, and (bio)degradable polymers according to interim criteria set out in the Annex XV dossier.  2.1 Use of Synthetic Polymers in Medicinal Products  ECHA’s broad definition of microplastic puts a significant number of excipients used in the pharmaceutical industry and listed in the European Pharmacopeia into scope of the restriction (e.g. cellulose acetate, hydroxypropylcellulose, polyvinylpolypyrrolidone, hydroxypropylmethylcellulose phthalate, polymethacrylates, polyethylenglycol, and microcrystalline cellulose). Excipients are essential constituents in the formulation of medicinal products.  The European Pharmacopoeia includes an adopted list of excipients which are approved and safe for use in drug products; these are polymers in many cases. Excipients listed in pharmacopoeias show a good safety profile with regard to human or animal health and are comprehensively tested in accordance with the required safety studies for approval of drug products.  Furthermore, under European pharmaceutical law, the use of excipients depicted in monograph(s) of the pharmacopoeias, e.g., but not limited to, European Pharmacopoeia, is favoured. The requirements for the submission and approval of drug products are regulated in the EU guidelines and processes overseen by the European Medicines Agency (EMA) and health authorities of the EU member states which include proven safety and efficacy. Formulating a drug product is a complex task in which different factors like drug transport, drug release, uniformity (of content), hardness, and also shelf life have to be considered, as well as patient (human and animal) acceptance. The formulations of certain active pharmaceutical ingredients (API) need compensation of undesired physicochemical properties in order to improve the producibility, pharmacokinetic profile and the therapeutic effect. The use of polymers is very broad in medicinal products as they provide solutions to various API specific properties. There are limited alternatives to exchange polymers as excipients in medicinal products and the alternatives are not suitable for most formulations due to their inability to mimic the specific and necessary properties as outlined above. Therefore, the vast majority of solid oral dosage forms contain polymers.  Thus, changing the composition and formulation of a drug product once approved is an extremely involved process, in many cases substituting may not be possible due to changes in bioavailability &/or efficacy of the API. However, where changes may be viable extensive studies would be necessary which would take years to perform and may include bioequivalence studies, in addition to approval of variation of one or multiple authorities responsible for drug product approval in respective markets.  To conclude, AnimalhealthEurope would like to stress that a restriction on the use of polymers will affect most solid form drug products for human or veterinary use, and potentially other dosage forms. This would have severe impact on the availability of drugs in Europe, and patients’ safety. As such, AnimalhealthEurope welcomes the derogation of medicinal products for human and veterinary use from the restriction of intentionally added microplastics. |  |  |
| **5** | Association of the European Self-Medication Industry - AESGP | Scope or restriction option analysis;  Environmental emissions;  Information on alternatives;  Information on costs;  Other socio economic analysis (SEA) issues;  Transitional period;  Request for exemption |  |  |  |  |
| **6** | BAH e.V. | Definition of microplastics:  The proposed definition of microplastics is very broad and is not generally accepted. Definition of microplastic has to be precised, because not all polymers (in the relevant size) are microplastics (every plastic is a polymer but not every polymer is a plastic). Furthermore, no standardised methods are known for the qualitative and quantitative analysis of microparticles in the environment and diverse products.  Transitional period:  Medical devices are used in a large scale in the EU and most other regions of the world regulated. In the EU the Medical Device Regulation (MDR, Regulation 2017/745 of April 5, 2017) requests in many places that a risk management according to EN ISO 14971: 2012 Medical devices - Application of risk management to medical devices (ISO 14971:2007, Corrected version 2007-10-01) must be in place before any medical device can be brought on the market. This standard mentions in the introduction and many other places that all environmental risks must be considered and reduced to the minimum possible before placing on the market: “Risks can be related to injury or damage, primarily to the patient, but also to the operator, other persons, data, property, other equipment and the environment.” Based on these requests medical devices can be regarded to be similarly safe for the environment as medicinal products.  In case where an exemption would be granted, it has to be noticed that Medical Device Regulation (MDR, Regulation 2017/745 of April 5, 2017) requests in many places that a risk management according to EN ISO 14971: 2012 Medical devices - Application of risk management to medical devices (ISO 14971:2007, Corrected version 2007-10-01) must be in place before any medical device can be brought on the market. This standard mentions in the introduction and many other places that all environmental risks must be considered and reduced to the minimum possible before placing on the market: “Risks can be related to injury or damage, primarily to the patient, but also to the operator, other persons, data, property, other equipment and the environment.” Based on this request medical devices can be regarded to be similarly safe for the environment as medicinal products.  In addition, the MDR regulation (Regulation (EU) 2017/745 of the European Parliament and oft the Council of 5 April 2017 on medical devices,…) provides in Annex I (General safety and Performance Requirements) in 14.7 requirements with respect to waste and its safe disposal and also the requirement that “such procedures shall be described in the instruction for use”. In general, medical device have an instruction for use (IfU), and details of information in the instruction for use for safe disposal of the medical device are defined in Annex I in 23.4 (v) (and it is also defined that this information should be available upon request even an information of use is not required according to Annex I 23.1 (d)). In addition, in Annex I 23. detailed requirements for label and IfU are stipulated.  This means medical devices under MDR regulation have already detailed information in the IfU available, and therefore an additional labelling (paragraph 7) for medical devices derogated from paragraph 1 of Annex XV Restriction report for intentionally added microplastic based on paragraphs 4(a), 4(b) or 5 is not necessary and appropriate.  Additionally, as mentioned in the Annex to the Annex XV Report on Microplastic medical devices containing polymeric particles have only industrial or professional uses. This is true as well for the dental materials. Substance based medical devices even if used by consumers are used under a regime similar to medicinal products.  Based on these considerations we would ask for an exemption of medical devices similar to the exemption given for medicinal products (Restriction Report, Table 3, 4b).  As far as no exemption is given for any reason, we would ask for a prolongation of the entering in force for medical devices of EiF + 6 years based on following considerations:  First, medical devices cover a very broad field of products, e.g. substance-based medical devices, wheelchair, pacemaker. For some products, e.g. dental base materials, substance-based medical devices, there are no alternatives for synthetic polymers used. In the rare case an alternative is available, any change of formulation of medical devices requires a new conformity evaluation (e.g. generation of pre-clinical data, animal tests and clinical data, risk management) and potentially a new registration in other parts of the world. This takes time and could lead to supply shortages.  Secondly, manufacturers of medical devices are currently implementing the new Medical Device Regulation (regulation (EC) 2017/745, MDR) which is applicable on 26 May 2020. The implementation of the new MDR regulation is a huge challenge for the whole medical devices industry, and also for the notified bodies, till 27 May 2024. For now, there are not enough notified bodies certified to comply with the MDR. There is also the need to take into account the MDR transitional provisions. Indeed, according to Article 120 paragraph 2 MDR certificates issued by Notified Bodies in accordance with the Medical device Directive (MDD) shall remain valid until the end of the period indicated on the certificate, which shall not exceed five years from its issuance and shall however become void at the latest on 27 May 2024. Manufacturers of medical devices with a MDD certificate will then have to implement the MDR requirements.  For these reasons, a derogation of 2 years for this restriction is not realistic. More realistic would be a derogation of at least 6 years after entry in force (as for ‘leave-on’ cosmetic products), so that manufacturers can have the time to change the formulation of their products and also to implement the MDR requirements (taking also into account manufacturers of medical devices with a MDD certificate which have a transitional period according to MDR).  For the same reasons, a derogation of [EIF +18 months] (point 7, table 3) for medical devices falling under the exemptions of point 5 (table 3) is largely insufficient and would need to be extended to at least [EIF +3 years] after the first label/IFU updates under the MDR has been completed.  **Answer to specific info request 1:**  Beside the tiered approach mentioned above, all other data and approaches, that show that the polymer is not persistent should be used (e.g. for medical devices ISO 10993-13:2010, Biological evaluation of medical devices – Part 13: Identification and quantification of degradation products from polymeric medical devices, in combination with ISO 10993-9: 2009, Biological evaluation of medical devices -- Part 9: Framework for identification and quantification of potential degradation products and their corresponding ISO EN standards).  **Answer to specific info request 5:**  a. Tonnages of microplastics used:  Although medicinal products are excused from the restriction (Paragraph 1) a comprehensive reporting for these products is expected by ECHA.  The reporting requirements in Paragraph 8 state that companies placing a microplastic derogated from Paragraph 1 on the market shall send to ECHA in the format required by Article 111 of REACH, by 31 January of each calendar year:  (a) The identity of the polymer(s) used in the previous year  (b) a description of the use of the microplastic  (c) the quantity of microplastics used in previous year, and  (d) the quantity of microplastics released to the environment either estimated or measured in the previous year  ECHA shall publish a report summarising the information received by 31 March every year.  In our opinion point (d) is not that easy to estimate, because it is not known for most of the polymers, which content is actually released unchanged into the environment.  An analytical measurement would be desired, but in various chapters of the Restriction Dossier it is stated that there is a lack of standard analytical methods for the analysis of microplastics in the environment. This results in the need to develop and validate methods for the analysis of microplastics in the environment, where that's a real challenge in the short of time.  Furthermore, there are no criteria published how ECHA would assess the reported information and how/if they will draw a conclusion.  This reporting requirement means a high additional burden to Pharmaceutical Companies, where a benefit of a reporting is not apparent.  Technical function:  Usually, medicinal products consist of one (or more) active substances and of various excipients which give their function to the respective dosage form. With the help of a certain selection of excipients, the optimal therapeutic efficacy can be generated for an active ingredient. With the help of certain excipients, the release of solid oral dosage forms can also be controlled, i.e. solid oral dosage forms can be generated with immediate and controlled release.  Due to the broad definition of microplastics, many excipients used for immediate and controlled release dosage forms fall within the scope of this restriction.  It seemed that excipients for immediate release have initially not been in the focus of the restriction, but since excipients can be used for different functions in one dosage form, they will fall under the restriction although this is not intended (e.g. ethylcellulose can be used both in coatings and as disintegrant).  The Dossier Submitter needs to be aware that immediate release formulations are no “synthetic polymer free” alternatives to controlled release formulations.  Releases to the environment (incl. pathways):  Uncoated and coated solid oral dosage forms are typically degraded within the gastro intestinal tract to release the API. The degraded substances are usually excreted.  b. We know from the past, in connection with the implementation of new labelling requirements, that costs of several hundred thousand euros can quickly arise, which is why we disagree with the applicant's assessment (pp. 99 to 101 And Table 28 on p. 115) and do not consider the labelling costs to be negligible.  Benefits to affected patients:  For controlled release formulations following benefits could be noted:  - constant level of drug concentration in the body, and prolongation of the therapeutic effect  - Reduced frequency of intake (e.g. once a day to once a month instead of 3 to 4 times per day)  - Minimisation of the drug-peak  - Reduction of side-effects of drugs  - Improvement of patients’ compliance  Benefits for immediate release formulations:  - Taste masking  - Distinctiveness  c. At the moment, there are no like-to-like alternatives for synthetic polymers.  Medicinal products may only be marketed after receipt of a marketing authorisation (MA). Such a MA will be issued by Health Authorities (e.g. European Medicines Agency (EMA) for EU or the National Competent Authorities (NCAs) for each country) after a thorough evaluation of the submitted documentation demonstrating the quality, safety and efficacy of the medicinal product concerned. Changes to the data provided in these documents are mostly only allowed after the approval of the responsible Health Authority. A lot of medicinal products are not only marketed in the EU but also outside. A reformulation would thus not only affect approved marketing authorisations in the EU but potentially worldwide. Such proposed changes are submitted as variations to Health Authorities for their evaluation and approval.  d. The use of polymers is essential in order to guarantee certain product properties.  Polymers used, for example, for film coating have continuously optimized oral solid dosage forms in recent decades, e.g. from a taste point of view, from a stability point of view or with regard to the discussed release modifications of active ingredients. In this context, the use of sugar icing or shellac, which have been frequently used, is no longer regarded as state of the art. Due to the GMP requirements, however, the pharmaceutical industry must constantly adapt to the state of the art in science and technology, why the use of sugar or shellac coatings cannot be regarded as an alternative.  At the moment all polymers (water-soluble as well as water-insoluble) are currently in scope of the proposed restriction, water-soluble polymers cannot be proposed as alternatives for water-insoluble polymers.  It would be interested whether there might be a potential for a derogation of water-soluble polymers from the restriction proposal, which would be in line with ECHA’s “Note on substance identification and the potential scope of a restriction on uses of “microplastics” (updated version 1.1, Oct 2018 )”.  At the moment, there are no like-to-like alternatives for synthetic polymers.  The formulation, i.e. the composition of a medicinal product, is decisive for its safety and efficacy. It can happen that development without polymers leads to a non-functional dosage form.  The search for alternatives and the reformulation can easily take several years. In addition, a variation (probably Type II) would have to be submitted in the course of the reformulation to the regulatory authorities.  The effects on the quality, safety, harmlessness and/or efficacy of the medicinal product concerned must be justified and demonstrated.  An assessment of the impact on the three main CTD modules  - Module 3 (e.g. related to the quality of the medicinal product including the manufacture of the product, test methods used, stability studies),  - Module 4 (related to the non-clinical - e.g. safety-relevant - data)  - Module 5 (related to clinical data)  would have to be done on a case-by-case basis for each formulation.  Furthermore, bioequivalence studies would have to be carried out to demonstrate the bioequivalence between current and new formulations.  Due to the many variables it is difficult to provide an accurate cost forecast. |  | a./b. The restriction proposal has an enormous impact on a large amount of substance-based medical devices using substances that contain solid synthetic polymers (e.g. modified cellulose, carbomer or sodium carbomer, acrylates crosscopolymer). One excipient in a medical device can have various functions (e.g. film forming, primary thickening, mechanical or rheological properties) depending on when and for what purpose used in the manufacturing process. Therefore, the concentration of synthetic polymers used is > 0,1% (w/w), in most cases < 1%. Such substances are essential in the formulation of the substance-based medical devices.  c. At the moment, we are not aware of any analytical methods for detection and quantification in the medical devices.  **Answer to specific info request 3:**  a./b. One excipient in a medicinal product can have various functions as for example as coating and/ or disintegrant depending on when and for what purpose it is used in the manufacturing process (whether polymer or not). Therefore, the concentration of synthetic polymers used cannot be provided generally. However, in most cases one can assume a concentration > 0,1% (w/w) and < 1%.  c. At the moment, we are not aware of any analytical methods for detection and quantification of polymers in medicinal products. Starting materials (e.g. polymers) have to comply with their individual specification before they could be used for the manufacture of medicinal products. Therefore, they are analysed in context with their individual specification. The particle size of polymer respectively a particle size range is often a parameter to which reference is made in the specification.  There are two test methods for analysing particle size distribution, which are described in the European Pharmacopoeia (EP):” Particle-size distribution estimation by analytical sieving” (2.9.38) or “laser light diffraction” (2.9.31). Unfortunately, the mentioned test methods cannot be used for the finished product respectively the enclosed polymer content.  d.In general, impurities consisting of polymers should not be found in any starting material. Under GMP regulations, it is not allowed to use polymer contaminated starting materials for the manufacture of medicinal products. Moreover, starting materials used for the manufacturing of medicinal products have to comply with their specifications. General requirements for the control of excipients can be found e.g., in the Guideline on “Excipients in the Dossier for Application for Marketing Authorisation of a Medicinal Product (EMEA/CHMP/QWP/396951/2006)”.  **Answer to specific info request 4:**  Our understanding is that filling materials of liquid chromatography columns also fall under this derogation. Liquid chromatography columns in small scale are e.g. used in laboratories for chromatographic analysis or are used in large scale for purification of reaction products in production plants. Used columns/filling materials are disposed as hazardous waste according to local waste treatment regulations as residues of chemicals or of the active pharmaceutical ingredient (API) might be contained in case of either application. Such materials should be e.g., incinerated, thus ensuring that no environmental exposure of microplastic will take place.  A confirmation that our understanding of this derogation is correct would be highly appreciated. |  |  |
| **7** | Band en Milieu (ELT management company NL) | **Content:**  Environmental emissions;  Baseline;  Information on alternatives;  Information on costs;  Information on benefits;  Other socio economic analysis (SEA) issues;  Request for exemption |  |  |  |  |
| **8** | BASF (industry) | BASF says the phase out of plastics and plastic additives is of “little concern”, noting that the problem is already being addressed by focusing on the need to accelerate waste management processes (Stringer, 2019).  BASF says its customers are voluntarily introducing plastics based on recycled materials: “We see an increasing interest in recycling, such as demands for recycled material or support by making certain products recyclable” (Stringer, 2019). |  |  |  |  |
| **9** | BE industry | Proclaim that they are fully behind a voluntary national agreement to phase out microplastics in all rinse-off cosmetic products and toothpastes by 31 December (CW, 2019l). |  |  |  |  |
| **10** | Belgium | **Comment:**  Impact on the Pharmaceutical Industry and EFPIA’s Position  ECHA’s proposed definition of microplastic comprises all solid polymers at ambient conditions with a particle size smaller than 5 mm in all dimensions. Not subject to the restriction are naturally occurring, not chemically modified polymers, and (bio)degradable polymers according to interim criteria set out in the Annex XV dossier.  Use of Synthetic Polymers in Medicinal Products  ECHA’s broad definition of microplastic puts a significant number of excipients used in the pharmaceutical industry and listed in the European Pharmacopeia into scope of the restriction (e.g. cellulose acetate, hydroxypropylcellulose, polyvinylpolypyrrolidone, hydroxypropylmethylcellulose phthalate, polymethacrylates, polyethylenglycol, and microcrystalline cellulose). Excipients are essential constituents in the formulation of medicinal products.  The European Pharmacopoeia includes an adopted list of excipients which are approved and safe for use in drug products; these are polymers in many cases. Excipients listed in pharmacopoeias show a good safety profile with regard to human or animal health and are comprehensively tested in accordance with the required safety studies for approval of drug products.  Furthermore, under European pharmaceutical law, the use of excipients depicted in monograph(s) of the pharmacopoeias, e.g., but not limited to, European Pharmacopoeia, is favoured. The requirements for the submission and approval of drug products are regulated in the EU guidelines and processes overseen by the European Medicines Agency (EMA) and health authorities of the EU member states which include proven safety and efficacy. Formulating a drug product is a complex task in which different factors like drug transport, drug release, uniformity (of content), hardness, and also shelf life have to be considered, as well as patient (human and animal) acceptance. The formulations of certain active pharmaceutical ingredients (API) need compensation of undesired physicochemical properties in order to improve the producibility, pharmacokinetic profile and the therapeutic effect. The use of polymers is very broad in medicinal products as they provide solutions to various API specific properties. There are limited alternatives to exchange polymers as excipients in medicinal products and the alternatives are not suitable for most formulations due to their inability to mimic the specific and necessary properties as outlined above. Therefore, the vast majority of solid oral dosage forms contain polymers.  Thus, changing the composition and formulation of a drug product once approved is an extremely involved process, in many cases substituting may not be possible due to changes in bioavailability &/or efficacy of the API. However, where changes may be viable extensive studies would be necessary which would take years to perform and may include bioequivalence studies, in addition to approval of variation of one or multiple authorities responsible for drug product approval in respective markets.  To conclude, EFPIA would like to stress that a restriction on the use of polymers will affect most solid form drug products for human or veterinary use, and potentially other dosage forms. This would have severe impact on the availability of drugs in Europe, and patients’ safety. As such, EFPIA welcomes the derogation of medicinal products for human and veterinary use from the restriction of intentionally added microplastics.  Reporting  ECHA´s draft restriction proposal requests reporting of the used excipients, which are considered as microplastics under the ECHA definition. Such reporting is required to be performed on an annual basis and includes: the identity, the use and quantity of the polymer, and the estimated quantity released to the environment. In EFPIA’s point of view, this raises a high bureaucratic burden for the pharmaceutical sector without any benefit to the patient or environment. Volumes of polymeric excipients are expected to remain the same over time due to their importance for e.g. oral dosage forms. Polymers have been essential to the formulation technology of medicinal products for decades. New excipients on the market for tablets are limited and any additions will most likely only have minor structural edits by cross linking of existing polymers or differently modified cellulose backbones but all fall under the broad definition used for microplastics.  Labelling  According to the EU pharmaceutical law, the package leaflet contains a note on disposal of unused medicinal product. This information covers the labelling needs described in ECHA’s restriction proposal.  EFPIA therefore concludes that no revised package leaflet or any other additional labelling of medicinal products is needed.  Conclusion  The Pharmaceutical sector uses a variety of synthetic polymers e.g. derivatized celluloses, which would fall under the current very broad definition of microplastics by ECHA. These excipients, which are proven safe and would be difficult to replace, are critical to ensuring the uninterrupted supply of high quality and efficacious medicines of importance to patients.  We consider it inaccurate to define every polymer with a size < 5 mm as a microplastic. This definition would even include oligonucleotides, polysaccharides and peptides, when being chemically treated during manufacture. Even microcrystalline cellulose, as being chemically treated during the manufacturing process and commonly used in galenics, would fall under the definition of microplastic, while chemically untreated cellulose, also used in galenics, would not fall under this definition.  The key points and requests EFPIA would like to make to ECHA are:  - We strongly support the derogation of medicinal products from the REACH restriction.  - We believe the definition of microplastics, whereby it includes common polymeric excipients, would benefit from a refinement of the scope/definition.  - The requirements for reporting and labelling of medicinal products will entail a high bureaucratic burden for companies without a beneficial impact on society or the environment. To this point we ask that you support our proposal whereby current medicinal product labelling regulations are adequate to meet the requirements of ECHA’s restriction proposal and the medicinal product derogation should extend to reporting requirements. |  | **Answer to specific info request 3:**  In most cases synthetic polymers are intentionally used in solid drug formulations with a concentration >1% (w/w). In some formulations synthetic polymers might also be used in the concentration range 0.1-1% (w/w).  Currently there is no analytical method available to detect the particle size and particle number of polymers in finished medicinal products for human and veterinary use. The polymer raw material prior to formulation is analysed for compliance with specification. The specification of solid polymers raw materials includes in most cases a particle size range. Here the particle size is analysed by e.g. laser diffraction or sieving. These techniques cannot be used to analyse the particulate size of the polymer contained in e.g. the tablet after formulation/ compression.  **Answer to specific info request 4:**  Our understanding is that filling materials of liquid chromatography columns also fall under this derogation, therefore we cite one example. Liquid chromatography columns in small scale are e.g. used in laboratories for chromatographic analysis or are used in large scale for purification of reaction products in production plants. Either way, used columns/filling materials are disposed as hazardous waste according to local waste treatment regulations as it may contain residues of chemicals or e.g. active pharmaceutical ingredient (API). The material is e.g. incinerated and no environmental exposure of microplastic takes place. |  |  |
| **11** | BIR Tyre & Rubber Committee | See enclosed document named "BIR document microplastic restriction 20190517 Final version" |  |  |  |  |
| **12** | British Coatings Federation |  |  | Microplastic content 7-10% in water-borne paints, coatings and printing inks using the proposed definition |  |  |
| **13** | Bundesverband Korrosionsschutz e.V. | **Comment:**  The proposed reporting requirements for manufacturers of paints, coatings and printing inks and also for industrial users of these products involve a large amount of bureaucracy – while they come nowhere near the given objective of tracking uses and potential releases to the environment. The obligation of industrial users to estimate the release of microplastics to the environment overlooks that comprehensive water protection rules are already in place for industrial sites. These rules regulate how to treat waste waters prior to their direct release into waters or their indirect release. Overall, the reporting requirements constitute one-sided and unjustified burdens on industrial users. We doubt that ECHA is aware of the manifold uses of paints and coatings in the industrial corrosion protection sector. |  | **Answer to specific info request 5:**  The users of paints and coatings for corrosion protection are dependent on the supply of the manufacturers of these coatings. As many of the coating materials are also restricted by German guidelines, alternatives (if there are any) cannot be chosen by the user. The formulation of paints and varnishes for corrosion protection is task of the paint industry. The companies working in the field of corrosion protection are predominantly small and medium-sized enterprises (SMEs). These companies don´t have the personnel to prepare the proposed annual reports – which are to be sent electronically to ECHA – include the following points:  a. the identity of the polymer(s) used in the previous year;  b. a description of the use of the microplastic,  c. the quantity of microplastics used in the previous year, and  d. the quantity of microplastics released to the environment, either estimated or measured in the previous year.  **Comment:**  For these reasons, we think that flock fibres up to 15 mm long, should not be declared as microplastic in relationto your study and recommendations. |  |  |
| **14** | Bundesverband der Pharmazeutischen Industrie | see attachment |  |  |  |  |
| **15** | CEFIC | Criticizes the microplastics definition by ECHA for being “too broad” leaving “room for interpretation” making implementation and enforcement of restrictions “challenging” (Tani, 2019).  Two of seven Long-range Research Initiatives by CEFIC deal with microplastics. A total of €600,000 (CW, 2018f).  Speaking for the chemical industry trade association CEFIC, Blanca Serrano Ramón also expressed reservations about ECHA’s proposal, arguing that the agency’s definition of microplastics as a ‘polymer containing particle’ was too broad.  Cefic believes that REACH is an appropriate regulatory tool to address chemical risk EU-wide.  However, as currently proposed, the Annex XV proposal for a restriction on intentionally added microplastics does not follow the requirements of the REACH regulation, does not achieve the intended objective of protecting human health and the environment, and therefore cannot be supported by industry  • The assessment of a group of substances in a generic manner i.e. polymers and polymer-containing particles with very different properties and behaviours is not a suitable basis for the assessment of environmental effects, in particular hazard and risk according to the REACH provisions  • The broad and generic definition makes the restriction extremely difficult to understand, interpret, and ultimately, enforce;  • The restriction lacks the first defining element of risk – an identified hazard – and hence bases the proposed regulatory measure on risk posed by the “extreme persistency of polymers”  • Since a hazard or a risk posed by microplastics has not been identified in accordance with the rules of the REACH Regulation, the proposed measure does not follow the principles laid down in the REACH Regulation; | The principles that drive the use of the precautionary principle state that the scientific risk assessment should be based on the best scientific data available. The scientific evidence alleged does not meet this standard of evidence required;  • The restriction proposal introduces an extensive set of reporting requirements to a very large number of derogated uses, creating significant additional administrative burden without significant added value.  Many of these concerns arise from the fact that the proposed restriction dossier is based on a generic and extensive definition of microplastics that covers nearly all solid polymers and polymer containing particles, in spite of their very different chemical and physical properties. It is conceivable that many of the concerns above could be addressed by devising a restriction with a narrower, more targeted scope. Specifically, this would mean to assess materials with more comparable physchem properties in their specific applications and derive measures for uses identified as high risk. The scope could be adjusted as science evolves and adequate alternatives are developed. In our view, the risk assessment carried out by ECHA concluding that such an approach would be equal in terms of effectiveness, practicality and monitorability is not adequate. A revised assessment of a more targeted approach would be very much welcome. | “*A proposal by Echa to restrict microplastics cannot be seen as appropriately meeting a legitimate objective under the REACH Regulation, according to analysis carried out by a leading international law firm*” (Buxton, 2019). The analysis was commissioned by CEFIC. |  |  |
| **16** | CEPE | **Comment:**  - Scope is too broad and unworkable  - Use inside an industrial installation should be derogated and not be subject to labeling and reporting  - Reporting makes no sense and has no benefits and should be omitted from the restriction  See under section IV the uploaded CEPE comments. |  | **Answer to specific info request 3:**  3 a) For Waterborne paints a minimum of 5% of solid polymer dispersion is needed to achieve properties of adhesion etc.  3 b)  1%; 100% of Waterborne paints.   1%: 10-20% of Solventborne paints  3 c)To our expert knowledge, no analytical methods exist that would detect and quantify microplastics in the presence of all the other components in a complex paint mixture (which will include inorganic particulates, non-particulate polymers, organic compounds, etc., as well as any microplastics meeting the regulatory definition).  3 d) No  **Answer to specific info request 5:**  5 a) see under Section IV the CEPE comments  5 b) The waterborne dispersions of solid polymer particles acting as ‘film-formers’ have enabled the paint and printing industry to comply with new societal demands on human health aspects and protecting the environment. The use of organic solvents (VOC’s) has been greatly reduced in the sector. Today the majority of paints for buildings are water borne (over 80 %).  5 c) When losing the film-forming capability of the polymer dispersion the paint would no longer protect the substrate in a reliable fashion. A negative influence on the overall sustainability of the paint and the underlying substrate will be the result.  Concerning alternatives. The only technical alternatives with equal properties would be paints based on polymers in solutions of VOCs. But a return to the massive use of such paints would cause other problems than the residual release of microplastics from the waterborne paints of today.  Using bio-degradable polymers in waterborne dispersion would significantly reduce the functionality of the paint and the longevity of the surface protection. It would increase the release of particles to the environment from the partially degraded film and underlying substrate.  5 d) not applicable |  |  |
| **17** | Chemical Industries Association | **Comment:**  The UK Chemical Industries Association (CIA) welcomes the opportunity to provide comment on the proposed restriction on intentionally added microplastics. Whilst we support the REACH Regulation as the legislative vehicle for addressing the management of chemicals and associated risks where applicable, we are concerned about the way in which the regulation is being proposed to be used for restricting the use of intentionally added microplastics to products. CIA thereby offer the following points for consideration and highlight that we support the input provided by the European Chemistry Council, Cefic.  CIA is the organisation that represents chemical and pharmaceutical companies located throughout the UK. The UK chemical and pharmaceutical industries have a strong record as manufacturing’s number one export earner and a provider of essential inputs to UK value chains. This includes products and technologies which are key enablers of sustainable solutions including for climate change, food production and healthcare.  CIA supports regulatory action where this is justified but, in this case, we do not believe this to be so and consider the proposal to be a misuse of the REACH framework and the precautionary principle. Our primary concern is that the required scientific evidence to justify this proposal from ECHA has not been met. It also differs to previous REACH restrictions by focussing on a group of substances (i.e. polymers, when in fact these are a class of substances) rather than individual substances. In our view, restrictions put forward for consideration should fully meet the REACH legislative criteria and not be of a generic approach. Recognition also does not appear to be given to the fact that polymers can have very different properties and behaviours (i.e. low or high density, nanosize, low molecular weight or oligomers, cationic, anionic, amphoteric, non-ionic, hydrophobicity, those containing reactive functional groups) that would impact their water dispersability, bioaccumulation and biodegradability.  In terms of the proposed definitions, we believe these need more consideration. In the evidence we submitted in 2018 we stressed the point that the term “plastic” is often misused as this is often applied generally to refer to all polymers; this is scientifically incorrect. It may therefore be appropriate to include a definition of “plastic” in the proposal rather than “polymer” to ensure clarity. As presently defined in the proposal, a broad range of polymers would be covered by the definition; in our view, a narrower scope that focussed on uses that are identified to be of high risk would be more appropriate. This could then be periodically reviewed as scientific evidence becomes available.  Looking at reporting requirements we question whether these need to be so extensive since as currently proposed we expect there to be increased administrative burdens on companies with associated costs since these would apply to a large number of derogated uses. We also ask for clarification on the legal basis of these reporting obligations, especially considering the complex supply chains within the chemical sector. |  |  |  |  |
| **18** | CIRFS Europian Man-made Fibres Association | CIRFS is the association for Europe’s € 10 billion man-made fibres industry, representing the industry to the European authorities and providing the industry with a wide range of services. Its members cover more than three-quarters of European man-made fibres output.  The European man-made fibres industry, with a total production in 2018 of ca. 4.6 million tonnes, is the world’s second largest in terms of output and one of the global leaders in terms of innovation and quality. Man-made fibres are essential and critical in many different applications.  Under the REACH Regulation, man-made fibres have been defined as ‘articles’, made from polymers and are neither ‘substances’ nor ‘mixtures’. As a matter of principle, articles cannot be subject to a restriction process under REACH.  CIRFS therefore considers that man-made fibres are out of the scope of the current proposed restriction. |  |  |  |  |
| **19** | CMSSA – Czech Seed Trade Association | Detailed input will be provided through the document that is submitted through Section IV. |  |  |  |  |
| **20** | Confederation of European Paper Industries | The Confederation of European Paper Industries (CEPI), representing the manufacturing sector of pulp and paper, is concerned about the way the ECHA  restriction proposal* has been developed.  Although we agree on the urgent need to restrict substances or mixtures containing microplastics that will persist in nature and cause environmental harm, we believe the scope definition requires clarification to avoid unintended impacts on substances, mixtures and articles made from natural and modified polymers of lignin and cellulose pulp. These should be excluded from the scope of the Restriction.  The justification for the proposed amendment can be found in the annexes on kraft lignin and sulfonated lignin.  *Annex XV restriction report proposal for a restriction of intentionally added microplastics (Version number 1.1 of 20 March 2019) |  |  |  |  |
| **21** | Cosmetics Europe | Cosmetics Europe hereby submits three addition documents, following its submission of 15 documents on 20^th^ May. The documents address the specific features of Leave-on cosmetic products (information provided elsewhere in our previous submission, but gathered into a single document); Solubility; and Biodegradability. |  |  |  |  |
| **22** | Cosmetics New Zealand | **Content:**  Hazard or exposure;  Information on costs |  |  |  |  |
| **23** | CTPA | The Cosmetic, Toiletry and Perfumery Association (CTPA) has said it could take longer to reformulate products, if the proposed UK ban on microbeads was expanded to include ‘leave-on’ products. (Lovell, 2017).  A UK cosmetics trade body has said personal care products should not be the main target of government policies, to prevent microplastics polluting the ocean. (Lovell, 2017).  Cosmetics, Toiletry and Perfumery Association (CTPA) director-general Chris Flower said: “If we are to stem the tide of microplastic marine litter, we must tackle the major sources and these are not cosmetic products.” (Lovell, 2017). |  |  | CTPA science director Emma Meredith told Chemical Watch that product re-development could take up to four years if raw materials became unavailable. “*We are unaware of any evidence to show that ingredients from leave-on cosmetic and personal care products have been found to pose a risk to the marine environment. Only rinse-off cleansing and exfoliating products have been associated with marine litter.*” (Lovell, 2017). |  |
| **24** | Danish Coatings and Adhesives Association |  | Description of analytical methods;  Information on benefits;  Request for exemption |  |  |  |
| **25** | Dansk Industri et al | Please find herewith including in the attachment below, the Joint Nordic Industry – and Chemicals Federations response to ECHAs public consultation on the proposed restriction on intentionally added plastics, as submitted jointly by IKEM (Innovation and Chemical Industries in Sweden), Kemian Teollisuus (The Chemical Industry Federation of Finland), Norsk Industry (Federation of Norwegian Chemical Industries) and DI (Confederation of Danish Industry).  We the Nordic Industry – and Chemicals Federations share the Commission and ECHAs view that plastic and microplastic releases to the environment need to be addressed and significantly reduced at EU and Global level.  We acknowledge that REACH is an appropriate regulatory tool to mitigate risk EU-wide. However, as currently proposed, the Annex XV proposal for a restriction on intentionally added microplastics is based on a generic and extensive definition of microplastics that covers nearly all polymers and polymer containing particles, in spite of their very different chemical and physical properties. The EU wide unacceptable risk to health or environment is not validated in the same accuracy than is done for substances.  We propose devising a restriction with a narrower, more targeted scope. Specifically, this would mean to assess materials with more comparable properties in their specific applications and derive measures for uses identified as high risk. The scope could be adjusted as science evolves and adequate alternatives are developed. In our view, the risk assessment carried out by ECHA concluding that such an approach would be equal in terms of effectiveness, practicality and monitorability is not adequate. A revised assessment of a more targeted approach would be very much welcome.  We, as members of Cefic, refer to the Cefic’s general comments and specific messages submitted in response to the public consultation.  Representing a large number of down stream users, we would like to offer some additional comments/ examples relation to the definition and the proposed labelling and reporting requirements in the attachment below. | It is also different to other REACH restrictions in that the reasoning is mainly based on precautionary principle. |  |  |  |
| **26** | Deutsche Bauchemie e.V. | **Comment:**  Position of Deutsche Bauchemie (German association for construction chemical products) in the public consultation on the ECHA proposal for a restriction of intentionally added microplastics  The industry association Deutsche Bauchemie expressly welcomes ECHA’s initiative to reduce the release of microplastics to the environment.  Deutsche Bauchemie shares the position of the German chemical industry association Verband der Chemischen Industrie (VCI) that the restriction proposal contravenes the provisions of the REACH Regulation. Therefore, Deutsche Bauchemie endorses the views brought forward by the VCI. In particular, this holds true for the following points of criticism:  • Insufficient description of substance identity  • Lack of identification of hazard and risk  • Lack of detail in the risk assessment  • Lack of efficacy, effectiveness and proportionality  • Lack of legal basis for extensive product labelling and for the proposed disproportionate annual reporting requirement  Additionally, Deutsche Bauchemie would highlight the following points:  1. Disproportionate cost and effort for the annual reporting requirement (8)  For mixtures that fall under the exemptions of 5b and 5c, the party placing them on the market needs to annually report to ECHA several items of information (8a to 8d). Given the lack of identification of hazards and risks and the inadequate risk assessment (see VCI position), the cost and effort of this annual reporting requirement are disproportionately high and – as there is no need for action demonstrated – this is inappropriate. It would cause disproportionate cost and effort especially for many small and medium-sized enterprises (SMEs) in the construction chemical sector.  • Moreover, many of these companies are not familiar with the “REACH Reporting Tools” such as e.g. IUCLID. This means an additional strain in implementation.  • However, it is worth noting that also many SMEs in the construction chemical sector manufacture and distribute a wide range of different products. That would bring a comprehensive and complex “reporting matrix”, because the details under 8a to 8d would need to be determined and reported individually and separately for a multitude of different polymers and microplastic materials.  2. Data to be reported (8(a) to 8(d))  According to 8), the manufacturers of impacted products would have to report annually to ECHA the data concretised under 8(a) to 8(d).  • Usually, manufacturers of construction chemical products do not have sufficient information on the identity of the polymer to report the data under 8(a). This applies especially for polymers that are not classified as hazardous. In these cases, the information available to manufacturers does not enable them to comply with the reporting requirement stipulated in 8(a).  • According to the restriction proposal, frequently several subsequent actors in the supply chain would need to report to ECHA. It must be expected that the volumes of microplastics would be grossly overestimated due to multiple reporting along the supply chain.  • Under the restriction proposal, many materials which mainly consist of inorganic, non-polymeric shares and only to a very low degree of polymers would be defined as “microplastics”. According to 8(a), the identity of the polymer(s) and, according to 8(c) and 8(d), the quantities of microplastics used and possibly released microplastics have to be reported. Consequently, it must be feared that a wrong picture will form of the quantities of polymers that are used and possibly released.  3. Transitional periods for reporting to ECHA  12 months after entry into force of the restriction, impacted manufacturers of products that fall under the exemptions of 5(b) or 5(c) are to report data to ECHA for the previous calendar year.  • Comprehensive adaptations of company-internal IT systems are necessary to gather and evaluate the required data and to submit them to ECHA.  • Moreover, the data collection period for the data to be reported practically starts, without transitional period, directly with the entry into force of the restriction – as reporting is to cover the period of the previous year. This means that the adaptation of IT systems would need to be completed already at the time of entry into force.  • In view of both of the above points, a one-year transitional period for reporting to ECHA is much too short and should be extended considerably.  4. Water-soluble polymers should not fall under the microplastic definition  Water-soluble polymers are not present as particles in the environment and should therefore be excluded from the Microplastics definition and the scope of the restriction. In this regard, Deutsche Bauchemie also joins the positions of VCI and CEFIC. | Disregard of the principles for the application of the precautionary principle | Impact on the construction chemical industry  Having intensively examined the ECHA restriction proposal, the possible impact on manufacturers of construction chemical products is appraised as follows:  • Construction chemical products contain various constituents which meet the proposed definition of microplastics. However, in the intended use, these constituents are not released to the environment. Consequently, a ban of the placing on the market (1) of construction chemical products is not expected.  • Constituents (ingredients of formulations) of construction chemical products (mixtures) fall under exemptions, because in their use they are  o either permanently modified in a “non-microplastic form” (5(b))   Example: polymer dispersions as binders which irreversibly convert into a polymer film during use.  o or permanently incorporated into a solid matrix (5(c)).   Example: use of polymer fibres or polymer surface-coated fillers and pigments in cement-bonded products. In their use, the polymer fibres or the polymer surface-coated fillers and pigments are permanently incorporated in the cement matrix.  Thus, under the exemption rules 5(b) and 5(c), these products can continue to be placed on the market; however, they are subject to labelling, communication and annual reporting requirements.  Position on the restriction proposal |  |  |
| **27** | DIGITALEUROPE | DIGITALEUROPE believes strongly that ECHA’s definition of plastics should be revised to take into account the specificities of plastics and their solid forms, along with their final application. Plastics used as part of a production process which are due to lose their microplastic properties should not be part of the scope of the restriction report, as their final form will not be microplastic. As microplastics are fused in the printing process, printing inks and ink toners should not be considered in the scope of this proposal. |  | Before printing, all toner is microplastics by the definition provided in the Annex XV report. The particle size is in the microparticles area for every cartridge in the market (1 nm to 5 mm). All particle above 5 mm cannot be used in the toner. If a liquid printing ink contains microplastics, the concentration must usually be above 1% to have effect.  We are not aware of microplastics corresponding to the definition proposed in the restriction being present in a substance or a mixture as an impurity. |  |  |
| **28** | EDANA | **Comment:**  please see EDANA statements in attachment   |  |  |  |  |
| **29** | EFfCI - The European Federation for Cosmetic Ingredients | see attached doc |  |  |  |  |
| **30** | ETRMA – European Tyre & Rubber Manufacturers Association | ETRMA provides general and specific comments on the document attached. |  |  |  |  |
| **31** | EurEau | Klara Ramm, chair of the [EurEau](http://www.eureau.org/) Committee on Economics and Legal Affairs: “*One way of solving the underinvestment issues would be for the EU to fully implement the Polluter Pays Principle. This becomes even more vital as the technologies to remove emerging pollutants such as pharmaceuticals, microplastics or pesticides require substantial investments from the water sector, which is not the originator of the pollution*” (Ramm, 2018) |  |  |  |  |
| **32** | European Crop Protection Association (ECPA) | **Comment:**  Transitional period  • The crop protection industry recognises the importance of the microplastic issue. ECPA member companies are committed to minimising the use of substances clearly categorizable as microplastics and developing safe and effective alternatives.  • Until suitable alternatives become available we will continue to support technologies involving polymers where these enable compliance with requirements in EU legislation on safety, health, efficacy and environment. These benefits should not be lost without an appropriate transition period allowing companies that supply such products for crop protection to search for and develop and register alternative technologies. Once alternatives are available, the crop protection sector will additionally need time to register new formulations and seek approval from national and EU regulators.  • For such a broad impact, an overall transition period of 15 years is estimated as being the minimum period required to develop suitable replacement technologies, and implement these changes into new registered formulations. The proposed 5 years is only sufficient to meet the stringent registration requirements for a single plant protection product in isolation, but leaves no time for the preceding development of new replacement technologies, reformulation activities (due to capacity these cannot all be done in parallel), and further downstream user’s own replacement efforts. A review of replacement technology progress at 10 years is recommended.  Information on benefits  • The beneficial uses of polymers in plant protection products include minimizing the amount of active substance used, reducing potential operator exposure to pesticides, help prevent undesirable environmental impact, and permit the products to meet the required technical specifications. These benefits risk being lost before suitable replacements are available.  Scope or restriction option analysis  • The current microplastic definition is extremely broad and not defined in a way that would allow industry to comply or regulators to enforce. There are no reliable and commonly available testing methods that would allow this definition to be applied in practice. The would result in significant uncertainty and unpredictable impact, while losing the original focus to address the problem of clear particulate plastics, for example potentially bringing into scope colloids and macromolecules that are not commonly understood to be microplastics.  **Comment:**  In essence, ECOS welcomes ECHA`s proposal to restrict intentionally added microplastics in products. We are glad to see a general acknowledgement of the adverse effects of microplastics and a commitment to prevent their use in various sectors. At the same time, however, we believe the proposal can be strengthened in several aspects, most importantly the concentration limit of 0.01% w/w. This would still allow a high amount of microplastics in products, representing millions of particles with the potential to end up in the environment. To really end microplastics pollution and give a clear signal to the market, we propose a complete ban on microplastics being intentionally added to any type of product (with an exception for medical and research purposes under strict conditions). This would push industries to search for natural solutions, that have actually already existed before the introduction of microplastics.  In our position paper (see Annex) we provide more elaborate feedback on the following aspects:  1. Definition  2. Biodegradability  3. Hazards by and exposure to microplastics  4. Transitional period  5. Exemptions |  | In our view the lower size limit should be 1µm. Polymers <1µm are macromolecules best regulated as substances following a risk based approach. With the European Commission now signalling a REACH polymer registration concept on a similar timeline to the proposed microplastic restriction, this offers an established regulatory mechanism to proportionately control risks.  • Repurposing the CLP definition of a solid to aid in the microplastic definition makes some regulatory sense. However, a melting point is a bulk property, which is contradictory when it should define the state of individual particles, which may be macromolecules. Furthermore, particles which are heterogeneous (e.g. composite or structured materials) may contain polymeric and non-polymeric components with multiple differing melting points. The CLP definition can only be unambiguously applied if the lower size limit is >1µm, and the melting point references the polymeric components.  • 1nm size scale cannot easily be measured, especially in complex and concentrated mixtures. The size measured can depend on the environment / matrix in which it is measured e.g. pH, temperature, ionic strength, etc. This would make compliance and any enforcement nearly impossible, highly variable (different results for the same polymer in different products) and highly contestable. The vast majority of companies, especially SMEs, do not have the required equipment to measure according to the proposed definition, and it is not clear that enforcement authorities and official control laboratories also have access to the required equipment. If the size limit cannot be easily, accurately, and reproducibly measured, it cannot be enforced. As a result, the lower size should be at least 1µm, where reliable particle sizing methods can be employed.  • The "polymer containing particle" definition applies to particles with polymer content as low as 1%, as well as coated particles of up to 5mm. Because the reporting provisions are for "microplastic" quantity rather than the polymer quantity used / released to the environment. This will result in massive exaggeration of the real emissions for treated seeds (it will include the mass of the seed), microencapsulated formulations (it will include the mass of the active substance and solvent), and low polymer content particles (it will include all the non-polymer mass). Only the polymer quantity should be reported.  Information on costs  • The impacts on the crop protection and downstream industries have been severely underestimated. The uncertainty caused by the proposed definition, and in particular the change to a 1nm lower size limit, means that a potentially large number of macromolecular or colloidal co-formulants are possibly in scope and have not been accounted for in the ECHA impact assessment.  • The reformulation cost for a plant protection product has been severely underestimated in the ECHA impact assessment.  **Answer to specific info request 1:**  In higher tier tests used to assess degradation in soil and aquatic environments, such as OCED 307 and OECD 308, the degradation rate (DT50) and subsequent persistence criteria are usually based on the disappearance of the applied test substance over time and therefore only consider primary/initial degradation steps rather than complete mineralisation. Suitable extraction and analytical methods would need to be developed to enable direct measurement of the applied test substance in test systems over time. This will be technically challenging for insoluble polymeric microplastics since analytical methods generally require compounds to be present in solution to be measurable. If direct analysis of the test substance is not feasible, the use of radiolabelled material may be helpful in assessing the ultimate degradation (mineralisation) of polymers in soil and aquatic laboratory studies by following the amount of 14CO2 generated over time. However, the extent of mineralisation observed using 14C-material will vary depending on the nature of the chemical structure and the position of radiolabelling. OECD 307 and 308 test guidelines specify that the radiolabel should be positioned in the most stable part(s) of the molecule in order to assess the route of degradation, which may or may not be mineralised during the course of metabolism. Note that radiolabelling of chemically modified natural polymers may not be possible. A compound and/or its metabolites may also become assimilated into the test system biomass, which is generally reflected in an increase in unextracted residues in addition to CO2 formation in such studies, and therefore complete mineralisation may not be observed. Hence, the extent of mineralisation may not reflect the extent of primary degradation and any persistence criteria set on the basis of mineralisation alone in these test systems should be used with caution.  **Answer to specific info request 3:**  A) The minimum concentration of microplastics intentionally added in end products, and required to fulfil their intended technical function, depends strongly on the intended function and chemistry of the polymer/microplastic involved. Representative data is not available at this time.  B) Representative data are not available at this time.  C) Analytical methods  Table 4.11 in the JRC report “Requirements on measurements for the implementation of the European Commission definition of the term 'nanomaterial'” (available here: http://publications.jrc.ec.europa.eu/repository/bitstream/JRC73260/irmm_nanomaterials%20%28online%29.pdf) provides a convenient overview of analytical methods available for the measurement of particles at the nanoscale.  Standard light scattering equipment is widely available in most companies, which measures adequately the micrometer range, but does not extend to the nanometer scale. Dynamic light scattering (DLS) has more limited availability, but does extend into the nanometer range, and is often proffered as a readily implementable method. The following comments relate to DLS, in particular in the nanometer range, where it has a theoretical advantage over other light scattering methods.  • DLS is based on interference patterns due to motion of small particles undergoing Brownian motion in dilute solution in a low-viscosity Newtonian medium. The scattering interference patterns are fitted to models which yield particle size distribution curves. Interference patterns can arise from molecules, droplets, particles or any ‘entity’ which is mobile in the medium and scatters light. Therefore, the presence of an interference pattern and an ‘apparent’ particle size distribution does not demonstrate a particulate dispersion. Considerable knowledge of the system and expert analysis is required to yield scientifically valid interpretations of the results. Plant protection products are usually mixtures of between 5-10 co-formulants, any of which in turn may be in itself a mixture. Determining whether the scattering from such a system results from the presence of microplastics would be extremely difficult, particularly in the submicron range.  • DLS has to be performed at very low concentrations in order that ‘true’ Brownian motion takes place. The viscosity of the medium must be low. Plant protection products as placed on the market are usually concentrated as possible to reduce transport costs. In addition, products with organic solvents often contain thickeners to remove aspiration hazards. Dilution of such a sample obviously changes the concentration, and thus the potential conformation of large polymers, and hence their size – mostly of relevance in the submicron range. The results of any determination are thus measurement dependent, and do not necessarily reflect the polymers in the product as they are placed on the market.  • Considerable sample purification (e.g. filtration, centrifugation) is required to remove impurities, especially from commercial products. This may dramatically limit the ability to accurately characterise the sample (e.g. polymer concentration) and interpret the results.  • Both active substances, and non-polymeric particles (e.g. silica, kaolin etc) may be present in a formulation and scatter, masking the presence of potential microplastics.  • Although resolution for DLS down to 1nm is claimed, this is for well characterised ‘ideal’ systems (e.g. highly pure, low polydispersity, high dilution, know scattering profile). A more realistic lower limit for commercial samples would probably be around 20nm. If there is more than one source of scattering the interpretation becomes much more complex and resolution / accuracy will be lost.  • The apparent particle size distributions can depend on temperature as well as concentration, viscosity, anisotropy of the ‘scattering entity’ and assumptions in the interpretative algorithm.  • Based on the above considerations, the application of DLS to characterise aqueous solutions/dispersions of commercial plant protection products, or polymer co-formulants as raw material, is likely to be a complex task which is unlikely to yield reliable information suitable for either quality control or enforcement purposes. The presence or absence of an apparent particle size curve would not be conclusive and would not give information about the nature of the ‘scattering’ entity (molecule, ‘liquid’ droplet, ‘solid’ particle etc.).  • The key conclusions from the JRC nanomaterial measurement report relevant to nanoscale particle size measurements are summarised as:  o Different size measurement methods may provide significantly different size values.  o No method can distinguish if a large particle is an aggregate/single particle and large numbers of individual particles at the same time.  o No single method alone can cover in a single measurement the complete size range from 1nm to above 100nm.  o None of the currently available methods can determine for all kinds of nanomaterial whether they fulfil the definition or not… there are significant difficulties with the nanomaterial definition for polydisperse materials.  D) We are not aware of microplastics present in substances or mixtures as an impurity, but this is clearly a theoretical possibility. |  |  |
| **33** | European offshore oil and gas industry | Nik Robinson, secretary of the European Oilfield Speciality Chemicals Association (Eosca), told Chemical Watch that the EU study had relied on “poor and unreferenced reports” (Oziel, 2018). |  |  |  |  |
| **34** | European Oilfield Speciality Chemicals Association | Expressing [concern](https://chemicalwatch-com.proxy.findit.dtu.dk/65720/) about Echa’s digression from the Commission’s original definition of a microplastic to a “broader ‘catch-all’” one (Oziel, 2018).  The definition of microplastics and their “fate and partitioning” is crucial to the discussion about potential discharges and remedies (Oziel, 2018).  The European Oilfield Speciality Chemicals Association has warned its members, as well as non-members, that the consequences could be “heavy controls or even bans being placed on the marketing and use of products containing microplastics”. (Buxton, 2018)  EOSCA have submitted full general comments in the attached non-confidential attachment. This includes comments on the following points:  * EOSCA's understanding of the clarification of the definition;  * EOSCA's support for the derogation for industrial sites due to the reduced risk from these non-wide and dispersive uses;  * EOSCA's comments and proposals for labelling and relevant instructions communication;  * EOSCA's comments and proposals for reporting; and  * EOSCA's clarification on ECHAs comments about substitution availability within the offshore oilfield industry.  In EOSCA's response to the previous consultation on behalf of the oil and gas industry details of reported microplastic concentrations were given.  Rather than repeat these, the reviewer is referred to this submission. However, EOSCA has proposed and encouraged all members to respond individually to this consultation, and specifically this point due to formulation and concentration data being Confidential Business Information. It is hoped that these data will give a better understanding in light of the new proposed definition for microplastics. |  |  |  |  |
| **35** | European Polymer Dispersion and Latex Association (EPDLA) | **Comment:**  Baseline  The EPDLA supports CEFIC’s position regarding the applicability of REACH on this topic due to the scope of the restriction proposal being too broad and not sufficiently defined, causing tremendous efforts which are not proportional towards the targeted effect. By assessing a group of substances identified generically, as opposed to a group of individually identified substances without having identified hazard, it is also doubtful that the planned restriction is covered by the REACH regulation as is. Even administrative bodies are questioning the use of REACH for this microplastics restriction. While the EPDLA supports efforts to reduce (micro)plastic pollution into the environment, the precautionary principle seems to be abused in this instance.  Dispersions have been successfully and safely used for decades e.g. in coatings and adhesives and have helped to significantly reduce VOC emissions and improve workplace hygiene due to the reduction of solvent usage in such applications.  The EPDLA agrees that industrial sites should be derogated from this restriction, but further suggests that professional sites with equivalent risk management measures (RMM) for workers should receive the same derogation. In professional settings, well trained, well instructed and well-equipped workers are implementing RMMs very effectively, and we consider that the use of microplastics in such professional settings should also be considered safe and therefore derogated from this restriction.  Additionally, the EPDLA believes it is important to understand how ECHA will interpret collected data, knowing that there is a time lag between producing and selling a polymer dispersion to a paint producer, and then until they produce the paint? Another delay occurs when the paint is sold to distributors, paint shops, outlets, which are exempt from reporting. Here another delay occurs, until the sale to the end consumer, who may cause another lag, not using the paint immediately after purchase. The analysis methods to be used on the collected data has not made clear and nor have the benefits in collecting the data in the first place, particularly for polymer dispersions, for which the microplastic “property” disappears anyway through film forming. We believe such reporting does not provide any benefit and we thus ask to remove the reporting requirements for industrial uses, or at least for any products which will film-form and/ or end up in a solid matrix.  Workability - labelling  Within industry (B2B), communication on health and safety and on how to handle products properly is well established in using safety data sheets (SDS). The member companies of EPDLA as a rule supply to industrial customers who convert dispersions, for example: into coatings or adhesives. Therefore, EPDLA recommends using the established communication via SDS as long as products and information are transferred between industrial plants. The efficiency of this information exchange has been proven acceptable over decades.  Any reporting and/or labelling requirements would hit small and medium enterprises (SMEs) much harder than larger companies and the EPDLA is concerned that this may be unworkable for these smaller companies, either forcing them from business or reducing their capability for research into innovative products (see the file enclosed). |  | The definition of “solid” according to CLP is not directly applicable to (polymer) dispersions. Polymers and polymer dispersions do not exhibit a melting point, instead they exhibit a glass transition temperature. Dispersions which exhibit film formation at room temperature or lower must be considered to be liquid because, by definition, solid particles wouldn’t be able to from a film. Therefore, an additional criterion such as “particles that cannot alter their shape at 20°C are considered to be solid” could help to sharpen the definition when applied to dispersions.  Analytically, the lower limit proposed by ECHA is simply not enforceable. To be enforceable, a restriction must have limits that could be proven in mixtures with available analytical methods. On this basis, the lower size limit needs adaptation into the µm-scale. As one example, the identification of Microplastics with µ-FTIR in the environment reaches lower limits of reliability at only 5 – 10 µm due to scattering at the surfaces of particles. With µ-RAMAN spectroscopy measurements of 1-2 µm could be achieved. (Analyst, 2009, 134, 1586; Baseman, Microplastics Analyses in European Waters 2019, p. 18) Neither method is standard in analytical laboratories. In the Microplastics conference 2018, only a few studies were published where authors claimed to be able to identify the chemical basis for particles in the range of 500 nm. The proposed lower limit of 1 nm is far from any standard analytical capability. At 1 nm it is impossible to distinguish between a medium sized organic molecule of any kind and a polymer – and this is before even considering the determination of the chemical identity of a potentially present polymer.  According to REACH, a polymer is a molecule that contains a sequence of at least 3 monomer units, which are covalently bound to at least one other monomer unit or other reactant. With a C-C single bond length being generally 1.54 angstroms = 0.154 nm, only 7 covalently bonded carbon atoms in a molecule’s backbone (7 x 0.154 = 1.078 nm) would qualify such a small molecule as a microplastic if it contained 3 monomer units among the 7 carbons. The EPDLA questions how can it be justified that a polymer as outlined above, would fall under the MP Restriction while no such requirement would apply to identical e.g. C7/C8 or C9 hydrocarbons of natural origin? Furthermore, we question how molecule size can be measured this precisely and reliably on such an atomic scale to make the regulation enforceable?  Remark: Obviously, this argument is only applicable for mixtures where not only the size but also the chemical identity of the polymer has to be determined. For a pure dispersion (typically our products) where the chemical nature is known, the size can certainly be measured and there is always a more or less broad size distribution. So, in that case only an improved “solid”- definition (like above) would really help.  Proportionality - reporting  It is clear from the proposal that ECHA aims to target cosmetics, agro-applications, detergents and other applications where Microplastics are intentionally released down the drain or into the environment. The EPDLA supports efforts to reduce (micro)plastic pollution the environment by e.g. pushing for biodegradable solutions for such applications. Technical feasibility has to be commented on by relevant industry associations and companies. By focusing the restriction on these industries, more than 70 % of the estimated problem detailed in ECHA’s own document is already addressed within the restriction.  For the remaining 20 – 25 % of the problem calculated by ECHA, the restriction means the whole plastics industry shall be burdened with additional bureaucratic effort for labelling and reporting for pellets and dispersions even though only a minor part of this is estimated to contribute to the (known and accepted) problem. For industrial sites, emissions are controlled by local authorities. Industrial waste is, as a rule, treated as hazardous waste and direct release into municipal sewerage or surface waters is forbidden by law. Retail and professional users will not be obliged to report their usage, even though this is almost half of the dispersion-based paint market and a potential source for possible losses into the drain. (This will be addressed by user-guides. As an example, the paint industry will provide guidance how to treat used brushes and rollers properly, however there is no guarantee that users will follow this guidance). The EPDLA feels a significant amount of effort and cost is burdened onto industry, especially downstream users, and yet most of this measure does not actually target the issue.  It is well known, that the main “entry path” of microplastics to the environment is via secondary microplastics. The planned restriction will have almost no effect on such emissions into the environment. The EPDLA questions if the proposed restriction is legally proportionate.  ECHA estimates a 400 kt reduction of microplastics emissions in 20 years (20 kt/annum) through the planned restriction at a cost of €9.4 bn. This corresponds to only ca. 0.2 wt-% of the total amount of improperly handled plastic waste in the EU. The EPDLA believes the proportionality principle is violated in this restriction and suggests time and money would be more effectively spent supporting improving waste collection systems on a global scale.  **Answer to specific info request 3:**  Considering dispersions and taking into account their main uses in the market by volume, i.e. coatings and adhesives, EPDLA members are not aware of any application in these areas where such a low amount of an insoluble powder exhibits intended technical effects. We could see a meaningful level starting at 0.1 %, e.g. for matting.  Levels of polymer dispersions in applications for paints, coatings, and construction may well exceed 20%!  Applying unreasonable lower limits creates problems with existing analytical methods for mixtures and compromises enforceability and workability of the regulation (see the file enclosed |  |  |
| **36** | European Synthetic Turf Council (ESTC) | **Comment:**  ESTC is the EMEA Synthetic Turf Council, a non-profit trade association representing European, Middle East and African based companies manufacturing synthetic turf surfaces and the components used to form the surfaces and also companies that install and maintain synthetic turf surfaces. Members also include sports federations that use synthetic turf surfaces. The attached letter details ESTC’s initial response to the Annex XV restriction report - proposal for a restriction on intentionally added microplastics. In conjunction with our members we are currently collecting further information and propose to make a further submission to ECHA during the consultation period.  Synthetic turf surfaces used for sports, recreational or landscaping applications provide an attractive, hard-wearing, safe, low maintenance surfacing solution for many situations where natural turf alternatives are not cost-effective, feasible or sustainable. Our figures show there are over 17000 synthetic turf sports fields across Europe, each being typically used for over 58,000 playing hours per year, which equates to over 1000,000,000 playing hours or over 33,000,000 people participating and benefiting in sport played on synthetic turf fields.  ESTC recognises that as with any man-made product, a synthetic turf surface needs to be installed, maintained and finally disposed of in a way that minimises its impact on the environment. We agree that the potential effect of intentionally added microplastics on the environment poses a legitimate concern and call for control measures that have a clear scope, based on the latest scientific evidence on the hazards and risks associated with infills used in synthetic turf surfaces and brings the most benefit to environmental protection.  To increase awareness of these control methods ESTC, through its participation with the European Standards Committee (CEN), has advocated that CEN TC 217: Surfaces for Sports Areas, develop a CEN Technical Report to promote the design and maintenance features that will minimise/ eliminate the potential for infill migration from sports fields. This Technical Report will support European Standard EN 15330-1: Specification for Synthetic Turf Sports Surfaces. CEN is currently seeking approval of the National Standards Bodies to approve this new work item, and it is hoped that the Technical Report can be published by early 2020 latest.  ESTC is also in conversation with FIFA and World Rugby to see if the two international sports federations for sports that are the primary users of synthetic turf fields containing infill within Europe will endorse the containment processes being considered and incorporate them into their respective field certification programmes.  A major concern to those operating and using fields today that contain polymeric infills, is what any restriction on the production and supply of such materials will mean for existing fields.  A synthetic turf sports surface is designed to provide the sport’s performance and player welfare characteristics considered necessary to allow sports to be played satisfactorily and safely. It is the combination of synthetic turf carpet, infill and possibly an underlying shockpad that provide these properties. If any one component is changed the playing surface will not perform as designed and intended.  It is also important to consider that you cannot simply remove a polymeric infill and replace it with an organic infill. Many fields with polymeric infills, satisfy the sports performance and player welfare regulations due to the elastic properties of the infill. Organic infills provide limited or no impact attenuation properties so such a system needs to include an impact absorbing shockpad that is laid beneath the synthetic turf carpet. This means existing fields would have to be fully resurfaced, not just have the infill changed. Typically, this could be expected to cost at least €200,000 per field.  A synthetic turf playing surface is normally expected to last between eight and ten years. Through this period top-dressing of the surface with additional infill, to compensate for infill compaction etc is required. If polymeric infills are no longer available on the market the performance of fields will deteriorate more rapidly than field owners envisaged, and they will be faced with having to either replace the synthetic turf surface sooner than budgeted or close fields due to them becoming unsuitable for use. Failure to be able to top-dress fields will also invalidate many manufacturer’s warranties.  Significant time would be required to enable the infrastructure changes required to meet any enforced replacement of polymeric infills and this would greatly compound the availability concerns around organic infills due to the higher frequency need to replace / top-dress fields with organic infill.  ESTC fully recognises the need to reduce and prevent microplastic pollution and acknowledge that polymeric infills fall within the proposed REACH definition of a micro-plastic. ESTC does not believe the severity of microplastic pollution of the environment is as problematic as some have suggested especially if snow removal is undertaken responsibly. ESTC does believe that through good field design and maintenance the quantity of infill migration can be reduced even further and that through the promotion of good practice and the support of sports federations, funding agencies and national governments this approach can become the norm throughout Europe. With such policies in place ESTC believes that a ban on the use of polymeric infills becomes unnecessary and requests that polymeric infill materials for synthetic turf sports fields be granted derogation from the proposed REACH restriction. By granting derogation the risk of communities across Europe suffering negative social, health and economic consequences, through having reduced access to good quality sports facilities, is removed. |  | A number of studies have been made into how much infill migrates into the environment, but many appear to be based on assumptions, resulting in conclusions that the volume of infill that is applied as periodic topdressing of a field equates to the quantity being lost to the environment. Reality shows there are a number of different pathways and only limited amounts are released to the environment. Examples from Sweden, The Netherlands and Denmark repeat the real loss is much lower -ss our letter for more details.  Guidance on the ways of controlling infill migration was published by ESTC in 2017. This guidance is currently being reviewed and enhanced, an updated guide being ready for publication by the autumn of this year. By adopting a range of simple design features to ensure infill remains within the footprint of the synthetic turf pitch and ensuring fields are correctly maintained (something the industry has been advocating for many years), ESTC believes the risk of risk of infill migration can be significantly reduced and or eliminated in many cases. This approach is already implemented in a number of countries and advocated by various organisations. | The synthetic turf industry is also developing surfacing solutions that will reduce the amount of infill used in synthetic turf surfaces and its ability to migrate. Increasingly the market is asking for surfacing systems that include shockpads. Shockpads are designed to contribute to the impact absorption properties of the playing surface. By including shockpads in the surfacing system the need to have high volumes of infill within the surface is reduced. Shockpads also reduce the rate at which the infill compacts, meaning less frequent top-dressing is required. Initially driven by the desire to reduce the spectacle of infill splash on televised matches FIFA has developed a test that assesses the potential of excessive splash to occur. Systems with low splash characteristics will not suffer from infill migration to nearly the same degree as systems with higher infill splash.  Good maintenance, using the correct specialist equipment, is also an important consideration when addressing infill dispersion. The need to maintain synthetic turf surfaces is something the industry and sports federations both recognise and advocate. ESTC already issues guidance on appropriate maintenance procedures and is, again, in the process of reviewing and updating this to ensure that the need to consider the impact on the environment of poor maintenance is communicated. |  |
| **37** | EuPC (European Plastics Converters) | **Comment:**  EuPC is the leading EU-level Trade Association, based in Brussels, representing European Plastics Converters. EuPC now totals about 51 European Plastics Converting national and European industry associations, it represents close to 50,000 companies, producing over 50 million tonnes of plastic products every year. The European plastics industry makes a significant contribution to the welfare in Europe by enabling innovation, creating quality of life to citizens and facilitating resource efficiency and climate protection. More than 1.6 million people are working in about 50,000 companies (mainly small and medium sized companies in the converting sector) to create a turnover in excess of 280 billion € per year  II. Re: Paragraph 7 and 8: Reporting (and labelling) requirement  1. Raw materials used to produce plastics articles and their supply chain should clearly be indicated as out of the scope of the restriction  Taking into account the current definition of microplastics any uses of plastics pellets or powder used to produce polymers, masterbatches, compounds or plastics articles would be considered a use of microplastics at industrial sites. This use is still allowed at industrial sites (art. 4a) but labelling and reporting requirements would apply. This would affect around 50,000 companies, mainly SMEs.  It is our view that this measure is not appropriate nor proportionate for plastics pellets, powders , masterbatches, compounds and regranulates eventually used in the production of plastics articles. We therefore call for these uses to be exempted from the restriction labelling and reporting requirements.  **Answer to specific info request 5:**  Re: Paragraph 8: Reporting requirement, d) the quantity of microplastics released to the environment, either estimated or measured in the previous year.  Based on a survey across its members, it is EuPC view that the reporting requirement on pellet loss cannot bring meaningful results as long as a methodology for reporting is not defined. Taking into account the low levels of plastics losses compared to the overall production any reporting is likely to be well below error margins providing no useful information on actual losses during the use of pellets at individual sites. On the other hand, the additional cost for industry is estimated at 0.5-1 billion €/year if the measurement option is chosen(50,000 companies time an average cost of 10,000-20,000 €/year/company). This cost is clearly disproportionate in view of the usefulness of the information gathered, which if it follows the requirements of the restriction might well lead to knowing not more than the current knowledge. The result of the EuPC investigation are summarized here below.  2. Background : potential methods to estimate/monitor releases, reliability and associated costs  There are several known methods to estimate or measure the release however, as described in the following sections those methods would not reflect actual release of microplastics to the environment or not feasible to carry out. | Alternatively, companies should take precautionary measures to prevent the release of microplastics and report the implemented measures. The latter is actually more pragmatic. Rather than focusing on measuring very low and difficult to evaluate release, the focus should be put on preventing releases. Informing Authorities on this process would enable them to decide if further action is needed.  This leaves applying the measure on the basis of the precautionary principle (see Communication of the Commission on the precautionary principle ). However the following criteria for the application of this principle are not met by the proposed restriction : proportionality, examination of cost (incomplete, see reporting obligation below) and benefits (not adequately demonstrated), subject to review taking into account new scientific knowledge ( the restriction is not temporary). | The dossier submitter then goes on to consider microplastics as a non threshold substance, therefore assuming that a plastic pellet is as dangerous as an heavy metal, persistent organic pollutant or very toxic substances. The cost efficiency of the measure is then compared to the one of those substances (see e.g p. 126 of the annex XV report). The hazard/risk caused by such substances cannot be compared to the one of microplastics and the cost efficiency of the measure should be evaluated on its own. The fact is that there is no relevant benchmark to assess the proportionality of the measure and that therefore Echa could not justify the proportionality of the measure.  •  2. Definition of microplastics and feasibility of quantifying particle dimension of 1 nm:  Microplastics are defined as solid polymer particles with all dimensions 1 nm ≤ x ≤ 5 mm which covers not only micro but also nano range of particle. Although this includes a wide range of polymer particle sizes, there is no internationally accepted definition on the size limits for microplastics. Different size ranges are set by different groups and the lower size limit is more debated and in many studies the decision has been pragmatic and simply determined by the sampling device being used. With regards to the reporting requirement, this definition is confusing since quantifying nano-sized microplastics would not be feasible even with currently available technologies which allows identification and quantification of microplastics larger than 10-20 µm. Further, for the very controlled environment such as cleanroom the international ISO 14644 standard includes guidelines and limit value for contamination control in cleanrooms. Even the highest level of cleanroom standards, maximum concentrations are defined only for aerosol particles with diameter between 0.1 and 5 µm while nanoparticles are not regulated by the cleanroom standards. It is not feasible for most of 50,000 companies who would not even need a cleanroom to put such nanoparticles under their control.  3. The proposed measure is disproportionate in view of the impact on environment and human health of microplastics compared to other substances. No consideration on the impact of the measure is considered in the dossier  The restriction proposal does not identify a hazardous potential specific to the microplastics themselves (to the exclusion of additives which should be assessed individually). Likewise, the restriction concludes that ingestion of microplastics does not significantly enhance bioaccumulation of POPs relevant to other types of particulates present in the environment based on the current scientific consensus.  Actually, most of microplastics used at industrial sites (e.g. polymer pellets) are classified as not hazardous and safety communication such as the provision of Safety Data Sheet and labelling are not even required. Since impact on environment and human health are not clearly identified, a reporting requirement which is similar to the notification requirement for Annex XIV substance for an Authorised use is very far-reaching requirement for more than 50,000 plastics related companies which are using pellets at their site.  If reports from companies are considered to be necessary, its administrative burden and financial impact especially on small to medium size companies should be analysed in order to minimise the impact. This is not considered at all in the restriction dossier.  III. RE : Paragraph 8, Reporting requirement, a) identity of the polymer used in the previous year  The restriction dossier does not justify why this information is required. For the sake of simplicity we would suggest to remove this §.  IV. Re: Paragraph 8, Reporting requirement, b) description of the use of the microplastics  Some guidance should be provided here. The Use of the microplastics should be described as “Use at industrial sites”, more details would create issues related to confidential business information  V. Re: Paragraph 8: Reporting requirement, c) quantity of microplastics placed on the market the previous year  We question the proportionality of this measure for plastics pellets. This equates to the whole industry reporting what it produces. This raises issue of Confidentiality of Business Information. At the minimum tonnage bands should be foreseen.  VI. Re: Paragraph 8: Reporting requirement, d) the quantity of microplastics released to the environment, either estimated or measured in the previous year.  A more detailed analysis is provided under question 5. Based on a survey across its members, it is EuPC view that the reporting requirement on pellet loss cannot bring meaningful results as long as a methodology for reporting is not defined. Taking into account the low levels of plastics losses compared to the overall production any reporting is likely to be well below error margins providing no useful information on actual losses during the use of pellets at individual sites. On the other hand, the additional cost for industry is estimated at 0.5-1 billion €/year if the measurement option is chosen(50,000 companies time an average cost of 10,000-20,000 €/year/company). This cost is clearly disproportionate in view of the usefulness of the information gathered, which if it follows the requirements of the restriction might well lead to knowing not more than what is known today.  a. Review of Feasibility of mass balance calculation:  Mass balance calculation (e.g. resource efficiency calculation) is not a reliable method to obtain released quantity of microplastics since: 1) the amount of release such as spill is usually much lower than the allowed margin of error during the weighting of the plastic material. If companies report outcome of such mass balance, it will deliver errors in weight calculation which could be influenced by many factors but will not derive actual release of microplastics into the environment, 2) Further, according to the survey conducted for the impacted sectors, companies responded that they are not able to determine loss to environmental based on their resource efficiency calculation since loss in production and loss as waste are not differentiated.  There is also a study conducted by Nova institute (2014) which estimates a loss rate between 0.1-1% of total European plastics production. However, this is derived based as resource efficiency in production and it would include some forms of process waste such as offcuts. While resources efficiency may not account pellet loss quantity, if companies refer the loss rate as their release rate it would be an overestimation of the release to the environment.  b. Feasibility of measuring release to the environment and socio-economic analysis:  As reported above, quantification of nano-sized particles is technically not feasible. Further, to measure the release of more than 10-20 µm range of particles from industrial sites which could occur in long term in small quantity, identification of released pathway (air, soil and water) and sampling of released particles would be necessary to estimate the rate of the release. There are some studies related to methods of capturing microplastics in the environment but those are not established as practically applicable methods at industrial sites yet.  Whilst losses to water may in certain cases be monitored there are many other potential emission points.  By interviewing an internationally recognized laboratory, the very rough estimation cost of investigation of considerable method covering release pathway via air, soil and water for one sequence of sampling is minimum > 5,000 € per facility. E.g. microplastics analysis cost could be around 800 EUR per environment sample if SEM and FTIR imaging instrument (for polymer identification) are used. Based on typical cost for asbestos and soil investigation, labour cost for sampling could be estimated as > 3,000 € per facility.  There are many factors to increase the cost such as number of release pathways, sampling frequency and sampling quantities that overall cost can be e.g. 5,000 to 100,000 €. Financial impact especially on small companies could be considered to be critical even by considering 5,000 €/facility. However, the financial impact of measurement cost is not included in the socio-economic analysis and this should be analysed in advance to establish any standardized method. For the purpose of estimating the cost we selected cost towards the lower end of the range at 10,000 -20,000 €/year/company.  c. Referring to available studies for the release rate:  According to the survey within the EuPC membership regarding the reporting requirement, some companies responded that they would refer an available study to estimate released quantity. However applying any emissions rate from available studies would not estimate actual release quantity as described in the following. And if this method would be practical, there is no need of reporting from companies since volume of polymers used among the European market is generally available.  According to the Eunomia study (Hann et al., 2018), Mepex (2014) and Danish EPA (2015) studies represent the most reliable estimates of release of microplastics to date. Mepex studies estimates 0.09% of total plastics productions (0.05% from transport and 0.04% from processors). The transport etimation (0.05%) is based on the OECD emission factor which is based on the wost case scenario for dust generation from solid powders in general but the solid powders would not necessary cover different kind and size of polymers. The estimate from processor (0.04%) is based on emission from a single Norwegian polystyrene plant. Danish EPA study estimates on average 0.01% of raw material consumption at plastics facilities and maximum 0.0013% of raw material consumption for processors that have joined OCS as the spill rate. Those estimate is based on the survey conducted at 8 companies however, the resported spill rate could include solid and liquid waste and not clear if it represents pure release of micprolastics.  As described above and in the Eunomia study, those studies are based on limited evidence and have limited application and therefore, referring the study will not represent actual release of microplastics. Many factors such as facility design, type of materials used, implementation of measures and providing of trainings to facility operators would make difference to the release rate. Further, when release into aquatic environment is the concern, geographical settings should be also accounted.  It may also be questioned to which extent it is useful having thousands of companies referring to a study already known by Echa…  Ref: Hann et al., 2018: http://ec.europa.eu/environment/marine/good-environmental-status/descriptor-10/pdf/microplastics_final_report_v5_full.pdf  3. Reporting frequency:  As described above if reporting is required for every year, measurement cost per facility will cause huge impact especially on small and medium sized converting companies who are the majority of more than 50,000 plastics related companies in Europe even if we consider the minimum cost of 5000 €/measurement campaign. Socio-economic analysis should be carried out first and depending on identified financial impact, a lower frequency of reporting requirement should be considered for medium to small companies. | Whilst we acknowledge and support the need as good house fathers to minimize emissions of waste, including microplastics in the environment, we would like to comment specifically on the restriction proposal.  I. The scope, the validity and proportionality of the restriction measure is not adequately defined and justified.  • REACH foresees restrictions to be applicable to specific substances on their own, in a mixture or incorporated into an article, whilst plastics covers a broad range of substances and compositions, the only commonality being to a certain extend some common physical properties such as form, specific density and limited biodegradability although the variation across plastics would be very important and the same type of properties could then also be attributed to other materials.  • A restriction under the REACH regulation is therefore not an adequate instrument and this can be also inferred from the weakness of the argumentation supporting the restriction as this assessment is unspecific.  • The proposed restriction is discriminatory to plastics : to be non-discriminatory the measure should specify physico-chemical (including biodegradability) properties ranges for any material (also in order to avoid “regrettable substitution”).  • The annex XV dossier further fails to demonstrate adequate control or proportionality of the envisaged measures.  • The dossier submitter recognizes that there is not sufficient information enabling to conclude to plastics toxicity other than physical hazard through the ingestion of some plastics (by the way not necessarily microplastics) nor that those microplastics have a potential for bioaccumation, but still bases all the evaluation of the restriction by considering microplastics as similar to Persistent Bioaccumulative and Toxic (PBT) or very Persistent and very Bioaccumulative (vPvB) substances. It is evident from the dossier itself that the criteria for PBT and vPvB substance do not apply as it is impossible to characterize such a wide range of products toxicity or bioaccumulation potential. We therefore require that any reference to PBT/vPvB like similarity are removed from the Echa annex XV dossier as it may at a later stage create confusion whether or not those microplastics fall under the article 57 d or e of REACH or impact customer behaviour.  1. Will the information gathered be reliable and useful? Are there alternatives?  The proposal does not include a methodology to estimate/measure quantity of microplastics released to the environment. Without a standardized methodology, defined order of magnitude of released quantity (e.g. report order of 0.001 kg or 10 kg) and definition of “no release”, the volumes reported by companies would not reflect actual release of microplastics to the environment ranging at best from incomplete assessment based on heterogeneous method to guesstimates, none of which will give a clearer picture of releases of pellets during use.  Before imposing an obligation to quantify the release of microplastics from industrial facilities, a reliable and standard method should be established first and in a second step a socio-economic analysis of the proportionality of such reporting based on the developed method.  In the meantime or alternatively, companies should take precautionary measures to prevent the release of microplastics and report the implemented measures. The later is actually more pragmatic. Rather than focusing on measuring very low difficult to evaluate release, the focus should be put on preventing releases. Informing Authorities on this process would enable them to decide if further action is needed. |  |
| **38** | EWIMA |  |  | EWIMA comments on the ECHA proposal for a restriction of microplastics  Definition of the size of microplastic particles  ‘Microplastic’ is defined as a material consisting of solid polymer-containing particles, where ≥ 1% w/w of particles have (i) all dimensions 1 nm ≤ x ≤ 5 mm, or (ii), for fibres, a length of 3 nm ≤ x ≤ 15 mm and length to diameter ratio of > 3. The lower limit of the particle size is very low. This is not suitable for small and medium sized companies in EU as it is technically highly demanding to evaluate particle sizes around 1 nm – 50 μm. Nearly every polymer would be affected and currently manufacturers of inks in EU would have no ways for substitution in their formulations. This would result in disadvantages for companies in EU with regard to their non-EU competitors as custom controls would not be able to control the imports and it´s conformities to these particle sizes (1 nm – 50 μm).  In addition, ‘nanomaterial’ is defined as particles in the size range of 1 – 100 nm. In order to distinguish ‘nanomaterial’ particles and ‘microplastic’ particles, the lower size definition of ‘microplastic’ particles should be > 100 nm, and the corresponding minimum length of microplastic fibres 300 nm.  Impact of a restriction of microplastics  Microplastics greater than 0.01% w/w are part of some inks manufactured for writing instruments industry.  The proposed restriction of microplastics will require from manufacturers of stationary products labelling of mixtures containing microplastics (e.g. paints, inks, glue) and reporting of the use of microplastics and mixtures containing microplastics at the industrial site. Labelling and reporting requirements can be seen critical as it will create costs for manufacturers without significant benefit.  Inks of writing instruments and related components/products include for technical means dispersions of fillers, binders and thickeners which are polymer-based. They are essential for the function of the product as they support technical properties e.g. film forming. As they disappear when they form a film, those functional polymers are derogated based on paragraph 5(b).  Some inks and for instance paints, glues, correction fluids can also be considered as derogated based on paragraph 5(c): Substances or mixtures containing microplastic where the microplastic is permanently incorporated into a solid matrix when used.  For inks and for instance paints, glues and correction fluids labelling requirements apply, as it cannot be excluded that unconsumed material will be released via down the drain to the environment.  The labelling of inks, paints and glues can be a useful instrument to provide the end consumer with product related information. It is intended to inform the consumer how to dispose the product properly to minimize the release to the environment by labelling.  More detailed labelling of products will not increase the consumers’ attention. Moreover, it makes more sense to inform the end consumer by national or regional disposal organizations with regard to proper disposal.  Reporting obligations  Despite of derogation 5(b) or 5(c), some inks and for instance paints, glues and correction fluids are submitted to declaration requirement.  Despite of derogation 4(a), Polymers, or mixtures containing microplastics that are used at the industrial site, are submitted to declaration requirement.  Any downstream user using a microplastic derogated from paragraph 1 on the basis of paragraph 4(a), 5(b) or 5(c) must report to ECHA the identity, the description, the use and the quantity of the microplastic used. ECHA will use the data sets for further assessments of risks for human and environment and observance of the chemicals market.  A definition of ‘industrial site’ is missing. Providing that each non-private user (= commercial user) of ‘microplastic’ is an industrial user, this reporting obligation will result in high additional bureaucratic efforts and costs – without any benefit for the environment. |  |  |
| **39** | Fachvereinigung Lebensmittelzusatzstoffe im Verband der Chemischen Industrie e. V. (VCI) | **Comment:**  The ECHA proposal to restrict microplastics within the REACH regulation also covers specific food additives, which fall under the proposed definition of microplastic. The use of E1205-E1208 in food supplements is mentioned explicitly in the dossier. The measure foreseen is a restriction, with no transition period.  4. Exemptions for food additives are required, technical substitutes often not available:  Since food additives are fully regulated under Regulation (EC) No. 1333/2008, including environmental factors, we consider that there is no reason to regulate uses of food additives in a REACH restriction for microplastics. Therefore we see a strong case to exclude this product group from the scope of the intended restriction measure. Furthermore we want to highlight that one essential part of the authorization of a food additive is that there is a reasonable technological need that cannot be achieved by other economically and technologically practicable means (Art. 6). Also in practice it is often not easy to switch from one additive to another one since the effects are often very specific to the food additive and the application. Reformulation of final foods is therefore not always easily achievable and needs extensive and expensive testing before alternative products of same quality are developed. This would add high burden to those companies that might have to substitute affected food additives.  5. Transition periods have to be sufficiently long to phase-out certain materials:  If food additives would have to be substituted because of future restrictions of microplastics, sufficient transition periods are needed to help companies to develop new, safe and stable products. Furthermore, we want to stress that the development and authorization of new food additives is a complex, costly and time-consuming process. |  | The "Fachvereinigung Lebensmittelzusatzstoffe im Verband der Chemischen Industrie e. V. (VCI)" [VCI sector group food additives], representing the interests of companies in the food additive industry operating in Germany, comments as follows:  1. Double regulations for food additives have to be avoided:  Food additives are fully regulated in the EU by Regulation (EC) No. 1333/2008. This regulation lays down rules on food additives to ensure the effective functioning of the internal market whilst ensuring a high level of protection of human health and a high level of consumer protection, including the protection of consumer interests and fair practices in food trade, taking into account, where appropriate, the protection of the environment (Art. 1). The protection of the environment by the use of food additives in foods is clearly in the scope of the food additives regulation. According to Art. 6 of said regulation a food additive may only be included in the Community list of approved food additives if it meets several conditions, including environmental factors. In our opinion Regulation (EC) No. 1333/2008 fully covers environmental risks originating from the use of food additives. Therefore a specific rule on microplastics covering food additives would create a double regulation in the EU which has to be avoided. | 2. Restrictions to specific sectors shall be proportionate:  In general, food additives are added to a food only in small quantities to fulfil a desired technological function. The number of food additives that might be considered as microplastic is very limited, e.g. some polymers that are insoluble in water. Additionally, the use of these food additives is usually very specific and the authorization often very specific, e.g. limited to food supplements. Overall, the release of these food additives to the environment is considered rather small compared to the release of microplastics from other sources. Measures that are planned to minimize the entry of microplastics into the environment have to be proportionate, especially if specific product groups are technologically needed to improve the quality and the safety of foods.  3. Definition and scope has to be clear and water-soluble polymers should be excluded:  The current definition is still not clear and leaves broad room for interpretation. To our understanding the scope would cover a broad range of polymers, including natural polymers, which are not considered as “plastics”. Also the examples of food additives given in the ECHA report that are used in food supplements (E1205-E1208) are not providing much clarity since some of the polymers are water-soluble while others are insoluble. In our opinion water-solubility should be a key exclusion criteria in the definition. |  |
| **40** | Fertilizers Europe | **Comment:**  Fertilizers Europe welcomes the alignment of the current restriction proposal with the Fertilising Product Regulation regarding the biodegradability of polymers used, however would like to provide more and, in some cases, new information on:  -tonnage  -specific biodegradability criteria for polymers used in our industry  -transitional period for non-CE marked fertilizers to be aligned with CE-marked fertilizers  Furthermore, new available information on the quantities emitted have been provided in Part I and Part II of the attached document  **Answer to specific info request 3:**  a. more detailed information are given in the 2 Questionnaires attached as confidential  b. this question aims to set even a lower boundaries. Also unintentional releases from packaging materials to products cannot be excluded, consequently we believe that the restriction proposal will not be enforceable in practice  c. no accepted methods to test for polymers in fertilizers are available |  | **Answer to specific info request 1:**  Fertilizers Europe industry and 2 expert partners are developing an accelerated biodegradation test in soil, results will be available in Q3 2019. ECHA and European Commission will be informed about the results of the test.  **Answer to specific info request 5:**  a. specific information on tonnages for microplastics used in anti-caking/anti-dusting agents and control release fertilizers (CRF), their technical function and releases to the environment have been provided in the Table 1 on page 8 of the document attached as non-confidential  b. Fertilizers Europe members are downstream users and not polymers producers. CRF and anti-caking/anti-dusting improve the physical behaviour of fertilizers with advantages along the supply chain.  c. no biodegradable alternatives currently exist for anti-caking/anti-dusting agents and they have to be developed by industry. For CRF criteria have been already requested by the Fertilising Product Regulation in CMC 9 and art.42 and first test results will be available by industry in Q3 2019  d. The alternative suggested by ECHA in the Restriction report could have an effect only in combination with few specific type of fertilizers |  |  |
| **41** | FFI Fachverband Faltschachtel-Industrie e.V. |  |  | **Comment:**  The German Folding Carton Companies as industrial users of paints, coatings and printing inks do neither have any information – e.g. on the identity of the polymers used in printing inks or varnishes – nor would obtain such details from their upstream suppliers (printing ink manufacturers. The reason is that otherwise confidential business information would need to be disclosed. However, without such particulars the folding carton industries cannot report any information, for instance, about the identity of polymers. Ideally, the upstream business operator from the chemical industries should provide the necessary information to the authorities. |  |  |
| **42** | FIDE - Federation of the European Dental Industry | **Comment:**  Definition of microplastics:  The proposed definition of microplastics is very broad and is not generally accepted. Definition of microplastic has to be precised, because not all polymers (in the relevant size) are microplastics (every plastic is a polymer but not every polymer is a plastic). Furthermore, no standardised methods are known for the qualitative and quantitative analysis of microparticles in the environment and diverse products.  Transitional period:  Medical devices are used in a large scale in the EU and most other regions of the world regulated. In the EU the Medical Device Regulation (MDR, Regulation 2017/745 of April 5, 2017) requests in many places that a risk management according to EN ISO 14971: 2012 Medical devices - Application of risk management to medical devices (ISO 14971:2007, Corrected version 2007-10-01) must be in place before any medical device can be brought on the market. This standard mentions in the introduction and many other places that all environmental risks must be considered and reduced to the minimum possible before placing on the market: “Risks can be related to injury or damage, primarily to the patient, but also to the operator, other persons, data, property, other equipment and the environment.” Based on these requests medical devices can be regarded to be similarly safe for the environment as medicinal products.  In case where an exemption would be granted, it has to be noticed that Medical Device Regulation (MDR, Regulation 2017/745 of April 5, 2017) requests in many places that a risk management according to EN ISO 14971: 2012 Medical devices - Application of risk management to medical devices (ISO 14971:2007, Corrected version 2007-10-01) must be in place before any medical device can be brought on the market. This standard mentions in the introduction and many other places that all environmental risks must be considered and reduced to the minimum possible before placing on the market: “Risks can be related to injury or damage, primarily to the patient, but also to the operator, other persons, data, property, other equipment and the environment.” Based on this request medical devices can be regarded to be similarly safe for the environment as medicinal products.  In addition, the MDR regulation (Regulation (EU) 2017/745 of the European Parliament and oft the Council of 5 April 2017 on medical devices,…) provides in Annex I (General safety and Performance Requirements) in 14.7 requirements with respect to waste and its safe disposal and also the requirement that “such procedures shall be described in the instruction for use”. In general, medical device have an instruction for use (IfU), and details of information in the instruction for use for safe disposal of the medical device are defined in Annex I in 23.4 (v) (and it is also defined that this information should be available upon request even an information of use is not required according to Annex I 23.1 (d)). In addition, in Annex I 23. detailed requirements for label and IfU are stipulated.  This means medical devices under MDR regulation have already detailed information in the IfU available, and therefore an additional labelling (paragraph 7) for medical devices derogated from paragraph 1 of Annex XV Restriction report for intentionally added microplastic based on paragraphs 4(a), 4(b) or 5 is not necessary and appropriate.  Additionally, as mentioned in the Annex to the Annex XV Report on Microplastic medical devices containing polymeric particles have only industrial or professional uses. This is true as well for the dental materials. Substance based medical devices even if used by consumers are used under a regime similar to medicinal products.  Based on these considerations we would ask for an exemption of medical devices similar to the exemption given for medicinal products (Restriction Report, Table 3, 4b).  As far as no exemption is given for any reason, we would ask for a prolongation of the entering in force for medical devices of EiF + 6 years based on following considerations:  First, medical devices cover a very broad field of products, e.g. substance-based medical devices, wheelchair, pacemaker. For some products, e.g. dental base materials, substance-based medical devices, there are no alternatives for synthetic polymers used. In the rare case an alternative is available, any change of formulation of medical devices requires a new conformity evaluation (e.g. generation of pre-clinical data, animal tests and clinical data, risk management) and potentially a new registration in other parts of the world. This takes time and could lead to supply shortages.  Secondly, manufacturers of medical devices are currently implementing the new Medical Device Regulation (regulation (EC) 2017/745, MDR) which is applicable on 26 May 2020. The implementation of the new MDR regulation is a huge challenge for the whole medical devices industry, and also for the notified bodies, till 27 May 2024. For now, there are not enough notified bodies certified to comply with the MDR. There is also the need to take into account the MDR transitional provisions. Indeed, according to Article 120 paragraph 2 MDR certificates issued by Notified Bodies in accordance with the Medical device Directive (MDD) shall remain valid until the end of the period indicated on the certificate, which shall not exceed five years from its issuance and shall however become void at the latest on 27 May 2024. Manufacturers of medical devices with a MDD certificate will then have to implement the MDR requirements.  For these reasons, a derogation of 2 years for this restriction is not realistic. More realistic would be a derogation of at least 6 years after entry in force (as for ‘leave-on’ cosmetic products), so that manufacturers can have the time to change the formulation of their products and also to implement the MDR requirements (taking also into account manufacturers of medical devices with a MDD certificate which have a transitional period according to MDR).  For the same reasons, a derogation of [EIF +18 months] (point 7, table 3) for medical devices falling under the exemptions of point 5 (table 3) is largely insufficient and would need to be extended to at least [EIF +3 years] after the first label/IFU updates under the MDR has been completed. |  |  |  |  |
| **43** | Finnish Association of Construction Product Industries | We thank Echa for the proposal and share Commissions view that plastic and microplastic releases to the environment need to be prevented.  • Microplastic restriction proposal is, however, different to other REACH restrictions in that the reasoning is mainly based on precautionary principle. The EU wide unacceptable risk to health or environment is not validated in the same accuracy than is done for substances.  • Could there be other approaches to achieve the target? If article 69 of REACH is not followed the restriction may lead to unpredictability of legislation and be precedent for other chemical restrictions without justification for unacceptable risk.  • Any measure to prevent the releases of plastic and microplastic to the environment needs to be proportionate with results achieved and costs created. With materials like plastic or polymers the measures should not be tailored against the well functioning material but against the release.  • The prevention of the release is the task of the whole supply chain including the end users. To be effective, any measure should be clear with wording and content. Every actor should be able to understand their duties and these need to be enforceable to ensure level playing field for companies.  • If REACH restriction is finally chosen to be the instrument to first time tackle the complex issue of plastic and minimise the releases to the environment, we propose that following considerations are taken:  • The definition of microplastic should be generally understood. The proposed draft definition is somewhat confusing, as it combines the dimensional definition, polymer content in particles and content of those particles in mixtures and the physical state not well applicable for polymers.  • The scope of restriction proposal is very broad, as it covers any use of any material fulfilling the definition. There is the risk, that many applications are banned which do not have potential for releases of microplastics to the environment.  We propose a step by step approach.  • In first phase, the microplastic restriction to cover microbeads and capsuled fertilizers/plant protection products with appropriate time allowed to move to biodegradable substitutes.  Combined with the abovementioned, we support the list of exempted applications in the draft restriction.  • The EU manufacturers and importers should be treated equally. The draft restrictions stipulates quite heavy reporting requirements for supply chains in the EU for exempted applications. Articles of polymeric material imported to the EU are produced without these requirements. The annual reporting of products is envisaged to create significant administrative burden with benefits still to be identified.  Proposal of change on derogations:  5c . Substances or mixtures containing microplastic where the microplastic is permanently incorporated into a solid matrix or encircled by a solid continuous material or structure when used. |  |  | In subsequent phases, conduct further studies and impact assessment for other applications. If these show release potential and unacceptable EU-wide risk from those applications, these are added to the scope of restriction. |  |
| **44** | Flock Association of Europe e.V. | See attachment. |  |  |  |  |
| **45** | Food Supplements Europe | Food Supplements Europe comments on the ECHA microplastics report  Date: 20 May 2019  Food Supplements Europe is the representative organisation of the European Food Supplements sector. It represents manufacturers and sellers of food supplements and their ingredients.  Food Supplements Europe appreciates the possibility to comment on the proposed measures to limit the exposure of microplastics in the environment. We do take the impact of our products on the environment seriously, including if there would be documented unintended negative environmental aspects originating from use of substances currently approved and used according to existing legislative requirements.  We therefore offer ECHA the following comments and suggestions at this stage of the consultation process. These comments will be refined and clarified further at consecutive stages of the consultation.  Food supplements in relation to the scope of the envisaged measure  Food supplements are products regulated under the legislative framework of foodstuffs. They are defined by Directive 2002/46 as foodstuffs containing nutrients and other substances, presented in dose form and intended to be consumed in small unit dose. Intake of food supplements in absolute quantities is therefore low (typically a few grams per day at max).  The substances identified in the report as being microplastics are food additives that have all been authorised according to the requirements laid down in the food additive legislation (Reg 1333/2008). These requirements focus on safety aspects related to the consumption of additives and include environmental factors as legitimate factor to authorise food additives (Article 6). There is therefore no need to address environmental aspects in separate legislation.  Definition and scope require clarification  The definition of “Microplastic” is not clear and leaves room for interpretation. It covers a broad range of polymers, not all of which are plastics. Given ECHA’s broad definition on microplastics, not only the identified additives, but also additional additives commonly used in food supplements e.g. as binders or disintegrants fall into the scope of the ban. It can legitimately be questioned if such additives are microplastics and therefore the definition should be made more specific.  Also, if the derogation under point 5b covers substances that in the finished product permanently lose their microplastic characteristics (i.e. no longer fall under the definition), a restriction, labeling or reporting to address the environmental concern is no longer relevant and thus should not be applied. It may even be misleading for consumers to require the labelling of microplastics on finished products while the products no longer contains microplastics.  The ban should be reconsidered in the light of the technological need of the additives covered and the burden of reformulation  The report lists four food additives as examples of microplastics (E1205-1208). Also other similar additives could be considered in the same way, such as E1201 and E1202 (Polyvinylpyrrolidone (povidone) and polyvinylpolypyrrolidone (crosspovidone)).  The food additives under consideration are not only used as controlled-release and taste-masking agent, with a film forming function in food supplements, as indicated by ECHA. They have a wider spectrum of functions in standard food supplements and not only in controlled-release forms. They are used for example in tablets as binding agents and disintegrants for immediate-release solid forms and they play a critical role in the product formulation and product properties.  No functional alternatives with the same technological functions have been identified. The use of starches or sugars as binding agents, increase the volume of the tablets and involve water, negatively affecting the stability of the product and increasing degradation. The use of starches as disintegrants has the same disadvantages, i.e. products becoming too big to be swallowed and affecting the stability of the shelf life. Starch and silicate derivatives are not soluble, produce undesired foaming and do not meet the efficacy standard for disintegration time.  Should the ban be implemented, a sufficiently long transition period should in any case be foreseen, similar to what is accepted for cosmetics, to avoid market and supply disruption.  The restriction should not discriminate food supplements  The identified substances (E1205-E1208) are food additives that have been authorised in accordance with EU legislation for use in food supplements. They are safe, necessary and used in accordance with the specified conditions of use.  The measure foreseen is a restriction, with no transition period. This means a de facto ban of food supplements using these additives immediately at the entry into force of the restriction.  Food supplements have the same galenic form as medicinal products and the technological functions of these additives are similar in both product categories, Yet, the use of these substances in medicinal products is subject to labelling and reporting and not to restriction. This introduces discrimination and unequal treatment between both uses.  This is all the more relevant, since a specific product can have a different regulatory status depending on the Member State, e.g. food supplement in one and medicinal product in another. The same galenic forms should not be affected by a restriction proposal in a different way, just depending on their regulatory status.  Food supplements, using the same ingredients as medicinal products should therefore at least benefit from the same derogation as medicinal products.  Conclusions:  The restriction covers a number of food additives that have been authorised and are indispensable for the manufacturing of food supplements.  The release of these substances is not likely to result in a significant burden on the environment because of the small unit dose form of food supplements. The ban however is likely to affect many manufacturers, require a lot of research and development resources and may in the end see products removed from the market.  Similar products under the framework of medicinal products are not covered by the restriction, but subject to labelling and reporting. Both categories should be treated equally.  Substances that lose their characteristics of microplastics in the final product should not be covered by any restriction, labelling or reporting requirements.  Before the restriction is applied to food supplements, a thorough impact assessment of burden vs benefit and appropriateness should be carried out. |  | In addition, some of the substances identified in the area of food supplements (such as E 1201, E 1208 and E 1209) are soluble in water and should not be considered as microplastic, despite these seemingly falling under the definition. We would ask ECHA to consider the extent to which substances that are water-soluble lose their properties to be considered as microplastic and are no longer released as microplastics in the environment. There may be scientific justification to explicitly exclude solid polymers containing particles which are water-soluble from the scope.  • Food supplements are products intended to be consumed in small unit dose form (a few grams per day at max).  • Foods Supplements are consumed by a limited part of the European population (18% according to the most recent Belgian food consumption survey)  • Additives E1205-E1208 are allowed under specific conditions of use i.e. only in solid forms- except chewable forms- with maximum levels set by the Regulation 1333/2008.  • Solid forms represent only a part of all the dosage forms available on the European market for Food Supplements, e.g. Syrups, Sprays, Ampoules, Drops.  Therefore, the measure when applied to food supplements will have a limited impact on the problem of microplastic. While the contribution of food supplements to the reduction of microplastics in the environment is likely to be limited in terms of cumulative overall volume. It would have a great impact on companies as many companies may be affected for part of their product portfolio. The resources needed to address this by reformulation or other means would not be proportionate. | The ECHA proposal now specifically introduces environmental effects as a criterion for the additives to stay on the market. However, ECHA has in our opinion not presented any data demonstrating these additives’ environmental effects. The report does not indicate that an environmental risk assessment has been conducted by ECHA taking into account the consumption pattern of food supplements and the actual use levels in food supplements of the additives in question.  The ECHA’s proposes a complete ban of the use of these additives in food supplements and does not even foresee a transition period that would allow reformulation of products currently on the market and regularly used by European citizens.  We therefore believe that the ban proposed by ECHA of certain additives in food supplements is not proportionate and appears to go against the Commission’s intention of less but better regulation since it will result in the immediate and irrevocable removal of established products from the market without there being documented environmental risks.  Reformulating of all product would require considerable time and effort and may affect product quality, efficacy and safety, which requires extensive testing. It is expected to add to the burden for the affected companies and in the end may result in removal of products from the market, which would not be proportionate.  The development of alternative technological additives would require important efforts in research and development. If this would be required for food supplements, this would create a considerable hurdle and disproportionate costs, keeping in mind that over 95% of all companies affected are small and medium sized.  The measure covering food supplements is likely to have a minimal impact on the release of microplastics in the environment.  It has not been possible to collect quantitative data on use within the narrow timeframe since the publication of the report. However, considering the following points the intake of these food additives, and thus the ultimate release in the environment is likely to be small. |  |
| **46** | German Olympic Sports Confederation (DOSB) and the German Football Association (DFB) | Called for a 6-years transition period before the proposed EU ban on intentionally added microplastics in synthetic turf is enforced (Oziel, 2019). |  |  |  |  |
| **47** | Global Silicones Council | Executive Director, GSC  Global Silicones Council (GSC) Comments on Proposed EU Microplastics Restriction  Microplastics should not be considered non-threshold materials for the purposes of risk assessment.  The proposal’s assumption that microplastics should be treated as non-threshold substances for the purposes of risk assessment, is not supported by the information presented in the restriction proposal and is inconsistent with the available science. To justify assigning a non-threshold status to microplastics for purposes of risk assessment, the proposal argues that an important property of microplastics when considering appropriate risk assessment is their ‘extreme’, arguably permanent, persistence in the environment. The proposal suggests that 'extreme persistence' creates the potential for a non-reversible pollution stock associated with the potential for environmental and/or human health risks without a full scientific assessment. The concern with this assumption is that laboratory based assessments of media half-life are not equally predictive for actual environmental persistence for all compounds and do not necessarily consider the range of degradation mechanisms (chemical, ultraviolet, physical, etc.) that could be operative in real-life scenarios. In addition, environmental persistence is an exposure characteristic, the importance of which is mediated by a number of other considerations including the potential toxicity of the compound under consideration.  The proposal also provides evidence that contradicts the concept that microplastics should be treated as non-threshold materials as it acknowledges that concentrations of microplastics observed to affect organisms via water in laboratory studies are generally much higher than concentrations measured in the environment. This demonstrates that the primary factors that will determine the risk associated with microplastics in the environment can be measured, and these materials should not be considered non-threshold substances.  The proposed scope of the restriction is too broad and should be narrowed considerably.  ‘Polymer containing particles should be better defined in the restriction proposal. While the restriction proposal under 2. d) (I) uses as a criterion a continuous polymer surface, under 2 d) (II) this criterion is dominated by a more general definition – the 1% polymer criterion. The criteria for “polymer containing particles” are overly broad, and would likely include a number of materials that would not meet the definition of microplastic.  Liquids should be unambiguously considered outside the scope of the proposed restriction. Dimethicone (polydimethylsiloxane (PDMS) and amodimethicone are liquids according to the CLP definition and clearly out of scope of the restriction. For clarity with regulated parties, liquids should be identified as outside the scope of the restriction.  Silicone polymers provide immeasurable socioeconomic benefits.  Silicone polymers are extremely valuable commercial materials socioeconomically. Silicone polymers contribute greater than $8 billion USD (AMEC Silicone Socioeconomic Study, 2015) in value to the global economy. The benefits afforded by silicone polymers in Europe in terms of product innovation capability and job creation cannot be understated. Silicone polymers are used in a number of societally critical applications including transportation, construction, electronics, and health care. The socioeconomic impacts of the proposed restriction could have implications well beyond Europe due to global supply chains and the impact of the restriction would likely be disproportionate to any benefits. |  | There are a number of naturally occurring compounds (e.g., sand, clay, and silt) that are 'extremely persistent’, in the environment, and can exist in the size range that microplastics have been defined in the proposal.  In our view the concerns linked to presence of microplastics in the environment, as detailed in this dossier, are not sufficient to argue that there is a risk. Hazard and exposure data presented provide evidence that a quantitative risk assessment can be conducted for these materials. Several of the studies cited in the proposal demonstrate a traditional risk assessment approach for microplastics is warranted. For example, the Bessing et. al. 2018 study that is referenced in the proposal identifies 168 effect thresholds for aquatic species obtained from 66 studies. In addition, the Burns and Boxall 2018 study concludes that the comparison of measured environmental concentrations with effects endpoints does not support that microplastics are negatively impacting the health of organisms in the environment. The Burns and Boxall study also found that concentrations of microplastics seen to cause effects on organisms are orders of magnitude higher than concentrations of microplastics measured in the environment. Another study cited in the proposal, (Lusher et al.) notes that field studies on wild populations document only the ingestion of microplastics, with no evidence of negative health effects in aquatic organisms or at the population/community level. The proposal accurately concludes that the available literature provides ‘only limited evidence that risks are occurring in the environment, despite ingestion and the presence of microplastics in organisms across different trophic levels’. This further demonstrates that the societal consequences associated with the proposed restriction, including disruption to global supply chains and deterioration to innovation would be extremely disproportionate relative to the current evidence of harm to the environment provided in the dossier. It is critical that the restriction proposal include only those substances that have been evaluated individually based on their characteristics, uses, potential for environmental releases, and human health and environmental risks.  The chemical identities of the specific microplastics listed, and their associated hazard characteristics, need to be more explicitly defined in the proposal. Particle size alone, is insufficient to determine the applicability of the proposed restriction to a specific microplastic given the range of materials in commerce that could fit the proposal’s broad definition. Further, it is more likely that the chemical characteristics of a compound will drive its toxicological profile and environmental fate rather than just size.  In addition, many of the complex hazard properties associated with microplastics that are cited in the restriction proposal would be similar for naturally occurring materials that exist in the environment in the size range of microplastics if particle size, persistence, and degradation are the most important determinants for hazard. The magnitude of the exposure to biota from these naturally occurring materials would be expected to be far greater than exposure to biota from manufactured microplastics.  Polymers that don’t meet the definition of microplastic should be explicitly excluded from the restriction proposal.  Film formers and gels should be outside the scope of the restriction dossier. As stated by ECHA, these materials do not satisfy the definition of microplastics since, during their use, the physical properties of these polymers are permanently modified such that the polymers either cease to exist or ‘swell’ in contact with water or a solvent such that they are not solid particles. To include polymers of this type in the scope of the Annex XV dossier would be inconsistent with the stated goal of the restriction, because the use of these polymers in commerce will not result in the release of microplastics to the environment. Silicone polymers that are film forming agents (e.g., trimethylsiloxysilicate, soluble polymethylsilsesquioxanes), and gels (e.g., cetearyl dimethicone/vinyl dimethicone crosspolymer) should be removed from the restriction dossier and supporting documents, and labeling and reporting requirements should not be necessary for these materials. To make it clear that these polymers are not included in the scope of the proposed restriction, the phrase “on end use or disposal” should be added to the definition in Table 3, paragraph 2(a). Without this important addition, the annex XV proposal would apply to numerous products that effectively cease to exist as a microplastic particle at the point of use. This would result in significant socio-economic costs to commerce, as well administrative burden on ECHA, without an equivalent benefit to human health or the environment. At a minimum, polymers derogated under paragraphs 5(b) and 5(c) should be exempt from labeling and reporting requirements, as they are transformed during the production process and effectively cease to exist as a microplastic particle at the point of use. As currently proposed, the labelling and reporting requirements for derogations will create a significant burden for industry.  Other silicone polymers that should be excluded from the scope of the restriction include polymethylsilsequioxanes, antifoams such as PDMS treated silica (also referred to as simethicone), and dimethicone silylate (silica dimethicone silyate) . These materials provide invaluable socioeconomic and product performance benefits in commerce and cannot be easily replaced in the applications in which they are used. These materials are typically used in very small amounts in product formulations and their relative contribution to the environmental release of polymers in the microplastic size range is relatively small.  **Answer to specific info request 6:**  Soluble polymethylsilsesquioxanes  In response to ECHA’s specific information request, we offer the following information on soluble polymethylsilsesquioxanes.  It is expected that soluble polymethylsilsesquioxanes (dissolved in non-polar solvents) behave in a similar manner to trimethylsiloxysilicate, which is not a particulate form at the point of use nor does the material form particles upon application, and therefore should be out of scope of the microplastic definition. Furthermore, soluble polymethylsilsesquioxanes are dissolved in solvents during application and do not meet the definition of a particle. They do not exhibit clear boundaries and are liquids at 20oC at a standard pressure of 101.3 kPa. Therefore, soluble polymethylsilsesquioxanes are out of scope of the Annex XV proposal and should be removed from the restriction dossier and supporting documentation. | Persistence, even extreme persistence, alone is not sufficient to assume an associated hazard without a full evidence based scientific assessment.  A risk assessment approach should be used to assess the potential risks associated with microplastics.  The proposed restriction appears to consider size as the most important factor for the behavior and risk associated with microplastics in the environment. This presumes that the hazards identified for a specific microplastic would necessarily be similar for all microplastics, and that other considerations, like chemical composition, are of lesser importance. To determine the hazards for specific microplastics a separate hazard evaluation would need to be conducted for those materials. The scope of the proposed restriction should be refined to clearly identify the chemical identities and the associated hazards of the specific microplastics included in the proposal to facilitate a proper risk evaluation for each microplastic that is impacted by the restriction proposal. | May 20, 2019  Mr. Bjorn Hansen  Executive Director  European Chemicals Agency  P.O. Box 400  00121 Helsinki  Finland  Re: Global Silicones Council (GSC) Comments on Proposed EU Microplastics Restriction  Dear Mr. Hansen:  The Global Silicones Council (GSC) is submitting the attached comments on the proposed EU microplastics restriction on intentionally added microplastics. The GSC also supports the comments on the proposed restriction submitted by the American Chemistry Council (ACC) and the European Chemical Industry Council (Cefic). We appreciate your consideration of these comments.  Please feel free to contact me directly at (703) 249-6197 or karluss_thomas@americanchemistry.com, if you have any questions.  Sincerely,    Karluss Thomas |
| **48** | I&P Europe - Imaging and Printing Association e.V. | **Answer to specific info request 5:**  Labelling requirement  • Preparation of label changes (e.g. adding additional text to existing labels) will cause efforts & costs for industry because not only the artwork of each label needs to be adjusted it could even mean that there might not be even enough space on the label available to add any additional information. In that case we have to use more expensive multi-page labels or make different versions of the products for different countries. Both options will lead to significant costs. Even a short text will require a lot of space if it has to be present in over 20 languages. Having in mind that products are sold in many countries in the EU with all the different languages coming into play it means that any additional text will also require corresponding proper translation. Additional translations are not available free of charge.  • Keeping in mind the typically products in their current design are sitting in many warehouses, not only across the EU but even outside EU, from which customer demands are served and any supply chain typically consists of many steps before reaching the final customer. Hence changing a label means for industry to pay a lot of attention and even more so creates huge efforts related to inventory management in order to ensure that only regulatory compliant products are shipped to customers.  • Adding more and more information to labels overall raises the question about the meaningfulness of labels and whether the customer could understand and digest the information or whether too much information creates information overflow and makes the label valueless.  Some suggestions  - Preferably: the information can be given in the SDS. As an alternative the relevant information can also be given in our industry in the safety instructions of the printer/printing machine instead of the label  - Any mandatory information on the label should be kept to a minimum (max one short sentence)  - No requirement to apply the information to all packaging layers (like it is in CLP)  Reporting obligation  • The proposed reporting requirement requires disclosing the identity of the polymer. The identity of substances is often confidential business information. Therefore the identity of the polymers is not always known.  Clarrification of the term “printing inks”  The term “printing inks” is used in the headline of Table 32 (Table 32: Summary of the socio-economic impacts of the proposed restriction on printing inks). However, according to our analysis and understanding the description and items in the table relate only to the product group toners. Therefore we suggest to replace the term “printing inks” with “toners” in order to avoid any possible misunderstanding. |  |  | **Answer to specific info request 3:**  Toners consist for 100% of microplastics. Some printing inks contain microplastics.  In general the microplastic concentration in printing inks is above 1% to be effective. We are not aware of any microplastic impurities, but we have also not tested specifically for this. In general the concentration limit of 0.01% is very hard to check as the concentration limit is very low.  • It is not entirely clear who has reporting obligations. Downstream users are mentioned. Are these only the downstream users placing microplastics on the market (as mentioned in the Annex XV report) or also end-users of microplastics (as suggested in the online information session on 3 April, for uses derogated under 4a)?  Why are only downstream users required to report and not importers?  If a microplastic is imported, then sold to another company who makes a mixture out of it and then to another one that uses the mixture to make an industrial printing ink and then the printing is used by an industrial user to print, which parties in this chain have a reporting obligation? How do you prevent double reporting of the same data? |  |
| **49** | IKW, Industrieverband Körperpflege- und Waschmittel e. V. | **Comment:**  Position of IKW  IKW, the German Cosmetic, Toiletry, Perfumery and Detergent Association (Industrieverband Körperpflege- und Waschmittel e. V.), is the industry and trade association of manufacturers and distributors of cosmetic, toiletry, perfumery, detergents and household cleaning products. The majority of the more than 430 members of IKW are small and medium-size manufacturers and distributors. More than 340 members of IKW manufacture cosmetic products.  IKW is supporting the contribution of the European association Cosmetics Europe and of VCI (Verband der chemischen Industrie e. V., German Chemical Industry Association) to the public consultation on microplastics.  The accumulation of plastic in aquatic environments is one of the major challenges today. However, a study by the Fraunhofer Institute for Environmental, Safety, and Energy Technology UMSICHT (“Kunststoffe in der Umwelt: Mikro- und Makroplastik”, J. Bertling, R. Bertling, L. Hamann, 2018) confirms again that microplastics in cosmetics play a minor role in environmental pollution caused by plastic debris. Nevertheless, cosmetics industry took action to minimize its contribution.  It is our understanding that the restriction dossier focusses on solid microplastic particles potentially released to the aquatic environment exclusively.  Microplastics in rinse off products  Within the framework of the “Kosmetikdialog”, a dialogue between the German Federal Ministry for the Environment, Nature Conservation and Nuclear Safety (BMU), the German Environment Agency (UBA) and the cosmetics industry in Germany represented by IKW, in 2013 a voluntary phase-out of microbeads in scrub and exfoliating cosmetic products was initiated. Based on the German initiative Cosmetics Europe recommended 2015 for its European membership a voluntary phase-out of microbeads in rinse off products for peeling and cleansing purposes until 2020. This voluntary action has been completed to more than 97 % until 2017. This clearly shows that a voluntary initiative can achieve quick and significant success. Our membership is prepared to further phase-out microplastics in all rinse off categories.  Apart from development activities, this requires comprehensive tests. This process is especially for SMEs challenging. According to the restriction dossier leave on products contribute 2 % to the overall microplastics emissions. However, more than 79 % of the estimated costs of the restriction would relate to leave on cosmetics. Thus, in our view the cosmetic manufacturers of leave on products are affected disproportionate. Therefore, we oppose a ban of microplastics in leave on products and recommend reconsidering the proposed regulation.  Labelling and reporting requirements  For some polymers labelling and reporting requirements are described in the restriction report. Especially reporting requirements seem not to be practicable for the cosmetics industry due to the great variety of different polymers used in the broad cosmetic product palette combined with low amounts applied and thus are opposed by our broad membership.  Conclusion  In summary, for our broad membership it is feasible to phase-out microplastic particles in rinse off cosmetic products, whereas for proportionality reasons we oppose a ban of microplastics in leave on products. Furthermore, we recommend reconsidering the proposed regulation concerning labelling and reporting requirements for specific polymers.  The cosmetics industry is highly interested in further improving the environmental sustainability of its products. We collect and process the corresponding information and are, therefore, in ongoing talks to the environmental protection authorities. |  | Microplastics in leave on products  Especially in leave on products a variety of microplastic particles regarding chemical composition, size, specific properties and functions is used – generally in low concentrations. However, the total tonnage of microplastic in leave on products compared to rinse off products is significant lower. Until now most cosmetic manufacturers have not intended to phase-out microplastic particles, because the scientific criticism focusses on microplastic use in rinse off products which are released completely into the domestic wastewater in contrast to leave on products. | The replacement of microplastic particles in leave on products would be a complex process. Alternative substances must first be tested regarding their safety, efficacy, environmental sustainability and product stability so that they meet the corresponding requirements. |  |
| **50** | International Fragrance Association | **Comment:**  IFRA Europe welcomes the chance to participate in this public consultation and to provide comments on the Annex XV proposal to restrict the use of intentionally added microplastics.  IFRA and its member companies are committed to ensuring the safe use of fragrance. We take seriously our responsibility to care for people and the environment, setting and sticking to high standards in our work.  Fragrance encapsulation chemistry provides benefits to the consumer, environment and to industry.  IFRA Europe has been proactive over the years prior to the proposed restriction and is deeply committed to ensuring the environmental safety of fragrance encapsulation. To show our cooperation, we have taken every measure to provide ECHA with the knowledge of our products. On several occasions, we have provided the state-of-the-art information on our products in scope of the restriction. This includes notably:  (1) Survey of Fragrance Encapsulation Systems, Survey results prepared jointly with IFRA Europe and RPA, 10 May 2018;  (2) IFRA Europe active participation to the stakeholders’ workshop on 30th – 31st May 2018;  (3) IFRA Europe data submission to ECHA, 1 October 2018, on behalf of three independent fragrance companies;  (4) Note on conference call with ECHA (ex-Kemi), 17 Dec 2018.  The fragrance industry is therefore surprised and disappointed by the wording used in the Annex XV proposal. As it currently stands, the proposal does not recognize the relatively small low impact of microplastics (and fragrance encapsulation in particular) on the environment resulting from plastics disposal.  IFRA Europe is keen to provide solutions. Our record of cooperation – such as our work with ECHA on REACH and registration of essential oils – demonstrates this point. In this spirit we remain strongly committed to continued work looking at the environmental safety of fragrance encapsulation and the development of viable alternatives to current fragrance encapsulations.  IFRA would like to take this opportunity to applaud ECHA for enabling derogation based on biodegradability. We will work to understand the biodegradability of our current technologies and, where appropriate, develop and assess alternatives. In evaluating the derogation, Industry experts realized that although the proposed derogation is flexible, it is also too stringent. However, they agree that it is manageable with subtle modifications. It will take significant time to adequately assess existing fragrance encapsulate systems, develop potential alternatives, and prove that the alternatives meet the biodegradation derogation – not least because the evaluation of microplastic and polymer biodegradation is an area of emerging science. To help guide ECHA to maintaining a proportionate and fit for purpose restriction, AISE and IFRA experts have drafted a cross-sectorial input based on the proposed Biodegradability Derogation 3 b). This input is provided as an attachment to Question 1 of this public consultation.  A restriction would have a huge economic impact and far-reaching implications for the whole European fragrance value chain, with the industry needing to make significant investments in the development of alternatives. These and other implications are set out in more detail in an independent report examining the potential socio-economic impact on the value chain of a restriction on the continued use of fragrance encapsulation, conducted by The Huggard Consulting Group (HCG) for IFRA Europe. The interim report is provided as an attachment to Question 4 of this public consultation. It indicates the importance of the fragrance and specifically of encapsulation to the European economy and to the wellbeing of its citizens. It also discusses the potential for unwanted outcomes from the proposed restriction such as a possible increase in re-washing and subsequent increase in use of resources and release of microfibres.  Considering the associated documents, and our commitment to work with ECHA, IFRA asks ECHA that transition periods for fragrance encapsulation are extended an additional 5-10 years and that the biodegradability criteria be modified per AISE and IFRA Europe recommendations. Longer transition periods will curb economic impacts and allow the industry time to adapt to this restriction while more flexible biodegradability criteria will make the development of alternatives possible. With these modifications to the restriction proposal, it will be possible to introduce a proportionate and fit for purpose restriction.  **Answer to specific info request 1:**  IFRA Europe and A.I.S.E. have jointly drafted comments and suggestions for consideration about biodegradability (criteria and testing methodologies). Please see document attached "microplastics AISE and IFRA Europe Comments Annex XV Biodegradation Derogation 5 16 19".  Response to Question 3d:  IFRA Europe is not aware of microplastics corresponding to the definition proposed in the restriction being present in a substance or a mixture as an impurity.  **Answer to specific info request 5:**  IFRA Europe has produced an interim socio-economic analysis. Please see document attached "190516 microplastics HCG SEA IFRA Encapsulation Interim". |  | **Answer to specific info request 3:**  Response to Question 3a:  In order to provide fragrance functionality to laundry and cleaning or cosmetic products, there requires intact polymer capsule presence at levels that contain fragrances to deliver long lasting consumer noticeable fragrance performance. At polymer levels below 0.01 percent consumer performance is lost rendering the finished product non-functional for fragrance delivery. Therefore, fragrance formulations only deliver functional benefit when capsule polymers are present above 0.01% in the finished product.  Response to Question 3b (based on RPA/IFRA Europe report):  With the exception of laundry care, the responses from IFRA members showed different use patterns of fragrance encapsulation systems across the different product categories. Overall, the use of fragrance encapsulation technologies represents a low proportion of the European market, as illustrated by the table below.  Percentage of the total EU market that contains fragrance encapsulates (Table 3.3. in the RPA/IFRA Europe report, see file attached).  With respect to personal care products, intentionally added polymers used for fragrance encapsulation account for only a small percentage of the total tonnages indicated as being used in rinse-off products (e.g. shampoos, shower gels) and leave-on products (e.g. deodorants).  To achieve their intended function, all these applications contain fragrance encapsulation above the 0.01% threshold established in the restriction proposal.  Response to Question 3c:  Regarding capsule polymer used for fragrance delivery in laundry and cleaning, and cosmetic products, there are no industry standard analytical methods that are available today. Nor are there any methods yet being developed, that enable measurement of polymer identity for purposes environmental monitoring, with sufficient low levels of detection, for the expected environmental release levels of polymer from fragrance in laundry and cleaning products. Additionally there are no industry standard methods available, or being developed for measuring levels of polymer in finished laundry and cleaning products or cosmetics, for purpose of compliance to the intended final ECHA restriction on microplastics. It is anticipated that development of such methods will take several years, to achieve an industry consistent standard, and may in the end not be achieved if a variety of different methods specific to proprietary polymer technologies are developed by different manufacturers". | There are currently no viable alternatives to fragrance encapsulation providing the performance attributes that are valued by manufacturers and consumers (stability in consumer product, delivery of a formulated mixture of fragrance oils, delivery delayed to time of requirement and adherence to the substrate – skin and cloth). This point has already been outlined in the IFRA Europe - RPA report of May 2018 and during follow-up discussions with ECHA. If the environmental data were set aside and the industry obliged to end encapsulation technology without an alternative being in place, the composition and dosage of the perfume would have to be completely reformulated, and consumers would be offered a lower level of performance. |  |
| 51 | IOGP |  |  | IOGP provides additional comments on 1) dissolved polymers and 2) labelling & reporting   |  |  |
| 52 | IPEC Europe | **(see attachment)** |  |  |  |  |
| 53 | Japan Business Machine and Information System Industries Association | a: Tonnages of microplastics used, releases to the environment, including pathways:  Toners for copiers and printers are supplied as the cartridges and the cartridges are used by being set in a machine, and a fixed image is formed inside the machine during printing process. So, it is not a particle and microplastics when the print is discharged from the machine.  Also, due to the system to retrieve used cartridges with toner left inside and the proper disposal are fixed, the possibility for used toner cartridges to be environmentally disposed are quite low. Therefore, the environmental emission will be negligible level.  In the table 32, it is stated that the estimated toner emission to the environment is average 3%. We believe that the amount of spilled toner is quite low and the frequent is also extremely low. Furthermore, when the toner spill from cartridge, we recommend to our users to wipe or vacuum the spilled toners and it is retrieved and disposed as municipal waste. Therefore, the releases of toners and microplastics to the environment are negligible and the number of average 3% of environment emission is not appropriate.  B: costs and benefits to producers, professionals, consumers:  Our toners are not classified as hazardous and generally we have limited chances to update the labels after product launch. If the labelling of toner products needs to be updated by the proposed requirements, the manufactures need the additional cost for changing.  There is no environmental emission of microplastics (=toners) under the normal use condition of machines and there is already a mechanism in which toner remaining in used cartridges is retrieved and properly processed so we strongly believe that there is extremely low possibility that users dispose used toner cartridges to the environment. Thus, there is no benefit of reduction of environmental emission by proposed labelling.  C: technical and economic feasibility of potential alternatives, including information on product performance, the price differences, the number of products, expected costs and timelines for reformulation and transitioning to a full-scale production: |  | We would like to provide information for toner:   1. Toner consists of 100% of microplastics. Setting concentration limit has no meaning for our products.     b. e) greater than 1.0% w/w.  Toner is a powder with a particle size of 4-10μm and it consist of colorants, matrix polymer, additives so it meets the proposed definition of microplastics. The polymers used in toner are vary and it depends on the manufacture, but there is no alternative since the toner itself meets the definition of microplastics.  Polymers in toner are used as a matrix to bind colorants and additives. In the fixing process after transferring process to paper, it is softened by heat and pressure, particles are fused, and it plays an essential function of the electrophotographic system of fixing on paper, so electrophotography cannot work out without a polymer.  If the toner diameter is more than 5 mm, it cannot work out as a product.  In addition, there is no alternative polymer substance which is out of scope of microplastics (i.e. natural polymer) and satisfy various criteria (i.e. moderate thermoplasticity) for electrophotography.  Although the use of biodegradable polymers deviating from the definition of microplastics is considered, biodegradable polymers known in the world are extremely limited. They do not satisfy the requirements (i.e. thermoplasticity, water resistance and light resistance) for electrophotography. Therefore, there is no guarantee that alternative materials will be found at this time.  D: hazard and risk of the alternatives:  Toner is uniquely formulated to function in laser printers and no other materials have the same properties. There is no alternative. |  |  |
| 54 | Japan Chemical Industry Association | **Comment:**  1) REACH Restriction is intended to regulate the manufacture, launch and use of substances at unacceptable risks to human health and environment throughout the EU when the measures are considered insufficient. However, this restriction proposal regulates only for the reason of form and persistency, and deviates from the REACH framework.  5) Although the reporting of the amount of use and release of microplastics are specified in Table 17 8, a threshold should be set for the amount reported based on rationality and workability.  6) Table 17 8 specifies the reporting of the names of polymers used in microplastics. However, considering CBI, the report should allow the use of generic names for polymers or polymer structures.  7) Table 21 describes the test methods for determining biodegradability. REACH Annex XI, Section 1.3 specifies that, as long as certain credibility is met, QSAR results may be used in lieu of testing. In accordance with the regulation, a possibility of evaluation by QSAR should not be excluded.  8) With many unclear points in the proposed regulations, the guidance should be issued. In particular, the following points require detailed explanation.  • Specific examples of microplastics subject to restriction  • Quantitative analysis method of size and shape on microplastics, Analysis method and estimation method for amount of microplastics released to the environment, and accuracy of quantitative values  • CBI Protection Guidelines  • Description on label and SDS | In addition, under the current situation with numerous uncertainties about the hazardousness, restricting its use by placing excessive weight on precautionary principles will inhibit the sound development of chemical industry and those industries using microplastics. | 3) According to the guidelines of ECHA for “Identification and naming of substances under REACH and CLP" (April 2017) and "How to identify a substance on the borderline of a mono- and multi-constituent" (May 2017), if the main constituent is not less than 80% (w/w) and a total of the remaining constituent substances (impurities, and intentionally added additives to maintain stability of the substance) is less than 20% (w/w), this substance shall be deemed as "mono-constituent", and the evaluation on physicochemical properties, hazard to human health, and environmental impact will be conducted on that single substance. Therefore, if a polymer is added for stabilization of a particle (e.g. inorganic oxides) consisting of a single substance and its concentration is less than 20% (w/w), this particle is judged as "mono-constituent", and thereby the evaluation on environment should be conducted on that single substance, excluded from the polymer-containing particle.  4) In Table 17 3.a., “polymers that occur in natural that have not been chemically modified” do not apply to microplastics. The reason is that these components are essentially biodegradable in the natural world. Therefore, even if chemically modified, when it is confirmed to be degraded at the level equivalent to or higher than that of natural polymers before modification, it is considered that such polymers can be excluded from the scope. Therefore, if degradability test results of chemically modified natural polymers are equivalent to those for unmodified natural polymers, chemically modified natural polymers should be considered as outside the scope. | 2) Microplastic is defined in Table 17 2 of the Restriction Report, but the grounds for upper and lower limits of the size are unclear. The definition should be based on internationally agreed concept, and setting non-measurable values in the definition would only be ineffective.  **Answer to specific info request 1:**  If test data such as ISO 14851, 14855-1 and 17556 are available, they should be considered in determining biodegradability. |  |
| 55 | JCIA (Japan Cosmetic Industry Association) | **Comment:**  On behalf of Japan Cosmetic Industry Association representing interest of more than one thousand Japanese cosmetic companies, I would like to express my sincere gratitude to the European Union’s great efforts to address the emerging issues such as microplastics to protect and preserve the global environment. For Japanese cosmetic industries also environment sustainability is one of the core important values in the world. However, JCIA considers that this proposed restriction for intentionally added microplastics submitted by ECHA in March 2019 is inappropriate from several perspectives such as proportionality, enforceability and technical feasibility. We concern serious negative consequences to international cosmetic industry caused by this proposed restriction. We are submitting the following comments on ECHA’s Annex XV restriction report.  Proportionality  JCIA and our member companies concern that ECHA’s proposed restriction on microplastics would result in serious disruption and economic harm to Japanese cosmetic manufacturers and their suppliers, and would also result in significant barriers to trade in cosmetic products exports from Japan to EU region, because current proposed restriction is much stricter and broader in scope than the internationally regulatory approaches including in Japan and other countries.  This ECHA’s proposed restriction bans polymers leave-on cosmetic products and furthermore include non-plastic polymers, as well as the expansive definition of solids defined as all substances other than liquid or gas. It would be significantly inconsistent with internationally recognized regulatory approaches that limit bans using in exfoliating or rinse-off cosmetic products. We believe that inclusion of this category of products in the proposed restriction is not only greater negative impact on our industry but also unjustified by a proportionate predicted benefit for the environment.  While our industry is currently evaluating the precise impact of the proposed restriction, the majority of Japanese products exported to the EU would be affected on our initial estimate. The costs related with reformulating tens of thousands of products would place considerable financial burden on manufacturers, cut off some resources from innovation and market opportunities elsewhere, despite of the very small contribution of microplastics used in cosmetic products to plastic litter in the environment. By ECHA’s own estimates, 79.3% of the costs of the overall restriction will be borne by leave-on cosmetics products, yet these products correspond to just 2% of the overall emissions from intentionally added microplastics.  In our view, the dossier makes several erroneous assumptions regarding the commercial impact of the proposed restriction on cosmetic manufacturers and seriously understates the expected negative commercial impact.  In the ECHA dossier on microplastics, not a conventional PEC/PNEC risk assessment approaches but ‘case-by-case’ approaches were adopted. However, whether their conclusion that a PEC/PNEC risk assessment approach cannot be applied for microplastics is still controversial from the view of multi-stakeholders. According to the article by Gouin T. et al. (Environ. Toxicol. Chem on June 24, 2019), the current state-of-the-science related to effects and exposure to microplastics implies that it is unlikely that the presence of microplastics in the environment currently represents a risk. Of course, the quality and quantity of existing data requires substantial improvement and further research and innovation addressing development of methodology is needed, but we consider that the proposed restriction on microplastics by ‘case-by-case’ approach for a non-threshold substance is premature. Therefore, we strongly suggest providing more reliable assessment data through discussion with multi-stakeholders before final conclusion.  In conclusion, JCIA strongly disagree with ECHA’s proposed restriction on microplastics. We believe the restriction, as presently envisioned, would have a disproportionate impact on our industry, and will serve as a significant barrier to trade. We urge ECHA to reconsider the proposed restriction especially revising the definitions to encompass only solid plastics, and to remove leave-on products from scope. In this way, ECHA’s proposed restriction would more effectively address those substances that are truly contributing to microplastic litter in the environment.  We highly appreciate the opportunity to present our opinions on this report and sincerely hope taking into the consideration those comments. |  |  | In the dossier, ECHA assumes there are natural alternatives for all the polymers that would be banned. However, ECHA does not provide suitability of the alternatives, industry cannot find substitutes for most ingredients would be banned by the proposed restriction. Loss of product performance, time to reformulate and SMEs which suffer severe impacts are not sufficiently taken into consideration in the dossier.  Regulation.  Furthermore, the proposed restriction also does not mention any analytical methods to characterize the restricted substances. Robust analytical methods should be prepared before the implementation of the restriction so that companies can demonstrate comply with this restriction.  Enforceability  The polymers/microplastics subject to Annex XV restriction are neither identified nor identifiable because there is no description how to judge it is acceptable or not in this proposed restriction. Therefore, the proposed restriction does not meet the procedures stipulated by Article 68 of REACH |  |
| 56 | KTF Organisation AB | **Content:**  Hazard or exposure;  Other socio economic analysis (SEA) issues |  |  |  |  |
| 57 | MedTech Europe | As described in MedTech Europe’s submission to the Call for Evidence (May 2018), microplastics are used in low quantities in a wide range of medical device and in vitro diagnostic (IVD) medical device applications. Microplastic particles are the basis of the technology used in essentially all automated IVD tests worldwide. If the proposed restriction were adopted, this would substantially impact the ability to provide essential diagnostic tests to professional laboratories that generate results for billions of patients in Europe and worldwide. The IVDs for clinical chemistry and laboratory medicine provide screening, diagnosis, prediction and monitoring of medical conditions including critical care infectious, rare or genetic diseases. IVDs ensure the safety of the blood supply in most countries around the world, including Europe.  MedTech Europe requests that the use of microplastics in medical devices, in vitro diagnostic medical devices and in similar products (lab only, Research Use Only, Quality Control) is exempted from the scope of the restriction. As described in our reply to Question 4, it is not feasible to implement technical means for the containment of microplastics and/or to guarantee disposal of microplastics-containing waste as hazardous waste for a number of applications (e.g. IER used in medical applications), and the required technical measures are expected to pose significant financial and logistical challenges for many other medical technology applications (notably microplastics used in IVD reagents).  **Answer to specific info request 4:**  Please note: the derogation referred to is Paragraph 5a, not Paragraph 5b.  MedTech Europe is the European trade association for the medical technology industry including diagnostics, medical devices and digital health. Our members are national, European and multinational companies as well as a network of national medical technology associations who research, develop, manufacture, distribute and supply health-related technologies, services and solutions.  MedTech Europe requests that the use of microplastics in medical devices, in vitro diagnostic (IVD) medical devices and similar products (lab only, Research Use Only, Quality Control) is exempted from the restriction. For applications such as ion exchange resins used in medical applications, containment of microplastics (required to comply with derogation 5a) is not feasible from a technical point of view. For other applications, such as microplastics used in IVD reagents, it may require re-design of products and would pose serious logistical and financial challenges for hospitals and labs across Europe. Moreover, due to the nature of IVD applications (reagent-instrument combination), the impact of the restriction would go beyond reagents using microplastics and could affect any IVD instrument that is using both microplastic- and non-microplastic-containing reagents. Therefore, we consider that the anticipated cost of the proposed restriction is disproportionate to the limited environmental benefit that it is expected to achieve.  To comply with the proposed restriction, medical devices and IVDs would require substantially more than 2 years (paragraph 6b) to develop, validate and implement containment measures for microplastics. Depending on the application, this process may take 5 to 12 years to complete. The proposed restriction does not consider the long design cycles for medical technology products and hence holds a risk that critical healthcare products will not be available to patients in Europe (and worldwide, as also global change notifications may be required). By contrast, exempting medical devices and IVDs from the scope of the restriction would only have a minimal impact on ECHA’s overall aim of reducing the release of microplastics to the environment.  Medical devices:  Ion exchange resins (IER) used for medical applications: The Annex to the restriction report acknowledges that systems exist which are not fully closed (page 213: “Suppliers of these resins have indicated that in some cases, the microplastics can be supplied in bulk to the customers for them to load in their own manufacturing facilities”). However, these ion exchange resins are also used by professionals, for example bulk handling/refilling of IER for medical water treatment in hospitals. These uses cannot be covered by derogation 5a and would be banned by the restriction. Where closed systems exist, derogation 5a would require changing the waste classification to ‘hazardous’ even for applications that do not become biohazardous. It is questionable if manufacturers of these products will be able to instruct their customers to incinerate where there is no legal basis in EU or local waste regulations to incinerate waste or dispose of it as hazardous. Also from an environmental perspective, requiring disposal of these uncontaminated materials would be sub-optimal to recycling them.  In vitro diagnostic (IVD) medical devices and similar lab only/RUO applications:  The IVD industry is predicated on the use of polymer microspheres as the basis for the automated/high volume testing platforms used by labs across Europe. Microplastic particles are an integral part of the function of IVD reagents and are the basis for the function of the reagent in conjunction with the IVD instrument. Depending on the application, containment of microplastics may require changes at the level of the reagent composition, the level of the IVD instrument, or (most likely) their combination. In each case, a significant number of years would be needed to implement the technical measures requested by ECHA in the restriction proposal. For IVDs, the proposed technical measures are not a ‘simple’ containment measure but likely to require extensive development, testing, re-validation of the reagent and/or instrument as well as regulatory approval of the design changes by Notified Bodies under the IVD Regulation. This is a process that could take up to 5-12 years (see Figure 7 in the Annex to the restriction report) for each product, similar to when microplastics would need to be substituted. As a large number of different reagents are usually run on the same IVD instrument, any instrument that uses only one reagent containing microplastics would need to undergo design changes, virtually affecting all IVD instruments available on the market. In addition to the design cycle, hardware updates, installation and service of existing instrument placements are expected to add to the time that will be needed to make these changes.  For customers, in labs where microplastics are currently part of the liquid waste (non-hazardous), this will require segregation of the waste water to route it separately from the public sewer system. This would pose significant financial and logistical issues for a large proportion of healthcare institutions in Europe, as they would need to retrofit their existing infrastructure to collect microplastics-containing waste separately and dispose of it as hazardous waste. It would also be more demanding on healthcare professionals (e.g. manual handling of liquid waste).  On the basis of the current criteria for the classification of waste, the microplastics would not trigger a classification of waste as hazardous, and there would be no legal requirement to incinerate that waste fraction. Costs of incinerating waste water classified as hazardous will typically be very high on a per tonne basis. Moreover, obtaining access to a permitted incinerator plant is likely to pose a significant challenge to some healthcare facilities, which would make it impossible for them to meet the conditions of the restriction.  Similar uses that would also be permitted on the basis of this proposed derogation:  If medical devices and IVDs (and similar lab only/RUO applications) are not exempted from the restriction, we strongly suggest that ECHA also cover the use of microplastics in lab only and Research Use Only (RUO) products by the derogation under Paragraph 5a. The way the restriction proposal is currently written would still pose a huge problem for these applications. Since lab only/RUO products (non CE-marked) are not covered by Regulation (EU) 2017/746, they would face an immediate ban at the time of entry into force of the restriction (unless they already fulfil the conditions of derogation 5a or of ‘Scientific Research & Development’ (SR&D) per REACH Art. 56(3)). As the technical function of microplastics in lab only and RUO products does not differ from their use in IVDs, there is no reason to treat them differently for the purpose of this restriction.  Similarly, we believe that the derogation should also cover the use of microplastics in Quality Control (e.g. reagents for calibration of IVD instruments). (Imported) Calibration reagents containing microplastics could cease to be available in the European Economic Area (EEA) if they cannot benefit from the SR&D exemption (relatively small business), which could have important consequences for the IVD industry and patients in the EEA as without these reagents, certain IVD instruments could not be kept operational.  All the above applications would require the same amount of time to comply with the restriction proposal as medical devices and IVDs covered by Regulations (EU) 2017/745 (MDR: Medical Devices Regulation) and 2017/746 (IVDR: In Vitro Diagnostic Medical Devices Regulation), respectively.  Additionally, the restriction proposal does not consider that up until May 2024 (due to the so-called ‘grace period’), some medical devices and IVDs will still be placed on the market under the current medical device directives (Council Directive 93/42/EEC on medical devices, Council Directive 90/385/EEC on active implantable medical devices, Directive 98/79/EC on in vitro diagnostic medical devices).These products are currently not covered by Paragraph 6b and would face an immediate ban at the time the restriction enters into force.  Labelling:  If medical devices and IVDs (and similar lab only/RUO applications) are not exempted from the restriction, MedTech Europe believes that the best means to communicate safe use information on microplastics are Safety Data Sheets provided in accordance with the REACH Regulation. This regulation has been developed to address chemical concerns and is better equipped to do so than the medical device regulations, which have their own requirements for labels and Instructions for Use. As medical device and IVD labels are already strictly regulated (as are the Instructions for Use), changing them may have regulatory consequences, is costly and will require substantially more time than updating Safety Data Sheets, which are commonly used by the IVD industry for professional use IVDs. Since the IVD sector does not have consumer uses of microplastics and the potential release is limited to the waste phase, we consider the disposal section (Section 13: Disposal considerations) of a Safety Data Sheet the most appropriate way of providing instructions to users of IVD products on how to collect and dispose of microplastics-containing waste.  Also for medical devices (non-IVD) which are mixtures containing microplastics and are used by healthcare professionals, the restriction should give manufacturers flexibility in how they communicate safe use instructions to users, giving them the opportunity to avoid the strictly regulated means of communication such as MDR-labels or Instructions for Use.  Any obligation to communicate information on microplastics on medical device or IVD labels or in the Instructions for Use would have important consequences under the MD/IVD regulations, as changes to a label or IFU may be subject to design change processes, which are strictly regulated. Depending on the change, this might require involvement of the Notified Body. Label and/or IFU changes may also be subject to approval or notification obligations in non-EU markets.  Moreover, any labelling obligation regarding microplastics will require additional resources from manufacturers and will need sufficient time for implementation. The labels and Instructions for Use (IFU) in accordance with Regulation (EU) 2017/745 have to be updated and in place by May 2020. For in vitro diagnostic medical devices covered by Regulation (EU) 2017/746, they have to be updated and in place by May 2022. Medical device and IVD manufacturers are hence already in the process of updating their labels and IFUs. Therefore, IFU and label updates for microplastics would have to be addressed in the next round of label/IFU updates (hence not associated with the current MDR/IVDR updates). It is a duplication of requirements, competing for the same resources and cannot be considered as part of an activity that would need to happen anyway, as it seems to be presented in the restriction report (in which the cost for labelling is stated as ‘negligible’).  If ECHA were to impose including information on microplastics on medical device and IVD labels or in the Instructions for Use, the proposed 18 months for compliance are largely insufficient and would need to be extended to at least 3 years after the first label/IFU updates under the MDR and the IVDR have been completed.  **Answer to specific info request 5:**  c.  Medical devices cover a very broad field of products, e.g. dental base material, substance-based medical devices, wheelchair, pacemaker.  For some products, e.g. some substance-based medical devices, there are no alternatives for synthetic polymers used. It is questionable whether it is possible to formulate those medical devices without solid synthetic polymers.  For products with potential alternatives it would require a lot of time, effort and cost for the manufacturer who need to reformulate all affected medical devices. Reformulating a product takes at least between 21 months and 3 years, and to this time 18 months should be added for regulatory approval of the product. We estimate the cost at approximately 1.1 mio Euro per formulation, which include 0.6 mio Euro technical costs and at least 0.5 mio Euro clinical costs. It is also important to note that across the EU, a large number of products, mainly from small and medium-sized enterprises, are affected.  Then, there is a second difficulty: the alternative needs to have comparable efficacity and safety profile. A one-to-one substitution of an ingredient is not possible because of the interaction with the other ingredients. This interaction needs to be considered by formulating the product and an alternative is not always feasible. The reformulation may have an impact on the quality, safety and efficacy of the device.  The reformulation obligation for all affected product could lead to disadvantages for manufacturers inside the EU in comparison to non-EU manufacturers. |  | The cost of complying with technical containment of the microplastics and disposal as hazardous waste is disproportionate to the expected benefit of the restriction and would fall not only on MD/IVD manufacturers but also on healthcare facilities across Europe. ECHA identified that the total use of microplastics by the medical device and IVD sector represents 0.1% of all industrial and professional uses. The restriction report also describes that the risk reduction capacity through containment measures for our sector represents 0.27% of the microplastics used (avoided release of microplastics of 270 kg/year). Including medical devices and IVDs in the restriction proposal would therefore only marginally contribute to the overall effort to reduce microplastics in the environment, whereas the socio-economic impact of including these uses is substantial.  A detailed socio-economic assessment (SEA) of the restriction proposal for the medical device and IVD sector is added to this statement as a confidential attachment. The main focus of the SEA is on IVD and similar applications due to the high number of affected products and users. It is clear from this assessment that the sector is hugely and disproportionately affected by this restriction proposal. The overall result (low cost effectiveness) is considered to be representative for the whole MD/IVD sector. Importantly, the SEA’s focus on in vitro diagnostics should not give rise to challenge the socio-economic relevance and unquestionable benefits of other medical applications that may contain microplastics such as life-saving adsorbers and ion exchange resins in blood treatment for critical and chronic care. In conclusion, MedTech Europe strongly supports a complete exemption of medical technology products (medical devices, IVDs and similar products) from the proposed restriction.  **Answer to specific info request 1:**  Beside the tiered approach mentioned above, all other data and approaches, that show that the polymer is not persistent should be used (e.g. for medical devices ISO 10993-13:2010, Biological evaluation of medical devices – Part 13: Identification and quantification of degradation products from polymeric medical devices, in combination with ISO 10993-9: 2009, Biological evaluation of medical devices -- Part 9: Framework for identification and quantification of potential degradation products and their corresponding ISO EN standards).  **Answer to specific info request 3:**  b.  The restriction proposal has an enormous impact on a large amount of substance-based medical devices using substances that contain solid synthetic polymers (e.g. modified cellulose, carbomer or sodium carbomer, acrylates crosscopolymer). One excipient in a medical device can have various functions (e.g. film forming, primary thickening, mechanical or rheological properties) depending on when and for what purpose used in the manufacturing process. Therefore, the concentration of synthetic polymers used is > 0,1% (w/w), in most cases < 1%. Such substances are essential in the formulation of the substance-based medical devices.  c.  At the moment, we are not aware of any analytical methods for detection and quantification in the medical devices.  Moreover, the concentration of microplastics in waste water from IVD instruments is very low and therefore the volume of waste water will typically be high in proportion to the volume of microplastics. Incineration of high volumes of waste water with low concentrations of microplastics is not desirable from an environmental point of view and would present a substantial additional cost and logistical burden for labs across Europe. The current legal framework in the EU for managing waste water from IVD instruments accepts that waste water with low concentration of microplastics in the effluent is treated as ‘household’ waste water. |  |  |
| 58 | Mondi (industry) |  |  | Graeme Smith, innovation and sustainability manager at Mondi: “*Whatever drop in polymer demand and packaging we see, will just be balanced out by other demands and other polymer markets”* and due to the barriers of recycling them, “*PVC and PVCD coatings, for example, are a thing of the past*” (CW, 2019s). |  |  |
| 59 | Perfumery Association |  |  |  |  |  |
| 60 | Personal Care Products Council | **Content:**  Scope or restriction option analysis;  Hazard or exposure;  Environmental emissions;  Description of analytical methods;  Information on alternatives;  Information on costs;  Information on benefits;  Other socio economic analysis (SEA) issues;  Transitional peri |  |  |  |  |
| 61 | Plantum | Please find our comments in the attachment in Section IV. Plantum is a member of ESA (European Seed Association): we worked with ESA on these comments and fully support them. |  |  |  |  |
| 62 | PlasticsEurope | **Comment:**  PlasticsEurope is the leading pan-European association representing a network of plastics manufacturers. We represent more than 100 member companies, producing over 90% of all polymers across the EU28 member states plus Norway, Switzerland and Turkey. The European plastics industry already makes a significant contribution to the welfare in Europe by enabling innovation, creating quality of life to citizens and facilitating resource efficiency and climate protection. Whatever its origin, plastic waste in any environment is unacceptable, and the plastic industry is fully committed to find solutions to plastic pollution and continuing global collaborative partnerships to tackle the problem at source. For more information, visit the Marine Litter Solutions, the Operation Clean Sweep® and the Alliance to End Plastic Waste websites. PlasticsEurope is also a member of the CEFIC-led Microplastics platform.  We agree that the REACH regulation is an appropriate regulatory tool to address risk related to chemicals EU-wide. However, we believe that the current Annex XV proposal for a restriction on intentionally added microplastics does not meet the REACH regulation requirements, and as a result would not meet the intended objective. Therefore, it cannot be supported by industry for the following reasons:  • By assessing a group of substances identified generically, the Annex XV restriction proposal goes against the provisions of the REACH Regulation;  • This general assessment makes it extremely difficult to understand, interpret and enforce the restriction, and put large administrative burden on the industry;  Plastics raw materials containment is at the core of the plastics industry commitment towards zero pellet loss in the environment. For this reason, PlasticsEurope joined the Operation Clean Sweep® (OCS) programme in 2015 to align and concentrate all industry efforts globally under a common approach to prevent pellet loss.  In order to further accelerate and strengthen the OCS programme, PlasticsEurope has committed to the development of an OCS certification scheme with third party auditing . The OCS certification scheme enables the plastics industry and all value chain handlers of pellets to transparently and jointly demonstrate its efforts towards zero pellet loss into the environment. PlasticsEurope believes that this approach is the most effective and efficient approach to achieve zero plastic pellets loss. This is also fully in line with future measures and actions to curb microplastics pollution proposed in the EU Plastic Strategy which was published in 2018 − development of measures to reduce plastic pellet spillage (e.g. certification scheme along the plastic supply chain and/or Best Available Techniques reference document under the Industrial Emissions Directive. The OCS certification scheme sets common requirements (based on the six pillars of the OCS pledge) that will be audited regularly by accredited third parties. For producers of plastics raw materials and, if relevant, other parts of the value chain, these requirements will be introduced in existing environmental or quality management systems. PlasticsEurope is currently consulting the plastics value chain on the development of the OCS certification scheme.  OCS is also one of the top priorities in PlasticsEurope 2030 Voluntary Commitment which was published in 2018. This places PlasticsEurope in a strong leadership position to continuously improve the programme, extend its implementation to the whole plastic value chain and increase transparency in its efforts to externally communicate efforts of its members in tackling pellet loss.  In practice, PlasticsEurope OCS commitments include the following:  • End of 2019, to develop a common self-assessment tool to help members improve in implementing pellet containment best-practices,  • By 2030, to engage with all major European ports. Three major EU ports have already signed,  • End of 2018, to develop a transparent, harmonised monitoring scheme for the collection of relevant and comparable information from all signed members to measure progress.  PlasticsEurope also publishes annually an OCS report which provides an overview of OCS implementation in Europe by PlasticsEurope member companies.  With the significant progress that has been achieved in strengthening and accelerating the uptake of OCS across the value chain above in mind, and because pellets are industrially transformed into plastic items and do not retain their shape and size in the final product, PlasticsEurope strongly advocates that plastics raw materials such as pellets should not be regulated under the proposed REACH restriction for intentionally added microplastics. We are therefore calling for a full exemption i.e. exclusion, from the current Annex XV proposal for a restriction on intentionally added microplastics for plastics raw materials since these will be covered more adequately by the OCS certification scheme which is currently under development. | The scientific evidence provided to substantiate the proposed restrictions does not meet the standards required on the application of the precautionary principles. | As indicated in ECHA’s restriction report, microplastics are of concern because of their potential environmental and health risks posed by their presence in the environment. Therefore, microplastics where the physical properties are permanently modified during the use or manufacturing process should not be covered under any REACH restriction, as these uses will not pose a risk to the environment or human health. In Europe, approximately 80 percent of plastic raw materials are in the form of round to oval granules of approximately 2-5 mm in diameter, called pellets. During their conversion into final products, plastics pellets are transformed by e.g. molding, melting etc. which modifies their physical properties and they do not retain their size and shape. As plastics pellets will no longer be present after their conversion into final articles they are not intentionally released during or after product use.  We believe that the scope of the proposed definition of microplastics is ambiguous and much broader than the current common understanding of the term ’microplastic’. In practice, applying the proposed definition would mean that almost any particle would be considered a microplastic and subject to either a ban or reporting and labelling. For example, any product commercialised as powder, if treated with a polymeric substance will be falling under the microplastic definition. This would result in reporting throughout the supply chain leading to volumes being reported multiple times.  Finally, in its proposed definition of ‘polymer-containing particle’ ECHA includes particles that contain >1% w/w polymer. This would potentially include some powders in which dustiness is reduced by using small amount of polymers to minimize environmental losses, prevent potential inhalation issues and explosion risk. Such mixtures have not been adequately addressed during the call for evidence since not foreseen as being microplastics. Such a low % may lead to riskier situations where to avoid being under the scope of the restriction some operators may be tempted to reduce the concentration of these compacted agents below the 1% w/w limit. In order to prevent such situations, we propose to increase the limit of the % w/w polymer. The exact limit should be based on an assessment of the critical concentration to avoid regrettable technical evolution only driven by the scope of the restriction. | The restriction does not provide the defining element of risk – an identified hazard – and therefore bases the provision on risk posed by the “extreme persistency of polymers”;  • Since a hazard or a risk posed by microplastics has not been identified in accordance with the rules of the REACH Regulation, the proposed measures cannot be considered appropriate and proportional to an objective that is legitimate under the REACH Regulation;  **Answer to specific info request 1:**  PlasticsEurope is currently assessing the cost impact of the proposed ECHA restriction on intentionally added microplastics on its membership. |  |
| 63 | Polish Association of Cosmetic and Detergent Industry | **Content:**  Scope or restriction option analysis;  Other socio economic analysis (SEA) issues;  Transitional period |  |  |  |  |
| 64 | SOIA – Cefic Sector Group | Soia (the Synthetic Organic Ion Exchangers and Adsorbents Group), a Sector Group of Cefic, the European Chemical Industry Council, fully recognise the current concerns over microplastics and release of this material type in the environment. The restriction proposal as it is published by ECHA will have significant impact on the manufacturers, importers and downstream users of Ion Exchange Resins (hereafter called IER) and subsequently consumers.  The scope of the definition is much broader than the definition of microplastics (although all plastics are polymers, not all polymers are microplastics) and it would lead to the entire IER portfolios of the EU manufacturers to be considered as microplastics (e.g. drinking water softeners, cartridges for dishwasher, cars, coffee machines, water dispenser etc). In all these applications there are no intentional release to the environment.  In our opinion the restriction proposal should have a more focused scope, a more precise microplastic definition and target specific uses identified as high risk. Please see the whole argumentation in attachment for further explanations regarding our position. |  |  |  |  |
| 65 | Sport and Play Construction Association | **Content:**  Scope or restriction option analysis |  |  |  |  |
| 66 | STANPA, Spanish Cosmetic, Toiletry and Perfumery Association | **Content:**  Scope or restriction option analysis;  Information on alternatives;  Information on costs;  Transitional period;  Request for exemption |  |  |  |  |
| 67 | SVEFF – the Swedish Paint and Adhesives Manufacturers | We would like to take the opportunity, in this first phase of the consultation, to raise some points regarding the reporting requirement in paragraph 8 of the proposal.  SUMMARY  The reporting requirement in paragraph 8 has not been sufficiently described or analysed in the restriction proposal. There are several aspects that determine the cost and benefits of the scheme that stands yet to be defined.  1. A very large number of companies are covered by the reporting requirement as it includes users at industrial sites. An assessment on the number of companies covered in the paint and adhesives sector in Sweden alone is around 11 000 (formulators and users) – most of which are SMEs.  2. These companies are expected to report information that is not available to them. Most polymers are not classified as hazardous and thus information on polymer identity is not forwarded in the supply chain. The level of detail of “polymer identity” stands yet to be defined by the dossier holder.  3. The complexity of the database is underestimated and thus the cost for ECHA and the industry is underestimated. We believe the cost do not stand in proportion to the expected benefits.  4. For ECHA to reliably be able to track unintended release of microplastics using the envisaged database, the input needs to be of good quality. Given the unresolved issue of how to identity and/or group polymers in microplastics and the number of actors involved we question the usefulness of the database.  Detailed arguments and references are included in the attachment |  |  |  |  |
| 68 | Titanium Dioxide Manufacturers Association (TDMA) | This definition goes well beyond what would commonly be considered a plastic. TDMA believe a definition listing the key plastics found in the environment could be the most effective approach.  TDMA understand that information is important in understanding and managing problems but believes the reporting requirements proposed in the restriction would create bureaucracy without improving the environment. The difficulties of double counting, unclear definition and measurement will probably result in meaningless data which will bring more confusion than clarity. If this is repeated each year, the data will probably be ignored and will result in reporting for reporting sake, distracting resources from preventing plastics entering the environment.  TDMA supports efforts to reduce plastics entering the environment but believe this restriction could be counterproductive by focusing the resources and attention from the key materials concerned. This definition would require endless discussion and guidance of whether to be included as well as technical issues such as measurement methods. The most effective regulations are normally clear and simple. We hope you will take our comments into consideration and remain available for any questions or clarification.  EU Transparency Register n° 64879142323-90  TDMA is a sector group of Cefic |  | The Titanium Dioxide Manufacturers Association (TDMA) is a sector group of Cefic, which represents the leading producers and suppliers of titanium dioxide in Europe.  Titanium dioxide is implicated in the microplastics restriction proposal as some specific grades of titanium dioxide are organically coated and could be included in the current definition. They are particles between 1nm and 5mm with a polymer surface coating and the polymer content may exceed 1% w/w.  TDMA supports the points made in the Cefic submission including the definition not being substance specific as required by REACH, the overly broad definition, the lack of identified hazard, the unreasonable requirements for reporting outside the scope of the restriction and the overall disproportionate nature of the proposal. |  |  |
| 69 | The Life Sciences Manufacturers Alliance (an ad hoc group composed by GE Healthcare Life Sciences, Merck KGaA and Thermo Fisher Scientific) | See attachment. |  |  |  |  |
| 70 | The Oxo-biodegradable Plastic Association | Michael Stephen, Chairman of the Oxo-biodegradable Plastic Association “The European Commissioners are either being very dim or don’t want to listen,” Stephens insisted, adding that Timmermans’ assertion that oxo-plastics break down into microplastics is “simply not true”” (Morgan, 2018) |  |  |  |  |
| 71 | The Plant Care Industries Association (IVA) | **Comment:**  Following the Annex XV restriction report submitted by ECHA on 11 January 2019, related to a possible restriction of intentionally added microplastics, The Plant Care Industries Association (IVA) takes note of the proposal for a restriction on microplastics, including those used in the agricultural sector. IVA welcomes the alignment of the current restriction proposal with the regulatory requirements of the new Fertilizers Regulation regarding the biodegradability of polymers used in CE-marked and non-CE marked fertilizers. IVA, as the german national association for the fertilizers industry adopts the answers of the European Fertilizers association, Fertilizers Europe, as it stands.  **Answer to specific info request 3:**  a. a. Fertilizers Europe collected information from its members via 2 Questionnaires that have been already shared with ECHA on 30th November 2018. The Questionnaires contain information respectively for technical additives (anti-caking/anti-dusting agents) and for polymers used in controlled release fertilizers (CRF). Information about the concentration of polymer-containing particles (expressed in w/w) in the final products is reported for each fertilizer product used. The Questionnaires are attached as a “Confidential Attachment”.  **Answer to specific info request 5:**  The answers to Question No. 5 (the tonnages microplastics used, their technical functions and releases to the environment) are submitted as a "Confidential Attachment" |  | **Answer to specific info request 1:**  The biodegradation of natural materials and polymers is generally slow. Good information can be found in method ISO 17556:2012 that is mentioned as a permitted test method in Table 21 of the restriction. Especially Annex F and G are informative since they describe biodegradation experiments in soil of naturally occurring materials and synthetic polymers.  • In Annex F the biodegradation of birch leaves, oak leaves and pine needles is described. In Table F1 the biodegradation after 1 year is mentioned. The leaves and pine needles are degraded for about 56-62%. If one would extrapolate the curves in the figure F.1, below the table F.1, it can be deduced that the 24 month/90% (conversion compared to a reference) criterion will not be met. Even the extended biodegradation criterion of 48 months/90% (conversion to a reference) is possibly challenging to meet. Since the materials in Annex F are naturally occurring there is no environmental concern and slow biodegradation is well accepted.  • In Annex G a round-robin testing (ring testing) was performed. The data are presented in the graph below. Clearly, there is a large spread in the data and in some labs, the tested material starch/poly(butylene adipate-co-butylene terephthalate) blend could be considered biodegradable, whereas in most laboratories the material failed to biodegrade sufficiently (Only for 7 out of 17 test a plateau level in CO2 evolved was reached indicating that the biodegradation was complete in the tested period for both the reference (microcrystalline cellulose) and the starch/poly(butylene adipate-co-butylene terephthalate) blend . From these 4 test show incomplete biodegradation 20-58% biodegradation and 3 test complete biodegradation (97-107%).). Given the inconsistent results, it is obvious that a better test is required.  The EU commission (see Article 42 of the new Fertilizer Regulation) will set biodegradation criteria within the next 5 years in a delegated act. This means that a biodegradation period of 48 months plus release period will not ‘fit’ in the 5 years allowed. Therefore, a project was started by the industry with two expert partners (OWS in Belgium and Tomas Bata University in Czech Republic) to develop an accelerated biodegradation test. This test is done in soil at 37 oC in soil. Biodegradation tests at lower temperatures (25 oC) are as well done for comparison. The target of this study is to show that an accelerated biodegradation study is possible for CRF coatings and that test criteria can be set for biodegradation. The partners expect publication of the paper in the third quarter of this year.  The period of 5 years allowed under the new Fertilizers Regulation for development of the biodegradation criteria plus 2 years for implementation is very challenging given the technical challenges. Industry needs to develop new biodegradable coating technologies, ensure that they can be applied both safely and economically on fertilizer granules, test the performance of these new CRF in lab and under practical conditions for multiple seasons and build the new coating installations to produce these new CRF.  b. b. The current proposal for restriction sets a limit of 0.01% and by the given definition of microplastic, the cut-off applies to mixtures containing solid polymers in a concentration equal or above 1%. This means that a product containing 1 ppm or more of solid polymer in a size between 1 nm and 5 mm falls in the scope of the restriction. Question 3 aims to set even lower boundaries. Assuming that a method to detect 1 ppm (or even ten or hundred time less) of polymer in products (and to determine whether this amount is in a solid form) is technically feasible, we believe that at this level an unintentional release from packaging material to products cannot be excluded, for instance by abrasion during product handling. In this perspective, Fertilizers Europe believes that the proposed combined limit and the intention to eventually even tighten the proposal will not be enforceable in practice.  c. c. At the moment there is no accepted and validated qualitative or quantitative method to test for polymers in fertilizers.  d. Since the fertilizer industry is mainly a downstream user of formulated products for a diverse range of technical applications, they are not in the position to comment whether products that they use might contain polymers or microplastics as ingredients. |  |  |
| 72 | The Polish Union of Cosmetics Industry | **Comment:**  We would like to express our concern regarding the scope of the restriction proposed by ECHA. In our opinion it is too broad taking into consideration the initial purpose of the restriction proposal (i.e. plastic litter in the marine environment) and it will impact on key product categories most specifically leave-on cosmetic products. As the ECHA proposal dossier itself states leave-on cosmetics are estimated to be 2% of the overall emissions of intentionally added microplastics to products, it also indicates that 79.3% of the costs of the overall restriction (i.e. the costs of the restriction for all implicated sectors) will be borne by leave-on cosmetic products alone. It is clear that our sector is totally disproportionately affected and it will directly impact cosmetic companies in a way that we feel is unreasonable and unequal. There is no scientific data suggesting that non-solid plastics (polymers) in leave on cosmetics may negatively impact environment. ECHA proposal should be considered as contradictory to the Better Regulation Principles which are one of the key policies of the European Commission. Therefore, we are of the opinion that leave-on cosmetic products should be exempted from the restriction proposal.  In our assessment, definition proposed by ECHA is too broad going beyond plastic litter in the marine environment. It includes many non-plastic polymers which are not part of the plastic litter debate. It is unacceptable that non-plastic substances used in cosmetic products may fall in scope as ECHA does not refer to ‘plastic’ in its definitions. It should be emphasized that all plastics are polymers but not all polymers are plastics.  In our view ECHA should narrow the definition of microplastics to provide that the restriction applies to only those substances which are plastics. Materials that are not solid in the finished product as well as elastomeric materials should not fall into the scope of the restriction - the same as liquid polymers and film formers which were taken into account and are out of scope for the present proposed restriction.  We believe that the microplastic definition should address, among others, the plastics, biodegradability, solubility and microplastic particles.  We definitely do not agree with evaluation of alternatives availability presented by ECHA. ECHA makes false assumption that there are alternative ingredients to all microplastics immediately available in the market. This is a major shortcoming for what concerns the assumption on the reformulation cost in the proposal. The ECHA proposal disregards evidence submitted by Cosmetics Europe during the call for evidence on the availability of alternatives.  Based on our experience one to one substitution of ingredients is not always feasible. Suitability of alternatives depends on many factors like the interaction with the other ingredients in the formulation, cost and performance. Fundamental research and investments would be needed to find, deviating cosmetic business from its normal course, which could result in losing competitiveness or even withdrawing entire products lines. What is more, cosmetic companies are completely dependent on the capacity of their suppliers to propose new raw materials, which was not addressed in the ECHA dossier. We are of the position that the availability of alternatives should be re-evaluated by ECHA as it is a key factor as to whether a product can be reformulated.  In our view, the proposed transition periods for cosmetic industry are definitely too short to complete all necessary reformulations. The average reformulation process takes around 4.5-5 years – taking into consideration that some crucial properties of products will be affected (e.g. physico-chemical form, viscosity, thixotropic properties – which are not replaceable in one-to-one exchange of the ingredient). However, if there is no suitable alternative from suppliers, fundamental research is needed first to develop new raw materials so the process could be extended to 8-10 years.  What also concerns us is that cosmetic companies would be forced to reformulate many products at the same time - containing not only one substance, but potentially few polymers (substances) at the time. This would be a tremendous scale of reformulation of large part of the portfolio of each cosmetic products producer. Moreover, ECHA has not acknowledged the technical time related to the shelf-life test which requires between 30 and 36 months to be added to the 4.5-5 years for the baseline reformulation. Consequently, we postulate to double the transitional periods proposed in the dossier.  In our opinion, the proposal by ECHA makes wrong (false) assumptions regarding SMEs, for example that the impact of the restriction might not be negative for them. Proposal states that SMEs “tend to specialise in niche organic and natural products”. We can confirm that this is not the case. Cosmetics Europe current estimate is only around 7% of SMEs in its membership focus only on niche organic and natural products and it is also the case for the Polish market.  What is more, the dossier underestimates elements related to reformulation costs, reformulation capacity and time which are key to impact assessment. It would result in a severe socio-economic burden on the cosmetics industry including for SMEs, resulting in an impact on competitiveness, jobs and growth of the sector and on consumer choice, for a very limited benefit to the environment. |  |  | To the best of our knowledge there are no known alternatives for many critical cosmetics functions. |  |
| 73 | The Swedish Cosmetic and Detergent Association and The Swedish Association of Professional Hygiene & Cleaning | The Swedish Cosmetic and Detergent Association and The Swedish Association of Professional Hygiene & Cleaning would like to give comments to the consultation of the ECHA proposal on restriction of microplastics. The answer covers detergents and maintenance products only.  Our comments concern the legal certainty and precautionary principle, the definition, the reporting obligation and the derogations for biodegradable and film forming polymers.  See attachment for further details. |  |  |  |  |
| 74 | Union Française des Semenciers | We support and agree with the ESA input done at this consultation |  |  |  |  |
| 75 | Verband der Chemischen Industrie e.V. (VCI) | Today we provide the English translation of the detailled German VCI position submitted to this public consultation on 20 March 2019 – see attachment.  **Comment:**  FOR DETAILS OF THE VCI POSITION SEE ATTACHMENT  Executive Summary of the VCI Position:  At the request of the European Commission, the European Chemicals Agency (EC-HA) has submitted a proposal to restrict microplastics within the framework of a so-called Annex XV dossier in accordance with the REACH Regulation (title: Proposal for a Restriction: Substance Name(s): intentionally added microplastics). A public consultation on this Annex XV dossier is ongoing until 20 September 2019.  The title of the restriction and also almost all statements in the dossier (e.g. statements on substance identity or risk assessment) suggest that it is a restriction of microplastics. In fact, however, the proposed restriction addresses all polymers and virtually all polymer-containing or polymer-coated materials. The specifications, definitions and scope of the restriction are so complex and so extensive that it is unclear what exactly should be covered.  In the opinion of the VCI, the restriction proposal infringes important provisions of the REACH Regulation and the tenets of the precautionary principle:  1. Insufficient description of substance identity:  The general addressing of all polymers or microplastics does not fulfil the requirements of the REACH Regulation for a precise identification of the substances to be restricted. Overall, it is unclear in the ECHA Annex XV dossier what exactly should be restricted - polymers or microplastics. The precise identification of the substances to be restricted as required under REACH and a risk assessment and assessment of socio-economic impacts based on this are missing.  2. Lack of identification of hazard and risk:  The provisions of Title VIII of REACH are disregarded by proposing a restriction in the absence of the first determining element of the risk - i.e. an identified hazard. Overall, with a simple reference to the "extreme stability" (persistence) of the particles, a fictitious, alleged risk is constructed, without having any evidence of a real risk or - after the scientific risk assessment - having any reasonable cause for concern that can be derived.    3. Lack of detail in the risk assessment:  Any risk assessment in accordance with REACH must be substance-related. A grouping of substances may be possible under certain, closely defined conditions. However, the demonstration required under REACH that all polymers or microplastic materials covered by the restriction have the same properties and thus the same risk is not provided.  4. Disregard of the principles and standards for the application of the precautionary principle:  The reasoning presented in the Annex XV dossier to justify the proposed restriction does not come up to the standard required in the European Union for the application of the precautionary principle. Overall, the scientific evidence presented in the Annex XV dossier is inadequate, incomplete and inconclusive.    5. Lack of efficacy, effectiveness and proportionality:  With the proposed restriction, only a small fraction of the microplastics introduced into the environment will be covered. The REACH requirement that a restriction must be appropriate to reduce risks to an acceptable level within a reasonable time and in a reasonable way is therefore not met.  Moreover, it will be virtually impossible to analytically demonstrate the effectiveness of the restriction by monitoring environmental concentrations resulting from the definition of the materials to be restricted - given the extremely wide particle size range of 1nm to 5mm and the complex structural requirements of e. g. "continuous polymer surface coatings of any thickness".  6. Lack of legal basis for extensive product labelling and for the proposed disproportionate annual reporting requirement:  It is not acceptable that a detailed labelling and an extensive annual reporting re-quirement are to be introduced for almost all polymer-containing products even if they are exempted from the restriction. Such obligations have to be fulfilled by all down-stream users. There is no sufficient legal basis for this.  Conclusions/Recommendations:    • The VCI does not reject a restriction of certain specified uses of microplastics in principle.    • The VCI prefers that restriction measures be taken within the framework of the REACH regulation.    • However, in order for the restriction now presented to comply with the requirements of the REACH Regulation, extensive adjustments must be made.    • First proposals for such adjustments are contained in the detailed VCI assessment (see attachment).  FOR DETAILS SEE ATTACHMENT |  | **Answer to specific info request 3:**  FOR DETAILS OF THE VCI ASSESSMENT SEE ATTACHMENT  Definition Microplastics: Konzentration/Dimension    Nach dem Beschränkungsvorschlag dürfen Polymere nicht als eigenständiger Stoff oder in einem Gemisch als Microplastics in einer Konzentration ab 0,01 % in Verkehr gebracht werden.    Als Microplastic werden Partikel ab 1 nm Größe mit einem Polymergehalt ab 1% oder einer kontinuierlichen Polymerbeschichtung beliebiger Dicke definiert.  Die in der Beschränkung vorgeschlagenen Definitionen führen zu folgenden Unklarheiten und Problemen:  • Problematische Erfassung einzelner Polymermoleküle:    Von der geringen unteren Größengrenze von 1 nm sind bereits einzelne Polymermoleküle erfasst. Diese sind oftmals keine Polymere, sondern kleinere Einheiten der Makromoleküle, so etwa n-Alkane wie sie in der Natur vorkommen. Für solche Moleküle ist eine Beschreibung des Zustands nach den etablierten Begriffen von fest und flüssig nicht möglich, wie ein aktueller Bericht des JRC aufzeigt:  “From the above classification it is also evident that single molecules cannot be solid (nor liquid), because the classification can only be applied to ensembles big enough to form a phase for which the state (solid, liquid, gaseous) can be assessed. This is one reason why single molecules, with the exemptions discussed above, do not fall under the EC NM definition, as pointed out previously.”    • Keine Möglichkeit der analytischen Erfassung/keine Möglichkeit der Kontrolle und des Vollzugs:    Eine analytische Erfassung einzelner Polymermoleküle oder nur weniger nanometergroßer Partikel ist nach aktuellem Stand der Technik nicht möglich, insbesondere nicht in komplexen Mischungen.  Die im Beschränkungsvorschlag der ECHA zitierte Literatur lässt einen ähnlichen Schluss zu: „Depending on the setup of the application small particles can also be measured down to the range of 20 μm or if needed even lower to the range of 1 μm us-ing micro-FTIR or micro-Raman (Primpke et al., 2017).”  Voraussetzung für die Wirksamkeit, Umsetzung Kontrolle der Beschränkung sind validierte Messmethoden von Polymeren und von Microplastic in diversen Medien. Die-se liegen bis dato noch nicht vor; schon gar nicht für den Größenbereich (1nm) und Konzentrationsbereich (0,01 %), wie im Annex XV-Dossier für die Beschränkung vorgeschlagen.  • Die Konzentrationsgrenze von 0,01 % für Polymere in Mischungen als Microplastic ist zu niedrig:  Der geringe erlaubte Gehalt von Polymere in Mischungen als Microplastic von weniger als 0.01 % verschärft die bereits erläuterten Schwierigkeiten in Bezug auf die Analytik. Selbst für PBT-Stoffe ist nach REACH mit 0,1 % ein höherer erlaubter Gehalt vorgegeben.  • Wasserlösliche Polymere fallen auch unter die Definition von Microplastic:  Der Beschränkungsvorschlag der ECHA-Definition zu „Microplastic“ umfasst sowohl wasserlösliche als auch nicht wasserlösliche Polymere. Laut separate Annex zum Annex XV Dossier (S. 19) gilt: “Solubility” is […] not proposed for inclusion as an ele-ment in the regulatory definition.” Dies ist problematisch, da wasserlösliche Polymere nicht in Form eines Partikels in der Umwelt, wie z. B. in Oberflächengewässern, vor-liegen. Das gesamte Beschränkungsdossier bezieht sich jedoch auf Microplastic als Partikel in der Umwelt.  Zudem sind die derzeit verfügbaren Methoden ungeeignet, um z. B. lösliche Polymere in der Umwelt nachzuweisen. Da die Probenvorbereitung eine Siebung der Proben vorsieht, werden nur partikuläre und nicht gelöste Bestandteile erfasst. Lösliche Polymer werden in einem Umweltmonitoring somit nicht erfasst.  Es stellt sich auch die Frage, warum lösliche Polymere mit unter den Beschränkungsvorschlag fallen, da die ECHA von der Kommission den Auftrag erhalten hat, die Beschränkung von wasserunlöslichen Polymeren zu betrachten.  • Unpraktikable Vorgabe des Polymeranteils von 1 % bzw. der Dicke der Beschichtung eines Partikels:  Neben der geringen Partikelgröße scheint der Polymeranteil von nur 1 % oder einer Monolage aus Material willkürlich. Es ist nicht ersichtlich, warum die Eigenschaften eines aus verschiedenen Materialien bestehenden Partikels (z. B. einem polymerbeschichteten Pigment) ab dieser Untergrenze von dem Polymer bestimmt werden. Auch anorganische Pigmente (z. B. Eisenoxid, Titandioxid), die zur Verwendung in wasserbasierten Farben mit einer Monolage eines wasserlöslichen Polymers beschichtet werden, werden somit zu Microplastic und fallen unter die Beschränkung.  Schließlich verschärft der geringe erlaubte Gehalt von als Microplastic definierten Partikeln von weniger als 0.01 % in Mischungen die bereits erläuterten Schwierigkeiten in Bezug auf die Analytik.  • Konsequenzen in der praktischen Umsetzung  Alle Kriterien zusammen genommen würden zum Beispiel bedeuten, dass in einem Endkundenprodukt wie einer Farbe oder einer Kosmetikformulierung 0,01 % eines 1 nm großen polymerhaltigen Partikels detektiert werden müssten. Dann müsste bewertet werden, ob die polymerhaltigen Partikel mehr als 1 % oder eine Monoschicht eines Polymers beinhalten.  Damit könnte theoretisch eine Gesamtkonzentration von 100 ppm eines Polymeren ausreichen, um ein Produkt aufgrund der Anwesenheit von Mikroplastik zu verbieten (1 % Polymeranteil an 0.01 % der Partikel).  Der VCI hat deshalb erhebliche Zweifel an der auf Seite 133 des Annex XV gezogenen Schlussfolgerung:  „The Dossier Submitter considers that the restriction is implementable and enforceable, although harmonised analytical methods for detecting microplastics in products are yet to be agreed and a framework of test methods and criteria for identifying (bio)degradable ‘microplastics’ will likely require additional research and development to progress beyond the criteria proposed here.”  Fazit:  Die bei der Definition von Microplastic vorgeschlagene Kombination aus einer unteren Dimensionsgrenze von 1 nm, einem Polymeranteil von 1 % oder einer durchgängigen Polymerschicht beliebiger Dicke in einem polymerhaltigen Partikel sowie einer erlaubten Microplastic-Konzentration von 0.01 % haben zur Folge, dass praktisch ausnahmslos alle polymerhaltigen Stoffe und Gemische unter die Beschränkung fallen und eine Abgrenzung nicht möglich ist.    Der gesamte Beschränkungsvorschlag ist sehr schwer verständlich, so dass eine erhebliche Rechtsunsicherheit geschaffen wird.    Mit bestehenden analytischen Verfahren sind eine Umsetzung und ein Vollzug nicht zu gewährleisten, was zusätzlich zur Rechtsunsicherheit beiträgt.    Folgende Anpassungen sind daher mindestens erforderlich:  - Anhebung der unteren Partikelgrenze auf 1 µm  - Anhebung der 1 % w/w Konzentrationsgrenze eines polymerhaltigen Partikels auf eine Konzentration, ab der das Polymer entscheidend ist für die Eigenschaften des Partikels  - Anhebung der erlaubten Mindestkonzentration von 0,01 % auf 0,1 % analog zu PBT/ vPvB Substanzen  - Die Vorgaben in der Beschränkung müssen so gestaltet werden, dass geeignete Messverfahren zu Verfügung stehen. Dies schließt auch die Konzentrationsgrenze ein.    - Wasserlösliche Polymere sollten nicht unter die Beschränkung fallen. |  |  |
| 76 | Verband der deutschen Lack- und Druckfarbenindustrie e.V. | The proposed labelling and, in particular, the comprehensive reporting requirements for manufacturers of paints, coatings and printing inks and also for industrial users of these products involve a large amount of bureaucracy – while they come nowhere near the given objective of tracking uses and potential releases to the environment. The reasons are, firstly, that consumers and craftspeople, who use large shares of building paints, are exempted from the reporting requirements. Secondly, a total volume of 1.2 million tonnes of paints, coatings and printing inks is annually exported to other EU Member States.  The obligation of industrial users (e.g. in the automotive and printing industries) to estimate the release of microplastics to the environment overlooks that comprehensive water protection rules are already in place for industrial sites. These rules regulate how to treat waste waters prior to their direct release into waters or their indirect release. Thus, the above proposal stands in contradiction the principle of subsidiarity.  The comprehensive labelling and reporting requirements for the paints and printing inks industry, which only accounts for a fraction of microplastics used, are ineffective and disproportionate. Either the whole supply chain would need to be covered (with a further increase of the workload and costs for the industry), or it would be sufficient to exclusively cover those industries that manufacture microplastics or place them on the market for the first time.  Most microplastics stem from decomposing plastic wastes, tire abrasion, or laundering of synthetic clothing. Only a very minor share originates from intentionally added microplastics. The disposal of plastic wastes in the environment is a global problem, and more effective solutions for waste avoidance and recovery need to be found. The proposed regulation is disproportionate, scientifically inadequate and of no benefit to the environment.  Therefore, we suggest (1) a clearer and more focused definition of the scope of the proposed restriction (Table 3, No. 2); (2) a deletion of reporting requirements for industrial uses (Table 3, No. 8); and (3) industrial producers should be free in providing “instructions for use” according to the specifications of the products (Table 3, No. 7).  Additional information is provided in our position paper in the attachment.  **Answer to specific info request 5:**  The ECHA proposal includes labelling and, in particular, comprehensive reporting requirements for the manufacturers and industrial users of many paints, coatings and printing inks. In Germany alone, more than 60,000 companies should be impacted, including roughly 8,000 print shops.  The labelling requirement would impact in Germany 250 manufactures of paints and printing inks; most of them are small and medium-sized enterprises (SMEs).  The reporting requirement concerns (1) Manufacturers of binders; (2) ca. 250 companies in Germany who – as “downstream users” – use binders for the manufacture of paints, coatings and printing inks; (3) many industrial users of paints, coatings and printing inks who – also as “downstream users” – would be subject to reporting, for example automotive industry, metal industry, print shops, furniture industry, electrical industry, mechanical engineering companies, corrosion protection businesses, industrial window coating businesses; and (4) many thousands of users and retailers who mix paints/coatings according to their customers’ wishes in mixing stations – where individual, tailor-made paints/coatings are placed on the market at the point of sale.  The proposed annual reporting requirement would constitute an excessive strain. Without an impact assessment, it remains unclear what effort and costs the reporting requirement precisely involves. Based on the current proposal, the manufacturers of paints, coatings and printing inks (depending on size and product portfolio) estimate at least 50% of one full-time position for a suitably qualified person. For our sector, this would mean additional costs of at least 6 million euros per annum. | **Comment:**  ECHA’s restriction proposal contradicts the provisions of the REACH Regulation (Annex XV and Section 2 of Annex VI) by assessing and acting on a “group of substances identified generically”. The proposal includes a definition of microplastics, which is extremely wide as well as unclear, and subjective underlying criteria for labelling and reporting requirements.  ECHA has exceeded its authority under Title VIII of REACH by proposing a restriction in the absence of the first defining both the element of risk and an identified hazard. The scientific evidence alleged to substantiate the proposed restrictions does not meet the standard of evidence required by the preponderance of case law on the application of the precautionary principle. | **Answer to specific info request 1:**  A degradation of the polymers used in coatings is not desirable, because in the sense of sustainability, durability and maintainability are very important in the building und industrial sector. Coating materials make a considerable contribution to resource preservation. Paints, coatings, plasters and other coatings protect surfaces against external influences and thus extend the service life of buildings, vehicles, installations, mechanical equipment and other items of daily use.  **Answer to specific info request 3:**  ECHA’s restriction proposal contains a definition of microplastics, which is too broad and leaving much room for interpretation. According to our interpretation of microplastic definition, corresponding polymers are used in many paints, coatings and printing inks - primarily as film-formers and non-film forming additives:  Film-forming function: Polymers according to the definition in table 3 of the proposal are used as binders in many applications, including paints, coatings and printing inks. Binders can be liquid (e.g. oils), semi-solid (e.g. waxes) or solid polymers (e.g. resins). In waterborne products the polymers are dispersed in water while in solvent based products the polymers are dissolved in organic solvents. Binders encase the solid components of paints and varnishes, i.e. pigments and fillers, and form solid polymer-containing particles.  The function of binders is to serve film formation by binding the components of paints and coatings with each other and with the substrate. Only binders enable film formation in coatings through polymerisation, polycondensation or polyaddition. Film formation, e.g. drying and hardening, brings about a hard and mechanically resistant layer that adheres to the substrate. Through the physico-chemical process of film formation, binders lose the particle property of microplastics according to the definition under 2 and, consequently, fall under the mentioned rule 5.b. Furthermore, these are firmly incorporated in a polymer structure (binder matrix) by curing, so that they are subject to rule 5.c of the restriction proposal. The share of binders in the composition varies from 2% (printing inks) over 25% (building paints) to up to 80% (powder coatings, printing inks).  Non-film forming function: Small quantities of polymer-based additives (e.g. waxes or spheres) are added to coating materials, in order to improve or modify their properties. Additives for paints and coatings are bound in a polymer structure in curing and fall under rule 5.c of the proposal. Additives are used in paints, coatings and printing inks in quantities from 2 % to 10%.  The paint manufacturers do not obtain details – e.g. on identity and concentration of the polymers used in pre-products (for example, binders and additives) – from their upstream suppliers. Without such particulars neither the paint manufacturers nor the industrial users can report any information on type of polymers and release to environment.  For self-control and for monitoring within the supply chain, identification of the corresponding polymers in various media is necessary. Suitable analytical methods are not available, especially not for the size range  (1 nm) and concentration range (0.01%). The development of new methods is hardly possible for these large number of different polymers covered by the microplastic definition of the ECHA restriction proposal. |  |  |
| 77 | Wirtschaftsverband Papierverarbeitung (WPV) e.V. | The German Paper and Board Converting Companies as industrial users of paints, coatings and printing inks do neither have any information – e.g. on the identity of the polymers used in printing inks or varnishes – nor would obtain such details from their upstream suppliers (e.g. printing ink manufacturers). The reason is that otherwise confidential business information would need to be disclosed. However, without such particulars the paper and board converting industries cannot report any information, for instance, about the identity of polymers. Ideally, the upstream business operator from the chemical industries should provide the necessary information to the authorities. |  |  |  |  |
| 78 | Cefic - Resins Technical Platform (RTP) | **Content:**  Scope or restriction option analysis |  |  |  |  |

**References**

Buxton, L., 2018, Oil and gas industry faces microplastics scrunity, ChemicalWatch, Link: <https://chemicalwatch.com/65720/oil-and-gas-industry-faces-microplastics-scrutiny?q=microplastic> – accessed 20-8-2019.

Buxton, L., 2019, Legal opinion casts doubt over proposed EU microplastics restriction, ChemicalWatch, Link: <https://chemicalwatch.com/77579/legal-opinion-casts-doubt-over-proposed-eu-microplastics-restriction?q=microPlastics> - accessed 11-6-2019.

ChemicalWatch (CW), 2018f, Industry prioritises microplastics for chemical risk research funding, Link: https://chemicalwatch-com.proxy.findit.dtu.dk/69887/industry-prioritises-microplastics-for-chemical-risk-research-funding?q=microPlastics - accessed 14-8-2019.

ECHA, 2019, General Comments and answers to specific information requests, Helsinki: European Chemicals Agency, Link: https://echa.europa.eu/registry-of-restriction-intentions/-/dislist/details/0b0236e18244cd73 - accessed 28-10-2019

Lovell, T., 2017, Cosmetics firms urge UK to limit microbeads ban to rinse-off products, ChemicalWatch, Link: <https://chemicalwatch.com/54402/cosmetics-firms-urge-uk-to-limit-microbeads-ban-to-rinse-off-products?q=microplastic> – accessed 20-8-2019.

Lovell, T., 2017, UK government urged to target ‘major sources’ of microplastics, ChemicalWatch, Link: <https://chemicalwatch.com/51974/uk-government-urged-to-target-major-sources-of-microplastics?q=microplastic> – accessed 20-8-2019.

Morgan, S., 2018, Commission maps out plastics vision in new strategy, EURACTIV, Link: <https://www.euractiv.com/section/energy-environment/news/commission-maps-out-plastics-vision-in-new-strategy/> - accessed 28-10-2019

Oziel, C., 2018, NGOs attack Echa’s ‘limited’ microplastics restriction proposal, ChemicalWatch, Link: https://chemicalwatch-com.proxy.findit.dtu.dk/67582/ngos-attack-echas-limited-microplastics-restriction-proposal?q=microPlastics - accessed 14-8-2019.

Oziel, C., 2018, Oil and gas industry challenges EU estimate on microplastics use, ChemicalWatch, Link: https://chemicalwatch-com.proxy.findit.dtu.dk/67188/oil-and-gas-industry-challenges-eu-estimate-on-microplastics-use?q=microPlastics - accessed 14-8-2019.

Oziel, C., 2019, Top German sports associations call for artificial turf ban transition, ChemicalWatch, Link: <https://chemicalwatch.com/77732/top-german-sports-associations-call-for-artificial-turf-ban-transition?q=microPlastics> - accessed 11-6-2019.

Ramm, K., 2018, Time to invest in Europe’s water infrastructure, EURACTIV, Link: <https://www.euractiv.com/section/energy-environment/opinion/time-to-invest-in-europes-water-infrastructure/> - accessed 28-10-2019.

Stringer, L., 2019, Feature: EU plastics plan presents opportunities and production challenges, ChemicalWatch, Link: https://chemicalwatch.com/74101/feature-eu-plastics-plan-presents-opportunities-and-production-challenges?q=microPlastics - accessed 12-6-2019.

Tani, C., 2019, Echa definition of microplastics ‘too broad’ – Cefic, ChemicalWatch, Link: https://chemicalwatch.com/74140/echa-definition-of-microplastics-too-broad-cefic?q=microPlastics - accessed 12-6-2019.
