# Supplementary material for: Stakeholder analysis with regard to a recent European restriction proposal on microplastics
Source: PLoS One. 2020 Jun 22;15(6):e0235062. doi: 10.1371/journal.pone.0235062 (PMC7307934; doi:10.1371/journal.pone.0235062)
Supplement: S4 Table — (DOCX) [file pone.0235062.s005.docx]

S4 Table: International Authorities categorization table

| ***International Authorities (politicians, parliaments, etc.)*** | | | | | | |
| --- | --- | --- | --- | --- | --- | --- |
| **No**. | **Stakeholder** | **Criteria**  (for statement e.g. economy, innovation, environment etc.) | **Principles**  (separate from criteria e.g. PP, values etc.p) | **Scientific argumentation**  Case Reports & Case Series (observational)  Case-control (observational)  Cohort (observational)  Randomized-controlled trials (experimental)  Systematic review | **Research needs**  (identified needs in statement) | **Other** |
| 1 | AMAP | The reports says there is a need for timely and effective action on chemicals of emerging Arctic concern. (CW, 2017m). |  | Chemicals of emerging concern are those discovered in humans, the environment or water sources that had not previously been detected, or were only present at insignificant levels. They are persistent and may have the potential to cause adverse health effects. (CW, 2017m). |  |  |
| 2 | ECHA | ECHA has made available the video of its microplastics restriction (WC, 2019e).  Started a consultation on its proposal for restriction on intentionally added microplastics in consumer products in April 2019. The consultation is open until 20 September 2019 (Buxton, 2019).  Echa published the proposal on 30 January, after a request by the European Commission a year earlier for a REACH Annex XV restriction dossier for microplastics (CW, 2019g).  Published an [inventory](https://chemicalwatch.com/74545/) containing information on 419 substances used as additives in plastics in the union (CW, 2019l).  [Echa, Commission allay fears of artificial turf closures after microplastic ban](https://chemicalwatch.com/80620/echa-commission-allay-fears-of-artificial-turf-closures-after-microplastic-ban) (Tani, 2019).  Echa has published a Q&A document to help respondents to the public consultation on the proposal to restrict intentionally added microplastics, which runs from 20 March to 20 September (CW,2019u).  January 30, Echa published a REACH restriction proposal for intentionally added microplastics (Santoni, 2019).  ECHA has submitted restriction proposal on [microplastics](https://chemicalwatch.com/63198/) when intentionally added to consumer or professional use products of any kind (CW, 2019y).  ECHA proclaims that intentionally added microplastics are more likely to enter and accumulate in terrestrial and freshwater environments rather than the oceans (CW, 2018b).  Additives to cosmetic products account for a “relatively tiny” part of microplastics finding their way into the environment compared to agricultural products, a senior scientific officer from the European Chemicals Agency (ECHA) said on Friday. (ends 2019c)  Head of unit at the Helsinki-based regulator, Matti Vainio, told a Commission conference on Friday that the restrictions, which aim to avoid almost 30,000 tonnes of microplastics a year entering the environment, could come into force in 2020. (ends 2019e) |  | “We have identified diverse sources of microplastics to the environment from ‘intentional uses’ in cosmetics, detergents, other household products, paints, and agricultural uses, amongst others. Many of these microplastics are washed down the drain at the point of use and because of how wastewater in the EU is treated, these microplastics will not typically be released directly to aquatic environments, but are likely to concentrate in sewage sludge that is then frequently applied to agricultural soils as a fertiliser,” he said. (Ends 2018a) | Published a note outlining the identification of microplastics and the steps the agency will take to understand remaining unresolved issues (CW, 2018k).  In its reply to the NGO letter of the Helsinki workshop on microplastics on the May 30-31, Ms McGuinness said:  Echa’s scope for the call for evidence was "very wide" and covered all intentionally added microplastics in all uses. A call for evidence, she added, is just "one of the means" by which the agency gathers information when investigating a potential restriction and preparing a restriction report. Additionally, she said, Echa did ask for information on releases of microplastics to the environment, which it regards as being "important" in assessing risks. It did this, she added, in an objective fashion. "Echa has screened 15,000 scientific articles that could be relevant for this specific restriction," she said. The agency also requested that stakeholders collate additional information "such as ongoing unpublished research" related to the risks of microplastics (Oziel, 2018).  Echa said it will publish "further thoughts" on its approach to the definition in June, but did say that it is wary of a definition being  "too simplistic for the purposes of a REACH restriction in practice" (Oziel, 2018).  The oil and gas industry has been named as an area for investigation in Echa’s recent call for evidence on the use of products with intentionally added microplastics. (Buxton, 2018)  The consultation, which has a deadline of 11 May, follows a European Commission request to investigate the need for a [restriction](https://chemicalwatch.com/63198) on such particles. The scope, Echa said, is "intentionally wide" and is not limited to intentional uses in [consumer](https://chemicalwatch.com/63737/) and professional products. (Buxton, 2018)  “The granular infill material that is typically used in artificial turf pitches is understood to be an ‘intentionally-added microplastic’, but neither ECHA nor the European Commission are proposing that these pitches should be closed,” (Ends 2019a)  “All factors, including the important role that sport fields play in promoting physical exercise, health and social inclusion, are taken into account in the decision-making process,” (Ends 2019a)  McGuinness responded that ECHA “did ask for information on releases of microplastics to the environment” and “screened 15,000” relevant scientific articles.  “ECHA  considered it appropriate and efficient to request in this call for evidence that stakeholders collate additional information such as ongoing unpublished research related to the risks of microplastics,” she wrote. (Ends 2018g) |  |
| 3 | ECHA’s enforcement forum | Have until spring 2020 to comment on the restriction proposal for intentionally added microplastics by ECHA (CW, 2019g). |  |  |  |  |
| 4 | EEA | “[*Governments*](https://chemicalwatch.com/74304)*and authorities in the EEA have been taking measures to tackle the issue of plastic pollution*” (Buxton, 2019). |  | The sector’s (textiles, red) large environmental footprint comes from both its natural and synthetic fibres, with the former (e.g. cotton, wool) reflecting the resource intensity of agriculture while the latter are mainly fossil based and responsible for “a lot” of the microplastics found in the ocean, Mortensen said. (Ends 2019b) |  |  |
| 5 | EEB | [Criticised](https://chemicalwatch-com.proxy.findit.dtu.dk/67278/) the Commission’s draft laws for failing to address the presence of hazardous substances used in single-use plastic products (Buxton, 2018).  We’ll be pushing hard to tighten this proposal to ensure real impact,” said European Environmental Bureau chemicals policy officer Elise Vitali on behalf of the campaign platform Rethink Plastic. (About the ANNEX 15 restriction proposal) (Ends 2019e) |  |  |  |  |
| 6 | EFSA |  |  |  | “Currently there is no evidence to suggest that there is a food safety risk. The European Food Safety Authority (EFSA) has played down any concerns over what it has referred to as an "emerging issue".” (Burrows 2017) |  |
| 7 | EOSCA | The European Oilfield Speciality Chemicals Association has warned its members, as well as non-members, that the consequences could be "heavy controls or even bans being placed on the marketing and use of products containing microplastics". (CW, 2018x) |  |  |  |  |
| 8 | European Commission | “A few months ago, commission vice-president Frans Timmermans had already questioned the feasibility of a specific tax on microplastics. "The only sustainable method is to create recyclable plastic and take out microplastics," the Dutchman said [according to The Guardian](https://www.theguardian.com/environment/2017/oct/06/eu-rules-out-tax-on-plastic-products-to-reduce-waste).  "You can't take out microplastics with a tax. You need to make sure things are reused, and not put in the ocean."” (Teffer 2018)  "You can't take out microplastics with a tax," Commission deputy head Frans Timmermans said at an event in Malta on Friday. "Nothing disciplines companies more than consumer practices," he said, referring to consumer trends toward recyclable packaging.” (EUObserver 2017)  “Speaking to journalists at the same event, EU environment commissioner Karmenu Vella said: "Nothing disciplines companies more than consumer practices. We are on the verge of changing consumer habits. I sense a turning point, like that we saw 10 to 15 years ago on climate change." (Burrows 2017)  The European Commission looks set to propose major changes to the provisions in the EU ecolabel Regulation, disqualifying products that contain substances classified as toxic or as SVHCs. (CW, 2017h)  All detergents with the label must now be free of microplastics. (CW, 2017k).  The European Commission will introduce stricter limits to its EU Ecolabel criteria for cleaning products in June. (CW, 2017n).   - Microplastics will be banned from products (CW, 2017n).   The two other major issues highlighted were microplastic beads used in personal care products and their contribution to plastics litter in the marine environment and, the Commission’s upcoming strategy for endocrine disrupting chemicals (EDCs). (Chynoweth, 2013).  “The European Commission proposed new rules targeting the 10 single-use plastic products most often found on beaches and seas in the EU, as well as lost and abandoned fishing gear, constituting together 70 % of all marine litter items. The proposed rules will include a ban of certain products, consumption reduction, collection targets, and obligations for producers” (Ketelsen, 2018)  Karmenu Vella, the European Commissioner for Environment, Maritime Affairs and Fisheries: “As for microplastics, we are indeed working on restricting their use in certain products, such as cosmetics. To cut consumption of single-use plastic bags we have set clear and binding targets, leaving member states the flexibility to choose the most efficient way of reaching them, be it charges or other forms of restrictions.” (Morgan, 2018) |  | **“**Oxobiodegradable plastics are a case in point. Though sold on the basis that they will help to "protect the environment" because they biodegrade, the European Commission thinks otherwise: "[They] have been found to offer no proven environmental advantage over conventional plastics, while their rapid fragmentation into microplastics cause concerns.**”** (Burrows 2018) | In January 2018 the European Commission published the EU strategy on plastics (Buxton, 2019) including a  request to ECHA to prepare a REACH Annex XV restriction dossier on the use of intentionally added microplastic particles in all consumer and professional use products (Buxton, 2019).  Proclaim in a report of 2017 that "evidence that suggests the use of microplastics in offshore oil and gas could be substantial, in the magnitude of hundreds of tonnes" (Oziel, 2018).  The European Commission has asked Echa to prepare a REACH Annex XV restriction dossier on the use of intentionally added microplasticparticles to all consumer and professional use products. (Buxton, 2018)  The Commission has also asked Echa to work on a REACH restriction proposal for the use of oxo-plastics – these are used in carrier bags and food packaging. They include additives designed to promote oxidation of the material so that it fragments. (Buxton, 2018)  A three-year Commission reassessment of the ecolabel’s value - which concludes that the scheme is worth keeping - says it is not being used widely enough to make a significant difference to product use and production patterns - and partly blames the requirements on hazardous substances. (CW, 2017h).  The European Commission has opened a public consultation on policy options to reduce microplastics entering the marine environment. (CW, 2017j).  EU Environment Commissioner Janez Potočnik has underlined concerns about the chemical content of plastics during a conference in Brussels this week. (CW, 2013a).  Some respondents to the green paper also said that current legislation does not sufficiently address the risks arising from the use of microplastics in products. (CW, 2013a).  Meanwhile, this week, the Netherlands invited EU member states and the European Commission to start discussion on the occurrence of microplastics in water systems and to propose a way forward on this issue. (Chynoweth, 2013).  Natasha Bertaud, executive spokesperson, “The European Union will not ban artificial turf pitches, at least yet, but the European  Chemical Agency (ECHA) is looking at alternatives, as this material is an important source of microplastics.  “No such proposal is under preparation form the Commission,” (Rios, 2019)  “The European Commission revealed on Thursday (1 February) how it plans to update drinking water rules, with the goal of improving human health, reducing emissions and tackling plastic waste high on the list of priorities.” (Fortuna, 2019).  Frans Timmermans, vice-president of EC, *“The only sustainable method is to create recyclable plastic and take out microplastics. You can’t take out microplastics with a tax. You need to make sure things are reused, and not put in the ocean.”* (Harvey, 2017)  Environment commissioner Karmenu Vella noted that a proposed new risk-based approach to water quality monitoring would also apply to the new category of contamination by microplastics.  “There is no conclusive evidence that they pose a threat to the water supply, but we want to be on the safe side. If it is established that microplastics pose a threat, suppliers will have to act and make certain that they don’t pollute tap water,” Vella said. (Ends 2018j) |  |
| 9 | European Commission’s chief scientific advisors | “*The European Commission’s group of chief scientific advisers has recommended that the EU executive broadens existing policy to reduce and ultimately prevent*[*microplastic*](https://chemicalwatch.com/75836/)*pollution*” (Buxton, 2019). |  |  |  |  |
| 10 | European Council |  |  |  | Calls for the Commission to boost research and innovation in reducing unintentional leakage of microplastics into the environment (Buxton, 2018). |  |
| 11 | European Parliament | Backed that levels of microplastics is monitored (CW, 2018c).  Voted 597 to 15, to support a ban on microplastics intentionally added to cosmetics, personal care products, detergents and cleaning products by 2020 (Stringer, 2018)  Europe should impose a complete ban on oxo-degradable plastics by 2020, and prohibit the use of microplastics in cosmetics, the European Parliament said on Thursday as it passed a resolution on the EU executive's strategy on plastics. (Ends 2018d) |  |  |  |  |
| 12 | EP’s Environment Committee | In July, called on EC to [bolster](https://chemicalwatch-com.proxy.findit.dtu.dk/68664/envi-presses-eu-commission-to-toughen-up-plastics-strategy) its plastics strategy, by banning microplastics in cosmetics, personal care, detergents and cleaning products by 2020 in order to protect the oceans (Buxton, 2018) |  |  |  |  |
| 13 | IUCN |  |  | A report from conservation group IUCN estimated on Wednesday that between 0.8 and 2.5 million tons of primary microplastics end up in oceans worldwide every year, the equivalent of one plastic bag cast away per person per week. These are particles smaller than five millimetres released directly into the environment.  According to the analysis, between 15% and 31% of all plastic currently in oceans could originate from these primary sources, while the rest comes from secondary, larger plastic waste that degrades into smaller fragments in the water. The proportion of primary microplastics is even larger in Europe and North America, which are better at dealing with secondary sources through sound waste management, it added. (Ends 2017a) |  |  |
| 14 | MEPs | “MEPs voted on Tuesday (10 July) to amend European Commission plans to cut plastic pollution and suggested a ban on microplastics in cosmetics and other products by 2020, as the European Parliament starts to review the Plastics Strategy” (Morgan, 2018).  Mark Demesmaeker, European Conservatives and Reformists (ECR) MEP: “largely welcomed the Commission’s January strategy but called for a ban on micro-plastics in cosmetic and cleaning products by the end of the decade.“ (Morgan, 2019)  “MEPs voted to amend Commission plans to cut plastic pollution, they also suggested a ban on microplastics, often used in the production of artificial turf” (Valero & Kirk, 2018)  Magrete Auken, European Greens, “No biodegradable plastic bag is a solution to littering. But the so called ‘oxo-biodegradable’ plastic bags will fragment into microplastics in the environment” (Crisp, 2014) |  |  |  |  |
| 15 | Nordic Council of Ministers | In April 2019 the Nordic Council of Ministers, urged [firmer action](https://chemicalwatch.com/76283) to combat plastic and microplastic pollution in seas and oceans (Buxton, 2019).  Environment ministers from the Nordic Council countries have launched a programme to reduce the global impact of plastics on the environment. (CW, 2017l).  Its programme sets out a vision for more sustainable use and recycling of plastics in a circular economy, in particular, aiming to reduce plastic debris and microplastics in the seas. (CW, 2017l).  A Nordic inter-parliamentary committee has backed a proposal that recommends banning the use of microplastics in cosmetics across five Nordic countries. ( Zainzinger, 2017).  Chairperson of the committee, Hanna Kosonen, said the ban could be a signal to policy makers around the world, “because it puts the adverse effects of microplastics in the spotlight”. (Zainzinger, 2017).  Ms Kosonen said the committee does not expect passage of the measure would dramatically reduce microplastics in seas and rivers. (Zainzinger, 2017).  The document – which has been sent to the EU’s governing bodies, the UN Environment Programme, and the G7 and G20 groups of nations – also asks the NCM to prepare a study to consider which specific elements should be included in a global agreement on microplastics and plastic waste in marine ecosystems. (ends 2019d)  The sustainability committee of the Nordic Council, an inter-parliamentary body, last week voted in favour of a proposal recommending a complete ban on adding microbeads to products such as body scrubs and toothpaste.  “This is because it puts the adverse effects of microplastics in the spotlight and has the potential to accelerate political action and decisions to the benefit of both people and the environment,” said Hanna Kosonen, who chairs  the committee. (Ends 2017b) |  |  |  |  |
| 16 | Nordic Countries | “*Nordic environment and climate ministers are urging firmer action to combat plastic and microplastic*[*pollution*](https://chemicalwatch.com/76147/r)*in seas and oceans*” (Buxton, 2019).  “*The ministers stressed it is a global problem and more concrete measures need to be implemented worldwide in order to make progress. They acknowledge recent wider recognition of the issues, but decry a "lack of focus on the need for stricter and more committal global governance"*” (Buxton, 2019).  *"The Nordic region must be a pioneer in reducing the environmental impact of plastics. With this declaration, we are continuing to take the lead globally,"* (by Guðmundur Ingi Guðbrandsson, environment minister for Iceland, holding the presidency of the Nordic Council of Ministers (Buxton, 2019). |  |  |  |  |
| 17 | RAC | Have until spring 2020 to comment on the restriction proposal for intentionally added microplastics by ECHA (CW, 2019g).  Confirm the upcoming restriction on intentionally-added microplastics in March 2019 (Davies, 2019) |  |  |  |  |
| 18 | SAPEA |  |  |  | Professor Bart Koelmans, chair of the Science Advice for Policy by European Academies (SAPEA) working group, said that the “lack of evidence for risk does not mean we should assume that there is no risk”.  “Concentrations of microplastics in the environment are increasing”, he said, adding that “if we keep polluting at the current rate, we will have a real problem in the future” (Ends 2019f). |  |
| 19 | UN Environment | Conclude in its 2018 *Summary For Policy Makers* document that "*current governance strategies and approaches provide a fragmented approach that does not adequately address marine plastic litter and microplastics”* (CW, 2019m).  Jacob Duer, chief, chemicals and health branch, UN Environment Programme stated that “*plastic waste is emerging as one of our greatest environmental challenges*” (CW, 2019o). |  |  | A UN report has described an "urgent need" to study the presence of microplastics, and associated chemicals, in the edible parts of fish destined for human consumption. (Davies, 2017). |  |
| 20 | UNEA | Erik Solheim, ”Their plan for new legislation on single-use plastics, renewed action on microplastics and regulation on biodegradeable standards is exactly the kind of leadership we need. We have no time to lose.” (Morgan, 2018) |  |  | “[*Resolved*](https://papersmart.unon.org/resolution/uploads/k1800210.english.pdf)*to convene meetings of an ad hoc open-ended expert group on marine litter and microplastics*”, Dec. 2017 (CW, 2019m) |  |
| 21 | UNEP | In March 2019 the 4^th^ UN environment assembly decided to continue “*the intergovernmental process relating to marine litter and the proliferation of microplastics and the expert group created to identify stronger international governance structures*” (Buxton, 2019).  A UN Environment Programme (Unep) report recommends an eventual phase-out and ban on microplastics in personal care products and cosmetics. (CW, 2015c). |  |  |  |  |
| 22 | WHO | “Microplastics are of particular concern due to the negative effects on marine and freshwater environments, aquatic life, biodiversity, and possibly to human health since their small size facilitates uptake and bioaccumulation by organisms, or toxic effects from the complex mixture of chemicals these particles consist of,” (Ends 2018k) |  |  |  |  |

**References**

# Burrows, D., 2017, Microplastics threat poses dilemma for new EU strategy, EUobserver, Link: <https://euobserver.com/health/140194>

Buxton, L., 2018, Envi presses EU Commission to toughen up plastics strategy, ChemicalWatchLink: https://chemicalwatch-com.proxy.findit.dtu.dk/68664/envi-presses-eu-commission-to-toughen-up-plastics-strategy?q=microPlastics - accessed 14-8-2019.

Buxton, L., 2018, Council of Ministers urges action on tracking substances of concern, ChemicalWatchLink: <https://chemicalwatch-com.proxy.findit.dtu.dk/68023/council-of-ministers-urges-action-on-tracking-substances-of-concern?q=microPlastics> - accessed 14-8-2019.

Buxton, L., 2018, Oil and gas industry faces microplastics scrunity, ChemicalWatch, Link: <https://chemicalwatch.com/65720/oil-and-gas-industry-faces-microplastics-scrutiny?q=microplastic> – accessed 20-8-2019.

Buxton, L., 2018, EU prepares comprehensive microplastics restriction, ChemicalWatch, Link: <https://chemicalwatch.com/63198/eu-prepares-comprehensive-microplastics-restriction?q=microplastic> – accessed 20-8-2019.

Buxton, L., 2019, Chief scientists back broader EU policy to tackle microplastics pollution, ChemicalWatch, Link: <https://chemicalwatch.com/77313/chief-scientists-back-broader-eu-policy-to-tackle-microplastics-pollution?q=microPlastics> - accessed 11-6-2019.

Buxton, L., 2019, Picture of hazardous chemicals in Finnish waters now ‘complete’, ChemicalWatch, Link: <https://chemicalwatch.com/77258/picture-of-hazardous-chemicals-in-finnish-waters-now-complete?q=microPlastics> - accessed 11-6-2019.

Buxton, L., 2019, Nordic countries push for global agreement on ocean plastics, ChemicalWatch, Link: <https://chemicalwatch.com/76283/nordic-countries-push-for-global-agreement-on-ocean-plastics?q=microPlastics> - accessed 11-6-2019.

Buxton, L., 2019, Echa outlines proposed microplastics restriction measures, ChemicalWatch, Link: https://chemicalwatch.com/73819/echa-outlines-proposed-microplastics-restriction-measures?q=microPlastics - accessed 8-8-2019.

ChemicalWatch (CW), 2013a, EU consultation reveals concerns about plastics chemical content, Link: <https://chemicalwatch.com/16685/eu-consultation-reveals-concerns-about-plastics-chemical-content?q=microplastic> – accessed 20-8-2019.

ChemicalWatch (CW), 2015c, Unep report calls for phase-out of microbeads, Link: <https://chemicalwatch.com/24108/unep-report-calls-for-phase-out-of-microbeads?q=microplastic> – accessed 20-8-2019.

ChemicalWatch (CW), 2016f, Efsa calls for standard analytical methods for microplastics, Link: <https://chemicalwatch.com/48280/efsa-calls-for-standard-analytical-methods-for-microplastics?q=microplastic> – accessed 20-8-2019

ChemicalWatch (CW), 2017h, Rethink on the cards for EU ecolabel’s chemicals provisions, Link: <https://chemicalwatch.com/57302/rethink-on-the-cards-for-eu-ecolabels-chemicals-provisions?q=microplastic> – accessed 20-8-2019.

ChemicalWatch (CW), 2017j, European Commission opens public consultation on marine microplastics, Link: <https://chemicalwatch.com/57215/european-commission-opens-public-consultation-on-marine-microplastics?q=microplastic> – accessed 20-8-2019.

ChemicalWatch (CW), 2017k, Revised EU Ecolabel criteria for cleaning products published, Link: <https://chemicalwatch.com/57176/revised-eu-ecolabel-criteria-for-cleaning-products-published?q=microplastic> – accessed 20-8-2019.

ChemicalWatch (CW), 2017l, Nordic Council launches sustainable plastics programme, Link: <https://chemicalwatch.com/55559/nordic-council-launches-sustainable-plastics-programme?q=microplastic> – accessed 20-8-2019.

ChemicalWatch (CW), 2017m, Arctic monitoring programme finds new chemicals of confern, Link: <https://chemicalwatch.com/55415/arctic-monitoring-programme-finds-new-chemicals-of-concern?q=microplastic> – accessed 20-8-2019.

ChemicalWatch (CW), 2017n, EU Ecolabel introduces new criteria for cleaning products, Link: <https://chemicalwatch.com/55038/eu-ecolabel-introduces-new-criteria-for-cleaning-products?q=microplastic> – accessed 20-8-2019.

ChemicalWatch (CW), 2018b, Intentional microplastics are primarily a soil and freshwater problem, Echa finds, Link: https://chemicalwatch-com.proxy.findit.dtu.dk/72238/intentional-microplastics-are-primarily-a-soil-and-freshwater-problem-echa-finds?q=microPlastics - accessed 14-8-2019.

ChemicalWatch (CW), 2018k, Agency publishes new microplastics note, Link: <https://chemicalwatch-com.proxy.findit.dtu.dk/68652/echa-round-up?q=microPlastics> - accessed 14-8-2019.

ChemicalWatch (CW), 2019e, ECHA round-up, Link: <https://chemicalwatch.com/76833/echa-round-up?q=microPlastics> - accessed 11-6-2019.

ChemicalWatch (CW), 2019g, Echa begins consultation on microplastics restriction proposal, Link: https://chemicalwatch.com/75836/echa-begins-consultation-on-microplastics-restriction-proposal?q=microPlastics - accessed 11-6-2019.

ChemicalWatch (CW), 2019l, Commission assesses plastics strategy voluntary pledges, Link: https://chemicalwatch.com/74886/commission-assesses-plastics-strategy-voluntary-pledges?q=microPlastics - accessed 11-6-2019.

ChemicalWatch (CW), 2019m, NGO Platform: Trends in chemical management science and governance, Link: <https://chemicalwatch.com/74531/ngo-platform-trends-in-chemical-management-science-and-governance?q=microPlastics> - accessed 11-06-2019.

ChemicalWatch (CW), 2019o, NGO Platform: Guest Column: The 2019 chemical conundrum, Link: https://chemicalwatch.com/74486/guest-column-the-2019-chemical-conundrum?q=microPlastics - accessed 12-6-2019.

ChemicalWatch (CW), 2019u, Q&A on microplastics restriction, Link: <https://chemicalwatch.com/79566/echa-round-up?q=microPlastics> - accessed 8-8-2019.

ChemicalWatch (CW), 2019y, ECHA round-up on restriction proposals, Link: https://chemicalwatch.com/73454/echa-round-up?q=microPlastics - accessed 8-8-2019.

Chynoweth, E., 2013, Cosmetics industry sees EU regulation as a global driver, ChemicalWatch, Link: <https://chemicalwatch.com/15281/cosmetics-industry-sees-eu-regulation-as-a-global-driver?q=microplastic> – accessed 20-8-2019.

Crisp, J., 2014, Ex-Tory MP entangled in EU plastic bag lobbying row, EURACTIV, Link: <https://www.euractiv.com/section/sustainable-dev/news/ex-tory-mp-entangled-in-eu-plastic-bag-lobbying-row/> - accessed 28-10-2019

Davies, E., 2017, UN report says need for more data on microplastics in food fish, ChemicalWatch, Link: <https://chemicalwatch.com/53486/un-report-says-need-for-more-data-on-microplastics-in-food-fish?q=microplastic> – accessed 20-8-2019.

Davies, E., 2019, Echa sets up working group for deluge of EDC authorisation applications, ChemicalWatch Link: <https://chemicalwatch.com/75447/echa-sets-up-working-group-for-deluge-of-edc-authorisation-applications?q=microPlastics> - accessed 11-6-2019.

Ends, 2017a, Study calls for a eco-design approach to plastics pollution, Link: <https://www.endseurope.com/article/48508/study-calls-for-ecodesign-approach-to-plastic-pollution> - accessed 23-10-2019

Ends, 2017b, Nordic parliamentarians call for microplastics ban, Link: <https://www.endseurope.com/article/48260/nordic-parliamentarians-call-for-microplastics-ban> - accessed 23-10-2019

Ends, 2018a, ECHA to back microplastics restrictions, Link: <https://www.endseurope.com/article/54307/echa-to-back-microplastics-restrictions> - accessed 23-10-2019

Ends, 2018d, Plastics: MEPs call for wider bans, incentives to recycle, Link: <https://www.endseurope.com/article/53710/plastics-meps-call-for-wider-bans-incentives-to-recycle> - accessed 23-10-2019

Ends, 2018g, ECHA defends ‘objectivity’ of microplastics call, Link: <https://www.endseurope.com/article/52975/echa-defends-objectivity-of-microplastics-call> - accessed 23-10-2019

Ends, 2018j, Brussels to cut bottle use with new tap water rules, Link: <https://www.endseurope.com/article/51734/brussels-to-cut-bottle-use-with-new-tap-water-rules> - accessed 23-10-2019

Ends, 2018k, EU drinking water rules to tackle BPA, Link: [https://www.endseurope.com/article/51723/eu-drinking-water-rules-to-tackle-bpa - accessed 23-10-2019](https://www.endseurope.com/article/51723/eu-drinking-water-rules-to-tackle-bpa%20-%20accessed%2023-10-2019)

Ends, 2019a, No plan to close artificial turf pitches, says ECHA, Link: [https://www.endseurope.com/article/56119/no-plan-to-close-artificial-turf-pitches-says-echa - accessed 23-10-2019](https://www.endseurope.com/article/56119/no-plan-to-close-artificial-turf-pitches-says-echa%20-%20accessed%2023-10-2019)

Ends, 2019b, Textiles set to be next EU circular economy priority, Link: [https://www.endseurope.com/article/55500/textiles-set-to-be-next-eu-circular-economy-priority - accessed 23-10-2019](https://www.endseurope.com/article/55500/textiles-set-to-be-next-eu-circular-economy-priority%20-%20accessed%2023-10-2019)

Ends, 2019c, Fertiliser producers set to bear the brunt of microplastics restrictions, Link: [https://www.endseurope.com/article/55464/fertiliser-producers-set-to-bear-the-brunt-of-microplastics-restrictions - accessed 23-10-2019](https://www.endseurope.com/article/55464/fertiliser-producers-set-to-bear-the-brunt-of-microplastics-restrictions%20-%20accessed%2023-10-2019)

Ends, 2019e, Details of proposed EU microplastics ban revealed, Link: <https://www.endseurope.com/article/54656/details-of-proposed-eu-microplastics-ban-revealed> - accessed 23-10-2019

Ends, 2019f, Uncertain risk of microplastics pollution, scientists say, Link: <https://www.endseurope.com/article/54588/uncertain-risk-of-microplastics-pollution-scientists-say> - accessed 23-10-2019

EUObserver, 2017, EU unwilling to tax plastics, EUObserver, Link: https://euobserver.com/tickers/139319 - accessed 28-10-2019

Fortuna, G., 2019, Tap water rules no longer in deep water, but health concerns persist, EURACTIV, Link: <https://www.euractiv.com/section/health-consumers/news/tap-water-rules-no-longer-in-deep-water-but-health-concerns-persist/> - accessed 28-10-2019

Harvey, F., 2017, EU rules out tax on plastics products to reduce waste, EURACTIV, Link: <https://www.euractiv.com/section/circular-economy/news/eu-rules-out-tax-on-plastic-products-to-reduce-waste/> - accessed 28-10-2019

Ketelsen, M., 2018, How to reduce the impact of plastic products on the marine environment?, EURACTIV, Link: <https://www.euractiv.com/section/energy-environment/video/how-to-reduce-the-impact-of-plastic-products-on-the-marine-environment/> - accessed 28-10-2019

Morgan, S., 2018, Death-knell for microplastics?, EURACTIV, Link: <https://www.euractiv.com/section/energy-environment/news/death-knell-for-microplastics/> - accessed 28-10-2019

Morgan, S., 2018, MEPs vote to turn ‘plastic wastelands into fields of gold’, EURACTIV, Link: <https://www.euractiv.com/section/energy-environment/news/meps-vote-to-turn-plastic-wastelands-into-fields-of-gold/> - accessed 28-10-2019

Morgan, S., 2018, Commission maps out plastics vision in new strategy, EURACTIV, Link: <https://www.euractiv.com/section/energy-environment/news/commission-maps-out-plastics-vision-in-new-strategy/> - accessed 28-10-2019

Morgan, S., 2018, Karmenu Vella: ‘Well-designed’ plastics tax could help hit environment targets, EURACTIV, Link: <https://www.euractiv.com/section/circular-economy/interview/karmenu-vella-well-designed-plastics-tax-could-help-hit-environment-targets/> - accessed 28-10-2019

Oziel, C., 2018, NGOs attack Echa’s ‘limited’ microplastics restriction proposal, ChemicalWatch, Link: https://chemicalwatch-com.proxy.findit.dtu.dk/67582/ngos-attack-echas-limited-microplastics-restriction-proposal?q=microPlastics - accessed 14-8-2019.

Oziel, C., 2018, Oil and gas industry challenges EU estimate on microplastics use, ChemicalWatch, Link: https://chemicalwatch-com.proxy.findit.dtu.dk/67188/oil-and-gas-industry-challenges-eu-estimate-on-microplastics-use?q=microPlastics - accessed 14-8-2019.

Rios, B., 2019, EU will not ban artificial turf pitches but could impose restrictions, EURACTIV, Link: <https://www.euractiv.com/section/health-consumers/news/eu-will-not-ban-artificial-turf-pitches-but-could-impose-restrictions/> - accessed 28-10-2019

Santoni, O., 2019, Expert Focus: EU regulatory update on the status of cosmetic ingredients, ChemicalWatch, Link: https://chemicalwatch.com/78802/expert-focus-eu-regulatory-update-on-the-status-of-cosmetic-ingredients?q=microPlastics - accessed 8-8-2019.

Stringer, L., 2018, The European Parliament votes in support of a microplastics ban, ChemicalWatch, Link: https://chemicalwatch-com.proxy.findit.dtu.dk/70327/european-parliament-votes-in-favour-of-microplastics-ban?q=microPlastics - accessed 14-8-2019.

Tani, C., 2019, Echa, Commission allay fears of artificial turf closures after microplastic ban, ChemicalWatch, Link: https://chemicalwatch.com/80620/echa-commission-allay-fears-of-artificial-turf-closures-after-microplastic-ban?q=microPlastics - accessed 8-8-2019.

Valero, J. & Kirk, F., 2018, The Brief – Juncker’s plan for the elections? Results, EURACTIV, Link: <https://www.euractiv.com/section/politics/news/the-brief-junckers-plan-for-the-elections-results/> - accessed 28-10-2019

Zainzinger, V., 2017, Nordic Council considers ban on microbeads in cosmetics, ChemicalWatchLink: <https://chemicalwatch.com/52599/nordic-council-considers-ban-on-microbeads-in-cosmetics?q=microplastic> – accessed 20-8-2019.
