# Supplementary material for: Stakeholder analysis with regard to a recent European restriction proposal on microplastics
Source: PLoS One. 2020 Jun 22;15(6):e0235062. doi: 10.1371/journal.pone.0235062 (PMC7307934; doi:10.1371/journal.pone.0235062)
Supplement: S5 Table — (DOCX) [file pone.0235062.s006.docx]

S5 Table: International NGOs categorization table

| ***International NGOs*** | | | | | | |  |
| --- | --- | --- | --- | --- | --- | --- | --- |
| **No**. | **Stakeholder** | **Criteria**  (for statement e.g. economy, innovation, environment etc.) | **Principles**  (separate from criteria e.g. PP, values etc.p) | **Scientific argumentation**  Case Reports & Case Series (observational)  Case-control (observational)  Cohort (observational)  Randomized-controlled trials (experimental)  Systematic review | **Research needs**  (identified needs in statement) | **Other** |  |
| 1 | Beat the Microbead | A group of NGOs is putting pressure on the European Commission to introduce an EU ban on all microplastics in cosmetics and to take regulatory action on polymer ingredients. (Zainzinger, 2017).  The NGO “Beat the Microbead” has called for industry action to go beyond synthetic, solid particles used for exfoliating and cleansing (Oziel, 2018).  A group of NGOs is putting pressure on the European Commission to introduce an EU ban on all microplastics in cosmetics and to take regulatory action on polymer ingredients. (Zainzinger, 2017).  The Beat the Microbead coalition says in a position paper that the plastic pollution of waters by the cosmetics industry will not stop unless the Commission sets clear rules. (Zainzinger, 2017).  New EU regulation should ban all polymer ingredients that are persistent, bioaccumulative or toxic to ecosystems from being used in cosmetic products, the NGOs demand. (Zainzinger, 2017). |  |  |  |  |  |
| 2 | ChemSec |  |  |  | ChemSec supports the dossier submitter’s conclusion that the risks arising from the releases to the environment of intentionally used microplastics are not adequately controlled. We therefore support the need for a restriction.  ChemSec supports the restriction by providing evidence on:  - Available alternatives  - Research on alternatives  - List of microplastics/polymers identified in hygiene and cosmetic products |  |  |
| 2/3 | ChemSec & Beuc | ChemSec and European consumer organisation Beuc are urging the European Commission to implement bans on several substances used in detergents. (Buxton, 2017). |  |  |  |  |  |
| 4 | CIEL (Centre for International Environment Law) |  |  | David Azoulay - Centre for International Environmental Law (Ciel); Priscilla Villa – Earthworks; Yvette Arellano - Texas Environmental Justice Advocacy Services (Tejas); Miriam Gordon - UPSTREAM; Doun Moon - Global Alliance for Incinerator Alternatives (GAIA); and Kathryn Miller and Kristen Thompson - Exeter University, publish the report “Plastic and Health: The hidden cost of a plastic planet,” in Feb. 2019. The report conclude that the "current narrow approaches to assessing and addressing plastic impacts" as "inadequate and inappropriate" and that plastic should be recognized as a POP (Stringer, 2019). |  |  |  |
| 5 | ClientEarth | ClientEarth welcomes this Restriction and, in particular, its scope which covers all sources of intentionally added microplastics, irrespective of the sector or specific use. This inclusive scope is justified considering the breadth of the environmental disaster. However, the derogations and transitional periods raise concerns as our contribution highlights. |  |  |  |  |  |
| 6 | Cousteau Divers | Pierre-Yves Cousteau, CEO, Cousteau Divers: “The slogan Reduce – Reuse – Recycle is currently broken, as re-used and recycled materials continue to release invisible plastic particles that we breathe in and eat. You and I, in our daily lives, can already have an impact by refusing single-use plastic.” (Cousteau, 2017) |  |  |  |  |  |
| 7 | Earthwatch Europe |  |  | **Comment:**  Earthwatch Europe have published a report with the support of Eunomia, which looks at the risks to businesses of microplastic pollution across a number of sectors. Artificial sports turf, equestrian facilities and playgrounds is one of the sectors covered (4.3, page 17). Other sectors we cover in the report that are of relevance to the consultation on intentionally added microplastics include on plastic pellets (nurdles) (4.1, page 14); other undiscovered microplastic sources (4.6, page 23). Please see attached report for details.  **Answer to specific info request 2:**  Earthwatch's report, attcached, provides information on tonnage of infill released into the environment in the UK (1-5 tonnes of infill per pitch per year) and examples of best practice operational measures that can be taken to reduce or eliminate this, which could be implemented voluntarily or be made a requirement through regulatory action. These include:  1) use of natural (organic) infill  2) appropriate inside storage for infill used for top-ups  3) handling procedures to reduce loss when moving infill around  4) changing room cleaning procedures such as the correct disposal of infill when cleaned up  5) filters in drains in changing rooms and in local rain water drains, including regular emptying  6) player education and designated ‘shake-off’ zones where infill is removed from clothing  Further information is available in the attached report. |  |  |  |
| 8 | ECOS | a)  ECOS urges for a complete ban instead of a concentration limit of 0.01% w/w. This concentration is too high, allowing millions of particles being added to products with the potential to end up in the environment. To really end microplastics pollution and give a clear signal to the market, we therefore propose a complete ban. This would push industries to search for natural solutions, that have already existed before the introduction of microplastics.  In our view, exemptions from ban are only justified for naturally occurring polymers (e.g. cotton, rubber, wool, etc.,) and for microplastics that are essential and irreplaceable for vital medicinal products or research purposes (e.g. stem-cell research, material science and biomedical applications to micro- and nanoresearch, coulter counter validation methods, environmental monitoring of micro and nanoplastics, recovery rates of micro- and nanoplastics in laboratory experiments, etc).  Other applications that consider the use of microplastics (e.g. personal care products, film-forming paints and coatings) should be banned, as there is not enough evidence that leakage into the environment can be prevented.  Medical waste is a specific type of waste which should follow specific health and safety regulations, which at the moment, not all EU countries have such regulations by the fact that there is no classification for medical waste. Some countries impose mandatory incineration of infectious waste, as well as and security regulations. According to the Polish Zero Waste Association (PZWA) (https://noharm-europe.org/sites/default/files/documents-files/4793/13-Gluszynski.pdf) there is a lack of dedicated regulation and overriding policies concerning this type of waste. According to PZWA “there are different regulations in members states, including waste classification and requirements for disposal, as not all countries have developed guidelines for this sector”. “Some countries impose mandatory incineration of infectious waste, as well as non-risk waste if it is sent to recycling”.  Based in the PZWA document and the EU HCWM (http://www.hcwm.eu/) website, it is still needed to define an EU standardised approach to Healthcare Waste Management.  Our position is that medical waste should be considered hazardous waste and should follow health and safety regulations and guidelines for proper disposal. |  | ECOS encourages that in Chapter 1.2.1 “General considerations” it is clearly specified that “The terms plastic or plastics do not have a precise meaning because they reflect rather complex formulated systems whose exact composition is generally unknown”. However, this aspect is not considered in Chapter 2.2.1.6 “(Bio)degradability criteria”. The criteria in order to demonstrate the (bio)degradability of microplastics do not consider the fact that a biodegradable microplastic can be a mixture of different biodegradable polymers and additives (for example: 65% “Polymer A”, 15% “Polymer B”, 18% “Polymer C”, 1% “Additive A” and 1% “Additive B”).  The criteria to evaluate (1) ready biodegradation (i.e. 60% mineralization after 28 days), (2) enhanced/modified ready biodegradation (i.e. 60% mineralization after 60 days) and (3) inherent biodegradation (70% mineralization) are identical to criteria used for pure chemicals. A microplastic containing 85% “biodegradable polymer” and 15% “conventional not-biodegradable polymer” might easily reach the 60% or 70% pass level. Consequently, with the current proposed pass levels, it cannot be guaranteed that not-biodegradable polymers will not be released in the environment.  In order to make the criteria to evaluate ready biodegradation, enhanced/modified ready biodegradation and inherent biodegradation more stringent, it would be good to look to the historical evolution of the biodegradation criteria in the field of (industrial and home) compostable plastics and packaging. The first version of the American standard specification for compostable plastics (ASTM D6400 (1999)) required that for products consisting of a single polymer (homopolymers) at least 60% of the organic carbon must be converted to carbon dioxide when compared to the positive control after 180 days under controlled composting conditions, while for products consisting of more than one polymer, a higher pass level of 90% was applied. Due to the fact that practice showed that it was difficult to determine if a polymer is a homopolymer or not, this lower pass level for homopolymers was removed when the standard specification was revised. In ASTM D6400 (2012) only the 90% pass level is mentioned. Moreover, the standard specification has become even more stringent by requiring that all constituents which are present in the product in a concentration between 1% and 10% need to be evaluated separately on biodegradation. This rule was implemented in order to avoid that products containing 95% “biodegradable polymer” and 5% “not-biodegradable polymer” are still able to claim that they are compostable (as they can theoretically reach the 90% pass level). Both (1) the 90% biodegradation pass level and (2) the rule that constituents which are present in a concentration between 1% and 10% need to be evaluated separately on biodegradation are now applied in the international standard specifications for industrial compostable products (ISO 17088 Specifications for compostable plastics (2012) and ISO 18606 Packaging and the environment - Organic recycling (2013)). In a draft version of a European standard specification for home compostable carrier bags, the rule that constituents between 1% and 10% need to be evaluated separately on biodegradation is even made more stringent by requiring that constituents between 1% and 15% need to be evaluated separately on biodegradation.  Considering this information, ECOS suggests to make the biodegradation criteria to evaluate ready biodegradation, enhanced/modified ready biodegradation and inherent biodegradation more stringent by referring to higher pass levels (90% relative biodegradation when compared to a suitable reference material) for these tests and by also requiring also that constituents present in a concentration between 1% and 15% in the final microplastic are evaluated separately on biodegradation.  Furthermore, (4) the biodegradation requirement relative to a reference material could be made more stringent by requiring that constituents present in a concentration between 1% and 15% in the final microplastic are evaluated separately on biodegradation.  Moreover, in this requirement (4), it is only allowed to reach at least 90% biodegradation relative to a suitable positive reference material. Alternatively, it could also be allowed to reach at least 90% absolute biodegradation. This is currently also allowed by following standard specifications:  Specifications for compostable products:  - EN 13432 Requirements for packaging recoverable through composting and biodegradation - Test scheme and evaluation criteria for the final acceptance of packaging (2000)  - NF T51-800 Plastics - Specifications for plastics suitable for home composting (2015)  - AS 4736 Biodegradable plastics – Biodegradable plastics suitable for composting and other microbial treatment (2006)  - ASTM D6400 Standard Specification for Labeling of Plastics Designed to be Aerobically Composted in Municipal or Industrial Facilities (2012)  - ASTM D6868 Standard Specification for Labeling of End Items that Incorporate Plastics and Polymers as Coatings or Additives with Paper and Other Substrates Designed to be Aerobically Composted in Municipal or Industrial Facilities (2017)  - ISO 17088 Specifications for compostable plastics (2012)  - ISO 18606 Packaging and the environment - Organic recycling (2013)  Specification for products biodegradable in soil:  - EN 17033 Plastics - Biodegradable mulch films for use in agriculture and horticulture - Requirements and test methods (2018)  Specification for products suitable for waste water treatment plants:   EN 14987 Plastics - Evaluation of disposability in waste water treatment plants - Test scheme for final acceptance and specifications (2006)  Four test methods are mentioned in requirement (4): EN ISO 14851 and EN ISO 14852 (freshwater inoculum), EN ISO 18830 (seawater/sediment inoculum) and ISO 17556 (soil inoculum). Additionally, it would be useful to refer to a biodegradation test method in a pelagic seawater environment. At this moment no EN ISO test methods are developed, but the following American test method could be used as reference: ASTM D6691 Standard Test Method for Determining Aerobic Biodegradation of Plastic Materials in the Marine Environment by a Defined Microbial Consortium or Natural Sea Water Inoculum (2009).  In order to demonstrate (bio)degradability using higher tier assessment, the degradation half-life (= the time taken for 50% transformation of a test substance when the transformation can be described by first-order kinetics) needs to be less than 60 days (marine, fresh or estuarine water) or 180 days (marine, fresh or estuarine sediment or soil). The test methods in order to determine the degradation half-life (OECD TG 307, OECD TG 308 and OECD TG 309) are designed for pure chemicals, but not for microplastics, which might be a mixture of several polymers and additives. If 50% transformation is reached for a microplastic containing different polymers, this could imply that only 1 of the polymers is degraded. In order to avoid that not-biodegradable constituents are present in a microplastic, the half-life of each constituent of the microplastic should be determined as part of higher tier biodegradability assessments.  There are some studies who have been focusing on the quantity of microplastics released to the environment and on their different pathways of release to the environment. Regarding the pathways, they might result from agriculture, washing machines, consumer products, medical applications, urban and transport infrastructures, intentional shredding and fragmentation, fragmentation by physical and chemical weathering; handling during manufacturing or maintenance, etc. as described in the figure (http://www.grida.no/resources/6929) who is based in GESAMP reports and several reliable scientific publications. Considering that plastic production is following an exponential projection (http://www.grida.no/resources/6923) and that there is a cumulative effect in the environment the real scale of the problem is not well understood. Regarding still pathways, it is important to understand how plastic move form the economy to the environment (http://www.grida.no/resources/6908).  A study by Napper and Thompson (2016) focused on the release of synthetic microplastic plastic fibers from domestic washing machines, identified that an average wash load of 6kg, could potentially release between 138,000 and over 700,000 fibers per wash https://doi.org/10.1016/j.marpolbul.2016.09.025). The study focused in 3 types of fibers (polyester-cotton blend, polyester and acrylic). Since fibers are the most common microplastic found worldwide, this is of relevance, and about 35% of microplastics released into the world’s oceans are from synthetic textiles (https://phys.org/news/2018-09-microplastics-world-oceans-synthetic-textiles.html and also downloadable report with this article).  Another relevant study was conducted by Jambeck et al., 2015 (https://science.sciencemag.org/content/347/6223/768/tab-pdf DOI: 10.1126/science.1260352), whose title is plastic waste inputs from land into the ocean. This study published in Science is the scientific background to the common sentence “by 2050, there will be more plastic than fish in the ocean” that the report from Ellen McArthur Foundation popularized. The study calculated that 275 million metric tons (MT) of plastic waste was generated in 192 coastal countries in 2010, with 4.8 to 12.7 million MT entering the ocean. Although the study refers to plastic of different sizes, eventually even the larger pieces will fragment into smaller pieces of microscopic or potentially nanoscopic size.  Regarding quantities of microplastics being released to the environment, there are 2 studies from Lebreton et al., that are particularly relevant. One shows evidence that the Great Pacific Garbage Patch is rapidly accumulating plastic, and the second one identifies the river plastic emissions to the world’s oceans (https://www.nature.com/articles/ncomms15611). The first study predicted at least 79 (45–129) thousand tonnes of ocean plastic are floating inside an area of 1.6 million km2; a figure four to sixteen times higher than previously reported for this region. Microplastics accounted for 8% of the total mass but 94% of the estimated 1.8 (1.1–3.6) trillion pieces floating in the area.  The second study estimated that between 1.15 and 2.41 million tonnes of plastic waste currently enters the ocean every year from rivers, with over 74% of emissions occurring between May and October. The top 20 polluting rivers, mostly located in Asia, account for 67% of the global total. The following infographic shows the plastic input into the oceans based on these studies (http://www.grida.no/resources/6906).  In order to evaluate the importance of different pathways, and input release contribution to the environment, it is important to estimate and report similar units to allow comparison among studies. The JPI-Oceans BASEMAN project, cited in the ECHA restriction proposal and the Annex to the Annex supporting documents, has prepared the 3 reports (sediment – DOI 10.13140/RG.2.2.36256.89601/1; seawater – DOI: 10.13140/RG.2.2.14181.45282 and biota – DOI 10.13140/RG.2.2.28588.72321/1; who propose as reporting units:  1. no. MPs per area (# particles km-2 \| # particles m-2)  2. no. MPs per volume (# particles m-3)  3. mass of MP per area (g MP km-2 \| g MP m-2)  4. mass of MP per volume (g MP L-1 \| g MP m-3)  The BASEMAN protocol whose title is “Harmonized protocol for monitoring microplastics in biota” includes even more detailed information about how to report units in section 3. Because plastic is a lightweight material, it is important to have a quantification of microplastics released to the environment in tonnes/yr but also include an estimation of the amount in number and volume of particles released into the environment.  b)  The 2017 Report on Intentionally added microplastics in products (http://ec.europa.eu/environment/chemicals/reach/pdf/39168%20Intentionally%20added%20microplastics%20-%20Final%20report%2020171020.pdf) produced by the Environmental Agency of Austria, Amec Foster Wheeler and Peter Fisk Associates, describes that “According to the Danish Environmental Protection Agency (2015)102, expanded polystyrene (EPS) is used in construction (wall insulation), packaging, furniture (beanbags), pillows and a few other unspecified applications. GESAMP (2015)103, furthermore mentions the use of EPS in cool boxes, floats and cups, as well as buoys for mariculture/aquaculture.”  The PlasticsEurope Facts report 2018, page 23, also illustrates that among the total converter demand main market sector, “plastic furniture and furniture equipment” are included in other important market sectors which represent 16% of the total in Europe (https://www.plasticseurope.org/en/resources/publications/619-plastics-facts-2018).  c)  There are several different analytical methods to detect and quantify microplastics in the products mentioned, such as micro-RAMAN, micro-FTIR, FTIR-ATR, Py-GC/MS, Hyperspectral Imaging, optical microscopy, Scanning electron microscopy (SEM). Most of these techniques and methodologies are cutting-edge and require trained experts to operate them. Some of the techniques are destructive and therefore their use should also include non destructive techniques to quantify microplastics.  Micro-RAMAN can identify particles as small as 1 µm (micrometer), while modern micro-FTIR devices can reach 10 µm. To date, environmental monitoring results are usually reported to ≥100 µm, (BASEMAN reports above), and by recommendation of the Marine Strategy Framework Directive on descriptor 10 – Marine Litter, the recommendations ≥300 µm (Frias and Nash, 2019). FTIR-ATR is used for particles which are on average 1 mm in size, although for some devices, it can go to a lower size limit.  Attempts to identify the polymer type using physical characteristics such as particle colour, type and shape, by visual inspection using optical microscopy provide about 40% of false positive results. Py GC/MS is a destructive technique and it would need to be coupled to a FTIR system to have a full characterisation and quantification in number. Py-GC/MS will quantify microplastics in mass and therefore should not be used as a quantifying technique.  Staining techniques can also be used for microplastic identification, such as rose Bengal or Nile red. Nile red pigments the polymeric material while rose Bengal works by coating or colouring it. In both cases, open source software (Galaxy count or ImageJ) can be used for counting particles. It is important to address the fact that some pigments and dyes need to be in solution form, and in the case of Nile red, usually acetone, (CH3)2CO, CAS no. 67-64-1, is used. This solvent is known to contribute to the degradation of certain polymers (e.g. polystyrene), therefore causing identification artefacts.  SEM can be used for the purpose of surface characterization. SEM-EDS can provide information on the surface of the plastic and on elemental composition. SEC-HPLC can also be used for size exclusion separation, but information on the polymer composition is only possible when using the MS detector. All these methods are similar to Py-GC/MS which should be not be used by itself and therefore not recommended.  Sampling microplastics in all product categories, would require using specific protocols depending on the environmental matrix (water, sediment, biota) where the samples are found in the environment.  Collection and processing of environmental samples might require filtration and/or digestion of organic/biologic tissues, before optical inspection. Processing should not have any step with temperatures higher than 40ºC and it should use the minimum amount of chemical substances as possible, as these might interfere with the polymers itself (BASEMAN reports). All methods require testing, optimization, inter-calibration and standardizing. More work is still needed to develop analytical procedures and protocols that can widely be used while sample collecting, processing and identification before analysis.  The PlasticsEurope Facts report 2018, page 23, also illustrates that among the Total converter demand main market sector, “plastic furniture and furniture equipment” are included in other important market sectors which represent 16% of the total in Europe (https://www.plasticseurope.org/en/resources/publications/619-plastics-facts-2018). The study on the EU furniture market situation and a possible furniture products initiative (http://ec.europa.eu/DocsRoom/documents/7572/attachments/1/translations), identifies in page 71, that plastics represent 9% of the share of materials used in furniture production. Table 83 of the same document states under materials used that “plastics and metal shall be allowed in a percentage up to 2% of the total weight of the piece of furniture” as a current requirement to have an EU Ecolabel.  A study from Shiri, Krafft and Thurm, (https://doi.org/10.1063/1.5092935), describes the process on how plastic waste is washed and shredded to be then “intentionally added” to furniture. The paper shows all stages of plastic lumber product development using commingled waste plastics.  A 2014 report exploring the EU furniture market situation and a possible furniture products initiative (https://www.ceps.eu/publications/eu-furniture-market-situation-and-possible-furniture-products-initiative) shows that “plastics, bamboo, rattan cane, glass and other materials accounted for 11% of main materials used in 2010 in EU28”. Plastics alone represent 9% of the share of materials used in furniture production (by value). The report mentions “In terms of materials used, there is a trend away from the use of wood in EU office furniture production towards plastic and metal, although wood still accounts for important shares of production (80% in office desks, 30% in worktops, 50% in cabinets, storage and filing system and wall to wall units)”.  Although none of the reports clearly specifies the size range of the plastic polymers used, since they are using shredding machines to incorporate plastics into furniture, it seems to us that microplastics are being intentionally added to those products.  Therefore, this sector should be included, as microplastics are intentionally added as a technical function on furniture, and they might potentially release microplastics into the environment. The (http://eippcb.jrc.ec.europa.eu/reference/BREF/stm_bref_0806.pdf)  To date and to our current knowledge there are no studies that have quantified the potential amount of microplastics that might be released into the environment. Nonetheless, this sector should be included in this ECHA proposal, as it has been highlighted by Plastics Europe in the Facts report 2018 as an important demand sector. | Input on ANNEX TO THE ANNEX XV RESTRICTION REPORT – MICROPLASTICS  Following items could be improved in this annex:  - Page 25: Pass or fail criteria for biodegradability are also established for plastics suitable for a waste water treatment plant (EN 14987 Plastics - Evaluation of disposability in waste water treatment plants - Test scheme for final acceptance and specifications (2006))  - Page 25 (bullet point 1): Besides a 90% pass level relative to the positive reference material, also an absolute 90% pass level is allowed by the standard specifications for compostable materials.  - Page 26 (fourth paragraph): Information is given about the Biodegradable WATER certification scheme. It is the OK biodegradable WATER certification scheme. It could be added that this certification scheme is based on a European standard specification (EN 14987 Plastics - Evaluation of disposability in waste water treatment plants - Test scheme for final acceptance and specifications (2006))  - Page 26 (Table 3): When referring to inherent biodegradation it is mentioned that the log phase should be no longer than 3 days. Log phase is a term which is not used in biodegradation test methods. Is it possible that this is a typo and that this should be replaced by lag phase? A lag phase is defined as the period from inoculation in a die-away test until the degradation percentage has increased to about 10%.  Table 88 include an extensive list of polymers intentionally added to products as described in the text above, therefore it is not clear to ECOS the reason why only 19 polymers are mentioned here. Also, in terms of consistency of definition in the ECHA proposal, the definition of microplastics here, should be the same as the one stressed in point 1. It is unclear the reason why only 19 polymers are mentioned, when PVC and other relevant polymers are mentioned in the list in table 88. |  |  |
| 9 | European Environmental Bureau (EEB) | The EEB supports the dossier submitter’s conclusion according to which the risks arising from the releases to the environment of intentionally used microplastics are not adequately controlled. We therefore support the need for a restriction.  We fully support the Dossier Submitter’s conclusion that intentionally added microplastics should be treated as non-threshold substances. As stated at the Restriction proposal, the extreme persistence in the environment of micro and nano plastics leads to an increasing and irreversible environmental stock, with unpredictable negative consequences to our ecosystems, warranting the need for regulatory action to reduce environmental releases of microplastics to the environment.  The EEB would like to contribute to support the restriction by:  - Providing recent scientific evidence on:  - Adverse ecotoxicological effects and trophic transfer  - Risks of nanoplastics resulting from the degradation/ transformation of microplastics  - Biomagnification and bioaccumulation  - Synergetic effects with other environmental pollutants  - Exposure of European ecosystems  - Providing comments on the need to expand the scope to microbeads used in synthetic turf  - Providing comments on the need to improve the proposed biodegradation criteria.  Further, we would like to stress the need for ECHA’s Committees to assess, in their opinions, if the proposed derogations are properly justified and supported by scientific evidence and their impacts on the environment have been properly quantified.  Existing biodegradability criteria today should all be improved to be more ambitious and present no adverse environmental effects. Since there is no sufficiently ambitious standard available today, ECHA should take a precautionary approach and not include biodegradable polymers as an exemption.  If biodegradable polymers are finally exempted, this exemption should cover only products that have a low likelihood of entering other environmental compartments and the proposed criteria should be improved according to the considerations outlined in ECOS’ submission to this public consultation.  Please see the attachement for further information on this and other issues  we would like to stress the need for ECHA’s Committees to assess, in their opinions, if the proposed derogations are properly justified and supported by scientific evidence and their impacts on the environment have been properly quantified. | ECHA should take a precautionary approach and be consistent: a so-called biodegradable polymer/polymer mixture that is added to a product is still a plastic. There are naturally occurring alternatives for most functions performed by plastics in products (e.g. scrub in the scrubbing agents could very well be clay, why would it need to be plastic?).  Examples of ‘best practice’ operational conditions (OCs) and risk management measures (RMMs) to prevent or minimise the release of infill material to the environment, including an estimate of their effectiveness.  The report by Hans et al (2018) highlights that although the majority of the market uses rubber crumb from recycled tyres—often referred to simply as SBR (styrene-butadiene rubber), performance infill can be made from organic alternatives such as cork and coconut husk, which are available in the EU market.  A report by the Earthwatch Institute (2019) offers guidance on key actions to reduce losses of microplastics used as turf infill, including:  • Install or plan appropriate measures to adequately mitigate release of infill into the environment. Organic infill such as cork, if appropriate for the installation, will completely mitigate the microplastics issue for artificial turf.  • Investigate infill loss mitigation measures that can be built in from the beginning:  ◦ appropriate inside storage for infill used for top-ups  ◦ handling procedures to reduce loss when moving infill around  ◦ changing room cleaning procedures such as the correct disposal of infill when cleaned up  ◦ filters in drains in changing rooms and in local rainwater drains, including regular emptying  ◦ player education and designated ‘shake-off’ zones where infill is removed from clothing  ◦ special attention should be paid to these sites including buffers to prevent migration of the infill by wind and rain towards water courses (<50M).  Earthwatch Institute (2019) MICROPLASTICS: HOW SHOULD BUSINESS RESPOND? https://microplastics.earthwatch.org.uk/  Please see the attachement for further information on this and other issues | Available scientific evidence shows also the widespread presence of microplastics in terrestrial and aquatic environments, in biota, in rainwater, air and snow in Europe.  There is evidence that at present, microplastics represent a high risk to the environment in several locations.  Available industry data shows increasing trends in plastics’ (and microplastics’) production and use in Europe that will result in the inevitable release of microplastics to the environment, despite the future potential adoption of risk management measures for some uses.  Existing biodegradability criteria in standards and test methods do not work for different environmental matrixes. A polymer that is soil biodegradable, will not necessarily biodegrade in other environmental matrixes such as water, or may persist much longer and biodegrade (if at all) after a much longer time.  The quantity of microplastics used as synthetic turf infill material in individual Member States or the EU/EEA (Tonnes/yr). Hanns et al. (2018) Investigating options for reducing releases in the aquatic environment of microplastics emitted by (but not intentionally added in) products. Final Report. ICF and Eunomia. https://www.eunomia.co.uk/reports-tools/investigating-options-for-reducing-releases-in-the-aquatic-environment-of-microplastics-emitted-by-products/)  The report by Hans et al (2018) estimates that a total of 51,616 pitches exist in Europe with an installed area of 112 million square meters. Using the infill density of 16.1 kg/m2 the total infill estimated to be installed in Europe is 1.8 million tonnes.  The quantity of microplastics released to the environment (Tonnes/yr, all relevant compartments), and an assessment of the different pathways by which microplastics can be released into the environment and an evaluation of their relative importance.  The report by Hans et al (2018) estimates the infill loss to the total installed infill in Europe between 18,000 and 72,000 tonnes per year. Polymeric infill from artificial sports turf can be inadvertently removed by players (when attached to their clothing or footwear), and also through maintenance activities such as snow clearance in some countries. It may then enter drains, soil, or surface water, or be removed as part of waste collection. | Although scientific uncertainties remain regarding the impacts of microplastics to ecosystems and human health, the existing scientific evidence already shows toxic effects on a wide range of organisms along food chains, as well as trophic transfer and bioaccumulation.  Existing biodegradability criteria today should ALL be improved to be more ambitious and present no adverse environmental effects. Since there is no sufficiently ambitious standard available today, ECHA should take a precautionary approach and not include biodegradable polymers as an exemption. |  |  |
| 9/5/3/11 | European Environmental Bureau, ClientEarth, ChemSec, and Greenpeace | “*ClientEarth lawyer Alice Bernard said Echa's priority should be to ensure no risks are overlooked or underestimated. "Yet we witnessed a call for evidence with an excessive focus on protecting the interests of the businesses responsible for this microplastic pollution."*” (Oziel, 2018).  “*The "long-overdue" restriction proposal, the NGOs said, will exclude "relevant and harmful uses of microplastics" before the Committees for Risk Assessment and Socio-economic Analysis (Rac and Seac) give their opinion*” (Oziel, 2018). |  |  | The campaigners had said the call focused too much on asking industry to submit information to exclude certain uses from restrictions, and “did not show interest in receiving information from other stakeholders about the hazards and risks of microplastics”. (European Environmental Bureau, ClientEarth, ChemSec, and Greenpeace). (ends 2018g)  The campaigners say that while the agency invited industries using microplastics to provide information so it could exclude certain uses from restrictions, it “did not show interest in receiving information from other stakeholders about the hazards and risks of microplastics”.  The agency showed an “excessive focus on protecting the interests of the industry”, the campaigners said. (Ends 2018h) | In June, several NGOs criticised Echa for:  "[Unduly limiting](https://chemicalwatch-com.proxy.findit.dtu.dk/67582)" the scope of the proposal by having taken into account industry concerns before putting the proposal before its scientific committees (Oziel, 2018).  ECHA "did not show interest" in receiving information from non-industry stakeholders about the hazards and risks of microplastics (Oziel, 2018).  ECHA lacked the objectivity required by its role in the restriction process and misinterpreted the conditions that may justify the addition of derogations to the restriction (Oziel, 2018). |  |
| 10 | Fauna & Flora International |  |  | Please see our general comments in the attached consultation response.  **Content:**  Scope or restriction option analysis;  Hazard or exposure;  Environmental emissions;  Information on alternatives;  Information on costs;  Information on benefits;  Other socio economic analysis (SEA) issues;  Transitional period;  Request for exemption |  |  |  |
| 11 | Greenpeace | Represented by Melissa Wang, discusses the latest trends in chemical management science and highlights the issue of plastic pollution (CW, 2019m).  Greenpeace warned about the industry's use of large quantities of polyester and its contribution to pollution of the oceans with microplastic fibres (Lovell, 2018).  *"There's no single bad player; the industry as a whole is failing to regulate the use of microplastics [in] everyday products,"* says Taehyun Park, oceans campaigner at Greenpeace East Asia. *"The only sure and efficient way to successfully make sure that microplastics [are removed] from consumer products is to ban them."* (Franklin, 2016). |  | A study by Greenpeace, June 21 2018, in the Antarctic, has revealed the presence of microplastics and per- and polyfluorinated alkylated substances (PFASs) in most of the seawater and snow (CE, 2018l). |  |  |  |
| 12 | New Economics Foundation & Zero Waste Europe |  |  |  | David Powell, head of Environment & Green Transition at the New Economics Foundation & Ariadna Rodrigo is Sustainable Products Campaigner at Zero Waste Europe:  “none of the proposals or legislation tabled comes close to addressing the depth of our reliance on plastic. As argued i[n our new report published today](http://zerowasteeurope.eu/wp-content/uploads/2018/09/PlasticsTax_FINAL.pdf), we urgently need to look at the potential for new taxes to change behaviour and shift economic incentives for industry and consumers alike” (Rodrigo & Powell, 2018) |  |  |
| 13 | Plastic Soup Foundation | Alternatives are widely available. Our 'Zero Plastic Inside’ Brands (currently 65 brands) that promised to be 100% plastic free and showed us all their ingredients make clear it is possible, see also https://www.beatthemicrobead.org/look-for-the-zero/.  One of the major socio-economic impacts we expect are the additional costs for the European society to clean-up microplastics that leak into the environment. Especially the gigantic costs of cleaning up (water)soils polluted with microplastics is concerning. Intentionally added microplastics are likely to accumulate in terrestrial and aquatic environments. They can be extremely persistent, last for thousands of years and are practically impossible to remove. Concentrations in hotspots like some coastal areas already exceed tentative effect thresholds. Microplastics should therefore be restricted to minimize release as the current situation is not adequately controlled.  The transition period of 4-years and 6-years for rinse-off and leave-on cosmetics respectively, is much too long. Alternatives to microplastic ingredients are widely available on the market. More than 60 brands under our ‘Zero Plastic Inside’ certification prove that it is possible to make quality products without adding microplastic ingredients.  We regret the exclusion of semi-solid and liquid polymers and a lower size limit in the definition of microplastics of 1 nanometer. Especially, because the physical and chemical properties of these substances are increasingly considered as a risk to marine and terrestrial life, including humans.  In the Beat the Microbead database (https://www.beatthemicrobead.org/product-lists/) over 11,000 products are collected and the ingredients are checked on the presence of microplastics. For many polymers(ingredients) it is was not clear if they should be considered as (micro)plastic. The best available references were at that time from UNEP and TAUW commissioned by the Environmental ministry of Belgium. Many polymer substances were not on these lists, therefore we asked companies to send us the Product Data Safety Sheets. Remarkably many substances lack any information concerning persistency, bioaccumulating and toxicity properties.  We added available product safety sheets we received from several companies and suppliers as an attachment. |  | The scope of the restriction option should cover the current hiatus that exists for polymers within REACH. We don't see any reason why polymers, and in particularly plastics, should be treated different than any other chemical substance. Plastics are very persistent (P), bioaccumulate (B) in the environment and are related with toxic (T) effects. The PBT-criteria should be applied as the baseline. The risk of intentionally added microplastics leaking into the environment is severe, copious amount of plastic leak into the environment. For example, in only 1 anti-wrinkle crème of 50ml (Olaz) we found 1.5 million microplastics with a diameter range of 1.6 to 103 micrometre (see attachment OLAZ). These particles are so small they can actually enter organs once they enter an organism (see attachment Barboza et al. 2018). Furthermore, these particles are so small, they cannot even be detected yet in the environment (see attachment Microplastics in fisheries and aquaculture, FAO 2017).  page V: “Microplastic contamination of aquatic environments will continue to increase in the foreseeable future and at present there are significant knowledge gaps on the occurrence in aquatic environments and organisms of the smaller sized microplastics (less than 150 μm), and their possible effects on seafood safety. Currently there are no methods available for the observation and quantification of nanoplastics in aquatic environments and organisms”.  Sludge from sewer treatment plants is used in many EU countries on agricultural land. This contains copious amounts of microplastics. This should be considered as an intentionally added microplastics. The same is true for compost from food waste from households and companies (organic waste) that can be bought on the market and is used by consumers and farmers. A recent study in the Netherlands revealed this problem is much bigger than previously assumed. In the Dutch Parliament questions were asked to the Minister of Environment.  In Dutch, but the images give a good perspective of the problem of microplastics in compost: https://www.nhnieuws.nl/nieuws/245470/gemeenten-geven-vervuilde-compost-gratis-weg-aan-inwoners  See also the attachment Olaz wrinkle cream which contains 1,48 million polyethylene microplastics in a 50ml jar.  Copious amounts of microplastics are intentionally added to sewer treatment plants. In recent events these so called “bio-beads” washed on the Dutch coastline in huge amounts. See also:  https://www.wur.nl/en/Research-Results/Research-Institutes/marine-research/show-marine/Again-biobeads-on-the-Dutch-coast.htm  And see the attachment of Turner at al. 2019. |  |  |  |
| 14 | Rethink Plastics | It follows a call for action on microplastics by NGO umbrella group Rethink Plastics. Last month, the group [urged](https://chemicalwatch.com/60212/ngos-urge-tighter-regulations-for-microplastics) the Commission to implement immediate legislative measures to reduce microplastic pollution at the source. (CW, 2017b)  NGO umbrella group Rethink Plastics has called on the European Commission to implement immediate legislative measures to reduce microplastic pollution at the source. (CW, 2017c) |  |  |  |  |  |
| 15 | Seas at Risk |  |  |  | Emma Priestland, Seas at Risk: “Those that are added intentionally would have to be registered under REACH for the first time. For those that are generated, the Commission is examining options such as labelling and minimum requirements for product design and durability” (Keating, 2018) |  |  |
| 16 | (UEFA) UNION OF EUROPEAN FOOTBALL ASSOCIATIONS | Scope or restriction option analysis;  Information on alternatives;  Information on costs;  Other socio economic analysis (SEA) issues;  Transitional period |  |  |  |  |  |
| 17 | WFF European Policy Office | Andreas Baumüller, Head of Natural Resources at the WWF European Policy Office: *“Healthy rivers, lakes and wetlands are our life support system, but EU member states are trying to destroy the law that protects them and the window to save them is closing”* (Baumüller, 2018) |  |  |  |  |  |

**References**

Baumüller, A., 2018, Healthy rivers and lakes are not ‘nice-to-haves’, they are essential to our existence, EURACTIV, Link: <https://www.euractiv.com/section/energy-environment/opinion/healthy-rivers-and-lakes-are-not-nice-to-haves-they-are-essential-to-our-existence/> - accessed 28-10-2019

Buxton, L., 2017, NGOs urge EU ban on substances in detergents, ChemicalWatch, Link: <https://chemicalwatch.com/58350/ngos-urge-eu-ban-on-substances-in-detergents?q=microplastic> – accessed 20-8-2019.

ChemicalWatch (CW), 2017c, NGOs urge tighter regulations for microplastics, Link: <https://chemicalwatch.com/60212/ngos-urge-tighter-regulations-for-microplastics?q=microplastic> – accessed 20-8-2019.

ChemicalWatch (CW), 2018i, Norway's EPA proposes artificial turf microplastics pollution rules, Link: https://chemicalwatch-com.proxy.findit.dtu.dk/68742/norways-epa-proposes-artificial-turf-microplastics-pollution-rules?q=microPlastics - accessed 14-8-2019.

ChemicalWatch (CW), 2019m, NGO Platform: Trends in chemical management science and governance, Link: <https://chemicalwatch.com/74531/ngo-platform-trends-in-chemical-management-science-and-governance?q=microPlastics> - accessed 11-06-2019.

Cousteau, P., 2017, Plastics: The invisible oil spill, EURACTIV, Link: <https://www.euractiv.com/section/circular-economy/opinion/plastics-the-invisible-oil-spill/> - accessed 28-10-2019

ECHA, 2019, General Comments and answers to specific information requests, Helsinki: European Chemicals Agency, Link: [https://echa.europa.eu/registry-of-restriction-intentions/-/dislist/details/0b0236e18244cd73 - accessed 28-10-2019](https://echa.europa.eu/registry-of-restriction-intentions/-/dislist/details/0b0236e18244cd73%20-%20accessed%2028-10-2019)

Ends, 2018g, ECHA defends ‘objectivity’ of microplastics call, Link: [https://www.endseurope.com/article/52975/echa-defends-objectivity-of-microplastics-call - accessed 22-10-2019](https://www.endseurope.com/article/52975/echa-defends-objectivity-of-microplastics-call%20-%20accessed%2022-10-2019).

Ends, 2018h, ECHA lacked objectivity, say microplastics campaigners, Link: <https://www.endseurope.com/article/52922/echa-lacked-objectivity-say-microplastics-campaigners> - accessed 22-10-2019

Franklin, K., 2016, Greenpeace slams cosmetic industry microbeads commitments, ChemicalWatch, Link: <https://chemicalwatch.com/48785/greenpeace-slams-cosmetic-industry-microbeads-commitments?q=microplastic> – accessed 20-8-2019.

Keating, D., 2018, A plan for plastics, EURACTIV, Link: <https://www.euractiv.com/section/circular-economy/news/a-plan-for-plastics/> - accessed 28-10-2019

Lovell, T., 2018, NGO report urges fashion brands to join collaborative initiatives, ChemicalWatch, Link: https://chemicalwatch-com.proxy.findit.dtu.dk/67541/ngo-report-urges-fashion-brands-to-join-collaborative-initiatives?q=microPlastics - accessed 14-8-2019.

Oziel, C., 2018, Denmark to impose temporary ban on microplastics in cosmetics, ChemicalWatch, Link: https://chemicalwatch-com.proxy.findit.dtu.dk/72707/denmark-to-impose-temporary-ban-on-microplastics-in-cosmetics?q=microPlastics - accessed 14-8-2019.

Oziel, O., 2018, NGOs attack Echa’s ‘limited’ microplastics restriction proposal, ChemicalWatch, Link: https://chemicalwatch-com.proxy.findit.dtu.dk/67582/ngos-attack-echas-limited-microplastics-restriction-proposal?q=microPlastics - accessed 14-8-2019.

Rodrigo, A. & Powell, D., 2018, Can we tax our way out of the plastic pollution crisis, EURACTIV, Link: <https://www.euractiv.com/section/circular-economy/opinion/can-we-tax-our-way-out-of-the-plastic-pollution-crisis/> - accessed 28-10-2019

Stringer, L., 2019, Plastics exposure a global health crisis, says NGO report, ChemicalWatch, Link: https://chemicalwatch.com/74500/plastics-exposure-a-global-health-crisis-says-ngo-report?q=microPlastics - accessed 12-6-2019.

Zainzinger, V., 2017, NGO coalition urges EU-wide ban on microplastics in cosmetics, ChemicalWatch, Link: <https://chemicalwatch.com/60737/ngo-coalition-urges-eu-wide-ban-on-microplastics-in-cosmetics?q=microplastic>, accessed 20-8-2019.
