# Supplementary material for: Stakeholder analysis with regard to a recent European restriction proposal on microplastics
Source: PLoS One. 2020 Jun 22;15(6):e0235062. doi: 10.1371/journal.pone.0235062 (PMC7307934; doi:10.1371/journal.pone.0235062)
Supplement: S6 Table — (DOCX) [file pone.0235062.s007.docx]

S6 Table: National Authorities categorization table

| ***National Authorities (politicians, parliaments, governments etc.)*** | | | | | | |
| --- | --- | --- | --- | --- | --- | --- |
| **No**. | **Stakeholder** | **Criteria**  (for statement e.g. economy, innovation, environment etc.) | **Principles**  (separate from criteria e.g. PP, values etc.p) | **Scientific argumentation**  Case Reports & Case Series (observational)  Case-control (observational)  Cohort (observational)  Randomized-controlled trials (experimental)  Systematic review | **Research needs**  (identified needs in statement) | **Other** |
| 1 | Belgian Government | Belgium has notified the European Commission of a draft plan to voluntarily phase out microplastics in all consumer products by 2019. (CW, 2017d). |  |  | The deliberate addition of microplastics into disposable consumer products must be "significantly reduced", the notification says. To this end, parties will be obliged to follow the scientific and technological evolutions in this area and to "take the necessary measures in case of new proven problems". (CW, 2017d) |  |
| 2 | Bettina Hoffman (German Green Party) | German Green party environment spokesperson Bettina Hoffmann called on the government to act in line with the precautionary principle and “to stop the entry of microplastics in our environment”, including banning them from cosmetics and from entering compost and digestate from biogas plants. (German green party) (Ends 2018b) |  |  |  |  |
| 3 | Danish EPA | “*The 30 January collaboration, formed to reduce plastic pollution and promote a circular plastic economy, comes after the government released an action plan on reducing plastic waste in December*” 2018 (Buxton, 2019).  “*Late last year, Denmark also said it will*[*impose*](https://chemicalwatch.com/72707/)*a temporary ban on microplastics added to rinse-off cosmetics from 1 January 2020 at the latest. This will stay in place until an EU-wide measure comes into play. It is also considering extending the ban to all cosmetics products within three years*” (Buxton, 2019). |  | The Partnership for Microplastics report, released by the Danish EPA, highlights how little is known about the effects of microplastics on the environment. (CW, 2018zd)  Car tyres, paint, shoes, textiles and other secondary sources are responsible for almost all microplasticpollution in Denmark, according to a study by the country's EPA. (CW, 2015a).  The agency found that secondary microplastics – abrasions of larger plastic particles that are broken down as they make their way to the aquatic environment – make up 99% of the total amount emitted to the aquatic environment. (CW, 2015a). | Microplastics are unlikely to present a risk of contamination to groundwater (CW, 2018u) |  |
| 4 | Danish Government | Minister for Environment and Food Jakob Ellemann-Jensen proclaims that Denmark is to introduce a temporary ban on microplastics for rinse-off cosmetics until an EU-wide measure is implemented. The government also consider a MP ban for all cosmetics within three years (CW, 2018a).  Urged EC to plug knowledge gaps on microplastics in the EU plastics strategy (Zainzinger, 2018)  The Danish government urged the European Commission to help plug the gaps around knowledge of microplastics as it prepares to publish its EU plastics [strategy](https://chemicalwatch.com/52436/). (CW, 2017a).  Denmark’s new environment and food minister, Esben Lunde Larsen, is urging the European Commission to introduce a Europe-wide ban on the use of microplastics in cosmetic products. (Buxton, 2016).  “*We have to limit the spread of microplastics so that it does not end up in the aquatic environment and in the food chain,*” Mr Larsen said in a release. (Buxton, 2016).  “*A ban on microplastics in cosmetics should be considered as a first step in solving the issue and further actions have to be considered,*” Elisabeth Paludan, deputy head of chemicals at the MoE, told Chemical Watch. (Buxton, 2016).  Eva Kjer, the Danish environment and food minister, has said that the spread of microplastic must be curtailed, so that it does not end up in the aquatic environment and food chain. (CW, 2016j). |  |  |  |  |
| 5 | Deutscher Landkreistag & Deutscher Städte- und Gemeindebund | Attachment |  |  |  |  |
| 6 | French Government | Banned rinse-off cosmetic products containing microplastics, January 2018 (CW, 2018e).  France has notified the European Commission it will ban from the market rinse-off cosmetic products containing microplastics by 1 January 2018. (CW, 2016a). |  |  |  |  |
| 7 | German UBA | German Environment Agency (UBA), “UBA believes that voluntary commitments by the cosmetic industry are no longer sufficient and will call on the European Commission to consider a full ban. Since 2013, a voluntary agreement has been in place with the German industry. But UBA expert Marcus Gast claimed that this deal only covers a small number of microplastics, such as those found in toothpaste” (Morgan, 2017) |  | The report, MikroPlastik: Development of an environmental assessment concept, says the effects of microplastics have not been fully analysed. (CW, 2016h).  The report, MikroPlastik: Development of an environmental assessment concept, says the effects of microplastics have not been fully analysed. (CW, 2016h). |  |  |
| 8 | Italian Government | Italy has notified the European Commission of plans to ban the production and marketing of rinse-off cosmetics products containing microplastics (Stringer, 2018).  Tyres are responsible for the unintentional release of microplastics in the marine environment, which are covered by the plastics strategy currently being discussed at EU level. (Italien environmental minister) (Ends 2018f) |  |  |  |  |
| 9 | Mary Creagh (British MP) | Mary Creagh, chair of the environmental audit committee: *“We need a full, legal ban, preferably at an international level as pollution does not respect borders,” she added. “If this isn’t possible after our vote to leave the EU, then the government should introduce a national ban. The best way to reduce this pollution is to prevent plastic being flushed into the sea in the first place”* (Vidal, 2016) |  |  |  |  |
| 10 | Multiple countries | Austria, Sweden and France called for policies to tackle marine litter, with Austria and Sweden both calling for action to address the problem of microplastics in [cosmetics](http://www.endseurope.com/article/45315/). (Ends 2016a) |  |  |  |  |
| 11 | Netherlands Council of Ministers | Microplastics in products is an area of particular concern. In the Council of Ministers, [the Netherlands](http://www.endseurope.com/32144) is pushing for a ban on microplastics in personal care products to reduce the threat they pose to the marine environment. (Ends 2013a) |  | The Dutch document is a response to the European Commission’s [green paper](http://www.endseurope.com/30930) on plastic waste, which identified microplastics as a “particular concern”. They derive mostly from the degradation of plastic litter in the sea. A number of scientific studies have shown they can harm plankton and other marine species. (Ends 2013c) |  |  |
| 12 | Norwegian EPA | The Norwegian EPA has opened a consultation on a proposal to prevent the spread of microplastics from artificial turf pitches that use rubber granules (CW, 2019h).  Proposed measures to reduce the spread of microplastics from artificial turf in sports facilities into the environment (CW, 2018i).  “One of the proposals in the regulation is the requirement for a physical barrier around the course. The measure is effective, but it can be expensive to implement,” said Ellen Hambro, director of Norway’s Environment Agency.  **Comment:**  The Norwegian Environment Agency welcomes the restriction proposal on intentionally added microplastics and thanks ECHA for their efforts on preparing the proposal. The Norwegian CA agrees that there is a need for action at EU level to prevent emissions and minimise releases of microplastics to the environment and hence support the inclusion of intentionally added microplastics in Reach Annex XVII.  Due to the growing awareness and concern with microplastic emissions to the environment in recent years, the Norwegian Environment Agency have initiated several projects to increase the knowledge and to consider possible measures on how to best reduce the releases. An important aim of this national work has been on limiting the release of marine litter which act as a source of secondary microplastics. However, intentionally added microplastics are an important source to the overall emissions of microplastics and we see the current restriction proposal from ECHA as a valuable measure on how to minimise the releases and the potential for cumulative effects arising from the presence of both primary (intentionally added) and secondary microplastics in the environment.  Microplastics in the construction sector  Plastic fibres in concrete is one of the major contributors to plastic pollution from the construction sector in Norway. The Norwegian Environment Agency has produced a guideline for reducing the content of plastic materials in construction in collaboration with the Norwegian Public Roads Administration (NPRA), which is a major partner in Norwegian infrastructure projects related to roads. This guideline is available from the following link (only in Norwegian): https://www.miljodirektoratet.no/globalassets/publikasjoner/M1085/M1085.pdf  **Answer to specific info request 1:**  The proposed definition of microplastic is quite broad and will also cover aspects of particles not traditionally considered as microplastic. A clear definition and requirements to the properties of the particles is of great importance in order to identify a clear scope. The Norwegian Environment Agency considers the assessment of biodegradability to be an important aspect for defining whether a particle is considered as microplastic or not. Whether a material is biodegradable depends strongly on the environment where the particle ends up. As an example, microplastic particles from cosmetics may end up in an aquatic environment directly, if the spill water is discharged without treatment. The same particles may alternatively end up in sewage sludge, which may be used as soil improvement, which again may cause the particles to end up in the soil. We would therefore like to stress that the tiered testing approach should ensure that the material is tested for degradability in all relevant environments.  The Norwegian CA acknowledges the current research efforts on biodegradable materials and supports the suggestion that the biodegradability tests should be put in an appendix in order to allow for regular updates. |  | The Norwegian EPA states “*Microplastics have been found in 35 samples of sediment and bottom-living organisms collected from the seabed of the North Sea and the Barents Sea*” and “*among the most common findings in the sediments analysed were paint, synthetic rubber, chlorinated polyethylene, polyacrylamide and polyethylene terephthalate*” (CW, 2019p).  **Answer to specific info request 2:**  a) Norway has about 1700 artificial sports fields, and it is estimated that 90% uses infill material (granules) produced from tyres.  b) It is estimated that the average sport fields need to refill 5.5 tons of granules per year. It is not known how much of this is released into the environment; some is compacted into the bottom support of the sports field.  c) The Norwegian Environment Agency has on commission from the Ministry of Climate and Environment outlined a draft regulation on design and management of artificial sports fields that uses loose infill containing plastic. Our conclusion from the regulation work in 2018 is that spreading of plastic infill will best be avoided with a regulation that has requirements both on how the fields are built up and how they are maintained. The requirements are functional, so the technical solutions can differ.  The requirements are:  1. A physical barrier around the field, at least 20 cm high. The physical barrier will hinder transport out from the field area for example during heavy rain and flooding incidents.  2. Solutions that hinders particle transport into sewage system from drainage and to nearby environment from surface water. Solutions will differ depending on the field design and local conditions but can for example be particle catching equipment in yard manholes.  3. Measures that reduces spreading of infill material out of the field via the users (players etc.). There are several measures that the users can do to reduce spreading. The draft regulation aims primary to measures like shoe cleaning and removing infill material from clothing, by installing equipment like grating, brushes etc. It can also be relevant to steer the traffic to these measures. These measures are to be seen in correlation with the requirement for the field responsible to give information about the need to reduce spreading and how the users can reduce it.  4. Deposits for snow. Most of Norway have snowfall in wintertime and if the snow is cleared away outside the field it shall be deposited in an area design to hold on the granules, so they can be returned to the field after the snow has melt. The deposit area is to have solid base and have barriers that ensures that the infill material remains in the deposit area. | The Norwegian Environment Agency is looking at treatment solutions for capturing microplastics from roads in congested areas to prevent them from entering the wastewater system. (CW, 2018v)  The move forms part of a [strategy](https://chemicalwatch.com/54110/) set out by Norway’s climate and environment minister Vidar Helgesen last year to investigate ways of reducing marine litter and microplastic in the ocean. (CW, 2018v)  The Norwegian Environment Agency has investigated possible measures to reduce the spread of microplastics into the environment. (CW, 2017t).  The EA say that 10% of plastic infill is lost each year. "Many sports clubs are already making an effort to prevent micro-plastic discharge, but more needs to be done. It is possible to reduce emissions by up to 98% from such courses,” Hambro explains. (Ends 2018e)  It was found that for most applications, the plastic reinforcement fibres can be substituted with iron/steel fibres, and this is to be incorporated in the procedures for construction projects carried out by the Norwegian Public Roads Administration as a means to reduce the plastic littering. We are currently not aware whether other major partners (related to e.g. railways) are taking similar actions.  The Norwegian CA notes that the proposed restriction is quite complex with many derogations and definitions and there are some possible overlaps with other regulations which may lead to challenges in terms of enforceability. There could possibly also be some difficulties related to the analytical methods needed for analysing particle size, particle size distribution and determination of (bio)degradability for polymers.  Monitoring data  Some recently published reports/monitoring data on the occurrence and releases of microplastics in the Norwegian environment are available from the following links:  Freshwater microplastics in Norway (M-1212/2018)  https://www.miljodirektoratet.no/globalassets/publikasjoner/m1212/m1212.pdf  Microplastics in polychaetes from the Norwegian Continental Shelf (M-1222/2018)  https://www.miljodirektoratet.no/globalassets/publikasjoner/m1222/m1222.pdf  Microplastics in sediments on the Norwegian Continental Shelf II: Identification through FT-IR analysis (M-1231/2018)  https://www.miljodirektoratet.no/globalassets/publikasjoner/m1231/m1231.pdf  It should be noted that the monitoring data does not allow a distinction between secondary and ‘intentionally added’ microplastics. | Incorporation of the requirements above is estimated to reduce the spreading of infill material with up to 96- 98 percent.  d) The costs described below are based on a standard size football field, with no systems to reduce the loss of infill material in place. Many fields in Norway do have some systems in place already, hence the expected cost will be towards the lower end of the given range.  Concrete barrier (min. 20 cm) around the sports field, incl. Digging and concrete formwork: 4 900-24 500 Euro  Shock-absorbing area between the concrete barrier and the field: 13 100 Euro  Area for deposit of snow: 0-23 600 Euro  Filter to catch the infill material from water run off:  4 900-5 700 Euro  Systems to catch infill material at entry/exit points to the field: 4 100-5 100 Euro  Brushes to catch infill material at entry/exit points to the field: 410-510 Euro  Engineering/project management: 1 020-10 400 Euro  e) As part of the preparatory work in relation to the national draft regulation, substitution of the granulates with alternative materials was investigated. At present, none of the materials considered was found to be viable alternatives. The main obstacle for replacing the use of rubber granulates is cold and wet weather. It was noted that development of new materials could lead to replacement of the rubber granulates. The report "Environmentally friendly substitute products for rubber granulates as infill for artificial turf fields" (M-955/2018) from this work can be found via the following link:  https://www.miljodirektoratet.no/globalassets/publikasjoner/m955/m955.pdf |
| 13 | Norwegian Government | *“According to the Norwegian environment minister Ola Elvestuen, turf pitches containing them are one of the largest sources of microplastics spreading in Norway”* (CW, 2019h).  Norway’s climate and environment minister has called for an investigation into ways of tackling marine litter and microplastic in the ocean. (CW, 2017p).  Norway’s Special Representative for the Oceans and former minister for EU affairs, Vidar Helgesen: “The EU’s plastic strategy and its circular economy package is very important. Norway is part of the single market and we welcome common European approaches. The EU-ban on single-use plastics will have a tremendous effect, because the EU is such a big market,”; “I am not sure that in 10 years we will have less plastics in the oceans than we have today, but the flow of plastics into the oceans will be smaller.” And “The attention to the issue among the electorate and at the highest political level is a good sign, but sadly we will see for a number of years increased plastic in the oceans before new measures will take effect. You have a number of countries particularly in the Asia-Pacific where it is critically important to get waste management systems into place and where it will take time,” (Breum 2019) |  |  | Norway’s climate and environment minister has asked for proposals for rules to be drawn up to reduce the spread of microplastic from artificial turf used in sports facilities. (CW, 2018z) |  |
| 14 | RIVM |  |  | Tyres, paints and abrasive cleaning agents can release microplastic armoniz that end up in soil, water and air, according to a report commissioned by the Dutch environment ministry. (CW, 2016e). | RIVM says it is essential to create awareness of the problem among consumers and professionals. It argues the release of microplastics can be reduced through innovation and by implementing measures preventing the distribution of particles into the environment. (CW, 2016e). |  |
| 15 | Swedish Association of Local Authorities and Regions | **Answer to specific info request 1:**  Legislation and voluntary standards is practically applicable to the situation where rubber granules is used on artificial turfs. (see separate attachment)  **Answer to specific info request 2:**  2. The supervision and analyzing costs can get extremely high for the municipalities if granules is not under the scope of this legislation. (see separate attachment) |  |  | **Answer to specific info request 5:**  SALAR recommend ECHA to further analyse the market for rubber infill materials in horseback riding ground surfaces. |  |
| 16 | Swedish Chemicals Agency | Warn that artificial turf contributes to an increased presence of both microplastics and hazardous substances (2018m).  KEMI recommended that manufacturing companies voluntarily restrict the use of microplastics in deodorants, eye creams, hair gels and hair mousses, body creams and face creams, all kinds of makeup – mascara, eyeliner, rouge, powder and lipstick – anti-ageing skin products, tanning products and sunscreens, and bleaching creams. (Swedish KEMI) (Ends 2018i) |  | Sweden’s chemical agency Kemi, which carried out the research, says “the work being done at EU level on restriction proposals could result in reliable decision material and clear and armonized rules and regulations which would also be cost-effective”. (CW, 2018y) | A Swedish investigation into whether further national restrictions on microplastics in cosmetics and other chemical products are needed concluded that such action would be better carried out at EU level in the first instance. (CW, 2018y)  The agency says other measures are needed to reduce the amount of microplastics in oceans and lakes. (CW, 2016j). |  |
| 17 | Swedish Government | The deputy prime minister of Sweden, Isabella Lövin, stresses: “The government will push the international community to agree strong measures to tackle the adverse impacts of chemicals and plastics” (Stringer, 2019).  A Swedish ban on rinse-off cosmetics containing microbeads will enter into force at the beginning of July, the country’s environment ministry has said. (CW, 2018za)  Sweden is considering extending its proposed ban on microbeads in rinse-off cosmetics to all products that release microplastics. (CW, 2018zb)  Sweden has proposed a ban on rinse-off cosmetics that contain microplastics to reduce their impact on the marine environment. (CW, 2017g).  Sweden said that with today’s technology it is impossible to capture and collect plastic particles once they are released into the environment. “*It is therefore necessary to take measures at the source*,” the notification said, adding “*there are no less restrictive measures*” which would achieve the same purpose. (CW, 2017g).  The Swedish government is planning national legislation to prevent microplastics getting into the environment, and emission control limits for construction products, (Lovell, 2016) |  |  |  |  |
| 18 | The Swedish Medical Products Agency | The Swedish Medical Products Agency supports initiatives that are taken to meet the global climate goals. The Swedish Medical Products Agency also supports a derogation for medical devices and in vitro diagnostic medical devices.  However, The Swedish Medical Products Agency believes that the consequences of the proposal on Medical Devices and In vitro diagnostic medical devices need to be further explored, especially related to consequences on patient safety, socio-economic impacts and time for implementation. The proposal can lead to shortage of products and put patients at risk.  The socio-economic impact presented by the Dossier Submitter appears to be based on a limited number of products used in professional settings. Despite lack of access to a complete list of products containing microplastic, the summary of products and their uses appear to be incomplete.  The Swedish Medical Products Agency has noticed that CE-labelled absorbance products such as incontinence products are not mentioned in the documentation on the proposed restriction. Such absorbance products can contain superabsorbent microplastics. This product category is subsidized by the Swedish government and are not only used by professionals under controlled conditions. These products are high volume products, both used by professionals and other users, at both hospitals and in the homes of patients. Today, waste from such products are typically handled as household waste. In Sweden the majority of household wastes are incinerated. Still, requirements on incineration or deposition as hazardous waste management treatment of such waste can have impact on the health care system, including patient safety.  The Dossier Submitter suggests that the use and release shall be monitored and reported, and that compliance can be monitored at member state level by reviewing PSUR.  According to article 86 in the medical device regulation (2017/745), “manufacturers of class IIa, class IIb and class III devices shall prepare a periodic safety update report (‘PSUR’). Manufacturers of class IIb and class III devices shall update the PSUR at least annually and class IIa devices shall update the PSUR when necessary and at least every two years. For class III devices or implantable devices, manufacturers shall submit PSURs by means of the electronic system. For class I, IIa and IIb devices, manufacturers shall make PSURs available to the notified body involved in the conformity assessment and, upon request, to competent authorities.” This will increase the burden on notified bodies and competent authorities. The be noticed, an electronic system is still not in place and the majority of the PSURs are not going to be actively submitted to this system and would have to be requested by the competent authorities. Also, the environmental aspects of devices are not covered by regulation 2017/745, in particular the provisions on PSURs, and this would be an additional demand put on the manufacturers based on other legislation. Hence, the feasibility to monitor and report requires further attention.  The Dossier Submitter suggests that medical devices and in vitro diagnostic products can adapt to the regulation within 2 years, i.e. implement technical means where microplastics would be contained throughout their use and incinerated at the end of their life-cycle and update labels, SDS, IFU to provide enough instructions to prevent release to the environment. Even such seemingly non-intrusive modifications may entail scrutiny by notified bodies, for the devices that require a certificate. The Swedish Medical Products Agency believe that this is probably a too short implementation period and suggest that the implementation time for updates of information should be decided when all processes for MDR and IVDR are in place. Competent authorities, notified bodies and companies are working hard to adapt to the new requirements. As of today, all processes are not yet in place. Additionally, microplastics are most often added to the products with purpose to provide unique functions. Such products can be advanced. A transition time of 2 years to replace such product with non-microplastic solutions is for most products more than a challenge, even modifying product to contain microplastics throughout their use can be challenging. Research and development achievements are probably required. Such technical means most likely require more than 2 years implementation time.  The Swedish Medical Products Agency highly recommend that the consequences of the proposal for Medical Devices and In vitro diagnostic products are further evaluated to avoid shortage of products and that patient’s safety are compromised. |  | **Comment:**  The Swedish Medical Products Agency wants to emphasize the importance that the restriction report includes a clear conformation for competent authorities to perform in market surveillance of microplastic in cosmetic products. We would also like to express concerns regarding inadequate in market surveillance if the legal requirements are unclear, for e.g. in the case of unclear description of what and when something is a microplastic at different stages (from production to the final product).  **Answer to specific info request 3:**  In medical devices, microplastics can be intentionally added with properties essential for the function of the device. The amount can be far above the proposed concentration limit of 0.01%, as described in Annex XV restriction report, proposal for a restriction, version 1.1., March 20, 2019. | Regulation (EC) No 1223/2009 on cosmetic products states in article 3 that “a cosmetic product made available on the market shall be safe for human health when used under normal or reasonably foreseeable conditions of use”. The safety of a cosmetic product is demonstrated by ensuring that a cosmetic product has undergone a safety assessment (which, among other things, must take systemic -and local toxicity into account).  The Swedish Medical Products Agency wants to highlight concerns regarding the risk to human health if the transitional periods for alternative ingredients to microplastics are too short (for other rinse-off and leave on cosmetic products). Also, the risk to human health if alternatives to microplastics are introduced too quickly.  Animal testing is strictly prohibited for finished cosmetic products and ingredients exclusively used in cosmetics or specifically carried out in the context of the EU cosmetic regulation. Since alternative methods for systemic toxicity is at current date not available, implications for the development of new ingredients are at place. Abundant data from tests of good quality are needed for the safety assessment, to ensure that a cosmetic product is safe for human health under normal condition of use.  However, for alternatives to microplastic ingredients that are already available on the market (and already assessed as safe), a transitional period of 4-6 years could be appropriate. |  |
| 19 | United Kingdom Government | No intention to ban wet wipes (CW, 2018s)  A ban on the manufacture of cosmetics and personal care products containing plastic microbeads comes into effect in the UK today. (CW, 2018ze)  The UK's new environment minister has repeated the government's pledge to introduce legislation to ban the manufacture of rinse-off cosmetics containing microbeads from 1 January 2018, and their sale from 30 June the same year. (Zainzinger, 2017)  A UK cross-party committee of MPs has called for a ban on plastic microbeads in cosmetics. (CW, 2016c).  The UK House of Commons’ Environmental Audit Committee has launched an inquiry into the environmental impact of microplastics. (CW, 2016i). |  |  | The environment ministry, Defra, has promised to review evidence on solid plastic particles in products outside of the scope of the ban, together with the Hazardous Substances Advisory Committee (HSAC), and to "assess the potential for further actions". (CW, 2017f). |  |
| 20 | UVW | New challenges posed by microplastics were also discussed at the seminar. Greet De Gueldre of Belgian wastewater treatment firm Aquafin and Michaël Bentvelsen, speaking on behalf of Dutch association [UVW](http://www.uvw.nl/), made a case for restricting the use of these substances in personal care products.  In their joint presentation, they said NGOs had been effective at raising awareness of this issue in the Netherlands. Their actions led some retailers to ban sales of products with plastic ingredients. The country’s authorities are also planning to request a ban on these products at European level. (Ends 2013b) |  |  |  |  |
| 21 | Vereniging Sport en Gemeenten | Information on costs;Information on benefits;Other socio economic analysis (SEA) issues;Transitional period;Request for exemption  **Attachment:** |  |  |  |  |

**References**

Breum, M. 2019. Plastic pollution increasing at the top of the Earth. EUObserver. Link: <https://euobserver.com/nordic/144003>

Buxton, L, 2016, Denmark calls for EU ban on microplastics in cosmetics, ChemicalWatch, Link: <https://chemicalwatch.com/47321/denmark-calls-for-eu-ban-on-microplastics-in-cosmetics?q=microplastic> – accessed 20-8-2019.

Buxton, L., 2019, Cross-party agreement bolsters Danish microplastic efforts, ChemicalWatch, Link: https://chemicalwatch.com/74047/cross-party-agreement-bolsters-danish-microplastic-efforts?q=microPlastics - accessed 12-6-2019.

ChemicalWatch (CW), 2015a, Denmark identifies main sources of microplastic pollution, Link: <https://chemicalwatch.com/43667/denmark-identifies-main-sources-of-microplastic-pollution?q=microplastic> – accessed 20-8-2019.

ChemicalWatch (CW), 2016a, France to ban microplastics in some cosmetics products, Link: <https://chemicalwatch.com/50368/france-to-ban-microplastics-in-some-cosmetics-products?q=microplastic> – accessed 20-8-2019.

ChemicalWatch (CW), 2016c, UK MPs call for ban on microbeads in cosmetics, Link: <https://chemicalwatch.com/49263/uk-mps-call-for-ban-on-microbeads-in-cosmetics?q=microplastic> – accessed 20-8-2019.

ChemicalWatch (CW), 2016e, RIVM tracks microplastics from tyres, paints and cleaning agents, Link: <https://chemicalwatch.com/48495/rivm-tracks-microplastics-from-tyres-paints-and-cleaning-agents?q=microplastic> – accessed 20-8-2019.

ChemicalWatch (CW), 2016h, Germany’s UBA published microplastics report, Link: <https://chemicalwatch.com/46653/germanys-uba-publishes-microplastics-report?q=microplastic> – accessed 20-8-2016.

ChemicalWatch (CW), 2016i, UK parliamentary committee launches microbeads inquiry, Link: <https://chemicalwatch.com/46591/uk-parliamentary-committee-launches-microbeads-inquiry?q=microplastic> – accessed 20-8-2019.

ChemicalWatch (CW), 2016j, Sweden and Denmark move on microbeads, Link: <https://chemicalwatch.com/44608/sweden-and-denmark-move-on-microbeads?q=microplastic> – accessed 20-8-2019.

ChemicalWatch (CW), 2017a, Help close microplastic knowledge caps, Denmark tells Commission, Link: <https://chemicalwatch.com/62446/help-close-microplastic-knowledge-gaps-denmark-tells-commission?q=microplastic> – accessed 20-8-2019.

ChemicalWatch (CW), 2017d, Belgium mulls ‘total ban’ on microplastics in consumer products, Link: <https://chemicalwatch.com/59707/belgium-mulls-total-ban-on-microplastics-in-consumer-products?q=microplastic> – accessed 20-8-2019.

ChemicalWatch (CW), 2017g, Sweden proposes ban on microbeads in rinse-off cosmetics, Link: <https://chemicalwatch.com/57599/sweden-proposes-ban-on-microbeads-in-rinse-off-cosmetics?q=microplastic> – accessed 20-8-2019.

ChemicalWatch (CW), 2017p, Norwegian environment minister launches microplastics investigation, Link: <https://chemicalwatch.com/54110/norwegian-environment-minister-launches-microplastics-investigation?q=microplastic> – accessed 20-8-2019.

ChemicalWatch (CW), 2017t, Norway looks at reducing microplastic pollution, Link: <https://chemicalwatch.com/51964/norway-looks-at-reducing-microplastic-pollution?q=microplastic> – accessed 20-8-2019.

ChemicalWatch (CW), 2018i, Norway's EPA proposes artificial turf microplastics pollution rules, Link: https://chemicalwatch-com.proxy.findit.dtu.dk/68742/norways-epa-proposes-artificial-turf-microplastics-pollution-rules?q=microPlastics - accessed 14-8-2019.

ChemicalWatch (CW), 2018m, Swedish chemicals agency warns about hazardous substances in artificial turf, Link: https://chemicalwatch-com.proxy.findit.dtu.dk/68235/swedish-chemicals-agency-warns-about-hazardous-substances-in-artificial-turf?q=microPlastics - accessed 14-8-2019.

ChemicalWatch (CW), 2018e, France ban rinse-off cosmetic products containing microplastics, Link: https://chemicalwatch-com.proxy.findit.dtu.dk/70462/french-assembly-votes-to-ban-plastic-fcms-in-school-canteens?q=microPlastics - accessed 14-8-2019.

ChemicalWatch (CW), 2018s, UK government denies ban on wet wipes, Link: https://chemicalwatch-com.proxy.findit.dtu.dk/66792/uk-government-denies-ban-on-wet-wipes?q=microPlastics - accessed 14-8-2019.

ChemicalWatch (CW), 2018u, Microplastics 'low risk' for groundwater pollution – Danish study, Link: <https://chemicalwatch-com.proxy.findit.dtu.dk/66444/microplastics-low-risk-for-groundwater-pollution-danish-study?q=microPlastics> - accessed 14-8-2019.

ChemicalWatch (CW), 2018v, Norway investigating solutions for tackling road dust microplastics, Link: <https://chemicalwatch.com/66144/norway-investigating-solutions-for-tackling-road-dust-microplastics?q=microplastic> – accessed 20-8-2019.

ChemicalWacth (CW), 2018y, Sweden advocates developing microplastic restrictions at EU level, Link: <https://chemicalwatch.com/65507/sweden-advocates-developing-microplastic-restrictions-at-eu-level?q=microplastic> – accessed 20-8-2019.

ChemicalWatch (CW), 2018z, Norway planning rules on emissions from artificial turf microplastics, Link: <https://chemicalwatch.com/64144/norway-planning-rules-on-emissions-from-artificial-turf-microplastics?q=microplastic> – accessed 20-8-2019.

ChemicalWatch (CW), 2018zb, Sweden considering wider restrictions on microplastics, Link: <https://chemicalwatch.com/63246/sweden-considering-wider-restrictions-on-microplastics?q=microplastic> – accessed 20-8-2019.

ChemicalWatch (CW), 2018zd, Danish study finds understanding of microplastics ‘extremely defectice’, Link: <https://chemicalwatch.com/63130/danish-study-finds-understanding-of-microplastics-extremely-defective?q=microplastic> – accessed 16-1-2018.

ChemicalWatch (CW), 2019h, Norway consults on proposal to curb artificial turf microplastics emissions, Link: https://chemicalwatch.com/79366/norway-consults-on-proposal-to-curb-artificial-turf-microplastics-emissions?q=microPlastics - accessed 11-6-2019.

ChemicalWatch (CW), 2019p, Norway finds microplastics at bottom of North Sea, Link: https://chemicalwatch.com/74427/norway-finds-microplastics-at-bottom-of-north-sea?q=microPlastics - accessed 12-6-2019.

ECHA, 2019, General Comments and answers to specific information requests, Helsinki: European Chemicals Agency, Link: [https://echa.europa.eu/registry-of-restriction-intentions/-/dislist/details/0b0236e18244cd73 - accessed 28-10-2019](https://echa.europa.eu/registry-of-restriction-intentions/-/dislist/details/0b0236e18244cd73%20-%20accessed%2028-10-2019).

Ends, 2013a, Stakeholders back EU landfill ban for plastics, Link: <https://www.endseurope.com/article/33242/stakeholders-back-eu-landfill-ban-for-plastics> - accessed 23-10-2019

Ends, 2013c, Ban microplastics in cosmetics, says the Netherlands, Link: <https://www.endseurope.com/article/32144/ban-microplastics-in-cosmetics-says-the-netherlands> - accessed 23-10-2019

Ends, 2016a, Strong support for circular economy eco-design at Council, Link: <https://www.endseurope.com/article/45350/strong-support-for-circular-economy-ecodesign-at-council> - accessed 23-10-2019

Ends, 2018b, Evidence of microplastics contamination in human gut, Link: <https://www.endseurope.com/article/54056/evidence-of-microplastic-contamination-in-human-gut> - accessed 23-10-2019

Ends, 2018e, Norway to tackle discharge of microplastics from sports field, Link: <https://www.endseurope.com/article/53317/norway-to-tackle-discharge-of-microplastic-from-sports-fields> - accessed 23-10-2019

Ends, 2018f, Italian environment minister calls for labelling of tyres to boost recycling, Link: <https://www.endseurope.com/article/53032/italian-environment-minister-calls-for-labelling-of-tyres-to-boost-recycling> - accessed 23-10-2019

Ends, 2018i, Sweden calls for further restrictions on microplastics, Link: <https://www.endseurope.com/article/52295/sweden-calls-for-further-restrictions-on-microplastics> - accessed 23-10-2019

Lovell, T., 2016, Sweden to legislate against microplastics, ChemicalWatch, Link: <https://chemicalwatch.com/49942/sweden-to-legislate-against-microplastics?q=microplastic> – accessed 20-8-2019.

Morgan, S., 2017, UN agrees on resolution to combat ‘planetary crisis’ of ocean plastics, EURACTIV, Link: <https://www.euractiv.com/section/energy-environment/news/un-agrees-on-resolution-to-combat-planetary-crisis-of-ocean-plastics/> - accessed 28-10-2019

Stringer, L., 2018 Italy to ban microplastics used in rinse-off cosmetics products, ChemicalWatch, Link: https://chemicalwatch-com.proxy.findit.dtu.dk/67533/italy-to-ban-microplastics-used-in-rinse-off-cosmetics-products?q=microPlastics - accessed 14-8-2019.

Stringer, L., 2019, Sweden deputy PM: stronger global action on chemicals needed, ChemicalWatch, Link: https://chemicalwatch.com/74998/sweden-deputy-pm-stronger-global-action-on-chemicals-needed?q=microPlastics - accessed 11-6-2019.

Vidal, J., 2016, Microplastics should be banned in cosmetics to save oceans, British MPs say, EURACTIV, Link: <https://www.euractiv.com/section/climate-environment/news/microplastics-should-be-banned-in-cosmetics-to-save-oceans-british-mps-say/> - accessed 28-10-2019

Zainzinger, V., 2017, Ban on microbreads in UK rinse-off cosmetics confirmed, ChemicalWatch, Link: <https://chemicalwatch.com/57857/ban-on-microbeads-in-uk-rinse-off-cosmetics-confirmed?q=microplastic> – accessed 20-8-2019.

Zainzinger, V., 2018, Bottled water study flags small-sized pigment particles, microplastics, ChemicalWatch, Link: https://chemicalwatch-com.proxy.findit.dtu.dk/69219/bottled-water-study-flags-small-sized-pigment-particles-microplastics?q=microPlastics - accessed 14-8-2019.
