# Supplementary material for: Stakeholder analysis with regard to a recent European restriction proposal on microplastics
Source: PLoS One. 2020 Jun 22;15(6):e0235062. doi: 10.1371/journal.pone.0235062 (PMC7307934; doi:10.1371/journal.pone.0235062)
Supplement: S7 Table — (DOCX) [file pone.0235062.s008.docx]

S7 Table: National NGOs categorization table

| ***National NGOs*** |
| --- |

| **No**. | **Stakeholder** | **Criteria**  (for statement e.g. economy, innovation, environment etc.) | **Principles**  (separate from criteria e.g. PP, values etc.p) | **Scientific argumentation**  Case Reports & Case Series (observational)  Case-control (observational)  Cohort (observational)  Randomized-controlled trials (experimental)  Systematic review | **Research needs**  (identified needs in statement) | **Other** |
| --- | --- | --- | --- | --- | --- | --- |
| 1 | Badischer Fußballverband e. V. | a.: Nach neueren Erkenntnissen werden zwischen 0,25 t/a und 5 t/a in Deutschland an Befüllungsgranulat pro Kunststoffrasenfläche verwendet (Fraunhofer 2018, S. 11). Das entspricht einer Gesamtmenge von ca. 7.500 bis 9.900 t/a.  b.: Nach dem aktuellen Forschungsstand besteht nach Kenntnis des DFB ein hohes Maß an Unsicherheit darüber, wie und in welchen Mengen das als Mikroplastik definierte Granulat auf Sportplätzen in die Umwelt freigesetzt wird. Nach den uns zur Verfügung stehen-den Informationen gibt es große Unterschiede bei der Einschätzung der Menge an Mikroplastiken, die in den einzelnen Mitgliedstaaten oder in der EU/EWR als Füllmaterial für Kunstrasen verwendet wird. Insbesondere Umfang und Methodologie der Forschung in diesem Bereich sind bisher noch wenig standardisiert und nachvollziehbar. Der DFB geht davon aus, dass der Anteil des Eintrags von Mikroplastik über Kunststoffrasenplätze je nach Mitgliedstaat ca. 1 bis 3 Prozent im Verhältnis zum Gesamteintrag beträgt. Demnach ist der Umwelteintrag verglichen mit anderen Hauptquellen relativ gering (Europäische Kommission 2018, ii)).  d.: In Deutschland gibt es ca. 5.000 für den Fußballspielbetrieb gemeldete Kunststoffrasenplätze (DFBnet), sowie ca. 1.000 DFB-Minispielfelder. Jährlich werden in Deutschland ca. 300 Kunststoffrasenplätze neu gebaut, sowie 150 Kunststoffrasenplätze von Grund auf erneuert. Hinsichtlich der bestehenden Plätze dürfte eine Umstellung auf alternative Füllstoffe notwendig sein. Hierfür halten die Sportanlagenbetreiber (Kommunen oder Vereine) Mittel für Sportstättenbau und -sanierung vor, die bei einem vollständigen Ver-bot und einer Verwendung alternativer Füllstoffe deutlich höher ausfallen würden. Laut eigener Berechnungen belaufen sich die jährlichen Mehrkosten deutschlandweit auf einen hohen einstelligen Millionenbetrag. Die insgesamt zu erwartenden Kosten eines Verbotes können aufgrund fehlender Kenntnisse über geeignete alternative Füllstoffe (Geeignetheit, Verfügbarkeit) derzeit nicht seriös beziffert werden. Auf Grundlage aktueller Daten zum Bau von Kunststoffrasenplätzen dürfte der Gesamtbetrag für den Austausch des Füllstoffes der Kunststoffrasensysteme im hohen zweistelligen Millionenbereich (bis zu 90 Mio. EUR) liegen, wobei zur Präzisierung dieses Schätzwertes vertiefte Analysen erforderlich sind. Die Kosten für eine Umsetzung gezielter Risikomanagementmaßnahmen zur Zurückhaltung des Materialaustrags dürften nach Schätzungen und je nach Umfang der Maßnahmen pro Kunststoffrasensystem bei 3.000 bis 10.000 EUR liegen.  e.: Der gemeinwohlorientierte Sport ist die größte zivilgesellschaftliche Bewegung in Deutschland und Europa. In Deutschland engagieren sich knapp acht Millionen Bürger freiwillig und ehrenamtlich im Sport. Das entspricht einer jährlichen Wertschöpfung und einem Wohlfahrtsgewinn allein in Deutschland von ca. 6,7 Milliarden Euro. Vergleichbare Zahlen lassen sich auch für die gesamte EU feststellen. In den EU-Mitgliedstaaten engagieren sich im Jahre 2010 zwischen 92 und 94 Millionen Menschen freiwillig für Ziele des Gemeinwohls, davon die meisten im Sport (ca. 35 bis 40 Prozent aller freiwillig Tätigen in der EU) (Europäische Kommission 2010).  Der Sport schafft ein strukturiertes, an die gesamte Bevölkerung gerichtetes und für alle offenes Bewegungs- und Sportangebot, durch das wichtige soziale und gesundheitsfördernde Funktionen in der Gesellschaft erfüllt werden. Sportvereine in Deutschland zählen zehn Millionen Mitgliedschaften im Kinder- und Jugendalter (DOSB-Bestandserhebung 2018), allein im DFB liegt diese Zahl bei 2,1 Millionen (DFB-Mitgliederstatistik 2018). Damit sind Sportvereine die wichtigste Anlaufstelle für Kinder und Jugendliche außerhalb der Schule und übernehmen unverzichtbare Aufgaben für die ganzheitliche Persönlichkeitsbildung junger Menschen. Dem Sport kommt eine wichtige Vorbild- und Lehrfunktion im Bereich der Integration und demokratischen Grundbildung zu. Für das herausragende gesellschaftliche Engagement des Sports spricht nicht zuletzt, dass die Sportvereine eng mit Schulen, Kindergärten, Unternehmen, Krankenkassen oder anderen öffentlichen Institutionen zusammenarbeiten. Um allen Bürgern den Zugang zum Sport zu ermöglichen, sind adäquate Sportstätten in ausreichender Anzahl Grundvoraussetzung. Ein für alle zugängliches und umfangreiches Sportangebot ist – vor allen Dingen in Großstädten und Ballungsgebieten – nur durch die Verfügbarkeit von ganzjährig nutzbaren Sportanlagen zu gewährleisten. . Kunststoffrasenplätze spielen hierbei, insbesondere für den Fußball, eine wichtige Rolle, da sie eine intensivere Nutzung als Naturrasen- oder Tennenplätze erlauben. Allein mit Naturrasen- und Tennenplätzen lässt sich der derzeitige Trainings- und Spielbetrieb, insbesondere bei den Kinder- und Jugendmannschaften, nicht aufrechterhalten. Ein Kunststoffrasenplatz ersetzt etwa 2,5 Naturrasenplätze (DFBnet). Auf weniger als 10 Prozent der Naturrasenplätze finden an Wochenenden mehr als 2 Spiele statt. Bei Kunststoffrasenplätzen finden hingegen bei über 40 Prozent der Plätze mehr als 2 Spiele statt. Weniger als 10 Prozent der Naturrasenplätze wird an einem Wochenende mehr als 150 Minuten genutzt. Bei Kunstrasenplätzen werden hingegen etwa 35 Prozent an einem Wochenende mehr als 150 Minuten genutzt. 27.773 Spielstätten in Deutschland (ca. 70 Prozent) werden von Sportvereinen genutzt. Ein Drittel der Kunstrasenplätze werden von 2 oder mehr Vereinen mit alle ihren Jugend- und Seniorenmannschaften benutzt. Etwas über ein Drittel aller Naturrasenplätze wird von mehr als 5 Mannschaften bespielt. Bei Kunstrasenplätzen werden fast drei Viertel (72 Prozent) von mehr als 5 Mannschaften genutzt. Etwa 10 Prozent aller Naturrasenplätze wird von mehr als 10 Mannschaften bespielt. Bei Kunststoffrasenplätzen sind es ca. 41 Prozent der Plätze, die von mehr als 10 Mannschaften genutzt. Nur 1 Prozent aller Naturrasenplätze wird von mehr als 15 Mannschaften bespielt. Bei Kunstrasenplätzen beträgt der Anteil immerhin noch knapp 18 Prozent. Etwa 6 Prozent werden sogar von über 20 Mannschaften bespielt. Je größer die Vereinsgröße (insbesondere Anzahl der Mannschaften), desto höher ist der Anteil der Vereine, die auch eine Spielstätte vom Typ Kunstrasen haben.  Ein Verbot des Inverkehrbringens von Kunststoffgranulaten als Füllstoff in Kunststoffrasensystemen direkt bei Inkrafttreten der Beschränkung wäre daher unverhältnismäßig. Es würde zu hohen, unerwarteten Umstellungskosten und Mehrkosten für Vereine und Kommunen führen, wodurch dem gemeinwohlorientierten Sport Mittel entzogen würden. Bei fehlender Finanzierbarkeit dieser Mehrkosten ist zudem von einer Schließung vieler Sportplätzen auszugehen, wodurch das Sportangebot in Schulen und Vereinen stark leiden würde. Gerade auf Vereinsebene stellt ein solch außerordentlicher Kosten-punkt ein großes finanzielles Risiko dar, dass das sportliche und gesellschaftliche Gesamtangebot des Vereins gefährden kann. Eine Beschränkung ohne Übergangsfristen, die eine mittelfristige Umstellung und Kostenstreckung erlauben, würde das Breitensportangebot in Deutschland sehr negativ beeinflussen.  Im Hinblick auf den Beschränkungsvorschlag der ECHA gemäß Anhang XV der REACH-Verordnung spricht sich der DFB daher für eine angemessene Übergangsfrist von mindestens sechs Jahren bis zu einem vollständigen Inverkehrbringungsverbot des Kunststoffgranulats zur Verwendung in neuen Kunststoffrasensystemen sowie für die Umstellung bestehender Flächen aus.  Im Badischen Fußballverband sind 609 Vereine mit über 200.000 Mitgliedern organisiert. Ein besonderer Schwerpunkt der verbandlichen Arbeit liegt im Bereich der Kooperation zwischen Schule und Verein sowie der Installierung von Maßnahmen und Projekten im Bereich des Freiwilligendienstes (FSJ). Knapp 60 FSJ-Projekte betreuen knapp 400 Kooperationsmaßnahmen Schule und Verein, die bei einer negativen Entscheidung hinsichtlich der Kunstrasenplätze in Gefahr geraten und damit das gesellschaftliche Engagement der Vereine in den Schulen gefährden würden. Besonders die Möglichkeit einer ganzjährigen Jugendarbeit mit Hilfe der Kunstrasenplätze als Existenzgrundlage für die Vereine würde damit verloren gehen, weshalb sich der Badische Fußballverband der Darstellung des DFB anschließt. |  | Neben dem häufig genutzten Kunststoffgranulat existieren für Kunststoffrasensysteme alternative Füllstoffe, die in Teilen auch bereits beim Betrieb von Sportanlagen genutzt werden. So werden in Deutschland aktuell Kunststoffrasenplätze teilweise mit Sand und/oder Kork verfüllt. Zudem gibt es auch Kunststoffrasensysteme, die ohne elastischen Füllstoff betrieben werden können.  Es existieren bisher allerdings nur wenige belastbare Studien darüber, wie sich diese Alternativen qualitäts- und kostenmäßig (z.B. hinsichtlich der Bespielbarkeit und Lebensdauer) vergleichen lassen. Zudem müsste untersucht werden, ob und wie sich die Bespielbar-keit oder das Verletzungsrisiko der alternativ befüllten Kunststoffrasenflächen bei den verschiedenen Alternativfüllungen verändert (Plan Miljø Studie 2017). Es bedarf daher dringend weiterer wissenschaftlicher Expertise zur Praxistauglichkeit alternativer organischer Füllstoffe und zur sportartspezifischen Eignung von Kunststoffrasenplätzen, die ohne Füllstoffe auskommen. Sowohl eine wissenschaftliche Folgenabschätzung als auch die dringend erforderliche Entwicklung alternativer Füllstoffe durch die Industrie sind eine zentrale Forderung der von der Thematik betroffenen Sportverbände in Deutschland. Sie vertreten die Meinung, dass die Maßnahmen, die ein Verbot des Kunststoffgranulats verursachen würden, nicht kurzfristig umsetzbar sind und Alternativen nur mittel- bis langfristig erarbeitet und bereitgestellt werden können. | Gezielte Risikomanagementmaßnahmen können die Freisetzung von Füllstoffen in die Umwelt bereits signifikant vermindern. Technische Maßnahmen zur Zurückhaltung eines Materialaustrags vor Ort (z.B. Rinnenfilter mit Sedimentationsstrecken an Abläufen, Schmutzfangmatten, Schuhbürsten am Ausgang) und organisatorische Maßnahmen beim Betrieb der Sportplätze (z.B. regelmäßige Reinigung der Spielfeldränder, Auffangsiebe) können zu einer starken Verringerung des Austrags von Mikroplastik beitragen. |  |
| 2 | Berliner Fußball-Verband | a.: Nach neueren Erkenntnissen werden zwischen 0,25 t/a und 5 t/a in Deutschland an Befüllungsgranulat pro Kunststoffrasenfläche verwendet (Fraunhofer 2018, S. 11). Das entspricht einer Gesamtmenge von ca. 7.500 bis 9.900 t/a.  d.: In Deutschland gibt es ca. 5.000 für den Fußballspielbetrieb gemeldete Kunststoffrasenplätze (DFBnet), sowie ca. 1.000 DFB-Minispielfelder. Jährlich werden in Deutschland ca. 300 Kunststoffrasenplätze neu gebaut, sowie 150 Kunststoffrasenplätze von Grund auf erneuert. Hinsichtlich der bestehenden Plätze dürfte eine Umstellung auf alternative Füllstoffe notwendig sein. Hierfür halten die Sportanlagenbetreiber (Kommunen oder Vereine) Mittel für Sportstättenbau und -sanierung vor, die bei einem vollständigen Ver-bot und einer Verwendung alternativer Füllstoffe deutlich höher ausfallen würden. Laut eigener Berechnungen belaufen sich die jährlichen Mehrkosten deutschlandweit auf einen hohen einstelligen Millionenbetrag. Die insgesamt zu erwartenden Kosten eines Verbotes können aufgrund fehlender Kenntnisse über geeignete alternative Füllstoffe (Geeignetheit, Verfügbarkeit) derzeit nicht seriös beziffert werden. Auf Grundlage aktueller Daten zum Bau von Kunststoffrasenplätzen dürfte der Gesamtbetrag für den Austausch des Füllstoffes der Kunststoffrasensysteme im hohen zweistelligen Millionenbereich (bis zu 90 Mio. EUR) liegen, wobei zur Präzisierung dieses Schätzwertes vertiefte Analysen erforderlich sind. Die Kosten für eine Umsetzung gezielter Risikomanagementmaßnahmen zur Zurückhaltung des Materialaustrags dürften nach Schätzungen und je nach Umfang der Maßnahmen pro Kunststoffrasensystem bei 3.000 bis 10.000 EUR liegen.  e.: Der gemeinwohlorientierte Sport ist die größte zivilgesellschaftliche Bewegung in Deutschland und Europa. In Deutschland engagieren sich knapp acht Millionen Bürger freiwillig und ehrenamtlich im Sport. Das entspricht einer jährlichen Wertschöpfung und einem Wohlfahrtsgewinn allein in Deutschland von ca. 6,7 Milliarden Euro. Vergleichbare Zahlen lassen sich auch für die gesamte EU feststellen. In den EU-Mitgliedstaaten engagieren sich im Jahre 2010 zwischen 92 und 94 Millionen Menschen freiwillig für Ziele des Gemeinwohls, davon die meisten im Sport (ca. 35 bis 40 Prozent aller freiwillig Tätigen in der EU) (Europäische Kommission 2010).  Der Sport schafft ein strukturiertes, an die gesamte Bevölkerung gerichtetes und für alle offenes Bewegungs- und Sportangebot, durch das wichtige soziale und gesundheitsfördernde Funktionen in der Gesellschaft erfüllt werden. Sportvereine in Deutschland zählen zehn Millionen Mitgliedschaften im Kinder- und Jugendalter (DOSB-Bestandserhebung 2018), allein im DFB liegt diese Zahl bei 2,1 Millionen (DFB-Mitgliederstatistik 2018). Damit sind Sportvereine die wichtigste Anlaufstelle für Kinder und Jugendliche außerhalb der Schule und übernehmen unverzichtbare Aufgaben für die ganzheitliche Persönlichkeitsbildung junger Menschen. Dem Sport kommt eine wichtige Vorbild- und Lehrfunktion im Bereich der Integration und demokratischen Grundbildung zu. Für das herausragende gesellschaftliche Engagement des Sports spricht nicht zuletzt, dass die Sportvereine eng mit Schulen, Kindergärten, Unternehmen, Krankenkassen oder anderen öffentlichen Institutionen zusammenarbeiten. Um allen Bürgern den Zugang zum Sport zu ermöglichen, sind adäquate Sportstätten in ausreichender Anzahl Grundvoraussetzung. Ein für alle zugängliches und umfangreiches Sportangebot ist – vor allen Dingen in Großstädten und Ballungsgebieten – nur durch die Verfügbarkeit von ganzjährig nutzbaren Sportanlagen zu gewährleisten. Kunststoffrasenplätze spielen hierbei, insbesondere für den Fußball, eine wichtige Rolle, da sie eine intensivere Nutzung als Naturrasen- oder Tennenplätze erlauben. Allein mit Naturrasen- und Tennenplätzen lässt sich der derzeitige Trainings- und Spielbetrieb, insbesondere bei den Kinder- und Jugendmannschaften, nicht aufrechterhalten. Ein Kunststoffrasenplatz ersetzt etwa 2,5 Naturrasenplätze (DFBnet). Auf weniger als 10 Prozent der Naturrasenplätze finden an Wochenenden mehr als 2 Spiele statt. Bei Kunststoffrasenplätzen finden hingegen bei über 40 Prozent der Plätze mehr als 2 Spiele statt. Weniger als 10 Prozent der Naturrasenplätze wird an einem Wochenende mehr als 150 Minuten genutzt. Bei Kunstrasenplätzen werden hingegen etwa 35 Prozent an einem Wochenende mehr als 150 Minuten genutzt. 27.773 Spielstätten in Deutschland (ca. 70 Prozent) werden von Sportvereinen genutzt. Ein Drittel der Kunstrasenplätze werden von 2 oder mehr Vereinen mit alle ihren Jugend- und Seniorenmannschaften benutzt. Etwas über ein Drittel aller Naturrasenplätze wird von mehr als 5 Mannschaften bespielt. Bei Kunstrasenplätzen werden fast drei Viertel (72 Prozent) von mehr als 5 Mannschaften genutzt. Etwa 10 Prozent aller Naturrasenplätze wird von mehr als 10 Mannschaften bespielt. Bei Kunststoffrasenplätzen sind es ca. 41 Prozent der Plätze, die von mehr als 10 Mannschaften genutzt. Nur 1 Prozent aller Naturrasenplätze wird von mehr als 15 Mannschaften bespielt. Bei Kunstrasenplätzen beträgt der Anteil immerhin noch knapp 18 Prozent. Etwa 6 Prozent werden sogar von über 20 Mannschaften bespielt. Je größer die Vereinsgröße (insbesondere Anzahl der Mannschaften), desto höher ist der Anteil der Vereine, die auch eine Spielstätte vom Typ Kunstrasen haben.  Ein Verbot des Inverkehrbringens von Kunststoffgranulaten als Füllstoff in Kunststoffrasensystemen direkt bei Inkrafttreten der Beschränkung wäre daher unverhältnismäßig. Es würde zu hohen, unerwarteten Umstellungskosten und Mehrkosten für Vereine und Kommunen führen, wodurch dem gemeinwohlorientierten Sport Mittel entzogen würden. Bei fehlender Finanzierbarkeit dieser Mehrkosten ist zudem von einer Schließung vieler Sportplätzen auszugehen, wodurch das Sportangebot in Schulen und Vereinen stark leiden würde. Gerade auf Vereinsebene stellt ein solch außerordentlicher Kosten-punkt ein großes finanzielles Risiko dar, dass das sportliche und gesellschaftliche Gesamtangebot des Vereins gefährden kann. Eine Beschränkung ohne Übergangsfristen, die eine mittelfristige Umstellung und Kostenstreckung erlauben, würde das Breitensportangebot in Deutschland sehr negativ beeinflussen. Im Hinblick auf den Beschränkungsvorschlag der ECHA gemäß Anhang XV der REACH-Verordnung spricht sich der DFB daher für eine angemessene Übergangsfrist von mindestens sechs Jahren bis zu einem vollständigen Inverkehrbringungsverbot des Kunststoffgranulats zur Verwendung in neuen Kunststoffrasensystemen sowie für die Umstellung bestehender Flächen aus. |  | c.: Gezielte Risikomanagementmaßnahmen können die Freisetzung von Füllstoffen in die Umwelt bereits signifikant vermindern. Technische Maßnahmen zur Zurückhaltung eines Materialaustrags vor Ort (z.B. Rinnenfilter mit Sedimentationsstrecken an Abläufen, Schmutzfangmatten, Schuhbürsten am Ausgang) und organisatorische Maßnahmen beim Betrieb der Sportplätze (z.B. regelmäßige Reinigung der Spielfeldränder, Auffangsiebe) können zu einer starken Verringerung des Austrags von Mikroplastik beitragen.  Neben dem häufig genutzten Kunststoffgranulat existieren für Kunststoffrasensysteme alternative Füllstoffe, die in Teilen auch bereits beim Betrieb von Sportanlagen genutzt werden. So werden in Deutschland aktuell Kunststoffrasenplätze teilweise mit Sand und/oder Kork verfüllt. Zudem gibt es auch Kunststoffrasensysteme, die ohne elastischen Füllstoff betrieben werden können.  Es existieren bisher allerdings nur wenige belastbare Studien darüber, wie sich diese Alternativen qualitäts- und kostenmäßig (z.B. hinsichtlich der Bespielbarkeit und Lebensdauer) vergleichen lassen. Zudem müsste untersucht werden, ob und wie sich die Bespielbar-keit oder das Verletzungsrisiko der alternativ befüllten Kunststoffrasenflächen bei den verschiedenen Alternativfüllungen verändert (Plan Miljø Studie 2017). Es bedarf daher dringend weiterer wissenschaftlicher Expertise zur Praxistauglichkeit alternativer organischer Füllstoffe und zur sportartspezifischen Eignung von Kunststoffrasenplätzen, die ohne Füllstoffe auskommen. Sowohl eine wissenschaftliche Folgenabschätzung als auch die dringend erforderliche Entwicklung alternativer Füllstoffe durch die Industrie sind eine zentrale Forderung der von der Thematik betroffenen Sportverbände in Deutschland. Sie vertreten die Meinung, dass die Maßnahmen, die ein Verbot des Kunststoffgranulats verursachen würden, nicht kurzfristig umsetzbar sind und Alternativen nur mittel- bis langfristig erarbeitet und bereitgestellt werden können. | b.: Nach dem aktuellen Forschungsstand besteht nach Kenntnis des DFB ein hohes Maß an Unsicherheit darüber, wie und in welchen Mengen das als Mikroplastik definierte Granulat auf Sportplätzen in die Umwelt freigesetzt wird. Nach den uns zur Verfügung stehen-den Informationen gibt es große Unterschiede bei der Einschätzung der Menge an Mikroplastiken, die in den einzelnen Mitgliedstaaten oder in der EU/EWR als Füllmaterial für Kunstrasen verwendet wird. Insbesondere Umfang und Methodologie der Forschung in diesem Bereich sind bisher noch wenig standardisiert und nachvollziehbar. Der DFB geht davon aus, dass der Anteil des Eintrags von Mikroplastik über Kunststoffrasenplätze je nach Mitgliedstaat ca. 1 bis 3 Prozent im Verhältnis zum Gesamteintrag beträgt. Demnach ist der Umwelteintrag verglichen mit anderen Hauptquellen relativ gering (Europäische Kommission 2018, ii)). |  |
| 3 | Breast Cancer UK | Breast Cancer UK is a charity which aims to prevent breast cancer by promoting a healthy lifestyle and reducing public exposure to carcinogenic and other hazardous chemicals in the environment. In particular we are concerned about the potential role of exposures to environmental chemicals in increasing breast cancer risk. We consider microplastics to be potentially harmful to human health and the environment. We believe their presence in the environment may increase breast cancer risk, due to the potential for these particles to release harmful additives and to accumulate and release other substances of concern.  We welcome ECHA’s Annex XV proposal to restrict intentionally added microplastics in consumer and professional products and are grateful for the opportunity to respond to the proposal. Breast Cancer UK supports restricting the use of intentionally added microplastic particles in products of any kind.  We are especially concerned about chemical “additives” present in microplastics (e.g. plasticisers and compounds used in manufacture such as bisphenols), and the potential for microplastics to act as “vectors” for environmental pollutants; these substances may be transferred to marine and other organisms, following ingestion of microplastics (1, 2). Marine species include those regularly consumed by humans, such as mussels and oysters, as well as endangered species such as humpback dolphins.  Reducing significantly microlitter pollution in marine, freshwater and terrestrial environments should be a priority, given the potential harm this type of pollution causes. Although we appreciate microplastics may also arise from degradation of macroplastics, a ban on the use of added microplastics in products of any type would be one step towards reducing environmental pollution.  1. Carbery, M. et al. (2018). Environment International 115:400-409  2. Rochman, C. M. et al. (2013). Scientific Reports 3: 3263.  3. Zhu, J. et al. (2019). Science of the total Environment 659: 649-654.  4. Karlsson, T. M. et al. (2018). Marine Pollution Bulletin 129: 52-60  5. Toussain, B. et al. (2019). Food Additives & Contaminants Part A 36(5): 639-673.  6. Brody et al. (2018). Environmental research 160: 152-182  7. Pastor-Barriuso, R. et al. (2016). Environmental Health Perspectives 124 (10): 1575-1582  8. Lu, K et al. (2018). Chemosphere 202: 514-520.  9. Mezynska, M. and Brzóska, M. M. (2018). Environmental Science and Pollution Research 25: 3211–3232.  10. Lares et al. (2018). Water Research 133: 236-246.  11. Talvitie, J. et al. (2018). Water Research 109: 164-172.  12. Feng, L.-J. et al. (2018). Environmental Pollution 238: 859-865.  13. Kalčíková, G. et al. (2018). Chemosphere 188: 25-31.  Dr Margaret Wexler  Head of Science, Breast Cancer UK  margaret.wexler@breastcanceruk.org.uk |  | Studies have found common persistent organic pollutants can be up to 10 million times higher in plastic pellets than in sea water (4). As well as being potentially detrimental to the health of marine organisms and birds, microplastics and associated environmental pollutants have the potential to be passed up the food chain. A recent study by the European Commission’s Joint Research Centre (5) highlights the presence of micro and nano-plastics in animals and food products and concludes “There is a growing concern about the impact of human activities on the whole life chain, and there is a legitimate concern that the smaller plastic fraction, through bioaccumulation and trophic transfer, may ultimately contaminate the human population”.  Many chemical additives that leach from microplastics, such as bisphenols, heavy metals and phthalates, are endocrine disrupting chemicals (EDCs), which can affect the function of the hormone system. In particular EDCs which act as oestrogen mimics are associated with increased breast cancer risk (6). Endocrine disrupting chemicals may exert their effects at very low doses, and it is becoming increasingly apparent that environmental exposures to mixtures of such chemicals may be especially harmful (7).  Studies have shown microplastics may enhance toxicity (as well as bioaccumulation) of heavy metals in fish. For example, the presence of microplastics enhanced the toxicity and bioaccumulation of cadmium in zebrafish (8), causing oxidative damage and inflammation. Environmental exposure to cadmium (which is an EDC), is a risk factor for breast cancer (9).  Recent studies show that microplastics are not removed fully from wastewater treatment plants (WWTPs). The activated sludge process (the most common type of sewage treatment used globally) has a retention capacity of up to around 98-99% (10, 11), with most of the microplastics remaining within the activated sludge solids. Despite this, WWTPs remain point sources for microplastics (and nanoplastics) discharge, due to the high volume of effluent that is released constantly.  Microplastics may have a negative impact on the activated sludge treatment process itself; a recent study found respiration of activated sludge flocs was acutely inhibited by the presence of polystyrene nanoplastics (12), due to a change in composition of the extracellular polymeric substance (EPS) that surrounds activated sludge microorganisms and is integral to floc formation. Such changes will affect sludge settling and reduced respiration will affect the ability of activated sludge microorganisms to biodegrade pollutants. Another concern is the presence of microplastics (containing environmental pollutants) in the activated sludge solids that are removed and used commonly as land fertiliser (following appropriate treatment). One study which examined the fate of polyethylene microbeads from cosmetics using a laboratory scale bioreactor run to simulate an activated sludge WWTP found approximately half the microbeads were captured in the activated sludge (13). Other studies (cited above) suggest that most of the microplastics that enter an activated sludge WWTP will end up in the excess sludge solids. | As stated in ECHA’s background document, intentionally added microplastics can be released into the environment during the use of these products (typically via wastewater), potentially contributing to environmental litter and leading to concerns that their use may pose a risk to the environment and/or human health. |  |
| 4 | Deutscher Fussball-Bund e.V. | a.: Nach neueren Erkenntnissen werden zwischen 0,25 t/a und 5 t/a in Deutschland an Befüllungsgranulat pro Kunststoffrasenfläche verwendet (Fraunhofer 2018, S. 11). Das entspricht einer Gesamtmenge von ca. 7.500 bis 9.900 t/a.  b.: Nach dem aktuellen Forschungsstand besteht nach Kenntnis des DFB ein hohes Maß an Unsicherheit darüber, wie und in welchen Mengen das als Mikroplastik definierte Granulat auf Sportplätzen in die Umwelt freigesetzt wird. Nach den uns zur Verfügung stehen-den Informationen gibt es große Unterschiede bei der Einschätzung der Menge an Mikroplastik, die in den einzelnen Mitgliedstaaten oder in der EU/EWR als Füllmaterial für Kunstrasen verwendet wird. Insbesondere Umfang und Methodologie der Forschung in diesem Bereich sind bisher noch wenig standardisiert und nachvollziehbar. Der DFB geht davon aus, dass der Anteil des Eintrags von Mikroplastik über Kunststoffrasenplätze je nach Mitgliedstaat ca. 1 bis 3 Prozent im Verhältnis zum Gesamteintrag beträgt. Demnach ist der Umwelteintrag verglichen mit anderen Hauptquellen relativ gering (Europäische Kommission 2018, ii)).  d.: In Deutschland gibt es ca. 5.000 für den Fußballspielbetrieb gemeldete Kunststoffrasenplätze (DFBnet), sowie ca. 1.000 DFB-Minispielfelder. Jährlich werden in Deutschland ca. 300 Kunststoffrasenplätze neu gebaut, sowie 150 Kunststoffrasenplätze von Grund auf erneuert. Hinsichtlich der bestehenden Plätze dürfte eine Umstellung auf alternative Füllstoffe notwendig sein. Hierfür halten die Sportanlagenbetreiber (Kommunen oder Vereine) Mittel für Sportstättenbau und -sanierung vor, die bei einem vollständigen Ver-bot und einer Verwendung alternativer Füllstoffe deutlich höher ausfallen würden. Laut eigener Berechnungen belaufen sich die jährlichen Mehrkosten deutschlandweit auf einen hohen einstelligen Millionenbetrag. Die insgesamt zu erwartenden Kosten eines Verbotes können aufgrund fehlender Kenntnisse über geeignete alternative Füllstoffe (Geeignetheit, Verfügbarkeit) derzeit nicht seriös beziffert werden. Auf Grundlage aktueller Daten zum Bau von Kunststoffrasenplätzen dürfte der Gesamtbetrag für den Austausch des Füllstoffes der Kunststoffrasensysteme im hohen zweistelligen Millionenbereich (bis zu 90 Mio. EUR) liegen, wobei zur Präzisierung dieses Schätzwertes vertiefte Analysen erforderlich sind. Die Kosten für eine Umsetzung gezielter Risikomanagementmaßnahmen zur Zurückhaltung des Materialaustrags dürften nach Schätzungen und je nach Umfang der Maßnahmen pro Kunststoffrasensystem bei 3.000 bis 10.000 EUR liegen.  e.: Der gemeinwohlorientierte Sport ist die größte zivilgesellschaftliche Bewegung in Deutschland und Europa. In Deutschland engagieren sich knapp acht Millionen Bürger freiwillig und ehrenamtlich im Sport. Das entspricht einer jährlichen Wertschöpfung und einem Wohlfahrtsgewinn allein in Deutschland von ca. 6,7 Milliarden Euro. Vergleichbare Zahlen lassen sich auch für die gesamte EU feststellen. In den EU-Mitgliedstaaten engagieren sich im Jahre 2010 zwischen 92 und 94 Millionen Menschen freiwillig für Ziele des Gemeinwohls, davon die meisten im Sport (ca. 35 bis 40 Prozent aller freiwillig Tätigen in der EU) (Europäische Kommission 2010).  Der Sport schafft ein strukturiertes, an die gesamte Bevölkerung gerichtetes und für alle offenes Bewegungs- und Sportangebot, durch das wichtige soziale und gesundheitsfördernde Funktionen in der Gesellschaft erfüllt werden. Sportvereine in Deutschland zählen zehn Millionen Mitgliedschaften im Kinder- und Jugendalter (DOSB-Bestandserhebung 2018), allein im DFB liegt diese Zahl bei 2,1 Millionen (DFB-Mitgliederstatistik 2018). Damit sind Sportvereine die wichtigste Anlaufstelle für Kinder und Jugendliche außerhalb der Schule und übernehmen unverzichtbare Aufgaben für die ganzheitliche Persönlichkeitsbildung junger Menschen. Dem Sport kommt eine wichtige Vorbild- und Lehrfunktion im Bereich der Integration und demokratischen Grundbildung zu. Für das herausragende gesellschaftliche Engagement des Sports spricht nicht zuletzt, dass die Sportvereine eng mit Schulen, Kindergärten, Unternehmen, Krankenkassen oder anderen öffentlichen Institutionen zusammenarbeiten. Um allen Bürgern den Zugang zum Sport zu ermöglichen, sind adäquate Sportstätten in ausreichender Anzahl Grundvoraussetzung. Ein für alle zugängliches und umfangreiches Sportangebot ist – vor allen Dingen in Großstädten und Ballungsgebieten – nur durch die Verfügbarkeit von ganzjährig nutzbaren Sportanlagen zu gewährleisten. Kunststoffrasenplätze spielen hierbei, insbesondere für den Fußball, eine wichtige Rolle, da sie eine intensivere Nutzung als Naturrasen- oder Tennenplätze erlauben. Allein mit Naturrasen- und Tennenplätzen lässt sich der derzeitige Trainings- und Spielbetrieb, insbesondere bei den Kinder- und Jugendmannschaften, nicht aufrechterhalten. Ein Kunststoffrasenplatz ersetzt etwa 2,5 Naturrasenplätze (DFBnet). Auf weniger als 10 Prozent der Naturrasenplätze finden an Wochenenden mehr als 2 Spiele statt. Bei Kunststoffrasenplätzen finden hingegen bei über 40 Prozent der Plätze mehr als 2 Spiele statt. Weniger als 10 Prozent der Naturrasenplätze wird an einem Wochenende mehr als 150 Minuten genutzt. Bei Kunstrasenplätzen werden hingegen etwa 35 Prozent an einem Wochenende mehr als 150 Minuten genutzt. 27.773 Spielstätten in Deutschland (ca. 70 Prozent) werden von Sportvereinen genutzt. Ein Drittel der Kunstrasenplätze werden von 2 oder mehr Vereinen mit alle ihren Jugend- und Seniorenmannschaften benutzt. Etwas über ein Drittel aller Naturrasenplätze wird von mehr als 5 Mannschaften bespielt. Bei Kunstrasenplätzen werden fast drei Viertel (72 Prozent) von mehr als 5 Mannschaften genutzt. Etwa 10 Prozent aller Naturrasenplätze wird von mehr als 10 Mannschaften bespielt. Bei Kunststoffrasenplätzen sind es ca. 41 Prozent der Plätze, die von mehr als 10 Mannschaften genutzt. Nur 1 Prozent aller Naturrasenplätze wird von mehr als 15 Mannschaften bespielt. Bei Kunstrasenplätzen beträgt der Anteil immerhin noch knapp 18 Prozent. Etwa 6 Prozent werden sogar von über 20 Mannschaften bespielt. Je größer die Vereinsgröße (insbesondere Anzahl der Mannschaften), desto höher ist der Anteil der Vereine, die auch eine Spielstätte vom Typ Kunstrasen haben.  Ein Verbot des Inverkehrbringens von Kunststoffgranulaten als Füllstoff in Kunststoffrasensystemen direkt bei Inkrafttreten der Beschränkung wäre daher unverhältnismäßig. Es würde zu hohen, unerwarteten Umstellungskosten und Mehrkosten für Vereine und Kommunen führen, wodurch dem gemeinwohlorientierten Sport Mittel entzogen würden. Bei fehlender Finanzierbarkeit dieser Mehrkosten ist zudem von einer Schließung vieler Sportplätzen auszugehen, wodurch das Sportangebot in Schulen und Vereinen stark leiden würde. Gerade auf Vereinsebene stellt ein solch außerordentlicher Kosten-punkt ein großes finanzielles Risiko dar, dass das sportliche und gesellschaftliche Gesamtangebot des Vereins gefährden kann. Eine Beschränkung ohne Übergangsfristen, die eine mittelfristige Umstellung und Kostenstreckung erlauben, würde das Breitensportangebot in Deutschland sehr negativ beeinflussen.  Im Hinblick auf den Beschränkungsvorschlag der ECHA gemäß Anhang XV der REACH-Verordnung spricht sich der DFB daher für eine angemessene Übergangsfrist von mindestens sechs Jahren bis zu einem vollständigen Inverkehrbringungsverbot des Kunststoffgranulats zur Verwendung in neuen Kunststoffrasensystemen sowie für die Umstellung bestehender Flächen aus. |  | c.: Gezielte Risikomanagementmaßnahmen können die Freisetzung von Füllstoffen in die Umwelt bereits signifikant vermindern. Technische Maßnahmen zur Zurückhaltung eines Materialaustrags vor Ort (z.B. Rinnenfilter mit Sedimentationsstrecken an Abläufen, Schmutzfangmatten, Schuhbürsten am Ausgang) und organisatorische Maßnahmen beim Betrieb der Sportplätze (z.B. regelmäßige Reinigung der Spielfeldränder, Auffangsiebe) können zu einer starken Verringerung des Austrags von Mikroplastik beitragen.  Neben dem häufig genutzten Kunststoffgranulat existieren für Kunststoffrasensysteme alternative Füllstoffe, die in Teilen auch bereits beim Betrieb von Sportanlagen genutzt werden. So werden in Deutschland aktuell Kunststoffrasenplätze teilweise mit Sand und/oder Kork verfüllt. Zudem gibt es auch Kunststoffrasensysteme, die ohne elastischen Füllstoff betrieben werden können.  Es existieren bisher allerdings nur wenige belastbare Studien darüber, wie sich diese Alternativen qualitäts- und kostenmäßig (z.B. hinsichtlich der Bespielbarkeit und Lebensdauer) vergleichen lassen. Zudem müsste untersucht werden, ob und wie sich die Bespielbarkeit oder das Verletzungsrisiko der alternativ befüllten Kunststoffrasenflächen bei den verschiedenen Alternativfüllungen verändert (Plan Miljø Studie 2017). Es bedarf daher dringend weiterer wissenschaftlicher Expertise zur Praxistauglichkeit alternativer organischer Füllstoffe und zur sportartspezifischen Eignung von Kunststoffrasenplätzen, die ohne Füllstoffe auskommen. Sowohl eine wissenschaftliche Folgenabschätzung als auch die dringend erforderliche Entwicklung alternativer Füllstoffe durch die Industrie sind eine zentrale Forderung der von der Thematik betroffenen Sportverbände in Deutschland. Sie vertreten die Meinung, dass die Maßnahmen, die ein Verbot des Kunststoffgranulats verursachen würden, nicht kurzfristig umsetzbar sind und Alternativen nur mittel- bis langfristig erarbeitet und bereitgestellt werden können. |  |  |
| 5/6 | Deutscher Olympischer Sportbund e.V. (DOSB, German Olympic Sports Confederation) & DFB | D)  In Germany, there are approx. 5,000 artificial turf pitches registered for league and match use, as well as approx. 1,000 “mini-pitches. The German Olympic Sport Organisation (DOSB) is furthermore aware of a large number of other artificial turf pitches used for sports purposes. An exact quantification of the sports facilities affected by a possible restriction in Germany is currently not possible as a reliable data basis is lacking.  About 300 new artificial turf pitches are built in Germany every year, and 150 new artificial turf pitches are completely reconstructed. Regarding these pitches, a conversion to alternative infill may be necessary. Sports facility operators (municipalities or clubs) foresee funds for these construction and renovation measures of sports facilities, however, the financial investment needed will be significantly higher if a restriction is imposed and alternative infill materials have to be used. According to calculations by the German Football Federation (Deutscher Fußballbund, DFB), the annual additional costs throughout Germany would amount to a high single-digit million sum.  In addition to these already planned construction measures, a restriction would also affect all other artificial turf pitches filled with plastic granulate, as sports facility operators would no longer be able to acquire the required infill for regular re-filling. As a result, sports facility operators would incur costs for changing pitches that they had not budgeted for.  The costs of a restriction can currently not be quantified reliably due to a lack of knowledge about suitable alternative infill materials. On the basis of current data on the construction of artificial turf pitches, the total amount for the replacement of the infill material in the plastic turf systems in Germany is likely to be in the high double-digit million range.  E)  Not-for-profit sport is the largest civil society movement in Germany and Europe. It creates a structured sports offer that is open to the all of society and through which important social and health-promoting functions are fulfilled. Sports clubs in Germany have 10 million memberships in children and young people. Sports clubs are thus the most important contact point for children and young people outside school and assume indispensable tasks for the holistic personal development of young people.  In Germany, almost 8 million citizens are involved in sport on a voluntary basis. This makes sport the largest civil movement in Germany. Volunteers in sport perform a total of around 446 million voluntary, socially significant and unpaid working hours each year in a variety of functions. This amount of work corresponds to an annual added value and thus a welfare gain of approx. 6.7 billion euros in Germany alone. Comparable figures can also be established for the entire EU. In the EU Member States, between 92 and 94 million people volunteer for public welfare goals in 2010, most of them in sport (about 35 to 40 percent of all volunteers in the EU).  By involving large sections of the population in the daily work of sports clubs, it is possible to create and maintain a comprehensive, broad and accessible range of sports offers for all ages and levels of society. Sport also plays an important role in the field of integration and democratic basic education. In the German sports clubs alone, 2.6 million people with a migration background are socially integrated through sport.  Adequate sports facilities in sufficient numbers are a prerequisite for ensuring that all citizens have access to sport. For the organisation of sporting offers and the exercise of the social roles of sport, clubs are largely dependent on publicly financed sports facilities, but they also provide adequate sports facilities through their own investments. Attractively priced offers ensure that all sections of the population have access to sport. A comprehensive range of sports facilities accessible to all - especially in large cities and agglomerations - can only be guaranteed through the availability of sports facilities that can be used all year round. Artificial turf pitches play an important role in this regard, especially for football, as they allow more intensive use than turf or cinder pitches. It can be assumed that turf and cinder pitches alone will not be sufficient to maintain the current training and play facilities.  A restriction of plastic granulates as infill with the entry into force of the restriction would therefore be disproportionate, as it would lead to high, unforeseen conversion costs for clubs and municipalities, thereby depriving the not-for-profit sport of funds. If these additional costs cannot be financed, many sports fields are also in danger of being closed, which would severely affect the sporting offer available in schools and clubs. At club level in particular, such an extraordinary cost represents a major financial risk that could jeopardise the sports and social offerings of the club as a whole. A restriction without transition periods that would allow a medium-term changeover and cost extension, would therefore have a very negative impact on the not-for-profit sports offer in Germany.  German sports therefore advocates for an appropriate transitional period of at least six years until a complete ban on placing granular infill on the market for use in new plastic turf systems and the conversion of existing surfaces. Due to the high social relevance of sport, it is necessary to develop a common and step-by-step approach in order to appropriately balance the reduction of the discharge of microplastics with the need to practise sport among broad sections of the population.  Called for a 6-years transition period before the proposed EU ban on intentionally added microplastics in synthetic turf is enforced (Oziel, 2019). |  | Targeted risk management measures can already significantly reduce the release of rubber granulate infill into the environment. Technical measures for the retention of material on site (e.g. gutter filters with sedimentation sections at drains, dirt trap mats, shoe brushes at the exit) and organisational measures for the operation of the sports fields (e.g. regular cleaning of the field edges, collecting sieves) can contribute to a significant reduction in the release of infill material. | **Answer to specific info request 2:**  A) and B)  For the German Olympic Sport Confederation (DOSB) the currently available data is highly insufficient, as no data, risk analyses and impact assessments are available for the released quantities of infill or the effects of a restriction on the availability of sports facilities. The development of a better knowledge base should therefore be a first step, before ECHA imposes a direct restriction. Further scientific studies are needed in this complex area in order to close knowledge gaps and to develop more environmentally friendly materials for sports field construction and to enable an overall assessment of existing plastic turf systems based on sustainability criteria.  C)  Apart from the predominantly used plastic granulate, alternative filling materials exist for plastic turf systems, some of which are already used in the operation of sports facilities. In Germany, some plastic turf pitches are currently filled with sand and/or cork. There are also plastic turf systems that can be operated without an elastic filler. However, so far there are only few reliable studies on how these alternatives compare in terms of quality and cost (e.g. in terms of playability and service life).  In addition, it would have to be investigated whether and how the playability or the injury risk changes with the various alternative fillings. There is therefore an urgent need for further scientific expertise on the practical suitability of alternative, organic fillers and on the sports-specific suitability of plastic turf pitches that do not require fillers. Both a scientific impact assessment and the urgently needed development of alternative fillers by industry are central demands of the non-profit sports associations in Germany affected by the topic. They are of the opinion that the measures that would result in a ban on plastic granulate cannot be implemented in the short term and that alternatives can only be developed and made available in the medium to long term. |  |
| 7 | Ellen McArthur Foundation | Jocelyn Blériot, Ellen MacArthur Foundation: “*Significant evidence indicates that oxo-degradable plastics do not simply break down and become innocuous, but instead fragment into tiny pieces. As such, they contribute to microplastic pollution, posing a risk to the ocean and other ecosystems, potentially for decades to come – whether we see it or not*” (Blériot, 2017). |  |  |  |  |
| 8 | Fidra, an environmental charity based in Scotland | “Up to 195,000 tonnes of pre-production pellets – the small lentil-sized building blocks the plastic industry uses to make everything from bottles to car dashboards – leak into the oceans every year. It's one of the easier taps to turn off, according to Fidra, an environmental charity based in Scotland that is working on the issue at national and EU-level.  "To stop this source, all key players along the supply chain need to act, not just the proportion that have done so to date," said Sarah Archer, senior projects manager.” (Burrows, 2017)  **Content:**  Scope or restriction option analysis;  Hazard or exposure;  Environmental emissions;  Information on alternatives;  Information on benefits;  Other socio economic analysis (SEA) issues;  Transitional period |  |  |  |  |
| 9 | Football Federation Saxony-Anhalt | a.: Nach neueren Erkenntnissen werden zwischen 0,25 t/a und 5 t/a in Deutschland an Befüllungsgranulat pro Kunststoffrasenfläche verwendet (Fraunhofer 2018, S. 11). Das entspricht einer Gesamtmenge von ca. 7.500 bis 9.900 t/a.  b.: Nach dem aktuellen Forschungsstand besteht nach Kenntnis des DFB ein hohes Maß an Unsicherheit darüber, wie und in welchen Mengen das als Mikroplastik definierte Granulat auf Sportplätzen in die Umwelt freigesetzt wird. Nach den uns zur Verfügung stehen-den Informationen gibt es große Unterschiede bei der Einschätzung der Menge an Mikroplastiken, die in den einzelnen Mitgliedstaaten oder in der EU/EWR als Füllmaterial für Kunstrasen verwendet wird. Insbesondere Umfang und Methodologie der Forschung in diesem Bereich sind bisher noch wenig standardisiert und nachvollziehbar. Der DFB geht davon aus, dass der Anteil des Eintrags von Mikroplastik über Kunststoffrasenplätze je nach Mitgliedstaat ca. 1 bis 3 Prozent im Verhältnis zum Gesamteintrag beträgt. Demnach ist der Umwelteintrag verglichen mit anderen Hauptquellen relativ gering (Europäische Kommission 2018, ii)).  d.: In Deutschland gibt es ca. 5.000 für den Fußballspielbetrieb gemeldete Kunststoffrasenplätze (DFBnet), sowie ca. 1.000 DFB-Minispielfelder. Jährlich werden in Deutschland ca. 300 Kunststoffrasenplätze neu gebaut, sowie 150 Kunststoffrasenplätze von Grund auf erneuert. Hinsichtlich der bestehenden Plätze dürfte eine Umstellung auf alternative Füllstoffe notwendig sein. Hierfür halten die Sportanlagenbetreiber (Kommunen oder Vereine) Mittel für Sportstättenbau und -sanierung vor, die bei einem vollständigen Ver-bot und einer Verwendung alternativer Füllstoffe deutlich höher ausfallen würden. Laut eigener Berechnungen belaufen sich die jährlichen Mehrkosten deutschlandweit auf einen hohen einstelligen Millionenbetrag. Die insgesamt zu erwartenden Kosten eines Verbotes können aufgrund fehlender Kenntnisse über geeignete alternative Füllstoffe (Geeignetheit, Verfügbarkeit) derzeit nicht seriös beziffert werden. Auf Grundlage aktueller Daten zum Bau von Kunststoffrasenplätzen dürfte der Gesamtbetrag für den Austausch des Füllstoffes der Kunststoffrasensysteme im hohen zweistelligen Millionenbereich (bis zu 90 Mio. EUR) liegen, wobei zur Präzisierung dieses Schätzwertes vertiefte Analysen erforderlich sind. Die Kosten für eine Umsetzung gezielter Risikomanagementmaßnahmen zur Zurückhaltung des Materialaustrags dürften nach Schätzungen und je nach Umfang der Maßnahmen pro Kunststoffrasensystem bei 3.000 bis 10.000 EUR liegen.  e.: Der gemeinwohlorientierte Sport ist die größte zivilgesellschaftliche Bewegung in Deutschland und Europa. Im Fußballverband Sachsen-Anhalt engagieren sich knapp 19.000 Bürger freiwillig und ehrenamtlich im organisierten Fußball für rund 91.000 Mitglieder in über 790 Sportvereinen.  Der Sport schafft ein strukturiertes, an die gesamte Bevölkerung gerichtetes und für alle offenes Bewegungs- und Sportangebot, durch das wichtige soziale und gesundheitsfördernde Funktionen in der Gesellschaft erfüllt werden. Fußballvereine in Sachsen-Anhalt zählen rund 41.000 Mitgliedschaften im Kinder- und Jugendalter. Damit sind die Fußballvereine die wichtigste Anlaufstelle für Kinder und Jugendliche außerhalb der Schule und übernehmen unverzichtbare Aufgaben für die ganzheitliche Persönlichkeitsbildung junger Menschen. Dem Sport kommt eine wichtige Vorbild- und Lehrfunktion im Bereich der Integration und demokratischen Grundbildung zu. Für das herausragende gesellschaftliche Engagement des Sports spricht nicht zuletzt, dass die Sportvereine eng mit Schulen, Kindergärten, Unternehmen, Krankenkassen oder anderen öffentlichen Institutionen zusammenarbeiten. Um allen Bürgern den Zugang zum Sport zu ermöglichen, sind adäquate Sportstätten in ausreichender Anzahl Grundvoraussetzung. Ein für alle zugängliches und umfangreiches Sportangebot ist nur durch die Verfügbarkeit von ganzjährig nutzbaren Sportanlagen zu gewährleisten. Kunststoffrasenplätze spielen hierbei, insbesondere für den Fußball, eine wichtige Rolle, da sie eine intensivere Nutzung als Naturrasen- oder Tennenplätze erlauben. Allein mit Naturrasen- und Tennenplätzen lässt sich der derzeitige Trainings- und Spielbetrieb, insbesondere bei den Kinder- und Jugendmannschaften, nicht aufrechterhalten.  Im Fußball in Sachsen-Anhalt gibt es rund 81 Sportanlagen (zum Teil mit mehreren Spielfeldern) an denen für den Fußball und (meist überlappend) Hockey Kunststoffrasenplätze vorhanden sind. Insgesamt nutzen Fußball- und die 5 Hockeyvereine insgesamt knapp 650 Sportplätze. Die circa 13 % Kunststoffrasenplätze befinden sich schwerpunktmäßig in den beiden Großstädten Magdeburg und Halle (Saale). Hier wäre ein Trainings- und Wettkampfbetrieb etlicher Vereine ohne diese Anlagen undenkbar. An diesen Standorten nutzen die Anlagen nicht nur mehrere Fußballvereine gleichzeitig sondern teilen sich die Sportarten Fußball und Hockey auch den Sportplatz.  Ungeachtet der Tatsache, dass ohne diese Plätze der Trainings- und Wettkampfbedarf nicht auch nur annährend bedient werden könnte, wäre es ebenso unvorstellbar diese Stunden auf neu zu errichtende Naturrasenplätze umzulagern, da die hohe Stundenzahl der Nutzung auf dem Naturbelag nicht möglich ist.  Ein Verbot des Inverkehrbringens von Kunststoffgranulaten als Füllstoff in Kunststoffrasensystemen direkt bei Inkrafttreten der Beschränkung wäre daher unverhältnismäßig. Es würde zu hohen, unerwarteten Umstellungskosten und Mehrkosten für Vereine und Kommunen führen, wodurch dem gemeinwohlorientierten Sport Mittel entzogen würden. Bei fehlender Finanzierbarkeit dieser Mehrkosten ist zudem von einer Schließung vieler Sportplätzen auszugehen, wodurch das Sportangebot in Schulen und Vereinen stark leiden würde. Gerade auf Vereinsebene stellt ein solch außerordentlicher Kostenpunkt ein großes finanzielles Risiko dar, dass das sportliche und gesellschaftliche Gesamtangebot des Vereins gefährden würde. Eine Beschränkung ohne Übergangsfristen, die eine mittelfristige Umstellung und Kostenstreckung erlauben, würde das Sportangebot in Sachsen-Anhalt sehr negativ beeinflussen.  Im Hinblick auf den Beschränkungsvorschlag der ECHA gemäß Anhang XV der REACH-Verordnung spricht sich der LandesSportBund Sachsen-Anhalt daher für eine angemessene Übergangsfrist von mindestens sechs Jahren bis zu einem vollständigen Inverkehrbringungsverbot des Kunststoffgranulats zur Verwendung in neuen Kunststoffrasensystemen sowie für die Umstellung bestehender Flächen aus. |  | c.: Gezielte Risikomanagementmaßnahmen können die Freisetzung von Füllstoffen in die Umwelt bereits signifikant vermindern. Technische Maßnahmen zur Zurückhaltung eines Materialaustrags vor Ort (z.B. Rinnenfilter mit Sedimentationsstrecken an Abläufen, Schmutzfangmatten, Schuhbürsten am Ausgang) und organisatorische Maßnahmen beim Betrieb der Sportplätze (z.B. regelmäßige Reinigung der Spielfeldränder, Auffangsiebe) können zu einer starken Verringerung des Austrags von Mikroplastik beitragen.  Neben dem häufig genutzten Kunststoffgranulat existieren für Kunststoffrasensysteme alternative Füllstoffe, die in Teilen auch bereits beim Betrieb von Sportanlagen genutzt werden. So werden in Deutschland aktuell Kunststoffrasenplätze teilweise mit Sand und/oder Kork verfüllt. Zudem gibt es auch Kunststoffrasensysteme, die ohne elastischen Füllstoff betrieben werden können.  Es existieren bisher allerdings nur wenige belastbare Studien darüber, wie sich diese Alternativen qualitäts- und kostenmäßig (z.B. hinsichtlich der Bespielbarkeit und Lebensdauer) vergleichen lassen. Zudem müsste untersucht werden, ob und wie sich die Bespielbar-keit oder das Verletzungsrisiko der alternativ befüllten Kunststoffrasenflächen bei den verschiedenen Alternativfüllungen verändert (Plan Miljø Studie 2017). Es bedarf daher dringend weiterer wissenschaftlicher Expertise zur Praxistauglichkeit alternativer organischer Füllstoffe und zur sportartspezifischen Eignung von Kunststoffrasenplätzen, die ohne Füllstoffe auskommen. Sowohl eine wissenschaftliche Folgenabschätzung als auch die dringend erforderliche Entwicklung alternativer Füllstoffe durch die Industrie sind eine zentrale Forderung der von der Thematik betroffenen Sportverbände in Deutschland. Sie vertreten die Meinung, dass die Maßnahmen, die ein Verbot des Kunststoffgranulats verursachen würden, nicht kurzfristig umsetzbar sind und Alternativen nur mittel- bis langfristig erarbeitet und bereitgestellt werden können. |  |  |
| 10 | Fußball und Leichtathletik-Verband Westfalen e.V. | a.: Nach neueren Erkenntnissen werden zwischen 0,25 t/a und 5 t/a in Deutschland an Befüllungsgranulat pro Kunststoffrasenfläche verwendet (Fraunhofer 2018, S. 11). Das entspricht einer Gesamtmenge von ca. 7.500 bis 9.900 t/a.  b.: Nach dem aktuellen Forschungsstand besteht nach Kenntnis des DFB ein hohes Maß an Unsicherheit darüber, wie und in welchen Mengen das als Mikroplastik definierte Granulat auf Sportplätzen in die Umwelt freigesetzt wird. Nach den uns zur Verfügung stehen-den Informationen gibt es große Unterschiede bei der Einschätzung der Menge an Mikroplastiken, die in den einzelnen Mitgliedstaaten oder in der EU/EWR als Füllmaterial für Kunstrasen verwendet wird. Insbesondere Umfang und Methodologie der Forschung in diesem Bereich sind bisher noch wenig standardisiert und nachvollziehbar. Der DFB geht davon aus, dass der Anteil des Eintrags von Mikroplastik über Kunststoffrasenplätze je nach Mitgliedstaat ca. 1 bis 3 Prozent im Verhältnis zum Gesamteintrag beträgt. Demnach ist der Umwelteintrag verglichen mit anderen Hauptquellen relativ gering (Europäische Kommission 2018, ii)).  d.: In Deutschland gibt es ca. 5.000 für den Fußballspielbetrieb gemeldete Kunststoffrasenplätze (DFBnet), sowie ca. 1.000 DFB-Minispielfelder. Jährlich werden in Deutschland ca. 300 Kunststoffrasenplätze neu gebaut, sowie 150 Kunststoffrasenplätze von Grund auf erneuert. Hinsichtlich der bestehenden Plätze dürfte eine Umstellung auf alternative Füllstoffe notwendig sein. Hierfür halten die Sportanlagenbetreiber (Kommunen oder Vereine) Mittel für Sportstättenbau und -sanierung vor, die bei einem vollständigen Ver-bot und einer Verwendung alternativer Füllstoffe deutlich höher ausfallen würden. Laut eigener Berechnungen belaufen sich die jährlichen Mehrkosten deutschlandweit auf einen hohen einstelligen Millionenbetrag. Die insgesamt zu erwartenden Kosten eines Verbotes können aufgrund fehlender Kenntnisse über geeignete alternative Füllstoffe (Geeignetheit, Verfügbarkeit) derzeit nicht seriös beziffert werden. Auf Grundlage aktueller Daten zum Bau von Kunststoffrasenplätzen dürfte der Gesamtbetrag für den Austausch des Füllstoffes der Kunststoffrasensysteme im hohen zweistelligen Millionenbereich (bis zu 90 Mio. EUR) liegen, wobei zur Präzisierung dieses Schätzwertes vertiefte Analysen erforderlich sind. Die Kosten für eine Umsetzung gezielter Risikomanagementmaßnahmen zur Zurückhaltung des Materialaustrags dürften nach Schätzungen und je nach Umfang der Maßnahmen pro Kunststoffrasensystem bei 3.000 bis 10.000 EUR liegen.  e.: Der gemeinwohlorientierte Sport ist die größte zivilgesellschaftliche Bewegung in Deutschland und Europa. In Deutschland engagieren sich knapp acht Millionen Bürger freiwillig und ehrenamtlich im Sport. Das entspricht einer jährlichen Wertschöpfung und einem Wohlfahrtsgewinn allein in Deutschland von ca. 6,7 Milliarden Euro. Vergleichbare Zahlen lassen sich auch für die gesamte EU feststellen. In den EU-Mitgliedstaaten engagieren sich im Jahre 2010 zwischen 92 und 94 Millionen Menschen freiwillig für Ziele des Gemeinwohls, davon die meisten im Sport (ca. 35 bis 40 Prozent aller freiwillig Tätigen in der EU) (Europäische Kommission 2010).  Der Sport schafft ein strukturiertes, an die gesamte Bevölkerung gerichtetes und für alle offenes Bewegungs- und Sportangebot, durch das wichtige soziale und gesundheitsfördernde Funktionen in der Gesellschaft erfüllt werden. Sportvereine in Deutschland zählen zehn Millionen Mitgliedschaften im Kinder- und Jugendalter (DOSB-Bestandserhebung 2018), allein im DFB liegt diese Zahl bei 2,1 Millionen (DFB-Mitgliederstatistik 2018). Damit sind Sportvereine die wichtigste Anlaufstelle für Kinder und Jugendliche außerhalb der Schule und übernehmen unverzichtbare Aufgaben für die ganzheitliche Persönlichkeitsbildung junger Menschen. Dem Sport kommt eine wichtige Vorbild- und Lehrfunktion im Bereich der Integration und demokratischen Grundbildung zu. Für das herausragende gesellschaftliche Engagement des Sports spricht nicht zuletzt, dass die Sportvereine eng mit Schulen, Kindergärten, Unternehmen, Krankenkassen oder anderen öffentlichen Institutionen zusammenarbeiten. Um allen Bürgern den Zugang zum Sport zu ermöglichen, sind adäquate Sportstätten in ausreichender Anzahl Grundvoraussetzung. Ein für alle zugängliches und umfangreiches Sportangebot ist – vor allen Dingen in Großstädten und Ballungsgebieten – nur durch die Verfügbarkeit von ganzjährig nutzbaren Sportanlagen zu gewährleisten. Kunststoffrasenplätze spielen hierbei, insbesondere für den Fußball, eine wichtige Rolle, da sie eine intensivere Nutzung als Naturrasen- oder Tennenplätze erlauben. Allein mit Naturrasen- und Tennenplätzen lässt sich der derzeitige Trainings- und Spielbetrieb, insbesondere bei den Kinder- und Jugendmannschaften, nicht aufrechterhalten. Ein Kunststoffrasenplatz ersetzt etwa 2,5 Naturrasenplätze (DFBnet). Auf weniger als 10 Prozent der Naturrasenplätze finden an Wochenenden mehr als 2 Spiele statt. Bei Kunststoffrasenplätzen finden hingegen bei über 40 Prozent der Plätze mehr als 2 Spiele statt. Weniger als 10 Prozent der Naturrasenplätze wird an einem Wochenende mehr als 150 Minuten genutzt. Bei Kunstrasenplätzen werden hingegen etwa 35 Prozent an einem Wochenende mehr als 150 Minuten genutzt. 27.773 Spielstätten in Deutschland (ca. 70 Prozent) werden von Sportvereinen genutzt. Ein Drittel der Kunstrasenplätze werden von 2 oder mehr Vereinen mit alle ihren Jugend- und Seniorenmannschaften benutzt. Etwas über ein Drittel aller Naturrasenplätze wird von mehr als 5 Mannschaften bespielt. Bei Kunstrasenplätzen werden fast drei Viertel (72 Prozent) von mehr als 5 Mannschaften genutzt. Etwa 10 Prozent aller Naturrasenplätze wird von mehr als 10 Mannschaften bespielt. Bei Kunststoffrasenplätzen sind es ca. 41 Prozent der Plätze, die von mehr als 10 Mannschaften genutzt. Nur 1 Prozent aller Naturrasenplätze wird von mehr als 15 Mannschaften bespielt. Bei Kunstrasenplätzen beträgt der Anteil immerhin noch knapp 18 Prozent. Etwa 6 Prozent werden sogar von über 20 Mannschaften bespielt. Je größer die Vereinsgröße (insbesondere Anzahl der Mannschaften), desto höher ist der Anteil der Vereine, die auch eine Spielstätte vom Typ Kunstrasen haben.  Ein Verbot des Inverkehrbringens von Kunststoffgranulaten als Füllstoff in Kunststoffrasensystemen direkt bei Inkrafttreten der Beschränkung wäre daher unverhältnismäßig. Es würde zu hohen, unerwarteten Umstellungskosten und Mehrkosten für Vereine und Kommunen führen, wodurch dem gemeinwohlorientierten Sport Mittel entzogen würden. Bei fehlender Finanzierbarkeit dieser Mehrkosten ist zudem von einer Schließung vieler Sportplätzen auszugehen, wodurch das Sportangebot in Schulen und Vereinen stark leiden würde. Gerade auf Vereinsebene stellt ein solch außerordentlicher Kosten-punkt ein großes finanzielles Risiko dar, dass das sportliche und gesellschaftliche Gesamtangebot des Vereins gefährden kann. Eine Beschränkung ohne Übergangsfristen, die eine mittelfristige Umstellung und Kostenstreckung erlauben, würde das Breitensportangebot in Deutschland sehr negativ beeinflussen.  Im Hinblick auf den Beschränkungsvorschlag der ECHA gemäß Anhang XV der REACH-Verordnung spricht sich der DFB daher für eine angemessene Übergangsfrist von mindestens sechs Jahren bis zu einem vollständigen Inverkehrbringungsverbot des Kunststoffgranulats zur Verwendung in neuen Kunststoffrasensystemen sowie für die Umstellung bestehender Flächen aus. |  | c.: Gezielte Risikomanagementmaßnahmen können die Freisetzung von Füllstoffen in die Umwelt bereits signifikant vermindern. Technische Maßnahmen zur Zurückhaltung eines Materialaustrags vor Ort (z.B. Rinnenfilter mit Sedimentationsstrecken an Abläufen, Schmutzfangmatten, Schuhbürsten am Ausgang) und organisatorische Maßnahmen beim Betrieb der Sportplätze (z.B. regelmäßige Reinigung der Spielfeldränder, Auffangsiebe) können zu einer starken Verringerung des Austrags von Mikroplastik beitragen.  Neben dem häufig genutzten Kunststoffgranulat existieren für Kunststoffrasensysteme alternative Füllstoffe, die in Teilen auch bereits beim Betrieb von Sportanlagen genutzt werden. So werden in Deutschland aktuell Kunststoffrasenplätze teilweise mit Sand und/oder Kork verfüllt. Zudem gibt es auch Kunststoffrasensysteme, die ohne elastischen Füllstoff betrieben werden können.  Es existieren bisher allerdings nur wenige belastbare Studien darüber, wie sich diese Alternativen qualitäts- und kostenmäßig (z.B. hinsichtlich der Bespielbarkeit und Lebensdauer) vergleichen lassen. Zudem müsste untersucht werden, ob und wie sich die Bespielbar-keit oder das Verletzungsrisiko der alternativ befüllten Kunststoffrasenflächen bei den verschiedenen Alternativfüllungen verändert (Plan Miljø Studie 2017). Es bedarf daher dringend weiterer wissenschaftlicher Expertise zur Praxistauglichkeit alternativer organischer Füllstoffe und zur sportartspezifischen Eignung von Kunststoffrasenplätzen, die ohne Füllstoffe auskommen. Sowohl eine wissenschaftliche Folgenabschätzung als auch die dringend erforderliche Entwicklung alternativer Füllstoffe durch die Industrie sind eine zentrale Forderung der von der Thematik betroffenen Sportverbände in Deutschland. Sie vertreten die Meinung, dass die Maßnahmen, die ein Verbot des Kunststoffgranulats verursachen würden, nicht kurzfristig umsetzbar sind und Alternativen nur mittel- bis langfristig erarbeitet und bereitgestellt werden können. |  |  |
| 11 | Fußball-Verband Mittelrhein e.V | **Answer to specific info request 2:**  a.: Nach neueren Erkenntnissen werden zwischen 0,25 t/a und 5 t/a in Deutschland an Befüllungsgranulat pro Kunststoffrasenfläche verwendet (Fraunhofer 2018, S. 11). Das entspricht einer Gesamtmenge von ca. 7.500 bis 9.900 t/a.  b.: Nach dem aktuellen Forschungsstand besteht nach Kenntnis des DFB ein hohes Maß an Unsicherheit darüber, wie und in welchen Mengen das als Mikroplastik definierte Granulat auf Sportplätzen in die Umwelt freigesetzt wird. Nach den uns zur Verfügung stehen-den Informationen gibt es große Unterschiede bei der Einschätzung der Menge an Mikroplastiken, die in den einzelnen Mitgliedstaaten oder in der EU/EWR als Füllmaterial für Kunstrasen verwendet wird. Insbesondere Umfang und Methodologie der Forschung in diesem Bereich sind bisher noch wenig standardisiert und nachvollziehbar. Der DFB geht davon aus, dass der Anteil des Eintrags von Mikroplastik über Kunststoffrasenplätze je nach Mitgliedstaat ca. 1 bis 3 Prozent im Verhältnis zum Gesamteintrag beträgt. Demnach ist der Umwelteintrag verglichen mit anderen Hauptquellen relativ gering (Europäische Kommission 2018, ii)). |  | c.: Gezielte Risikomanagementmaßnahmen können die Freisetzung von Füllstoffen in die Umwelt bereits signifikant vermindern. Technische Maßnahmen zur Zurückhaltung eines Materialaustrags vor Ort (z.B. Rinnenfilter mit Sedimentationsstrecken an Abläufen, Schmutzfangmatten, Schuhbürsten am Ausgang) und organisatorische Maßnahmen beim Betrieb der Sportplätze (z.B. regelmäßige Reinigung der Spielfeldränder, Auffangsiebe) können zu einer starken Verringerung des Austrags von Mikroplastik beitragen.  Neben dem häufig genutzten Kunststoffgranulat existieren für Kunststoffrasensysteme alternative Füllstoffe, die in Teilen auch bereits beim Betrieb von Sportanlagen genutzt werden. So werden in Deutschland aktuell Kunststoffrasenplätze teilweise mit Sand und/oder Kork verfüllt. Zudem gibt es auch Kunststoffrasensysteme, die ohne elastischen Füllstoff betrieben werden können.  Es existieren bisher allerdings nur wenige belastbare Studien darüber, wie sich diese Alternativen qualitäts- und kostenmäßig (z.B. hinsichtlich der Bespielbarkeit und Lebensdauer) vergleichen lassen. Zudem müsste untersucht werden, ob und wie sich die Bespielbar-keit oder das Verletzungsrisiko der alternativ befüllten Kunststoffrasenflächen bei den verschiedenen Alternativfüllungen verändert (Plan Miljø Studie 2017). Es bedarf daher dringend weiterer wissenschaftlicher Expertise zur Praxistauglichkeit alternativer organischer Füllstoffe und zur sportartspezifischen Eignung von Kunststoffrasenplätzen, die ohne Füllstoffe auskommen. Sowohl eine wissenschaftliche Folgenabschätzung als auch die dringend erforderliche Entwicklung alternativer Füllstoffe durch die Industrie sind eine zentrale Forderung der von der Thematik betroffenen Sportverbände in Deutschland. Sie vertreten die Meinung, dass die Maßnahmen, die ein Verbot des Kunststoffgranulats verursachen würden, nicht kurzfristig umsetzbar sind und Alternativen nur mittel- bis langfristig erarbeitet und bereitgestellt werden können. |  | d.: In Deutschland gibt es ca. 5.000 für den Fußballspielbetrieb gemeldete Kunststoffrasenplätze (DFBnet), sowie ca. 1.000 DFB-Minispielfelder. Jährlich werden in Deutschland ca. 300 Kunststoffrasenplätze neu gebaut, sowie 150 Kunststoffrasenplätze von Grund auf erneuert. Hinsichtlich der bestehenden Plätze dürfte eine Umstellung auf alternative Füllstoffe notwendig sein. Hierfür halten die Sportanlagenbetreiber (Kommunen oder Vereine) Mittel für Sportstättenbau und -sanierung vor, die bei einem vollständigen Ver-bot und einer Verwendung alternativer Füllstoffe deutlich höher ausfallen würden. Laut eigener Berechnungen belaufen sich die jährlichen Mehrkosten deutschlandweit auf einen hohen einstelligen Millionenbetrag. Die insgesamt zu erwartenden Kosten eines Verbotes können aufgrund fehlender Kenntnisse über geeignete alternative Füllstoffe (Geeignetheit, Verfügbarkeit) derzeit nicht seriös beziffert werden. Auf Grundlage aktueller Daten zum Bau von Kunststoffrasenplätzen dürfte der Gesamtbetrag für den Austausch des Füllstoffes der Kunststoffrasensysteme im hohen zweistelligen Millionenbereich (bis zu 90 Mio. EUR) liegen, wobei zur Präzisierung dieses Schätzwertes vertiefte Analysen erforderlich sind. Die Kosten für eine Umsetzung gezielter Risikomanagementmaßnahmen zur Zurückhaltung des Materialaustrags dürften nach Schätzungen und je nach Umfang der Maßnahmen pro Kunststoffrasensystem bei 3.000 bis 10.000 EUR liegen.  e.: Der gemeinwohlorientierte Sport ist die größte zivilgesellschaftliche Bewegung in Deutschland und Europa. In Deutschland engagieren sich knapp acht Millionen Bürger freiwillig und ehrenamtlich im Sport. Das entspricht einer jährlichen Wertschöpfung und einem Wohlfahrtsgewinn allein in Deutschland von ca. 6,7 Milliarden Euro. Vergleichbare Zahlen lassen sich auch für die gesamte EU feststellen. In den EU-Mitgliedstaaten engagieren sich im Jahre 2010 zwischen 92 und 94 Millionen Menschen freiwillig für Ziele des Gemeinwohls, davon die meisten im Sport (ca. 35 bis 40 Prozent aller freiwillig Tätigen in der EU) (Europäische Kommission 2010).  Der Sport schafft ein strukturiertes, an die gesamte Bevölkerung gerichtetes und für alle offenes Bewegungs- und Sportangebot, durch das wichtige soziale und gesundheitsfördernde Funktionen in der Gesellschaft erfüllt werden. Sportvereine in Deutschland zählen zehn Millionen Mitgliedschaften im Kinder- und Jugendalter (DOSB-Bestandserhebung 2018), allein im DFB liegt diese Zahl bei 2,1 Millionen (DFB-Mitgliederstatistik 2018). Damit sind Sportvereine die wichtigste Anlaufstelle für Kinder und Jugendliche außerhalb der Schule und übernehmen unverzichtbare Aufgaben für die ganzheitliche Persönlichkeitsbildung junger Menschen. Dem Sport kommt eine wichtige Vorbild- und Lehrfunktion im Bereich der Integration und demokratischen Grundbildung zu. Für das herausragende gesellschaftliche Engagement des Sports spricht nicht zuletzt, dass die Sportvereine eng mit Schulen, Kindergärten, Unternehmen, Krankenkassen oder anderen öffentlichen Institutionen zusammenarbeiten. Um allen Bürgern den Zugang zum Sport zu ermöglichen, sind adäquate Sportstätten in ausreichender Anzahl Grundvoraussetzung. Ein für alle zugängliches und umfangreiches Sportangebot ist – vor allen Dingen in Großstädten und Ballungsgebieten – nur durch die Verfügbarkeit von ganzjährig nutzbaren Sportanlagen zu gewährleisten. Kunststoffrasenplätze spielen hierbei, insbesondere für den Fußball, eine wichtige Rolle, da sie eine intensivere Nutzung als Naturrasen- oder Tennenplätze erlauben. Allein mit Naturrasen- und Tennenplätzen lässt sich der derzeitige Trainings- und Spielbetrieb, insbesondere bei den Kinder- und Jugendmannschaften, nicht aufrechterhalten. Ein Kunststoffrasenplatz ersetzt etwa 2,5 Naturrasenplätze (DFBnet). Auf weniger als 10 Prozent der Naturrasenplätze finden an Wochenenden mehr als 2 Spiele statt. Bei Kunststoffrasenplätzen finden hingegen bei über 40 Prozent der Plätze mehr als 2 Spiele statt. Weniger als 10 Prozent der Naturrasenplätze wird an einem Wochenende mehr als 150 Minuten genutzt. Bei Kunstrasenplätzen werden hingegen etwa 35 Prozent an einem Wochenende mehr als 150 Minuten genutzt. 27.773 Spielstätten in Deutschland (ca. 70 Prozent) werden von Sportvereinen genutzt. Im Verbandsgebiet des FVM werden 360 Kunstrasenplätze genutzt. Ein Drittel der Kunstrasenplätze werden von 2 oder mehr Vereinen mit alle ihren Jugend- und Seniorenmannschaften benutzt. Etwas über ein Drittel aller Naturrasenplätze wird von mehr als 5 Mannschaften bespielt. Bei Kunstrasenplätzen werden fast drei Viertel (72 Prozent) von mehr als 5 Mannschaften genutzt. Etwa 10 Prozent aller Naturrasenplätze wird von mehr als 10 Mannschaften bespielt. Bei Kunststoffrasenplätzen sind es ca. 41 Prozent der Plätze, die von mehr als 10 Mannschaften genutzt. Nur 1 Prozent aller Naturrasenplätze wird von mehr als 15 Mannschaften bespielt. Bei Kunstrasenplätzen beträgt der Anteil immerhin noch knapp 18 Prozent. Etwa 6 Prozent werden sogar von über 20 Mannschaften bespielt. Je größer die Vereinsgröße (insbesondere Anzahl der Mannschaften), desto höher ist der Anteil der Vereine, die auch eine Spielstätte vom Typ Kunstrasen haben.  Ein Verbot des Inverkehrbringens von Kunststoffgranulaten als Füllstoff in Kunststoffrasensystemen direkt bei Inkrafttreten der Beschränkung wäre daher unverhältnismäßig. Es würde zu hohen, unerwarteten Umstellungskosten und Mehrkosten für Vereine und Kommunen führen, wodurch dem gemeinwohlorientierten Sport Mittel entzogen würden. Bei fehlender Finanzierbarkeit dieser Mehrkosten ist zudem von einer Schließung vieler Sportplätzen auszugehen, wodurch das Sportangebot in Schulen und Vereinen stark leiden würde. Gerade auf Vereinsebene stellt ein solch außerordentlicher Kosten-punkt ein großes finanzielles Risiko dar, dass das sportliche und gesellschaftliche Gesamtangebot des Vereins gefährden kann. Eine Beschränkung ohne Übergangsfristen, die eine mittelfristige Umstellung und Kostenstreckung erlauben, würde das Breitensportangebot in Deutschland sehr negativ beeinflussen.  Im Hinblick auf den Beschränkungsvorschlag der ECHA gemäß Anhang XV der REACH-Verordnung spricht sich der DFB daher für eine angemessene Übergangsfrist von mindestens sechs Jahren bis zu einem vollständigen Inverkehrbringungsverbot des Kunststoffgranulats zur Verwendung in neuen Kunststoffrasensystemen sowie für die Umstellung bestehender Flächen aus. |
| 12 | Fußballverband Niederrhein e.V. | **Answer to specific info request 2:**  a.: Nach neueren Erkenntnissen werden zwischen 0,25 t/a und 5 t/a in Deutschland an Befüllungsgranulat pro Kunststoffrasenfläche verwendet (Fraunhofer 2018, S. 11). Das entspricht einer Gesamtmenge von ca. 7.500 bis 9.900 t/a.  b.: Nach dem aktuellen Forschungsstand besteht nach Kenntnis des DFB ein hohes Maß an Unsicherheit darüber, wie und in welchen Mengen das als Mikroplastik definierte Granulat auf Sportplätzen in die Umwelt freigesetzt wird. Nach den uns zur Verfügung stehen-den Informationen gibt es große Unterschiede bei der Einschätzung der Menge an Mikroplastiken, die in den einzelnen Mitgliedstaaten oder in der EU/EWR als Füllmaterial für Kunstrasen verwendet wird. Insbesondere Umfang und Methodologie der Forschung in diesem Bereich sind bisher noch wenig standardisiert und nachvollziehbar. Der DFB geht davon aus, dass der Anteil des Eintrags von Mikroplastik über Kunststoffrasenplätze je nach Mitgliedstaat ca. 1 bis 3 Prozent im Verhältnis zum Gesamteintrag beträgt. Demnach ist der Umwelteintrag verglichen mit anderen Hauptquellen relativ gering (Europäische Kommission 2018, ii)). |  | c.: Gezielte Risikomanagementmaßnahmen können die Freisetzung von Füllstoffen in die Umwelt bereits signifikant vermindern. Technische Maßnahmen zur Zurückhaltung eines Materialaustrags vor Ort (z.B. Rinnenfilter mit Sedimentationsstrecken an Abläufen, Schmutzfangmatten, Schuhbürsten am Ausgang) und organisatorische Maßnahmen beim Betrieb der Sportplätze (z.B. regelmäßige Reinigung der Spielfeldränder, Auffangsiebe) können zu einer starken Verringerung des Austrags von Mikroplastik beitragen.  Neben dem häufig genutzten Kunststoffgranulat existieren für Kunststoffrasensysteme alternative Füllstoffe, die in Teilen auch bereits beim Betrieb von Sportanlagen genutzt werden. So werden in Deutschland aktuell Kunststoffrasenplätze teilweise mit Sand und/oder Kork verfüllt. Zudem gibt es auch Kunststoffrasensysteme, die ohne elastischen Füllstoff betrieben werden können.  Es existieren bisher allerdings nur wenige belastbare Studien darüber, wie sich diese Alternativen qualitäts- und kostenmäßig (z.B. hinsichtlich der Bespielbarkeit und Lebensdauer) vergleichen lassen. Zudem müsste untersucht werden, ob und wie sich die Bespielbar-keit oder das Verletzungsrisiko der alternativ befüllten Kunststoffrasenflächen bei den verschiedenen Alternativfüllungen verändert (Plan Miljø Studie 2017). Es bedarf daher dringend weiterer wissenschaftlicher Expertise zur Praxistauglichkeit alternativer organischer Füllstoffe und zur sportartspezifischen Eignung von Kunststoffrasenplätzen, die ohne Füllstoffe auskommen. Sowohl eine wissenschaftliche Folgenabschätzung als auch die dringend erforderliche Entwicklung alternativer Füllstoffe durch die Industrie sind eine zentrale Forderung der von der Thematik betroffenen Sportverbände in Deutschland. Sie vertreten die Meinung, dass die Maßnahmen, die ein Verbot des Kunststoffgranulats verursachen würden, nicht kurzfristig umsetzbar sind und Alternativen nur mittel- bis langfristig erarbeitet und bereitgestellt werden können. |  | d.: In Deutschland gibt es ca. 5.000 für den Fußballspielbetrieb gemeldete Kunststoffrasenplätze (DFBnet), sowie ca. 1.000 DFB-Minispielfelder. Jährlich werden in Deutschland ca. 300 Kunststoffrasenplätze neu gebaut, sowie 150 Kunststoffrasenplätze von Grund auf erneuert. Hinsichtlich der bestehenden Plätze dürfte eine Umstellung auf alternative Füllstoffe notwendig sein. Hierfür halten die Sportanlagenbetreiber (Kommunen oder Vereine) Mittel für Sportstättenbau und -sanierung vor, die bei einem vollständigen Ver-bot und einer Verwendung alternativer Füllstoffe deutlich höher ausfallen würden. Laut eigener Berechnungen belaufen sich die jährlichen Mehrkosten deutschlandweit auf einen hohen einstelligen Millionenbetrag. Die insgesamt zu erwartenden Kosten eines Verbotes können aufgrund fehlender Kenntnisse über geeignete alternative Füllstoffe (Geeignetheit, Verfügbarkeit) derzeit nicht seriös beziffert werden. Auf Grundlage aktueller Daten zum Bau von Kunststoffrasenplätzen dürfte der Gesamtbetrag für den Austausch des Füllstoffes der Kunststoffrasensysteme im hohen zweistelligen Millionenbereich (bis zu 90 Mio. EUR) liegen, wobei zur Präzisierung dieses Schätzwertes vertiefte Analysen erforderlich sind. Die Kosten für eine Umsetzung gezielter Risikomanagementmaßnahmen zur Zurückhaltung des Materialaustrags dürften nach Schätzungen und je nach Umfang der Maßnahmen pro Kunststoffrasensystem bei 3.000 bis 10.000 EUR liegen.  e.: Der gemeinwohlorientierte Sport ist die größte zivilgesellschaftliche Bewegung in Deutschland und Europa. In Deutschland engagieren sich knapp acht Millionen Bürger freiwillig und ehrenamtlich im Sport. Das entspricht einer jährlichen Wertschöpfung und einem Wohlfahrtsgewinn allein in Deutschland von ca. 6,7 Milliarden Euro. Vergleichbare Zahlen lassen sich auch für die gesamte EU feststellen. In den EU-Mitgliedstaaten engagieren sich im Jahre 2010 zwischen 92 und 94 Millionen Menschen freiwillig für Ziele des Gemeinwohls, davon die meisten im Sport (ca. 35 bis 40 Prozent aller freiwillig Tätigen in der EU) (Europäische Kommission 2010).  Der Sport schafft ein strukturiertes, an die gesamte Bevölkerung gerichtetes und für alle offenes Bewegungs- und Sportangebot, durch das wichtige soziale und gesundheitsfördernde Funktionen in der Gesellschaft erfüllt werden. Sportvereine in Deutschland zählen zehn Millionen Mitgliedschaften im Kinder- und Jugendalter (DOSB-Bestandserhebung 2018), allein im DFB liegt diese Zahl bei 2,1 Millionen (DFB-Mitgliederstatistik 2018). Damit sind Sportvereine die wichtigste Anlaufstelle für Kinder und Jugendliche außerhalb der Schule und übernehmen unverzichtbare Aufgaben für die ganzheitliche Persönlichkeitsbildung junger Menschen. Dem Sport kommt eine wichtige Vorbild- und Lehrfunktion im Bereich der Integration und demokratischen Grundbildung zu. Für das herausragende gesellschaftliche Engagement des Sports spricht nicht zuletzt, dass die Sportvereine eng mit Schulen, Kindergärten, Unternehmen, Krankenkassen oder anderen öffentlichen Institutionen zusammenarbeiten. Um allen Bürgern den Zugang zum Sport zu ermöglichen, sind adäquate Sportstätten in ausreichender Anzahl Grundvoraussetzung. Ein für alle zugängliches und umfangreiches Sportangebot ist – vor allen Dingen in Großstädten und Ballungsgebieten – nur durch die Verfügbarkeit von ganzjährig nutzbaren Sportanlagen zu gewährleisten. Kunststoffrasenplätze spielen hierbei, insbesondere für den Fußball, eine wichtige Rolle, da sie eine intensivere Nutzung als Naturrasen- oder Tennenplätze erlauben. Allein mit Naturrasen- und Tennenplätzen lässt sich der derzeitige Trainings- und Spielbetrieb, insbesondere bei den Kinder- und Jugendmannschaften, nicht aufrechterhalten. Ein Kunststoffrasenplatz ersetzt etwa 2,5 Naturrasenplätze (DFBnet). Auf weniger als 10 Prozent der Naturrasenplätze finden an Wochenenden mehr als 2 Spiele statt. Bei Kunststoffrasenplätzen finden hingegen bei über 40 Prozent der Plätze mehr als 2 Spiele statt. Weniger als 10 Prozent der Naturrasenplätze wird an einem Wochenende mehr als 150 Minuten genutzt. Bei Kunstrasenplätzen werden hingegen etwa 35 Prozent an einem Wochenende mehr als 150 Minuten genutzt. 27.773 Spielstätten in Deutschland (ca. 70 Prozent) werden von Sportvereinen genutzt. Ein Drittel der Kunstrasenplätze werden von 2 oder mehr Vereinen mit alle ihren Jugend- und Seniorenmannschaften benutzt. Etwas über ein Drittel aller Naturrasenplätze wird von mehr als 5 Mannschaften bespielt. Bei Kunstrasenplätzen werden fast drei Viertel (72 Prozent) von mehr als 5 Mannschaften genutzt. Etwa 10 Prozent aller Naturrasenplätze wird von mehr als 10 Mannschaften bespielt. Bei Kunststoffrasenplätzen sind es ca. 41 Prozent der Plätze, die von mehr als 10 Mannschaften genutzt. Nur 1 Prozent aller Naturrasenplätze wird von mehr als 15 Mannschaften bespielt. Bei Kunstrasenplätzen beträgt der Anteil immerhin noch knapp 18 Prozent. Etwa 6 Prozent werden sogar von über 20 Mannschaften bespielt. Je größer die Vereinsgröße (insbesondere Anzahl der Mannschaften), desto höher ist der Anteil der Vereine, die auch eine Spielstätte vom Typ Kunstrasen haben.  Ein Verbot des Inverkehrbringens von Kunststoffgranulaten als Füllstoff in Kunststoffrasensystemen direkt bei Inkrafttreten der Beschränkung wäre daher unverhältnismäßig. Es würde zu hohen, unerwarteten Umstellungskosten und Mehrkosten für Vereine und Kommunen führen, wodurch dem gemeinwohlorientierten Sport Mittel entzogen würden. Bei fehlender Finanzierbarkeit dieser Mehrkosten ist zudem von einer Schließung vieler Sportplätzen auszugehen, wodurch das Sportangebot in Schulen und Vereinen stark leiden würde. Gerade auf Vereinsebene stellt ein solch außerordentlicher Kosten-punkt ein großes finanzielles Risiko dar, dass das sportliche und gesellschaftliche Gesamtangebot des Vereins gefährden kann. Eine Beschränkung ohne Übergangsfristen, die eine mittelfristige Umstellung und Kostenstreckung erlauben, würde das Breitensportangebot in Deutschland sehr negativ beeinflussen.  Im Hinblick auf den Beschränkungsvorschlag der ECHA gemäß Anhang XV der REACH-Verordnung spricht sich der DFB daher für eine angemessene Übergangsfrist von mindestens sechs Jahren bis zu einem vollständigen Inverkehrbringungsverbot des Kunststoffgranulats zur Verwendung in neuen Kunststoffrasensystemen sowie für die Umstellung bestehender Flächen aus. |
| 13 | Fußballverband Rheinland | a.: Nach neueren Erkenntnissen werden zwischen 0,25 t/a und 5 t/a in Deutschland an Befüllungsgranulat pro Kunststoffrasenfläche verwendet (Fraunhofer 2018, S. 11). Das entspricht einer Gesamtmenge von ca. 7.500 bis 9.900 t/a.  b.: Nach dem aktuellen Forschungsstand besteht nach Kenntnis des DFB ein hohes Maß an Unsicherheit darüber, wie und in welchen Mengen das als Mikroplastik definierte Granulat auf Sportplätzen in die Umwelt freigesetzt wird. Nach den uns zur Verfügung stehen-den Informationen gibt es große Unterschiede bei der Einschätzung der Menge an Mikroplastiken, die in den einzelnen Mitgliedstaaten oder in der EU/EWR als Füllmaterial für Kunstrasen verwendet wird. Insbesondere Umfang und Methodologie der Forschung in diesem Bereich sind bisher noch wenig standardisiert und nachvollziehbar. Der DFB geht davon aus, dass der Anteil des Eintrags von Mikroplastik über Kunststoffrasenplätze je nach Mitgliedstaat ca. 1 bis 3 Prozent im Verhältnis zum Gesamteintrag beträgt. Demnach ist der Umwelteintrag verglichen mit anderen Hauptquellen relativ gering (Europäische Kommission 2018, ii)). |  | c.: Gezielte Risikomanagementmaßnahmen können die Freisetzung von Füllstoffen in die Umwelt bereits signifikant vermindern. Technische Maßnahmen zur Zurückhaltung eines Materialaustrags vor Ort (z.B. Rinnenfilter mit Sedimentationsstrecken an Abläufen, Schmutzfangmatten, Schuhbürsten am Ausgang) und organisatorische Maßnahmen beim Betrieb der Sportplätze (z.B. regelmäßige Reinigung der Spielfeldränder, Auffangsiebe) können zu einer starken Verringerung des Austrags von Mikroplastik beitragen.  Neben dem häufig genutzten Kunststoffgranulat existieren für Kunststoffrasensysteme alternative Füllstoffe, die in Teilen auch bereits beim Betrieb von Sportanlagen genutzt werden. So werden in Deutschland aktuell Kunststoffrasenplätze teilweise mit Sand und/oder Kork verfüllt. Zudem gibt es auch Kunststoffrasensysteme, die ohne elastischen Füllstoff betrieben werden können.  Es existieren bisher allerdings nur wenige belastbare Studien darüber, wie sich diese Alternativen qualitäts- und kostenmäßig (z.B. hinsichtlich der Bespielbarkeit und Lebensdauer) vergleichen lassen. Zudem müsste untersucht werden, ob und wie sich die Bespielbar-keit oder das Verletzungsrisiko der alternativ befüllten Kunststoffrasenflächen bei den verschiedenen Alternativfüllungen verändert (Plan Miljø Studie 2017). Es bedarf daher dringend weiterer wissenschaftlicher Expertise zur Praxistauglichkeit alternativer organischer Füllstoffe und zur sportartspezifischen Eignung von Kunststoffrasenplätzen, die ohne Füllstoffe auskommen. Sowohl eine wissenschaftliche Folgenabschätzung als auch die dringend erforderliche Entwicklung alternativer Füllstoffe durch die Industrie sind eine zentrale Forderung der von der Thematik betroffenen Sportverbände in Deutschland. Sie vertreten die Meinung, dass die Maßnahmen, die ein Verbot des Kunststoffgranulats verursachen würden, nicht kurzfristig umsetzbar sind und Alternativen nur mittel- bis langfristig erarbeitet und bereitgestellt werden können. |  | d.: In Deutschland gibt es ca. 5.000 für den Fußballspielbetrieb gemeldete Kunststoffrasenplätze (DFBnet), sowie ca. 1.000 DFB-Minispielfelder. Jährlich werden in Deutschland ca. 300 Kunststoffrasenplätze neu gebaut, sowie 150 Kunststoffrasenplätze von Grund auf erneuert. Hinsichtlich der bestehenden Plätze dürfte eine Umstellung auf alternative Füllstoffe notwendig sein. Hierfür halten die Sportanlagenbetreiber (Kommunen oder Vereine) Mittel für Sportstättenbau und -sanierung vor, die bei einem vollständigen Ver-bot und einer Verwendung alternativer Füllstoffe deutlich höher ausfallen würden. Laut eigener Berechnungen belaufen sich die jährlichen Mehrkosten deutschlandweit auf einen hohen einstelligen Millionenbetrag. Die insgesamt zu erwartenden Kosten eines Verbotes können aufgrund fehlender Kenntnisse über geeignete alternative Füllstoffe (Geeignetheit, Verfügbarkeit) derzeit nicht seriös beziffert werden. Auf Grundlage aktueller Daten zum Bau von Kunststoffrasenplätzen dürfte der Gesamtbetrag für den Austausch des Füllstoffes der Kunststoffrasensysteme im hohen zweistelligen Millionenbereich (bis zu 90 Mio. EUR) liegen, wobei zur Präzisierung dieses Schätzwertes vertiefte Analysen erforderlich sind. Die Kosten für eine Umsetzung gezielter Risikomanagementmaßnahmen zur Zurückhaltung des Materialaustrags dürften nach Schätzungen und je nach Umfang der Maßnahmen pro Kunststoffrasensystem bei 3.000 bis 10.000 EUR liegen.  e.: Der gemeinwohlorientierte Sport ist die größte zivilgesellschaftliche Bewegung in Deutschland und Europa. In Deutschland engagieren sich knapp acht Millionen Bürger freiwillig und ehrenamtlich im Sport. Das entspricht einer jährlichen Wertschöpfung und einem Wohlfahrtsgewinn allein in Deutschland von ca. 6,7 Milliarden Euro. Vergleichbare Zahlen lassen sich auch für die gesamte EU feststellen. In den EU-Mitgliedstaaten engagieren sich im Jahre 2010 zwischen 92 und 94 Millionen Menschen freiwillig für Ziele des Gemeinwohls, davon die meisten im Sport (ca. 35 bis 40 Prozent aller freiwillig Tätigen in der EU) (Europäische Kommission 2010).  Der Sport schafft ein strukturiertes, an die gesamte Bevölkerung gerichtetes und für alle offenes Bewegungs- und Sportangebot, durch das wichtige soziale und gesundheitsfördernde Funktionen in der Gesellschaft erfüllt werden. Sportvereine in Deutschland zählen zehn Millionen Mitgliedschaften im Kinder- und Jugendalter (DOSB-Bestandserhebung 2018), allein im DFB liegt diese Zahl bei 2,1 Millionen (DFB-Mitgliederstatistik 2018). Damit sind Sportvereine die wichtigste Anlaufstelle für Kinder und Jugendliche außerhalb der Schule und übernehmen unverzichtbare Aufgaben für die ganzheitliche Persönlichkeitsbildung junger Menschen. Dem Sport kommt eine wichtige Vorbild- und Lehrfunktion im Bereich der Integration und demokratischen Grundbildung zu. Für das herausragende gesellschaftliche Engagement des Sports spricht nicht zuletzt, dass die Sportvereine eng mit Schulen, Kindergärten, Unternehmen, Krankenkassen oder anderen öffentlichen Institutionen zusammenarbeiten. Um allen Bürgern den Zugang zum Sport zu ermöglichen, sind adäquate Sportstätten in ausreichender Anzahl Grundvoraussetzung. Ein für alle zugängliches und umfangreiches Sportangebot ist – vor allen Dingen in Großstädten und Ballungsgebieten – nur durch die Verfügbarkeit von ganzjährig nutzbaren Sportanlagen zu gewährleisten. Kunststoffrasenplätze spielen hierbei, insbesondere für den Fußball, eine wichtige Rolle, da sie eine intensivere Nutzung als Naturrasen- oder Tennenplätze erlauben. Allein mit Naturrasen- und Tennenplätzen lässt sich der derzeitige Trainings- und Spielbetrieb, insbesondere bei den Kinder- und Jugendmannschaften, nicht aufrechterhalten. Ein Kunststoffrasenplatz ersetzt etwa 2,5 Naturrasenplätze (DFBnet). Auf weniger als 10 Prozent der Naturrasenplätze finden an Wochenenden mehr als 2 Spiele statt. Bei Kunststoffrasenplätzen finden hingegen bei über 40 Prozent der Plätze mehr als 2 Spiele statt. Weniger als 10 Prozent der Naturrasenplätze wird an einem Wochenende mehr als 150 Minuten genutzt. Bei Kunstrasenplätzen werden hingegen etwa 35 Prozent an einem Wochenende mehr als 150 Minuten genutzt. 27.773 Spielstätten in Deutschland (ca. 70 Prozent) werden von Sportvereinen genutzt. Ein Drittel der Kunstrasenplätze werden von 2 oder mehr Vereinen mit alle ihren Jugend- und Seniorenmannschaften benutzt. Etwas über ein Drittel aller Naturrasenplätze wird von mehr als 5 Mannschaften bespielt. Bei Kunstrasenplätzen werden fast drei Viertel (72 Prozent) von mehr als 5 Mannschaften genutzt. Etwa 10 Prozent aller Naturrasenplätze wird von mehr als 10 Mannschaften bespielt. Bei Kunststoffrasenplätzen sind es ca. 41 Prozent der Plätze, die von mehr als 10 Mannschaften genutzt. Nur 1 Prozent aller Naturrasenplätze wird von mehr als 15 Mannschaften bespielt. Bei Kunstrasenplätzen beträgt der Anteil immerhin noch knapp 18 Prozent. Etwa 6 Prozent werden sogar von über 20 Mannschaften bespielt. Je größer die Vereinsgröße (insbesondere Anzahl der Mannschaften), desto höher ist der Anteil der Vereine, die auch eine Spielstätte vom Typ Kunstrasen haben.  Ein Verbot des Inverkehrbringens von Kunststoffgranulaten als Füllstoff in Kunststoffrasensystemen direkt bei Inkrafttreten der Beschränkung wäre daher unverhältnismäßig. Es würde zu hohen, unerwarteten Umstellungskosten und Mehrkosten für Vereine und Kommunen führen, wodurch dem gemeinwohlorientierten Sport Mittel entzogen würden. Bei fehlender Finanzierbarkeit dieser Mehrkosten ist zudem von einer Schließung vieler Sportplätzen auszugehen, wodurch das Sportangebot in Schulen und Vereinen stark leiden würde. Gerade auf Vereinsebene stellt ein solch außerordentlicher Kostenpunkt ein großes finanzielles Risiko dar, dass das sportliche und gesellschaftliche Gesamtangebot des Vereins gefährden kann. Eine Beschränkung ohne Übergangsfristen, die eine mittelfristige Umstellung und Kostenstreckung erlauben, würde das Breitensportangebot im Verbandsgebiet des Fußballverbandes Rheinland und sicher in ganz Deutschland sehr negativ beeinflussen.  Im Hinblick auf den Beschränkungsvorschlag der ECHA gemäß Anhang XV der REACH-Verordnung spricht sich der Fußballverbandes Rheinland daher für eine angemessene Übergangsfrist von mindestens sechs Jahren bis zu einem vollständigen Inverkehrbringungsverbot des Kunststoffgranulats zur Verwendung in neuen Kunststoffrasensystemen sowie für die Umstellung bestehender Flächen aus. |
| 14 | Germany Südwestdeutscher Fußballverband | a.: Nach neueren Erkenntnissen werden zwischen 0,25 t/a und 5 t/a in Deutschland an Befüllungsgranulat pro Kunststoffrasenfläche verwendet (Fraunhofer 2018, S. 11). Das entspricht einer Gesamtmenge von ca. 7.500 bis 9.900 t/a.  b.: Nach dem aktuellen Forschungsstand besteht nach Kenntnis des DFB ein hohes Maß an Unsicherheit darüber, wie und in welchen Mengen das als Mikroplastik definierte Granulat auf Sportplätzen in die Umwelt freigesetzt wird. Nach den uns zur Verfügung stehen-den Informationen gibt es große Unterschiede bei der Einschätzung der Menge an Mikroplastiken, die in den einzelnen Mitgliedstaaten oder in der EU/EWR als Füllmaterial für Kunstrasen verwendet wird. Insbesondere Umfang und Methodologie der Forschung in diesem Bereich sind bisher noch wenig standardisiert und nachvollziehbar. Der DFB geht davon aus, dass der Anteil des Eintrags von Mikroplastik über Kunststoffrasenplätze je nach Mitgliedstaat ca. 1 bis 3 Prozent im Verhältnis zum Gesamteintrag beträgt. Demnach ist der Umwelteintrag verglichen mit anderen Hauptquellen relativ gering (Europäische Kommission 2018, ii)). |  | c.: Gezielte Risikomanagementmaßnahmen können die Freisetzung von Füllstoffen in die Umwelt bereits signifikant vermindern. Technische Maßnahmen zur Zurückhaltung eines Materialaustrags vor Ort (z.B. Rinnenfilter mit Sedimentationsstrecken an Abläufen, Schmutzfangmatten, Schuhbürsten am Ausgang) und organisatorische Maßnahmen beim Betrieb der Sportplätze (z.B. regelmäßige Reinigung der Spielfeldränder, Auffangsiebe) können zu einer starken Verringerung des Austrags von Mikroplastik beitragen.  Neben dem häufig genutzten Kunststoffgranulat existieren für Kunststoffrasensysteme alternative Füllstoffe, die in Teilen auch bereits beim Betrieb von Sportanlagen genutzt werden. So werden in Deutschland aktuell Kunststoffrasenplätze teilweise mit Sand und/oder Kork verfüllt. Zudem gibt es auch Kunststoffrasensysteme, die ohne elastischen Füllstoff betrieben werden können.  Es existieren bisher allerdings nur wenige belastbare Studien darüber, wie sich diese Alternativen qualitäts- und kostenmäßig (z.B. hinsichtlich der Bespielbarkeit und Lebensdauer) vergleichen lassen. Zudem müsste untersucht werden, ob und wie sich die Bespielbar-keit oder das Verletzungsrisiko der alternativ befüllten Kunststoffrasenflächen bei den verschiedenen Alternativfüllungen verändert (Plan Miljø Studie 2017). Es bedarf daher dringend weiterer wissenschaftlicher Expertise zur Praxistauglichkeit alternativer organischer Füllstoffe und zur sportartspezifischen Eignung von Kunststoffrasenplätzen, die ohne Füllstoffe auskommen. Sowohl eine wissenschaftliche Folgenabschätzung als auch die dringend erforderliche Entwicklung alternativer Füllstoffe durch die Industrie sind eine zentrale Forderung der von der Thematik betroffenen Sportverbände in Deutschland. Sie vertreten die Meinung, dass die Maßnahmen, die ein Verbot des Kunststoffgranulats verursachen würden, nicht kurzfristig umsetzbar sind und Alternativen nur mittel- bis langfristig erarbeitet und bereitgestellt werden können. |  | d.: In Deutschland gibt es ca. 5.000 für den Fußballspielbetrieb gemeldete Kunststoffrasenplätze (DFBnet), sowie ca. 1.000 DFB-Minispielfelder. Jährlich werden in Deutschland ca. 300 Kunststoffrasenplätze neu gebaut, sowie 150 Kunststoffrasenplätze von Grund auf erneuert. Hinsichtlich der bestehenden Plätze dürfte eine Umstellung auf alternative Füllstoffe notwendig sein. Hierfür halten die Sportanlagenbetreiber (Kommunen oder Vereine) Mittel für Sportstättenbau und -sanierung vor, die bei einem vollständigen Ver-bot und einer Verwendung alternativer Füllstoffe deutlich höher ausfallen würden. Laut eigener Berechnungen belaufen sich die jährlichen Mehrkosten deutschlandweit auf einen hohen einstelligen Millionenbetrag. Die insgesamt zu erwartenden Kosten eines Verbotes können aufgrund fehlender Kenntnisse über geeignete alternative Füllstoffe (Geeignetheit, Verfügbarkeit) derzeit nicht seriös beziffert werden. Auf Grundlage aktueller Daten zum Bau von Kunststoffrasenplätzen dürfte der Gesamtbetrag für den Austausch des Füllstoffes der Kunststoffrasensysteme im hohen zweistelligen Millionenbereich (bis zu 90 Mio. EUR) liegen, wobei zur Präzisierung dieses Schätzwertes vertiefte Analysen erforderlich sind. Die Kosten für eine Umsetzung gezielter Risikomanagementmaßnahmen zur Zurückhaltung des Materialaustrags dürften nach Schätzungen und je nach Umfang der Maßnahmen pro Kunststoffrasensystem bei 3.000 bis 10.000 EUR liegen.  e.: Der gemeinwohlorientierte Sport ist die größte zivilgesellschaftliche Bewegung in Deutschland und Europa. In Deutschland engagieren sich knapp acht Millionen Bürger freiwillig und ehrenamtlich im Sport. Das entspricht einer jährlichen Wertschöpfung und einem Wohlfahrtsgewinn allein in Deutschland von ca. 6,7 Milliarden Euro. Vergleichbare Zahlen lassen sich auch für die gesamte EU feststellen. In den EU-Mitgliedstaaten engagieren sich im Jahre 2010 zwischen 92 und 94 Millionen Menschen freiwillig für Ziele des Gemeinwohls, davon die meisten im Sport (ca. 35 bis 40 Prozent aller freiwillig Tätigen in der EU) (Europäische Kommission 2010).  Der Sport schafft ein strukturiertes, an die gesamte Bevölkerung gerichtetes und für alle offenes Bewegungs- und Sportangebot, durch das wichtige soziale und gesundheitsfördernde Funktionen in der Gesellschaft erfüllt werden. Sportvereine in Deutschland zählen zehn Millionen Mitgliedschaften im Kinder- und Jugendalter (DOSB-Bestandserhebung 2018), allein im DFB liegt diese Zahl bei 2,1 Millionen (DFB-Mitgliederstatistik 2018). Damit sind Sportvereine die wichtigste Anlaufstelle für Kinder und Jugendliche außerhalb der Schule und übernehmen unverzichtbare Aufgaben für die ganzheitliche Persönlichkeitsbildung junger Menschen. Dem Sport kommt eine wichtige Vorbild- und Lehrfunktion im Bereich der Integration und demokratischen Grundbildung zu. Für das herausragende gesellschaftliche Engagement des Sports spricht nicht zuletzt, dass die Sportvereine eng mit Schulen, Kindergärten, Unternehmen, Krankenkassen oder anderen öffentlichen Institutionen zusammenarbeiten. Um allen Bürgern den Zugang zum Sport zu ermöglichen, sind adäquate Sportstätten in ausreichender Anzahl Grundvoraussetzung. Ein für alle zugängliches und umfangreiches Sportangebot ist – vor allen Dingen in Großstädten und Ballungsgebieten – nur durch die Verfügbarkeit von ganzjährig nutzbaren Sportanlagen zu gewährleisten. Kunststoffrasenplätze spielen hierbei, insbesondere für den Fußball, eine wichtige Rolle, da sie eine intensivere Nutzung als Naturrasen- oder Tennenplätze erlauben. Allein mit Naturrasen- und Tennenplätzen lässt sich der derzeitige Trainings- und Spielbetrieb, insbesondere bei den Kinder- und Jugendmannschaften, nicht aufrechterhalten. Ein Kunststoffrasenplatz ersetzt etwa 2,5 Naturrasenplätze (DFBnet). Auf weniger als 10 Prozent der Naturrasenplätze finden an Wochenenden mehr als 2 Spiele statt. Bei Kunststoffrasenplätzen finden hingegen bei über 40 Prozent der Plätze mehr als 2 Spiele statt. Weniger als 10 Prozent der Naturrasenplätze wird an einem Wochenende mehr als 150 Minuten genutzt. Bei Kunstrasenplätzen werden hingegen etwa 35 Prozent an einem Wochenende mehr als 150 Minuten genutzt. 27.773 Spielstätten in Deutschland (ca. 70 Prozent) werden von Sportvereinen genutzt. Ein Drittel der Kunstrasenplätze werden von 2 oder mehr Vereinen mit alle ihren Jugend- und Seniorenmannschaften benutzt. Etwas über ein Drittel aller Naturrasenplätze wird von mehr als 5 Mannschaften bespielt. Bei Kunstrasenplätzen werden fast drei Viertel (72 Prozent) von mehr als 5 Mannschaften genutzt. Etwa 10 Prozent aller Naturrasenplätze wird von mehr als 10 Mannschaften bespielt. Bei Kunststoffrasenplätzen sind es ca. 41 Prozent der Plätze, die von mehr als 10 Mannschaften genutzt. Nur 1 Prozent aller Naturrasenplätze wird von mehr als 15 Mannschaften bespielt. Bei Kunstrasenplätzen beträgt der Anteil immerhin noch knapp 18 Prozent. Etwa 6 Prozent werden sogar von über 20 Mannschaften bespielt. Je größer die Vereinsgröße (insbesondere Anzahl der Mannschaften), desto höher ist der Anteil der Vereine, die auch eine Spielstätte vom Typ Kunstrasen haben.  Ein Verbot des Inverkehrbringens von Kunststoffgranulaten als Füllstoff in Kunststoffrasensystemen direkt bei Inkrafttreten der Beschränkung wäre daher unverhältnismäßig. Es würde zu hohen, unerwarteten Umstellungskosten und Mehrkosten für Vereine und Kommunen führen, wodurch dem gemeinwohlorientierten Sport Mittel entzogen würden. Bei fehlender Finanzierbarkeit dieser Mehrkosten ist zudem von einer Schließung vieler Sportplätzen auszugehen, wodurch das Sportangebot in Schulen und Vereinen stark leiden würde. Gerade auf Vereinsebene stellt ein solch außerordentlicher Kosten-punkt ein großes finanzielles Risiko dar, dass das sportliche und gesellschaftliche Gesamtangebot des Vereins gefährden kann. Eine Beschränkung ohne Übergangsfristen, die eine mittelfristige Umstellung und Kostenstreckung erlauben, würde das Breitensportangebot in Deutschland sehr negativ beeinflussen.  Im Hinblick auf den Beschränkungsvorschlag der ECHA gemäß Anhang XV der REACH-Verordnung spricht sich der DFB daher für eine angemessene Übergangsfrist von mindestens sechs Jahren bis zu einem vollständigen Inverkehrbringungsverbot des Kunststoffgranulats zur Verwendung in neuen Kunststoffrasensystemen sowie für die Umstellung bestehender Flächen aus. |
| 15 | l GLOBAL 2000 - Friends of the Earth Austria |  |  | 36 detergent samples were tested in the laboratory of the Federal Environment Agency Austria for possible microplastic compounds (> 50 μm) . In addition the ingredients -according to the ingredients list provided online by the producer - of more than 300 detergents have been compared to the list of 520 polymers published by ECHA early 2019 (Annex XV Restriction Report). In 119 detergents microplastics and/or synthetic Polymers form the 520 Polymer scenario were found. Furthermore detergent producers were contacted and asked if synthetic polymers in their products are water soluble or not. This information is reflected in the report.  The main conclusions are:  1. A comprehensive ban on all non-biodegradable synthetic polymers is urgently needed.  2. With the laboratory test used it was not possible to detect all non water soluble polymers that are in the detergents. Without better and standardized testing methods a restriction will not be feasible.  3. We call for legislation that ensures that in all cases all ingredients are listed on packaging, just like with personal care products. At the moment European legislation permits for cleaning products to reference to a website for a complete list of ingredients by product. |  |  |
| 16 | Landessportbund Rheinland-Pfalz | a.: Nach neueren Erkenntnissen werden zwischen 0,25 t/a und 5 t/a in Deutschland an Befüllungsgranulat pro Kunststoffrasenfläche verwendet (Fraunhofer 2018, S. 11). Das entspricht einer Gesamtmenge von ca. 7.500 bis 9.900 t/a.  b.: Nach dem aktuellen Forschungsstand besteht nach Kenntnis des DFB ein hohes Maß an Unsicherheit darüber, wie und in welchen Mengen das als Mikroplastik definierte Granulat auf Sportplätzen in die Umwelt freigesetzt wird. Nach den uns zur Verfügung stehenden Informationen gibt es große Unterschiede bei der Einschätzung der Menge an Mikroplastiken, die in den einzelnen Mitgliedstaaten oder in der EU/EWR als Füllmaterial für Kunstrasen verwendet wird. Insbesondere Umfang und Methodologie der Forschung in diesem Bereich sind bisher noch wenig standardisiert und nachvollziehbar. Der DFB geht davon aus, dass der Anteil des Eintrags von Mikroplastik über Kunststoffrasenplätze je nach Mitgliedstaat ca. 1 bis 3 Prozent im Verhältnis zum Gesamteintrag beträgt. Demnach ist der Umwelteintrag verglichen mit anderen Hauptquellen relativ gering (Europäische Kommission 2018, ii)). |  | c.: Gezielte Risikomanagementmaßnahmen können die Freisetzung von Füllstoffen in die Umwelt bereits signifikant vermindern. Technische Maßnahmen zur Zurückhaltung eines Materialaustrags vor Ort (z.B. Rinnenfilter mit Sedimentationsstrecken an Abläufen, Schmutzfangmatten, Schuhbürsten am Ausgang) und organisatorische Maßnahmen beim Betrieb der Sportplätze (z.B. regelmäßige Reinigung der Spielfeldränder, Auffangsiebe) können zu einer starken Verringerung des Austrags von Mikroplastik beitragen.  Neben dem häufig genutzten Kunststoffgranulat existieren für Kunststoffrasensysteme alternative Füllstoffe, die in Teilen auch bereits beim Betrieb von Sportanlagen genutzt werden. So werden in Deutschland aktuell Kunststoffrasenplätze teilweise mit Sand und/oder Kork verfüllt. Zudem gibt es auch Kunststoffrasensysteme, die ohne elastischen Füllstoff betrieben werden können.  Es existieren bisher allerdings nur wenige belastbare Studien darüber, wie sich diese Alternativen qualitäts- und kostenmäßig (z.B. hinsichtlich der Bespielbarkeit und Lebensdauer) vergleichen lassen. Zudem müsste untersucht werden, ob und wie sich die Bespielbarkeit oder das Verletzungsrisiko der alternativ befüllten Kunststoffrasenflächen bei den verschiedenen Alternativfüllungen verändert (Plan Miljö Studie 2017). Es bedarf daher dringend weiterer wissenschaftlicher Expertise zur Praxistauglichkeit alternativer organischer Füllstoffe und zur sportartspezifischen Eignung von Kunststoffrasenplätzen, die ohne Füllstoffe auskommen. Sowohl eine wissenschaftliche Folgenabschätzung als auch die dringend erforderliche Entwicklung alternativer Füllstoffe durch die Industrie sind eine zentrale Forderung der von der Thematik betroffenen Sportverbände in Deutschland. Sie vertreten die Meinung, dass die Maßnahmen, die ein Verbot des Kunststoffgranulats verursachen würden, nicht kurzfristig umsetzbar sind und Alternativen nur mittel- bis langfristig erarbeitet und bereitgestellt werden können. |  | d.: In Deutschland gibt es ca. 5.000 für den Fußballspielbetrieb gemeldete Kunststoffrasenplätze (DFBnet), sowie ca. 1.000 DFB-Minispielfelder. Jährlich werden in Deutschland ca. 300 Kunststoffrasenplätze neu gebaut, sowie 150 Kunststoffrasenplätze von Grund auf erneuert. Hinsichtlich der bestehenden Plätze dürfte eine Umstellung auf alternative Füllstoffe notwendig sein. Hierfür halten die Sportanlagenbetreiber (Kommunen oder Vereine) Mittel für Sportstättenbau und -sanierung vor, die bei einem vollständigen Verbot und einer Verwendung alternativer Füllstoffe deutlich höher ausfallen würden. Laut eigener Berechnungen belaufen sich die jährlichen Mehrkosten deutschlandweit auf einen hohen einstelligen Millionenbetrag. Die insgesamt zu erwartenden Kosten eines Verbotes können aufgrund fehlender Kenntnisse über geeignete alternative Füllstoffe (Geeignetheit, Verfügbarkeit) derzeit nicht seriös beziffert werden. Auf Grundlage aktueller Daten zum Bau von Kunststoffrasenplätzen dürfte der Gesamtbetrag für den Austausch des Füllstoffes der Kunststoffrasensysteme im hohen zweistelligen Millionenbereich (bis zu 90 Mio. EUR) liegen, wobei zur Präzisierung dieses Schätzwertes vertiefte Analysen erforderlich sind. Die Kosten für eine Umsetzung gezielter Risikomanagementmaßnahmen zur Zurückhaltung des Materialaustrags dürften nach Schätzungen und je nach Umfang der Maßnahmen pro Kunststoffrasensystem bei 3.000 bis 10.000 EUR liegen.  e.: Der gemeinwohlorientierte Sport ist die größte zivilgesellschaftliche Bewegung in Deutschland und Europa. In Deutschland engagieren sich knapp acht Millionen Bürger freiwillig und ehrenamtlich im Sport. Das entspricht einer jährlichen Wertschöpfung und einem Wohlfahrtsgewinn allein in Deutschland von ca. 6,7 Milliarden Euro. Vergleichbare Zahlen lassen sich auch für die gesamte EU feststellen. In den EU-Mitgliedstaaten engagieren sich im Jahre 2010 zwischen 92 und 94 Millionen Menschen freiwillig für Ziele des Gemeinwohls, davon die meisten im Sport (ca. 35 bis 40 Prozent aller freiwillig Tätigen in der EU) (Europäische Kommission 2010).  Der Sport schafft ein strukturiertes, an die gesamte Bevölkerung gerichtetes und für alle offenes Bewegungs- und Sportangebot, durch das wichtige soziale und gesundheitsfördernde Funktionen in der Gesellschaft erfüllt werden. Sportvereine in Deutschland zählen zehn Millionen Mitgliedschaften im Kinder- und Jugendalter (DOSB-Bestandserhebung 2018), allein im DFB liegt diese Zahl bei 2,1 Millionen (DFB-Mitgliederstatistik 2018). Damit sind Sportvereine die wichtigste Anlaufstelle für Kinder und Jugendliche außerhalb der Schule und übernehmen unverzichtbare Aufgaben für die ganzheitliche Persönlichkeitsbildung junger Menschen. Dem Sport kommt eine wichtige Vorbild- und Lehrfunktion im Bereich der Integration und demokratischen Grundbildung zu. Für das herausragende gesellschaftliche Engagement des Sports spricht nicht zuletzt, dass die Sportvereine eng mit Schulen, Kindergärten, Unternehmen, Krankenkassen oder anderen öffentlichen Institutionen zusammenarbeiten. Um allen Bürgern den Zugang zum Sport zu ermöglichen, sind adäquate Sportstätten in ausreichender Anzahl Grundvoraussetzung. Ein für alle zugängliches und umfangreiches Sportangebot ist – vor allen Dingen in Großstädten und Ballungsgebieten – nur durch die Verfügbarkeit von ganzjährig nutzbaren Sportanlagen zu gewährleisten. Kunststoffrasenplätze spielen hierbei, insbesondere für den Fußball, eine wichtige Rolle, da sie eine intensivere Nutzung als Naturrasen- oder Tennenplätze erlauben. Allein mit Naturrasen- und Tennenplätzen lässt sich der derzeitige Trainings- und Spielbetrieb, insbesondere bei den Kinder- und Jugendmannschaften, nicht aufrechterhalten. Ein Kunststoffrasenplatz ersetzt etwa 2,5 Naturrasenplätze (DFBnet). Auf weniger als 10 Prozent der Naturrasenplätze finden an Wochenenden mehr als 2 Spiele statt. Bei Kunststoffrasenplätzen finden hingegen bei über 40 Prozent der Plätze mehr als 2 Spiele statt. Weniger als 10 Prozent der Naturrasenplätze wird an einem Wochenende mehr als 150 Minuten genutzt. Bei Kunstrasenplätzen werden hingegen etwa 35 Prozent an einem Wochenende mehr als 150 Minuten genutzt. 27.773 Spielstätten in Deutschland (ca. 70 Prozent) werden von Sportvereinen genutzt. Ein Drittel der Kunstrasenplätze werden von 2 oder mehr Vereinen mit alle ihren Jugend- und Seniorenmannschaften benutzt. Etwas über ein Drittel aller Naturrasenplätze wird von mehr als 5 Mannschaften bespielt. Bei Kunstrasenplätzen werden fast drei Viertel (72 Prozent) von mehr als 5 Mannschaften genutzt. Etwa 10 Prozent aller Naturrasenplätze wird von mehr als 10 Mannschaften bespielt. Bei Kunststoffrasenplätzen sind es ca. 41 Prozent der Plätze, die von mehr als 10 Mannschaften genutzt. Nur 1 Prozent aller Naturrasenplätze wird von mehr als 15 Mannschaften bespielt. Bei Kunstrasenplätzen beträgt der Anteil immerhin noch knapp 18 Prozent. Etwa 6 Prozent werden sogar von über 20 Mannschaften bespielt. Je größer die Vereinsgröße (insbesondere Anzahl der Mannschaften), desto höher ist der Anteil der Vereine, die auch eine Spielstätte vom Typ Kunstrasen haben.  Ein Verbot des Inverkehrbringens von Kunststoffgranulaten als Füllstoff in Kunststoffrasensystemen direkt bei Inkrafttreten der Beschränkung wäre daher unverhältnismäßig. Es würde zu hohen, unerwarteten Umstellungskosten und Mehrkosten für Vereine und Kommunen führen, wodurch dem gemeinwohlorientierten Sport Mittel entzogen würden. Bei fehlender Finanzierbarkeit dieser Mehrkosten ist zudem von einer Schließung vieler Sportplätzen auszugehen, wodurch das Sportangebot in Schulen und Vereinen stark leiden würde. Gerade auf Vereinsebene stellt ein solch außerordentlicher Kosten-punkt ein großes finanzielles Risiko dar, dass das sportliche und gesellschaftliche Gesamtangebot des Vereins gefährden kann. Eine Beschränkung ohne Übergangsfristen, die eine mittelfristige Umstellung und Kostenstreckung erlauben, würde das Breitensportangebot in Deutschland sehr negativ beeinflussen.  Im Hinblick auf den Beschränkungsvorschlag der ECHA gemäß Anhang XV der REACH-Verordnung spricht sich der DFB daher für eine angemessene Übergangsfrist von mindestens sechs Jahren bis zu einem vollständigen Inverkehrbringungsverbot des Kunststoffgranulats zur Verwendung in neuen Kunststoffrasensystemen sowie für die Umstellung bestehender Flächen aus. |
| 17 | LandesSportBund Sachsen-Anhalt e.V. | a.: Nach neueren Erkenntnissen werden zwischen 0,25 t/a und 5 t/a in Deutschland an Befüllungsgranulat pro Kunststoffrasenfläche verwendet (Fraunhofer 2018, S. 11). Das entspricht einer Gesamtmenge von ca. 7.500 bis 9.900 t/a.  b.: Nach dem aktuellen Forschungsstand besteht nach Kenntnis des DFB ein hohes Maß an Unsicherheit darüber, wie und in welchen Mengen das als Mikroplastik definierte Granulat auf Sportplätzen in die Umwelt freigesetzt wird. Nach den uns zur Verfügung stehen-den Informationen gibt es große Unterschiede bei der Einschätzung der Menge an Mikroplastiken, die in den einzelnen Mitgliedstaaten oder in der EU/EWR als Füllmaterial für Kunstrasen verwendet wird. Insbesondere Umfang und Methodologie der Forschung in diesem Bereich sind bisher noch wenig standardisiert und nachvollziehbar. Der DFB geht davon aus, dass der Anteil des Eintrags von Mikroplastik über Kunststoffrasenplätze je nach Mitgliedstaat ca. 1 bis 3 Prozent im Verhältnis zum Gesamteintrag beträgt. Demnach ist der Umwelteintrag verglichen mit anderen Hauptquellen relativ gering (Europäische Kommission 2018, ii)). |  | c.: Gezielte Risikomanagementmaßnahmen können die Freisetzung von Füllstoffen in die Umwelt bereits signifikant vermindern. Technische Maßnahmen zur Zurückhaltung eines Materialaustrags vor Ort (z.B. Rinnenfilter mit Sedimentationsstrecken an Abläufen, Schmutzfangmatten, Schuhbürsten am Ausgang) und organisatorische Maßnahmen beim Betrieb der Sportplätze (z.B. regelmäßige Reinigung der Spielfeldränder, Auffangsiebe) können zu einer starken Verringerung des Austrags von Mikroplastik beitragen.  Neben dem häufig genutzten Kunststoffgranulat existieren für Kunststoffrasensysteme alternative Füllstoffe, die in Teilen auch bereits beim Betrieb von Sportanlagen genutzt werden. So werden in Deutschland aktuell Kunststoffrasenplätze teilweise mit Sand und/oder Kork verfüllt. Zudem gibt es auch Kunststoffrasensysteme, die ohne elastischen Füllstoff betrieben werden können.  Es existieren bisher allerdings nur wenige belastbare Studien darüber, wie sich diese Alternativen qualitäts- und kostenmäßig (z.B. hinsichtlich der Bespielbarkeit und Lebensdauer) vergleichen lassen. Zudem müsste untersucht werden, ob und wie sich die Bespielbar-keit oder das Verletzungsrisiko der alternativ befüllten Kunststoffrasenflächen bei den verschiedenen Alternativfüllungen verändert (Plan Miljø Studie 2017). Es bedarf daher dringend weiterer wissenschaftlicher Expertise zur Praxistauglichkeit alternativer organischer Füllstoffe und zur sportartspezifischen Eignung von Kunststoffrasenplätzen, die ohne Füllstoffe auskommen. Sowohl eine wissenschaftliche Folgenabschätzung als auch die dringend erforderliche Entwicklung alternativer Füllstoffe durch die Industrie sind eine zentrale Forderung der von der Thematik betroffenen Sportverbände in Deutschland. Sie vertreten die Meinung, dass die Maßnahmen, die ein Verbot des Kunststoffgranulats verursachen würden, nicht kurzfristig umsetzbar sind und Alternativen nur mittel- bis langfristig erarbeitet und bereitgestellt werden können. |  | d.: In Deutschland gibt es ca. 5.000 für den Fußballspielbetrieb gemeldete Kunststoffrasenplätze (DFBnet), sowie ca. 1.000 DFB-Minispielfelder. Jährlich werden in Deutschland ca. 300 Kunststoffrasenplätze neu gebaut, sowie 150 Kunststoffrasenplätze von Grund auf erneuert. Hinsichtlich der bestehenden Plätze dürfte eine Umstellung auf alternative Füllstoffe notwendig sein. Hierfür halten die Sportanlagenbetreiber (Kommunen oder Vereine) Mittel für Sportstättenbau und -sanierung vor, die bei einem vollständigen Ver-bot und einer Verwendung alternativer Füllstoffe deutlich höher ausfallen würden. Laut eigener Berechnungen belaufen sich die jährlichen Mehrkosten deutschlandweit auf einen hohen einstelligen Millionenbetrag. Die insgesamt zu erwartenden Kosten eines Verbotes können aufgrund fehlender Kenntnisse über geeignete alternative Füllstoffe (Geeignetheit, Verfügbarkeit) derzeit nicht seriös beziffert werden. Auf Grundlage aktueller Daten zum Bau von Kunststoffrasenplätzen dürfte der Gesamtbetrag für den Austausch des Füllstoffes der Kunststoffrasensysteme im hohen zweistelligen Millionenbereich (bis zu 90 Mio. EUR) liegen, wobei zur Präzisierung dieses Schätzwertes vertiefte Analysen erforderlich sind. Die Kosten für eine Umsetzung gezielter Risikomanagementmaßnahmen zur Zurückhaltung des Materialaustrags dürften nach Schätzungen und je nach Umfang der Maßnahmen pro Kunststoffrasensystem bei 3.000 bis 10.000 EUR liegen.  e.: Der gemeinwohlorientierte Sport ist die größte zivilgesellschaftliche Bewegung in Deutschland und Europa. Im Fußballverband Sachsen-Anhalt engagieren sich knapp 19.000 Bürger freiwillig und ehrenamtlich im organisierten Fußball für rund 91.000 Mitglieder in über 790 Sportvereinen.  Der Sport schafft ein strukturiertes, an die gesamte Bevölkerung gerichtetes und für alle offenes Bewegungs- und Sportangebot, durch das wichtige soziale und gesundheitsfördernde Funktionen in der Gesellschaft erfüllt werden. Fußballvereine in Sachsen-Anhalt zählen rund 41.000 Mitgliedschaften im Kinder- und Jugendalter. Damit sind die Fußballvereine die wichtigste Anlaufstelle für Kinder und Jugendliche außerhalb der Schule und übernehmen unverzichtbare Aufgaben für die ganzheitliche Persönlichkeitsbildung junger Menschen. Dem Sport kommt eine wichtige Vorbild- und Lehrfunktion im Bereich der Integration und demokratischen Grundbildung zu. Für das herausragende gesellschaftliche Engagement des Sports spricht nicht zuletzt, dass die Sportvereine eng mit Schulen, Kindergärten, Unternehmen, Krankenkassen oder anderen öffentlichen Institutionen zusammenarbeiten. Um allen Bürgern den Zugang zum Sport zu ermöglichen, sind adäquate Sportstätten in ausreichender Anzahl Grundvoraussetzung. Ein für alle zugängliches und umfangreiches Sportangebot ist nur durch die Verfügbarkeit von ganzjährig nutzbaren Sportanlagen zu gewährleisten. Kunststoffrasenplätze spielen hierbei, insbesondere für den Fußball, eine wichtige Rolle, da sie eine intensivere Nutzung als Naturrasen- oder Tennenplätze erlauben. Allein mit Naturrasen- und Tennenplätzen lässt sich der derzeitige Trainings- und Spielbetrieb, insbesondere bei den Kinder- und Jugendmannschaften, nicht aufrechterhalten.  Im Fußball in Sachsen-Anhalt gibt es rund 81 Sportanlagen (zum Teil mit mehreren Spielfeldern) an denen für den Fußball und (meist überlappend) Hockey Kunststoffrasenplätze vorhanden sind. Insgesamt nutzen Fußball- und die 5 Hockeyvereine insgesamt knapp 650 Sportplätze. Die circa 13 % Kunststoffrasenplätze befinden sich schwerpunktmäßig in den beiden Großstädten Magdeburg und Halle (Saale). Hier wäre ein Trainings- und Wettkampfbetrieb etlicher Vereine ohne diese Anlagen undenkbar. An diesen Standorten nutzen die Anlagen nicht nur mehrere Fußballvereine gleichzeitig sondern teilen sich die Sportarten Fußball und Hockey auch den Sportplatz.  Ungeachtet der Tatsache, dass ohne diese Plätze der Trainings- und Wettkampfbedarf nicht auch nur annährend bedient werden könnte, wäre es ebenso unvorstellbar diese Stunden auf neu zu errichtende Naturrasenplätze umzulagern, da die hohe Stundenzahl der Nutzung auf dem Naturbelag nicht möglich ist.  Ein Verbot des Inverkehrbringens von Kunststoffgranulaten als Füllstoff in Kunststoffrasensystemen direkt bei Inkrafttreten der Beschränkung wäre daher unverhältnismäßig. Es würde zu hohen, unerwarteten Umstellungskosten und Mehrkosten für Vereine und Kommunen führen, wodurch dem gemeinwohlorientierten Sport Mittel entzogen würden. Bei fehlender Finanzierbarkeit dieser Mehrkosten ist zudem von einer Schließung vieler Sportplätzen auszugehen, wodurch das Sportangebot in Schulen und Vereinen stark leiden würde. Gerade auf Vereinsebene stellt ein solch außerordentlicher Kostenpunkt ein großes finanzielles Risiko dar, dass das sportliche und gesellschaftliche Gesamtangebot des Vereins gefährden würde. Eine Beschränkung ohne Übergangsfristen, die eine mittelfristige Umstellung und Kostenstreckung erlauben, würde das Sportangebot in Sachsen-Anhalt sehr negativ beeinflussen.  Im Hinblick auf den Beschränkungsvorschlag der ECHA gemäß Anhang XV der REACH-Verordnung spricht sich der LandesSportBund Sachsen-Anhalt daher für eine angemessene Übergangsfrist von mindestens sechs Jahren bis zu einem vollständigen Inverkehrbringungsverbot des Kunststoffgranulats zur Verwendung in neuen Kunststoffrasensystemen sowie für die Umstellung bestehender Flächen aus. |
| 18 | Legambiente | We understand that matting/opacizing microplastics and thickening microplastics are excluded from the scope of this proposal. These are in large part acrylic/metacrylic/styrenic co-polymers.  On top of this we must consider that these figures only refer to a relevant amount of home detergents, but do not cover them all: we should add to the figures other detergents that also contain or may contain the same matting/opacizing microplastics, such as furniture detergents. Besides, syntetic thickeners and matting/opacizing microplastics are present in personal care products too. |  | According to a study from AISE, liquid laudry detergents in Europe in 2017 had a market of 1.600.000 ton this report, pg. 22)  <https://www.aise.eu/documents/document/20160229142408-16-02-01_prep-l2_project_description_final_(3).pdf>  We consider that 0.4% can be a realistic gross estimation of the average presence of matting/opacizing materials in liquid soap;  We also estimate that of that share, on average 40% is composed of microplastics.  Given these figures the total amount of matting/opacizing microplastics going down the pipe every year in Europe can sum up to 2560 Ton. (=1.600.000 * 0.4% * 40%). As a benchmark, we mention here that UNEP in 2017 has estimated that the total amount of MP released into the environment from cosmetics is 42.000T/y worldwide.  We would like to stress here that canadian regulation on microplastics clearly defines matting/opacizing microplastics as such  ("Microbeads - A Science summary" July 2015) <http://www.ec.gc.ca/ese-ees/adda4c5f-f397-48d5-ad17-63f989ebd0e5/microbeads_science%20summary_en.pdf>  on the base of a Dutch study ("Review of microplastics in cosmetics", H.A. Leslie, July 2014)  <https://science.vu.nl/en/Images/Plastic_ingredients_in_Cosmetics_07-2014_FINAL_tcm296-409859.pdf>  and bans them (Ban on the manufacturing, import, and placing on the market of any toiletries for cleansing or hygiene that contain microbeads. Entry into Force: 1 July 2018).  There are, on the other hand, several existing alternatives, already widely available and used, to thickeners and matting/opacizing plastics.  For thickeners:  Common salt is used to increase viscosity in formulas containing surfactants such as SLES.  It is worth mentioning here that other natural thickeners used for such purpose are  • clays such as bentonite  • cullulose and modified cellulose (CMC, HMC, HPMC)  • Gums such as Xantham Gum, guar, locust bean)  • Carbohydrates (carragenine, pectine, alginic acid)  • proteines (caseine)  •  Several studies are available on natural thickeners, such as:  <https://www.researchgate.net/publication/292447286_Thickener_choice_-_A_way_to_improve_cosmetics_sensory_properties>  <https://patents.google.com/patent/US20030158324> (paragraph 0018)  For Matting/opacizing microplastics:  Natural alternatives to matting/opacizing microplastics are also present in the market and produced and used by important international companies such as BASF (euperlan)  Other products that are used for this purpose are non-organic pigments such as TiO2, waxes (including emusions of natural bee wax), kaolinite, talcs, fatty alchools and esthers and stearates of fatty alchools (<https://www.berg-schmidt.de/en/Cosmetic/c25_opacifier.php>).  Other patents and pubblications on this topic can be found here:  <https://patents.google.com/patent/US7176171B2/en>  <https://patents.google.com/patent/US5851541A/en>  <https://pubs.acs.org/doi/full/10.1021/bk-2013-1148.ch001?src=recsys> |  |  |
| 19 | NA 005-01-22 AA DIN-NABau (Germany) | Information on alternatives;  Information on costs |  |  |  |  |
| 20 | NABU |  |  | Microplastics and poorly biodegradable polymer compounds are currently under-regulated because of gaps in the EU’s chemical and product regulations, according to a new study commissioned by German environmental Group NABU. NABU’s report calls on the European Commission to regulate and restrict through REACH polymer compounds that are slow to biodegrade. It adds that all microplastics, not just microbeads, should be banned from cosmetics, washing and cleaning products, since individual national bans cannot solve the problem alone. “I am worried that the European Commission or Parliament will not define exactly what they mean by microplastics,” she said, particularly given the relatively strong influence of the industry lobby in Brussels. (Ends 2018c) |  |  |
| 21 | Niedersächsischer Fußballverband e. V. | a.: Nach neueren Erkenntnissen werden zwischen 0,25 t/a und 5 t/a in Deutschland an Befüllungsgranulat pro Kunststoffrasenfläche verwendet (Fraunhofer 2018, S. 11). Das entspricht einer Gesamtmenge von ca. 7.500 bis 9.900 t/a.  b.: Nach dem aktuellen Forschungsstand besteht nach Kenntnis des DFB ein hohes Maß an Unsicherheit darüber, wie und in welchen Mengen das als Mikroplastik definierte Granulat auf Sportplätzen in die Umwelt freigesetzt wird. Nach den uns zur Verfügung stehen-den Informationen gibt es große Unterschiede bei der Einschätzung der Menge an Mikroplastiken, die in den einzelnen Mitgliedstaaten oder in der EU/EWR als Füllmaterial für Kunstrasen verwendet wird. Insbesondere Umfang und Methodologie der Forschung in diesem Bereich sind bisher noch wenig standardisiert und nachvollziehbar. Der DFB geht davon aus, dass der Anteil des Eintrags von Mikroplastik über Kunststoffrasenplätze je nach Mitgliedstaat ca. 1 bis 3 Prozent im Verhältnis zum Gesamteintrag beträgt. Demnach ist der Umwelteintrag verglichen mit anderen Hauptquellen relativ gering (Europäische Kommission 2018, ii)). |  | c.: Gezielte Risikomanagementmaßnahmen können die Freisetzung von Füllstoffen in die Umwelt bereits signifikant vermindern. Technische Maßnahmen zur Zurückhaltung eines Materialaustrags vor Ort (z.B. Rinnenfilter mit Sedimentationsstrecken an Abläufen, Schmutzfangmatten, Schuhbürsten am Ausgang) und organisatorische Maßnahmen beim Betrieb der Sportplätze (z.B. regelmäßige Reinigung der Spielfeldränder, Auffangsiebe) können zu einer starken Verringerung des Austrags von Mikroplastik beitragen.  Neben dem häufig genutzten Kunststoffgranulat existieren für Kunststoffrasensysteme alternative Füllstoffe, die in Teilen auch bereits beim Betrieb von Sportanlagen genutzt werden. So werden in Deutschland aktuell Kunststoffrasenplätze teilweise mit Sand und/oder Kork verfüllt. Zudem gibt es auch Kunststoffrasensysteme, die ohne elastischen Füllstoff betrieben werden können.  Es existieren bisher allerdings nur wenige belastbare Studien darüber, wie sich diese Alternativen qualitäts- und kostenmäßig (z.B. hinsichtlich der Bespielbarkeit und Lebensdauer) vergleichen lassen. Zudem müsste untersucht werden, ob und wie sich die Bespielbar-keit oder das Verletzungsrisiko der alternativ befüllten Kunststoffrasenflächen bei den verschiedenen Alternativfüllungen verändert (Plan Miljø Studie 2017). Es bedarf daher dringend weiterer wissenschaftlicher Expertise zur Praxistauglichkeit alternativer organischer Füllstoffe und zur sportartspezifischen Eignung von Kunststoffrasenplätzen, die ohne Füllstoffe auskommen. Sowohl eine wissenschaftliche Folgenabschätzung als auch die dringend erforderliche Entwicklung alternativer Füllstoffe durch die Industrie sind eine zentrale Forderung der von der Thematik betroffenen Sportverbände in Deutschland. Sie vertreten die Meinung, dass die Maßnahmen, die ein Verbot des Kunststoffgranulats verursachen würden, nicht kurzfristig umsetzbar sind und Alternativen nur mittel- bis langfristig erarbeitet und bereitgestellt werden können. |  | d.: In Deutschland gibt es ca. 5.000 für den Fußballspielbetrieb gemeldete Kunststoffrasenplätze (DFBnet), sowie ca. 1.000 DFB-Minispielfelder. Jährlich werden in Deutschland ca. 300 Kunststoffrasenplätze neu gebaut, sowie 150 Kunststoffrasenplätze von Grund auf erneuert. Hinsichtlich der bestehenden Plätze dürfte eine Umstellung auf alternative Füllstoffe notwendig sein. Hierfür halten die Sportanlagenbetreiber (Kommunen oder Vereine) Mittel für Sportstättenbau und -sanierung vor, die bei einem vollständigen Ver-bot und einer Verwendung alternativer Füllstoffe deutlich höher ausfallen würden. Laut eigener Berechnungen belaufen sich die jährlichen Mehrkosten deutschlandweit auf einen hohen einstelligen Millionenbetrag. Die insgesamt zu erwartenden Kosten eines Verbotes können aufgrund fehlender Kenntnisse über geeignete alternative Füllstoffe (Geeignetheit, Verfügbarkeit) derzeit nicht seriös beziffert werden. Auf Grundlage aktueller Daten zum Bau von Kunststoffrasenplätzen dürfte der Gesamtbetrag für den Austausch des Füllstoffes der Kunststoffrasensysteme im hohen zweistelligen Millionenbereich (bis zu 90 Mio. EUR) liegen, wobei zur Präzisierung dieses Schätzwertes vertiefte Analysen erforderlich sind. Die Kosten für eine Umsetzung gezielter Risikomanagementmaßnahmen zur Zurückhaltung des Materialaustrags dürften nach Schätzungen und je nach Umfang der Maßnahmen pro Kunststoffrasensystem bei 3.000 bis 10.000 EUR liegen.  e.: Der gemeinwohlorientierte Sport ist die größte zivilgesellschaftliche Bewegung in Deutschland und Europa. In Deutschland engagieren sich knapp acht Millionen Bürger freiwillig und ehrenamtlich im Sport. Das entspricht einer jährlichen Wertschöpfung und einem Wohlfahrtsgewinn allein in Deutschland von ca. 6,7 Milliarden Euro. Vergleichbare Zahlen lassen sich auch für die gesamte EU feststellen. In den EU-Mitgliedstaaten engagieren sich im Jahre 2010 zwischen 92 und 94 Millionen Menschen freiwillig für Ziele des Gemeinwohls, davon die meisten im Sport (ca. 35 bis 40 Prozent aller freiwillig Tätigen in der EU) (Europäische Kommission 2010).  Der Sport schafft ein strukturiertes, an die gesamte Bevölkerung gerichtetes und für alle offenes Bewegungs- und Sportangebot, durch das wichtige soziale und gesundheitsfördernde Funktionen in der Gesellschaft erfüllt werden. Sportvereine in Deutschland zählen zehn Millionen Mitgliedschaften im Kinder- und Jugendalter (DOSB-Bestandserhebung 2018), allein im DFB liegt diese Zahl bei 2,1 Millionen (DFB-Mitgliederstatistik 2018). Damit sind Sportvereine die wichtigste Anlaufstelle für Kinder und Jugendliche außerhalb der Schule und übernehmen unverzichtbare Aufgaben für die ganzheitliche Persönlichkeitsbildung junger Menschen. Dem Sport kommt eine wichtige Vorbild- und Lehrfunktion im Bereich der Integration und demokratischen Grundbildung zu. Für das herausragende gesellschaftliche Engagement des Sports spricht nicht zuletzt, dass die Sportvereine eng mit Schulen, Kindergärten, Unternehmen, Krankenkassen oder anderen öffentlichen Institutionen zusammenarbeiten. Um allen Bürgern den Zugang zum Sport zu ermöglichen, sind adäquate Sportstätten in ausreichender Anzahl Grundvoraussetzung. Ein für alle zugängliches und umfangreiches Sportangebot ist – vor allen Dingen in Großstädten und Ballungsgebieten – nur durch die Verfügbarkeit von ganzjährig nutzbaren Sportanlagen zu gewährleisten. Kunststoffrasenplätze spielen hierbei, insbesondere für den Fußball, eine wichtige Rolle, da sie eine intensivere Nutzung als Naturrasen- oder Tennenplätze erlauben. Allein mit Naturrasen- und Tennenplätzen lässt sich der derzeitige Trainings- und Spielbetrieb, insbesondere bei den Kinder- und Jugendmannschaften, nicht aufrechterhalten. Ein Kunststoffrasenplatz ersetzt etwa 2,5 Naturrasenplätze (DFBnet). Auf weniger als 10 Prozent der Naturrasenplätze finden an Wochenenden mehr als 2 Spiele statt. Bei Kunststoffrasenplätzen finden hingegen bei über 40 Prozent der Plätze mehr als 2 Spiele statt. Weniger als 10 Prozent der Naturrasenplätze wird an einem Wochenende mehr als 150 Minuten genutzt. Bei Kunstrasenplätzen werden hingegen etwa 35 Prozent an einem Wochenende mehr als 150 Minuten genutzt. 27.773 Spielstätten in Deutschland (ca. 70 Prozent) werden von Sportvereinen genutzt. Ein Drittel der Kunstrasenplätze werden von 2 oder mehr Vereinen mit alle ihren Jugend- und Seniorenmannschaften benutzt. Etwas über ein Drittel aller Naturrasenplätze wird von mehr als 5 Mannschaften bespielt. Bei Kunstrasenplätzen werden fast drei Viertel (72 Prozent) von mehr als 5 Mannschaften genutzt. Etwa 10 Prozent aller Naturrasenplätze wird von mehr als 10 Mannschaften bespielt. Bei Kunststoffrasenplätzen sind es ca. 41 Prozent der Plätze, die von mehr als 10 Mannschaften genutzt. Nur 1 Prozent aller Naturrasenplätze wird von mehr als 15 Mannschaften bespielt. Bei Kunstrasenplätzen beträgt der Anteil immerhin noch knapp 18 Prozent. Etwa 6 Prozent werden sogar von über 20 Mannschaften bespielt. Je größer die Vereinsgröße (insbesondere Anzahl der Mannschaften), desto höher ist der Anteil der Vereine, die auch eine Spielstätte vom Typ Kunstrasen haben.  Ein Verbot des Inverkehrbringens von Kunststoffgranulaten als Füllstoff in Kunststoffrasensystemen direkt bei Inkrafttreten der Beschränkung wäre daher unverhältnismäßig. Es würde zu hohen, unerwarteten Umstellungskosten und Mehrkosten für Vereine und Kommunen führen, wodurch dem gemeinwohlorientierten Sport Mittel entzogen würden. Bei fehlender Finanzierbarkeit dieser Mehrkosten ist zudem von einer Schließung vieler Sportplätzen auszugehen, wodurch das Sportangebot in Schulen und Vereinen stark leiden würde. Gerade auf Vereinsebene stellt ein solch außerordentlicher Kosten-punkt ein großes finanzielles Risiko dar, dass das sportliche und gesellschaftliche Gesamtangebot des Vereins gefährden kann. Eine Beschränkung ohne Übergangsfristen, die eine mittelfristige Umstellung und Kostenstreckung erlauben, würde das Breitensportangebot in Deutschland sehr negativ beeinflussen.  Im Verbandsgebiet des Niedersächsischen Fußballverbandes e.V. (NFV) mit mehr als 2.600 Mitgliedsvereinen befinden sich 193 Kunstrasenplätze im Spiel- und Trainingsbetrieb. Daneben nutzten unsere Mitgliedsvereine für den ergänzenden Trainings- und Freizeitspielbetrieb mehr als 100 Kunstrasen-Minispielfelder. Sofern diese Spiel- Trainings- und Freizeitsportflächen derart kurzfristig nicht weiter zur Verfügung stehen sollten, stellt dies für das gesamte Sportangebot unserer Mitgliedsvereine eine unverhältnismäßige Einschränkung dar, die in der angedachten Kürze auch nicht zu kompensieren ist.  Im Hinblick auf den Beschränkungsvorschlag der ECHA gemäß Anhang XV der REACH-Verordnung spricht sich der DFB und der NFV daher für eine angemessene Übergangsfrist von mindestens sechs Jahren bis zu einem vollständigen Inverkehrbringungsverbot des Kunststoffgranulats zur Verwendung in neuen Kunststoffrasensystemen sowie für die Umstellung bestehender Flächen aus. |
| 22 | Saarländischer Fußballverband e.V. | a.: Nach neueren Erkenntnissen werden zwischen 0,25 t/a und 5 t/a in Deutschland an Befüllungsgranulat pro Kunststoffrasenfläche verwendet (Fraunhofer 2018, S. 11). Das entspricht einer Gesamtmenge von ca. 7.500 bis 9.900 t/a.  b.: Nach dem aktuellen Forschungsstand besteht nach Kenntnis des DFB ein hohes Maß an Unsicherheit darüber, wie und in welchen Mengen das als Mikroplastik definierte Granulat auf Sportplätzen in die Umwelt freigesetzt wird. Nach den uns zur Verfügung stehen-den Informationen gibt es große Unterschiede bei der Einschätzung der Menge an Mikroplastiken, die in den einzelnen Mitgliedstaaten oder in der EU/EWR als Füllmaterial für Kunstrasen verwendet wird. Insbesondere Umfang und Methodologie der Forschung in diesem Bereich sind bisher noch wenig standardisiert und nachvollziehbar. Der DFB geht davon aus, dass der Anteil des Eintrags von Mikroplastik über Kunststoffrasenplätze je nach Mitgliedstaat ca. 1 bis 3 Prozent im Verhältnis zum Gesamteintrag beträgt. Demnach ist der Umwelteintrag verglichen mit anderen Hauptquellen relativ gering (Europäische Kommission 2018, ii)). |  | c.: Gezielte Risikomanagementmaßnahmen können die Freisetzung von Füllstoffen in die Umwelt bereits signifikant vermindern. Technische Maßnahmen zur Zurückhaltung eines Materialaustrags vor Ort (z.B. Rinnenfilter mit Sedimentationsstrecken an Abläufen, Schmutzfangmatten, Schuhbürsten am Ausgang) und organisatorische Maßnahmen beim Betrieb der Sportplätze (z.B. regelmäßige Reinigung der Spielfeldränder, Auffangsiebe) können zu einer starken Verringerung des Austrags von Mikroplastik beitragen.  Neben dem häufig genutzten Kunststoffgranulat existieren für Kunststoffrasensysteme alternative Füllstoffe, die in Teilen auch bereits beim Betrieb von Sportanlagen genutzt werden. So werden in Deutschland aktuell Kunststoffrasenplätze teilweise mit Sand und/oder Kork verfüllt. Zudem gibt es auch Kunststoffrasensysteme, die ohne elastischen Füllstoff betrieben werden können.  Es existieren bisher allerdings nur wenige belastbare Studien darüber, wie sich diese Alternativen qualitäts- und kostenmäßig (z.B. hinsichtlich der Bespielbarkeit und Lebensdauer) vergleichen lassen. Zudem müsste untersucht werden, ob und wie sich die Bespielbar-keit oder das Verletzungsrisiko der alternativ befüllten Kunststoffrasenflächen bei den verschiedenen Alternativfüllungen verändert (Plan Miljø Studie 2017). Es bedarf daher dringend weiterer wissenschaftlicher Expertise zur Praxistauglichkeit alternativer organischer Füllstoffe und zur sportartspezifischen Eignung von Kunststoffrasenplätzen, die ohne Füllstoffe auskommen. Sowohl eine wissenschaftliche Folgenabschätzung als auch die dringend erforderliche Entwicklung alternativer Füllstoffe durch die Industrie sind eine zentrale Forderung der von der Thematik betroffenen Sportverbände in Deutschland. Sie vertreten die Meinung, dass die Maßnahmen, die ein Verbot des Kunststoffgranulats verursachen würden, nicht kurzfristig umsetzbar sind und Alternativen nur mittel- bis langfristig erarbeitet und bereitgestellt werden können. |  | d.: In Deutschland gibt es ca. 5.000 für den Fußballspielbetrieb gemeldete Kunststoffrasenplätze (DFBnet), sowie ca. 1.000 DFB-Minispielfelder. Jährlich werden in Deutschland ca. 300 Kunststoffrasenplätze neu gebaut, sowie 150 Kunststoffrasenplätze von Grund auf erneuert. Hinsichtlich der bestehenden Plätze dürfte eine Umstellung auf alternative Füllstoffe notwendig sein. Hierfür halten die Sportanlagenbetreiber (Kommunen oder Vereine) Mittel für Sportstättenbau und -sanierung vor, die bei einem vollständigen Ver-bot und einer Verwendung alternativer Füllstoffe deutlich höher ausfallen würden. Laut eigener Berechnungen belaufen sich die jährlichen Mehrkosten deutschlandweit auf einen hohen einstelligen Millionenbetrag. Die insgesamt zu erwartenden Kosten eines Verbotes können aufgrund fehlender Kenntnisse über geeignete alternative Füllstoffe (Geeignetheit, Verfügbarkeit) derzeit nicht seriös beziffert werden. Auf Grundlage aktueller Daten zum Bau von Kunststoffrasenplätzen dürfte der Gesamtbetrag für den Austausch des Füllstoffes der Kunststoffrasensysteme im hohen zweistelligen Millionenbereich (bis zu 90 Mio. EUR) liegen, wobei zur Präzisierung dieses Schätzwertes vertiefte Analysen erforderlich sind. Die Kosten für eine Umsetzung gezielter Risikomanagementmaßnahmen zur Zurückhaltung des Materialaustrags dürften nach Schätzungen und je nach Umfang der Maßnahmen pro Kunststoffrasensystem bei 3.000 bis 10.000 EUR liegen.  e.: Der gemeinwohlorientierte Sport ist die größte zivilgesellschaftliche Bewegung in Deutschland und Europa. In Deutschland engagieren sich knapp acht Millionen Bürger freiwillig und ehrenamtlich im Sport. Das entspricht einer jährlichen Wertschöpfung und einem Wohlfahrtsgewinn allein in Deutschland von ca. 6,7 Milliarden Euro. Vergleichbare Zahlen lassen sich auch für die gesamte EU feststellen. In den EU-Mitgliedstaaten engagieren sich im Jahre 2010 zwischen 92 und 94 Millionen Menschen freiwillig für Ziele des Gemeinwohls, davon die meisten im Sport (ca. 35 bis 40 Prozent aller freiwillig Tätigen in der EU) (Europäische Kommission 2010).  Der Sport schafft ein strukturiertes, an die gesamte Bevölkerung gerichtetes und für alle offenes Bewegungs- und Sportangebot, durch das wichtige soziale und gesundheitsfördernde Funktionen in der Gesellschaft erfüllt werden. Sportvereine in Deutschland zählen zehn Millionen Mitgliedschaften im Kinder- und Jugendalter (DOSB-Bestandserhebung 2018), allein im DFB liegt diese Zahl bei 2,1 Millionen (DFB-Mitgliederstatistik 2018). Damit sind Sportvereine die wichtigste Anlaufstelle für Kinder und Jugendliche außerhalb der Schule und übernehmen unverzichtbare Aufgaben für die ganzheitliche Persönlichkeitsbildung junger Menschen. Dem Sport kommt eine wichtige Vorbild- und Lehrfunktion im Bereich der Integration und demokratischen Grundbildung zu. Für das herausragende gesellschaftliche Engagement des Sports spricht nicht zuletzt, dass die Sportvereine eng mit Schulen, Kindergärten, Unternehmen, Krankenkassen oder anderen öffentlichen Institutionen zusammenarbeiten. Um allen Bürgern den Zugang zum Sport zu ermöglichen, sind adäquate Sportstätten in ausreichender Anzahl Grundvoraussetzung. Ein für alle zugängliches und umfangreiches Sportangebot ist – vor allen Dingen in Großstädten und Ballungsgebieten – nur durch die Verfügbarkeit von ganzjährig nutzbaren Sportanlagen zu gewährleisten. Kunststoffrasenplätze spielen hierbei, insbesondere für den Fußball, eine wichtige Rolle, da sie eine intensivere Nutzung als Naturrasen- oder Tennenplätze erlauben. Allein mit Naturrasen- und Tennenplätzen lässt sich der derzeitige Trainings- und Spielbetrieb, insbesondere bei den Kinder- und Jugendmannschaften, nicht aufrechterhalten. Ein Kunststoffrasenplatz ersetzt etwa 2,5 Naturrasenplätze (DFBnet). Auf weniger als 10 Prozent der Naturrasenplätze finden an Wochenenden mehr als 2 Spiele statt. Bei Kunststoffrasenplätzen finden hingegen bei über 40 Prozent der Plätze mehr als 2 Spiele statt. Weniger als 10 Prozent der Naturrasenplätze wird an einem Wochenende mehr als 150 Minuten genutzt. Bei Kunstrasenplätzen werden hingegen etwa 35 Prozent an einem Wochenende mehr als 150 Minuten genutzt. 27.773 Spielstätten in Deutschland (ca. 70 Prozent) werden von Sportvereinen genutzt. Ein Drittel der Kunstrasenplätze werden von 2 oder mehr Vereinen mit alle ihren Jugend- und Seniorenmannschaften benutzt. Etwas über ein Drittel aller Naturrasenplätze wird von mehr als 5 Mannschaften bespielt. Bei Kunstrasenplätzen werden fast drei Viertel (72 Prozent) von mehr als 5 Mannschaften genutzt. Etwa 10 Prozent aller Naturrasenplätze wird von mehr als 10 Mannschaften bespielt. Bei Kunststoffrasenplätzen sind es ca. 41 Prozent der Plätze, die von mehr als 10 Mannschaften genutzt. Nur 1 Prozent aller Naturrasenplätze wird von mehr als 15 Mannschaften bespielt. Bei Kunstrasenplätzen beträgt der Anteil immerhin noch knapp 18 Prozent. Etwa 6 Prozent werden sogar von über 20 Mannschaften bespielt. Je größer die Vereinsgröße (insbesondere Anzahl der Mannschaften), desto höher ist der Anteil der Vereine, die auch eine Spielstätte vom Typ Kunstrasen haben.  Ein Verbot des Inverkehrbringens von Kunststoffgranulaten als Füllstoff in Kunststoffrasensystemen direkt bei Inkrafttreten der Beschränkung wäre daher unverhältnismäßig. Es würde zu hohen, unerwarteten Umstellungskosten und Mehrkosten für Vereine und Kommunen führen, wodurch dem gemeinwohlorientierten Sport Mittel entzogen würden. Bei fehlender Finanzierbarkeit dieser Mehrkosten ist zudem von einer Schließung vieler Sportplätzen auszugehen, wodurch das Sportangebot in Schulen und Vereinen stark leiden würde. Gerade auf Vereinsebene stellt ein solch außerordentlicher Kosten-punkt ein großes finanzielles Risiko dar, dass das sportliche und gesellschaftliche Gesamtangebot des Vereins gefährden kann. Eine Beschränkung ohne Übergangsfristen, die eine mittelfristige Umstellung und Kostenstreckung erlauben, würde das Breitensportangebot in Deutschland sehr negativ beeinflussen.  Im Hinblick auf den Beschränkungsvorschlag der ECHA gemäß Anhang XV der REACH-Verordnung spricht sich der DFB daher für eine angemessene Übergangsfrist von mindestens sechs Jahren bis zu einem vollständigen Inverkehrbringungsverbot des Kunststoffgranulats zur Verwendung in neuen Kunststoffrasensystemen sowie für die Umstellung bestehender Flächen aus.  Im Saarland wird auf 141 Kunstrasenplätzen gespielt. Ca. 44% aller Vereine mit Spielbetrieb verfügen über einen Kunstrasenplatz, der Rest verteilt sich auf Rasenplatz und Hartplatz (Tenne). |
| 23 | Spanish Professional Football League | It is fundamental for citizens to have access to good and sustainable sport facilities all year long.  In Spain 3.600 persons (out of 12.000 surveyed persons) practice sports to be in shape and the majority of them (11.160) do it at least once a week, regardless of whether it is holidays or school period of the year.  In these conditions, synthetic turf pitches play an important role as they can be used by citizens in a more intensive manner than natural turf pitches.  Additionally, there are increasingly more players in national University championships. For instance, 251 players took part in football championships in 2017. In the same championships but for rugby 333 players participated.  The use of synthetic turf with granules from ELT has increased in Spain in the last years. 140 pitches of LaLiga’s clubs members use synthetic turf pitch. This type of pitch is increasingly used in other sports like rugby and golf.  According to the latest statistics from the High Council for Sport in Spain, there are 8. 331 football pitches (p. 189), 5. 000 of which with synthetic turf. Additionally, there are 15.637 sports infrastructures dedicated mainly to football (p. 190). Hence the potential number of pitches with synthetic turf in Spain may be higher.  Municipal authorities, schools and universities are increasingly having synthetic turf pitches with ELT granules installed in their recreational areas.  However, we do not dispose of specific information on the number of these spaces and it is difficult to describe the impact on these facilities by the introduction of the prohibition. It is noteworthy that many of these facilities may be owned by public authorities. |  |  | LaLiga fully supports that microplastics and in particular the use of ELT granular in synthetic turf pitches needs to be examined. However, LaLiga believes as well that a gradual approach will accommodate public authorities’ objectives to protect and safeguard the environment and guarantee citizens, and in particular, children’s access to sport facilities.  An immediate prohibition of ELT granular in synthetic turf pitches may have a large social and economic impact that can prove to be disproportionate for all stakeholders involved, including in particular, citizens.  It is a crucial moment now for European, national and regional authorities as well as for manufacturers and sport associations to gather more data and conduct a proper assessment of the advantages and disadvantages of the use of ELT granular in synthetic turf pitches in comparison to the use of other alternatives and an evaluation of the costs thereof. |  |
| 24 | Sportbund Pfalz e. V. | a.: Nach neueren Erkenntnissen werden zwischen 0,25 t/a und 5 t/a in Deutschland an Befüllungsgranulat pro Kunststoffrasenfläche verwendet (Fraunhofer 2018, S. 11). Das entspricht einer Gesamtmenge von ca. 7.500 bis 9.900 t/a.  b.: Nach dem aktuellen Forschungsstand besteht nach Kenntnis des DFB ein hohes Maß an Unsicherheit darüber, wie und in welchen Mengen das als Mikroplastik definierte Granulat auf Sportplätzen in die Umwelt freigesetzt wird. Nach den uns zur Verfügung stehen-den Informationen gibt es große Unterschiede bei der Einschätzung der Menge an Mikroplastiken, die in den einzelnen Mitgliedstaaten oder in der EU/EWR als Füllmaterial für Kunstrasen verwendet wird. Insbesondere Umfang und Methodologie der Forschung in diesem Bereich sind bisher noch wenig standardisiert und nachvollziehbar. Der DFB geht davon aus, dass der Anteil des Eintrags von Mikroplastik über Kunststoffrasenplätze je nach Mitgliedstaat ca. 1 bis 3 Prozent im Verhältnis zum Gesamteintrag beträgt. Demnach ist der Umwelteintrag verglichen mit anderen Hauptquellen relativ gering (Europäische Kommission 2018, ii)). |  | c.: Gezielte Risikomanagementmaßnahmen können die Freisetzung von Füllstoffen in die Umwelt bereits signifikant vermindern. Technische Maßnahmen zur Zurückhaltung eines Materialaustrags vor Ort (z.B. Rinnenfilter mit Sedimentationsstrecken an Abläufen, Schmutzfangmatten, Schuhbürsten am Ausgang) und organisatorische Maßnahmen beim Betrieb der Sportplätze (z.B. regelmäßige Reinigung der Spielfeldränder, Auffangsiebe) können zu einer starken Verringerung des Austrags von Mikroplastik beitragen.  Neben dem häufig genutzten Kunststoffgranulat existieren für Kunststoffrasensysteme alternative Füllstoffe, die in Teilen auch bereits beim Betrieb von Sportanlagen genutzt werden. So werden in Deutschland aktuell Kunststoffrasenplätze teilweise mit Sand und/oder Kork verfüllt. Zudem gibt es auch Kunststoffrasensysteme, die ohne elastischen Füllstoff betrieben werden können.  Es existieren bisher allerdings nur wenige belastbare Studien darüber, wie sich diese Alternativen qualitäts- und kostenmäßig (z.B. hinsichtlich der Bespielbarkeit und Lebensdauer) vergleichen lassen. Zudem müsste untersucht werden, ob und wie sich die Bespielbar-keit oder das Verletzungsrisiko der alternativ befüllten Kunststoffrasenflächen bei den verschiedenen Alternativfüllungen verändert (Plan Miljø Studie 2017). Es bedarf daher dringend weiterer wissenschaftlicher Expertise zur Praxistauglichkeit alternativer organischer Füllstoffe und zur sportartspezifischen Eignung von Kunststoffrasenplätzen, die ohne Füllstoffe auskommen. Sowohl eine wissenschaftliche Folgenabschätzung als auch die dringend erforderliche Entwicklung alternativer Füllstoffe durch die Industrie sind eine zentrale Forderung der von der Thematik betroffenen Sportverbände in Deutschland. Sie vertreten die Meinung, dass die Maßnahmen, die ein Verbot des Kunststoffgranulats verursachen würden, nicht kurzfristig umsetzbar sind und Alternativen nur mittel- bis langfristig erarbeitet und bereitgestellt werden können. |  | d.: In Deutschland gibt es ca. 5.000 für den Fußballspielbetrieb gemeldete Kunststoffrasenplätze (DFBnet), sowie ca. 1.000 DFB-Minispielfelder. Jährlich werden in Deutschland ca. 300 Kunststoffrasenplätze neu gebaut, sowie 150 Kunststoffrasenplätze von Grund auf erneuert. Hinsichtlich der bestehenden Plätze dürfte eine Umstellung auf alternative Füllstoffe notwendig sein. Hierfür halten die Sportanlagenbetreiber (Kommunen oder Vereine) Mittel für Sportstättenbau und -sanierung vor, die bei einem vollständigen Ver-bot und einer Verwendung alternativer Füllstoffe deutlich höher ausfallen würden. Laut eigener Berechnungen belaufen sich die jährlichen Mehrkosten deutschlandweit auf einen hohen einstelligen Millionenbetrag. Die insgesamt zu erwartenden Kosten eines Verbotes können aufgrund fehlender Kenntnisse über geeignete alternative Füllstoffe (Geeignetheit, Verfügbarkeit) derzeit nicht seriös beziffert werden. Auf Grundlage aktueller Daten zum Bau von Kunststoffrasenplätzen dürfte der Gesamtbetrag für den Austausch des Füllstoffes der Kunststoffrasensysteme im hohen zweistelligen Millionenbereich (bis zu 90 Mio. EUR) liegen, wobei zur Präzisierung dieses Schätzwertes vertiefte Analysen erforderlich sind. Die Kosten für eine Umsetzung gezielter Risikomanagementmaßnahmen zur Zurückhaltung des Materialaustrags dürften nach Schätzungen und je nach Umfang der Maßnahmen pro Kunststoffrasensystem bei 3.000 bis 10.000 EUR liegen.  e.: Der gemeinwohlorientierte Sport ist die größte zivilgesellschaftliche Bewegung in Deutschland und Europa. In Deutschland engagieren sich knapp acht Millionen Bürger freiwillig und ehrenamtlich im Sport. Das entspricht einer jährlichen Wertschöpfung und einem Wohlfahrtsgewinn allein in Deutschland von ca. 6,7 Milliarden Euro. Vergleichbare Zahlen lassen sich auch für die gesamte EU feststellen. In den EU-Mitgliedstaaten engagieren sich im Jahre 2010 zwischen 92 und 94 Millionen Menschen freiwillig für Ziele des Gemeinwohls, davon die meisten im Sport (ca. 35 bis 40 Prozent aller freiwillig Tätigen in der EU) (Europäische Kommission 2010).  Der Sport schafft ein strukturiertes, an die gesamte Bevölkerung gerichtetes und für alle offenes Bewegungs- und Sportangebot, durch das wichtige soziale und gesundheitsfördernde Funktionen in der Gesellschaft erfüllt werden. Sportvereine in Deutschland zählen zehn Millionen Mitgliedschaften im Kinder- und Jugendalter (DOSB-Bestandserhebung 2018), allein im DFB liegt diese Zahl bei 2,1 Millionen (DFB-Mitgliederstatistik 2018). Damit sind Sportvereine die wichtigste Anlaufstelle für Kinder und Jugendliche außerhalb der Schule und übernehmen unverzichtbare Aufgaben für die ganzheitliche Persönlichkeitsbildung junger Menschen. Dem Sport kommt eine wichtige Vorbild- und Lehrfunktion im Bereich der Integration und demokratischen Grundbildung zu. Für das herausragende gesellschaftliche Engagement des Sports spricht nicht zuletzt, dass die Sportvereine eng mit Schulen, Kindergärten, Unternehmen, Krankenkassen oder anderen öffentlichen Institutionen zusammenarbeiten. Um allen Bürgern den Zugang zum Sport zu ermöglichen, sind adäquate Sportstätten in ausreichender Anzahl Grundvoraussetzung. Ein für alle zugängliches und umfangreiches Sportangebot ist – vor allen Dingen in Großstädten und Ballungsgebieten – nur durch die Verfügbarkeit von ganzjährig nutzbaren Sportanlagen zu gewährleisten. Kunststoffrasenplätze spielen hierbei, insbesondere für den Fußball, eine wichtige Rolle, da sie eine intensivere Nutzung als Naturrasen- oder Tennenplätze erlauben. Allein mit Naturrasen- und Tennenplätzen lässt sich der derzeitige Trainings- und Spielbetrieb, insbesondere bei den Kinder- und Jugendmannschaften, nicht aufrechterhalten. Ein Kunststoffrasenplatz ersetzt etwa 2,5 Naturrasenplätze (DFBnet). Auf weniger als 10 Prozent der Naturrasenplätze finden an Wochenenden mehr als 2 Spiele statt. Bei Kunststoffrasenplätzen finden hingegen bei über 40 Prozent der Plätze mehr als 2 Spiele statt. Weniger als 10 Prozent der Naturrasenplätze wird an einem Wochenende mehr als 150 Minuten genutzt. Bei Kunstrasenplätzen werden hingegen etwa 35 Prozent an einem Wochenende mehr als 150 Minuten genutzt. 27.773 Spielstätten in Deutschland (ca. 70 Prozent) werden von Sportvereinen genutzt. Ein Drittel der Kunstrasenplätze werden von 2 oder mehr Vereinen mit alle ihren Jugend- und Seniorenmannschaften benutzt. Etwas über ein Drittel aller Naturrasenplätze wird von mehr als 5 Mannschaften bespielt. Bei Kunstrasenplätzen werden fast drei Viertel (72 Prozent) von mehr als 5 Mannschaften genutzt. Etwa 10 Prozent aller Naturrasenplätze wird von mehr als 10 Mannschaften bespielt. Bei Kunststoffrasenplätzen sind es ca. 41 Prozent der Plätze, die von mehr als 10 Mannschaften genutzt. Nur 1 Prozent aller Naturrasenplätze wird von mehr als 15 Mannschaften bespielt. Bei Kunstrasenplätzen beträgt der Anteil immerhin noch knapp 18 Prozent. Etwa 6 Prozent werden sogar von über 20 Mannschaften bespielt. Je größer die Vereinsgröße (insbesondere Anzahl der Mannschaften), desto höher ist der Anteil der Vereine, die auch eine Spielstätte vom Typ Kunstrasen haben.  Ein Verbot des Inverkehrbringens von Kunststoffgranulaten als Füllstoff in Kunststoffrasensystemen direkt bei Inkrafttreten der Beschränkung wäre daher unverhältnismäßig. Es würde zu hohen, unerwarteten Umstellungskosten und Mehrkosten für Vereine und Kommunen führen, wodurch dem gemeinwohlorientierten Sport Mittel entzogen würden. Bei fehlender Finanzierbarkeit dieser Mehrkosten ist zudem von einer Schließung vieler Sportplätzen auszugehen, wodurch das Sportangebot in Schulen und Vereinen stark leiden würde. Gerade auf Vereinsebene stellt ein solch außerordentlicher Kosten-punkt ein großes finanzielles Risiko dar, dass das sportliche und gesellschaftliche Gesamtangebot des Vereins gefährden kann. Eine Beschränkung ohne Übergangsfristen, die eine mittelfristige Umstellung und Kostenstreckung erlauben, würde das Breitensportangebot in Deutschland sehr negativ beeinflussen.  Im Hinblick auf den Beschränkungsvorschlag der ECHA gemäß Anhang XV der REACH-Verordnung spricht sich der DFB daher für eine angemessene Übergangsfrist von mindestens sechs Jahren bis zu einem vollständigen Inverkehrbringungsverbot des Kunststoffgranulats zur Verwendung in neuen Kunststoffrasensystemen sowie für die Umstellung bestehender Flächen aus. |
| 25 | The English Football Association | Scope or restriction option analysis;  Baseline;  Information on alternatives;  Information on costs;  Information on benefits;  Other socio economic analysis (SEA) issues |  |  |  |  |
| 26 | Thüringer Fußball-Verband e.V. | a.: Nach neueren Erkenntnissen werden zwischen 0,25 t/a und 5 t/a in Deutschland an Befüllungsgranulat pro Kunststoffrasenfläche verwendet (Fraunhofer 2018, S. 11). Das entspricht einer Gesamtmenge von ca. 7.500 bis 9.900 t/a.  b.: Nach dem aktuellen Forschungsstand besteht nach Kenntnis des DFB ein hohes Maß an Unsicherheit darüber, wie und in welchen Mengen das als Mikroplastik definierte Granulat auf Sportplätzen in die Umwelt freigesetzt wird. Nach den uns zur Verfügung stehen-den Informationen gibt es große Unterschiede bei der Einschätzung der Menge an Mikroplastiken, die in den einzelnen Mitgliedstaaten oder in der EU/EWR als Füllmaterial für Kunstrasen verwendet wird. Insbesondere Umfang und Methodologie der Forschung in diesem Bereich sind bisher noch wenig standardisiert und nachvollziehbar. Der DFB geht davon aus, dass der Anteil des Eintrags von Mikroplastik über Kunststoffrasenplätze je nach Mitgliedstaat ca. 1 bis 3 Prozent im Verhältnis zum Gesamteintrag beträgt. Demnach ist der Umwelteintrag verglichen mit anderen Hauptquellen relativ gering (Europäische Kommission 2018, ii)). |  | c.: Gezielte Risikomanagementmaßnahmen können die Freisetzung von Füllstoffen in die Umwelt bereits signifikant vermindern. Technische Maßnahmen zur Zurückhaltung eines Materialaustrags vor Ort (z.B. Rinnenfilter mit Sedimentationsstrecken an Abläufen, Schmutzfangmatten, Schuhbürsten am Ausgang) und organisatorische Maßnahmen beim Betrieb der Sportplätze (z.B. regelmäßige Reinigung der Spielfeldränder, Auffangsiebe) können zu einer starken Verringerung des Austrags von Mikroplastik beitragen.  Neben dem häufig genutzten Kunststoffgranulat existieren für Kunststoffrasensysteme alternative Füllstoffe, die in Teilen auch bereits beim Betrieb von Sportanlagen genutzt werden. So werden in Deutschland aktuell Kunststoffrasenplätze teilweise mit Sand und/oder Kork verfüllt. Zudem gibt es auch Kunststoffrasensysteme, die ohne elastischen Füllstoff betrieben werden können.  Es existieren bisher allerdings nur wenige belastbare Studien darüber, wie sich diese Alternativen qualitäts- und kostenmäßig (z.B. hinsichtlich der Bespielbarkeit und Lebensdauer) vergleichen lassen. Zudem müsste untersucht werden, ob und wie sich die Bespielbar-keit oder das Verletzungsrisiko der alternativ befüllten Kunststoffrasenflächen bei den verschiedenen Alternativfüllungen verändert (Plan Miljø Studie 2017). Es bedarf daher dringend weiterer wissenschaftlicher Expertise zur Praxistauglichkeit alternativer organischer Füllstoffe und zur sportartspezifischen Eignung von Kunststoffrasenplätzen, die ohne Füllstoffe auskommen. Sowohl eine wissenschaftliche Folgenabschätzung als auch die dringend erforderliche Entwicklung alternativer Füllstoffe durch die Industrie sind eine zentrale Forderung der von der Thematik betroffenen Sportverbände in Deutschland. Sie vertreten die Meinung, dass die Maßnahmen, die ein Verbot des Kunststoffgranulats verursachen würden, nicht kurzfristig umsetzbar sind und Alternativen nur mittel- bis langfristig erarbeitet und bereitgestellt werden können. |  | d.: In Deutschland gibt es ca. 5.000 für den Fußballspielbetrieb gemeldete Kunststoffrasenplätze (DFBnet), sowie ca. 1.000 DFB-Minispielfelder. Jährlich werden in Deutschland ca. 300 Kunststoffrasenplätze neu gebaut, sowie 150 Kunststoffrasenplätze von Grund auf erneuert. Hinsichtlich der bestehenden Plätze dürfte eine Umstellung auf alternative Füllstoffe notwendig sein. Hierfür halten die Sportanlagenbetreiber (Kommunen oder Vereine) Mittel für Sportstättenbau und -sanierung vor, die bei einem vollständigen Ver-bot und einer Verwendung alternativer Füllstoffe deutlich höher ausfallen würden. Laut eigener Berechnungen belaufen sich die jährlichen Mehrkosten deutschlandweit auf einen hohen einstelligen Millionenbetrag. Die insgesamt zu erwartenden Kosten eines Verbotes können aufgrund fehlender Kenntnisse über geeignete alternative Füllstoffe (Geeignetheit, Verfügbarkeit) derzeit nicht seriös beziffert werden. Auf Grundlage aktueller Daten zum Bau von Kunststoffrasenplätzen dürfte der Gesamtbetrag für den Austausch des Füllstoffes der Kunststoffrasensysteme im hohen zweistelligen Millionenbereich (bis zu 90 Mio. EUR) liegen, wobei zur Präzisierung dieses Schätzwertes vertiefte Analysen erforderlich sind. Die Kosten für eine Umsetzung gezielter Risikomanagementmaßnahmen zur Zurückhaltung des Materialaustrags dürften nach Schätzungen und je nach Umfang der Maßnahmen pro Kunststoffrasensystem bei 3.000 bis 10.000 EUR liegen.  e.: Der gemeinwohlorientierte Sport ist die größte zivilgesellschaftliche Bewegung in Deutschland und Europa. In Deutschland engagieren sich knapp acht Millionen Bürger freiwillig und ehrenamtlich im Sport. Das entspricht einer jährlichen Wertschöpfung und einem Wohlfahrtsgewinn allein in Deutschland von ca. 6,7 Milliarden Euro. Vergleichbare Zahlen lassen sich auch für die gesamte EU feststellen. In den EU-Mitgliedstaaten engagieren sich im Jahre 2010 zwischen 92 und 94 Millionen Menschen freiwillig für Ziele des Gemeinwohls, davon die meisten im Sport (ca. 35 bis 40 Prozent aller freiwillig Tätigen in der EU) (Europäische Kommission 2010).  Der Sport schafft ein strukturiertes, an die gesamte Bevölkerung gerichtetes und für alle offenes Bewegungs- und Sportangebot, durch das wichtige soziale und gesundheitsfördernde Funktionen in der Gesellschaft erfüllt werden. Sportvereine in Deutschland zählen zehn Millionen Mitgliedschaften im Kinder- und Jugendalter (DOSB-Bestandserhebung 2018), allein im DFB liegt diese Zahl bei 2,1 Millionen (DFB-Mitgliederstatistik 2018). Damit sind Sportvereine die wichtigste Anlaufstelle für Kinder und Jugendliche außerhalb der Schule und übernehmen unverzichtbare Aufgaben für die ganzheitliche Persönlichkeitsbildung junger Menschen. Dem Sport kommt eine wichtige Vorbild- und Lehrfunktion im Bereich der Integration und demokratischen Grundbildung zu. Für das herausragende gesellschaftliche Engagement des Sports spricht nicht zuletzt, dass die Sportvereine eng mit Schulen, Kindergärten, Unternehmen, Krankenkassen oder anderen öffentlichen Institutionen zusammenarbeiten. Um allen Bürgern den Zugang zum Sport zu ermöglichen, sind adäquate Sportstätten in ausreichender Anzahl Grundvoraussetzung. Ein für alle zugängliches und umfangreiches Sportangebot ist – vor allen Dingen in Großstädten und Ballungsgebieten – nur durch die Verfügbarkeit von ganzjährig nutzbaren Sportanlagen zu gewährleisten. Kunststoffrasenplätze spielen hierbei, insbesondere für den Fußball, eine wichtige Rolle, da sie eine intensivere Nutzung als Naturrasen- oder Tennenplätze erlauben. Allein mit Naturrasen- und Tennenplätzen lässt sich der derzeitige Trainings- und Spielbetrieb, insbesondere bei den Kinder- und Jugendmannschaften, nicht aufrechterhalten. Ein Kunststoffrasenplatz ersetzt etwa 2,5 Naturrasenplätze (DFBnet). Auf weniger als 10 Prozent der Naturrasenplätze finden an Wochenenden mehr als 2 Spiele statt. Bei Kunststoffrasenplätzen finden hingegen bei über 40 Prozent der Plätze mehr als 2 Spiele statt. Weniger als 10 Prozent der Naturrasenplätze wird an einem Wochenende mehr als 150 Minuten genutzt. Bei Kunstrasenplätzen werden hingegen etwa 35 Prozent an einem Wochenende mehr als 150 Minuten genutzt. 27.773 Spielstätten in Deutschland (ca. 70 Prozent) werden von Sportvereinen genutzt. Ein Drittel der Kunstrasenplätze werden von 2 oder mehr Vereinen mit alle ihren Jugend- und Seniorenmannschaften benutzt. Etwas über ein Drittel aller Naturrasenplätze wird von mehr als 5 Mannschaften bespielt. Bei Kunstrasenplätzen werden fast drei Viertel (72 Prozent) von mehr als 5 Mannschaften genutzt. Etwa 10 Prozent aller Naturrasenplätze wird von mehr als 10 Mannschaften bespielt. Bei Kunststoffrasenplätzen sind es ca. 41 Prozent der Plätze, die von mehr als 10 Mannschaften genutzt. Nur 1 Prozent aller Naturrasenplätze wird von mehr als 15 Mannschaften bespielt. Bei Kunstrasenplätzen beträgt der Anteil immerhin noch knapp 18 Prozent. Etwa 6 Prozent werden sogar von über 20 Mannschaften bespielt. Je größer die Vereinsgröße (insbesondere Anzahl der Mannschaften), desto höher ist der Anteil der Vereine, die auch eine Spielstätte vom Typ Kunstrasen haben.  Ein Verbot des Inverkehrbringens von Kunststoffgranulaten als Füllstoff in Kunststoffrasensystemen direkt bei Inkrafttreten der Beschränkung wäre daher unverhältnismäßig. Es würde zu hohen, unerwarteten Umstellungskosten und Mehrkosten für Vereine und Kommunen führen, wodurch dem gemeinwohlorientierten Sport Mittel entzogen würden. Bei fehlender Finanzierbarkeit dieser Mehrkosten ist zudem von einer Schließung vieler Sportplätzen auszugehen, wodurch das Sportangebot in Schulen und Vereinen stark leiden würde. Gerade auf Vereinsebene stellt ein solch außerordentlicher Kosten-punkt ein großes finanzielles Risiko dar, dass das sportliche und gesellschaftliche Gesamtangebot des Vereins gefährden kann. Eine Beschränkung ohne Übergangsfristen, die eine mittelfristige Umstellung und Kostenstreckung erlauben, würde das Breitensportangebot in Deutschland sehr negativ beeinflussen.  Im Hinblick auf den Beschränkungsvorschlag der ECHA gemäß Anhang XV der REACH-Verordnung spricht sich der DFB daher für eine angemessene Übergangsfrist von mindestens sechs Jahren bis zu einem vollständigen Inverkehrbringungsverbot des Kunststoffgranulats zur Verwendung in neuen Kunststoffrasensystemen sowie für die Umstellung bestehender Flächen aus. |
| 27 | Westdeutscher Fußballverband e.V. | a.: Nach neueren Erkenntnissen werden zwischen 0,25 t/a und 5 t/a in Deutschland an Befüllungsgranulat pro Kunststoffrasenfläche verwendet (Fraunhofer 2018, S. 11). Das entspricht einer Gesamtmenge von ca. 7.500 bis 9.900 t/a.  b.: Nach dem aktuellen Forschungsstand besteht nach Kenntnis des DFB ein hohes Maß an Unsicherheit darüber, wie und in welchen Mengen das als Mikroplastik definierte Granulat auf Sportplätzen in die Umwelt freigesetzt wird. Nach den uns zur Verfügung stehen-den Informationen gibt es große Unterschiede bei der Einschätzung der Menge an Mikroplastiken, die in den einzelnen Mitgliedstaaten oder in der EU/EWR als Füllmaterial für Kunstrasen verwendet wird. Insbesondere Umfang und Methodologie der Forschung in diesem Bereich sind bisher noch wenig standardisiert und nachvollziehbar. Der DFB geht davon aus, dass der Anteil des Eintrags von Mikroplastik über Kunststoffrasenplätze je nach Mitgliedstaat ca. 1 bis 3 Prozent im Verhältnis zum Gesamteintrag beträgt. Demnach ist der Umwelteintrag verglichen mit anderen Hauptquellen relativ gering (Europäische Kommission 2018, ii)). |  | c.: Gezielte Risikomanagementmaßnahmen können die Freisetzung von Füllstoffen in die Umwelt bereits signifikant vermindern. Technische Maßnahmen zur Zurückhaltung eines Materialaustrags vor Ort (z.B. Rinnenfilter mit Sedimentationsstrecken an Abläufen, Schmutzfangmatten, Schuhbürsten am Ausgang) und organisatorische Maßnahmen beim Betrieb der Sportplätze (z.B. regelmäßige Reinigung der Spielfeldränder, Auffangsiebe) können zu einer starken Verringerung des Austrags von Mikroplastik beitragen.  Neben dem häufig genutzten Kunststoffgranulat existieren für Kunststoffrasensysteme alternative Füllstoffe, die in Teilen auch bereits beim Betrieb von Sportanlagen genutzt werden. So werden in Deutschland aktuell Kunststoffrasenplätze teilweise mit Sand und/oder Kork verfüllt. Zudem gibt es auch Kunststoffrasensysteme, die ohne elastischen Füllstoff betrieben werden können.  Es existieren bisher allerdings nur wenige belastbare Studien darüber, wie sich diese Alternativen qualitäts- und kostenmäßig (z.B. hinsichtlich der Bespielbarkeit und Lebensdauer) vergleichen lassen. Zudem müsste untersucht werden, ob und wie sich die Bespielbar-keit oder das Verletzungsrisiko der alternativ befüllten Kunststoffrasenflächen bei den verschiedenen Alternativfüllungen verändert (Plan Miljø Studie 2017). Es bedarf daher dringend weiterer wissenschaftlicher Expertise zur Praxistauglichkeit alternativer organischer Füllstoffe und zur sportartspezifischen Eignung von Kunststoffrasenplätzen, die ohne Füllstoffe auskommen. Sowohl eine wissenschaftliche Folgenabschätzung als auch die dringend erforderliche Entwicklung alternativer Füllstoffe durch die Industrie sind eine zentrale Forderung der von der Thematik betroffenen Sportverbände in Deutschland. Sie vertreten die Meinung, dass die Maßnahmen, die ein Verbot des Kunststoffgranulats verursachen würden, nicht kurzfristig umsetzbar sind und Alternativen nur mittel- bis langfristig erarbeitet und bereitgestellt werden können. |  | d.: In Deutschland gibt es ca. 5.000 für den Fußballspielbetrieb gemeldete Kunststoffrasenplätze (DFBnet), sowie ca. 1.000 DFB-Minispielfelder. Jährlich werden in Deutschland ca. 300 Kunststoffrasenplätze neu gebaut, sowie 150 Kunststoffrasenplätze von Grund auf erneuert. Hinsichtlich der bestehenden Plätze dürfte eine Umstellung auf alternative Füllstoffe notwendig sein. Hierfür halten die Sportanlagenbetreiber (Kommunen oder Vereine) Mittel für Sportstättenbau und -sanierung vor, die bei einem vollständigen Ver-bot und einer Verwendung alternativer Füllstoffe deutlich höher ausfallen würden. Laut eigener Berechnungen belaufen sich die jährlichen Mehrkosten deutschlandweit auf einen hohen einstelligen Millionenbetrag. Die insgesamt zu erwartenden Kosten eines Verbotes können aufgrund fehlender Kenntnisse über geeignete alternative Füllstoffe (Geeignetheit, Verfügbarkeit) derzeit nicht seriös beziffert werden. Auf Grundlage aktueller Daten zum Bau von Kunststoffrasenplätzen dürfte der Gesamtbetrag für den Austausch des Füllstoffes der Kunststoffrasensysteme im hohen zweistelligen Millionenbereich (bis zu 90 Mio. EUR) liegen, wobei zur Präzisierung dieses Schätzwertes vertiefte Analysen erforderlich sind. Die Kosten für eine Umsetzung gezielter Risikomanagementmaßnahmen zur Zurückhaltung des Materialaustrags dürften nach Schätzungen und je nach Umfang der Maßnahmen pro Kunststoffrasensystem bei 3.000 bis 10.000 EUR liegen.  e.: Der gemeinwohlorientierte Sport ist die größte zivilgesellschaftliche Bewegung in Deutschland und Europa. In Deutschland engagieren sich knapp acht Millionen Bürger freiwillig und ehrenamtlich im Sport. Das entspricht einer jährlichen Wertschöpfung und einem Wohlfahrtsgewinn allein in Deutschland von ca. 6,7 Milliarden Euro. Vergleichbare Zahlen lassen sich auch für die gesamte EU feststellen. In den EU-Mitgliedstaaten engagieren sich im Jahre 2010 zwischen 92 und 94 Millionen Menschen freiwillig für Ziele des Gemeinwohls, davon die meisten im Sport (ca. 35 bis 40 Prozent aller freiwillig Tätigen in der EU) (Europäische Kommission 2010). Der Westdeutsche Fußballverband e. V. (WDFV) ist der größte Fachsportverband in Nordrhein-Westfalen. Er vertritt mit seinen drei Mitgliedsverbänden mehr als 1,6 Millionen Sportler in rund 4.600 Vereinen.  Der Sport schafft ein strukturiertes, an die gesamte Bevölkerung gerichtetes und für alle offenes Bewegungs- und Sportangebot, durch das wichtige soziale und gesundheitsfördernde Funktionen in der Gesellschaft erfüllt werden. Sportvereine in Deutschland zählen zehn Millionen Mitgliedschaften im Kinder- und Jugendalter (DOSB-Bestandserhebung 2018), allein im DFB liegt diese Zahl bei 2,1 Millionen (DFB-Mitgliederstatistik 2018). Damit sind Sportvereine die wichtigste Anlaufstelle für Kinder und Jugendliche außerhalb der Schule und übernehmen unverzichtbare Aufgaben für die ganzheitliche Persönlichkeitsbildung junger Menschen. Dem Sport kommt eine wichtige Vorbild- und Lehrfunktion im Bereich der Integration und demokratischen Grundbildung zu. Für das herausragende gesellschaftliche Engagement des Sports spricht nicht zuletzt, dass die Sportvereine eng mit Schulen, Kindergärten, Unternehmen, Krankenkassen oder anderen öffentlichen Institutionen zusammenarbeiten. Um allen Bürgern den Zugang zum Sport zu ermöglichen, sind adäquate Sportstätten in ausreichender Anzahl Grundvoraussetzung. Ein für alle zugängliches und umfangreiches Sportangebot ist – vor allen Dingen in Großstädten und Ballungsgebieten – nur durch die Verfügbarkeit von ganzjährig nutzbaren Sportanlagen zu gewährleisten. Kunststoffrasenplätze spielen hierbei, insbesondere für den Fußball, eine wichtige Rolle, da sie eine intensivere Nutzung als Naturrasen- oder Tennenplätze erlauben. Allein mit Naturrasen- und Tennenplätzen lässt sich der derzeitige Trainings- und Spielbetrieb, insbesondere bei den Kinder- und Jugendmannschaften, nicht aufrechterhalten. Ein Kunststoffrasenplatz ersetzt etwa 2,5 Naturrasenplätze (DFBnet). Auf weniger als 10 Prozent der Naturrasenplätze finden an Wochenenden mehr als 2 Spiele statt. Bei Kunststoffrasenplätzen finden hingegen bei über 40 Prozent der Plätze mehr als 2 Spiele statt. Weniger als 10 Prozent der Naturrasenplätze wird an einem Wochenende mehr als 150 Minuten genutzt. Bei Kunstrasenplätzen werden hingegen etwa 35 Prozent an einem Wochenende mehr als 150 Minuten genutzt. 27.773 Spielstätten in Deutschland (ca. 70 Prozent) werden von Sportvereinen genutzt. Ein Drittel der Kunstrasenplätze werden von 2 oder mehr Vereinen mit alle ihren Jugend- und Seniorenmannschaften benutzt. Etwas über ein Drittel aller Naturrasenplätze wird von mehr als 5 Mannschaften bespielt. Bei Kunstrasenplätzen werden fast drei Viertel (72 Prozent) von mehr als 5 Mannschaften genutzt. Etwa 10 Prozent aller Naturrasenplätze wird von mehr als 10 Mannschaften bespielt. Bei Kunststoffrasenplätzen sind es ca. 41 Prozent der Plätze, die von mehr als 10 Mannschaften genutzt. Nur 1 Prozent aller Naturrasenplätze wird von mehr als 15 Mannschaften bespielt. Bei Kunstrasenplätzen beträgt der Anteil immerhin noch knapp 18 Prozent. Etwa 6 Prozent werden sogar von über 20 Mannschaften bespielt. Je größer die Vereinsgröße (insbesondere Anzahl der Mannschaften), desto höher ist der Anteil der Vereine, die auch eine Spielstätte vom Typ Kunstrasen haben.  Ein Verbot des Inverkehrbringens von Kunststoffgranulaten als Füllstoff in Kunststoffrasensystemen direkt bei Inkrafttreten der Beschränkung wäre daher unverhältnismäßig. Es würde zu hohen, unerwarteten Umstellungskosten und Mehrkosten für Vereine und Kommunen führen, wodurch dem gemeinwohlorientierten Sport Mittel entzogen würden. Bei fehlender Finanzierbarkeit dieser Mehrkosten ist zudem von einer Schließung vieler Sportplätzen auszugehen, wodurch das Sportangebot in Schulen und Vereinen stark leiden würde. Gerade auf Vereinsebene stellt ein solch außerordentlicher Kosten-punkt ein großes finanzielles Risiko dar, dass das sportliche und gesellschaftliche Gesamtangebot des Vereins gefährden kann. Eine Beschränkung ohne Übergangsfristen, die eine mittelfristige Umstellung und Kostenstreckung erlauben, würde das Breitensportangebot in Deutschland sehr negativ beeinflussen.  Im Hinblick auf den Beschränkungsvorschlag der ECHA gemäß Anhang XV der REACH-Verordnung spricht sich der DFB daher für eine angemessene Übergangsfrist von mindestens sechs Jahren bis zu einem vollständigen Inverkehrbringungsverbot des Kunststoffgranulats zur Verwendung in neuen Kunststoffrasensystemen sowie für die Umstellung bestehender Flächen aus. |
| 28 | Württembergischer Fußballverband | a.: Nach neueren Erkenntnissen werden zwischen 0,25 t/a und 5 t/a in Deutschland an Befüllungsgranulat pro Kunststoffrasenfläche verwendet (Fraunhofer 2018, S. 11). Das entspricht einer Gesamtmenge von ca. 7.500 bis 9.900 t/a.  b.: Nach dem aktuellen Forschungsstand besteht nach Kenntnis des DFB ein hohes Maß an Unsicherheit darüber, wie und in welchen Mengen das als Mikroplastik definierte Granulat auf Sportplätzen in die Umwelt freigesetzt wird. Nach den uns zur Verfügung stehen-den Informationen gibt es große Unterschiede bei der Einschätzung der Menge an Mikroplastiken, die in den einzelnen Mitgliedstaaten oder in der EU/EWR als Füllmaterial für Kunstrasen verwendet wird. Insbesondere Umfang und Methodologie der Forschung in diesem Bereich sind bisher noch wenig standardisiert und nachvollziehbar. Der DFB geht davon aus, dass der Anteil des Eintrags von Mikroplastik über Kunststoffrasenplätze je nach Mitgliedstaat ca. 1 bis 3 Prozent im Verhältnis zum Gesamteintrag beträgt. Demnach ist der Umwelteintrag verglichen mit anderen Hauptquellen relativ gering (Europäische Kommission 2018, ii)). |  | c.: Gezielte Risikomanagementmaßnahmen können die Freisetzung von Füllstoffen in die Umwelt bereits signifikant vermindern. Technische Maßnahmen zur Zurückhaltung eines Materialaustrags vor Ort (z.B. Rinnenfilter mit Sedimentationsstrecken an Abläufen, Schmutzfangmatten, Schuhbürsten am Ausgang) und organisatorische Maßnahmen beim Betrieb der Sportplätze (z.B. regelmäßige Reinigung der Spielfeldränder, Auffangsiebe) können zu einer starken Verringerung des Austrags von Mikroplastik beitragen.  Neben dem häufig genutzten Kunststoffgranulat existieren für Kunststoffrasensysteme alternative Füllstoffe, die in Teilen auch bereits beim Betrieb von Sportanlagen genutzt werden. So werden in Deutschland aktuell Kunststoffrasenplätze teilweise mit Sand und/oder Kork verfüllt. Zudem gibt es auch Kunststoffrasensysteme, die ohne elastischen Füllstoff betrieben werden können.  Es existieren bisher allerdings nur wenige belastbare Studien darüber, wie sich diese Alternativen qualitäts- und kostenmäßig (z.B. hinsichtlich der Bespielbarkeit und Lebensdauer) vergleichen lassen. Zudem müsste untersucht werden, ob und wie sich die Bespielbar-keit oder das Verletzungsrisiko der alternativ befüllten Kunststoffrasenflächen bei den verschiedenen Alternativfüllungen verändert (Plan Miljø Studie 2017). Es bedarf daher dringend weiterer wissenschaftlicher Expertise zur Praxistauglichkeit alternativer organischer Füllstoffe und zur sportartspezifischen Eignung von Kunststoffrasenplätzen, die ohne Füllstoffe auskommen. Sowohl eine wissenschaftliche Folgenabschätzung als auch die dringend erforderliche Entwicklung alternativer Füllstoffe durch die Industrie sind eine zentrale Forderung der von der Thematik betroffenen Sportverbände in Deutschland. Sie vertreten die Meinung, dass die Maßnahmen, die ein Verbot des Kunststoffgranulats verursachen würden, nicht kurzfristig umsetzbar sind und Alternativen nur mittel- bis langfristig erarbeitet und bereitgestellt werden können. |  | d.: In Deutschland gibt es ca. 5.000 für den Fußballspielbetrieb gemeldete Kunststoffrasenplätze (DFBnet), sowie ca. 1.000 DFB-Minispielfelder. Jährlich werden in Deutschland ca. 300 Kunststoffrasenplätze neu gebaut, sowie 150 Kunststoffrasenplätze von Grund auf erneuert. Hinsichtlich der bestehenden Plätze dürfte eine Umstellung auf alternative Füllstoffe notwendig sein. Hierfür halten die Sportanlagenbetreiber (Kommunen oder Vereine) Mittel für Sportstättenbau und -sanierung vor, die bei einem vollständigen Ver-bot und einer Verwendung alternativer Füllstoffe deutlich höher ausfallen würden. Laut eigener Berechnungen belaufen sich die jährlichen Mehrkosten deutschlandweit auf einen hohen einstelligen Millionenbetrag. Die insgesamt zu erwartenden Kosten eines Verbotes können aufgrund fehlender Kenntnisse über geeignete alternative Füllstoffe (Geeignetheit, Verfügbarkeit) derzeit nicht seriös beziffert werden. Auf Grundlage aktueller Daten zum Bau von Kunststoffrasenplätzen dürfte der Gesamtbetrag für den Austausch des Füllstoffes der Kunststoffrasensysteme im hohen zweistelligen Millionenbereich (bis zu 90 Mio. EUR) liegen, wobei zur Präzisierung dieses Schätzwertes vertiefte Analysen erforderlich sind. Die Kosten für eine Umsetzung gezielter Risikomanagementmaßnahmen zur Zurückhaltung des Materialaustrags dürften nach Schätzungen und je nach Umfang der Maßnahmen pro Kunststoffrasensystem bei 3.000 bis 10.000 EUR liegen.  e.: Der gemeinwohlorientierte Sport ist die größte zivilgesellschaftliche Bewegung in Deutschland und Europa. In Deutschland engagieren sich knapp acht Millionen Bürger freiwillig und ehrenamtlich im Sport. Das entspricht einer jährlichen Wertschöpfung und einem Wohlfahrtsgewinn allein in Deutschland von ca. 6,7 Milliarden Euro. Vergleichbare Zahlen lassen sich auch für die gesamte EU feststellen. In den EU-Mitgliedstaaten engagieren sich im Jahre 2010 zwischen 92 und 94 Millionen Menschen freiwillig für Ziele des Gemeinwohls, davon die meisten im Sport (ca. 35 bis 40 Prozent aller freiwillig Tätigen in der EU) (Europäische Kommission 2010).  Der Sport schafft ein strukturiertes, an die gesamte Bevölkerung gerichtetes und für alle offenes Bewegungs- und Sportangebot, durch das wichtige soziale und gesundheitsfördernde Funktionen in der Gesellschaft erfüllt werden. Sportvereine in Deutschland zählen zehn Millionen Mitgliedschaften im Kinder- und Jugendalter (DOSB-Bestandserhebung 2018), allein im DFB liegt diese Zahl bei 2,1 Millionen (DFB-Mitgliederstatistik 2018). Damit sind Sportvereine die wichtigste Anlaufstelle für Kinder und Jugendliche außerhalb der Schule und übernehmen unverzichtbare Aufgaben für die ganzheitliche Persönlichkeitsbildung junger Menschen. Dem Sport kommt eine wichtige Vorbild- und Lehrfunktion im Bereich der Integration und demokratischen Grundbildung zu. Für das herausragende gesellschaftliche Engagement des Sports spricht nicht zuletzt, dass die Sportvereine eng mit Schulen, Kindergärten, Unternehmen, Krankenkassen oder anderen öffentlichen Institutionen zusammenarbeiten. Um allen Bürgern den Zugang zum Sport zu ermöglichen, sind adäquate Sportstätten in ausreichender Anzahl Grundvoraussetzung. Ein für alle zugängliches und umfangreiches Sportangebot ist – vor allen Dingen in Großstädten und Ballungsgebieten – nur durch die Verfügbarkeit von ganzjährig nutzbaren Sportanlagen zu gewährleisten. Kunststoffrasenplätze spielen hierbei, insbesondere für den Fußball, eine wichtige Rolle, da sie eine intensivere Nutzung als Naturrasen- oder Tennenplätze erlauben. Allein mit Naturrasen- und Tennenplätzen lässt sich der derzeitige Trainings- und Spielbetrieb, insbesondere bei den Kinder- und Jugendmannschaften, nicht aufrechterhalten. Ein Kunststoffrasenplatz ersetzt etwa 2,5 Naturrasenplätze (DFBnet). Auf weniger als 10 Prozent der Naturrasenplätze finden an Wochenenden mehr als 2 Spiele statt. Bei Kunststoffrasenplätzen finden hingegen bei über 40 Prozent der Plätze mehr als 2 Spiele statt. Weniger als 10 Prozent der Naturrasenplätze wird an einem Wochenende mehr als 150 Minuten genutzt. Bei Kunstrasenplätzen werden hingegen etwa 35 Prozent an einem Wochenende mehr als 150 Minuten genutzt. 27.773 Spielstätten in Deutschland (ca. 70 Prozent) werden von Sportvereinen genutzt. Ein Drittel der Kunstrasenplätze werden von 2 oder mehr Vereinen mit alle ihren Jugend- und Seniorenmannschaften benutzt. Etwas über ein Drittel aller Naturrasenplätze wird von mehr als 5 Mannschaften bespielt. Bei Kunstrasenplätzen werden fast drei Viertel (72 Prozent) von mehr als 5 Mannschaften genutzt. Etwa 10 Prozent aller Naturrasenplätze wird von mehr als 10 Mannschaften bespielt. Bei Kunststoffrasenplätzen sind es ca. 41 Prozent der Plätze, die von mehr als 10 Mannschaften genutzt. Nur 1 Prozent aller Naturrasenplätze wird von mehr als 15 Mannschaften bespielt. Bei Kunstrasenplätzen beträgt der Anteil immerhin noch knapp 18 Prozent. Etwa 6 Prozent werden sogar von über 20 Mannschaften bespielt. Je größer die Vereinsgröße (insbesondere Anzahl der Mannschaften), desto höher ist der Anteil der Vereine, die auch eine Spielstätte vom Typ Kunstrasen haben. Zur Aufrechterhaltung des Spielbetriebs für unsere ca. 13.000 Mannschaften in rund 1.600 Vereine sind Kunstrasenplätze von elementarer Bedeutung.  Ein Verbot des Inverkehrbringens von Kunststoffgranulaten als Füllstoff in Kunststoffrasensystemen direkt bei Inkrafttreten der Beschränkung wäre daher unverhältnismäßig. Es würde zu hohen, unerwarteten Umstellungskosten und Mehrkosten für Vereine und Kommunen führen, wodurch dem gemeinwohlorientierten Sport Mittel entzogen würden. Bei fehlender Finanzierbarkeit dieser Mehrkosten ist zudem von einer Schließung vieler Sportplätzen auszugehen, wodurch das Sportangebot in Schulen und Vereinen stark leiden würde. Gerade auf Vereinsebene stellt ein solch außerordentlicher Kosten-punkt ein großes finanzielles Risiko dar, dass das sportliche und gesellschaftliche Gesamtangebot des Vereins gefährden kann. Eine Beschränkung ohne Übergangsfristen, die eine mittelfristige Umstellung und Kostenstreckung erlauben, würde das Breitensportangebot in Deutschland sehr negativ beeinflussen.  Im Hinblick auf den Beschränkungsvorschlag der ECHA gemäß Anhang XV der REACH-Verordnung spricht sich der DFB daher für eine angemessene Übergangsfrist von mindestens sechs Jahren bis zu einem vollständigen Inverkehrbringungsverbot des Kunststoffgranulats zur Verwendung in neuen Kunststoffrasensystemen sowie für die Umstellung bestehender Flächen aus. |

**References**

Blériot, J., 2017, Microplastics, macro problems, EURACTIV, Link: https://www.euractiv.com/section/circular-economy/opinion/microplastics-macro-problems/ - accessed 22-10-2019

# Burrows, D., 2017, Microplastics threat poses dilemma for new EU strategy, EUobserver, Link: https://euobserver.com/health/140194 - accessed 22-10-2019

ECHA, 2019, General Comments and answers to specific information requests, Helsinki: European Chemicals Agency, Link: https://echa.europa.eu/registry-of-restriction-intentions/-/dislist/details/0b0236e18244cd73 - accessed 28-10-2019

Ends, 2018c, EU ‘failing’ to regulate plastic pollution, Link: https://www.endseurope.com/article/53920/eu-failing-to-regulate-plastic-pollution - accessed 22-10-2019

Ends, 2013b, World Water Week puts spotlight on chemical use, Ends, Link: https://www.endseurope.com/article/32984/world-water-week-puts-spotlight-on-chemical-use - accessed 22-10-2019

Oziel, C., 2019, Top German sports associations call for artificial turf ban transition, ChemicalWatch, Link: <https://chemicalwatch.com/77732/top-german-sports-associations-call-for-artificial-turf-ban-transition?q=microPlastics> - accessed 11-6-2019.
