# Supplementary material for: Stakeholder analysis with regard to a recent European restriction proposal on microplastics
Source: PLoS One. 2020 Jun 22;15(6):e0235062. doi: 10.1371/journal.pone.0235062 (PMC7307934; doi:10.1371/journal.pone.0235062)
Supplement: S8 Table — (DOCX) [file pone.0235062.s009.docx]

S8 Table: SMEs categorization table

| ***SMEs*** | | | | | | |
| --- | --- | --- | --- | --- | --- | --- |
| **No**. | **Stakeholder** | **Criteria**  (for statement e.g. economy, innovation, environment etc.) | **Principles**  (separate from criteria e.g. PP, values etc.p) | **Scientific argumentation**   1. Case Reports & Case Series (observational) 2. Case-control (observational) 3. Cohort (observational) 4. Randomized-controlled trials (experimental) 5. Systematic review | **Research needs**  (identified needs in statement) | **Other** |
| 1 | Anthesis-Caleb (Consultant) | Paul Ashford, Managing Director Anthesis-Caleb says the EU faces a conundrum as it assesses potential options involving plastic (Ashford, 2019).  …“*the discussion on what one is has moved centre-stage. Debate on the definition is still ongoing, but most of it has focused on physical form. Little attention has been paid to the chemical composition outside of biodegradability and source (natural v. synthetic), both of which are polymeric properties rather than identifiers*” (Ashford, 2019). |  |  |  |  |
| 2 | Geholit+Wiemer Lack- und Kunststoff-Chemie GmbH (Germany) | Geholit+Wiemer GmbH is a medium size manufacturer of paints and coatings for industrial and craftsmen uses. The proposed restriction imposes on us new labelling and reporting requirements.  The reporting requirements present technical and financial burden to us which seems pointless, as we are not able to collect the information stipulated. The additional labelling will not have any beneficial effects on microplastics releases because environmentally sound waste treatment routines are already well established in all areas we deliver to.  1. Scope: Other than in cosmetic rinse of products, paints and coatings are not intended to be re-leased into the environment but to stay on the treated surfaces. During application all microplastic particles as referred to in the restriction proposal are bound into a solid matrix and clearly fall un-der derogation 5.b of the restriction.  2. Information collection: Detailed information about the polymers we use in our coatings is generally not provided by our suppliers as it is confidential business information. We usually receive only vague indications of the chemical composition. We have literally no information about the environmental releases that might occur during use or cleaning acitivities, although we are quite confident about the environmentally conscious behavior of our customers. Since non-industrial professionals are exempt from reporting requirements, information on releases cannot be collected. Information given in a report to ECHA would solely comprise ill-founded assumptions and estimates.  3. Labelling: Already instructions on waste treatment of our products are provided in section 6 (acci-dental release measures) and section 13 (disposal considerations) of our safety data sheets. Re-peating this information on a label will have no additional value, but will lead to overcrowded labels where important information about classification of the product should be prominent.  Legal requirements on waste disposal or treatment are already in place for industrial settings. Releases to the environment are therefore unlikely and/or minimized.  Craftsmen and other professional users of our products also have well-established working and disposal routines. New labels will have no effect on these routines.  Geholit+Wiemer is well aware of the environmental impacts of microplastics and strives in his own work-ings and in technical advice to our customers for minimization of environmental releases. To further this aspect additional, more detailed, information on cleaning procedures of machinery and equipment could be provided in the safety data sheets and be made mandatory through implementation in annex II of the REACH regulation. |  |  |  |  |
| 3 | Hyga GmbH & Co. KG (Germany) | It is possible to Change the recipes, but it is difficult. Because you have Change the complet recipes.  It is not possible to Change some Microplastic ingredients to an other ingredients. Because some of the Microplastic ingredients have an Special function. So you have to Change all ingredients in the recipe. |  |  |  |  |
| 4 | Italian SMEs |  |  |  | *“Italian SMEs in the cosmetics industry have raised*[*concerns*](https://chemicalwatch.com/74140)*about the potential economic impact of Echa’s microplastics restriction*[*proposal*](https://chemicalwatch.com/73819)*on their business”* (Tani, 2019). |  |

**References**

Ashford, P., 2019, Guest Column: Dealing with polymers under REACH, ChemicalWatch, Link: https://chemicalwatch.com/72674/guest-column-dealing-with-polymers-under-reach?q=microPlastics - accessed 8-8-2019.

ECHA, 2019, General Comments and answers to specific information requests, Helsinki: European Chemicals Agency, Link: <https://echa.europa.eu/registry-of-restriction-intentions/-/dislist/details/0b0236e18244cd73> - accessed 28-10-2019

Tani, C., 2019, Italian cosmetics producer SMEs braced for proposed EU microplastics restriction, ChemicalWatch, Link: https://chemicalwatch.com/78730/italian-cosmetics-producer-smes-braced-for-proposed-eu-microplastics-restriction?q=microPlastics - accessed 11-6-2019.
