# Supplementary material for: Stakeholder analysis with regard to a recent European restriction proposal on microplastics
Source: PLoS One. 2020 Jun 22;15(6):e0235062. doi: 10.1371/journal.pone.0235062 (PMC7307934; doi:10.1371/journal.pone.0235062)
Supplement: S10 Table — (DOCX) [file pone.0235062.s011.docx]

S10 Table: International NGOs microplastics comments

| **Stakeholder** | **Date** | **Expressed interests/opinion on microplastics at CW, Ends, EURACTIV, EUObserver** |
| --- | --- | --- |
| WFF European Policy Office  New Economics Foundation & Zero Waste Europe  Seas at Risk  Cousteau Divers | 13-11-2018  4-9-2018  7-2-2018  9-12-2017 | Andreas Baumüller, Head of Natural Resources at the WWF European Policy Office: *“Healthy rivers, lakes and wetlands are our life support system, but EU member states are trying to destroy the law that protects them and the window to save them is closing”* (Baumüller, 2018)  David Powell, head of Environment & Green Transition at the New Economics Foundation & Ariadna Rodrigo is Sustainable Products Campaigner at Zero Waste Europe: “none of the proposals or legislation tabled comes close to addressing the depth of our reliance on plastic. As argued i[n our new report published today](http://zerowasteeurope.eu/wp-content/uploads/2018/09/PlasticsTax_FINAL.pdf), we urgently need to look at the potential for new taxes to change behaviour and shift economic incentives for industry and consumers alike” (Rodrigo & Powell, 2018)  Emma Priestland, Seas at Risk: “Those that are added intentionally would have to be registered under REACH for the first time. For those that are generated, the Commission is examining options such as labelling and minimum requirements for product design and durability” (Keating, 2018)  Pierre-Yves Cousteau, CEO, Cousteau Divers: “The slogan Reduce – Reuse – Recycle is currently broken, as re-used and recycled materials continue to release invisible plastic particles that we breathe in and eat. You and I, in our daily lives, can already have an impact by refusing single-use plastic.” (Cousteau, 2017) |
| European Environmental Bureau, ClientEarth, ChemSec, and Greenpeace | 11 Jun 2018 | The campaigners had said the call focused too much on asking industry to submit information to exclude certain uses from restrictions, and “did not show interest in receiving information from other stakeholders about the hazards and risks of microplastics”. (European Environmental Bureau, ClientEarth, ChemSec, and Greenpeace). (ends 2018g) |
|  | 6 Jun 2018 | The campaigners say that while the agency invited industries using microplastics to provide information so it could exclude certain uses from restrictions, it “did not show interest in receiving information from other stakeholders about the hazards and risks of microplastics”.  The agency showed an “excessive focus on protecting the interests of the industry”, the campaigners said. (Ends 2018h) |
| Ciel | 20-2-2019 | David Azoulay - Centre for International Environmental Law (Ciel); Priscilla Villa – Earthworks; Yvette Arellano - Texas Environmental Justice Advocacy Services (Tejas); Miriam Gordon - UPSTREAM; Doun Moon - Global Alliance for Incinerator Alternatives (GAIA); and Kathryn Miller and Kristen Thompson - Exeter University, publish the report “Plastic and Health: The hidden cost of a plastic planet,” in Feb. 2019. The report conclude that the "current narrow approaches to assessing and addressing plastic impacts" as "inadequate and inappropriate" and that plastic should be recognized as a POP (Stringer, 2019). |
| Beat the Microbead | 11-12-2018 | The NGO “Beat the Microbead” has called for industry action to go beyond synthetic, solid particles used for exfoliating and cleansing (Oziel, 2018). |
| European Environmental Bureau, ClientEarth, ChemSec, and Greenpeace | 11-6-2018 | In June, several NGOs criticised Echa for:  "[Unduly limiting](https://chemicalwatch-com.proxy.findit.dtu.dk/67582)" the scope of the proposal by having taken into account industry concerns before putting the proposal before its scientific committees (Oziel, 2018).  “*The "long-overdue" restriction proposal, the NGOs said, will exclude "relevant and harmful uses of microplastics" before the Committees for Risk Assessment and Socio-economic Analysis (Rac and Seac) give their opinion*” (Oziel, 2018).  ECHA "did not show interest" in receiving information from non-industry stakeholders about the hazards and risks of microplastics (Oziel, 2018).  ECHA lacked the objectivity required by its role in the restriction process and misinterpreted the conditions that may justify the addition of derogations to the restriction (Oziel, 2018).  “*ClientEarth lawyer Alice Bernard said Echa's priority should be to ensure no risks are overlooked or underestimated. "Yet we witnessed a call for evidence with an excessive focus on protecting the interests of the businesses responsible for this microplastic pollution."*” (Oziel, 2018). |
|  | 2-11-2017 | A group of NGOs is putting pressure on the European Commission to introduce an EU ban on all microplastics in cosmetics and to take regulatory action on polymer ingredients. (Zainzinger, 2017).  The Beat the Microbead coalition says in a position paper that the plastic pollution of waters by the cosmetics industry will not stop unless the Commission sets clear rules. (Zainzinger, 2017).  New EU regulation should ban all polymer ingredients that are persistent, bioaccumulative or toxic to ecosystems from being used in cosmetic products, the NGOs demand. (Zainzinger, 2017).  It follows a call for action on microplastics by NGO umbrella group Rethink Plastics. Last month, the group [urged](https://chemicalwatch.com/60212/ngos-urge-tighter-regulations-for-microplastics) the Commission to implement immediate legislative measures to reduce microplastic pollution at the source. (Zainzinger, 2017). |
| Rethink Plastics | 19-10-2017 | NGO umbrella group Rethink Plastics has called on the European Commission to implement immediate legislative measures to reduce microplastic pollution at the source. (CW, 2017c) |
| ChemSec & Beuc | 24-8-2017 | ChemSec and European consumer organisation Beuc are urging the European Commission to implement bans on several substances used in detergents. (Buxton, 2017). |
| Greenpeace | 21-2-2019  18-7-2018  11-6-2018  26-7-2016 | Represented by Melissa Wang, discusses the latest trends in chemical management science and highlights the issue of plastic pollution (CW, 2019m).  A study by Greenpeace, June 21 2018, in the Antarctic, has revealed the presence of microplastics and per- and polyfluorinated alkylated substances (PFASs) in most of the seawater and snow (CE, 2018l).  Greenpeace warned about the industry's use of large quantities of polyester and its contribution to pollution of the oceans with microplastic fibres (CW, 2018p).  *"There's no single bad player; the industry as a whole is failing to regulate the use of microplastics [in] everyday products,"* says Taehyun Park, oceans campaigner at Greenpeace East Asia. *"The only sure and efficient way to successfully make sure that microplastics [are removed] from consumer products is to ban them."* (CW, 2016d). |

**References**

Baumüller, A., 2018, Healthy rivers and lakes are not ‘nice-to-haves’, they are essential to our existence, EURACTIV, Link: <https://www.euractiv.com/section/energy-environment/opinion/healthy-rivers-and-lakes-are-not-nice-to-haves-they-are-essential-to-our-existence/> - accessed 28-10-2019

Buxton, L., 2017, NGOs urge EU ban on substances in detergents, ChemicalWatch, Link: <https://chemicalwatch.com/58350/ngos-urge-eu-ban-on-substances-in-detergents?q=microplastic> – accessed 20-8-2019.

ChemicalWatch (CW), 2017c, NGOs urge tighter regulations for microplastics, Link: <https://chemicalwatch.com/60212/ngos-urge-tighter-regulations-for-microplastics?q=microplastic> – accessed 20-8-2019.

ChemicalWatch (CW), 2018i, Norway's EPA proposes artificial turf microplastics pollution rules, Link: https://chemicalwatch-com.proxy.findit.dtu.dk/68742/norways-epa-proposes-artificial-turf-microplastics-pollution-rules?q=microPlastics - accessed 14-8-2019.

ChemicalWatch (CW), 2019m, NGO Platform: Trends in chemical management science and governance, Link: <https://chemicalwatch.com/74531/ngo-platform-trends-in-chemical-management-science-and-governance?q=microPlastics> - accessed 11-06-2019.

Cousteau, P., 2017, Plastics: The invisible oil spill, EURACTIV, Link: <https://www.euractiv.com/section/circular-economy/opinion/plastics-the-invisible-oil-spill/> - accessed 28-10-2019

ECHA, 2019, General Comments and answers to specific information requests, Helsinki: European Chemicals Agency, Link: [https://echa.europa.eu/registry-of-restriction-intentions/-/dislist/details/0b0236e18244cd73 - accessed 28-10-2019](https://echa.europa.eu/registry-of-restriction-intentions/-/dislist/details/0b0236e18244cd73%20-%20accessed%2028-10-2019)

Ends, 2018g, ECHA defends ‘objectivity’ of microplastics call, Link: [https://www.endseurope.com/article/52975/echa-defends-objectivity-of-microplastics-call - accessed 22-10-2019](https://www.endseurope.com/article/52975/echa-defends-objectivity-of-microplastics-call%20-%20accessed%2022-10-2019).

Ends, 2018h, ECHA lacked objectivity, say microplastics campaigners, Link: <https://www.endseurope.com/article/52922/echa-lacked-objectivity-say-microplastics-campaigners> - accessed 22-10-2019

Franklin, K., 2016, Greenpeace slams cosmetic industry microbeads commitments, ChemicalWatch, Link: <https://chemicalwatch.com/48785/greenpeace-slams-cosmetic-industry-microbeads-commitments?q=microplastic> – accessed 20-8-2019.

Keating, D., 2018, A plan for plastics, EURACTIV, Link: <https://www.euractiv.com/section/circular-economy/news/a-plan-for-plastics/> - accessed 28-10-2019

Lovell, T., 2018, NGO report urges fashion brands to join collaborative initiatives, ChemicalWatch, Link: https://chemicalwatch-com.proxy.findit.dtu.dk/67541/ngo-report-urges-fashion-brands-to-join-collaborative-initiatives?q=microPlastics - accessed 14-8-2019.

Oziel, C., 2018, Denmark to impose temporary ban on microplastics in cosmetics, ChemicalWatch, Link: https://chemicalwatch-com.proxy.findit.dtu.dk/72707/denmark-to-impose-temporary-ban-on-microplastics-in-cosmetics?q=microPlastics - accessed 14-8-2019.

Oziel, O., 2018, NGOs attack Echa’s ‘limited’ microplastics restriction proposal, ChemicalWatch, Link: https://chemicalwatch-com.proxy.findit.dtu.dk/67582/ngos-attack-echas-limited-microplastics-restriction-proposal?q=microPlastics - accessed 14-8-2019.

Rodrigo, A. & Powell, D., 2018, Can we tax our way out of the plastic pollution crisis, EURACTIV, Link: <https://www.euractiv.com/section/circular-economy/opinion/can-we-tax-our-way-out-of-the-plastic-pollution-crisis/> - accessed 28-10-2019

Stringer, L., 2019, Plastics exposure a global health crisis, says NGO report, ChemicalWatch, Link: https://chemicalwatch.com/74500/plastics-exposure-a-global-health-crisis-says-ngo-report?q=microPlastics - accessed 12-6-2019.

Zainzinger, V., 2017, NGO coalition urges EU-wide ban on microplastics in cosmetics, ChemicalWatch, Link: <https://chemicalwatch.com/60737/ngo-coalition-urges-eu-wide-ban-on-microplastics-in-cosmetics?q=microplastic>, accessed 20-8-2019.
