# Supplementary material for: Stakeholder analysis with regard to a recent European restriction proposal on microplastics
Source: PLoS One. 2020 Jun 22;15(6):e0235062. doi: 10.1371/journal.pone.0235062 (PMC7307934; doi:10.1371/journal.pone.0235062)
Supplement: S11 Table — (DOCX) [file pone.0235062.s012.docx]

S11 Table: National Authorities microplastics comments

| **Stakeholder** | **Date** | **Expressed interests/opinion on microplastics at CW, Ends, EURACTIV, EUObserver** |
| --- | --- | --- |
| Mary Creagh (British MP) | 24-8-2016 | Mary Creagh, chair of the environmental audit committee: *“We need a full, legal ban, preferably at an international level as pollution does not respect borders,” she added. “If this isn’t possible after our vote to leave the EU, then the government should introduce a national ban. The best way to reduce this pollution is to prevent plastic being flushed into the sea in the first place”* (Vidal, 2016). |
| Bettina Hoffman (German Green Party) | 23-10-2018 | German Green party environment spokesperson Bettina Hoffmann called on the government to act in line with the precautionary principle and “to stop the entry of microplastics in our environment”, including banning them from cosmetics and from entering compost and digestate from biogas plants. (German green party) (Ends 2018b) |
| BE government | 4-10-2017 | Belgium has notified the European Commission of a draft plan to voluntarily phase out microplastics in all consumer products by 2019. (CW, 2017d).  The deliberate addition of microplastics into disposable consumer products must be "significantly reduced", the notification says. To this end, parties will be obliged to follow the scientific and technological evolutions in this area and to "take the necessary measures in case of new proven problems". (CW, 2017d) |
| DK EPA | 5-2-2019  30-4-2018  16-1-2018  19-11-2016 | “*The 30 January collaboration, formed to reduce plastic pollution and promote a circular plastic economy, comes after the government released an action plan on reducing plastic waste in December*” 2018 (Buxton, 2019).  Microplastics are unlikely to present a risk of contamination to groundwater (CW, 2018u)  “*Late last year, Denmark also said it will*[*impose*](https://chemicalwatch.com/72707/)*a temporary ban on microplastics added to rinse-off cosmetics from 1 January 2020 at the latest. This will stay in place until an EU-wide measure comes into play. It is also considering extending the ban to all cosmetics products within three years*” (Buxton, 2019)  The Partnership for Microplastics report, released by the Danish EPA, highlights how little is known about the effects of microplastics on the environment. (CW, 2018zd)  Car tyres, paint, shoes, textiles and other secondary sources are responsible for almost all microplasticpollution in Denmark, according to a study by the country's EPA. (CW, 2015a).  The agency found that secondary microplastics – abrasions of larger plastic particles that are broken down as they make their way to the aquatic environment – make up 99% of the total amount emitted to the aquatic environment. (CW, 2015a). |
| DK Government | 11-12-2018  3-8-2018  11-12-2017  11-5-2016  21-1-2016 | Minister for Environment and Food Jakob Ellemann-Jensen proclaims that Denmark is to introduce a temporary ban on microplastics for rinse-off cosmetics until an EU-wide measure is implemented. The government also consider a MP ban for all cosmetics within three years (CW, 2018a).  Urged EC to plug knowledge gaps on microplastics in the EU plastics strategy (Zainzinger, 2018).  The Danish government urged the European Commission to help plug the gaps around knowledge of microplastics as it prepares to publish its EU plastics [strategy](https://chemicalwatch.com/52436/). (CW, 2017a).  Denmark’s new environment and food minister, Esben Lunde Larsen, is urging the European Commission to introduce a Europe-wide ban on the use of microplastics in cosmetic products. (Buxton, 2016).  “*We have to limit the spread of microplastics so that it does not end up in the aquatic environment and in the food chain,*” Mr Larsen said in a release. (Buxton, 2016).  “*A ban on microplastics in cosmetics should be considered as a first step in solving the issue and further actions have to be considered,*” Elisabeth Paludan, deputy head of chemicals at the MoE, told Chemical Watch. (Buxton, 2016).  Eva Kjer, the Danish environment and food minister, has said that the spread of microplastic must be curtailed, so that it does not end up in the aquatic environment and food chain. (CW, 2016j). |
| FR government | 20-09-2018  18-10-2016 | Banned rinse-off cosmetic products containing microplastics, January 2018 (CW, 2018e).  France has notified the European Commission it will ban from the market rinse-off cosmetic products containing microplastics by 1 January 2018. (CW, 2016a). |
| German UPA | 7-12-2017 | German Environment Agency (UBA), “UBA believes that voluntary commitments by the cosmetic industry are no longer sufficient and will call on the European Commission to consider a full ban. Since 2013, a voluntary agreement has been in place with the German industry. But UBA expert Marcus Gast claimed that this deal only covers a small number of microplastics, such as those found in toothpaste” (Morgan, 2017) |
| GER EPA (UBA) | 18-4-2016 | The report, MikroPlastik: Development of an environmental assessment concept, says the effects of microplastics have not been fully analysed. (CW, 2016h). |
| I government | 8-6-2018 | Italy has notified the European Commission of plans to ban the production and marketing of rinse-off cosmetics products containing microplastics (Stringer, 2018). |
| NO EPA | 2-7-2019  19-2-2019  18-7-2018  24-4-201  5-1-2017 | The Norwegian EPA has opened a consultation on a proposal to prevent the spread of microplastics from artificial turf pitches that use rubber granules (CW, 2019h).  The Norwegian EPA states “*Microplastics have been found in 35 samples of sediment and bottom-living organisms collected from the seabed of the North Sea and the Barents Sea*” and “*among the most common findings in the sediments analysed were paint, synthetic rubber, chlorinated polyethylene, polyacrylamide and polyethylene terephthalate*” (CW, 2019p).  Proposed measures to reduce the spread of microplastics from artificial turf in sports facilities into the environment (CW, 2018i).  The Norwegian Environment Agency is looking at treatment solutions for capturing microplastics from roads in congested areas to prevent them from entering the wastewater system. (CW, 2018v)  The move forms part of a [strategy](https://chemicalwatch.com/54110/) set out by Norway's climate and environment minister Vidar Helgesen last year to investigate ways of reducing marine litter and microplastic in the ocean. (CW, 2018v)  The Norwegian Environment Agency has investigated possible measures to reduce the spread of microplastics into the environment. (CW, 2017t). |
| NO government | 2-7-2019  20-2-2018  9-3-2017 | *“According to the Norwegian environment minister Ola Elvestuen, turf pitches containing them are one of the largest sources of microplastics spreading in Norway”* (CW, 2019h).  Norway's climate and environment minister has asked for proposals for rules to be drawn up to reduce the spread of microplastic from artificial turf used in sports facilities. (CW, 2018z)  Norway's climate and environment minister has called for an investigation into ways of tackling marine litter and microplastic in the ocean. (CW, 2017p). |
| **Norway** | 25-01-2019 | Norway's Special Representative for the Oceans and former minister for EU affairs, Vidar Helgesen: "The EU's plastic strategy and its circular economy package is very important. Norway is part of the single market and we welcome common European approaches. The EU-ban on single-use plastics will have a tremendous effect, because the EU is such a big market,"; "I am not sure that in 10 years we will have less plastics in the oceans than we have today, but the flow of plastics into the oceans will be smaller.” And "The attention to the issue among the electorate and at the highest political level is a good sign, but sadly we will see for a number of years increased plastic in the oceans before new measures will take effect. You have a number of countries particularly in the Asia-Pacific where it is critically important to get waste management systems into place and where it will take time," (Breum 2019) |
| RIVM | 11-7-2016 | Tyres, paints and abrasive cleaning agents can release microplastic particles that end up in soil, water and air, according to a report commissioned by the Dutch environment ministry. (CW, 2016e).  RIVM says it is essential to create awareness of the problem among consumers and professionals. It argues the release of microplastics can be reduced through innovation and by implementing measures preventing the distribution of particles into the environment. (CW, 2016e). |
| SE government | 14-3-2019  6-2-2018  22-1-2018  12-7-2017  30-9-2016 | The deputy prime minister of Sweden, Isabella Lövin, stresses: “The government will push the international community to agree strong measures to tackle the adverse impacts of chemicals and plastics” (Stringer, 2019).  A Swedish ban on rinse-off cosmetics containing microbeads will enter into force at the beginning of July, the country's environment ministry has said. (CW, 2018za)  Sweden is considering extending its proposed ban on microbeads in rinse-off cosmetics to all products that release microplastics. (CW, 2018zb)  Sweden has proposed a ban on rinse-off cosmetics that contain microplastics to reduce their impact on the marine environment. (CW, 2017g).  Sweden said that with today's technology it is impossible to capture and collect plastic particles once they are released into the environment. "*It is therefore necessary to take measures at the source*," the notification said, adding "*there are no less restrictive measures*" which would achieve the same purpose. (CW, 2017g).  The Swedish government is planning national legislation to prevent microplastics getting into the environment, and emission control limits for construction products, (Lovell, 2016) |
| SE Chemicals Agency | 4-7-2018  29-3-2018  21-1-2016 | Warn that artificial turf contributes to an increased presence of both microplastics and hazardous substances (2018m).  A Swedish investigation into whether further national restrictions on microplastics in cosmetics and other chemical products are needed concluded that such action would be better carried out at EU level in the first instance. (CW, 2018y)  Sweden's chemical agency Kemi, which carried out the research, says "the work being done at EU level on restriction proposals could result in reliable decision material and clear and harmonised rules and regulations which would also be cost-effective". (CW, 2018y)  The agency says other measures are needed to reduce the amount of microplastics in oceans and lakes. (CW, 2016j). |
| UBA (Germany) | 18-4-2016 | The report, MikroPlastik: Development of an environmental assessment concept, says the effects of microplastics have not been fully analysed. (CW, 2016h). |
| UK government | 10-5-2018  9-1-2018  25-7-2017  25-8-2016  14-4-2016 | No intention to ban wet wipes (CW, 2018s)  A ban on the manufacture of cosmetics and personal care products containing plastic microbeads comes into effect in the UK today. (CW, 2018ze)  The UK's new environment minister has repeated the government's pledge to introduce legislation to ban the manufacture of rinse-off cosmetics containing microbeads from 1 January 2018, and their sale from 30 June the same year. (Zainzinger, 2017).  The environment ministry, Defra, has promised to review evidence on solid plastic particles in products outside of the scope of the ban, together with the Hazardous Substances Advisory Committee (HSAC), and to "assess the potential for further actions". (Zainzinger, 2017).  A UK cross-party committee of MPs has called for a ban on plastic microbeads in cosmetics. (CW, 2016c).  The UK House of Commons’ Environmental Audit Committee has launched an inquiry into the environmental impact of microplastics. (CW, 2016i). |
| National Authorities | 13 Jul 2018  15 Jun 201  27 Mar 2018  7 Mar 2016  30 Sep 2013  11 Jun 2013 | “One of the proposals in the regulation is the requirement for a physical barrier around the course. The measure is effective, but it can be expensive to implement,” said Ellen Hambro, director of Norway’s Environment Agency.  The EA say that 10% of plastic infill is lost each year. "Many sports clubs are already making an effort to prevent micro-plastic discharge, but more needs to be done. It is possible to reduce emissions by up to 98% from such courses,” Hambro explains. (Ends 2018e)  Tyres are responsible for the unintentional release of microplastics in the marine environment, which are covered by the plastics strategy currently being discussed at EU level. (Italien environmental minister) (Ends 2018f)  KEMI recommended that manufacturing companies voluntarily restrict the use of microplastics in deodorants, eye creams, hair gels and hair mousses, body creams and face creams, all kinds of makeup – mascara, eyeliner, rouge, powder and lipstick – anti-ageing skin products, tanning products and sunscreens, and bleaching creams. (Swedish KEMI) (Ends 2018i)  Austria, Sweden and France called for policies to tackle marine litter, with Austria and Sweden both calling for action to address the problem of microplastics in [cosmetics](http://www.endseurope.com/article/45315/). (Ends 2016a)  Microplastics in products is an area of particular concern. In the Council of Ministers, [the Netherlands](http://www.endseurope.com/32144) is pushing for a ban on microplastics in personal care products to reduce the threat they pose to the marine environment. (Ends 2013a)  The Dutch document is a response to the European Commission’s [green paper](http://www.endseurope.com/30930) on plastic waste, which identified microplastics as a “particular concern”. They derive mostly from the degradation of plastic litter in the sea. A number of scientific studies have shown they can harm plankton and other marine species. (Ends 2013c) |
| Sweden, MemberState, The Swedish Medical Products Agency | 2019/05/20  **Content:**  Scope or restriction option analysis;  Baseline;  Other socio economic analysis (SEA) issues | **Comment:**  The Swedish Medical Products Agency wants to emphasize the importance that the restriction report includes a clear conformation for competent authorities to perform in market surveillance of microplastic in cosmetic products. We would also like to express concerns regarding inadequate in market surveillance if the legal requirements are unclear, for e.g. in the case of unclear description of what and when something is a microplastic at different stages (from production to the final product).  **Answer to specific info request 3:**  In medical devices, microplastics can be intentionally added with properties essential for the function of the device. The amount can be far above the proposed concentration limit of 0.01%, as described in Annex XV restriction report, proposal for a restriction, version 1.1., March 20, 2019.  **Answer to specific info request 4:**  The Swedish Medical Products Agency supports initiatives that are taken to meet the global climate goals. The Swedish Medical Products Agency also supports a derogation for medical devices and in vitro diagnostic medical devices.  However, The Swedish Medical Products Agency believes that the consequences of the proposal on Medical Devices and In vitro diagnostic medical devices need to be further explored, especially related to consequences on patient safety, socio-economic impacts and time for implementation. The proposal can lead to shortage of products and put patients at risk.  The socio-economic impact presented by the Dossier Submitter appears to be based on a limited number of products used in professional settings. Despite lack of access to a complete list of products containing microplastic, the summary of products and their uses appear to be incomplete.  The Swedish Medical Products Agency has noticed that CE-labelled absorbance products such as incontinence products are not mentioned in the documentation on the proposed restriction. Such absorbance products can contain superabsorbent microplastics. This product category is subsidized by the Swedish government and are not only used by professionals under controlled conditions. These products are high volume products, both used by professionals and other users, at both hospitals and in the homes of patients. Today, waste from such products are typically handled as household waste. In Sweden the majority of household wastes are incinerated. Still, requirements on incineration or deposition as hazardous waste management treatment of such waste can have impact on the health care system, including patient safety.  The Dossier Submitter suggests that the use and release shall be monitored and reported, and that compliance can be monitored at member state level by reviewing PSUR.  According to article 86 in the medical device regulation (2017/745), “manufacturers of class IIa, class IIb and class III devices shall prepare a periodic safety update report (‘PSUR’). Manufacturers of class IIb and class III devices shall update the PSUR at least annually and class IIa devices shall update the PSUR when necessary and at least every two years. For class III devices or implantable devices, manufacturers shall submit PSURs by means of the electronic system. For class I, IIa and IIb devices, manufacturers shall make PSURs available to the notified body involved in the conformity assessment and, upon request, to competent authorities.” This will increase the burden on notified bodies and competent authorities. The be noticed, an electronic system is still not in place and the majority of the PSURs are not going to be actively submitted to this system and would have to be requested by the competent authorities. Also, the environmental aspects of devices are not covered by regulation 2017/745, in particular the provisions on PSURs, and this would be an additional demand put on the manufacturers based on other legislation. Hence, the feasibility to monitor and report requires further attention.  The Dossier Submitter suggests that medical devices and in vitro diagnostic products can adapt to the regulation within 2 years, i.e. implement technical means where microplastics would be contained throughout their use and incinerated at the end of their life-cycle and update labels, SDS, IFU to provide enough instructions to prevent release to the environment. Even such seemingly non-intrusive modifications may entail scrutiny by notified bodies, for the devices that require a certificate. The Swedish Medical Products Agency believe that this is probably a too short implementation period and suggest that the implementation time for updates of information should be decided when all processes for MDR and IVDR are in place. Competent authorities, notified bodies and companies are working hard to adapt to the new requirements. As of today, all processes are not yet in place. Additionally, microplastics are most often added to the products with purpose to provide unique functions. Such products can be advanced. A transition time of 2 years to replace such product with non-microplastic solutions is for most products more than a challenge, even modifying product to contain microplastics throughout their use can be challenging. Research and development achievements are probably required. Such technical means most likely require more than 2 years implementation time.  The Swedish Medical Products Agency highly recommend that the consequences of the proposal for Medical Devices and In vitro diagnostic products are further evaluated to avoid shortage of products and that patient’s safety are compromised.  **Answer to specific info request 5:**  Regulation (EC) No 1223/2009 on cosmetic products states in article 3 that “a cosmetic product made available on the market shall be safe for human health when used under normal or reasonably foreseeable conditions of use”. The safety of a cosmetic product is demonstrated by ensuring that a cosmetic product has undergone a safety assessment (which, among other things, must take systemic -and local toxicity into account).  The Swedish Medical Products Agency wants to highlight concerns regarding the risk to human health if the transitional periods for alternative ingredients to microplastics are too short (for other rinse-off and leave on cosmetic products). Also, the risk to human health if alternatives to microplastics are introduced too quickly.  Animal testing is strictly prohibited for finished cosmetic products and ingredients exclusively used in cosmetics or specifically carried out in the context of the EU cosmetic regulation. Since alternative methods for systemic toxicity is at current date not available, implications for the development of new ingredients are at place. Abundant data from tests of good quality are needed for the safety assessment, to ensure that a cosmetic product is safe for human health under normal condition of use.  However, for alternatives to microplastic ingredients that are already available on the market (and already assessed as safe), a transitional period of 4-6 years could be appropriate. |

**References**

Breum, M. 2019. Plastic pollution increasing at the top of the Earth. EUObserver. Link: <https://euobserver.com/nordic/144003>

Buxton, L, 2016, Denmark calls for EU ban on microplastics in cosmetics, ChemicalWatch, Link: <https://chemicalwatch.com/47321/denmark-calls-for-eu-ban-on-microplastics-in-cosmetics?q=microplastic> – accessed 20-8-2019.

Buxton, L., 2019, Cross-party agreement bolsters Danish microplastic efforts, ChemicalWatch, Link: https://chemicalwatch.com/74047/cross-party-agreement-bolsters-danish-microplastic-efforts?q=microPlastics - accessed 12-6-2019.

ChemicalWatch (CW), 2015a, Denmark identifies main sources of microplastic pollution, Link: <https://chemicalwatch.com/43667/denmark-identifies-main-sources-of-microplastic-pollution?q=microplastic> – accessed 20-8-2019.

ChemicalWatch (CW), 2016a, France to ban microplastics in some cosmetics products, Link: <https://chemicalwatch.com/50368/france-to-ban-microplastics-in-some-cosmetics-products?q=microplastic> – accessed 20-8-2019.

ChemicalWatch (CW), 2016c, UK MPs call for ban on microbeads in cosmetics, Link: <https://chemicalwatch.com/49263/uk-mps-call-for-ban-on-microbeads-in-cosmetics?q=microplastic> – accessed 20-8-2019.

ChemicalWatch (CW), 2016e, RIVM tracks microplastics from tyres, paints and cleaning agents, Link: <https://chemicalwatch.com/48495/rivm-tracks-microplastics-from-tyres-paints-and-cleaning-agents?q=microplastic> – accessed 20-8-2019.

ChemicalWatch (CW), 2016h, Germany’s UBA published microplastics report, Link: <https://chemicalwatch.com/46653/germanys-uba-publishes-microplastics-report?q=microplastic> – accessed 20-8-2016.

ChemicalWatch (CW), 2016i, UK parliamentary committee launches microbeads inquiry, Link: <https://chemicalwatch.com/46591/uk-parliamentary-committee-launches-microbeads-inquiry?q=microplastic> – accessed 20-8-2019.

ChemicalWatch (CW), 2016j, Sweden and Denmark move on microbeads, Link: <https://chemicalwatch.com/44608/sweden-and-denmark-move-on-microbeads?q=microplastic> – accessed 20-8-2019.

ChemicalWatch (CW), 2017a, Help close microplastic knowledge caps, Denmark tells Commission, Link: <https://chemicalwatch.com/62446/help-close-microplastic-knowledge-gaps-denmark-tells-commission?q=microplastic> – accessed 20-8-2019.

ChemicalWatch (CW), 2017d, Belgium mulls ‘total ban’ on microplastics in consumer products, Link: <https://chemicalwatch.com/59707/belgium-mulls-total-ban-on-microplastics-in-consumer-products?q=microplastic> – accessed 20-8-2019.

ChemicalWatch (CW), 2017g, Sweden proposes ban on microbeads in rinse-off cosmetics, Link: <https://chemicalwatch.com/57599/sweden-proposes-ban-on-microbeads-in-rinse-off-cosmetics?q=microplastic> – accessed 20-8-2019.

ChemicalWatch (CW), 2017p, Norwegian environment minister launches microplastics investigation, Link: <https://chemicalwatch.com/54110/norwegian-environment-minister-launches-microplastics-investigation?q=microplastic> – accessed 20-8-2019.

ChemicalWatch (CW), 2017t, Norway looks at reducing microplastic pollution, Link: <https://chemicalwatch.com/51964/norway-looks-at-reducing-microplastic-pollution?q=microplastic> – accessed 20-8-2019.

ChemicalWatch (CW), 2018i, Norway's EPA proposes artificial turf microplastics pollution rules, Link: https://chemicalwatch-com.proxy.findit.dtu.dk/68742/norways-epa-proposes-artificial-turf-microplastics-pollution-rules?q=microPlastics - accessed 14-8-2019.

ChemicalWatch (CW), 2018m, Swedish chemicals agency warns about hazardous substances in artificial turf, Link: https://chemicalwatch-com.proxy.findit.dtu.dk/68235/swedish-chemicals-agency-warns-about-hazardous-substances-in-artificial-turf?q=microPlastics - accessed 14-8-2019.

ChemicalWatch (CW), 2018e, France ban rinse-off cosmetic products containing microplastics, Link: https://chemicalwatch-com.proxy.findit.dtu.dk/70462/french-assembly-votes-to-ban-plastic-fcms-in-school-canteens?q=microPlastics - accessed 14-8-2019.

ChemicalWatch (CW), 2018s, UK government denies ban on wet wipes, Link: https://chemicalwatch-com.proxy.findit.dtu.dk/66792/uk-government-denies-ban-on-wet-wipes?q=microPlastics - accessed 14-8-2019.

ChemicalWatch (CW), 2018u, Microplastics 'low risk' for groundwater pollution – Danish study, Link: <https://chemicalwatch-com.proxy.findit.dtu.dk/66444/microplastics-low-risk-for-groundwater-pollution-danish-study?q=microPlastics> - accessed 14-8-2019.

ChemicalWatch (CW), 2018v, Norway investigating solutions for tackling road dust microplastics, Link: <https://chemicalwatch.com/66144/norway-investigating-solutions-for-tackling-road-dust-microplastics?q=microplastic> – accessed 20-8-2019.

ChemicalWacth (CW), 2018y, Sweden advocates developing microplastic restrictions at EU level, Link: <https://chemicalwatch.com/65507/sweden-advocates-developing-microplastic-restrictions-at-eu-level?q=microplastic> – accessed 20-8-2019.

ChemicalWatch (CW), 2018z, Norway planning rules on emissions from artificial turf microplastics, Link: <https://chemicalwatch.com/64144/norway-planning-rules-on-emissions-from-artificial-turf-microplastics?q=microplastic> – accessed 20-8-2019.

ChemicalWatch (CW), 2018zb, Sweden considering wider restrictions on microplastics, Link: <https://chemicalwatch.com/63246/sweden-considering-wider-restrictions-on-microplastics?q=microplastic> – accessed 20-8-2019.

ChemicalWatch (CW), 2018zd, Danish study finds understanding of microplastics ‘extremely defectice’, Link: <https://chemicalwatch.com/63130/danish-study-finds-understanding-of-microplastics-extremely-defective?q=microplastic> – accessed 16-1-2018.

ChemicalWatch (CW), 2019h, Norway consults on proposal to curb artificial turf microplastics emissions, Link: https://chemicalwatch.com/79366/norway-consults-on-proposal-to-curb-artificial-turf-microplastics-emissions?q=microPlastics - accessed 11-6-2019.

ChemicalWatch (CW), 2019p, Norway finds microplastics at bottom of North Sea, Link: https://chemicalwatch.com/74427/norway-finds-microplastics-at-bottom-of-north-sea?q=microPlastics - accessed 12-6-2019.

ECHA, 2019, General Comments and answers to specific information requests, Helsinki: European Chemicals Agency, Link: [https://echa.europa.eu/registry-of-restriction-intentions/-/dislist/details/0b0236e18244cd73 - accessed 28-10-2019](https://echa.europa.eu/registry-of-restriction-intentions/-/dislist/details/0b0236e18244cd73%20-%20accessed%2028-10-2019).

Ends, 2013a, Stakeholders back EU landfill ban for plastics, Link: <https://www.endseurope.com/article/33242/stakeholders-back-eu-landfill-ban-for-plastics> - accessed 23-10-2019

Ends, 2013c, Ban microplastics in cosmetics, says the Netherlands, Link: <https://www.endseurope.com/article/32144/ban-microplastics-in-cosmetics-says-the-netherlands> - accessed 23-10-2019

Ends, 2016a, Strong support for circular economy eco-design at Council, Link: <https://www.endseurope.com/article/45350/strong-support-for-circular-economy-ecodesign-at-council> - accessed 23-10-2019

Ends, 2018b, Evidence of microplastics contamination in human gut, Link: <https://www.endseurope.com/article/54056/evidence-of-microplastic-contamination-in-human-gut> - accessed 23-10-2019

Ends, 2018e, Norway to tackle discharge of microplastics from sports field, Link: <https://www.endseurope.com/article/53317/norway-to-tackle-discharge-of-microplastic-from-sports-fields> - accessed 23-10-2019

Ends, 2018f, Italian environment minister calls for labelling of tyres to boost recycling, Link: <https://www.endseurope.com/article/53032/italian-environment-minister-calls-for-labelling-of-tyres-to-boost-recycling> - accessed 23-10-2019

Ends, 2018i, Sweden calls for further restrictions on microplastics, Link: <https://www.endseurope.com/article/52295/sweden-calls-for-further-restrictions-on-microplastics> - accessed 23-10-2019

Lovell, T., 2016, Sweden to legislate against microplastics, ChemicalWatch, Link: <https://chemicalwatch.com/49942/sweden-to-legislate-against-microplastics?q=microplastic> – accessed 20-8-2019.

Morgan, S., 2017, UN agrees on resolution to combat ‘planetary crisis’ of ocean plastics, EURACTIV, Link: <https://www.euractiv.com/section/energy-environment/news/un-agrees-on-resolution-to-combat-planetary-crisis-of-ocean-plastics/> - accessed 28-10-2019

Stringer, L., 2018 Italy to ban microplastics used in rinse-off cosmetics products, ChemicalWatch, Link: https://chemicalwatch-com.proxy.findit.dtu.dk/67533/italy-to-ban-microplastics-used-in-rinse-off-cosmetics-products?q=microPlastics - accessed 14-8-2019.

Stringer, L., 2019, Sweden deputy PM: stronger global action on chemicals needed, ChemicalWatch, Link: https://chemicalwatch.com/74998/sweden-deputy-pm-stronger-global-action-on-chemicals-needed?q=microPlastics - accessed 11-6-2019.

Vidal, J., 2016, Microplastics should be banned in cosmetics to save oceans, British MPs say, EURACTIV, Link: <https://www.euractiv.com/section/climate-environment/news/microplastics-should-be-banned-in-cosmetics-to-save-oceans-british-mps-say/> - accessed 28-10-2019

Zainzinger, V., 2017, Ban on microbreads in UK rinse-off cosmetics confirmed, ChemicalWatch, Link: <https://chemicalwatch.com/57857/ban-on-microbeads-in-uk-rinse-off-cosmetics-confirmed?q=microplastic> – accessed 20-8-2019.

Zainzinger, V., 2018, Bottled water study flags small-sized pigment particles, microplastics, ChemicalWatch, Link: https://chemicalwatch-com.proxy.findit.dtu.dk/69219/bottled-water-study-flags-small-sized-pigment-particles-microplastics?q=microPlastics - accessed 14-8-2019.
