# Supplementary material for: Stakeholder analysis with regard to a recent European restriction proposal on microplastics
Source: PLoS One. 2020 Jun 22;15(6):e0235062. doi: 10.1371/journal.pone.0235062 (PMC7307934; doi:10.1371/journal.pone.0235062)
Supplement: S12 Table — (DOCX) [file pone.0235062.s013.docx]

S12 Table: National NGOs microplastics comments

| **Stakeholder** | **Date** | **Expressed interests/opinion on microplastics at CW, Ends, EURACTIV, EUObserver** |
| --- | --- | --- |
| Ellen McArthur Foundation | 21-11-2017 | Jocelyn Blériot, Ellen MacArthur Foundation: “*Significant evidence indicates that oxo-degradable plastics do not simply break down and become innocuous, but instead fragment into tiny pieces. As such, they contribute to microplastic pollution, posing a risk to the ocean and other ecosystems, potentially for decades to come – whether we see it or not*” (Blériot, 2017). |
| Fidra, an environmental charity based in Scotland | 7. DEC 2017 | “Up to 195,000 tonnes of pre-production pellets – the small lentil-sized building blocks the plastic industry uses to make everything from bottles to car dashboards – leak into the oceans every year. It's one of the easier taps to turn off, according to Fidra, an environmental charity based in Scotland that is working on the issue at national and EU-level.  "To stop this source, all key players along the supply chain need to act, not just the proportion that have done so to date," said Sarah Archer, senior projects manager.” (Burrows 2017) |
| NABU | 8-10-2018 | Microplastics and poorly biodegradable polymer compounds are currently under-regulated because of gaps in the EU’s chemical and product regulations, according to a new study commissioned by German environmental Group NABU. NABU’s report calls on the European Commission to regulate and restrict through REACH polymer compounds that are slow to biodegrade. It adds that all microplastics, not just microbeads, should be banned from cosmetics, washing and cleaning products, since individual national bans cannot solve the problem alone. “I am worried that the European Commission or Parliament will not define exactly what they mean by microplastics,” she said, particularly given the relatively strong influence of the industry lobby in Brussels. (Ends 2018c) |
| UVW | 6-9-2013 | New challenges posed by microplastics were also discussed at the seminar. Greet De Gueldre of Belgian wastewater treatment firm Aquafin and Michaël Bentvelsen, speaking on behalf of Dutch association [UVW](http://www.uvw.nl/), made a case for restricting the use of these substances in personal care products.  In their joint presentation, they said NGOs had been effective at raising awareness of this issue in the Netherlands. Their actions led some retailers to ban sales of products with plastic ingredients. The country’s authorities are also planning to request a ban on these products at European level. (Ends 2013b) |

**References**

Blériot, J., 2017, Microplastics, macro problems, EURACTIV, Link: <https://www.euractiv.com/section/circular-economy/opinion/microplastics-macro-problems/> - accessed 22-10-2019

# Burrows, D., 2017, Microplastics threat poses dilemma for new EU strategy, EUobserver, Link: <https://euobserver.com/health/140194> - accessed 22-10-2019

Ends, 2018c, EU ‘failing’ to regulate plastic pollution, Link: <https://www.endseurope.com/article/53920/eu-failing-to-regulate-plastic-pollution> - accessed 22-10-2019

Ends, 2013b, World Water Week puts spotlight on chemical use, Ends, Link: <https://www.endseurope.com/article/32984/world-water-week-puts-spotlight-on-chemical-use> - accessed 22-10-2019
