# Supplementary material for: Stakeholder analysis with regard to a recent European restriction proposal on microplastics
Source: PLoS One. 2020 Jun 22;15(6):e0235062. doi: 10.1371/journal.pone.0235062 (PMC7307934; doi:10.1371/journal.pone.0235062)
Supplement: S13 Table — (DOCX) [file pone.0235062.s014.docx]

S13 Table: Other Contributors microplastics comments

| **Stakeholder** | **Date** | **Expressed interests/opinion on microplastics at CW, Ends, EURACTIV, EUObserver** |
| --- | --- | --- |
| Anthesis-Caleb (Consultant) | 7-1-2019 | Paul Ashford, Managing Director Anthesis-Caleb says the EU faces a conundrum as it assesses potential options involving plastic (Ashford, 2019).  …“*the discussion on what one is has moved centre-stage. Debate on the definition is still ongoing, but most of it has focused on physical form. Little attention has been paid to the chemical composition outside of biodegradability and source (natural v. synthetic), both of which are polymeric properties rather than identifiers*” (Ashford, 2019). |
| German Olympic Sports Confederation (DOSB) and the German Football Association (DFB) | 22-5-2019 | Called for a 6-years transition period before the proposed EU ban on intentionally added microplastics in synthetic turf is enforced (Oziel, 2019). |
| Iceland UK | 10-5-2018 | Ian Schofield says: ”some potential alternatives, such as bio-based otoxy-biodegradable polymers, including polyethylene furanoate (PEF) and polylactic acid (PLA). However, he said, as a packaging technologist, "I’m finding it extremely difficult to introduce some of these materials, because no one can prove to me that they don’t break down into microplastics." The main barrier, in his view, is that a proven marine environment test for materials, that shows how they break down in the ocean, is not yet available. "This is something we need fast. (Stringer, 2018)). |
| Italian SMEs | 13-6-2019 | *“Italian SMEs in the cosmetics industry have raised*[*concerns*](https://chemicalwatch.com/74140)*about the potential economic impact of Echa’s microplastics restriction*[*proposal*](https://chemicalwatch.com/73819)*on their business”* (Tani, 2019). |
| RAC | 2-4-2019 | Have until spring 2020 to comment on the restriction proposal for intentionally added microplastics by ECHA (CW, 2019g).  Confirm the upcoming restriction on intentionally-added microplastics in March 2019 (Davies, 2019). |

**References**

Ashford, P., 2019, Guest Column: Dealing with polymers under REACH, ChemicalWatch, Link: https://chemicalwatch.com/72674/guest-column-dealing-with-polymers-under-reach?q=microPlastics - accessed 8-8-2019.

ChemicalWatch (CW), 2019g, Echa begins consultation on microplastics restriction proposal, Link: https://chemicalwatch.com/75836/echa-begins-consultation-on-microplastics-restriction-proposal?q=microPlastics - accessed 11-6-2019.

Davies, E., 2019, Echa sets up working group for deluge of EDC authorisation applications, ChemicalWatch, Link: <https://chemicalwatch.com/75447/echa-sets-up-working-group-for-deluge-of-edc-authorisation-applications?q=microPlastics> - accessed 11-6-2019.

Oziel, C., 2019, Top German sports associations call for artificial turf ban transition, ChemicalWatch, Link: <https://chemicalwatch.com/77732/top-german-sports-associations-call-for-artificial-turf-ban-transition?q=microPlastics> - accessed 11-6-2019.

Stringer, L., 2018, Feature: HCF 2018 ponders plastic, ChemicalWatch, Link: https://chemicalwatch-com.proxy.findit.dtu.dk/66766/feature-hcf-2018-ponders-plastic?q=microPlastics - accessed 14-8-2019.

Tani, C., 2019, Italian cosmetics producer SMEs braced for proposed EU microplastics restriction., ChemicalWatch, Link: https://chemicalwatch.com/78730/italian-cosmetics-producer-smes-braced-for-proposed-eu-microplastics-restriction?q=microPlastics - accessed 11-6-2019.
