# Supplementary material for: Stakeholder analysis with regard to a recent European restriction proposal on microplastics
Source: PLoS One. 2020 Jun 22;15(6):e0235062. doi: 10.1371/journal.pone.0235062 (PMC7307934; doi:10.1371/journal.pone.0235062)
Supplement: S14 Table — (DOCX) [file pone.0235062.s015.docx]

S14 Table: Academia & Researchers microplastics comments

| **Stakeholder** | **Date** | **Expressed interests/opinion on microplastics at CW, Ends, EURACTIV, EUObserver** |
| --- | --- | --- |
| Academia | 24-4-2019  7-9-2017 | Deonie Allen, French National Centre for Scientific Research (CNRS): *“Microplastics are transported in the atmosphere and can settle in isolated high mountain regions, far from any major city or local pollution source. They are atmospheric pollutants”* (Senet, 2019).  Dr Anne Marie Mahon, Galway-Mayo Institute of Technology:  “We don’t know what the [health] impact is and for that reason we should follow the precautionary principle and put enough effort into it now, immediately, so we can find out what the real risks are” (EURACTIVu) |
| Research organizations | 15-5-2018  15-5-2018  20-7-2016 | Henning Wilts, Circular Economy Research Department at the Wuppertal Institute for Climate, Environment and Energy: *“If you want to have less plastic waste overall, the ban on straws or single-use tableware will only help a little”* (Menn & Schafer, 2018)  Georg Mehlhart, Öko-Institut: ”*questions the relevance of plastic cutlery in general. “When it comes to pollution of the oceans, there are much bigger problems,” he said, referring to microplastics and packaging.”* (Menn & Schafer, 2018)  Chris Sherrington, *Eunomia e*nvironmental Research and Consulting: ”To address this challenge, we need to do two things. We need to start thinking about ways that we can move away from single-use packaging in the first place and we need to continue building plastics into a more circular economy that, by putting a value on plastic items, encourages them to be returned rather than lost.” (Sherrington, 2016) |
| Academia | 23 Oct 2018 | “While the highest plastic concentrations in animal studies have been found in the gut, the smallest microplastic particles are capable of entering the bloodstream, lymphatic system and may even reach the liver,” Schwabl said. “Now that we have first evidence for microplastics inside humans, we need further research to understand what this means for human health.” (Medical University of Vienna and the Environment Agency Austria) (Ends 2018b) |
| **Scientists** | 7. DEC 2017 | ” "The problem is widescale and the concentrations are low," explained Richard Thompson, professor of marine biology at Plymouth University in the UK, "but if we carry on as normal and have this conversation again in 20 years' time we may well have reached concentration levels that are a concern."”  “Microplastics have also become a food safety concern.  "We have enough information to take a precautionary approach … and [implement] serious regulation," Sherri Mason, a professor of chemistry at the State University of New York at Fredonia told EUobserver. "There is enough information that this is having an impact and has the potential to have a very serious impact."  ”Writing about the topic for the [British Medical Journal](http://www.bmj.com/content/358/bmj.j4334) in September, Stephanie Wright from King's College London concluded there is an "urgent need for bigger, better and more definitive studies. We need to establish toxic characteristics of microplastics, their behaviour in the body, and what constitutes a safe threshold for exposure when plastics are ether ingested or inhaled."”  “Professor Tamara Galloway from the University of Exeter told EUobserver that "we don't yet know if this is at a level likely to cause harm, although most people would probably rather not be consuming plastics with their seafood".The biggest challenge is developing methods able to detect the presence of diverse polymer fragments in food or in human tissues. "We are not even yet at the point of being able to do that, let alone defining control groups," professor Galloway added. Finding a group that hasn't been exposed could be tricky.” (Burrows 2017) |
| King’s College London scientists | 29-6-2019 | Scientists from King's College London warn of the potential human health risks of microplastics from both dietary and inhalation exposure. (CW, 2017i).  "*Although microplastics are widely studied in the context of the marine environment where they are a prolific pollutant, we are only just recognising the potential human exposure pathways*," say Stephanie Wright and Frank Kelly. (CW, 2017i). |

**References**

# Burrows, D., 2017, Microplastics threat poses dilemma for new EU strategy, EUobserver, Link: <https://euobserver.com/health/140194> - accessed 22-10-2019

ChemicalWatch (CW), 2017i, King’s College scientists warn of potential health risks from microplastics, Link: <https://chemicalwatch.com/57290/kings-college-scientists-warn-of-potential-health-risks-from-microplastics?q=microplastic> – accessed 20-8-2019.

Ends, 2018b, Microplastics also travel through the air, says new study, Link: [https://www.endseurope.com/article/54056/evidence-of-microplastic-contamination-in-human-gut - accessed 22-10-2019](https://www.endseurope.com/article/54056/evidence-of-microplastic-contamination-in-human-gut%20-%20accessed%2022-10-2019)

Menn, A. & Schafer, K., 2018, Why the Commission’s new plastic ban threatens to fall flat, EURACTIV, Link: <https://www.euractiv.com/section/energy-environment/news/why-the-new-commission-plastic-ban-threatens-to-fall-flat/> - accessed 22-10-2019

Senet, S., 2019, Microplastics also travel through the air, says new study, EURACTIV, Link: <https://www.euractiv.com/section/energy-environment/news/microplastics-also-travel-through-airspace/> - accessed 22-10-2019

Sherrington, C., 2016, Clearing up the confusion around marine plastics, EURACTIV, link: <https://www.euractiv.com/section/climate-environment/opinion/clearing-up-the-confusion-around-marine-plastics/> - accessed 22-10-2019
