# Supplementary material for: Stakeholder analysis with regard to a recent European restriction proposal on microplastics
Source: PLoS One. 2020 Jun 22;15(6):e0235062. doi: 10.1371/journal.pone.0235062 (PMC7307934; doi:10.1371/journal.pone.0235062)
Supplement: S15 Table — (DOCX) [file pone.0235062.s016.docx]

S15 Table: Trade and Industry Associations microplastics comments

| **Stakeholder** | **Date** | **Expressed interests/opinion on microplastics at CW, Ends, EURACTIV, EUObserver** |
| --- | --- | --- |
| BASF (industry) | 1-2-2019 | BASF says the phase out of plastics and plastic additives is of "little concern", noting that the problem is already being addressed by focusing on the need to accelerate waste management processes (Stringer, 2019).  BASF says its customers are voluntarily introducing plastics based on recycled materials: "We see an increasing interest in recycling, such as demands for recycled material or support by making certain products recyclable" (Stringer, 2019). |
| CEFIC | 15-5-2019  22-8-2018  8-5-2019 | “*A proposal by Echa to restrict microplastics cannot be seen as appropriately meeting a legitimate objective under the REACH Regulation, according to analysis carried out by a leading international law firm*” (Buxton, 2019).2017o  The analysis was commissioned by CEFIC.  Criticizes the microplastics definition by ECHA for being "too broad" leaving "room for interpretation" making implementation and enforcement of restrictions "challenging" (Tani, 2019)  Two of seven Long-range Research Initiatives by CEFIC deal with microplastics. A total of €600,000 (CW, 2018f).  Speaking for the chemical industry trade association CEFIC, Blanca Serrano Ramón also expressed reservations about ECHA's proposal, arguing that the agency's definition of microplastics as a 'polymer containing particle' was too broad. |
| CTPA | 14-3-2017  11-1-2017 | The Cosmetic, Toiletry and Perfumery Association (CTPA) has said it could take longer to reformulate products, if the proposed UK ban on microbeads was expanded to include 'leave-on' products. (Lovell, 2017).  CTPA science director Emma Meredith told Chemical Watch that product re-development could take up to four years if raw materials became unavailable. “*We are unaware of any evidence to show that ingredients from leave-on cosmetic and personal care products have been found to pose a risk to the marine environment. Only rinse-off cleansing and exfoliating products have been associated with marine litter.*” (Lovel, 2017).  A UK cosmetics trade body has said personal care products should not be the main target of government policies, to prevent microplastics polluting the ocean. (Lovell, 2017).  Cosmetics, Toiletry and Perfumery Association (CTPA) director-general Chris Flower said: “If we are to stem the tide of microplastic marine litter, we must tackle the major sources and these are not cosmetic products.” (Lovel, 2017). |
| EurEau | 2-5-2018 | Klara Ramm, chair of the [EurEau](http://www.eureau.org/) Committee on Economics and Legal Affairs: “*One way of solving the underinvestment issues would be for the EU to fully implement the Polluter Pays Principle. This becomes even more vital as the technologies to remove emerging pollutants such as pharmaceuticals, microplastics or pesticides require substantial investments from the water sector, which is not the originator of the pollution*” (Ramm, 2018) |
| European Oilfield Speciality Chemicals Association | 11-6-2018 | Expressing [concern](https://chemicalwatch-com.proxy.findit.dtu.dk/65720/) about Echa’s digression from the Commission’s original definition of a microplastic to a "broader ‘catch-all’" one (Oziel, 2018).  The definition of microplastics and their "fate and partitioning" is crucial to the discussion about potential discharges and remedies (Oziel, 2018) |
| European offshore oil and gas industry | 25-5-2018 | Nik Robinson, secretary of the European Oilfield Speciality Chemicals Association (Eosca), told Chemical Watch that the EU study had relied on "poor and unreferenced reports" (Oziel, 2018). |
| EOSCA | 5-4-2018 | The European Oilfield Speciality Chemicals Association has warned its members, as well as non-members, that the consequences could be "heavy controls or even bans being placed on the marketing and use of products containing microplastics". (Buxton, 2018) |
| German Olympic Sports Confederation (DOSB) and the German Football Association (DFB) | 22-5-2019 | Called for a 6-years transition period before the proposed EU ban on intentionally added microplastics in synthetic turf is enforced (Oziel, 2019) |
| Mondi (industry) | 1-2-2019 | Graeme Smith, innovation and sustainability manager at Mondi: "*Whatever drop in polymer demand and packaging we see, will just be balanced out by other demands and other polymer markets"* and due to the barriers of recycling them, "*PVC and PVCD coatings, for example, are a thing of the past*" (Stringer, 2019). |
| The Oxo-biodegradable Plastic Association | 16-1-2016 | Michael Stephen, Chairman of the Oxo-biodegradable Plastic Association “The European Commissioners are either being very dim or don’t want to listen,” Stephens insisted, adding that Timmermans’ assertion that oxo-plastics break down into microplastics is “simply not true”” (Morgan, 2018) |

**References**

Buxton, L., 2018, Oil and gas industry faces microplastics scrunity, ChemicalWatch, Link: <https://chemicalwatch.com/65720/oil-and-gas-industry-faces-microplastics-scrutiny?q=microplastic> – accessed 20-8-2019.

Buxton, L., 2019, Legal opinion casts doubt over proposed EU microplastics restriction, ChemicalWatch, Link: <https://chemicalwatch.com/77579/legal-opinion-casts-doubt-over-proposed-eu-microplastics-restriction?q=microPlastics> - accessed 11-6-2019.

ChemicalWatch (CW), 2018f, Industry prioritises microplastics for chemical risk research funding, Link: https://chemicalwatch-com.proxy.findit.dtu.dk/69887/industry-prioritises-microplastics-for-chemical-risk-research-funding?q=microPlastics - accessed 14-8-2019.

Lovell, T., 2017, Cosmetics firms urge UK to limit microbeads ban to rinse-off products, ChemicalWatch, Link: <https://chemicalwatch.com/54402/cosmetics-firms-urge-uk-to-limit-microbeads-ban-to-rinse-off-products?q=microplastic> – accessed 20-8-2019.

Lovell, T., 2017, UK government urged to target ‘major sources’ of microplastics, ChemicalWatch, Link: <https://chemicalwatch.com/51974/uk-government-urged-to-target-major-sources-of-microplastics?q=microplastic> – accessed 20-8-2019.

Morgan, S., 2018, Commission maps out plastics vision in new strategy, EURACTIV, Link: <https://www.euractiv.com/section/energy-environment/news/commission-maps-out-plastics-vision-in-new-strategy/> - accessed 28-10-2019

Oziel, C., 2018, NGOs attack Echa’s ‘limited’ microplastics restriction proposal, ChemicalWatch, Link: https://chemicalwatch-com.proxy.findit.dtu.dk/67582/ngos-attack-echas-limited-microplastics-restriction-proposal?q=microPlastics - accessed 14-8-2019.

Oziel, C., 2018, Oil and gas industry challenges EU estimate on microplastics use, ChemicalWatch, Link: https://chemicalwatch-com.proxy.findit.dtu.dk/67188/oil-and-gas-industry-challenges-eu-estimate-on-microplastics-use?q=microPlastics - accessed 14-8-2019.

Oziel, C., 2019, Top German sports associations call for artificial turf ban transition, ChemicalWatch, Link: <https://chemicalwatch.com/77732/top-german-sports-associations-call-for-artificial-turf-ban-transition?q=microPlastics> - accessed 11-6-2019.

Ramm, K., 2018, Time to invest in Europe’s water infrastructure, EURACTIV, Link: <https://www.euractiv.com/section/energy-environment/opinion/time-to-invest-in-europes-water-infrastructure/> - accessed 28-10-2019.

Stringer, L., 2019, Feature: EU plastics plan presents opportunities and production challenges, ChemicalWatch, Link: https://chemicalwatch.com/74101/feature-eu-plastics-plan-presents-opportunities-and-production-challenges?q=microPlastics - accessed 12-6-2019.

Tani, C., 2019, Echa definition of microplastics ‘too broad’ – Cefic, ChemicalWatch, Link: https://chemicalwatch.com/74140/echa-definition-of-microplastics-too-broad-cefic?q=microPlastics - accessed 12-6-2019.
