# Supplementary material for: Stakeholder analysis with regard to a recent European restriction proposal on microplastics
Source: PLoS One. 2020 Jun 22;15(6):e0235062. doi: 10.1371/journal.pone.0235062 (PMC7307934; doi:10.1371/journal.pone.0235062)
Supplement: S19 Table — (DOCX) [file pone.0235062.s020.docx]

S19 Table: National NGOs Annex XV comments

| **Stakeholder** | **ECHA date** | **Expressed interests/opinion on microplastics Annex XV comments** |
| --- | --- | --- |
| Germany, National NGO, Fußballverband Niederrhein e.V. | 2019/05/22  **Content:**  Scope or restriction option analysis;  Environmental emissions;  Information on costs;  Other socio economic analysis (SEA) issues;  Transitional period | **Answer to specific info request 2:**  a.: Nach neueren Erkenntnissen werden zwischen 0,25 t/a und 5 t/a in Deutschland an Befüllungsgranulat pro Kunststoffrasenfläche verwendet (Fraunhofer 2018, S. 11). Das entspricht einer Gesamtmenge von ca. 7.500 bis 9.900 t/a.  b.: Nach dem aktuellen Forschungsstand besteht nach Kenntnis des DFB ein hohes Maß an Unsicherheit darüber, wie und in welchen Mengen das als Mikroplastik definierte Granulat auf Sportplätzen in die Umwelt freigesetzt wird. Nach den uns zur Verfügung stehen-den Informationen gibt es große Unterschiede bei der Einschätzung der Menge an Mikroplastiken, die in den einzelnen Mitgliedstaaten oder in der EU/EWR als Füllmaterial für Kunstrasen verwendet wird. Insbesondere Umfang und Methodologie der Forschung in diesem Bereich sind bisher noch wenig standardisiert und nachvollziehbar. Der DFB geht davon aus, dass der Anteil des Eintrags von Mikroplastik über Kunststoffrasenplätze je nach Mitgliedstaat ca. 1 bis 3 Prozent im Verhältnis zum Gesamteintrag beträgt. Demnach ist der Umwelteintrag verglichen mit anderen Hauptquellen relativ gering (Europäische Kommission 2018, ii)).  c.: Gezielte Risikomanagementmaßnahmen können die Freisetzung von Füllstoffen in die Umwelt bereits signifikant vermindern. Technische Maßnahmen zur Zurückhaltung eines Materialaustrags vor Ort (z.B. Rinnenfilter mit Sedimentationsstrecken an Abläufen, Schmutzfangmatten, Schuhbürsten am Ausgang) und organisatorische Maßnahmen beim Betrieb der Sportplätze (z.B. regelmäßige Reinigung der Spielfeldränder, Auffangsiebe) können zu einer starken Verringerung des Austrags von Mikroplastik beitragen.  Neben dem häufig genutzten Kunststoffgranulat existieren für Kunststoffrasensysteme alternative Füllstoffe, die in Teilen auch bereits beim Betrieb von Sportanlagen genutzt werden. So werden in Deutschland aktuell Kunststoffrasenplätze teilweise mit Sand und/oder Kork verfüllt. Zudem gibt es auch Kunststoffrasensysteme, die ohne elastischen Füllstoff betrieben werden können.  Es existieren bisher allerdings nur wenige belastbare Studien darüber, wie sich diese Alternativen qualitäts- und kostenmäßig (z.B. hinsichtlich der Bespielbarkeit und Lebensdauer) vergleichen lassen. Zudem müsste untersucht werden, ob und wie sich die Bespielbar-keit oder das Verletzungsrisiko der alternativ befüllten Kunststoffrasenflächen bei den verschiedenen Alternativfüllungen verändert (Plan Miljø Studie 2017). Es bedarf daher dringend weiterer wissenschaftlicher Expertise zur Praxistauglichkeit alternativer organischer Füllstoffe und zur sportartspezifischen Eignung von Kunststoffrasenplätzen, die ohne Füllstoffe auskommen. Sowohl eine wissenschaftliche Folgenabschätzung als auch die dringend erforderliche Entwicklung alternativer Füllstoffe durch die Industrie sind eine zentrale Forderung der von der Thematik betroffenen Sportverbände in Deutschland. Sie vertreten die Meinung, dass die Maßnahmen, die ein Verbot des Kunststoffgranulats verursachen würden, nicht kurzfristig umsetzbar sind und Alternativen nur mittel- bis langfristig erarbeitet und bereitgestellt werden können.  d.: In Deutschland gibt es ca. 5.000 für den Fußballspielbetrieb gemeldete Kunststoffrasenplätze (DFBnet), sowie ca. 1.000 DFB-Minispielfelder. Jährlich werden in Deutschland ca. 300 Kunststoffrasenplätze neu gebaut, sowie 150 Kunststoffrasenplätze von Grund auf erneuert. Hinsichtlich der bestehenden Plätze dürfte eine Umstellung auf alternative Füllstoffe notwendig sein. Hierfür halten die Sportanlagenbetreiber (Kommunen oder Vereine) Mittel für Sportstättenbau und -sanierung vor, die bei einem vollständigen Ver-bot und einer Verwendung alternativer Füllstoffe deutlich höher ausfallen würden. Laut eigener Berechnungen belaufen sich die jährlichen Mehrkosten deutschlandweit auf einen hohen einstelligen Millionenbetrag. Die insgesamt zu erwartenden Kosten eines Verbotes können aufgrund fehlender Kenntnisse über geeignete alternative Füllstoffe (Geeignetheit, Verfügbarkeit) derzeit nicht seriös beziffert werden. Auf Grundlage aktueller Daten zum Bau von Kunststoffrasenplätzen dürfte der Gesamtbetrag für den Austausch des Füllstoffes der Kunststoffrasensysteme im hohen zweistelligen Millionenbereich (bis zu 90 Mio. EUR) liegen, wobei zur Präzisierung dieses Schätzwertes vertiefte Analysen erforderlich sind. Die Kosten für eine Umsetzung gezielter Risikomanagementmaßnahmen zur Zurückhaltung des Materialaustrags dürften nach Schätzungen und je nach Umfang der Maßnahmen pro Kunststoffrasensystem bei 3.000 bis 10.000 EUR liegen.  e.: Der gemeinwohlorientierte Sport ist die größte zivilgesellschaftliche Bewegung in Deutschland und Europa. In Deutschland engagieren sich knapp acht Millionen Bürger freiwillig und ehrenamtlich im Sport. Das entspricht einer jährlichen Wertschöpfung und einem Wohlfahrtsgewinn allein in Deutschland von ca. 6,7 Milliarden Euro. Vergleichbare Zahlen lassen sich auch für die gesamte EU feststellen. In den EU-Mitgliedstaaten engagieren sich im Jahre 2010 zwischen 92 und 94 Millionen Menschen freiwillig für Ziele des Gemeinwohls, davon die meisten im Sport (ca. 35 bis 40 Prozent aller freiwillig Tätigen in der EU) (Europäische Kommission 2010).  Der Sport schafft ein strukturiertes, an die gesamte Bevölkerung gerichtetes und für alle offenes Bewegungs- und Sportangebot, durch das wichtige soziale und gesundheitsfördernde Funktionen in der Gesellschaft erfüllt werden. Sportvereine in Deutschland zählen zehn Millionen Mitgliedschaften im Kinder- und Jugendalter (DOSB-Bestandserhebung 2018), allein im DFB liegt diese Zahl bei 2,1 Millionen (DFB-Mitgliederstatistik 2018). Damit sind Sportvereine die wichtigste Anlaufstelle für Kinder und Jugendliche außerhalb der Schule und übernehmen unverzichtbare Aufgaben für die ganzheitliche Persönlichkeitsbildung junger Menschen. Dem Sport kommt eine wichtige Vorbild- und Lehrfunktion im Bereich der Integration und demokratischen Grundbildung zu. Für das herausragende gesellschaftliche Engagement des Sports spricht nicht zuletzt, dass die Sportvereine eng mit Schulen, Kindergärten, Unternehmen, Krankenkassen oder anderen öffentlichen Institutionen zusammenarbeiten. Um allen Bürgern den Zugang zum Sport zu ermöglichen, sind adäquate Sportstätten in ausreichender Anzahl Grundvoraussetzung. Ein für alle zugängliches und umfangreiches Sportangebot ist – vor allen Dingen in Großstädten und Ballungsgebieten – nur durch die Verfügbarkeit von ganzjährig nutzbaren Sportanlagen zu gewährleisten. Kunststoffrasenplätze spielen hierbei, insbesondere für den Fußball, eine wichtige Rolle, da sie eine intensivere Nutzung als Naturrasen- oder Tennenplätze erlauben. Allein mit Naturrasen- und Tennenplätzen lässt sich der derzeitige Trainings- und Spielbetrieb, insbesondere bei den Kinder- und Jugendmannschaften, nicht aufrechterhalten. Ein Kunststoffrasenplatz ersetzt etwa 2,5 Naturrasenplätze (DFBnet). Auf weniger als 10 Prozent der Naturrasenplätze finden an Wochenenden mehr als 2 Spiele statt. Bei Kunststoffrasenplätzen finden hingegen bei über 40 Prozent der Plätze mehr als 2 Spiele statt. Weniger als 10 Prozent der Naturrasenplätze wird an einem Wochenende mehr als 150 Minuten genutzt. Bei Kunstrasenplätzen werden hingegen etwa 35 Prozent an einem Wochenende mehr als 150 Minuten genutzt. 27.773 Spielstätten in Deutschland (ca. 70 Prozent) werden von Sportvereinen genutzt. Ein Drittel der Kunstrasenplätze werden von 2 oder mehr Vereinen mit alle ihren Jugend- und Seniorenmannschaften benutzt. Etwas über ein Drittel aller Naturrasenplätze wird von mehr als 5 Mannschaften bespielt. Bei Kunstrasenplätzen werden fast drei Viertel (72 Prozent) von mehr als 5 Mannschaften genutzt. Etwa 10 Prozent aller Naturrasenplätze wird von mehr als 10 Mannschaften bespielt. Bei Kunststoffrasenplätzen sind es ca. 41 Prozent der Plätze, die von mehr als 10 Mannschaften genutzt. Nur 1 Prozent aller Naturrasenplätze wird von mehr als 15 Mannschaften bespielt. Bei Kunstrasenplätzen beträgt der Anteil immerhin noch knapp 18 Prozent. Etwa 6 Prozent werden sogar von über 20 Mannschaften bespielt. Je größer die Vereinsgröße (insbesondere Anzahl der Mannschaften), desto höher ist der Anteil der Vereine, die auch eine Spielstätte vom Typ Kunstrasen haben.  Ein Verbot des Inverkehrbringens von Kunststoffgranulaten als Füllstoff in Kunststoffrasensystemen direkt bei Inkrafttreten der Beschränkung wäre daher unverhältnismäßig. Es würde zu hohen, unerwarteten Umstellungskosten und Mehrkosten für Vereine und Kommunen führen, wodurch dem gemeinwohlorientierten Sport Mittel entzogen würden. Bei fehlender Finanzierbarkeit dieser Mehrkosten ist zudem von einer Schließung vieler Sportplätzen auszugehen, wodurch das Sportangebot in Schulen und Vereinen stark leiden würde. Gerade auf Vereinsebene stellt ein solch außerordentlicher Kosten-punkt ein großes finanzielles Risiko dar, dass das sportliche und gesellschaftliche Gesamtangebot des Vereins gefährden kann. Eine Beschränkung ohne Übergangsfristen, die eine mittelfristige Umstellung und Kostenstreckung erlauben, würde das Breitensportangebot in Deutschland sehr negativ beeinflussen.  Im Hinblick auf den Beschränkungsvorschlag der ECHA gemäß Anhang XV der REACH-Verordnung spricht sich der DFB daher für eine angemessene Übergangsfrist von mindestens sechs Jahren bis zu einem vollständigen Inverkehrbringungsverbot des Kunststoffgranulats zur Verwendung in neuen Kunststoffrasensystemen sowie für die Umstellung bestehender Flächen aus. |
|  |  |  |

| United Kingdom, National NGO, Fidra | 2019/05/30  **Content:**  Scope or restriction option analysis;  Hazard or exposure;  Environmental emissions;  Information on alternatives;  Information on benefits;  Other socio economic analysis (SEA) issues;  Transitional period | **Comment:**  Please see attached document 1 for general comments and specific answers as indicated below |
| --- | --- | --- |
|  |  | **Answer to specific info request 1:**  Please see attached document 1 |
|  |  | **Answer to specific info request 2:**  Please see attached documents:  1 - Fidra response  2 - Fidra & KIMO guidelines to reduce microplastic loss from pitches: Designers and Procurement Specialists  3 - Fidra & KIMO guidelines to reduce microplastic loss from pitches: Pitch owners and Maintenance teams  4 - Fidra & KIMO guidelines to reduce microplastic loss from pitches: Pitch users. |
|  |  | **Answer to specific info request 3:**  Please see document 1 attached |
|  |  | **Answer to specific info request 5:**  Please see documents attached:  1 - Fidra response  5 - Fidra, EIA, FFI summary of Supply Chain Approach |
| Germany, National NGO, Westdeutscher Fußballverband e.V. | 2019/06/06  **Content:**  Scope or restriction option analysis;  Environmental emissions;  Information on costs;  Other socio economic analysis (SEA) issues;  Transitional period | **Comment:**  - |
|  |  | **Answer to specific info request 2:**  a.: Nach neueren Erkenntnissen werden zwischen 0,25 t/a und 5 t/a in Deutschland an Befüllungsgranulat pro Kunststoffrasenfläche verwendet (Fraunhofer 2018, S. 11). Das entspricht einer Gesamtmenge von ca. 7.500 bis 9.900 t/a.  b.: Nach dem aktuellen Forschungsstand besteht nach Kenntnis des DFB ein hohes Maß an Unsicherheit darüber, wie und in welchen Mengen das als Mikroplastik definierte Granulat auf Sportplätzen in die Umwelt freigesetzt wird. Nach den uns zur Verfügung stehen-den Informationen gibt es große Unterschiede bei der Einschätzung der Menge an Mikroplastiken, die in den einzelnen Mitgliedstaaten oder in der EU/EWR als Füllmaterial für Kunstrasen verwendet wird. Insbesondere Umfang und Methodologie der Forschung in diesem Bereich sind bisher noch wenig standardisiert und nachvollziehbar. Der DFB geht davon aus, dass der Anteil des Eintrags von Mikroplastik über Kunststoffrasenplätze je nach Mitgliedstaat ca. 1 bis 3 Prozent im Verhältnis zum Gesamteintrag beträgt. Demnach ist der Umwelteintrag verglichen mit anderen Hauptquellen relativ gering (Europäische Kommission 2018, ii)).  c.: Gezielte Risikomanagementmaßnahmen können die Freisetzung von Füllstoffen in die Umwelt bereits signifikant vermindern. Technische Maßnahmen zur Zurückhaltung eines Materialaustrags vor Ort (z.B. Rinnenfilter mit Sedimentationsstrecken an Abläufen, Schmutzfangmatten, Schuhbürsten am Ausgang) und organisatorische Maßnahmen beim Betrieb der Sportplätze (z.B. regelmäßige Reinigung der Spielfeldränder, Auffangsiebe) können zu einer starken Verringerung des Austrags von Mikroplastik beitragen.  Neben dem häufig genutzten Kunststoffgranulat existieren für Kunststoffrasensysteme alternative Füllstoffe, die in Teilen auch bereits beim Betrieb von Sportanlagen genutzt werden. So werden in Deutschland aktuell Kunststoffrasenplätze teilweise mit Sand und/oder Kork verfüllt. Zudem gibt es auch Kunststoffrasensysteme, die ohne elastischen Füllstoff betrieben werden können.  Es existieren bisher allerdings nur wenige belastbare Studien darüber, wie sich diese Alternativen qualitäts- und kostenmäßig (z.B. hinsichtlich der Bespielbarkeit und Lebensdauer) vergleichen lassen. Zudem müsste untersucht werden, ob und wie sich die Bespielbar-keit oder das Verletzungsrisiko der alternativ befüllten Kunststoffrasenflächen bei den verschiedenen Alternativfüllungen verändert (Plan Miljø Studie 2017). Es bedarf daher dringend weiterer wissenschaftlicher Expertise zur Praxistauglichkeit alternativer organischer Füllstoffe und zur sportartspezifischen Eignung von Kunststoffrasenplätzen, die ohne Füllstoffe auskommen. Sowohl eine wissenschaftliche Folgenabschätzung als auch die dringend erforderliche Entwicklung alternativer Füllstoffe durch die Industrie sind eine zentrale Forderung der von der Thematik betroffenen Sportverbände in Deutschland. Sie vertreten die Meinung, dass die Maßnahmen, die ein Verbot des Kunststoffgranulats verursachen würden, nicht kurzfristig umsetzbar sind und Alternativen nur mittel- bis langfristig erarbeitet und bereitgestellt werden können.  d.: In Deutschland gibt es ca. 5.000 für den Fußballspielbetrieb gemeldete Kunststoffrasenplätze (DFBnet), sowie ca. 1.000 DFB-Minispielfelder. Jährlich werden in Deutschland ca. 300 Kunststoffrasenplätze neu gebaut, sowie 150 Kunststoffrasenplätze von Grund auf erneuert. Hinsichtlich der bestehenden Plätze dürfte eine Umstellung auf alternative Füllstoffe notwendig sein. Hierfür halten die Sportanlagenbetreiber (Kommunen oder Vereine) Mittel für Sportstättenbau und -sanierung vor, die bei einem vollständigen Ver-bot und einer Verwendung alternativer Füllstoffe deutlich höher ausfallen würden. Laut eigener Berechnungen belaufen sich die jährlichen Mehrkosten deutschlandweit auf einen hohen einstelligen Millionenbetrag. Die insgesamt zu erwartenden Kosten eines Verbotes können aufgrund fehlender Kenntnisse über geeignete alternative Füllstoffe (Geeignetheit, Verfügbarkeit) derzeit nicht seriös beziffert werden. Auf Grundlage aktueller Daten zum Bau von Kunststoffrasenplätzen dürfte der Gesamtbetrag für den Austausch des Füllstoffes der Kunststoffrasensysteme im hohen zweistelligen Millionenbereich (bis zu 90 Mio. EUR) liegen, wobei zur Präzisierung dieses Schätzwertes vertiefte Analysen erforderlich sind. Die Kosten für eine Umsetzung gezielter Risikomanagementmaßnahmen zur Zurückhaltung des Materialaustrags dürften nach Schätzungen und je nach Umfang der Maßnahmen pro Kunststoffrasensystem bei 3.000 bis 10.000 EUR liegen.  e.: Der gemeinwohlorientierte Sport ist die größte zivilgesellschaftliche Bewegung in Deutschland und Europa. In Deutschland engagieren sich knapp acht Millionen Bürger freiwillig und ehrenamtlich im Sport. Das entspricht einer jährlichen Wertschöpfung und einem Wohlfahrtsgewinn allein in Deutschland von ca. 6,7 Milliarden Euro. Vergleichbare Zahlen lassen sich auch für die gesamte EU feststellen. In den EU-Mitgliedstaaten engagieren sich im Jahre 2010 zwischen 92 und 94 Millionen Menschen freiwillig für Ziele des Gemeinwohls, davon die meisten im Sport (ca. 35 bis 40 Prozent aller freiwillig Tätigen in der EU) (Europäische Kommission 2010). Der Westdeutsche Fußballverband e. V. (WDFV) ist der größte Fachsportverband in Nordrhein-Westfalen. Er vertritt mit seinen drei Mitgliedsverbänden mehr als 1,6 Millionen Sportler in rund 4.600 Vereinen.  Der Sport schafft ein strukturiertes, an die gesamte Bevölkerung gerichtetes und für alle offenes Bewegungs- und Sportangebot, durch das wichtige soziale und gesundheitsfördernde Funktionen in der Gesellschaft erfüllt werden. Sportvereine in Deutschland zählen zehn Millionen Mitgliedschaften im Kinder- und Jugendalter (DOSB-Bestandserhebung 2018), allein im DFB liegt diese Zahl bei 2,1 Millionen (DFB-Mitgliederstatistik 2018). Damit sind Sportvereine die wichtigste Anlaufstelle für Kinder und Jugendliche außerhalb der Schule und übernehmen unverzichtbare Aufgaben für die ganzheitliche Persönlichkeitsbildung junger Menschen. Dem Sport kommt eine wichtige Vorbild- und Lehrfunktion im Bereich der Integration und demokratischen Grundbildung zu. Für das herausragende gesellschaftliche Engagement des Sports spricht nicht zuletzt, dass die Sportvereine eng mit Schulen, Kindergärten, Unternehmen, Krankenkassen oder anderen öffentlichen Institutionen zusammenarbeiten. Um allen Bürgern den Zugang zum Sport zu ermöglichen, sind adäquate Sportstätten in ausreichender Anzahl Grundvoraussetzung. Ein für alle zugängliches und umfangreiches Sportangebot ist – vor allen Dingen in Großstädten und Ballungsgebieten – nur durch die Verfügbarkeit von ganzjährig nutzbaren Sportanlagen zu gewährleisten. Kunststoffrasenplätze spielen hierbei, insbesondere für den Fußball, eine wichtige Rolle, da sie eine intensivere Nutzung als Naturrasen- oder Tennenplätze erlauben. Allein mit Naturrasen- und Tennenplätzen lässt sich der derzeitige Trainings- und Spielbetrieb, insbesondere bei den Kinder- und Jugendmannschaften, nicht aufrechterhalten. Ein Kunststoffrasenplatz ersetzt etwa 2,5 Naturrasenplätze (DFBnet). Auf weniger als 10 Prozent der Naturrasenplätze finden an Wochenenden mehr als 2 Spiele statt. Bei Kunststoffrasenplätzen finden hingegen bei über 40 Prozent der Plätze mehr als 2 Spiele statt. Weniger als 10 Prozent der Naturrasenplätze wird an einem Wochenende mehr als 150 Minuten genutzt. Bei Kunstrasenplätzen werden hingegen etwa 35 Prozent an einem Wochenende mehr als 150 Minuten genutzt. 27.773 Spielstätten in Deutschland (ca. 70 Prozent) werden von Sportvereinen genutzt. Ein Drittel der Kunstrasenplätze werden von 2 oder mehr Vereinen mit alle ihren Jugend- und Seniorenmannschaften benutzt. Etwas über ein Drittel aller Naturrasenplätze wird von mehr als 5 Mannschaften bespielt. Bei Kunstrasenplätzen werden fast drei Viertel (72 Prozent) von mehr als 5 Mannschaften genutzt. Etwa 10 Prozent aller Naturrasenplätze wird von mehr als 10 Mannschaften bespielt. Bei Kunststoffrasenplätzen sind es ca. 41 Prozent der Plätze, die von mehr als 10 Mannschaften genutzt. Nur 1 Prozent aller Naturrasenplätze wird von mehr als 15 Mannschaften bespielt. Bei Kunstrasenplätzen beträgt der Anteil immerhin noch knapp 18 Prozent. Etwa 6 Prozent werden sogar von über 20 Mannschaften bespielt. Je größer die Vereinsgröße (insbesondere Anzahl der Mannschaften), desto höher ist der Anteil der Vereine, die auch eine Spielstätte vom Typ Kunstrasen haben.  Ein Verbot des Inverkehrbringens von Kunststoffgranulaten als Füllstoff in Kunststoffrasensystemen direkt bei Inkrafttreten der Beschränkung wäre daher unverhältnismäßig. Es würde zu hohen, unerwarteten Umstellungskosten und Mehrkosten für Vereine und Kommunen führen, wodurch dem gemeinwohlorientierten Sport Mittel entzogen würden. Bei fehlender Finanzierbarkeit dieser Mehrkosten ist zudem von einer Schließung vieler Sportplätzen auszugehen, wodurch das Sportangebot in Schulen und Vereinen stark leiden würde. Gerade auf Vereinsebene stellt ein solch außerordentlicher Kosten-punkt ein großes finanzielles Risiko dar, dass das sportliche und gesellschaftliche Gesamtangebot des Vereins gefährden kann. Eine Beschränkung ohne Übergangsfristen, die eine mittelfristige Umstellung und Kostenstreckung erlauben, würde das Breitensportangebot in Deutschland sehr negativ beeinflussen.  Im Hinblick auf den Beschränkungsvorschlag der ECHA gemäß Anhang XV der REACH-Verordnung spricht sich der DFB daher für eine angemessene Übergangsfrist von mindestens sechs Jahren bis zu einem vollständigen Inverkehrbringungsverbot des Kunststoffgranulats zur Verwendung in neuen Kunststoffrasensystemen sowie für die Umstellung bestehender Flächen aus. |
| Austria, National NGO, l GLOBAL 2000 - Friends of the Earth Austria | 2019/07/12  **Content:**  Scope or restriction option analysis;  Description of analytical methods | 36 detergent samples were tested in the laboratory of the Federal Environment Agency Austria for possible microplastic compounds (> 50 μm) . In addition the ingredients -according to the ingredients list provided online by the producer - of more than 300 detergents have been compared to the list of 520 polymers published by ECHA early 2019 (Annex XV Restriction Report). In 119 detergents microplastics and/or synthetic Polymers form the 520 Polymer scenario were found. Furthermore detergent producers were contacted and asked if synthetic polymers in their products are water soluble or not. This information is reflected in the report.  The main conclusions are:  1. A comprehensive ban on all non-biodegradable synthetic polymers is urgently needed.  2. With the laboratory test used it was not possible to detect all non water soluble polymers that are in the detergents. Without better and standardized testing methods a restriction will not be feasible.  3. We call for legislation that ensures that in all cases all ingredients are listed on packaging, just like with personal care products. At the moment European legislation permits for cleaning products to reference to a website for a complete list of ingredients by product. |

| **Stakeholder** | **ECHA date** | **Expressed interests/opinion on microplastics Annex XV comments** |
| --- | --- | --- |
| Germany  Südwestdeutscher Fußballverband (National NGO, Germany) | 2019/05/15  **Content:**  Scope or restriction option analysis;  Environmental emissions;  Information on costs;  Other socio economic analysis (SEA) issues;  Transitional period | **Answer to specific info request 2:**  a.: Nach neueren Erkenntnissen werden zwischen 0,25 t/a und 5 t/a in Deutschland an Befüllungsgranulat pro Kunststoffrasenfläche verwendet (Fraunhofer 2018, S. 11). Das entspricht einer Gesamtmenge von ca. 7.500 bis 9.900 t/a.  b.: Nach dem aktuellen Forschungsstand besteht nach Kenntnis des DFB ein hohes Maß an Unsicherheit darüber, wie und in welchen Mengen das als Mikroplastik definierte Granulat auf Sportplätzen in die Umwelt freigesetzt wird. Nach den uns zur Verfügung stehen-den Informationen gibt es große Unterschiede bei der Einschätzung der Menge an Mikroplastiken, die in den einzelnen Mitgliedstaaten oder in der EU/EWR als Füllmaterial für Kunstrasen verwendet wird. Insbesondere Umfang und Methodologie der Forschung in diesem Bereich sind bisher noch wenig standardisiert und nachvollziehbar. Der DFB geht davon aus, dass der Anteil des Eintrags von Mikroplastik über Kunststoffrasenplätze je nach Mitgliedstaat ca. 1 bis 3 Prozent im Verhältnis zum Gesamteintrag beträgt. Demnach ist der Umwelteintrag verglichen mit anderen Hauptquellen relativ gering (Europäische Kommission 2018, ii)).  c.: Gezielte Risikomanagementmaßnahmen können die Freisetzung von Füllstoffen in die Umwelt bereits signifikant vermindern. Technische Maßnahmen zur Zurückhaltung eines Materialaustrags vor Ort (z.B. Rinnenfilter mit Sedimentationsstrecken an Abläufen, Schmutzfangmatten, Schuhbürsten am Ausgang) und organisatorische Maßnahmen beim Betrieb der Sportplätze (z.B. regelmäßige Reinigung der Spielfeldränder, Auffangsiebe) können zu einer starken Verringerung des Austrags von Mikroplastik beitragen.  Neben dem häufig genutzten Kunststoffgranulat existieren für Kunststoffrasensysteme alternative Füllstoffe, die in Teilen auch bereits beim Betrieb von Sportanlagen genutzt werden. So werden in Deutschland aktuell Kunststoffrasenplätze teilweise mit Sand und/oder Kork verfüllt. Zudem gibt es auch Kunststoffrasensysteme, die ohne elastischen Füllstoff betrieben werden können.  Es existieren bisher allerdings nur wenige belastbare Studien darüber, wie sich diese Alternativen qualitäts- und kostenmäßig (z.B. hinsichtlich der Bespielbarkeit und Lebensdauer) vergleichen lassen. Zudem müsste untersucht werden, ob und wie sich die Bespielbar-keit oder das Verletzungsrisiko der alternativ befüllten Kunststoffrasenflächen bei den verschiedenen Alternativfüllungen verändert (Plan Miljø Studie 2017). Es bedarf daher dringend weiterer wissenschaftlicher Expertise zur Praxistauglichkeit alternativer organischer Füllstoffe und zur sportartspezifischen Eignung von Kunststoffrasenplätzen, die ohne Füllstoffe auskommen. Sowohl eine wissenschaftliche Folgenabschätzung als auch die dringend erforderliche Entwicklung alternativer Füllstoffe durch die Industrie sind eine zentrale Forderung der von der Thematik betroffenen Sportverbände in Deutschland. Sie vertreten die Meinung, dass die Maßnahmen, die ein Verbot des Kunststoffgranulats verursachen würden, nicht kurzfristig umsetzbar sind und Alternativen nur mittel- bis langfristig erarbeitet und bereitgestellt werden können.  d.: In Deutschland gibt es ca. 5.000 für den Fußballspielbetrieb gemeldete Kunststoffrasenplätze (DFBnet), sowie ca. 1.000 DFB-Minispielfelder. Jährlich werden in Deutschland ca. 300 Kunststoffrasenplätze neu gebaut, sowie 150 Kunststoffrasenplätze von Grund auf erneuert. Hinsichtlich der bestehenden Plätze dürfte eine Umstellung auf alternative Füllstoffe notwendig sein. Hierfür halten die Sportanlagenbetreiber (Kommunen oder Vereine) Mittel für Sportstättenbau und -sanierung vor, die bei einem vollständigen Ver-bot und einer Verwendung alternativer Füllstoffe deutlich höher ausfallen würden. Laut eigener Berechnungen belaufen sich die jährlichen Mehrkosten deutschlandweit auf einen hohen einstelligen Millionenbetrag. Die insgesamt zu erwartenden Kosten eines Verbotes können aufgrund fehlender Kenntnisse über geeignete alternative Füllstoffe (Geeignetheit, Verfügbarkeit) derzeit nicht seriös beziffert werden. Auf Grundlage aktueller Daten zum Bau von Kunststoffrasenplätzen dürfte der Gesamtbetrag für den Austausch des Füllstoffes der Kunststoffrasensysteme im hohen zweistelligen Millionenbereich (bis zu 90 Mio. EUR) liegen, wobei zur Präzisierung dieses Schätzwertes vertiefte Analysen erforderlich sind. Die Kosten für eine Umsetzung gezielter Risikomanagementmaßnahmen zur Zurückhaltung des Materialaustrags dürften nach Schätzungen und je nach Umfang der Maßnahmen pro Kunststoffrasensystem bei 3.000 bis 10.000 EUR liegen.  e.: Der gemeinwohlorientierte Sport ist die größte zivilgesellschaftliche Bewegung in Deutschland und Europa. In Deutschland engagieren sich knapp acht Millionen Bürger freiwillig und ehrenamtlich im Sport. Das entspricht einer jährlichen Wertschöpfung und einem Wohlfahrtsgewinn allein in Deutschland von ca. 6,7 Milliarden Euro. Vergleichbare Zahlen lassen sich auch für die gesamte EU feststellen. In den EU-Mitgliedstaaten engagieren sich im Jahre 2010 zwischen 92 und 94 Millionen Menschen freiwillig für Ziele des Gemeinwohls, davon die meisten im Sport (ca. 35 bis 40 Prozent aller freiwillig Tätigen in der EU) (Europäische Kommission 2010).  Der Sport schafft ein strukturiertes, an die gesamte Bevölkerung gerichtetes und für alle offenes Bewegungs- und Sportangebot, durch das wichtige soziale und gesundheitsfördernde Funktionen in der Gesellschaft erfüllt werden. Sportvereine in Deutschland zählen zehn Millionen Mitgliedschaften im Kinder- und Jugendalter (DOSB-Bestandserhebung 2018), allein im DFB liegt diese Zahl bei 2,1 Millionen (DFB-Mitgliederstatistik 2018). Damit sind Sportvereine die wichtigste Anlaufstelle für Kinder und Jugendliche außerhalb der Schule und übernehmen unverzichtbare Aufgaben für die ganzheitliche Persönlichkeitsbildung junger Menschen. Dem Sport kommt eine wichtige Vorbild- und Lehrfunktion im Bereich der Integration und demokratischen Grundbildung zu. Für das herausragende gesellschaftliche Engagement des Sports spricht nicht zuletzt, dass die Sportvereine eng mit Schulen, Kindergärten, Unternehmen, Krankenkassen oder anderen öffentlichen Institutionen zusammenarbeiten. Um allen Bürgern den Zugang zum Sport zu ermöglichen, sind adäquate Sportstätten in ausreichender Anzahl Grundvoraussetzung. Ein für alle zugängliches und umfangreiches Sportangebot ist – vor allen Dingen in Großstädten und Ballungsgebieten – nur durch die Verfügbarkeit von ganzjährig nutzbaren Sportanlagen zu gewährleisten. Kunststoffrasenplätze spielen hierbei, insbesondere für den Fußball, eine wichtige Rolle, da sie eine intensivere Nutzung als Naturrasen- oder Tennenplätze erlauben. Allein mit Naturrasen- und Tennenplätzen lässt sich der derzeitige Trainings- und Spielbetrieb, insbesondere bei den Kinder- und Jugendmannschaften, nicht aufrechterhalten. Ein Kunststoffrasenplatz ersetzt etwa 2,5 Naturrasenplätze (DFBnet). Auf weniger als 10 Prozent der Naturrasenplätze finden an Wochenenden mehr als 2 Spiele statt. Bei Kunststoffrasenplätzen finden hingegen bei über 40 Prozent der Plätze mehr als 2 Spiele statt. Weniger als 10 Prozent der Naturrasenplätze wird an einem Wochenende mehr als 150 Minuten genutzt. Bei Kunstrasenplätzen werden hingegen etwa 35 Prozent an einem Wochenende mehr als 150 Minuten genutzt. 27.773 Spielstätten in Deutschland (ca. 70 Prozent) werden von Sportvereinen genutzt. Ein Drittel der Kunstrasenplätze werden von 2 oder mehr Vereinen mit alle ihren Jugend- und Seniorenmannschaften benutzt. Etwas über ein Drittel aller Naturrasenplätze wird von mehr als 5 Mannschaften bespielt. Bei Kunstrasenplätzen werden fast drei Viertel (72 Prozent) von mehr als 5 Mannschaften genutzt. Etwa 10 Prozent aller Naturrasenplätze wird von mehr als 10 Mannschaften bespielt. Bei Kunststoffrasenplätzen sind es ca. 41 Prozent der Plätze, die von mehr als 10 Mannschaften genutzt. Nur 1 Prozent aller Naturrasenplätze wird von mehr als 15 Mannschaften bespielt. Bei Kunstrasenplätzen beträgt der Anteil immerhin noch knapp 18 Prozent. Etwa 6 Prozent werden sogar von über 20 Mannschaften bespielt. Je größer die Vereinsgröße (insbesondere Anzahl der Mannschaften), desto höher ist der Anteil der Vereine, die auch eine Spielstätte vom Typ Kunstrasen haben.  Ein Verbot des Inverkehrbringens von Kunststoffgranulaten als Füllstoff in Kunststoffrasensystemen direkt bei Inkrafttreten der Beschränkung wäre daher unverhältnismäßig. Es würde zu hohen, unerwarteten Umstellungskosten und Mehrkosten für Vereine und Kommunen führen, wodurch dem gemeinwohlorientierten Sport Mittel entzogen würden. Bei fehlender Finanzierbarkeit dieser Mehrkosten ist zudem von einer Schließung vieler Sportplätzen auszugehen, wodurch das Sportangebot in Schulen und Vereinen stark leiden würde. Gerade auf Vereinsebene stellt ein solch außerordentlicher Kosten-punkt ein großes finanzielles Risiko dar, dass das sportliche und gesellschaftliche Gesamtangebot des Vereins gefährden kann. Eine Beschränkung ohne Übergangsfristen, die eine mittelfristige Umstellung und Kostenstreckung erlauben, würde das Breitensportangebot in Deutschland sehr negativ beeinflussen.  Im Hinblick auf den Beschränkungsvorschlag der ECHA gemäß Anhang XV der REACH-Verordnung spricht sich der DFB daher für eine angemessene Übergangsfrist von mindestens sechs Jahren bis zu einem vollständigen Inverkehrbringungsverbot des Kunststoffgranulats zur Verwendung in neuen Kunststoffrasensystemen sowie für die Umstellung bestehender Flächen aus. |
|  |  |  |
| Germany, Berliner Fußball-Verband,  National NGO | 2019/05/20  **Content:**  Environmental emissions;  Information on costs;  Other socio economic analysis (SEA) issues;  Transitional period | **Answer to specific info request 2:**  a.: Nach neueren Erkenntnissen werden zwischen 0,25 t/a und 5 t/a in Deutschland an Befüllungsgranulat pro Kunststoffrasenfläche verwendet (Fraunhofer 2018, S. 11). Das entspricht einer Gesamtmenge von ca. 7.500 bis 9.900 t/a.  b.: Nach dem aktuellen Forschungsstand besteht nach Kenntnis des DFB ein hohes Maß an Unsicherheit darüber, wie und in welchen Mengen das als Mikroplastik definierte Granulat auf Sportplätzen in die Umwelt freigesetzt wird. Nach den uns zur Verfügung stehen-den Informationen gibt es große Unterschiede bei der Einschätzung der Menge an Mikroplastiken, die in den einzelnen Mitgliedstaaten oder in der EU/EWR als Füllmaterial für Kunstrasen verwendet wird. Insbesondere Umfang und Methodologie der Forschung in diesem Bereich sind bisher noch wenig standardisiert und nachvollziehbar. Der DFB geht davon aus, dass der Anteil des Eintrags von Mikroplastik über Kunststoffrasenplätze je nach Mitgliedstaat ca. 1 bis 3 Prozent im Verhältnis zum Gesamteintrag beträgt. Demnach ist der Umwelteintrag verglichen mit anderen Hauptquellen relativ gering (Europäische Kommission 2018, ii)).  c.: Gezielte Risikomanagementmaßnahmen können die Freisetzung von Füllstoffen in die Umwelt bereits signifikant vermindern. Technische Maßnahmen zur Zurückhaltung eines Materialaustrags vor Ort (z.B. Rinnenfilter mit Sedimentationsstrecken an Abläufen, Schmutzfangmatten, Schuhbürsten am Ausgang) und organisatorische Maßnahmen beim Betrieb der Sportplätze (z.B. regelmäßige Reinigung der Spielfeldränder, Auffangsiebe) können zu einer starken Verringerung des Austrags von Mikroplastik beitragen.  Neben dem häufig genutzten Kunststoffgranulat existieren für Kunststoffrasensysteme alternative Füllstoffe, die in Teilen auch bereits beim Betrieb von Sportanlagen genutzt werden. So werden in Deutschland aktuell Kunststoffrasenplätze teilweise mit Sand und/oder Kork verfüllt. Zudem gibt es auch Kunststoffrasensysteme, die ohne elastischen Füllstoff betrieben werden können.  Es existieren bisher allerdings nur wenige belastbare Studien darüber, wie sich diese Alternativen qualitäts- und kostenmäßig (z.B. hinsichtlich der Bespielbarkeit und Lebensdauer) vergleichen lassen. Zudem müsste untersucht werden, ob und wie sich die Bespielbar-keit oder das Verletzungsrisiko der alternativ befüllten Kunststoffrasenflächen bei den verschiedenen Alternativfüllungen verändert (Plan Miljø Studie 2017). Es bedarf daher dringend weiterer wissenschaftlicher Expertise zur Praxistauglichkeit alternativer organischer Füllstoffe und zur sportartspezifischen Eignung von Kunststoffrasenplätzen, die ohne Füllstoffe auskommen. Sowohl eine wissenschaftliche Folgenabschätzung als auch die dringend erforderliche Entwicklung alternativer Füllstoffe durch die Industrie sind eine zentrale Forderung der von der Thematik betroffenen Sportverbände in Deutschland. Sie vertreten die Meinung, dass die Maßnahmen, die ein Verbot des Kunststoffgranulats verursachen würden, nicht kurzfristig umsetzbar sind und Alternativen nur mittel- bis langfristig erarbeitet und bereitgestellt werden können.  d.: In Deutschland gibt es ca. 5.000 für den Fußballspielbetrieb gemeldete Kunststoffrasenplätze (DFBnet), sowie ca. 1.000 DFB-Minispielfelder. Jährlich werden in Deutschland ca. 300 Kunststoffrasenplätze neu gebaut, sowie 150 Kunststoffrasenplätze von Grund auf erneuert. Hinsichtlich der bestehenden Plätze dürfte eine Umstellung auf alternative Füllstoffe notwendig sein. Hierfür halten die Sportanlagenbetreiber (Kommunen oder Vereine) Mittel für Sportstättenbau und -sanierung vor, die bei einem vollständigen Ver-bot und einer Verwendung alternativer Füllstoffe deutlich höher ausfallen würden. Laut eigener Berechnungen belaufen sich die jährlichen Mehrkosten deutschlandweit auf einen hohen einstelligen Millionenbetrag. Die insgesamt zu erwartenden Kosten eines Verbotes können aufgrund fehlender Kenntnisse über geeignete alternative Füllstoffe (Geeignetheit, Verfügbarkeit) derzeit nicht seriös beziffert werden. Auf Grundlage aktueller Daten zum Bau von Kunststoffrasenplätzen dürfte der Gesamtbetrag für den Austausch des Füllstoffes der Kunststoffrasensysteme im hohen zweistelligen Millionenbereich (bis zu 90 Mio. EUR) liegen, wobei zur Präzisierung dieses Schätzwertes vertiefte Analysen erforderlich sind. Die Kosten für eine Umsetzung gezielter Risikomanagementmaßnahmen zur Zurückhaltung des Materialaustrags dürften nach Schätzungen und je nach Umfang der Maßnahmen pro Kunststoffrasensystem bei 3.000 bis 10.000 EUR liegen.  e.: Der gemeinwohlorientierte Sport ist die größte zivilgesellschaftliche Bewegung in Deutschland und Europa. In Deutschland engagieren sich knapp acht Millionen Bürger freiwillig und ehrenamtlich im Sport. Das entspricht einer jährlichen Wertschöpfung und einem Wohlfahrtsgewinn allein in Deutschland von ca. 6,7 Milliarden Euro. Vergleichbare Zahlen lassen sich auch für die gesamte EU feststellen. In den EU-Mitgliedstaaten engagieren sich im Jahre 2010 zwischen 92 und 94 Millionen Menschen freiwillig für Ziele des Gemeinwohls, davon die meisten im Sport (ca. 35 bis 40 Prozent aller freiwillig Tätigen in der EU) (Europäische Kommission 2010).  Der Sport schafft ein strukturiertes, an die gesamte Bevölkerung gerichtetes und für alle offenes Bewegungs- und Sportangebot, durch das wichtige soziale und gesundheitsfördernde Funktionen in der Gesellschaft erfüllt werden. Sportvereine in Deutschland zählen zehn Millionen Mitgliedschaften im Kinder- und Jugendalter (DOSB-Bestandserhebung 2018), allein im DFB liegt diese Zahl bei 2,1 Millionen (DFB-Mitgliederstatistik 2018). Damit sind Sportvereine die wichtigste Anlaufstelle für Kinder und Jugendliche außerhalb der Schule und übernehmen unverzichtbare Aufgaben für die ganzheitliche Persönlichkeitsbildung junger Menschen. Dem Sport kommt eine wichtige Vorbild- und Lehrfunktion im Bereich der Integration und demokratischen Grundbildung zu. Für das herausragende gesellschaftliche Engagement des Sports spricht nicht zuletzt, dass die Sportvereine eng mit Schulen, Kindergärten, Unternehmen, Krankenkassen oder anderen öffentlichen Institutionen zusammenarbeiten. Um allen Bürgern den Zugang zum Sport zu ermöglichen, sind adäquate Sportstätten in ausreichender Anzahl Grundvoraussetzung. Ein für alle zugängliches und umfangreiches Sportangebot ist – vor allen Dingen in Großstädten und Ballungsgebieten – nur durch die Verfügbarkeit von ganzjährig nutzbaren Sportanlagen zu gewährleisten. Kunststoffrasenplätze spielen hierbei, insbesondere für den Fußball, eine wichtige Rolle, da sie eine intensivere Nutzung als Naturrasen- oder Tennenplätze erlauben. Allein mit Naturrasen- und Tennenplätzen lässt sich der derzeitige Trainings- und Spielbetrieb, insbesondere bei den Kinder- und Jugendmannschaften, nicht aufrechterhalten. Ein Kunststoffrasenplatz ersetzt etwa 2,5 Naturrasenplätze (DFBnet). Auf weniger als 10 Prozent der Naturrasenplätze finden an Wochenenden mehr als 2 Spiele statt. Bei Kunststoffrasenplätzen finden hingegen bei über 40 Prozent der Plätze mehr als 2 Spiele statt. Weniger als 10 Prozent der Naturrasenplätze wird an einem Wochenende mehr als 150 Minuten genutzt. Bei Kunstrasenplätzen werden hingegen etwa 35 Prozent an einem Wochenende mehr als 150 Minuten genutzt. 27.773 Spielstätten in Deutschland (ca. 70 Prozent) werden von Sportvereinen genutzt. Ein Drittel der Kunstrasenplätze werden von 2 oder mehr Vereinen mit alle ihren Jugend- und Seniorenmannschaften benutzt. Etwas über ein Drittel aller Naturrasenplätze wird von mehr als 5 Mannschaften bespielt. Bei Kunstrasenplätzen werden fast drei Viertel (72 Prozent) von mehr als 5 Mannschaften genutzt. Etwa 10 Prozent aller Naturrasenplätze wird von mehr als 10 Mannschaften bespielt. Bei Kunststoffrasenplätzen sind es ca. 41 Prozent der Plätze, die von mehr als 10 Mannschaften genutzt. Nur 1 Prozent aller Naturrasenplätze wird von mehr als 15 Mannschaften bespielt. Bei Kunstrasenplätzen beträgt der Anteil immerhin noch knapp 18 Prozent. Etwa 6 Prozent werden sogar von über 20 Mannschaften bespielt. Je größer die Vereinsgröße (insbesondere Anzahl der Mannschaften), desto höher ist der Anteil der Vereine, die auch eine Spielstätte vom Typ Kunstrasen haben.  Ein Verbot des Inverkehrbringens von Kunststoffgranulaten als Füllstoff in Kunststoffrasensystemen direkt bei Inkrafttreten der Beschränkung wäre daher unverhältnismäßig. Es würde zu hohen, unerwarteten Umstellungskosten und Mehrkosten für Vereine und Kommunen führen, wodurch dem gemeinwohlorientierten Sport Mittel entzogen würden. Bei fehlender Finanzierbarkeit dieser Mehrkosten ist zudem von einer Schließung vieler Sportplätzen auszugehen, wodurch das Sportangebot in Schulen und Vereinen stark leiden würde. Gerade auf Vereinsebene stellt ein solch außerordentlicher Kosten-punkt ein großes finanzielles Risiko dar, dass das sportliche und gesellschaftliche Gesamtangebot des Vereins gefährden kann. Eine Beschränkung ohne Übergangsfristen, die eine mittelfristige Umstellung und Kostenstreckung erlauben, würde das Breitensportangebot in Deutschland sehr negativ beeinflussen.  Im Hinblick auf den Beschränkungsvorschlag der ECHA gemäß Anhang XV der REACH-Verordnung spricht sich der DFB daher für eine angemessene Übergangsfrist von mindestens sechs Jahren bis zu einem vollständigen Inverkehrbringungsverbot des Kunststoffgranulats zur Verwendung in neuen Kunststoffrasensystemen sowie für die Umstellung bestehender Flächen aus. |
| Germany, National NGO, Fußballverband Rheinland | 2019/05/20  **Content:**  Environmental emissions;  Information on costs;  Other socio economic analysis (SEA) issues;  Transitional period | **Answer to specific info request 2:**  a.: Nach neueren Erkenntnissen werden zwischen 0,25 t/a und 5 t/a in Deutschland an Befüllungsgranulat pro Kunststoffrasenfläche verwendet (Fraunhofer 2018, S. 11). Das entspricht einer Gesamtmenge von ca. 7.500 bis 9.900 t/a.  b.: Nach dem aktuellen Forschungsstand besteht nach Kenntnis des DFB ein hohes Maß an Unsicherheit darüber, wie und in welchen Mengen das als Mikroplastik definierte Granulat auf Sportplätzen in die Umwelt freigesetzt wird. Nach den uns zur Verfügung stehen-den Informationen gibt es große Unterschiede bei der Einschätzung der Menge an Mikroplastiken, die in den einzelnen Mitgliedstaaten oder in der EU/EWR als Füllmaterial für Kunstrasen verwendet wird. Insbesondere Umfang und Methodologie der Forschung in diesem Bereich sind bisher noch wenig standardisiert und nachvollziehbar. Der DFB geht davon aus, dass der Anteil des Eintrags von Mikroplastik über Kunststoffrasenplätze je nach Mitgliedstaat ca. 1 bis 3 Prozent im Verhältnis zum Gesamteintrag beträgt. Demnach ist der Umwelteintrag verglichen mit anderen Hauptquellen relativ gering (Europäische Kommission 2018, ii)).  c.: Gezielte Risikomanagementmaßnahmen können die Freisetzung von Füllstoffen in die Umwelt bereits signifikant vermindern. Technische Maßnahmen zur Zurückhaltung eines Materialaustrags vor Ort (z.B. Rinnenfilter mit Sedimentationsstrecken an Abläufen, Schmutzfangmatten, Schuhbürsten am Ausgang) und organisatorische Maßnahmen beim Betrieb der Sportplätze (z.B. regelmäßige Reinigung der Spielfeldränder, Auffangsiebe) können zu einer starken Verringerung des Austrags von Mikroplastik beitragen.  Neben dem häufig genutzten Kunststoffgranulat existieren für Kunststoffrasensysteme alternative Füllstoffe, die in Teilen auch bereits beim Betrieb von Sportanlagen genutzt werden. So werden in Deutschland aktuell Kunststoffrasenplätze teilweise mit Sand und/oder Kork verfüllt. Zudem gibt es auch Kunststoffrasensysteme, die ohne elastischen Füllstoff betrieben werden können.  Es existieren bisher allerdings nur wenige belastbare Studien darüber, wie sich diese Alternativen qualitäts- und kostenmäßig (z.B. hinsichtlich der Bespielbarkeit und Lebensdauer) vergleichen lassen. Zudem müsste untersucht werden, ob und wie sich die Bespielbar-keit oder das Verletzungsrisiko der alternativ befüllten Kunststoffrasenflächen bei den verschiedenen Alternativfüllungen verändert (Plan Miljø Studie 2017). Es bedarf daher dringend weiterer wissenschaftlicher Expertise zur Praxistauglichkeit alternativer organischer Füllstoffe und zur sportartspezifischen Eignung von Kunststoffrasenplätzen, die ohne Füllstoffe auskommen. Sowohl eine wissenschaftliche Folgenabschätzung als auch die dringend erforderliche Entwicklung alternativer Füllstoffe durch die Industrie sind eine zentrale Forderung der von der Thematik betroffenen Sportverbände in Deutschland. Sie vertreten die Meinung, dass die Maßnahmen, die ein Verbot des Kunststoffgranulats verursachen würden, nicht kurzfristig umsetzbar sind und Alternativen nur mittel- bis langfristig erarbeitet und bereitgestellt werden können.  d.: In Deutschland gibt es ca. 5.000 für den Fußballspielbetrieb gemeldete Kunststoffrasenplätze (DFBnet), sowie ca. 1.000 DFB-Minispielfelder. Jährlich werden in Deutschland ca. 300 Kunststoffrasenplätze neu gebaut, sowie 150 Kunststoffrasenplätze von Grund auf erneuert. Hinsichtlich der bestehenden Plätze dürfte eine Umstellung auf alternative Füllstoffe notwendig sein. Hierfür halten die Sportanlagenbetreiber (Kommunen oder Vereine) Mittel für Sportstättenbau und -sanierung vor, die bei einem vollständigen Ver-bot und einer Verwendung alternativer Füllstoffe deutlich höher ausfallen würden. Laut eigener Berechnungen belaufen sich die jährlichen Mehrkosten deutschlandweit auf einen hohen einstelligen Millionenbetrag. Die insgesamt zu erwartenden Kosten eines Verbotes können aufgrund fehlender Kenntnisse über geeignete alternative Füllstoffe (Geeignetheit, Verfügbarkeit) derzeit nicht seriös beziffert werden. Auf Grundlage aktueller Daten zum Bau von Kunststoffrasenplätzen dürfte der Gesamtbetrag für den Austausch des Füllstoffes der Kunststoffrasensysteme im hohen zweistelligen Millionenbereich (bis zu 90 Mio. EUR) liegen, wobei zur Präzisierung dieses Schätzwertes vertiefte Analysen erforderlich sind. Die Kosten für eine Umsetzung gezielter Risikomanagementmaßnahmen zur Zurückhaltung des Materialaustrags dürften nach Schätzungen und je nach Umfang der Maßnahmen pro Kunststoffrasensystem bei 3.000 bis 10.000 EUR liegen.  e.: Der gemeinwohlorientierte Sport ist die größte zivilgesellschaftliche Bewegung in Deutschland und Europa. In Deutschland engagieren sich knapp acht Millionen Bürger freiwillig und ehrenamtlich im Sport. Das entspricht einer jährlichen Wertschöpfung und einem Wohlfahrtsgewinn allein in Deutschland von ca. 6,7 Milliarden Euro. Vergleichbare Zahlen lassen sich auch für die gesamte EU feststellen. In den EU-Mitgliedstaaten engagieren sich im Jahre 2010 zwischen 92 und 94 Millionen Menschen freiwillig für Ziele des Gemeinwohls, davon die meisten im Sport (ca. 35 bis 40 Prozent aller freiwillig Tätigen in der EU) (Europäische Kommission 2010).  Der Sport schafft ein strukturiertes, an die gesamte Bevölkerung gerichtetes und für alle offenes Bewegungs- und Sportangebot, durch das wichtige soziale und gesundheitsfördernde Funktionen in der Gesellschaft erfüllt werden. Sportvereine in Deutschland zählen zehn Millionen Mitgliedschaften im Kinder- und Jugendalter (DOSB-Bestandserhebung 2018), allein im DFB liegt diese Zahl bei 2,1 Millionen (DFB-Mitgliederstatistik 2018). Damit sind Sportvereine die wichtigste Anlaufstelle für Kinder und Jugendliche außerhalb der Schule und übernehmen unverzichtbare Aufgaben für die ganzheitliche Persönlichkeitsbildung junger Menschen. Dem Sport kommt eine wichtige Vorbild- und Lehrfunktion im Bereich der Integration und demokratischen Grundbildung zu. Für das herausragende gesellschaftliche Engagement des Sports spricht nicht zuletzt, dass die Sportvereine eng mit Schulen, Kindergärten, Unternehmen, Krankenkassen oder anderen öffentlichen Institutionen zusammenarbeiten. Um allen Bürgern den Zugang zum Sport zu ermöglichen, sind adäquate Sportstätten in ausreichender Anzahl Grundvoraussetzung. Ein für alle zugängliches und umfangreiches Sportangebot ist – vor allen Dingen in Großstädten und Ballungsgebieten – nur durch die Verfügbarkeit von ganzjährig nutzbaren Sportanlagen zu gewährleisten. Kunststoffrasenplätze spielen hierbei, insbesondere für den Fußball, eine wichtige Rolle, da sie eine intensivere Nutzung als Naturrasen- oder Tennenplätze erlauben. Allein mit Naturrasen- und Tennenplätzen lässt sich der derzeitige Trainings- und Spielbetrieb, insbesondere bei den Kinder- und Jugendmannschaften, nicht aufrechterhalten. Ein Kunststoffrasenplatz ersetzt etwa 2,5 Naturrasenplätze (DFBnet). Auf weniger als 10 Prozent der Naturrasenplätze finden an Wochenenden mehr als 2 Spiele statt. Bei Kunststoffrasenplätzen finden hingegen bei über 40 Prozent der Plätze mehr als 2 Spiele statt. Weniger als 10 Prozent der Naturrasenplätze wird an einem Wochenende mehr als 150 Minuten genutzt. Bei Kunstrasenplätzen werden hingegen etwa 35 Prozent an einem Wochenende mehr als 150 Minuten genutzt. 27.773 Spielstätten in Deutschland (ca. 70 Prozent) werden von Sportvereinen genutzt. Ein Drittel der Kunstrasenplätze werden von 2 oder mehr Vereinen mit alle ihren Jugend- und Seniorenmannschaften benutzt. Etwas über ein Drittel aller Naturrasenplätze wird von mehr als 5 Mannschaften bespielt. Bei Kunstrasenplätzen werden fast drei Viertel (72 Prozent) von mehr als 5 Mannschaften genutzt. Etwa 10 Prozent aller Naturrasenplätze wird von mehr als 10 Mannschaften bespielt. Bei Kunststoffrasenplätzen sind es ca. 41 Prozent der Plätze, die von mehr als 10 Mannschaften genutzt. Nur 1 Prozent aller Naturrasenplätze wird von mehr als 15 Mannschaften bespielt. Bei Kunstrasenplätzen beträgt der Anteil immerhin noch knapp 18 Prozent. Etwa 6 Prozent werden sogar von über 20 Mannschaften bespielt. Je größer die Vereinsgröße (insbesondere Anzahl der Mannschaften), desto höher ist der Anteil der Vereine, die auch eine Spielstätte vom Typ Kunstrasen haben.  Ein Verbot des Inverkehrbringens von Kunststoffgranulaten als Füllstoff in Kunststoffrasensystemen direkt bei Inkrafttreten der Beschränkung wäre daher unverhältnismäßig. Es würde zu hohen, unerwarteten Umstellungskosten und Mehrkosten für Vereine und Kommunen führen, wodurch dem gemeinwohlorientierten Sport Mittel entzogen würden. Bei fehlender Finanzierbarkeit dieser Mehrkosten ist zudem von einer Schließung vieler Sportplätzen auszugehen, wodurch das Sportangebot in Schulen und Vereinen stark leiden würde. Gerade auf Vereinsebene stellt ein solch außerordentlicher Kostenpunkt ein großes finanzielles Risiko dar, dass das sportliche und gesellschaftliche Gesamtangebot des Vereins gefährden kann. Eine Beschränkung ohne Übergangsfristen, die eine mittelfristige Umstellung und Kostenstreckung erlauben, würde das Breitensportangebot im Verbandsgebiet des Fußballverbandes Rheinland und sicher in ganz Deutschland sehr negativ beeinflussen.  Im Hinblick auf den Beschränkungsvorschlag der ECHA gemäß Anhang XV der REACH-Verordnung spricht sich der Fußballverbandes Rheinland daher für eine angemessene Übergangsfrist von mindestens sechs Jahren bis zu einem vollständigen Inverkehrbringungsverbot des Kunststoffgranulats zur Verwendung in neuen Kunststoffrasensystemen sowie für die Umstellung bestehender Flächen aus. |
| Germany, National NGO, Deutscher Olympischer Sportbund e.V. (DOSB, German Olympic Sports Confederation) | 2019/05/20  **Content:** | **Answer to specific info request 2:**  A) and B)  For the German Olympic Sport Confederation (DOSB) the currently available data is highly insufficient, as no data, risk analyses and impact assessments are available for the released quantities of infill or the effects of a restriction on the availability of sports facilities. The development of a better knowledge base should therefore be a first step, before ECHA imposes a direct restriction. Further scientific studies are needed in this complex area in order to close knowledge gaps and to develop more environmentally friendly materials for sports field construction and to enable an overall assessment of existing plastic turf systems based on sustainability criteria.  C)  Apart from the predominantly used plastic granulate, alternative filling materials exist for plastic turf systems, some of which are already used in the operation of sports facilities. In Germany, some plastic turf pitches are currently filled with sand and/or cork. There are also plastic turf systems that can be operated without an elastic filler. However, so far there are only few reliable studies on how these alternatives compare in terms of quality and cost (e.g. in terms of playability and service life).  In addition, it would have to be investigated whether and how the playability or the injury risk changes with the various alternative fillings. There is therefore an urgent need for further scientific expertise on the practical suitability of alternative, organic fillers and on the sports-specific suitability of plastic turf pitches that do not require fillers. Both a scientific impact assessment and the urgently needed development of alternative fillers by industry are central demands of the non-profit sports associations in Germany affected by the topic. They are of the opinion that the measures that would result in a ban on plastic granulate cannot be implemented in the short term and that alternatives can only be developed and made available in the medium to long term.  Targeted risk management measures can already significantly reduce the release of rubber granulate infill into the environment. Technical measures for the retention of material on site (e.g. gutter filters with sedimentation sections at drains, dirt trap mats, shoe brushes at the exit) and organisational measures for the operation of the sports fields (e.g. regular cleaning of the field edges, collecting sieves) can contribute to a significant reduction in the release of infill material.  D)  In Germany, there are approx. 5,000 artificial turf pitches registered for league and match use, as well as approx. 1,000 “mini-pitches. The German Olympic Sport Organisation (DOSB) is furthermore aware of a large number of other artificial turf pitches used for sports purposes. An exact quantification of the sports facilities affected by a possible restriction in Germany is currently not possible as a reliable data basis is lacking.  About 300 new artificial turf pitches are built in Germany every year, and 150 new artificial turf pitches are completely reconstructed. Regarding these pitches, a conversion to alternative infill may be necessary. Sports facility operators (municipalities or clubs) foresee funds for these construction and renovation measures of sports facilities, however, the financial investment needed will be significantly higher if a restriction is imposed and alternative infill materials have to be used. According to calculations by the German Football Federation (Deutscher Fußballbund, DFB), the annual additional costs throughout Germany would amount to a high single-digit million sum.  In addition to these already planned construction measures, a restriction would also affect all other artificial turf pitches filled with plastic granulate, as sports facility operators would no longer be able to acquire the required infill for regular re-filling. As a result, sports facility operators would incur costs for changing pitches that they had not budgeted for.  The costs of a restriction can currently not be quantified reliably due to a lack of knowledge about suitable alternative infill materials. On the basis of current data on the construction of artificial turf pitches, the total amount for the replacement of the infill material in the plastic turf systems in Germany is likely to be in the high double-digit million range.  E)  Not-for-profit sport is the largest civil society movement in Germany and Europe. It creates a structured sports offer that is open to the all of society and through which important social and health-promoting functions are fulfilled. Sports clubs in Germany have 10 million memberships in children and young people. Sports clubs are thus the most important contact point for children and young people outside school and assume indispensable tasks for the holistic personal development of young people.  In Germany, almost 8 million citizens are involved in sport on a voluntary basis. This makes sport the largest civil movement in Germany. Volunteers in sport perform a total of around 446 million voluntary, socially significant and unpaid working hours each year in a variety of functions. This amount of work corresponds to an annual added value and thus a welfare gain of approx. 6.7 billion euros in Germany alone. Comparable figures can also be established for the entire EU. In the EU Member States, between 92 and 94 million people volunteer for public welfare goals in 2010, most of them in sport (about 35 to 40 percent of all volunteers in the EU).  By involving large sections of the population in the daily work of sports clubs, it is possible to create and maintain a comprehensive, broad and accessible range of sports offers for all ages and levels of society. Sport also plays an important role in the field of integration and democratic basic education. In the German sports clubs alone, 2.6 million people with a migration background are socially integrated through sport.    Adequate sports facilities in sufficient numbers are a prerequisite for ensuring that all citizens have access to sport. For the organisation of sporting offers and the exercise of the social roles of sport, clubs are largely dependent on publicly financed sports facilities, but they also provide adequate sports facilities through their own investments. Attractively priced offers ensure that all sections of the population have access to sport. A comprehensive range of sports facilities accessible to all - especially in large cities and agglomerations - can only be guaranteed through the availability of sports facilities that can be used all year round. Artificial turf pitches play an important role in this regard, especially for football, as they allow more intensive use than turf or cinder pitches. It can be assumed that turf and cinder pitches alone will not be sufficient to maintain the current training and play facilities.  A restriction of plastic granulates as infill with the entry into force of the restriction would therefore be disproportionate, as it would lead to high, unforeseen conversion costs for clubs and municipalities, thereby depriving the not-for-profit sport of funds. If these additional costs cannot be financed, many sports fields are also in danger of being closed, which would severely affect the sporting offer available in schools and clubs. At club level in particular, such an extraordinary cost represents a major financial risk that could jeopardise the sports and social offerings of the club as a whole. A restriction without transition periods that would allow a medium-term changeover and cost extension, would therefore have a very negative impact on the not-for-profit sports offer in Germany.  German sports therefore advocates for an appropriate transitional period of at least six years until a complete ban on placing granular infill on the market for use in new plastic turf systems and the conversion of existing surfaces. Due to the high social relevance of sport, it is necessary to develop a common and step-by-step approach in order to appropriately balance the reduction of the discharge of microplastics with the need to practise sport among broad sections of the population. |
| Germany, National NGO,  Niedersächsischer Fußballverband e. V. | 2019/05/20  **Content:**  Environmental emissions;  Information on costs;  Other socio economic analysis (SEA) issues;  Transitional period | **Answer to specific info request 2:**  a.: Nach neueren Erkenntnissen werden zwischen 0,25 t/a und 5 t/a in Deutschland an Befüllungsgranulat pro Kunststoffrasenfläche verwendet (Fraunhofer 2018, S. 11). Das entspricht einer Gesamtmenge von ca. 7.500 bis 9.900 t/a.  b.: Nach dem aktuellen Forschungsstand besteht nach Kenntnis des DFB ein hohes Maß an Unsicherheit darüber, wie und in welchen Mengen das als Mikroplastik definierte Granulat auf Sportplätzen in die Umwelt freigesetzt wird. Nach den uns zur Verfügung stehen-den Informationen gibt es große Unterschiede bei der Einschätzung der Menge an Mikroplastiken, die in den einzelnen Mitgliedstaaten oder in der EU/EWR als Füllmaterial für Kunstrasen verwendet wird. Insbesondere Umfang und Methodologie der Forschung in diesem Bereich sind bisher noch wenig standardisiert und nachvollziehbar. Der DFB geht davon aus, dass der Anteil des Eintrags von Mikroplastik über Kunststoffrasenplätze je nach Mitgliedstaat ca. 1 bis 3 Prozent im Verhältnis zum Gesamteintrag beträgt. Demnach ist der Umwelteintrag verglichen mit anderen Hauptquellen relativ gering (Europäische Kommission 2018, ii)).  c.: Gezielte Risikomanagementmaßnahmen können die Freisetzung von Füllstoffen in die Umwelt bereits signifikant vermindern. Technische Maßnahmen zur Zurückhaltung eines Materialaustrags vor Ort (z.B. Rinnenfilter mit Sedimentationsstrecken an Abläufen, Schmutzfangmatten, Schuhbürsten am Ausgang) und organisatorische Maßnahmen beim Betrieb der Sportplätze (z.B. regelmäßige Reinigung der Spielfeldränder, Auffangsiebe) können zu einer starken Verringerung des Austrags von Mikroplastik beitragen.  Neben dem häufig genutzten Kunststoffgranulat existieren für Kunststoffrasensysteme alternative Füllstoffe, die in Teilen auch bereits beim Betrieb von Sportanlagen genutzt werden. So werden in Deutschland aktuell Kunststoffrasenplätze teilweise mit Sand und/oder Kork verfüllt. Zudem gibt es auch Kunststoffrasensysteme, die ohne elastischen Füllstoff betrieben werden können.  Es existieren bisher allerdings nur wenige belastbare Studien darüber, wie sich diese Alternativen qualitäts- und kostenmäßig (z.B. hinsichtlich der Bespielbarkeit und Lebensdauer) vergleichen lassen. Zudem müsste untersucht werden, ob und wie sich die Bespielbar-keit oder das Verletzungsrisiko der alternativ befüllten Kunststoffrasenflächen bei den verschiedenen Alternativfüllungen verändert (Plan Miljø Studie 2017). Es bedarf daher dringend weiterer wissenschaftlicher Expertise zur Praxistauglichkeit alternativer organischer Füllstoffe und zur sportartspezifischen Eignung von Kunststoffrasenplätzen, die ohne Füllstoffe auskommen. Sowohl eine wissenschaftliche Folgenabschätzung als auch die dringend erforderliche Entwicklung alternativer Füllstoffe durch die Industrie sind eine zentrale Forderung der von der Thematik betroffenen Sportverbände in Deutschland. Sie vertreten die Meinung, dass die Maßnahmen, die ein Verbot des Kunststoffgranulats verursachen würden, nicht kurzfristig umsetzbar sind und Alternativen nur mittel- bis langfristig erarbeitet und bereitgestellt werden können.  d.: In Deutschland gibt es ca. 5.000 für den Fußballspielbetrieb gemeldete Kunststoffrasenplätze (DFBnet), sowie ca. 1.000 DFB-Minispielfelder. Jährlich werden in Deutschland ca. 300 Kunststoffrasenplätze neu gebaut, sowie 150 Kunststoffrasenplätze von Grund auf erneuert. Hinsichtlich der bestehenden Plätze dürfte eine Umstellung auf alternative Füllstoffe notwendig sein. Hierfür halten die Sportanlagenbetreiber (Kommunen oder Vereine) Mittel für Sportstättenbau und -sanierung vor, die bei einem vollständigen Ver-bot und einer Verwendung alternativer Füllstoffe deutlich höher ausfallen würden. Laut eigener Berechnungen belaufen sich die jährlichen Mehrkosten deutschlandweit auf einen hohen einstelligen Millionenbetrag. Die insgesamt zu erwartenden Kosten eines Verbotes können aufgrund fehlender Kenntnisse über geeignete alternative Füllstoffe (Geeignetheit, Verfügbarkeit) derzeit nicht seriös beziffert werden. Auf Grundlage aktueller Daten zum Bau von Kunststoffrasenplätzen dürfte der Gesamtbetrag für den Austausch des Füllstoffes der Kunststoffrasensysteme im hohen zweistelligen Millionenbereich (bis zu 90 Mio. EUR) liegen, wobei zur Präzisierung dieses Schätzwertes vertiefte Analysen erforderlich sind. Die Kosten für eine Umsetzung gezielter Risikomanagementmaßnahmen zur Zurückhaltung des Materialaustrags dürften nach Schätzungen und je nach Umfang der Maßnahmen pro Kunststoffrasensystem bei 3.000 bis 10.000 EUR liegen.  e.: Der gemeinwohlorientierte Sport ist die größte zivilgesellschaftliche Bewegung in Deutschland und Europa. In Deutschland engagieren sich knapp acht Millionen Bürger freiwillig und ehrenamtlich im Sport. Das entspricht einer jährlichen Wertschöpfung und einem Wohlfahrtsgewinn allein in Deutschland von ca. 6,7 Milliarden Euro. Vergleichbare Zahlen lassen sich auch für die gesamte EU feststellen. In den EU-Mitgliedstaaten engagieren sich im Jahre 2010 zwischen 92 und 94 Millionen Menschen freiwillig für Ziele des Gemeinwohls, davon die meisten im Sport (ca. 35 bis 40 Prozent aller freiwillig Tätigen in der EU) (Europäische Kommission 2010).  Der Sport schafft ein strukturiertes, an die gesamte Bevölkerung gerichtetes und für alle offenes Bewegungs- und Sportangebot, durch das wichtige soziale und gesundheitsfördernde Funktionen in der Gesellschaft erfüllt werden. Sportvereine in Deutschland zählen zehn Millionen Mitgliedschaften im Kinder- und Jugendalter (DOSB-Bestandserhebung 2018), allein im DFB liegt diese Zahl bei 2,1 Millionen (DFB-Mitgliederstatistik 2018). Damit sind Sportvereine die wichtigste Anlaufstelle für Kinder und Jugendliche außerhalb der Schule und übernehmen unverzichtbare Aufgaben für die ganzheitliche Persönlichkeitsbildung junger Menschen. Dem Sport kommt eine wichtige Vorbild- und Lehrfunktion im Bereich der Integration und demokratischen Grundbildung zu. Für das herausragende gesellschaftliche Engagement des Sports spricht nicht zuletzt, dass die Sportvereine eng mit Schulen, Kindergärten, Unternehmen, Krankenkassen oder anderen öffentlichen Institutionen zusammenarbeiten. Um allen Bürgern den Zugang zum Sport zu ermöglichen, sind adäquate Sportstätten in ausreichender Anzahl Grundvoraussetzung. Ein für alle zugängliches und umfangreiches Sportangebot ist – vor allen Dingen in Großstädten und Ballungsgebieten – nur durch die Verfügbarkeit von ganzjährig nutzbaren Sportanlagen zu gewährleisten. Kunststoffrasenplätze spielen hierbei, insbesondere für den Fußball, eine wichtige Rolle, da sie eine intensivere Nutzung als Naturrasen- oder Tennenplätze erlauben. Allein mit Naturrasen- und Tennenplätzen lässt sich der derzeitige Trainings- und Spielbetrieb, insbesondere bei den Kinder- und Jugendmannschaften, nicht aufrechterhalten. Ein Kunststoffrasenplatz ersetzt etwa 2,5 Naturrasenplätze (DFBnet). Auf weniger als 10 Prozent der Naturrasenplätze finden an Wochenenden mehr als 2 Spiele statt. Bei Kunststoffrasenplätzen finden hingegen bei über 40 Prozent der Plätze mehr als 2 Spiele statt. Weniger als 10 Prozent der Naturrasenplätze wird an einem Wochenende mehr als 150 Minuten genutzt. Bei Kunstrasenplätzen werden hingegen etwa 35 Prozent an einem Wochenende mehr als 150 Minuten genutzt. 27.773 Spielstätten in Deutschland (ca. 70 Prozent) werden von Sportvereinen genutzt. Ein Drittel der Kunstrasenplätze werden von 2 oder mehr Vereinen mit alle ihren Jugend- und Seniorenmannschaften benutzt. Etwas über ein Drittel aller Naturrasenplätze wird von mehr als 5 Mannschaften bespielt. Bei Kunstrasenplätzen werden fast drei Viertel (72 Prozent) von mehr als 5 Mannschaften genutzt. Etwa 10 Prozent aller Naturrasenplätze wird von mehr als 10 Mannschaften bespielt. Bei Kunststoffrasenplätzen sind es ca. 41 Prozent der Plätze, die von mehr als 10 Mannschaften genutzt. Nur 1 Prozent aller Naturrasenplätze wird von mehr als 15 Mannschaften bespielt. Bei Kunstrasenplätzen beträgt der Anteil immerhin noch knapp 18 Prozent. Etwa 6 Prozent werden sogar von über 20 Mannschaften bespielt. Je größer die Vereinsgröße (insbesondere Anzahl der Mannschaften), desto höher ist der Anteil der Vereine, die auch eine Spielstätte vom Typ Kunstrasen haben.  Ein Verbot des Inverkehrbringens von Kunststoffgranulaten als Füllstoff in Kunststoffrasensystemen direkt bei Inkrafttreten der Beschränkung wäre daher unverhältnismäßig. Es würde zu hohen, unerwarteten Umstellungskosten und Mehrkosten für Vereine und Kommunen führen, wodurch dem gemeinwohlorientierten Sport Mittel entzogen würden. Bei fehlender Finanzierbarkeit dieser Mehrkosten ist zudem von einer Schließung vieler Sportplätzen auszugehen, wodurch das Sportangebot in Schulen und Vereinen stark leiden würde. Gerade auf Vereinsebene stellt ein solch außerordentlicher Kosten-punkt ein großes finanzielles Risiko dar, dass das sportliche und gesellschaftliche Gesamtangebot des Vereins gefährden kann. Eine Beschränkung ohne Übergangsfristen, die eine mittelfristige Umstellung und Kostenstreckung erlauben, würde das Breitensportangebot in Deutschland sehr negativ beeinflussen.  Im Verbandsgebiet des Niedersächsischen Fußballverbandes e.V. (NFV) mit mehr als 2.600 Mitgliedsvereinen befinden sich 193 Kunstrasenplätze im Spiel- und Trainingsbetrieb. Daneben nutzten unsere Mitgliedsvereine für den ergänzenden Trainings- und Freizeitspielbetrieb mehr als 100 Kunstrasen-Minispielfelder. Sofern diese Spiel- Trainings- und Freizeitsportflächen derart kurzfristig nicht weiter zur Verfügung stehen sollten, stellt dies für das gesamte Sportangebot unserer Mitgliedsvereine eine unverhältnismäßige Einschränkung dar, die in der angedachten Kürze auch nicht zu kompensieren ist.  Im Hinblick auf den Beschränkungsvorschlag der ECHA gemäß Anhang XV der REACH-Verordnung spricht sich der DFB und der NFV daher für eine angemessene Übergangsfrist von mindestens sechs Jahren bis zu einem vollständigen Inverkehrbringungsverbot des Kunststoffgranulats zur Verwendung in neuen Kunststoffrasensystemen sowie für die Umstellung bestehender Flächen aus. |
| Germany, National NGO, Football Federation Saxony-Anhalt | 2019/05/20  **Content:**  Scope or restriction option analysis;  Environmental emissions;  Information on costs;  Other socio economic analysis (SEA) issues;  Transitional period | **Answer to specific info request 2:**  a.: Nach neueren Erkenntnissen werden zwischen 0,25 t/a und 5 t/a in Deutschland an Befüllungsgranulat pro Kunststoffrasenfläche verwendet (Fraunhofer 2018, S. 11). Das entspricht einer Gesamtmenge von ca. 7.500 bis 9.900 t/a.  b.: Nach dem aktuellen Forschungsstand besteht nach Kenntnis des DFB ein hohes Maß an Unsicherheit darüber, wie und in welchen Mengen das als Mikroplastik definierte Granulat auf Sportplätzen in die Umwelt freigesetzt wird. Nach den uns zur Verfügung stehen-den Informationen gibt es große Unterschiede bei der Einschätzung der Menge an Mikroplastiken, die in den einzelnen Mitgliedstaaten oder in der EU/EWR als Füllmaterial für Kunstrasen verwendet wird. Insbesondere Umfang und Methodologie der Forschung in diesem Bereich sind bisher noch wenig standardisiert und nachvollziehbar. Der DFB geht davon aus, dass der Anteil des Eintrags von Mikroplastik über Kunststoffrasenplätze je nach Mitgliedstaat ca. 1 bis 3 Prozent im Verhältnis zum Gesamteintrag beträgt. Demnach ist der Umwelteintrag verglichen mit anderen Hauptquellen relativ gering (Europäische Kommission 2018, ii)).  c.: Gezielte Risikomanagementmaßnahmen können die Freisetzung von Füllstoffen in die Umwelt bereits signifikant vermindern. Technische Maßnahmen zur Zurückhaltung eines Materialaustrags vor Ort (z.B. Rinnenfilter mit Sedimentationsstrecken an Abläufen, Schmutzfangmatten, Schuhbürsten am Ausgang) und organisatorische Maßnahmen beim Betrieb der Sportplätze (z.B. regelmäßige Reinigung der Spielfeldränder, Auffangsiebe) können zu einer starken Verringerung des Austrags von Mikroplastik beitragen.  Neben dem häufig genutzten Kunststoffgranulat existieren für Kunststoffrasensysteme alternative Füllstoffe, die in Teilen auch bereits beim Betrieb von Sportanlagen genutzt werden. So werden in Deutschland aktuell Kunststoffrasenplätze teilweise mit Sand und/oder Kork verfüllt. Zudem gibt es auch Kunststoffrasensysteme, die ohne elastischen Füllstoff betrieben werden können.  Es existieren bisher allerdings nur wenige belastbare Studien darüber, wie sich diese Alternativen qualitäts- und kostenmäßig (z.B. hinsichtlich der Bespielbarkeit und Lebensdauer) vergleichen lassen. Zudem müsste untersucht werden, ob und wie sich die Bespielbar-keit oder das Verletzungsrisiko der alternativ befüllten Kunststoffrasenflächen bei den verschiedenen Alternativfüllungen verändert (Plan Miljø Studie 2017). Es bedarf daher dringend weiterer wissenschaftlicher Expertise zur Praxistauglichkeit alternativer organischer Füllstoffe und zur sportartspezifischen Eignung von Kunststoffrasenplätzen, die ohne Füllstoffe auskommen. Sowohl eine wissenschaftliche Folgenabschätzung als auch die dringend erforderliche Entwicklung alternativer Füllstoffe durch die Industrie sind eine zentrale Forderung der von der Thematik betroffenen Sportverbände in Deutschland. Sie vertreten die Meinung, dass die Maßnahmen, die ein Verbot des Kunststoffgranulats verursachen würden, nicht kurzfristig umsetzbar sind und Alternativen nur mittel- bis langfristig erarbeitet und bereitgestellt werden können.  d.: In Deutschland gibt es ca. 5.000 für den Fußballspielbetrieb gemeldete Kunststoffrasenplätze (DFBnet), sowie ca. 1.000 DFB-Minispielfelder. Jährlich werden in Deutschland ca. 300 Kunststoffrasenplätze neu gebaut, sowie 150 Kunststoffrasenplätze von Grund auf erneuert. Hinsichtlich der bestehenden Plätze dürfte eine Umstellung auf alternative Füllstoffe notwendig sein. Hierfür halten die Sportanlagenbetreiber (Kommunen oder Vereine) Mittel für Sportstättenbau und -sanierung vor, die bei einem vollständigen Ver-bot und einer Verwendung alternativer Füllstoffe deutlich höher ausfallen würden. Laut eigener Berechnungen belaufen sich die jährlichen Mehrkosten deutschlandweit auf einen hohen einstelligen Millionenbetrag. Die insgesamt zu erwartenden Kosten eines Verbotes können aufgrund fehlender Kenntnisse über geeignete alternative Füllstoffe (Geeignetheit, Verfügbarkeit) derzeit nicht seriös beziffert werden. Auf Grundlage aktueller Daten zum Bau von Kunststoffrasenplätzen dürfte der Gesamtbetrag für den Austausch des Füllstoffes der Kunststoffrasensysteme im hohen zweistelligen Millionenbereich (bis zu 90 Mio. EUR) liegen, wobei zur Präzisierung dieses Schätzwertes vertiefte Analysen erforderlich sind. Die Kosten für eine Umsetzung gezielter Risikomanagementmaßnahmen zur Zurückhaltung des Materialaustrags dürften nach Schätzungen und je nach Umfang der Maßnahmen pro Kunststoffrasensystem bei 3.000 bis 10.000 EUR liegen.  e.: Der gemeinwohlorientierte Sport ist die größte zivilgesellschaftliche Bewegung in Deutschland und Europa. Im Fußballverband Sachsen-Anhalt engagieren sich knapp 19.000 Bürger freiwillig und ehrenamtlich im organisierten Fußball für rund 91.000 Mitglieder in über 790 Sportvereinen.  Der Sport schafft ein strukturiertes, an die gesamte Bevölkerung gerichtetes und für alle offenes Bewegungs- und Sportangebot, durch das wichtige soziale und gesundheitsfördernde Funktionen in der Gesellschaft erfüllt werden. Fußballvereine in Sachsen-Anhalt zählen rund 41.000 Mitgliedschaften im Kinder- und Jugendalter. Damit sind die Fußballvereine die wichtigste Anlaufstelle für Kinder und Jugendliche außerhalb der Schule und übernehmen unverzichtbare Aufgaben für die ganzheitliche Persönlichkeitsbildung junger Menschen. Dem Sport kommt eine wichtige Vorbild- und Lehrfunktion im Bereich der Integration und demokratischen Grundbildung zu. Für das herausragende gesellschaftliche Engagement des Sports spricht nicht zuletzt, dass die Sportvereine eng mit Schulen, Kindergärten, Unternehmen, Krankenkassen oder anderen öffentlichen Institutionen zusammenarbeiten. Um allen Bürgern den Zugang zum Sport zu ermöglichen, sind adäquate Sportstätten in ausreichender Anzahl Grundvoraussetzung. Ein für alle zugängliches und umfangreiches Sportangebot ist nur durch die Verfügbarkeit von ganzjährig nutzbaren Sportanlagen zu gewährleisten. Kunststoffrasenplätze spielen hierbei, insbesondere für den Fußball, eine wichtige Rolle, da sie eine intensivere Nutzung als Naturrasen- oder Tennenplätze erlauben. Allein mit Naturrasen- und Tennenplätzen lässt sich der derzeitige Trainings- und Spielbetrieb, insbesondere bei den Kinder- und Jugendmannschaften, nicht aufrechterhalten.  Im Fußball in Sachsen-Anhalt gibt es rund 81 Sportanlagen (zum Teil mit mehreren Spielfeldern) an denen für den Fußball und (meist überlappend) Hockey Kunststoffrasenplätze vorhanden sind. Insgesamt nutzen Fußball- und die 5 Hockeyvereine insgesamt knapp 650 Sportplätze. Die circa 13 % Kunststoffrasenplätze befinden sich schwerpunktmäßig in den beiden Großstädten Magdeburg und Halle (Saale). Hier wäre ein Trainings- und Wettkampfbetrieb etlicher Vereine ohne diese Anlagen undenkbar. An diesen Standorten nutzen die Anlagen nicht nur mehrere Fußballvereine gleichzeitig sondern teilen sich die Sportarten Fußball und Hockey auch den Sportplatz.  Ungeachtet der Tatsache, dass ohne diese Plätze der Trainings- und Wettkampfbedarf nicht auch nur annährend bedient werden könnte, wäre es ebenso unvorstellbar diese Stunden auf neu zu errichtende Naturrasenplätze umzulagern, da die hohe Stundenzahl der Nutzung auf dem Naturbelag nicht möglich ist.  Ein Verbot des Inverkehrbringens von Kunststoffgranulaten als Füllstoff in Kunststoffrasensystemen direkt bei Inkrafttreten der Beschränkung wäre daher unverhältnismäßig. Es würde zu hohen, unerwarteten Umstellungskosten und Mehrkosten für Vereine und Kommunen führen, wodurch dem gemeinwohlorientierten Sport Mittel entzogen würden. Bei fehlender Finanzierbarkeit dieser Mehrkosten ist zudem von einer Schließung vieler Sportplätzen auszugehen, wodurch das Sportangebot in Schulen und Vereinen stark leiden würde. Gerade auf Vereinsebene stellt ein solch außerordentlicher Kostenpunkt ein großes finanzielles Risiko dar, dass das sportliche und gesellschaftliche Gesamtangebot des Vereins gefährden würde. Eine Beschränkung ohne Übergangsfristen, die eine mittelfristige Umstellung und Kostenstreckung erlauben, würde das Sportangebot in Sachsen-Anhalt sehr negativ beeinflussen.  Im Hinblick auf den Beschränkungsvorschlag der ECHA gemäß Anhang XV der REACH-Verordnung spricht sich der LandesSportBund Sachsen-Anhalt daher für eine angemessene Übergangsfrist von mindestens sechs Jahren bis zu einem vollständigen Inverkehrbringungsverbot des Kunststoffgranulats zur Verwendung in neuen Kunststoffrasensystemen sowie für die Umstellung bestehender Flächen aus. |
| Germany, National NGO,  Fußball und Leichtathletik-Verband Westfalen e.V. | 2019/05/20  **Content:**  Scope or restriction option analysis;  Environmental emissions;  Information on costs;  Other socio economic analysis (SEA) issues;  Transitional period | **Answer to specific info request 2:**  a.: Nach neueren Erkenntnissen werden zwischen 0,25 t/a und 5 t/a in Deutschland an Befüllungsgranulat pro Kunststoffrasenfläche verwendet (Fraunhofer 2018, S. 11). Das entspricht einer Gesamtmenge von ca. 7.500 bis 9.900 t/a.  b.: Nach dem aktuellen Forschungsstand besteht nach Kenntnis des DFB ein hohes Maß an Unsicherheit darüber, wie und in welchen Mengen das als Mikroplastik definierte Granulat auf Sportplätzen in die Umwelt freigesetzt wird. Nach den uns zur Verfügung stehen-den Informationen gibt es große Unterschiede bei der Einschätzung der Menge an Mikroplastiken, die in den einzelnen Mitgliedstaaten oder in der EU/EWR als Füllmaterial für Kunstrasen verwendet wird. Insbesondere Umfang und Methodologie der Forschung in diesem Bereich sind bisher noch wenig standardisiert und nachvollziehbar. Der DFB geht davon aus, dass der Anteil des Eintrags von Mikroplastik über Kunststoffrasenplätze je nach Mitgliedstaat ca. 1 bis 3 Prozent im Verhältnis zum Gesamteintrag beträgt. Demnach ist der Umwelteintrag verglichen mit anderen Hauptquellen relativ gering (Europäische Kommission 2018, ii)).  c.: Gezielte Risikomanagementmaßnahmen können die Freisetzung von Füllstoffen in die Umwelt bereits signifikant vermindern. Technische Maßnahmen zur Zurückhaltung eines Materialaustrags vor Ort (z.B. Rinnenfilter mit Sedimentationsstrecken an Abläufen, Schmutzfangmatten, Schuhbürsten am Ausgang) und organisatorische Maßnahmen beim Betrieb der Sportplätze (z.B. regelmäßige Reinigung der Spielfeldränder, Auffangsiebe) können zu einer starken Verringerung des Austrags von Mikroplastik beitragen.  Neben dem häufig genutzten Kunststoffgranulat existieren für Kunststoffrasensysteme alternative Füllstoffe, die in Teilen auch bereits beim Betrieb von Sportanlagen genutzt werden. So werden in Deutschland aktuell Kunststoffrasenplätze teilweise mit Sand und/oder Kork verfüllt. Zudem gibt es auch Kunststoffrasensysteme, die ohne elastischen Füllstoff betrieben werden können.  Es existieren bisher allerdings nur wenige belastbare Studien darüber, wie sich diese Alternativen qualitäts- und kostenmäßig (z.B. hinsichtlich der Bespielbarkeit und Lebensdauer) vergleichen lassen. Zudem müsste untersucht werden, ob und wie sich die Bespielbar-keit oder das Verletzungsrisiko der alternativ befüllten Kunststoffrasenflächen bei den verschiedenen Alternativfüllungen verändert (Plan Miljø Studie 2017). Es bedarf daher dringend weiterer wissenschaftlicher Expertise zur Praxistauglichkeit alternativer organischer Füllstoffe und zur sportartspezifischen Eignung von Kunststoffrasenplätzen, die ohne Füllstoffe auskommen. Sowohl eine wissenschaftliche Folgenabschätzung als auch die dringend erforderliche Entwicklung alternativer Füllstoffe durch die Industrie sind eine zentrale Forderung der von der Thematik betroffenen Sportverbände in Deutschland. Sie vertreten die Meinung, dass die Maßnahmen, die ein Verbot des Kunststoffgranulats verursachen würden, nicht kurzfristig umsetzbar sind und Alternativen nur mittel- bis langfristig erarbeitet und bereitgestellt werden können.  d.: In Deutschland gibt es ca. 5.000 für den Fußballspielbetrieb gemeldete Kunststoffrasenplätze (DFBnet), sowie ca. 1.000 DFB-Minispielfelder. Jährlich werden in Deutschland ca. 300 Kunststoffrasenplätze neu gebaut, sowie 150 Kunststoffrasenplätze von Grund auf erneuert. Hinsichtlich der bestehenden Plätze dürfte eine Umstellung auf alternative Füllstoffe notwendig sein. Hierfür halten die Sportanlagenbetreiber (Kommunen oder Vereine) Mittel für Sportstättenbau und -sanierung vor, die bei einem vollständigen Ver-bot und einer Verwendung alternativer Füllstoffe deutlich höher ausfallen würden. Laut eigener Berechnungen belaufen sich die jährlichen Mehrkosten deutschlandweit auf einen hohen einstelligen Millionenbetrag. Die insgesamt zu erwartenden Kosten eines Verbotes können aufgrund fehlender Kenntnisse über geeignete alternative Füllstoffe (Geeignetheit, Verfügbarkeit) derzeit nicht seriös beziffert werden. Auf Grundlage aktueller Daten zum Bau von Kunststoffrasenplätzen dürfte der Gesamtbetrag für den Austausch des Füllstoffes der Kunststoffrasensysteme im hohen zweistelligen Millionenbereich (bis zu 90 Mio. EUR) liegen, wobei zur Präzisierung dieses Schätzwertes vertiefte Analysen erforderlich sind. Die Kosten für eine Umsetzung gezielter Risikomanagementmaßnahmen zur Zurückhaltung des Materialaustrags dürften nach Schätzungen und je nach Umfang der Maßnahmen pro Kunststoffrasensystem bei 3.000 bis 10.000 EUR liegen.  e.: Der gemeinwohlorientierte Sport ist die größte zivilgesellschaftliche Bewegung in Deutschland und Europa. In Deutschland engagieren sich knapp acht Millionen Bürger freiwillig und ehrenamtlich im Sport. Das entspricht einer jährlichen Wertschöpfung und einem Wohlfahrtsgewinn allein in Deutschland von ca. 6,7 Milliarden Euro. Vergleichbare Zahlen lassen sich auch für die gesamte EU feststellen. In den EU-Mitgliedstaaten engagieren sich im Jahre 2010 zwischen 92 und 94 Millionen Menschen freiwillig für Ziele des Gemeinwohls, davon die meisten im Sport (ca. 35 bis 40 Prozent aller freiwillig Tätigen in der EU) (Europäische Kommission 2010).  Der Sport schafft ein strukturiertes, an die gesamte Bevölkerung gerichtetes und für alle offenes Bewegungs- und Sportangebot, durch das wichtige soziale und gesundheitsfördernde Funktionen in der Gesellschaft erfüllt werden. Sportvereine in Deutschland zählen zehn Millionen Mitgliedschaften im Kinder- und Jugendalter (DOSB-Bestandserhebung 2018), allein im DFB liegt diese Zahl bei 2,1 Millionen (DFB-Mitgliederstatistik 2018). Damit sind Sportvereine die wichtigste Anlaufstelle für Kinder und Jugendliche außerhalb der Schule und übernehmen unverzichtbare Aufgaben für die ganzheitliche Persönlichkeitsbildung junger Menschen. Dem Sport kommt eine wichtige Vorbild- und Lehrfunktion im Bereich der Integration und demokratischen Grundbildung zu. Für das herausragende gesellschaftliche Engagement des Sports spricht nicht zuletzt, dass die Sportvereine eng mit Schulen, Kindergärten, Unternehmen, Krankenkassen oder anderen öffentlichen Institutionen zusammenarbeiten. Um allen Bürgern den Zugang zum Sport zu ermöglichen, sind adäquate Sportstätten in ausreichender Anzahl Grundvoraussetzung. Ein für alle zugängliches und umfangreiches Sportangebot ist – vor allen Dingen in Großstädten und Ballungsgebieten – nur durch die Verfügbarkeit von ganzjährig nutzbaren Sportanlagen zu gewährleisten. Kunststoffrasenplätze spielen hierbei, insbesondere für den Fußball, eine wichtige Rolle, da sie eine intensivere Nutzung als Naturrasen- oder Tennenplätze erlauben. Allein mit Naturrasen- und Tennenplätzen lässt sich der derzeitige Trainings- und Spielbetrieb, insbesondere bei den Kinder- und Jugendmannschaften, nicht aufrechterhalten. Ein Kunststoffrasenplatz ersetzt etwa 2,5 Naturrasenplätze (DFBnet). Auf weniger als 10 Prozent der Naturrasenplätze finden an Wochenenden mehr als 2 Spiele statt. Bei Kunststoffrasenplätzen finden hingegen bei über 40 Prozent der Plätze mehr als 2 Spiele statt. Weniger als 10 Prozent der Naturrasenplätze wird an einem Wochenende mehr als 150 Minuten genutzt. Bei Kunstrasenplätzen werden hingegen etwa 35 Prozent an einem Wochenende mehr als 150 Minuten genutzt. 27.773 Spielstätten in Deutschland (ca. 70 Prozent) werden von Sportvereinen genutzt. Ein Drittel der Kunstrasenplätze werden von 2 oder mehr Vereinen mit alle ihren Jugend- und Seniorenmannschaften benutzt. Etwas über ein Drittel aller Naturrasenplätze wird von mehr als 5 Mannschaften bespielt. Bei Kunstrasenplätzen werden fast drei Viertel (72 Prozent) von mehr als 5 Mannschaften genutzt. Etwa 10 Prozent aller Naturrasenplätze wird von mehr als 10 Mannschaften bespielt. Bei Kunststoffrasenplätzen sind es ca. 41 Prozent der Plätze, die von mehr als 10 Mannschaften genutzt. Nur 1 Prozent aller Naturrasenplätze wird von mehr als 15 Mannschaften bespielt. Bei Kunstrasenplätzen beträgt der Anteil immerhin noch knapp 18 Prozent. Etwa 6 Prozent werden sogar von über 20 Mannschaften bespielt. Je größer die Vereinsgröße (insbesondere Anzahl der Mannschaften), desto höher ist der Anteil der Vereine, die auch eine Spielstätte vom Typ Kunstrasen haben.  Ein Verbot des Inverkehrbringens von Kunststoffgranulaten als Füllstoff in Kunststoffrasensystemen direkt bei Inkrafttreten der Beschränkung wäre daher unverhältnismäßig. Es würde zu hohen, unerwarteten Umstellungskosten und Mehrkosten für Vereine und Kommunen führen, wodurch dem gemeinwohlorientierten Sport Mittel entzogen würden. Bei fehlender Finanzierbarkeit dieser Mehrkosten ist zudem von einer Schließung vieler Sportplätzen auszugehen, wodurch das Sportangebot in Schulen und Vereinen stark leiden würde. Gerade auf Vereinsebene stellt ein solch außerordentlicher Kosten-punkt ein großes finanzielles Risiko dar, dass das sportliche und gesellschaftliche Gesamtangebot des Vereins gefährden kann. Eine Beschränkung ohne Übergangsfristen, die eine mittelfristige Umstellung und Kostenstreckung erlauben, würde das Breitensportangebot in Deutschland sehr negativ beeinflussen.  Im Hinblick auf den Beschränkungsvorschlag der ECHA gemäß Anhang XV der REACH-Verordnung spricht sich der DFB daher für eine angemessene Übergangsfrist von mindestens sechs Jahren bis zu einem vollständigen Inverkehrbringungsverbot des Kunststoffgranulats zur Verwendung in neuen Kunststoffrasensystemen sowie für die Umstellung bestehender Flächen aus. |

| Breast Cancer UK (National NGO, United Kingdom) | 2019/05/20  **Content:**  Hazard or exposure;  Environmental emissions | **Comment:**  Breast Cancer UK is a charity which aims to prevent breast cancer by promoting a healthy lifestyle and reducing public exposure to carcinogenic and other hazardous chemicals in the environment. In particular we are concerned about the potential role of exposures to environmental chemicals in increasing breast cancer risk. We consider microplastics to be potentially harmful to human health and the environment. We believe their presence in the environment may increase breast cancer risk, due to the potential for these particles to release harmful additives and to accumulate and release other substances of concern.  We welcome ECHA’s Annex XV proposal to restrict intentionally added microplastics in consumer and professional products and are grateful for the opportunity to respond to the proposal. Breast Cancer UK supports restricting the use of intentionally added microplastic particles in products of any kind.  We are especially concerned about chemical “additives” present in microplastics (e.g. plasticisers and compounds used in manufacture such as bisphenols), and the potential for microplastics to act as “vectors” for environmental pollutants; these substances may be transferred to marine and other organisms, following ingestion of microplastics (1, 2). Marine species include those regularly consumed by humans, such as mussels and oysters, as well as endangered species such as humpback dolphins (3). Studies have found common persistent organic pollutants can be up to 10 million times higher in plastic pellets than in sea water (4). As well as being potentially detrimental to the health of marine organisms and birds, microplastics and associated environmental pollutants have the potential to be passed up the food chain. A recent study by the European Commission’s Joint Research Centre (5) highlights the presence of micro and nano-plastics in animals and food products and concludes “There is a growing concern about the impact of human activities on the whole life chain, and there is a legitimate concern that the smaller plastic fraction, through bioaccumulation and trophic transfer, may ultimately contaminate the human population”.  Many chemical additives that leach from microplastics, such as bisphenols, heavy metals and phthalates, are endocrine disrupting chemicals (EDCs), which can affect the function of the hormone system. In particular EDCs which act as oestrogen mimics are associated with increased breast cancer risk (6). Endocrine disrupting chemicals may exert their effects at very low doses, and it is becoming increasingly apparent that environmental exposures to mixtures of such chemicals may be especially harmful (7).  Studies have shown microplastics may enhance toxicity (as well as bioaccumulation) of heavy metals in fish. For example, the presence of microplastics enhanced the toxicity and bioaccumulation of cadmium in zebrafish (8), causing oxidative damage and inflammation. Environmental exposure to cadmium (which is an EDC), is a risk factor for breast cancer (9).  As stated in ECHA’s background document, intentionally added microplastics can be released into the environment during the use of these products (typically via wastewater), potentially contributing to environmental litter and leading to concerns that their use may pose a risk to the environment and/or human health. Recent studies show that microplastics are not removed fully from wastewater treatment plants (WWTPs). The activated sludge process (the most common type of sewage treatment used globally) has a retention capacity of up to around 98-99% (10, 11), with most of the microplastics remaining within the activated sludge solids. Despite this, WWTPs remain point sources for microplastics (and nanoplastics) discharge, due to the high volume of effluent that is released constantly.  Microplastics may have a negative impact on the activated sludge treatment process itself; a recent study found respiration of activated sludge flocs was acutely inhibited by the presence of polystyrene nanoplastics (12), due to a change in composition of the extracellular polymeric substance (EPS) that surrounds activated sludge microorganisms and is integral to floc formation. Such changes will affect sludge settling and reduced respiration will affect the ability of activated sludge microorganisms to biodegrade pollutants. Another concern is the presence of microplastics (containing environmental pollutants) in the activated sludge solids that are removed and used commonly as land fertiliser (following appropriate treatment). One study which examined the fate of polyethylene microbeads from cosmetics using a laboratory scale bioreactor run to simulate an activated sludge WWTP found approximately half the microbeads were captured in the activated sludge (13). Other studies (cited above) suggest that most of the microplastics that enter an activated sludge WWTP will end up in the excess sludge solids.  Reducing significantly microlitter pollution in marine, freshwater and terrestrial environments should be a priority, given the potential harm this type of pollution causes. Although we appreciate microplastics may also arise from degradation of macroplastics, a ban on the use of added microplastics in products of any type would be one step towards reducing environmental pollution.  1. Carbery, M. et al. (2018). Environment International 115:400-409  2. Rochman, C. M. et al. (2013). Scientific Reports 3: 3263.  3. Zhu, J. et al. (2019). Science of the total Environment 659: 649-654.  4. Karlsson, T. M. et al. (2018). Marine Pollution Bulletin 129: 52-60  5. Toussain, B. et al. (2019). Food Additives & Contaminants Part A 36(5): 639-673.  6. Brody et al. (2018). Environmental research 160: 152-182  7. Pastor-Barriuso, R. et al. (2016). Environmental Health Perspectives 124 (10): 1575-1582  8. Lu, K et al. (2018). Chemosphere 202: 514-520.  9. Mezynska, M. and Brzóska, M. M. (2018). Environmental Science and Pollution Research 25: 3211–3232.  10. Lares et al. (2018). Water Research 133: 236-246.  11. Talvitie, J. et al. (2018). Water Research 109: 164-172.  12. Feng, L.-J. et al. (2018). Environmental Pollution 238: 859-865.  13. Kalčíková, G. et al. (2018). Chemosphere 188: 25-31.  Dr Margaret Wexler  Head of Science, Breast Cancer UK  margaret.wexler@breastcanceruk.org.uk |
| --- | --- | --- |
| Swedish Association of Local Authorities and Regions  (National NGO | 2019/05/20  **Content:**  Scope or restriction option analysis;  Hazard or exposure;  Environmental emissions;  Information on alternatives;  Information on costs;  Information on benefits;  Transitional period  **Attachment:**   | **Comment:**  - |
|  |  | **Answer to specific info request 1:**  Legislation and voluntary standards is practically applicable to the situation where rubber granules is used on artificial turfs. (see separate attachment) |
|  |  | **Answer to specific info request 2:**  2. The supervision and analyzing costs can get extremely high for the municipalities if granules is not under the scope of this legislation. (see separate attachment) |
|  |  | **Answer to specific info request 5:**  SALAR recommend ECHA to further analyse the market for rubber infill materials in horseback riding ground surfaces. |

| **Stakeholder** | **ECHA date** | **Expressed interests/opinion on microplastics Annex XV comments** |
| --- | --- | --- |
| Badischer Fußballverband e. V.  (National NGO, Germany) | 2019/05/18  **Content:**  Scope or restriction option analysis;  Environmental emissions;  Information on costs;  Other socio economic analysis (SEA) issues;  Transitional period  **Attachment:**   | **Answer to specific info request 2:**  a.: Nach neueren Erkenntnissen werden zwischen 0,25 t/a und 5 t/a in Deutschland an Befüllungsgranulat pro Kunststoffrasenfläche verwendet (Fraunhofer 2018, S. 11). Das entspricht einer Gesamtmenge von ca. 7.500 bis 9.900 t/a.  b.: Nach dem aktuellen Forschungsstand besteht nach Kenntnis des DFB ein hohes Maß an Unsicherheit darüber, wie und in welchen Mengen das als Mikroplastik definierte Granulat auf Sportplätzen in die Umwelt freigesetzt wird. Nach den uns zur Verfügung stehen-den Informationen gibt es große Unterschiede bei der Einschätzung der Menge an Mikroplastiken, die in den einzelnen Mitgliedstaaten oder in der EU/EWR als Füllmaterial für Kunstrasen verwendet wird. Insbesondere Umfang und Methodologie der Forschung in diesem Bereich sind bisher noch wenig standardisiert und nachvollziehbar. Der DFB geht davon aus, dass der Anteil des Eintrags von Mikroplastik über Kunststoffrasenplätze je nach Mitgliedstaat ca. 1 bis 3 Prozent im Verhältnis zum Gesamteintrag beträgt. Demnach ist der Umwelteintrag verglichen mit anderen Hauptquellen relativ gering (Europäische Kommission 2018, ii)).  c.: Gezielte Risikomanagementmaßnahmen können die Freisetzung von Füllstoffen in die Umwelt bereits signifikant vermindern. Technische Maßnahmen zur Zurückhaltung eines Materialaustrags vor Ort (z.B. Rinnenfilter mit Sedimentationsstrecken an Abläufen, Schmutzfangmatten, Schuhbürsten am Ausgang) und organisatorische Maßnahmen beim Betrieb der Sportplätze (z.B. regelmäßige Reinigung der Spielfeldränder, Auffangsiebe) können zu einer starken Verringerung des Austrags von Mikroplastik beitragen.  Neben dem häufig genutzten Kunststoffgranulat existieren für Kunststoffrasensysteme alternative Füllstoffe, die in Teilen auch bereits beim Betrieb von Sportanlagen genutzt werden. So werden in Deutschland aktuell Kunststoffrasenplätze teilweise mit Sand und/oder Kork verfüllt. Zudem gibt es auch Kunststoffrasensysteme, die ohne elastischen Füllstoff betrieben werden können.  Es existieren bisher allerdings nur wenige belastbare Studien darüber, wie sich diese Alternativen qualitäts- und kostenmäßig (z.B. hinsichtlich der Bespielbarkeit und Lebensdauer) vergleichen lassen. Zudem müsste untersucht werden, ob und wie sich die Bespielbar-keit oder das Verletzungsrisiko der alternativ befüllten Kunststoffrasenflächen bei den verschiedenen Alternativfüllungen verändert (Plan Miljø Studie 2017). Es bedarf daher dringend weiterer wissenschaftlicher Expertise zur Praxistauglichkeit alternativer organischer Füllstoffe und zur sportartspezifischen Eignung von Kunststoffrasenplätzen, die ohne Füllstoffe auskommen. Sowohl eine wissenschaftliche Folgenabschätzung als auch die dringend erforderliche Entwicklung alternativer Füllstoffe durch die Industrie sind eine zentrale Forderung der von der Thematik betroffenen Sportverbände in Deutschland. Sie vertreten die Meinung, dass die Maßnahmen, die ein Verbot des Kunststoffgranulats verursachen würden, nicht kurzfristig umsetzbar sind und Alternativen nur mittel- bis langfristig erarbeitet und bereitgestellt werden können.  d.: In Deutschland gibt es ca. 5.000 für den Fußballspielbetrieb gemeldete Kunststoffrasenplätze (DFBnet), sowie ca. 1.000 DFB-Minispielfelder. Jährlich werden in Deutschland ca. 300 Kunststoffrasenplätze neu gebaut, sowie 150 Kunststoffrasenplätze von Grund auf erneuert. Hinsichtlich der bestehenden Plätze dürfte eine Umstellung auf alternative Füllstoffe notwendig sein. Hierfür halten die Sportanlagenbetreiber (Kommunen oder Vereine) Mittel für Sportstättenbau und -sanierung vor, die bei einem vollständigen Ver-bot und einer Verwendung alternativer Füllstoffe deutlich höher ausfallen würden. Laut eigener Berechnungen belaufen sich die jährlichen Mehrkosten deutschlandweit auf einen hohen einstelligen Millionenbetrag. Die insgesamt zu erwartenden Kosten eines Verbotes können aufgrund fehlender Kenntnisse über geeignete alternative Füllstoffe (Geeignetheit, Verfügbarkeit) derzeit nicht seriös beziffert werden. Auf Grundlage aktueller Daten zum Bau von Kunststoffrasenplätzen dürfte der Gesamtbetrag für den Austausch des Füllstoffes der Kunststoffrasensysteme im hohen zweistelligen Millionenbereich (bis zu 90 Mio. EUR) liegen, wobei zur Präzisierung dieses Schätzwertes vertiefte Analysen erforderlich sind. Die Kosten für eine Umsetzung gezielter Risikomanagementmaßnahmen zur Zurückhaltung des Materialaustrags dürften nach Schätzungen und je nach Umfang der Maßnahmen pro Kunststoffrasensystem bei 3.000 bis 10.000 EUR liegen.  e.: Der gemeinwohlorientierte Sport ist die größte zivilgesellschaftliche Bewegung in Deutschland und Europa. In Deutschland engagieren sich knapp acht Millionen Bürger freiwillig und ehrenamtlich im Sport. Das entspricht einer jährlichen Wertschöpfung und einem Wohlfahrtsgewinn allein in Deutschland von ca. 6,7 Milliarden Euro. Vergleichbare Zahlen lassen sich auch für die gesamte EU feststellen. In den EU-Mitgliedstaaten engagieren sich im Jahre 2010 zwischen 92 und 94 Millionen Menschen freiwillig für Ziele des Gemeinwohls, davon die meisten im Sport (ca. 35 bis 40 Prozent aller freiwillig Tätigen in der EU) (Europäische Kommission 2010).  Der Sport schafft ein strukturiertes, an die gesamte Bevölkerung gerichtetes und für alle offenes Bewegungs- und Sportangebot, durch das wichtige soziale und gesundheitsfördernde Funktionen in der Gesellschaft erfüllt werden. Sportvereine in Deutschland zählen zehn Millionen Mitgliedschaften im Kinder- und Jugendalter (DOSB-Bestandserhebung 2018), allein im DFB liegt diese Zahl bei 2,1 Millionen (DFB-Mitgliederstatistik 2018). Damit sind Sportvereine die wichtigste Anlaufstelle für Kinder und Jugendliche außerhalb der Schule und übernehmen unverzichtbare Aufgaben für die ganzheitliche Persönlichkeitsbildung junger Menschen. Dem Sport kommt eine wichtige Vorbild- und Lehrfunktion im Bereich der Integration und demokratischen Grundbildung zu. Für das herausragende gesellschaftliche Engagement des Sports spricht nicht zuletzt, dass die Sportvereine eng mit Schulen, Kindergärten, Unternehmen, Krankenkassen oder anderen öffentlichen Institutionen zusammenarbeiten. Um allen Bürgern den Zugang zum Sport zu ermöglichen, sind adäquate Sportstätten in ausreichender Anzahl Grundvoraussetzung. Ein für alle zugängliches und umfangreiches Sportangebot ist – vor allen Dingen in Großstädten und Ballungsgebieten – nur durch die Verfügbarkeit von ganzjährig nutzbaren Sportanlagen zu gewährleisten. Kunststoffrasenplätze spielen hierbei, insbesondere für den Fußball, eine wichtige Rolle, da sie eine intensivere Nutzung als Naturrasen- oder Tennenplätze erlauben. Allein mit Naturrasen- und Tennenplätzen lässt sich der derzeitige Trainings- und Spielbetrieb, insbesondere bei den Kinder- und Jugendmannschaften, nicht aufrechterhalten. Ein Kunststoffrasenplatz ersetzt etwa 2,5 Naturrasenplätze (DFBnet). Auf weniger als 10 Prozent der Naturrasenplätze finden an Wochenenden mehr als 2 Spiele statt. Bei Kunststoffrasenplätzen finden hingegen bei über 40 Prozent der Plätze mehr als 2 Spiele statt. Weniger als 10 Prozent der Naturrasenplätze wird an einem Wochenende mehr als 150 Minuten genutzt. Bei Kunstrasenplätzen werden hingegen etwa 35 Prozent an einem Wochenende mehr als 150 Minuten genutzt. 27.773 Spielstätten in Deutschland (ca. 70 Prozent) werden von Sportvereinen genutzt. Ein Drittel der Kunstrasenplätze werden von 2 oder mehr Vereinen mit alle ihren Jugend- und Seniorenmannschaften benutzt. Etwas über ein Drittel aller Naturrasenplätze wird von mehr als 5 Mannschaften bespielt. Bei Kunstrasenplätzen werden fast drei Viertel (72 Prozent) von mehr als 5 Mannschaften genutzt. Etwa 10 Prozent aller Naturrasenplätze wird von mehr als 10 Mannschaften bespielt. Bei Kunststoffrasenplätzen sind es ca. 41 Prozent der Plätze, die von mehr als 10 Mannschaften genutzt. Nur 1 Prozent aller Naturrasenplätze wird von mehr als 15 Mannschaften bespielt. Bei Kunstrasenplätzen beträgt der Anteil immerhin noch knapp 18 Prozent. Etwa 6 Prozent werden sogar von über 20 Mannschaften bespielt. Je größer die Vereinsgröße (insbesondere Anzahl der Mannschaften), desto höher ist der Anteil der Vereine, die auch eine Spielstätte vom Typ Kunstrasen haben.  Ein Verbot des Inverkehrbringens von Kunststoffgranulaten als Füllstoff in Kunststoffrasensystemen direkt bei Inkrafttreten der Beschränkung wäre daher unverhältnismäßig. Es würde zu hohen, unerwarteten Umstellungskosten und Mehrkosten für Vereine und Kommunen führen, wodurch dem gemeinwohlorientierten Sport Mittel entzogen würden. Bei fehlender Finanzierbarkeit dieser Mehrkosten ist zudem von einer Schließung vieler Sportplätzen auszugehen, wodurch das Sportangebot in Schulen und Vereinen stark leiden würde. Gerade auf Vereinsebene stellt ein solch außerordentlicher Kosten-punkt ein großes finanzielles Risiko dar, dass das sportliche und gesellschaftliche Gesamtangebot des Vereins gefährden kann. Eine Beschränkung ohne Übergangsfristen, die eine mittelfristige Umstellung und Kostenstreckung erlauben, würde das Breitensportangebot in Deutschland sehr negativ beeinflussen.  Im Hinblick auf den Beschränkungsvorschlag der ECHA gemäß Anhang XV der REACH-Verordnung spricht sich der DFB daher für eine angemessene Übergangsfrist von mindestens sechs Jahren bis zu einem vollständigen Inverkehrbringungsverbot des Kunststoffgranulats zur Verwendung in neuen Kunststoffrasensystemen sowie für die Umstellung bestehender Flächen aus.  Im Badischen Fußballverband sind 609 Vereine mit über 200.000 Mitgliedern organisiert. Ein besonderer Schwerpunkt der verbandlichen Arbeit liegt im Bereich der Kooperation zwischen Schule und Verein sowie der Installierung von Maßnahmen und Projekten im Bereich des Freiwilligendienstes (FSJ). Knapp 60 FSJ-Projekte betreuen knapp 400 Kooperationsmaßnahmen Schule und Verein, die bei einer negativen Entscheidung hinsichtlich der Kunstrasenplätze in Gefahr geraten und damit das gesellschaftliche Engagement der Vereine in den Schulen gefährden würden. Besonders die Möglichkeit einer ganzjährigen Jugendarbeit mit Hilfe der Kunstrasenplätze als Existenzgrundlage für die Vereine würde damit verloren gehen, weshalb sich der Badische Fußballverband der Darstellung des DFB anschließt. |
| Landessportbund Rheinland-Pfalz (National NGO, Germany) | 2019/05/17  **Content:**  Scope or restriction option analysis;  Environmental emissions;  Information on costs;  Other socio economic analysis (SEA) issues;  Transitional period  **Attachment:**   | **Answer to specific info request 2:**  a.: Nach neueren Erkenntnissen werden zwischen 0,25 t/a und 5 t/a in Deutschland an Befüllungsgranulat pro Kunststoffrasenfläche verwendet (Fraunhofer 2018, S. 11). Das entspricht einer Gesamtmenge von ca. 7.500 bis 9.900 t/a.  b.: Nach dem aktuellen Forschungsstand besteht nach Kenntnis des DFB ein hohes Maß an Unsicherheit darüber, wie und in welchen Mengen das als Mikroplastik definierte Granulat auf Sportplätzen in die Umwelt freigesetzt wird. Nach den uns zur Verfügung stehenden Informationen gibt es große Unterschiede bei der Einschätzung der Menge an Mikroplastiken, die in den einzelnen Mitgliedstaaten oder in der EU/EWR als Füllmaterial für Kunstrasen verwendet wird. Insbesondere Umfang und Methodologie der Forschung in diesem Bereich sind bisher noch wenig standardisiert und nachvollziehbar. Der DFB geht davon aus, dass der Anteil des Eintrags von Mikroplastik über Kunststoffrasenplätze je nach Mitgliedstaat ca. 1 bis 3 Prozent im Verhältnis zum Gesamteintrag beträgt. Demnach ist der Umwelteintrag verglichen mit anderen Hauptquellen relativ gering (Europäische Kommission 2018, ii)).  c.: Gezielte Risikomanagementmaßnahmen können die Freisetzung von Füllstoffen in die Umwelt bereits signifikant vermindern. Technische Maßnahmen zur Zurückhaltung eines Materialaustrags vor Ort (z.B. Rinnenfilter mit Sedimentationsstrecken an Abläufen, Schmutzfangmatten, Schuhbürsten am Ausgang) und organisatorische Maßnahmen beim Betrieb der Sportplätze (z.B. regelmäßige Reinigung der Spielfeldränder, Auffangsiebe) können zu einer starken Verringerung des Austrags von Mikroplastik beitragen.  Neben dem häufig genutzten Kunststoffgranulat existieren für Kunststoffrasensysteme alternative Füllstoffe, die in Teilen auch bereits beim Betrieb von Sportanlagen genutzt werden. So werden in Deutschland aktuell Kunststoffrasenplätze teilweise mit Sand und/oder Kork verfüllt. Zudem gibt es auch Kunststoffrasensysteme, die ohne elastischen Füllstoff betrieben werden können.  Es existieren bisher allerdings nur wenige belastbare Studien darüber, wie sich diese Alternativen qualitäts- und kostenmäßig (z.B. hinsichtlich der Bespielbarkeit und Lebensdauer) vergleichen lassen. Zudem müsste untersucht werden, ob und wie sich die Bespielbarkeit oder das Verletzungsrisiko der alternativ befüllten Kunststoffrasenflächen bei den verschiedenen Alternativfüllungen verändert (Plan Miljö Studie 2017). Es bedarf daher dringend weiterer wissenschaftlicher Expertise zur Praxistauglichkeit alternativer organischer Füllstoffe und zur sportartspezifischen Eignung von Kunststoffrasenplätzen, die ohne Füllstoffe auskommen. Sowohl eine wissenschaftliche Folgenabschätzung als auch die dringend erforderliche Entwicklung alternativer Füllstoffe durch die Industrie sind eine zentrale Forderung der von der Thematik betroffenen Sportverbände in Deutschland. Sie vertreten die Meinung, dass die Maßnahmen, die ein Verbot des Kunststoffgranulats verursachen würden, nicht kurzfristig umsetzbar sind und Alternativen nur mittel- bis langfristig erarbeitet und bereitgestellt werden können.  d.: In Deutschland gibt es ca. 5.000 für den Fußballspielbetrieb gemeldete Kunststoffrasenplätze (DFBnet), sowie ca. 1.000 DFB-Minispielfelder. Jährlich werden in Deutschland ca. 300 Kunststoffrasenplätze neu gebaut, sowie 150 Kunststoffrasenplätze von Grund auf erneuert. Hinsichtlich der bestehenden Plätze dürfte eine Umstellung auf alternative Füllstoffe notwendig sein. Hierfür halten die Sportanlagenbetreiber (Kommunen oder Vereine) Mittel für Sportstättenbau und -sanierung vor, die bei einem vollständigen Verbot und einer Verwendung alternativer Füllstoffe deutlich höher ausfallen würden. Laut eigener Berechnungen belaufen sich die jährlichen Mehrkosten deutschlandweit auf einen hohen einstelligen Millionenbetrag. Die insgesamt zu erwartenden Kosten eines Verbotes können aufgrund fehlender Kenntnisse über geeignete alternative Füllstoffe (Geeignetheit, Verfügbarkeit) derzeit nicht seriös beziffert werden. Auf Grundlage aktueller Daten zum Bau von Kunststoffrasenplätzen dürfte der Gesamtbetrag für den Austausch des Füllstoffes der Kunststoffrasensysteme im hohen zweistelligen Millionenbereich (bis zu 90 Mio. EUR) liegen, wobei zur Präzisierung dieses Schätzwertes vertiefte Analysen erforderlich sind. Die Kosten für eine Umsetzung gezielter Risikomanagementmaßnahmen zur Zurückhaltung des Materialaustrags dürften nach Schätzungen und je nach Umfang der Maßnahmen pro Kunststoffrasensystem bei 3.000 bis 10.000 EUR liegen.  e.: Der gemeinwohlorientierte Sport ist die größte zivilgesellschaftliche Bewegung in Deutschland und Europa. In Deutschland engagieren sich knapp acht Millionen Bürger freiwillig und ehrenamtlich im Sport. Das entspricht einer jährlichen Wertschöpfung und einem Wohlfahrtsgewinn allein in Deutschland von ca. 6,7 Milliarden Euro. Vergleichbare Zahlen lassen sich auch für die gesamte EU feststellen. In den EU-Mitgliedstaaten engagieren sich im Jahre 2010 zwischen 92 und 94 Millionen Menschen freiwillig für Ziele des Gemeinwohls, davon die meisten im Sport (ca. 35 bis 40 Prozent aller freiwillig Tätigen in der EU) (Europäische Kommission 2010).  Der Sport schafft ein strukturiertes, an die gesamte Bevölkerung gerichtetes und für alle offenes Bewegungs- und Sportangebot, durch das wichtige soziale und gesundheitsfördernde Funktionen in der Gesellschaft erfüllt werden. Sportvereine in Deutschland zählen zehn Millionen Mitgliedschaften im Kinder- und Jugendalter (DOSB-Bestandserhebung 2018), allein im DFB liegt diese Zahl bei 2,1 Millionen (DFB-Mitgliederstatistik 2018). Damit sind Sportvereine die wichtigste Anlaufstelle für Kinder und Jugendliche außerhalb der Schule und übernehmen unverzichtbare Aufgaben für die ganzheitliche Persönlichkeitsbildung junger Menschen. Dem Sport kommt eine wichtige Vorbild- und Lehrfunktion im Bereich der Integration und demokratischen Grundbildung zu. Für das herausragende gesellschaftliche Engagement des Sports spricht nicht zuletzt, dass die Sportvereine eng mit Schulen, Kindergärten, Unternehmen, Krankenkassen oder anderen öffentlichen Institutionen zusammenarbeiten. Um allen Bürgern den Zugang zum Sport zu ermöglichen, sind adäquate Sportstätten in ausreichender Anzahl Grundvoraussetzung. Ein für alle zugängliches und umfangreiches Sportangebot ist – vor allen Dingen in Großstädten und Ballungsgebieten – nur durch die Verfügbarkeit von ganzjährig nutzbaren Sportanlagen zu gewährleisten. Kunststoffrasenplätze spielen hierbei, insbesondere für den Fußball, eine wichtige Rolle, da sie eine intensivere Nutzung als Naturrasen- oder Tennenplätze erlauben. Allein mit Naturrasen- und Tennenplätzen lässt sich der derzeitige Trainings- und Spielbetrieb, insbesondere bei den Kinder- und Jugendmannschaften, nicht aufrechterhalten. Ein Kunststoffrasenplatz ersetzt etwa 2,5 Naturrasenplätze (DFBnet). Auf weniger als 10 Prozent der Naturrasenplätze finden an Wochenenden mehr als 2 Spiele statt. Bei Kunststoffrasenplätzen finden hingegen bei über 40 Prozent der Plätze mehr als 2 Spiele statt. Weniger als 10 Prozent der Naturrasenplätze wird an einem Wochenende mehr als 150 Minuten genutzt. Bei Kunstrasenplätzen werden hingegen etwa 35 Prozent an einem Wochenende mehr als 150 Minuten genutzt. 27.773 Spielstätten in Deutschland (ca. 70 Prozent) werden von Sportvereinen genutzt. Ein Drittel der Kunstrasenplätze werden von 2 oder mehr Vereinen mit alle ihren Jugend- und Seniorenmannschaften benutzt. Etwas über ein Drittel aller Naturrasenplätze wird von mehr als 5 Mannschaften bespielt. Bei Kunstrasenplätzen werden fast drei Viertel (72 Prozent) von mehr als 5 Mannschaften genutzt. Etwa 10 Prozent aller Naturrasenplätze wird von mehr als 10 Mannschaften bespielt. Bei Kunststoffrasenplätzen sind es ca. 41 Prozent der Plätze, die von mehr als 10 Mannschaften genutzt. Nur 1 Prozent aller Naturrasenplätze wird von mehr als 15 Mannschaften bespielt. Bei Kunstrasenplätzen beträgt der Anteil immerhin noch knapp 18 Prozent. Etwa 6 Prozent werden sogar von über 20 Mannschaften bespielt. Je größer die Vereinsgröße (insbesondere Anzahl der Mannschaften), desto höher ist der Anteil der Vereine, die auch eine Spielstätte vom Typ Kunstrasen haben.  Ein Verbot des Inverkehrbringens von Kunststoffgranulaten als Füllstoff in Kunststoffrasensystemen direkt bei Inkrafttreten der Beschränkung wäre daher unverhältnismäßig. Es würde zu hohen, unerwarteten Umstellungskosten und Mehrkosten für Vereine und Kommunen führen, wodurch dem gemeinwohlorientierten Sport Mittel entzogen würden. Bei fehlender Finanzierbarkeit dieser Mehrkosten ist zudem von einer Schließung vieler Sportplätzen auszugehen, wodurch das Sportangebot in Schulen und Vereinen stark leiden würde. Gerade auf Vereinsebene stellt ein solch außerordentlicher Kosten-punkt ein großes finanzielles Risiko dar, dass das sportliche und gesellschaftliche Gesamtangebot des Vereins gefährden kann. Eine Beschränkung ohne Übergangsfristen, die eine mittelfristige Umstellung und Kostenstreckung erlauben, würde das Breitensportangebot in Deutschland sehr negativ beeinflussen.  Im Hinblick auf den Beschränkungsvorschlag der ECHA gemäß Anhang XV der REACH-Verordnung spricht sich der DFB daher für eine angemessene Übergangsfrist von mindestens sechs Jahren bis zu einem vollständigen Inverkehrbringungsverbot des Kunststoffgranulats zur Verwendung in neuen Kunststoffrasensystemen sowie für die Umstellung bestehender Flächen aus. |
|  |  | **Comment:**  For these reasons, we think that flock fibres up to 15 mm long, should not be declared as microplastic in relationto your study and recommendations. |
| Sportbund Pfalz e. V. (National NGO, Germany | 2019/05/17  **Content:**  Scope or restriction option analysis;  Environmental emissions;  Information on costs;  Other socio economic analysis (SEA) issues;  Transitional period  **Attachment:**   | **Comments**  **-** |
|  |  | **Answer to specific info request 2:**  a.: Nach neueren Erkenntnissen werden zwischen 0,25 t/a und 5 t/a in Deutschland an Befüllungsgranulat pro Kunststoffrasenfläche verwendet (Fraunhofer 2018, S. 11). Das entspricht einer Gesamtmenge von ca. 7.500 bis 9.900 t/a.  b.: Nach dem aktuellen Forschungsstand besteht nach Kenntnis des DFB ein hohes Maß an Unsicherheit darüber, wie und in welchen Mengen das als Mikroplastik definierte Granulat auf Sportplätzen in die Umwelt freigesetzt wird. Nach den uns zur Verfügung stehen-den Informationen gibt es große Unterschiede bei der Einschätzung der Menge an Mikroplastiken, die in den einzelnen Mitgliedstaaten oder in der EU/EWR als Füllmaterial für Kunstrasen verwendet wird. Insbesondere Umfang und Methodologie der Forschung in diesem Bereich sind bisher noch wenig standardisiert und nachvollziehbar. Der DFB geht davon aus, dass der Anteil des Eintrags von Mikroplastik über Kunststoffrasenplätze je nach Mitgliedstaat ca. 1 bis 3 Prozent im Verhältnis zum Gesamteintrag beträgt. Demnach ist der Umwelteintrag verglichen mit anderen Hauptquellen relativ gering (Europäische Kommission 2018, ii)).  c.: Gezielte Risikomanagementmaßnahmen können die Freisetzung von Füllstoffen in die Umwelt bereits signifikant vermindern. Technische Maßnahmen zur Zurückhaltung eines Materialaustrags vor Ort (z.B. Rinnenfilter mit Sedimentationsstrecken an Abläufen, Schmutzfangmatten, Schuhbürsten am Ausgang) und organisatorische Maßnahmen beim Betrieb der Sportplätze (z.B. regelmäßige Reinigung der Spielfeldränder, Auffangsiebe) können zu einer starken Verringerung des Austrags von Mikroplastik beitragen.  Neben dem häufig genutzten Kunststoffgranulat existieren für Kunststoffrasensysteme alternative Füllstoffe, die in Teilen auch bereits beim Betrieb von Sportanlagen genutzt werden. So werden in Deutschland aktuell Kunststoffrasenplätze teilweise mit Sand und/oder Kork verfüllt. Zudem gibt es auch Kunststoffrasensysteme, die ohne elastischen Füllstoff betrieben werden können.  Es existieren bisher allerdings nur wenige belastbare Studien darüber, wie sich diese Alternativen qualitäts- und kostenmäßig (z.B. hinsichtlich der Bespielbarkeit und Lebensdauer) vergleichen lassen. Zudem müsste untersucht werden, ob und wie sich die Bespielbar-keit oder das Verletzungsrisiko der alternativ befüllten Kunststoffrasenflächen bei den verschiedenen Alternativfüllungen verändert (Plan Miljø Studie 2017). Es bedarf daher dringend weiterer wissenschaftlicher Expertise zur Praxistauglichkeit alternativer organischer Füllstoffe und zur sportartspezifischen Eignung von Kunststoffrasenplätzen, die ohne Füllstoffe auskommen. Sowohl eine wissenschaftliche Folgenabschätzung als auch die dringend erforderliche Entwicklung alternativer Füllstoffe durch die Industrie sind eine zentrale Forderung der von der Thematik betroffenen Sportverbände in Deutschland. Sie vertreten die Meinung, dass die Maßnahmen, die ein Verbot des Kunststoffgranulats verursachen würden, nicht kurzfristig umsetzbar sind und Alternativen nur mittel- bis langfristig erarbeitet und bereitgestellt werden können.  d.: In Deutschland gibt es ca. 5.000 für den Fußballspielbetrieb gemeldete Kunststoffrasenplätze (DFBnet), sowie ca. 1.000 DFB-Minispielfelder. Jährlich werden in Deutschland ca. 300 Kunststoffrasenplätze neu gebaut, sowie 150 Kunststoffrasenplätze von Grund auf erneuert. Hinsichtlich der bestehenden Plätze dürfte eine Umstellung auf alternative Füllstoffe notwendig sein. Hierfür halten die Sportanlagenbetreiber (Kommunen oder Vereine) Mittel für Sportstättenbau und -sanierung vor, die bei einem vollständigen Ver-bot und einer Verwendung alternativer Füllstoffe deutlich höher ausfallen würden. Laut eigener Berechnungen belaufen sich die jährlichen Mehrkosten deutschlandweit auf einen hohen einstelligen Millionenbetrag. Die insgesamt zu erwartenden Kosten eines Verbotes können aufgrund fehlender Kenntnisse über geeignete alternative Füllstoffe (Geeignetheit, Verfügbarkeit) derzeit nicht seriös beziffert werden. Auf Grundlage aktueller Daten zum Bau von Kunststoffrasenplätzen dürfte der Gesamtbetrag für den Austausch des Füllstoffes der Kunststoffrasensysteme im hohen zweistelligen Millionenbereich (bis zu 90 Mio. EUR) liegen, wobei zur Präzisierung dieses Schätzwertes vertiefte Analysen erforderlich sind. Die Kosten für eine Umsetzung gezielter Risikomanagementmaßnahmen zur Zurückhaltung des Materialaustrags dürften nach Schätzungen und je nach Umfang der Maßnahmen pro Kunststoffrasensystem bei 3.000 bis 10.000 EUR liegen.  e.: Der gemeinwohlorientierte Sport ist die größte zivilgesellschaftliche Bewegung in Deutschland und Europa. In Deutschland engagieren sich knapp acht Millionen Bürger freiwillig und ehrenamtlich im Sport. Das entspricht einer jährlichen Wertschöpfung und einem Wohlfahrtsgewinn allein in Deutschland von ca. 6,7 Milliarden Euro. Vergleichbare Zahlen lassen sich auch für die gesamte EU feststellen. In den EU-Mitgliedstaaten engagieren sich im Jahre 2010 zwischen 92 und 94 Millionen Menschen freiwillig für Ziele des Gemeinwohls, davon die meisten im Sport (ca. 35 bis 40 Prozent aller freiwillig Tätigen in der EU) (Europäische Kommission 2010).  Der Sport schafft ein strukturiertes, an die gesamte Bevölkerung gerichtetes und für alle offenes Bewegungs- und Sportangebot, durch das wichtige soziale und gesundheitsfördernde Funktionen in der Gesellschaft erfüllt werden. Sportvereine in Deutschland zählen zehn Millionen Mitgliedschaften im Kinder- und Jugendalter (DOSB-Bestandserhebung 2018), allein im DFB liegt diese Zahl bei 2,1 Millionen (DFB-Mitgliederstatistik 2018). Damit sind Sportvereine die wichtigste Anlaufstelle für Kinder und Jugendliche außerhalb der Schule und übernehmen unverzichtbare Aufgaben für die ganzheitliche Persönlichkeitsbildung junger Menschen. Dem Sport kommt eine wichtige Vorbild- und Lehrfunktion im Bereich der Integration und demokratischen Grundbildung zu. Für das herausragende gesellschaftliche Engagement des Sports spricht nicht zuletzt, dass die Sportvereine eng mit Schulen, Kindergärten, Unternehmen, Krankenkassen oder anderen öffentlichen Institutionen zusammenarbeiten. Um allen Bürgern den Zugang zum Sport zu ermöglichen, sind adäquate Sportstätten in ausreichender Anzahl Grundvoraussetzung. Ein für alle zugängliches und umfangreiches Sportangebot ist – vor allen Dingen in Großstädten und Ballungsgebieten – nur durch die Verfügbarkeit von ganzjährig nutzbaren Sportanlagen zu gewährleisten. Kunststoffrasenplätze spielen hierbei, insbesondere für den Fußball, eine wichtige Rolle, da sie eine intensivere Nutzung als Naturrasen- oder Tennenplätze erlauben. Allein mit Naturrasen- und Tennenplätzen lässt sich der derzeitige Trainings- und Spielbetrieb, insbesondere bei den Kinder- und Jugendmannschaften, nicht aufrechterhalten. Ein Kunststoffrasenplatz ersetzt etwa 2,5 Naturrasenplätze (DFBnet). Auf weniger als 10 Prozent der Naturrasenplätze finden an Wochenenden mehr als 2 Spiele statt. Bei Kunststoffrasenplätzen finden hingegen bei über 40 Prozent der Plätze mehr als 2 Spiele statt. Weniger als 10 Prozent der Naturrasenplätze wird an einem Wochenende mehr als 150 Minuten genutzt. Bei Kunstrasenplätzen werden hingegen etwa 35 Prozent an einem Wochenende mehr als 150 Minuten genutzt. 27.773 Spielstätten in Deutschland (ca. 70 Prozent) werden von Sportvereinen genutzt. Ein Drittel der Kunstrasenplätze werden von 2 oder mehr Vereinen mit alle ihren Jugend- und Seniorenmannschaften benutzt. Etwas über ein Drittel aller Naturrasenplätze wird von mehr als 5 Mannschaften bespielt. Bei Kunstrasenplätzen werden fast drei Viertel (72 Prozent) von mehr als 5 Mannschaften genutzt. Etwa 10 Prozent aller Naturrasenplätze wird von mehr als 10 Mannschaften bespielt. Bei Kunststoffrasenplätzen sind es ca. 41 Prozent der Plätze, die von mehr als 10 Mannschaften genutzt. Nur 1 Prozent aller Naturrasenplätze wird von mehr als 15 Mannschaften bespielt. Bei Kunstrasenplätzen beträgt der Anteil immerhin noch knapp 18 Prozent. Etwa 6 Prozent werden sogar von über 20 Mannschaften bespielt. Je größer die Vereinsgröße (insbesondere Anzahl der Mannschaften), desto höher ist der Anteil der Vereine, die auch eine Spielstätte vom Typ Kunstrasen haben.  Ein Verbot des Inverkehrbringens von Kunststoffgranulaten als Füllstoff in Kunststoffrasensystemen direkt bei Inkrafttreten der Beschränkung wäre daher unverhältnismäßig. Es würde zu hohen, unerwarteten Umstellungskosten und Mehrkosten für Vereine und Kommunen führen, wodurch dem gemeinwohlorientierten Sport Mittel entzogen würden. Bei fehlender Finanzierbarkeit dieser Mehrkosten ist zudem von einer Schließung vieler Sportplätzen auszugehen, wodurch das Sportangebot in Schulen und Vereinen stark leiden würde. Gerade auf Vereinsebene stellt ein solch außerordentlicher Kosten-punkt ein großes finanzielles Risiko dar, dass das sportliche und gesellschaftliche Gesamtangebot des Vereins gefährden kann. Eine Beschränkung ohne Übergangsfristen, die eine mittelfristige Umstellung und Kostenstreckung erlauben, würde das Breitensportangebot in Deutschland sehr negativ beeinflussen.  Im Hinblick auf den Beschränkungsvorschlag der ECHA gemäß Anhang XV der REACH-Verordnung spricht sich der DFB daher für eine angemessene Übergangsfrist von mindestens sechs Jahren bis zu einem vollständigen Inverkehrbringungsverbot des Kunststoffgranulats zur Verwendung in neuen Kunststoffrasensystemen sowie für die Umstellung bestehender Flächen aus. |
| Saarländischer Fußballverband e.V. (National NGO, Germany) | **Date:** 2019/05/17  **Content:**  Scope or restriction option analysis;  Environmental emissions;  Information on costs;  Other socio economic analysis (SEA) issues;  Transitional period  **Attachment:**   | **Comment:**  - |
|  |  | **Answer to specific info request 2:**  a.: Nach neueren Erkenntnissen werden zwischen 0,25 t/a und 5 t/a in Deutschland an Befüllungsgranulat pro Kunststoffrasenfläche verwendet (Fraunhofer 2018, S. 11). Das entspricht einer Gesamtmenge von ca. 7.500 bis 9.900 t/a.  b.: Nach dem aktuellen Forschungsstand besteht nach Kenntnis des DFB ein hohes Maß an Unsicherheit darüber, wie und in welchen Mengen das als Mikroplastik definierte Granulat auf Sportplätzen in die Umwelt freigesetzt wird. Nach den uns zur Verfügung stehen-den Informationen gibt es große Unterschiede bei der Einschätzung der Menge an Mikroplastiken, die in den einzelnen Mitgliedstaaten oder in der EU/EWR als Füllmaterial für Kunstrasen verwendet wird. Insbesondere Umfang und Methodologie der Forschung in diesem Bereich sind bisher noch wenig standardisiert und nachvollziehbar. Der DFB geht davon aus, dass der Anteil des Eintrags von Mikroplastik über Kunststoffrasenplätze je nach Mitgliedstaat ca. 1 bis 3 Prozent im Verhältnis zum Gesamteintrag beträgt. Demnach ist der Umwelteintrag verglichen mit anderen Hauptquellen relativ gering (Europäische Kommission 2018, ii)).  c.: Gezielte Risikomanagementmaßnahmen können die Freisetzung von Füllstoffen in die Umwelt bereits signifikant vermindern. Technische Maßnahmen zur Zurückhaltung eines Materialaustrags vor Ort (z.B. Rinnenfilter mit Sedimentationsstrecken an Abläufen, Schmutzfangmatten, Schuhbürsten am Ausgang) und organisatorische Maßnahmen beim Betrieb der Sportplätze (z.B. regelmäßige Reinigung der Spielfeldränder, Auffangsiebe) können zu einer starken Verringerung des Austrags von Mikroplastik beitragen.  Neben dem häufig genutzten Kunststoffgranulat existieren für Kunststoffrasensysteme alternative Füllstoffe, die in Teilen auch bereits beim Betrieb von Sportanlagen genutzt werden. So werden in Deutschland aktuell Kunststoffrasenplätze teilweise mit Sand und/oder Kork verfüllt. Zudem gibt es auch Kunststoffrasensysteme, die ohne elastischen Füllstoff betrieben werden können.  Es existieren bisher allerdings nur wenige belastbare Studien darüber, wie sich diese Alternativen qualitäts- und kostenmäßig (z.B. hinsichtlich der Bespielbarkeit und Lebensdauer) vergleichen lassen. Zudem müsste untersucht werden, ob und wie sich die Bespielbar-keit oder das Verletzungsrisiko der alternativ befüllten Kunststoffrasenflächen bei den verschiedenen Alternativfüllungen verändert (Plan Miljø Studie 2017). Es bedarf daher dringend weiterer wissenschaftlicher Expertise zur Praxistauglichkeit alternativer organischer Füllstoffe und zur sportartspezifischen Eignung von Kunststoffrasenplätzen, die ohne Füllstoffe auskommen. Sowohl eine wissenschaftliche Folgenabschätzung als auch die dringend erforderliche Entwicklung alternativer Füllstoffe durch die Industrie sind eine zentrale Forderung der von der Thematik betroffenen Sportverbände in Deutschland. Sie vertreten die Meinung, dass die Maßnahmen, die ein Verbot des Kunststoffgranulats verursachen würden, nicht kurzfristig umsetzbar sind und Alternativen nur mittel- bis langfristig erarbeitet und bereitgestellt werden können.  d.: In Deutschland gibt es ca. 5.000 für den Fußballspielbetrieb gemeldete Kunststoffrasenplätze (DFBnet), sowie ca. 1.000 DFB-Minispielfelder. Jährlich werden in Deutschland ca. 300 Kunststoffrasenplätze neu gebaut, sowie 150 Kunststoffrasenplätze von Grund auf erneuert. Hinsichtlich der bestehenden Plätze dürfte eine Umstellung auf alternative Füllstoffe notwendig sein. Hierfür halten die Sportanlagenbetreiber (Kommunen oder Vereine) Mittel für Sportstättenbau und -sanierung vor, die bei einem vollständigen Ver-bot und einer Verwendung alternativer Füllstoffe deutlich höher ausfallen würden. Laut eigener Berechnungen belaufen sich die jährlichen Mehrkosten deutschlandweit auf einen hohen einstelligen Millionenbetrag. Die insgesamt zu erwartenden Kosten eines Verbotes können aufgrund fehlender Kenntnisse über geeignete alternative Füllstoffe (Geeignetheit, Verfügbarkeit) derzeit nicht seriös beziffert werden. Auf Grundlage aktueller Daten zum Bau von Kunststoffrasenplätzen dürfte der Gesamtbetrag für den Austausch des Füllstoffes der Kunststoffrasensysteme im hohen zweistelligen Millionenbereich (bis zu 90 Mio. EUR) liegen, wobei zur Präzisierung dieses Schätzwertes vertiefte Analysen erforderlich sind. Die Kosten für eine Umsetzung gezielter Risikomanagementmaßnahmen zur Zurückhaltung des Materialaustrags dürften nach Schätzungen und je nach Umfang der Maßnahmen pro Kunststoffrasensystem bei 3.000 bis 10.000 EUR liegen.  e.: Der gemeinwohlorientierte Sport ist die größte zivilgesellschaftliche Bewegung in Deutschland und Europa. In Deutschland engagieren sich knapp acht Millionen Bürger freiwillig und ehrenamtlich im Sport. Das entspricht einer jährlichen Wertschöpfung und einem Wohlfahrtsgewinn allein in Deutschland von ca. 6,7 Milliarden Euro. Vergleichbare Zahlen lassen sich auch für die gesamte EU feststellen. In den EU-Mitgliedstaaten engagieren sich im Jahre 2010 zwischen 92 und 94 Millionen Menschen freiwillig für Ziele des Gemeinwohls, davon die meisten im Sport (ca. 35 bis 40 Prozent aller freiwillig Tätigen in der EU) (Europäische Kommission 2010).  Der Sport schafft ein strukturiertes, an die gesamte Bevölkerung gerichtetes und für alle offenes Bewegungs- und Sportangebot, durch das wichtige soziale und gesundheitsfördernde Funktionen in der Gesellschaft erfüllt werden. Sportvereine in Deutschland zählen zehn Millionen Mitgliedschaften im Kinder- und Jugendalter (DOSB-Bestandserhebung 2018), allein im DFB liegt diese Zahl bei 2,1 Millionen (DFB-Mitgliederstatistik 2018). Damit sind Sportvereine die wichtigste Anlaufstelle für Kinder und Jugendliche außerhalb der Schule und übernehmen unverzichtbare Aufgaben für die ganzheitliche Persönlichkeitsbildung junger Menschen. Dem Sport kommt eine wichtige Vorbild- und Lehrfunktion im Bereich der Integration und demokratischen Grundbildung zu. Für das herausragende gesellschaftliche Engagement des Sports spricht nicht zuletzt, dass die Sportvereine eng mit Schulen, Kindergärten, Unternehmen, Krankenkassen oder anderen öffentlichen Institutionen zusammenarbeiten. Um allen Bürgern den Zugang zum Sport zu ermöglichen, sind adäquate Sportstätten in ausreichender Anzahl Grundvoraussetzung. Ein für alle zugängliches und umfangreiches Sportangebot ist – vor allen Dingen in Großstädten und Ballungsgebieten – nur durch die Verfügbarkeit von ganzjährig nutzbaren Sportanlagen zu gewährleisten. Kunststoffrasenplätze spielen hierbei, insbesondere für den Fußball, eine wichtige Rolle, da sie eine intensivere Nutzung als Naturrasen- oder Tennenplätze erlauben. Allein mit Naturrasen- und Tennenplätzen lässt sich der derzeitige Trainings- und Spielbetrieb, insbesondere bei den Kinder- und Jugendmannschaften, nicht aufrechterhalten. Ein Kunststoffrasenplatz ersetzt etwa 2,5 Naturrasenplätze (DFBnet). Auf weniger als 10 Prozent der Naturrasenplätze finden an Wochenenden mehr als 2 Spiele statt. Bei Kunststoffrasenplätzen finden hingegen bei über 40 Prozent der Plätze mehr als 2 Spiele statt. Weniger als 10 Prozent der Naturrasenplätze wird an einem Wochenende mehr als 150 Minuten genutzt. Bei Kunstrasenplätzen werden hingegen etwa 35 Prozent an einem Wochenende mehr als 150 Minuten genutzt. 27.773 Spielstätten in Deutschland (ca. 70 Prozent) werden von Sportvereinen genutzt. Ein Drittel der Kunstrasenplätze werden von 2 oder mehr Vereinen mit alle ihren Jugend- und Seniorenmannschaften benutzt. Etwas über ein Drittel aller Naturrasenplätze wird von mehr als 5 Mannschaften bespielt. Bei Kunstrasenplätzen werden fast drei Viertel (72 Prozent) von mehr als 5 Mannschaften genutzt. Etwa 10 Prozent aller Naturrasenplätze wird von mehr als 10 Mannschaften bespielt. Bei Kunststoffrasenplätzen sind es ca. 41 Prozent der Plätze, die von mehr als 10 Mannschaften genutzt. Nur 1 Prozent aller Naturrasenplätze wird von mehr als 15 Mannschaften bespielt. Bei Kunstrasenplätzen beträgt der Anteil immerhin noch knapp 18 Prozent. Etwa 6 Prozent werden sogar von über 20 Mannschaften bespielt. Je größer die Vereinsgröße (insbesondere Anzahl der Mannschaften), desto höher ist der Anteil der Vereine, die auch eine Spielstätte vom Typ Kunstrasen haben.  Ein Verbot des Inverkehrbringens von Kunststoffgranulaten als Füllstoff in Kunststoffrasensystemen direkt bei Inkrafttreten der Beschränkung wäre daher unverhältnismäßig. Es würde zu hohen, unerwarteten Umstellungskosten und Mehrkosten für Vereine und Kommunen führen, wodurch dem gemeinwohlorientierten Sport Mittel entzogen würden. Bei fehlender Finanzierbarkeit dieser Mehrkosten ist zudem von einer Schließung vieler Sportplätzen auszugehen, wodurch das Sportangebot in Schulen und Vereinen stark leiden würde. Gerade auf Vereinsebene stellt ein solch außerordentlicher Kosten-punkt ein großes finanzielles Risiko dar, dass das sportliche und gesellschaftliche Gesamtangebot des Vereins gefährden kann. Eine Beschränkung ohne Übergangsfristen, die eine mittelfristige Umstellung und Kostenstreckung erlauben, würde das Breitensportangebot in Deutschland sehr negativ beeinflussen.  Im Hinblick auf den Beschränkungsvorschlag der ECHA gemäß Anhang XV der REACH-Verordnung spricht sich der DFB daher für eine angemessene Übergangsfrist von mindestens sechs Jahren bis zu einem vollständigen Inverkehrbringungsverbot des Kunststoffgranulats zur Verwendung in neuen Kunststoffrasensystemen sowie für die Umstellung bestehender Flächen aus.  Im Saarland wird auf 141 Kunstrasenplätzen gespielt. Ca. 44% aller Vereine mit Spielbetrieb verfügen über einen Kunstrasenplatz, der Rest verteilt sich auf Rasenplatz und Hartplatz (Tenne). |
|  |  | **Comment:**  The BIR Tyre & Rubber Committee members have prepared a document to answer the specific questions on infill on synthetic turfs (see Enclosed document: BIR document microplastic restriction 20190517 Final version). Below a conclusion:  In order to be able to meet the objectives of the circular economy, it is indispensable that products have the possibility of a second product cycle. In the case of infill material from ELT, scientific data to date has shown this application in sports fields to be safe and suitable.  The use of primary rubber or other materials is often limited by the high costs involved. The total cost of a sports field can be a huge barrier for many local communities wishing to offer people (in particular children), the possibility of important sports interaction. A ban on the use of infill for synthetic turfs would have significant negative effects on football in Europe. The consequence to the society, if the construction and use of sports fields with infill material of any kind should be banned, is huge and is not justified, if a few simple rules in construction and maintenance of such fields are applied.   The amount that is spread to the environment is very limited and has been strongly exaggerated in the press. Furthermore, by using the "best practice" operational conditions, it can be limited to almost nothing.   It is documented that artificial turf pitches have a very positive impact on public health and the availability of contact sports all year around.   The only realistic alternative, from a performance point of view, to ELT-rubber as infill, is virgin TPE and EPDM rubber. Switching to virgin rubbers will be very expensive and will have negative impacts on the climate.   In case of a ban of ELT-rubber in artificial turf, this important market will disappear, and more end-of-life tyres will have to be incinerated e.g. in cement kilns. For each ton of ELT's that is shifted from recycling to incineration the environment will be exposed to a minimum of 1.1 tons extra emissions of CO2-equivalents.  The CEN TC217 WG 6 TG1 Task Group has propsed to draft a technical report to promote and advocate best practice (see question 3) in support of EN 15330-1.  Banning infill rubber granules will have a major impact on the business operations of all tire processing companies and the waste stream of ELT. The way forward is to define clear EU harmonized End of waste criteria for materials derived from ELT and "best practice" operational conditions.  Yours sincerely  BIR Tyre & Rubber Committee members  ESTATO Umweltservice GmbH  Genan Holding A/S  Granuband BV  Kargro Recycling bv  Murfitt Industries Ltd.  Ragn-Sells Tyre Recycling AB |
| Deutscher Fussball-Bund e.V.  (National NGO, Germany) | **Date:** 2019/05/17  **Content:**  Environmental emissions;  Information on alternatives;  Information on costs;  Information on benefits;  Transitional period  **Attachment:**   | **Answer to specific info request 2:**  a.: Nach neueren Erkenntnissen werden zwischen 0,25 t/a und 5 t/a in Deutschland an Befüllungsgranulat pro Kunststoffrasenfläche verwendet (Fraunhofer 2018, S. 11). Das entspricht einer Gesamtmenge von ca. 7.500 bis 9.900 t/a.  b.: Nach dem aktuellen Forschungsstand besteht nach Kenntnis des DFB ein hohes Maß an Unsicherheit darüber, wie und in welchen Mengen das als Mikroplastik definierte Granulat auf Sportplätzen in die Umwelt freigesetzt wird. Nach den uns zur Verfügung stehen-den Informationen gibt es große Unterschiede bei der Einschätzung der Menge an Mikroplastik, die in den einzelnen Mitgliedstaaten oder in der EU/EWR als Füllmaterial für Kunstrasen verwendet wird. Insbesondere Umfang und Methodologie der Forschung in diesem Bereich sind bisher noch wenig standardisiert und nachvollziehbar. Der DFB geht davon aus, dass der Anteil des Eintrags von Mikroplastik über Kunststoffrasenplätze je nach Mitgliedstaat ca. 1 bis 3 Prozent im Verhältnis zum Gesamteintrag beträgt. Demnach ist der Umwelteintrag verglichen mit anderen Hauptquellen relativ gering (Europäische Kommission 2018, ii)).  c.: Gezielte Risikomanagementmaßnahmen können die Freisetzung von Füllstoffen in die Umwelt bereits signifikant vermindern. Technische Maßnahmen zur Zurückhaltung eines Materialaustrags vor Ort (z.B. Rinnenfilter mit Sedimentationsstrecken an Abläufen, Schmutzfangmatten, Schuhbürsten am Ausgang) und organisatorische Maßnahmen beim Betrieb der Sportplätze (z.B. regelmäßige Reinigung der Spielfeldränder, Auffangsiebe) können zu einer starken Verringerung des Austrags von Mikroplastik beitragen.  Neben dem häufig genutzten Kunststoffgranulat existieren für Kunststoffrasensysteme alternative Füllstoffe, die in Teilen auch bereits beim Betrieb von Sportanlagen genutzt werden. So werden in Deutschland aktuell Kunststoffrasenplätze teilweise mit Sand und/oder Kork verfüllt. Zudem gibt es auch Kunststoffrasensysteme, die ohne elastischen Füllstoff betrieben werden können.  Es existieren bisher allerdings nur wenige belastbare Studien darüber, wie sich diese Alternativen qualitäts- und kostenmäßig (z.B. hinsichtlich der Bespielbarkeit und Lebensdauer) vergleichen lassen. Zudem müsste untersucht werden, ob und wie sich die Bespielbarkeit oder das Verletzungsrisiko der alternativ befüllten Kunststoffrasenflächen bei den verschiedenen Alternativfüllungen verändert (Plan Miljø Studie 2017). Es bedarf daher dringend weiterer wissenschaftlicher Expertise zur Praxistauglichkeit alternativer organischer Füllstoffe und zur sportartspezifischen Eignung von Kunststoffrasenplätzen, die ohne Füllstoffe auskommen. Sowohl eine wissenschaftliche Folgenabschätzung als auch die dringend erforderliche Entwicklung alternativer Füllstoffe durch die Industrie sind eine zentrale Forderung der von der Thematik betroffenen Sportverbände in Deutschland. Sie vertreten die Meinung, dass die Maßnahmen, die ein Verbot des Kunststoffgranulats verursachen würden, nicht kurzfristig umsetzbar sind und Alternativen nur mittel- bis langfristig erarbeitet und bereitgestellt werden können.  d.: In Deutschland gibt es ca. 5.000 für den Fußballspielbetrieb gemeldete Kunststoffrasenplätze (DFBnet), sowie ca. 1.000 DFB-Minispielfelder. Jährlich werden in Deutschland ca. 300 Kunststoffrasenplätze neu gebaut, sowie 150 Kunststoffrasenplätze von Grund auf erneuert. Hinsichtlich der bestehenden Plätze dürfte eine Umstellung auf alternative Füllstoffe notwendig sein. Hierfür halten die Sportanlagenbetreiber (Kommunen oder Vereine) Mittel für Sportstättenbau und -sanierung vor, die bei einem vollständigen Ver-bot und einer Verwendung alternativer Füllstoffe deutlich höher ausfallen würden. Laut eigener Berechnungen belaufen sich die jährlichen Mehrkosten deutschlandweit auf einen hohen einstelligen Millionenbetrag. Die insgesamt zu erwartenden Kosten eines Verbotes können aufgrund fehlender Kenntnisse über geeignete alternative Füllstoffe (Geeignetheit, Verfügbarkeit) derzeit nicht seriös beziffert werden. Auf Grundlage aktueller Daten zum Bau von Kunststoffrasenplätzen dürfte der Gesamtbetrag für den Austausch des Füllstoffes der Kunststoffrasensysteme im hohen zweistelligen Millionenbereich (bis zu 90 Mio. EUR) liegen, wobei zur Präzisierung dieses Schätzwertes vertiefte Analysen erforderlich sind. Die Kosten für eine Umsetzung gezielter Risikomanagementmaßnahmen zur Zurückhaltung des Materialaustrags dürften nach Schätzungen und je nach Umfang der Maßnahmen pro Kunststoffrasensystem bei 3.000 bis 10.000 EUR liegen.  e.: Der gemeinwohlorientierte Sport ist die größte zivilgesellschaftliche Bewegung in Deutschland und Europa. In Deutschland engagieren sich knapp acht Millionen Bürger freiwillig und ehrenamtlich im Sport. Das entspricht einer jährlichen Wertschöpfung und einem Wohlfahrtsgewinn allein in Deutschland von ca. 6,7 Milliarden Euro. Vergleichbare Zahlen lassen sich auch für die gesamte EU feststellen. In den EU-Mitgliedstaaten engagieren sich im Jahre 2010 zwischen 92 und 94 Millionen Menschen freiwillig für Ziele des Gemeinwohls, davon die meisten im Sport (ca. 35 bis 40 Prozent aller freiwillig Tätigen in der EU) (Europäische Kommission 2010).  Der Sport schafft ein strukturiertes, an die gesamte Bevölkerung gerichtetes und für alle offenes Bewegungs- und Sportangebot, durch das wichtige soziale und gesundheitsfördernde Funktionen in der Gesellschaft erfüllt werden. Sportvereine in Deutschland zählen zehn Millionen Mitgliedschaften im Kinder- und Jugendalter (DOSB-Bestandserhebung 2018), allein im DFB liegt diese Zahl bei 2,1 Millionen (DFB-Mitgliederstatistik 2018). Damit sind Sportvereine die wichtigste Anlaufstelle für Kinder und Jugendliche außerhalb der Schule und übernehmen unverzichtbare Aufgaben für die ganzheitliche Persönlichkeitsbildung junger Menschen. Dem Sport kommt eine wichtige Vorbild- und Lehrfunktion im Bereich der Integration und demokratischen Grundbildung zu. Für das herausragende gesellschaftliche Engagement des Sports spricht nicht zuletzt, dass die Sportvereine eng mit Schulen, Kindergärten, Unternehmen, Krankenkassen oder anderen öffentlichen Institutionen zusammenarbeiten. Um allen Bürgern den Zugang zum Sport zu ermöglichen, sind adäquate Sportstätten in ausreichender Anzahl Grundvoraussetzung. Ein für alle zugängliches und umfangreiches Sportangebot ist – vor allen Dingen in Großstädten und Ballungsgebieten – nur durch die Verfügbarkeit von ganzjährig nutzbaren Sportanlagen zu gewährleisten. Kunststoffrasenplätze spielen hierbei, insbesondere für den Fußball, eine wichtige Rolle, da sie eine intensivere Nutzung als Naturrasen- oder Tennenplätze erlauben. Allein mit Naturrasen- und Tennenplätzen lässt sich der derzeitige Trainings- und Spielbetrieb, insbesondere bei den Kinder- und Jugendmannschaften, nicht aufrechterhalten. Ein Kunststoffrasenplatz ersetzt etwa 2,5 Naturrasenplätze (DFBnet). Auf weniger als 10 Prozent der Naturrasenplätze finden an Wochenenden mehr als 2 Spiele statt. Bei Kunststoffrasenplätzen finden hingegen bei über 40 Prozent der Plätze mehr als 2 Spiele statt. Weniger als 10 Prozent der Naturrasenplätze wird an einem Wochenende mehr als 150 Minuten genutzt. Bei Kunstrasenplätzen werden hingegen etwa 35 Prozent an einem Wochenende mehr als 150 Minuten genutzt. 27.773 Spielstätten in Deutschland (ca. 70 Prozent) werden von Sportvereinen genutzt. Ein Drittel der Kunstrasenplätze werden von 2 oder mehr Vereinen mit alle ihren Jugend- und Seniorenmannschaften benutzt. Etwas über ein Drittel aller Naturrasenplätze wird von mehr als 5 Mannschaften bespielt. Bei Kunstrasenplätzen werden fast drei Viertel (72 Prozent) von mehr als 5 Mannschaften genutzt. Etwa 10 Prozent aller Naturrasenplätze wird von mehr als 10 Mannschaften bespielt. Bei Kunststoffrasenplätzen sind es ca. 41 Prozent der Plätze, die von mehr als 10 Mannschaften genutzt. Nur 1 Prozent aller Naturrasenplätze wird von mehr als 15 Mannschaften bespielt. Bei Kunstrasenplätzen beträgt der Anteil immerhin noch knapp 18 Prozent. Etwa 6 Prozent werden sogar von über 20 Mannschaften bespielt. Je größer die Vereinsgröße (insbesondere Anzahl der Mannschaften), desto höher ist der Anteil der Vereine, die auch eine Spielstätte vom Typ Kunstrasen haben.  Ein Verbot des Inverkehrbringens von Kunststoffgranulaten als Füllstoff in Kunststoffrasensystemen direkt bei Inkrafttreten der Beschränkung wäre daher unverhältnismäßig. Es würde zu hohen, unerwarteten Umstellungskosten und Mehrkosten für Vereine und Kommunen führen, wodurch dem gemeinwohlorientierten Sport Mittel entzogen würden. Bei fehlender Finanzierbarkeit dieser Mehrkosten ist zudem von einer Schließung vieler Sportplätzen auszugehen, wodurch das Sportangebot in Schulen und Vereinen stark leiden würde. Gerade auf Vereinsebene stellt ein solch außerordentlicher Kosten-punkt ein großes finanzielles Risiko dar, dass das sportliche und gesellschaftliche Gesamtangebot des Vereins gefährden kann. Eine Beschränkung ohne Übergangsfristen, die eine mittelfristige Umstellung und Kostenstreckung erlauben, würde das Breitensportangebot in Deutschland sehr negativ beeinflussen.  Im Hinblick auf den Beschränkungsvorschlag der ECHA gemäß Anhang XV der REACH-Verordnung spricht sich der DFB daher für eine angemessene Übergangsfrist von mindestens sechs Jahren bis zu einem vollständigen Inverkehrbringungsverbot des Kunststoffgranulats zur Verwendung in neuen Kunststoffrasensystemen sowie für die Umstellung bestehender Flächen aus. |
| Fußball-Verband Mittelrhein e.V.  (National NGO, Germany) | **Date:** 2019/05/15  **Content:**  Environmental emissions;  Information on costs;  Other socio economic analysis (SEA) issues;  Transitional period  **Attachment:**   | **Comment:**  - |
|  |  | **Answer to specific info request 2:**  a.: Nach neueren Erkenntnissen werden zwischen 0,25 t/a und 5 t/a in Deutschland an Befüllungsgranulat pro Kunststoffrasenfläche verwendet (Fraunhofer 2018, S. 11). Das entspricht einer Gesamtmenge von ca. 7.500 bis 9.900 t/a.  b.: Nach dem aktuellen Forschungsstand besteht nach Kenntnis des DFB ein hohes Maß an Unsicherheit darüber, wie und in welchen Mengen das als Mikroplastik definierte Granulat auf Sportplätzen in die Umwelt freigesetzt wird. Nach den uns zur Verfügung stehen-den Informationen gibt es große Unterschiede bei der Einschätzung der Menge an Mikroplastiken, die in den einzelnen Mitgliedstaaten oder in der EU/EWR als Füllmaterial für Kunstrasen verwendet wird. Insbesondere Umfang und Methodologie der Forschung in diesem Bereich sind bisher noch wenig standardisiert und nachvollziehbar. Der DFB geht davon aus, dass der Anteil des Eintrags von Mikroplastik über Kunststoffrasenplätze je nach Mitgliedstaat ca. 1 bis 3 Prozent im Verhältnis zum Gesamteintrag beträgt. Demnach ist der Umwelteintrag verglichen mit anderen Hauptquellen relativ gering (Europäische Kommission 2018, ii)).  c.: Gezielte Risikomanagementmaßnahmen können die Freisetzung von Füllstoffen in die Umwelt bereits signifikant vermindern. Technische Maßnahmen zur Zurückhaltung eines Materialaustrags vor Ort (z.B. Rinnenfilter mit Sedimentationsstrecken an Abläufen, Schmutzfangmatten, Schuhbürsten am Ausgang) und organisatorische Maßnahmen beim Betrieb der Sportplätze (z.B. regelmäßige Reinigung der Spielfeldränder, Auffangsiebe) können zu einer starken Verringerung des Austrags von Mikroplastik beitragen.  Neben dem häufig genutzten Kunststoffgranulat existieren für Kunststoffrasensysteme alternative Füllstoffe, die in Teilen auch bereits beim Betrieb von Sportanlagen genutzt werden. So werden in Deutschland aktuell Kunststoffrasenplätze teilweise mit Sand und/oder Kork verfüllt. Zudem gibt es auch Kunststoffrasensysteme, die ohne elastischen Füllstoff betrieben werden können.  Es existieren bisher allerdings nur wenige belastbare Studien darüber, wie sich diese Alternativen qualitäts- und kostenmäßig (z.B. hinsichtlich der Bespielbarkeit und Lebensdauer) vergleichen lassen. Zudem müsste untersucht werden, ob und wie sich die Bespielbar-keit oder das Verletzungsrisiko der alternativ befüllten Kunststoffrasenflächen bei den verschiedenen Alternativfüllungen verändert (Plan Miljø Studie 2017). Es bedarf daher dringend weiterer wissenschaftlicher Expertise zur Praxistauglichkeit alternativer organischer Füllstoffe und zur sportartspezifischen Eignung von Kunststoffrasenplätzen, die ohne Füllstoffe auskommen. Sowohl eine wissenschaftliche Folgenabschätzung als auch die dringend erforderliche Entwicklung alternativer Füllstoffe durch die Industrie sind eine zentrale Forderung der von der Thematik betroffenen Sportverbände in Deutschland. Sie vertreten die Meinung, dass die Maßnahmen, die ein Verbot des Kunststoffgranulats verursachen würden, nicht kurzfristig umsetzbar sind und Alternativen nur mittel- bis langfristig erarbeitet und bereitgestellt werden können.  d.: In Deutschland gibt es ca. 5.000 für den Fußballspielbetrieb gemeldete Kunststoffrasenplätze (DFBnet), sowie ca. 1.000 DFB-Minispielfelder. Jährlich werden in Deutschland ca. 300 Kunststoffrasenplätze neu gebaut, sowie 150 Kunststoffrasenplätze von Grund auf erneuert. Hinsichtlich der bestehenden Plätze dürfte eine Umstellung auf alternative Füllstoffe notwendig sein. Hierfür halten die Sportanlagenbetreiber (Kommunen oder Vereine) Mittel für Sportstättenbau und -sanierung vor, die bei einem vollständigen Ver-bot und einer Verwendung alternativer Füllstoffe deutlich höher ausfallen würden. Laut eigener Berechnungen belaufen sich die jährlichen Mehrkosten deutschlandweit auf einen hohen einstelligen Millionenbetrag. Die insgesamt zu erwartenden Kosten eines Verbotes können aufgrund fehlender Kenntnisse über geeignete alternative Füllstoffe (Geeignetheit, Verfügbarkeit) derzeit nicht seriös beziffert werden. Auf Grundlage aktueller Daten zum Bau von Kunststoffrasenplätzen dürfte der Gesamtbetrag für den Austausch des Füllstoffes der Kunststoffrasensysteme im hohen zweistelligen Millionenbereich (bis zu 90 Mio. EUR) liegen, wobei zur Präzisierung dieses Schätzwertes vertiefte Analysen erforderlich sind. Die Kosten für eine Umsetzung gezielter Risikomanagementmaßnahmen zur Zurückhaltung des Materialaustrags dürften nach Schätzungen und je nach Umfang der Maßnahmen pro Kunststoffrasensystem bei 3.000 bis 10.000 EUR liegen.  e.: Der gemeinwohlorientierte Sport ist die größte zivilgesellschaftliche Bewegung in Deutschland und Europa. In Deutschland engagieren sich knapp acht Millionen Bürger freiwillig und ehrenamtlich im Sport. Das entspricht einer jährlichen Wertschöpfung und einem Wohlfahrtsgewinn allein in Deutschland von ca. 6,7 Milliarden Euro. Vergleichbare Zahlen lassen sich auch für die gesamte EU feststellen. In den EU-Mitgliedstaaten engagieren sich im Jahre 2010 zwischen 92 und 94 Millionen Menschen freiwillig für Ziele des Gemeinwohls, davon die meisten im Sport (ca. 35 bis 40 Prozent aller freiwillig Tätigen in der EU) (Europäische Kommission 2010).  Der Sport schafft ein strukturiertes, an die gesamte Bevölkerung gerichtetes und für alle offenes Bewegungs- und Sportangebot, durch das wichtige soziale und gesundheitsfördernde Funktionen in der Gesellschaft erfüllt werden. Sportvereine in Deutschland zählen zehn Millionen Mitgliedschaften im Kinder- und Jugendalter (DOSB-Bestandserhebung 2018), allein im DFB liegt diese Zahl bei 2,1 Millionen (DFB-Mitgliederstatistik 2018). Damit sind Sportvereine die wichtigste Anlaufstelle für Kinder und Jugendliche außerhalb der Schule und übernehmen unverzichtbare Aufgaben für die ganzheitliche Persönlichkeitsbildung junger Menschen. Dem Sport kommt eine wichtige Vorbild- und Lehrfunktion im Bereich der Integration und demokratischen Grundbildung zu. Für das herausragende gesellschaftliche Engagement des Sports spricht nicht zuletzt, dass die Sportvereine eng mit Schulen, Kindergärten, Unternehmen, Krankenkassen oder anderen öffentlichen Institutionen zusammenarbeiten. Um allen Bürgern den Zugang zum Sport zu ermöglichen, sind adäquate Sportstätten in ausreichender Anzahl Grundvoraussetzung. Ein für alle zugängliches und umfangreiches Sportangebot ist – vor allen Dingen in Großstädten und Ballungsgebieten – nur durch die Verfügbarkeit von ganzjährig nutzbaren Sportanlagen zu gewährleisten. Kunststoffrasenplätze spielen hierbei, insbesondere für den Fußball, eine wichtige Rolle, da sie eine intensivere Nutzung als Naturrasen- oder Tennenplätze erlauben. Allein mit Naturrasen- und Tennenplätzen lässt sich der derzeitige Trainings- und Spielbetrieb, insbesondere bei den Kinder- und Jugendmannschaften, nicht aufrechterhalten. Ein Kunststoffrasenplatz ersetzt etwa 2,5 Naturrasenplätze (DFBnet). Auf weniger als 10 Prozent der Naturrasenplätze finden an Wochenenden mehr als 2 Spiele statt. Bei Kunststoffrasenplätzen finden hingegen bei über 40 Prozent der Plätze mehr als 2 Spiele statt. Weniger als 10 Prozent der Naturrasenplätze wird an einem Wochenende mehr als 150 Minuten genutzt. Bei Kunstrasenplätzen werden hingegen etwa 35 Prozent an einem Wochenende mehr als 150 Minuten genutzt. 27.773 Spielstätten in Deutschland (ca. 70 Prozent) werden von Sportvereinen genutzt. Im Verbandsgebiet des FVM werden 360 Kunstrasenplätze genutzt. Ein Drittel der Kunstrasenplätze werden von 2 oder mehr Vereinen mit alle ihren Jugend- und Seniorenmannschaften benutzt. Etwas über ein Drittel aller Naturrasenplätze wird von mehr als 5 Mannschaften bespielt. Bei Kunstrasenplätzen werden fast drei Viertel (72 Prozent) von mehr als 5 Mannschaften genutzt. Etwa 10 Prozent aller Naturrasenplätze wird von mehr als 10 Mannschaften bespielt. Bei Kunststoffrasenplätzen sind es ca. 41 Prozent der Plätze, die von mehr als 10 Mannschaften genutzt. Nur 1 Prozent aller Naturrasenplätze wird von mehr als 15 Mannschaften bespielt. Bei Kunstrasenplätzen beträgt der Anteil immerhin noch knapp 18 Prozent. Etwa 6 Prozent werden sogar von über 20 Mannschaften bespielt. Je größer die Vereinsgröße (insbesondere Anzahl der Mannschaften), desto höher ist der Anteil der Vereine, die auch eine Spielstätte vom Typ Kunstrasen haben.  Ein Verbot des Inverkehrbringens von Kunststoffgranulaten als Füllstoff in Kunststoffrasensystemen direkt bei Inkrafttreten der Beschränkung wäre daher unverhältnismäßig. Es würde zu hohen, unerwarteten Umstellungskosten und Mehrkosten für Vereine und Kommunen führen, wodurch dem gemeinwohlorientierten Sport Mittel entzogen würden. Bei fehlender Finanzierbarkeit dieser Mehrkosten ist zudem von einer Schließung vieler Sportplätzen auszugehen, wodurch das Sportangebot in Schulen und Vereinen stark leiden würde. Gerade auf Vereinsebene stellt ein solch außerordentlicher Kosten-punkt ein großes finanzielles Risiko dar, dass das sportliche und gesellschaftliche Gesamtangebot des Vereins gefährden kann. Eine Beschränkung ohne Übergangsfristen, die eine mittelfristige Umstellung und Kostenstreckung erlauben, würde das Breitensportangebot in Deutschland sehr negativ beeinflussen.  Im Hinblick auf den Beschränkungsvorschlag der ECHA gemäß Anhang XV der REACH-Verordnung spricht sich der DFB daher für eine angemessene Übergangsfrist von mindestens sechs Jahren bis zu einem vollständigen Inverkehrbringungsverbot des Kunststoffgranulats zur Verwendung in neuen Kunststoffrasensystemen sowie für die Umstellung bestehender Flächen aus. |
|  |  |  |
| Thüringer Fußball-Verband e.V. (National NGO, Germany) | **Date:** 2019/05/15  **Content:**  Scope or restriction option analysis;  Environmental emissions;  Information on costs;  Other socio economic analysis (SEA) issues;  Transitional period | **Comment:**  - |
|  |  | **Answer to specific info request 2:**  a.: Nach neueren Erkenntnissen werden zwischen 0,25 t/a und 5 t/a in Deutschland an Befüllungsgranulat pro Kunststoffrasenfläche verwendet (Fraunhofer 2018, S. 11). Das entspricht einer Gesamtmenge von ca. 7.500 bis 9.900 t/a.  b.: Nach dem aktuellen Forschungsstand besteht nach Kenntnis des DFB ein hohes Maß an Unsicherheit darüber, wie und in welchen Mengen das als Mikroplastik definierte Granulat auf Sportplätzen in die Umwelt freigesetzt wird. Nach den uns zur Verfügung stehen-den Informationen gibt es große Unterschiede bei der Einschätzung der Menge an Mikroplastiken, die in den einzelnen Mitgliedstaaten oder in der EU/EWR als Füllmaterial für Kunstrasen verwendet wird. Insbesondere Umfang und Methodologie der Forschung in diesem Bereich sind bisher noch wenig standardisiert und nachvollziehbar. Der DFB geht davon aus, dass der Anteil des Eintrags von Mikroplastik über Kunststoffrasenplätze je nach Mitgliedstaat ca. 1 bis 3 Prozent im Verhältnis zum Gesamteintrag beträgt. Demnach ist der Umwelteintrag verglichen mit anderen Hauptquellen relativ gering (Europäische Kommission 2018, ii)).  c.: Gezielte Risikomanagementmaßnahmen können die Freisetzung von Füllstoffen in die Umwelt bereits signifikant vermindern. Technische Maßnahmen zur Zurückhaltung eines Materialaustrags vor Ort (z.B. Rinnenfilter mit Sedimentationsstrecken an Abläufen, Schmutzfangmatten, Schuhbürsten am Ausgang) und organisatorische Maßnahmen beim Betrieb der Sportplätze (z.B. regelmäßige Reinigung der Spielfeldränder, Auffangsiebe) können zu einer starken Verringerung des Austrags von Mikroplastik beitragen.  Neben dem häufig genutzten Kunststoffgranulat existieren für Kunststoffrasensysteme alternative Füllstoffe, die in Teilen auch bereits beim Betrieb von Sportanlagen genutzt werden. So werden in Deutschland aktuell Kunststoffrasenplätze teilweise mit Sand und/oder Kork verfüllt. Zudem gibt es auch Kunststoffrasensysteme, die ohne elastischen Füllstoff betrieben werden können.  Es existieren bisher allerdings nur wenige belastbare Studien darüber, wie sich diese Alternativen qualitäts- und kostenmäßig (z.B. hinsichtlich der Bespielbarkeit und Lebensdauer) vergleichen lassen. Zudem müsste untersucht werden, ob und wie sich die Bespielbar-keit oder das Verletzungsrisiko der alternativ befüllten Kunststoffrasenflächen bei den verschiedenen Alternativfüllungen verändert (Plan Miljø Studie 2017). Es bedarf daher dringend weiterer wissenschaftlicher Expertise zur Praxistauglichkeit alternativer organischer Füllstoffe und zur sportartspezifischen Eignung von Kunststoffrasenplätzen, die ohne Füllstoffe auskommen. Sowohl eine wissenschaftliche Folgenabschätzung als auch die dringend erforderliche Entwicklung alternativer Füllstoffe durch die Industrie sind eine zentrale Forderung der von der Thematik betroffenen Sportverbände in Deutschland. Sie vertreten die Meinung, dass die Maßnahmen, die ein Verbot des Kunststoffgranulats verursachen würden, nicht kurzfristig umsetzbar sind und Alternativen nur mittel- bis langfristig erarbeitet und bereitgestellt werden können.  d.: In Deutschland gibt es ca. 5.000 für den Fußballspielbetrieb gemeldete Kunststoffrasenplätze (DFBnet), sowie ca. 1.000 DFB-Minispielfelder. Jährlich werden in Deutschland ca. 300 Kunststoffrasenplätze neu gebaut, sowie 150 Kunststoffrasenplätze von Grund auf erneuert. Hinsichtlich der bestehenden Plätze dürfte eine Umstellung auf alternative Füllstoffe notwendig sein. Hierfür halten die Sportanlagenbetreiber (Kommunen oder Vereine) Mittel für Sportstättenbau und -sanierung vor, die bei einem vollständigen Ver-bot und einer Verwendung alternativer Füllstoffe deutlich höher ausfallen würden. Laut eigener Berechnungen belaufen sich die jährlichen Mehrkosten deutschlandweit auf einen hohen einstelligen Millionenbetrag. Die insgesamt zu erwartenden Kosten eines Verbotes können aufgrund fehlender Kenntnisse über geeignete alternative Füllstoffe (Geeignetheit, Verfügbarkeit) derzeit nicht seriös beziffert werden. Auf Grundlage aktueller Daten zum Bau von Kunststoffrasenplätzen dürfte der Gesamtbetrag für den Austausch des Füllstoffes der Kunststoffrasensysteme im hohen zweistelligen Millionenbereich (bis zu 90 Mio. EUR) liegen, wobei zur Präzisierung dieses Schätzwertes vertiefte Analysen erforderlich sind. Die Kosten für eine Umsetzung gezielter Risikomanagementmaßnahmen zur Zurückhaltung des Materialaustrags dürften nach Schätzungen und je nach Umfang der Maßnahmen pro Kunststoffrasensystem bei 3.000 bis 10.000 EUR liegen.  e.: Der gemeinwohlorientierte Sport ist die größte zivilgesellschaftliche Bewegung in Deutschland und Europa. In Deutschland engagieren sich knapp acht Millionen Bürger freiwillig und ehrenamtlich im Sport. Das entspricht einer jährlichen Wertschöpfung und einem Wohlfahrtsgewinn allein in Deutschland von ca. 6,7 Milliarden Euro. Vergleichbare Zahlen lassen sich auch für die gesamte EU feststellen. In den EU-Mitgliedstaaten engagieren sich im Jahre 2010 zwischen 92 und 94 Millionen Menschen freiwillig für Ziele des Gemeinwohls, davon die meisten im Sport (ca. 35 bis 40 Prozent aller freiwillig Tätigen in der EU) (Europäische Kommission 2010).  Der Sport schafft ein strukturiertes, an die gesamte Bevölkerung gerichtetes und für alle offenes Bewegungs- und Sportangebot, durch das wichtige soziale und gesundheitsfördernde Funktionen in der Gesellschaft erfüllt werden. Sportvereine in Deutschland zählen zehn Millionen Mitgliedschaften im Kinder- und Jugendalter (DOSB-Bestandserhebung 2018), allein im DFB liegt diese Zahl bei 2,1 Millionen (DFB-Mitgliederstatistik 2018). Damit sind Sportvereine die wichtigste Anlaufstelle für Kinder und Jugendliche außerhalb der Schule und übernehmen unverzichtbare Aufgaben für die ganzheitliche Persönlichkeitsbildung junger Menschen. Dem Sport kommt eine wichtige Vorbild- und Lehrfunktion im Bereich der Integration und demokratischen Grundbildung zu. Für das herausragende gesellschaftliche Engagement des Sports spricht nicht zuletzt, dass die Sportvereine eng mit Schulen, Kindergärten, Unternehmen, Krankenkassen oder anderen öffentlichen Institutionen zusammenarbeiten. Um allen Bürgern den Zugang zum Sport zu ermöglichen, sind adäquate Sportstätten in ausreichender Anzahl Grundvoraussetzung. Ein für alle zugängliches und umfangreiches Sportangebot ist – vor allen Dingen in Großstädten und Ballungsgebieten – nur durch die Verfügbarkeit von ganzjährig nutzbaren Sportanlagen zu gewährleisten. Kunststoffrasenplätze spielen hierbei, insbesondere für den Fußball, eine wichtige Rolle, da sie eine intensivere Nutzung als Naturrasen- oder Tennenplätze erlauben. Allein mit Naturrasen- und Tennenplätzen lässt sich der derzeitige Trainings- und Spielbetrieb, insbesondere bei den Kinder- und Jugendmannschaften, nicht aufrechterhalten. Ein Kunststoffrasenplatz ersetzt etwa 2,5 Naturrasenplätze (DFBnet). Auf weniger als 10 Prozent der Naturrasenplätze finden an Wochenenden mehr als 2 Spiele statt. Bei Kunststoffrasenplätzen finden hingegen bei über 40 Prozent der Plätze mehr als 2 Spiele statt. Weniger als 10 Prozent der Naturrasenplätze wird an einem Wochenende mehr als 150 Minuten genutzt. Bei Kunstrasenplätzen werden hingegen etwa 35 Prozent an einem Wochenende mehr als 150 Minuten genutzt. 27.773 Spielstätten in Deutschland (ca. 70 Prozent) werden von Sportvereinen genutzt. Ein Drittel der Kunstrasenplätze werden von 2 oder mehr Vereinen mit alle ihren Jugend- und Seniorenmannschaften benutzt. Etwas über ein Drittel aller Naturrasenplätze wird von mehr als 5 Mannschaften bespielt. Bei Kunstrasenplätzen werden fast drei Viertel (72 Prozent) von mehr als 5 Mannschaften genutzt. Etwa 10 Prozent aller Naturrasenplätze wird von mehr als 10 Mannschaften bespielt. Bei Kunststoffrasenplätzen sind es ca. 41 Prozent der Plätze, die von mehr als 10 Mannschaften genutzt. Nur 1 Prozent aller Naturrasenplätze wird von mehr als 15 Mannschaften bespielt. Bei Kunstrasenplätzen beträgt der Anteil immerhin noch knapp 18 Prozent. Etwa 6 Prozent werden sogar von über 20 Mannschaften bespielt. Je größer die Vereinsgröße (insbesondere Anzahl der Mannschaften), desto höher ist der Anteil der Vereine, die auch eine Spielstätte vom Typ Kunstrasen haben.  Ein Verbot des Inverkehrbringens von Kunststoffgranulaten als Füllstoff in Kunststoffrasensystemen direkt bei Inkrafttreten der Beschränkung wäre daher unverhältnismäßig. Es würde zu hohen, unerwarteten Umstellungskosten und Mehrkosten für Vereine und Kommunen führen, wodurch dem gemeinwohlorientierten Sport Mittel entzogen würden. Bei fehlender Finanzierbarkeit dieser Mehrkosten ist zudem von einer Schließung vieler Sportplätzen auszugehen, wodurch das Sportangebot in Schulen und Vereinen stark leiden würde. Gerade auf Vereinsebene stellt ein solch außerordentlicher Kosten-punkt ein großes finanzielles Risiko dar, dass das sportliche und gesellschaftliche Gesamtangebot des Vereins gefährden kann. Eine Beschränkung ohne Übergangsfristen, die eine mittelfristige Umstellung und Kostenstreckung erlauben, würde das Breitensportangebot in Deutschland sehr negativ beeinflussen.  Im Hinblick auf den Beschränkungsvorschlag der ECHA gemäß Anhang XV der REACH-Verordnung spricht sich der DFB daher für eine angemessene Übergangsfrist von mindestens sechs Jahren bis zu einem vollständigen Inverkehrbringungsverbot des Kunststoffgranulats zur Verwendung in neuen Kunststoffrasensystemen sowie für die Umstellung bestehender Flächen aus. |
| Württembergischer Fußballverband  (National NGO, Germany) | **Date:** 2019/05/14  **Content:**  Scope or restriction option analysis;  Environmental emissions;  Information on costs;  Other socio economic analysis (SEA) issues;  Transitional period  **Attachment:**   | **Comment:** |
|  |  | **Answer to specific info request 1:** |
|  |  | **Answer to specific info request 2:**  a.: Nach neueren Erkenntnissen werden zwischen 0,25 t/a und 5 t/a in Deutschland an Befüllungsgranulat pro Kunststoffrasenfläche verwendet (Fraunhofer 2018, S. 11). Das entspricht einer Gesamtmenge von ca. 7.500 bis 9.900 t/a.  b.: Nach dem aktuellen Forschungsstand besteht nach Kenntnis des DFB ein hohes Maß an Unsicherheit darüber, wie und in welchen Mengen das als Mikroplastik definierte Granulat auf Sportplätzen in die Umwelt freigesetzt wird. Nach den uns zur Verfügung stehen-den Informationen gibt es große Unterschiede bei der Einschätzung der Menge an Mikroplastiken, die in den einzelnen Mitgliedstaaten oder in der EU/EWR als Füllmaterial für Kunstrasen verwendet wird. Insbesondere Umfang und Methodologie der Forschung in diesem Bereich sind bisher noch wenig standardisiert und nachvollziehbar. Der DFB geht davon aus, dass der Anteil des Eintrags von Mikroplastik über Kunststoffrasenplätze je nach Mitgliedstaat ca. 1 bis 3 Prozent im Verhältnis zum Gesamteintrag beträgt. Demnach ist der Umwelteintrag verglichen mit anderen Hauptquellen relativ gering (Europäische Kommission 2018, ii)).  c.: Gezielte Risikomanagementmaßnahmen können die Freisetzung von Füllstoffen in die Umwelt bereits signifikant vermindern. Technische Maßnahmen zur Zurückhaltung eines Materialaustrags vor Ort (z.B. Rinnenfilter mit Sedimentationsstrecken an Abläufen, Schmutzfangmatten, Schuhbürsten am Ausgang) und organisatorische Maßnahmen beim Betrieb der Sportplätze (z.B. regelmäßige Reinigung der Spielfeldränder, Auffangsiebe) können zu einer starken Verringerung des Austrags von Mikroplastik beitragen.  Neben dem häufig genutzten Kunststoffgranulat existieren für Kunststoffrasensysteme alternative Füllstoffe, die in Teilen auch bereits beim Betrieb von Sportanlagen genutzt werden. So werden in Deutschland aktuell Kunststoffrasenplätze teilweise mit Sand und/oder Kork verfüllt. Zudem gibt es auch Kunststoffrasensysteme, die ohne elastischen Füllstoff betrieben werden können.  Es existieren bisher allerdings nur wenige belastbare Studien darüber, wie sich diese Alternativen qualitäts- und kostenmäßig (z.B. hinsichtlich der Bespielbarkeit und Lebensdauer) vergleichen lassen. Zudem müsste untersucht werden, ob und wie sich die Bespielbar-keit oder das Verletzungsrisiko der alternativ befüllten Kunststoffrasenflächen bei den verschiedenen Alternativfüllungen verändert (Plan Miljø Studie 2017). Es bedarf daher dringend weiterer wissenschaftlicher Expertise zur Praxistauglichkeit alternativer organischer Füllstoffe und zur sportartspezifischen Eignung von Kunststoffrasenplätzen, die ohne Füllstoffe auskommen. Sowohl eine wissenschaftliche Folgenabschätzung als auch die dringend erforderliche Entwicklung alternativer Füllstoffe durch die Industrie sind eine zentrale Forderung der von der Thematik betroffenen Sportverbände in Deutschland. Sie vertreten die Meinung, dass die Maßnahmen, die ein Verbot des Kunststoffgranulats verursachen würden, nicht kurzfristig umsetzbar sind und Alternativen nur mittel- bis langfristig erarbeitet und bereitgestellt werden können.  d.: In Deutschland gibt es ca. 5.000 für den Fußballspielbetrieb gemeldete Kunststoffrasenplätze (DFBnet), sowie ca. 1.000 DFB-Minispielfelder. Jährlich werden in Deutschland ca. 300 Kunststoffrasenplätze neu gebaut, sowie 150 Kunststoffrasenplätze von Grund auf erneuert. Hinsichtlich der bestehenden Plätze dürfte eine Umstellung auf alternative Füllstoffe notwendig sein. Hierfür halten die Sportanlagenbetreiber (Kommunen oder Vereine) Mittel für Sportstättenbau und -sanierung vor, die bei einem vollständigen Ver-bot und einer Verwendung alternativer Füllstoffe deutlich höher ausfallen würden. Laut eigener Berechnungen belaufen sich die jährlichen Mehrkosten deutschlandweit auf einen hohen einstelligen Millionenbetrag. Die insgesamt zu erwartenden Kosten eines Verbotes können aufgrund fehlender Kenntnisse über geeignete alternative Füllstoffe (Geeignetheit, Verfügbarkeit) derzeit nicht seriös beziffert werden. Auf Grundlage aktueller Daten zum Bau von Kunststoffrasenplätzen dürfte der Gesamtbetrag für den Austausch des Füllstoffes der Kunststoffrasensysteme im hohen zweistelligen Millionenbereich (bis zu 90 Mio. EUR) liegen, wobei zur Präzisierung dieses Schätzwertes vertiefte Analysen erforderlich sind. Die Kosten für eine Umsetzung gezielter Risikomanagementmaßnahmen zur Zurückhaltung des Materialaustrags dürften nach Schätzungen und je nach Umfang der Maßnahmen pro Kunststoffrasensystem bei 3.000 bis 10.000 EUR liegen.  e.: Der gemeinwohlorientierte Sport ist die größte zivilgesellschaftliche Bewegung in Deutschland und Europa. In Deutschland engagieren sich knapp acht Millionen Bürger freiwillig und ehrenamtlich im Sport. Das entspricht einer jährlichen Wertschöpfung und einem Wohlfahrtsgewinn allein in Deutschland von ca. 6,7 Milliarden Euro. Vergleichbare Zahlen lassen sich auch für die gesamte EU feststellen. In den EU-Mitgliedstaaten engagieren sich im Jahre 2010 zwischen 92 und 94 Millionen Menschen freiwillig für Ziele des Gemeinwohls, davon die meisten im Sport (ca. 35 bis 40 Prozent aller freiwillig Tätigen in der EU) (Europäische Kommission 2010).  Der Sport schafft ein strukturiertes, an die gesamte Bevölkerung gerichtetes und für alle offenes Bewegungs- und Sportangebot, durch das wichtige soziale und gesundheitsfördernde Funktionen in der Gesellschaft erfüllt werden. Sportvereine in Deutschland zählen zehn Millionen Mitgliedschaften im Kinder- und Jugendalter (DOSB-Bestandserhebung 2018), allein im DFB liegt diese Zahl bei 2,1 Millionen (DFB-Mitgliederstatistik 2018). Damit sind Sportvereine die wichtigste Anlaufstelle für Kinder und Jugendliche außerhalb der Schule und übernehmen unverzichtbare Aufgaben für die ganzheitliche Persönlichkeitsbildung junger Menschen. Dem Sport kommt eine wichtige Vorbild- und Lehrfunktion im Bereich der Integration und demokratischen Grundbildung zu. Für das herausragende gesellschaftliche Engagement des Sports spricht nicht zuletzt, dass die Sportvereine eng mit Schulen, Kindergärten, Unternehmen, Krankenkassen oder anderen öffentlichen Institutionen zusammenarbeiten. Um allen Bürgern den Zugang zum Sport zu ermöglichen, sind adäquate Sportstätten in ausreichender Anzahl Grundvoraussetzung. Ein für alle zugängliches und umfangreiches Sportangebot ist – vor allen Dingen in Großstädten und Ballungsgebieten – nur durch die Verfügbarkeit von ganzjährig nutzbaren Sportanlagen zu gewährleisten. Kunststoffrasenplätze spielen hierbei, insbesondere für den Fußball, eine wichtige Rolle, da sie eine intensivere Nutzung als Naturrasen- oder Tennenplätze erlauben. Allein mit Naturrasen- und Tennenplätzen lässt sich der derzeitige Trainings- und Spielbetrieb, insbesondere bei den Kinder- und Jugendmannschaften, nicht aufrechterhalten. Ein Kunststoffrasenplatz ersetzt etwa 2,5 Naturrasenplätze (DFBnet). Auf weniger als 10 Prozent der Naturrasenplätze finden an Wochenenden mehr als 2 Spiele statt. Bei Kunststoffrasenplätzen finden hingegen bei über 40 Prozent der Plätze mehr als 2 Spiele statt. Weniger als 10 Prozent der Naturrasenplätze wird an einem Wochenende mehr als 150 Minuten genutzt. Bei Kunstrasenplätzen werden hingegen etwa 35 Prozent an einem Wochenende mehr als 150 Minuten genutzt. 27.773 Spielstätten in Deutschland (ca. 70 Prozent) werden von Sportvereinen genutzt. Ein Drittel der Kunstrasenplätze werden von 2 oder mehr Vereinen mit alle ihren Jugend- und Seniorenmannschaften benutzt. Etwas über ein Drittel aller Naturrasenplätze wird von mehr als 5 Mannschaften bespielt. Bei Kunstrasenplätzen werden fast drei Viertel (72 Prozent) von mehr als 5 Mannschaften genutzt. Etwa 10 Prozent aller Naturrasenplätze wird von mehr als 10 Mannschaften bespielt. Bei Kunststoffrasenplätzen sind es ca. 41 Prozent der Plätze, die von mehr als 10 Mannschaften genutzt. Nur 1 Prozent aller Naturrasenplätze wird von mehr als 15 Mannschaften bespielt. Bei Kunstrasenplätzen beträgt der Anteil immerhin noch knapp 18 Prozent. Etwa 6 Prozent werden sogar von über 20 Mannschaften bespielt. Je größer die Vereinsgröße (insbesondere Anzahl der Mannschaften), desto höher ist der Anteil der Vereine, die auch eine Spielstätte vom Typ Kunstrasen haben. Zur Aufrechterhaltung des Spielbetriebs für unsere ca. 13.000 Mannschaften in rund 1.600 Vereine sind Kunstrasenplätze von elementarer Bedeutung.  Ein Verbot des Inverkehrbringens von Kunststoffgranulaten als Füllstoff in Kunststoffrasensystemen direkt bei Inkrafttreten der Beschränkung wäre daher unverhältnismäßig. Es würde zu hohen, unerwarteten Umstellungskosten und Mehrkosten für Vereine und Kommunen führen, wodurch dem gemeinwohlorientierten Sport Mittel entzogen würden. Bei fehlender Finanzierbarkeit dieser Mehrkosten ist zudem von einer Schließung vieler Sportplätzen auszugehen, wodurch das Sportangebot in Schulen und Vereinen stark leiden würde. Gerade auf Vereinsebene stellt ein solch außerordentlicher Kosten-punkt ein großes finanzielles Risiko dar, dass das sportliche und gesellschaftliche Gesamtangebot des Vereins gefährden kann. Eine Beschränkung ohne Übergangsfristen, die eine mittelfristige Umstellung und Kostenstreckung erlauben, würde das Breitensportangebot in Deutschland sehr negativ beeinflussen.  Im Hinblick auf den Beschränkungsvorschlag der ECHA gemäß Anhang XV der REACH-Verordnung spricht sich der DFB daher für eine angemessene Übergangsfrist von mindestens sechs Jahren bis zu einem vollständigen Inverkehrbringungsverbot des Kunststoffgranulats zur Verwendung in neuen Kunststoffrasensystemen sowie für die Umstellung bestehender Flächen aus. |
|  |  | **Comment:**  We understand that matting/opacizing microplastics and thickening microplastics are excluded from the scope of this proposal. These are in large part acrylic/metacrylic/styrenic co-polymers.  According to a study from AISE, liquid laudry detergents in Europe in 2017 had a market of 1.600.000 ton this report, pg. 22)  <https://www.aise.eu/documents/document/20160229142408-16-02-01_prep-l2_project_description_final_(3).pdf>  We consider that 0.4% can be a realistic gross estimation of the average presence of matting/opacizing materials in liquid soap;  We also estimate that of that share, on average 40% is composed of microplastics.  Given these figures the total amount of matting/opacizing microplastics going down the pipe every year in Europe can sum up to 2560 Ton. (=1.600.000 * 0.4% * 40%).  As a benchmark, we mention here that UNEP in 2017 has estimated that the total amount of MP released into the environment from cosmetics is 42.000T/y worldwide.  On top of this we must consider that these figures only refer to a relevant amount of home detergents, but do not cover them all: we should add to the figures other detergents that also contain or may contain the same matting/opacizing microplastics, such as furniture detergents. Besides, syntetic thickeners and matting/opacizing microplastics are present in personal care products too.  We would like to stress here that canadian regulation on microplastics clearly defines matting/opacizing microplastics as such  ("Microbeads - A Science summary" July 2015) <http://www.ec.gc.ca/ese-ees/adda4c5f-f397-48d5-ad17-63f989ebd0e5/microbeads_science%20summary_en.pdf>  on the base of a Dutch study ("Review of microplastics in cosmetics", H.A. Leslie, July 2014)  <https://science.vu.nl/en/Images/Plastic_ingredients_in_Cosmetics_07-2014_FINAL_tcm296-409859.pdf>  and bans them (Ban on the manufacturing, import, and placing on the market of any toiletries for cleansing or hygiene that contain microbeads. Entry into Force: 1 July 2018).  There are, on the other hand, several existing alternatives, already widely available and used, to thickeners and matting/opacizing plastics.  For thickeners:  Common salt is used to increase viscosity in formulas containing surfactants such as SLES.  It is worth mentioning here that other natural thickeners used for such purpose are  • clays such as bentonite  • cullulose and modified cellulose (CMC, HMC, HPMC)  • Gums such as Xantham Gum, guar, locust bean)  • Carbohydrates (carragenine, pectine, alginic acid)  • proteines (caseine)  •  Several studies are available on natural thickeners, such as:  <https://www.researchgate.net/publication/292447286_Thickener_choice_-_A_way_to_improve_cosmetics_sensory_properties>  <https://patents.google.com/patent/US20030158324> (paragraph 0018)  For Matting/opacizing microplastics:  Natural alternatives to matting/opacizing microplastics are also present in the market and produced and used by important international companies such as BASF (euperlan)  Other products that are used for this purpose are non-organic pigments such as TiO2, waxes (including emusions of natural bee wax), kaolinite, talcs, fatty alchools and esthers and stearates of fatty alchools (<https://www.berg-schmidt.de/en/Cosmetic/c25_opacifier.php>).  Other patents and pubblications on this topic can be found here:  <https://patents.google.com/patent/US7176171B2/en>  <https://patents.google.com/patent/US5851541A/en>  <https://pubs.acs.org/doi/full/10.1021/bk-2013-1148.ch001?src=recsys> |
| Legambiente (Italy  National NGO) | 2019/05/08  **Content:**  Scope or restriction option analysis |  |

**References**

ECHA, 2019, General Comments and answers to specific information requests, Helsinki: European Chemicals Agency, Link: <https://echa.europa.eu/registry-of-restriction-intentions/-/dislist/details/0b0236e18244cd73> - accessed 28-10-2019
