# Supplementary material for: Stakeholder analysis with regard to a recent European restriction proposal on microplastics
Source: PLoS One. 2020 Jun 22;15(6):e0235062. doi: 10.1371/journal.pone.0235062 (PMC7307934; doi:10.1371/journal.pone.0235062)
Supplement: S20 Table — (DOCX) [file pone.0235062.s021.docx]

S20 Table: Other Contributors-Authorities Annex XV comments

| **Stakeholder** | | **ECHA date** | **Expressed interests/opinion on microplastics Annex XV comments** |
| --- | --- | --- | --- |
| Germany, Other contributor, NA 005-01-22 AA DIN-NABau | | 2019/07/19  **Content:**  Information on alternatives;  Information on costs | **Comment:**  Please see attached comment file for all our comments. |
| Switzerland, Other contributor, (UEFA) UNION OF EUROPEAN FOOTBALL ASSOCIATIONS | | 2019/07/19  **Content:**  Scope or restriction option analysis;  Information on alternatives;  Information on costs;  Other socio economic analysis (SEA) issues;  Transitional period | **Comment:**  More information can be found in the position paper attached. |
| United Kingdom, Other contributor, The English Football Association | | 2019/05/20  **Content:**  Scope or restriction option analysis;  Baseline;  Information on alternatives;  Information on costs;  Information on benefits;  Other socio economic analysis (SEA) issues | **Comment:**  See attached letter |
|  | |  | **Answer to specific info request 2:**  See attached letter |
|  | |  | **Answer to specific info request 5:**  See attached letter |
| Spain, Other contributor, Spanish Professional Football League | | 2019/05/20  **Content:**  Scope or restriction option analysis | **Comment:**  LaLiga fully supports that microplastics and in particular the use of ELT granular in synthetic turf pitches needs to be examined. However, LaLiga believes as well that a gradual approach will accommodate public authorities’ objectives to protect and safeguard the environment and guarantee citizens, and in particular, children’s access to sport facilities.  An immediate prohibition of ELT granular in synthetic turf pitches may have a large social and economic impact that can prove to be disproportionate for all stakeholders involved, including in particular, citizens.  It is a crucial moment now for European, national and regional authorities as well as for manufacturers and sport associations to gather more data and conduct a proper assessment of the advantages and disadvantages of the use of ELT granular in synthetic turf pitches in comparison to the use of other alternatives and an evaluation of the costs thereof.  **Answer to specific info request 2:**  It is fundamental for citizens to have access to good and sustainable sport facilities all year long.  In Spain 3.600 persons (out of 12.000 surveyed persons) practice sports to be in shape and the majority of them (11.160) do it at least once a week, regardless of whether it is holidays or school period of the year.  In these conditions, synthetic turf pitches play an important role as they can be used by citizens in a more intensive manner than natural turf pitches.  Additionally, there are increasingly more players in national University championships. For instance, 251 players took part in football championships in 2017. In the same championships but for rugby 333 players participated.  The use of synthetic turf with granules from ELT has increased in Spain in the last years. 140 pitches of LaLiga’s clubs members use synthetic turf pitch. This type of pitch is increasingly used in other sports like rugby and golf.  According to the latest statistics from the High Council for Sport in Spain, there are 8. 331 football pitches (p. 189), 5. 000 of which with synthetic turf. Additionally, there are 15.637 sports infrastructures dedicated mainly to football (p. 190). Hence the potential number of pitches with synthetic turf in Spain may be higher.  Municipal authorities, schools and universities are increasingly having synthetic turf pitches with ELT granules installed in their recreational areas.  However, we do not dispose of specific information on the number of these spaces and it is difficult to describe the impact on these facilities by the introduction of the prohibition. It is noteworthy that many of these facilities may be owned by public authorities. |
| Germany, Deutscher Landkreistag & Deutscher Städte- und Gemeindebund | | 2019/05/21 | Attachment |
| Germany, Regional or local authority, LandesSportBund Sachsen-Anhalt e.V. | | 2019/05/20  **Content:**  Scope or restriction option analysis;  Environmental emissions;  Information on costs;  Other socio economic analysis (SEA) issues;  Transitional period | **Answer to specific info request 2:**  a.: Nach neueren Erkenntnissen werden zwischen 0,25 t/a und 5 t/a in Deutschland an Befüllungsgranulat pro Kunststoffrasenfläche verwendet (Fraunhofer 2018, S. 11). Das entspricht einer Gesamtmenge von ca. 7.500 bis 9.900 t/a.  b.: Nach dem aktuellen Forschungsstand besteht nach Kenntnis des DFB ein hohes Maß an Unsicherheit darüber, wie und in welchen Mengen das als Mikroplastik definierte Granulat auf Sportplätzen in die Umwelt freigesetzt wird. Nach den uns zur Verfügung stehen-den Informationen gibt es große Unterschiede bei der Einschätzung der Menge an Mikroplastiken, die in den einzelnen Mitgliedstaaten oder in der EU/EWR als Füllmaterial für Kunstrasen verwendet wird. Insbesondere Umfang und Methodologie der Forschung in diesem Bereich sind bisher noch wenig standardisiert und nachvollziehbar. Der DFB geht davon aus, dass der Anteil des Eintrags von Mikroplastik über Kunststoffrasenplätze je nach Mitgliedstaat ca. 1 bis 3 Prozent im Verhältnis zum Gesamteintrag beträgt. Demnach ist der Umwelteintrag verglichen mit anderen Hauptquellen relativ gering (Europäische Kommission 2018, ii)).  c.: Gezielte Risikomanagementmaßnahmen können die Freisetzung von Füllstoffen in die Umwelt bereits signifikant vermindern. Technische Maßnahmen zur Zurückhaltung eines Materialaustrags vor Ort (z.B. Rinnenfilter mit Sedimentationsstrecken an Abläufen, Schmutzfangmatten, Schuhbürsten am Ausgang) und organisatorische Maßnahmen beim Betrieb der Sportplätze (z.B. regelmäßige Reinigung der Spielfeldränder, Auffangsiebe) können zu einer starken Verringerung des Austrags von Mikroplastik beitragen.  Neben dem häufig genutzten Kunststoffgranulat existieren für Kunststoffrasensysteme alternative Füllstoffe, die in Teilen auch bereits beim Betrieb von Sportanlagen genutzt werden. So werden in Deutschland aktuell Kunststoffrasenplätze teilweise mit Sand und/oder Kork verfüllt. Zudem gibt es auch Kunststoffrasensysteme, die ohne elastischen Füllstoff betrieben werden können.  Es existieren bisher allerdings nur wenige belastbare Studien darüber, wie sich diese Alternativen qualitäts- und kostenmäßig (z.B. hinsichtlich der Bespielbarkeit und Lebensdauer) vergleichen lassen. Zudem müsste untersucht werden, ob und wie sich die Bespielbar-keit oder das Verletzungsrisiko der alternativ befüllten Kunststoffrasenflächen bei den verschiedenen Alternativfüllungen verändert (Plan Miljø Studie 2017). Es bedarf daher dringend weiterer wissenschaftlicher Expertise zur Praxistauglichkeit alternativer organischer Füllstoffe und zur sportartspezifischen Eignung von Kunststoffrasenplätzen, die ohne Füllstoffe auskommen. Sowohl eine wissenschaftliche Folgenabschätzung als auch die dringend erforderliche Entwicklung alternativer Füllstoffe durch die Industrie sind eine zentrale Forderung der von der Thematik betroffenen Sportverbände in Deutschland. Sie vertreten die Meinung, dass die Maßnahmen, die ein Verbot des Kunststoffgranulats verursachen würden, nicht kurzfristig umsetzbar sind und Alternativen nur mittel- bis langfristig erarbeitet und bereitgestellt werden können.  d.: In Deutschland gibt es ca. 5.000 für den Fußballspielbetrieb gemeldete Kunststoffrasenplätze (DFBnet), sowie ca. 1.000 DFB-Minispielfelder. Jährlich werden in Deutschland ca. 300 Kunststoffrasenplätze neu gebaut, sowie 150 Kunststoffrasenplätze von Grund auf erneuert. Hinsichtlich der bestehenden Plätze dürfte eine Umstellung auf alternative Füllstoffe notwendig sein. Hierfür halten die Sportanlagenbetreiber (Kommunen oder Vereine) Mittel für Sportstättenbau und -sanierung vor, die bei einem vollständigen Ver-bot und einer Verwendung alternativer Füllstoffe deutlich höher ausfallen würden. Laut eigener Berechnungen belaufen sich die jährlichen Mehrkosten deutschlandweit auf einen hohen einstelligen Millionenbetrag. Die insgesamt zu erwartenden Kosten eines Verbotes können aufgrund fehlender Kenntnisse über geeignete alternative Füllstoffe (Geeignetheit, Verfügbarkeit) derzeit nicht seriös beziffert werden. Auf Grundlage aktueller Daten zum Bau von Kunststoffrasenplätzen dürfte der Gesamtbetrag für den Austausch des Füllstoffes der Kunststoffrasensysteme im hohen zweistelligen Millionenbereich (bis zu 90 Mio. EUR) liegen, wobei zur Präzisierung dieses Schätzwertes vertiefte Analysen erforderlich sind. Die Kosten für eine Umsetzung gezielter Risikomanagementmaßnahmen zur Zurückhaltung des Materialaustrags dürften nach Schätzungen und je nach Umfang der Maßnahmen pro Kunststoffrasensystem bei 3.000 bis 10.000 EUR liegen.  e.: Der gemeinwohlorientierte Sport ist die größte zivilgesellschaftliche Bewegung in Deutschland und Europa. In Sachsen-Anhalt engagieren sich knapp 45.000 Bürger freiwillig und ehrenamtlich im organisierten Sport für rund 355.000 Mitglieder in über 3.000 Sportvereinen.  Der Sport schafft ein strukturiertes, an die gesamte Bevölkerung gerichtetes und für alle offenes Bewegungs- und Sportangebot, durch das wichtige soziale und gesundheitsfördernde Funktionen in der Gesellschaft erfüllt werden. Sportvereine in Sachsen-Anhalt zählen rund 105.000 Mitgliedschaften im Kinder- und Jugendalter (LSB-Bestandserhebung 2019), allein in den Sportarten Fußball und Hockey liegt diese Zahl bei insgesamt ca. 31.500 aktiven Nachwuchssportlern (LSB-Bestandserhebung 2019). Damit sind diese 822 Fußball- und Hockeyvereine die wichtigste Anlaufstelle für Kinder und Jugendliche außerhalb der Schule und übernehmen unverzichtbare Aufgaben für die ganzheitliche Persönlichkeitsbildung junger Menschen. Dem Sport kommt eine wichtige Vorbild- und Lehrfunktion im Bereich der Integration und demokratischen Grundbildung zu. Für das herausragende gesellschaftliche Engagement des Sports spricht nicht zuletzt, dass die Sportvereine eng mit Schulen, Kindergärten, Unternehmen, Krankenkassen oder anderen öffentlichen Institutionen zusammenarbeiten. Um allen Bürgern den Zugang zum Sport zu ermöglichen, sind adäquate Sportstätten in ausreichender Anzahl Grundvoraussetzung. Ein für alle zugängliches und umfangreiches Sportangebot ist nur durch die Verfügbarkeit von ganzjährig nutzbaren Sportanlagen zu gewährleisten. Kunststoffrasenplätze spielen hierbei, insbesondere für den Fußball sowie für den Hockeysport, eine wichtige Rolle, da sie eine intensivere Nutzung als Naturrasen- oder Tennenplätze erlauben. Allein mit Naturrasen- und Tennenplätzen lässt sich der derzeitige Trainings- und Spielbetrieb, insbesondere bei den Kinder- und Jugendmannschaften, nicht aufrechterhalten.  In Sachsen-Anhalt gibt es rund 85 Sportanlagen (zum Teil mit mehreren Spielfeldern) an denen für den Fußball und Hockey Kunststoffrasenplätze vorhanden sind. Insgesamt nutzen Fußball- und Hockeyvereine insgesamt knapp 650 Sportplätze. Die circa 13 % Kunststoffrasenplätze befinden sich schwerpunktmäßig in den beiden Großstädten Magdeburg und Halle (Saale). Hier wäre ein Trainings- und Wettkampfbetrieb etlicher Vereine ohne diese Anlagen undenkbar. An diesen Standorten nutzen die Anlagen nicht nur mehrere Fußballvereine gleichzeitig sondern teilen sich die Sportarten Fußball und Hockey auch den Sportplatz.  Ungeachtet der Tatsache, dass ohne diese Plätze der Trainings- und Wettkampfbedarf nicht auch nur annährend bedient werden könnte, wäre es ebenso unvorstellbar diese Stunden auf neu zu errichtende Naturrasenplätze umzulagern, da die hohe Stundenzahl der Nutzung auf dem Naturbelag nicht möglich ist.  Ein Verbot des Inverkehrbringens von Kunststoffgranulaten als Füllstoff in Kunststoffrasensystemen direkt bei Inkrafttreten der Beschränkung wäre daher unverhältnismäßig. Es würde zu hohen, unerwarteten Umstellungskosten und Mehrkosten für Vereine und Kommunen führen, wodurch dem gemeinwohlorientierten Sport Mittel entzogen würden. Bei fehlender Finanzierbarkeit dieser Mehrkosten ist zudem von einer Schließung vieler Sportplätzen auszugehen, wodurch das Sportangebot in Schulen und Vereinen stark leiden würde. Gerade auf Vereinsebene stellt ein solch außerordentlicher Kostenpunkt ein großes finanzielles Risiko dar, dass das sportliche und gesellschaftliche Gesamtangebot des Vereins gefährden würde. Eine Beschränkung ohne Übergangsfristen, die eine mittelfristige Umstellung und Kostenstreckung erlauben, würde das Sportangebot in Sachsen-Anhalt sehr negativ beeinflussen.  Im Hinblick auf den Beschränkungsvorschlag der ECHA gemäß Anhang XV der REACH-Verordnung spricht sich der LandesSportBund Sachsen-Anhalt daher für eine angemessene Übergangsfrist von mindestens sechs Jahren bis zu einem vollständigen Inverkehrbringungsverbot des Kunststoffgranulats zur Verwendung in neuen Kunststoffrasensystemen sowie für die Umstellung bestehender Flächen aus. |
| Sweden, MemberState, The Swedish Medical Products Agency | | 2019/05/20  **Content:**  Scope or restriction option analysis;  Baseline;  Other socio economic analysis (SEA) issues | **Comment:**  The Swedish Medical Products Agency wants to emphasize the importance that the restriction report includes a clear conformation for competent authorities to perform in market surveillance of microplastic in cosmetic products. We would also like to express concerns regarding inadequate in market surveillance if the legal requirements are unclear, for e.g. in the case of unclear description of what and when something is a microplastic at different stages (from production to the final product).  **Answer to specific info request 3:**  In medical devices, microplastics can be intentionally added with properties essential for the function of the device. The amount can be far above the proposed concentration limit of 0.01%, as described in Annex XV restriction report, proposal for a restriction, version 1.1., March 20, 2019.  **Answer to specific info request 4:**  The Swedish Medical Products Agency supports initiatives that are taken to meet the global climate goals. The Swedish Medical Products Agency also supports a derogation for medical devices and in vitro diagnostic medical devices.  However, The Swedish Medical Products Agency believes that the consequences of the proposal on Medical Devices and In vitro diagnostic medical devices need to be further explored, especially related to consequences on patient safety, socio-economic impacts and time for implementation. The proposal can lead to shortage of products and put patients at risk.  The socio-economic impact presented by the Dossier Submitter appears to be based on a limited number of products used in professional settings. Despite lack of access to a complete list of products containing microplastic, the summary of products and their uses appear to be incomplete.  The Swedish Medical Products Agency has noticed that CE-labelled absorbance products such as incontinence products are not mentioned in the documentation on the proposed restriction. Such absorbance products can contain superabsorbent microplastics. This product category is subsidized by the Swedish government and are not only used by professionals under controlled conditions. These products are high volume products, both used by professionals and other users, at both hospitals and in the homes of patients. Today, waste from such products are typically handled as household waste. In Sweden the majority of household wastes are incinerated. Still, requirements on incineration or deposition as hazardous waste management treatment of such waste can have impact on the health care system, including patient safety.  The Dossier Submitter suggests that the use and release shall be monitored and reported, and that compliance can be monitored at member state level by reviewing PSUR.  According to article 86 in the medical device regulation (2017/745), “manufacturers of class IIa, class IIb and class III devices shall prepare a periodic safety update report (‘PSUR’). Manufacturers of class IIb and class III devices shall update the PSUR at least annually and class IIa devices shall update the PSUR when necessary and at least every two years. For class III devices or implantable devices, manufacturers shall submit PSURs by means of the electronic system. For class I, IIa and IIb devices, manufacturers shall make PSURs available to the notified body involved in the conformity assessment and, upon request, to competent authorities.” This will increase the burden on notified bodies and competent authorities. The be noticed, an electronic system is still not in place and the majority of the PSURs are not going to be actively submitted to this system and would have to be requested by the competent authorities. Also, the environmental aspects of devices are not covered by regulation 2017/745, in particular the provisions on PSURs, and this would be an additional demand put on the manufacturers based on other legislation. Hence, the feasibility to monitor and report requires further attention.  The Dossier Submitter suggests that medical devices and in vitro diagnostic products can adapt to the regulation within 2 years, i.e. implement technical means where microplastics would be contained throughout their use and incinerated at the end of their life-cycle and update labels, SDS, IFU to provide enough instructions to prevent release to the environment. Even such seemingly non-intrusive modifications may entail scrutiny by notified bodies, for the devices that require a certificate. The Swedish Medical Products Agency believe that this is probably a too short implementation period and suggest that the implementation time for updates of information should be decided when all processes for MDR and IVDR are in place. Competent authorities, notified bodies and companies are working hard to adapt to the new requirements. As of today, all processes are not yet in place. Additionally, microplastics are most often added to the products with purpose to provide unique functions. Such products can be advanced. A transition time of 2 years to replace such product with non-microplastic solutions is for most products more than a challenge, even modifying product to contain microplastics throughout their use can be challenging. Research and development achievements are probably required. Such technical means most likely require more than 2 years implementation time.  The Swedish Medical Products Agency highly recommend that the consequences of the proposal for Medical Devices and In vitro diagnostic products are further evaluated to avoid shortage of products and that patient’s safety are compromised.  **Answer to specific info request 5:**  Regulation (EC) No 1223/2009 on cosmetic products states in article 3 that “a cosmetic product made available on the market shall be safe for human health when used under normal or reasonably foreseeable conditions of use”. The safety of a cosmetic product is demonstrated by ensuring that a cosmetic product has undergone a safety assessment (which, among other things, must take systemic -and local toxicity into account).  The Swedish Medical Products Agency wants to highlight concerns regarding the risk to human health if the transitional periods for alternative ingredients to microplastics are too short (for other rinse-off and leave on cosmetic products). Also, the risk to human health if alternatives to microplastics are introduced too quickly.  Animal testing is strictly prohibited for finished cosmetic products and ingredients exclusively used in cosmetics or specifically carried out in the context of the EU cosmetic regulation. Since alternative methods for systemic toxicity is at current date not available, implications for the development of new ingredients are at place. Abundant data from tests of good quality are needed for the safety assessment, to ensure that a cosmetic product is safe for human health under normal condition of use.  However, for alternatives to microplastic ingredients that are already available on the market (and already assessed as safe), a transitional period of 4-6 years could be appropriate. |
| Vereniging Sport en Gemeenten (National Authority, Netherlands) | 2019/05/17  **Content:**  Information on costs;Information on benefits;Other socio economic analysis (SEA) issues;Transitional period;Request for exemption  **Attachment:**   | | |
|  |  |  |  |

**References**

ECHA, 2019, General Comments and answers to specific information requests, Helsinki: European Chemicals Agency, Link: <https://echa.europa.eu/registry-of-restriction-intentions/-/dislist/details/0b0236e18244cd73> - accessed 28-10-2019
